# Phylogenomics studies and molecular markers reliably demarcate genus *Pseudomonas sensu stricto* and twelve other *Pseudomonadaceae* species clades representing novel and emended genera

**DOI:** 10.3389/fmicb.2023.1273665

**Published:** 2024-01-05

**Authors:** Bashudev Rudra, Radhey S. Gupta

**Affiliations:** Department of Biochemistry and Biomedical Sciences, McMaster University, Hamilton, ON, Canada

**Keywords:** *Pseudomonas* classification, phylogenomic and comparative genomic analyses, conserved signature indels (CSIs), molecular markers specific for *Pseudomonas* species clades/groups, proposals for reclassification of *Pseudomonas* species into novel genera

## Abstract

Genus *Pseudomonas* is a large assemblage of diverse microorganisms, not sharing a common evolutionary history. To clarify their evolutionary relationships and classification, we have conducted comprehensive phylogenomic and comparative analyses on 388 *Pseudomonadaceae* genomes. In phylogenomic trees, *Pseudomonas* species formed 12 main clusters, apart from the “Aeruginosa clade” containing its type species, *P. aeruginosa*. In parallel, our detailed analyses on protein sequences from *Pseudomonadaceae* genomes have identified 98 novel conserved signature indels (CSIs), which are uniquely shared by the species from different observed clades/groups. Six CSIs, which are exclusively shared by species from the “Aeruginosa clade,” provide reliable demarcation of this clade corresponding to the genus *Pseudomonas sensu stricto* in molecular terms. The remaining 92 identified CSIs are specific for nine other *Pseudomonas* species clades and the genera *Azomonas* and *Azotobacter* which branch in between them. The identified CSIs provide strong independent evidence of the genetic cohesiveness of these species clades and offer reliable means for their demarcation/circumscription. Based on the robust phylogenetic and molecular evidence presented here supporting the distinctness of the observed *Pseudomonas* species clades, we are proposing the transfer of species from the following clades into the indicated novel genera: Alcaligenes clade – *Aquipseudomonas* gen. nov.; Fluvialis clade – *Caenipseudomonas* gen. nov.; Linyingensis clade – *Geopseudomonas* gen. nov.; Oleovorans clade – *Ectopseudomonas* gen. nov.; Resinovorans clade – *Metapseudomonas* gen. nov.; Straminea clade – *Phytopseudomonas* gen. nov.; and Thermotolerans clade – *Zestomonas* gen. nov. In addition, descriptions of the genera *Azomonas*, *Azotobacter*, *Chryseomonas, Serpens*, and *Stutzerimonas* are emended to include information for the CSIs specific for them. The results presented here should aid in the development of a more reliable classification scheme for *Pseudomonas* species.

## Introduction

Genus *Pseudomonas* (Migula, 1894) is a large assemblage of motile, rod-shaped, aerobic, non-spore forming, Gram-negative bacteria, generally containing one or more polar flagella that assist in their movement (Palleroni, 2005, 2015). The members of this genus presently contain >300 species with validly published names (Parte et al., 2020), and they span enormous genetic and metabolic diversity, inhabiting diverse niches and environments including soil, water, plants and animal tissues (Peix et al., 2009; Palleroni, 2015). Its members include species which are opportunistic pathogens of humans, animals, and plants, and other species of economic and ecological significance (Palleroni, 2005; Lund-Palau et al., 2016; Winsor et al., 2016; Xin et al., 2018; Rossi et al., 2021). The best studied species from this genus, which is also its nomenclature type (Migula, 1894; Skerman et al., 1980), is *Pseudomonas aeruginosa*, which is an opportunistic human pathogen capable of causing a wide array of life-threatening acute and chronic diseases (Stover et al., 2000; Planquette et al., 2013). Despite the clinical and environmental importance of *Pseudomonas* species, evolutionary relationships among the members of this genus are not clearly understood (Anzai et al., 2000; Peix et al., 2009; Palleroni, 2015; García-Valdés and Lalucat, 2016; Jun et al., 2016; Passarelli-Araujo et al., 2022). In different phylogenetic and genomic studies on *Pseudomonas* species, members of this genus consistently form multiple clades, which are unrelated to each other (i.e., not evolved from a common ancestor) (Peix et al., 2009; Gomila et al., 2015; Jun et al., 2016; Hesse et al., 2018; Peix et al., 2018; Rudra and Gupta, 2021; Saati-Santamaría et al., 2021; Lalucat et al., 2022). Additionally, in these trees, species from several genera including *Azomonas*, *Azotobacter* and *Chryseomonas* branch in between *Pseudomonas* species, making this genus polyphyletic (Jun et al., 2016; Hesse et al., 2018; Rudra and Gupta, 2021; Saati-Santamaría et al., 2021; Lalucat et al., 2022). In recent work, a large number of *Pseudomonas* species, which generally branched outside the main cluster of *Pseudomonas* species, have been reclassified into several novel genera (*viz. Atopomonas, Halopseudomonas* and *Stutzerimonas*) (Rudra and Gupta, 2021; Lalucat et al., 2022), or in other existing genera (*viz. Chryseomonas*, *Stenotrophomonas*, *Thiopseudomonas* and *Xanthomonas*) (Holmes et al., 1987; Rudra and Gupta, 2021; Saati-Santamaría et al., 2021).

Importantly, in all constructed phylogenomic trees, the type species *P. aeruginosa*, along with a limited number of other species, forms a distinct clade referred to as the “Aeruginosa clade” (Jun et al., 2016; Hesse et al., 2018; Peix et al., 2018; Rudra and Gupta, 2021; Saati-Santamaría et al., 2021; Lalucat et al., 2022; Passarelli-Araujo et al., 2022). The remainder (>95%) of the *Pseudomonas* species group into 12–18 main clusters, some of which are referred to as the Alcaligenes, Anguilliseptica, Flexibilis, Fluorescens, Kuykendallii, Linyingensis, Lutea, Massiliensis, Oleovorans, Oryzihabitans, Pertucinogena, Putida, Resinovorans, Rhizosphaerae, Straminea, Stutzeri and Syringae clades, named after one of the species from each of these clusters (Palleroni, 2015; Hesse et al., 2018; Peix et al., 2018; Girard et al., 2021; Rudra and Gupta, 2021; Saati-Santamaría et al., 2021; Lalucat et al., 2022). Species from the Pertucinogena and Stutzeri clusters were recently reclassified into the genera *Halopseudomonas* and *Stutzerimonas*, respectively (Rudra and Gupta, 2021; Lalucat et al., 2022). Of these species’ clades, according to the Code governing the nomenclature of Prokaryotes (Oren et al., 2023), the “Aeruginosa clade,” which contains the type species *P. aeruginosa*, constitute the genus *Pseudomonas sensu stricto*. It is generally recognized that the species from clades other than the “Aeruginosa clade,” should be reclassified into novel genera (Hesse et al., 2018; Peix et al., 2018; Girard et al., 2021; Rudra and Gupta, 2021; Lalucat et al., 2022; Passarelli-Araujo et al., 2022). This task requires that the boundaries of different *Pseudomonas* species clades, including the “Aeruginosa clade,” are reliably demarcated so that any proposed reclassification is stable. Different *Pseudomonas* species clades are presently identified primarily based on the clustering of species in phylogenetic trees. However, the numbers of observed species clusters as well as the species grouping within them often vary in different phylogenetic studies (Hesse et al., 2018; Girard et al., 2021; Rudra and Gupta, 2021; Lalucat et al., 2022; Rudra et al., 2022), which makes it difficult to reliably demarcate the boundaries of these clades.

The availability of whole genome sequences is enabling construction of more reliable phylogenetic trees based on large dataset of genes/proteins (Parks et al., 2018). Additionally, the genome sequences also provide an important resource for identification of novel molecular markers, such as conserved signature indels (CSIs), which are uniquely shared characteristics of different monophyletic clades of organisms. Due to their clade specificities, these novel molecular synapomorphies are providing robust means for the demarcation of different observed species clades/taxa in molecular terms (Gupta et al., 2013; Gupta, 2014; Adeolu et al., 2016; Gupta et al., 2020). The use of these markers in conjunction with phylogenomic analyses has recently led to the development of a reliable classification scheme for members of the highly polyphyletic genus *Bacillus* (Gupta et al., 2020). Genome sequences are now available for >300 *Pseudomonas* species in the NCBI genome database^1^ (Sayers et al., 2019). With the objective of clarifying evolutionary relationships and classification of *Pseudomonas* species, we have conducted comprehensive phylogenomic and molecular marker-based studies on their genome sequences. In two genome scale phylogenetic trees constructed in this study, *Pseudomonas* species formed approximately 13 main clades, like those seen in earlier work (Hesse et al., 2018; Girard et al., 2021; Lalucat et al., 2022; Passarelli-Araujo et al., 2022). In parallel, our detailed studies on protein sequences from *Pseudomonas* genomes have identified 98 novel CSIs which are unique characteristics of the species from different observed clades. Based on these CSIs, species from the “Aeruginosa clade” (i.e., genus *Pseudomonas sensu stricto*), 10 other *Pseudomonas* species clades, and the genera *Azomonas* and *Azotobacter*, can now be reliably demarcated based on multiple uniquely shared molecular characteristics. Based on the strong evidence obtained from our phylogenomic studies and identified molecular markers, we are proposing the reclassification of *Pseudomonas* species from the following clades, *viz.* Alcaligenes, Fluvialis, Linyingensis, Oleovorans, Resinovorans, Straminea, and Thermotolerans, into seven novel genera. In addition, we are also emending the descriptions of the genera *Azomonas, Azotobacter, Chryseomonas*, *Serpens* and *Stutzerimonas* to include information for the diagnostic CSIs for these genera.

## Methods

### Construction of phylogenetic trees

Genome sequences were downloaded from the NCBI for 342 named *Pseudomonas* species and 46 sequences from other *Pseudomonadaceae* genera available as of December 16, 2022, in the database. Each species is represented in the tree by a single genomic sequence, which is generally of the type strain, when available. Based on these genome sequences, a rooted phylogenetic tree was constructed based on concatenated sequences of 118 conserved proteins that are a part of the phyloeco set for the class *Gammaproteobacteria* (Wang and Wu, 2013) (listed in Supplementary Table S1). Genome sequences for *Moraxella bovoculi* and *M. bovis* were included in this dataset for rooting purposes. Another comprehensive phylogenetic tree was constructed based on the core proteins from the genomes of *Pseudomonadaceae* species. This latter tree was based on genome sequences for 174 species, which included most of the species from the other main clades of *Pseudomonas* species, but only 41 divergent species from the Fluorescens superclade (lineage). Trees were constructed using an internally developed pipeline described in earlier work (Adeolu et al., 2016; Gupta et al., 2020; Rudra and Gupta, 2021; Saini and Gupta, 2021). Briefly, the CD-HIT program (Li and Godzik, 2006; Fu et al., 2012) was used to identify protein families (or homologs of different proteins) where the proteins were present in at least 80% of the genomes in the dataset and they shared at least 50% of sequence length and identity. The Clustal Omega program (Sievers et al., 2011) was then used to generate multiple sequence alignments (MSA) of the proteins. These MSAs were converted into profile Hidden Markov Models (HMMs) using HMMer 3-1b2 (Eddy, 2011), which were then used to search for other members of the protein families in the input genomes. These analyses identified 1,503 protein families meeting the stated criteria (also listed in Supplementary Table S1). The sequence alignments of these proteins were trimmed using TrimAl program (Capella-Gutiérrez et al., 2009) to remove poorly aligned sections prior to their concatenation. The concatenated sequence alignment for the phyloeco set of proteins for *Gammaproteobacteria* was created similarly using the published profile HMMs for these proteins (Wang and Wu, 2013). The concatenated sequence alignments used for the construction of phyloeco and the core genome trees consisted of 42,362 and 494,143 amino acid (aa) positions, respectively. Using these alignments, maximum likelihood (ML) trees were initially constructed using FastTree 2 (Price et al., 2010) with the Whelan and Goldman (2001) model of protein sequence evolution. The resulting trees were optimized with RAxML 8 (Stamatakis, 2014) and to obtain the Shimodaira-Hasegawa (SH) statistical support values, which are similar to the bootstrap scores, for different nodes. The trees were labeled and formatted using MEGA X (Kumar et al., 2018). The percentage of conserved proteins (POCP) and average amino acid identity (AAI) for different pairs of genomes were calculated as described by Thompson et al. (2013) and Qin et al. (2014).

### Identification of conserved signature indels

Identification of CSIs was carried out by similar procedures as described in earlier work (Gupta, 2014, 2016; Gupta et al., 2020). Briefly, local BLASTp searches were carried out on protein sequences from the genomes of several *Pseudomonas* species representing different clades of interest and other outgroup species. Based on these BLAST searches, sequences of high scoring homologs (E value <1e-20) of different proteins were retrieved for several species (generally between 4 to 12) from the group of interest, and 10–15 species from other *Pseudomonas* clades or other *Pseudomonadaceae* genera. Multiple sequence alignments for the proteins were created using Clustal X 2.1 program (Jeanmougin et al., 1998). Alignments were visually examined for insertions or deletions of fixed length that were present in conserved regions (i.e., flanked on both sides by minimally 5–6 conserved aa residues in the neighboring 40–50 aa), and which were only found in the *Pseudomonas* species from the clade of interest. The indels which were not present in conserved regions were not further considered. The query sequences consisting of the conserved indels and their flanking 30–40 aa on each side were subjected to a second BLASTp search against the NCBI nr database and the top 250–500 hits were evaluated to determine the group specificities of the CSIs. Based on these results, indels which were specific for different clades of *Pseudomonas* were formatted using the SIG_CREATE and SIG_STYLE programs (Gupta, 2014, 2016). Due to space constraints, sequence information is shown for only a limited number of species in the main figures. However, unless otherwise indicated the CSIs reported here are specifically found in different named *Pseudomonas* species from the indicated groups. More detailed information for different CSIs is provided in the Supplemental Data files.

## Results

### Phylogenomic analyses of *Pseudomonas* and related species

**Figure 1:**
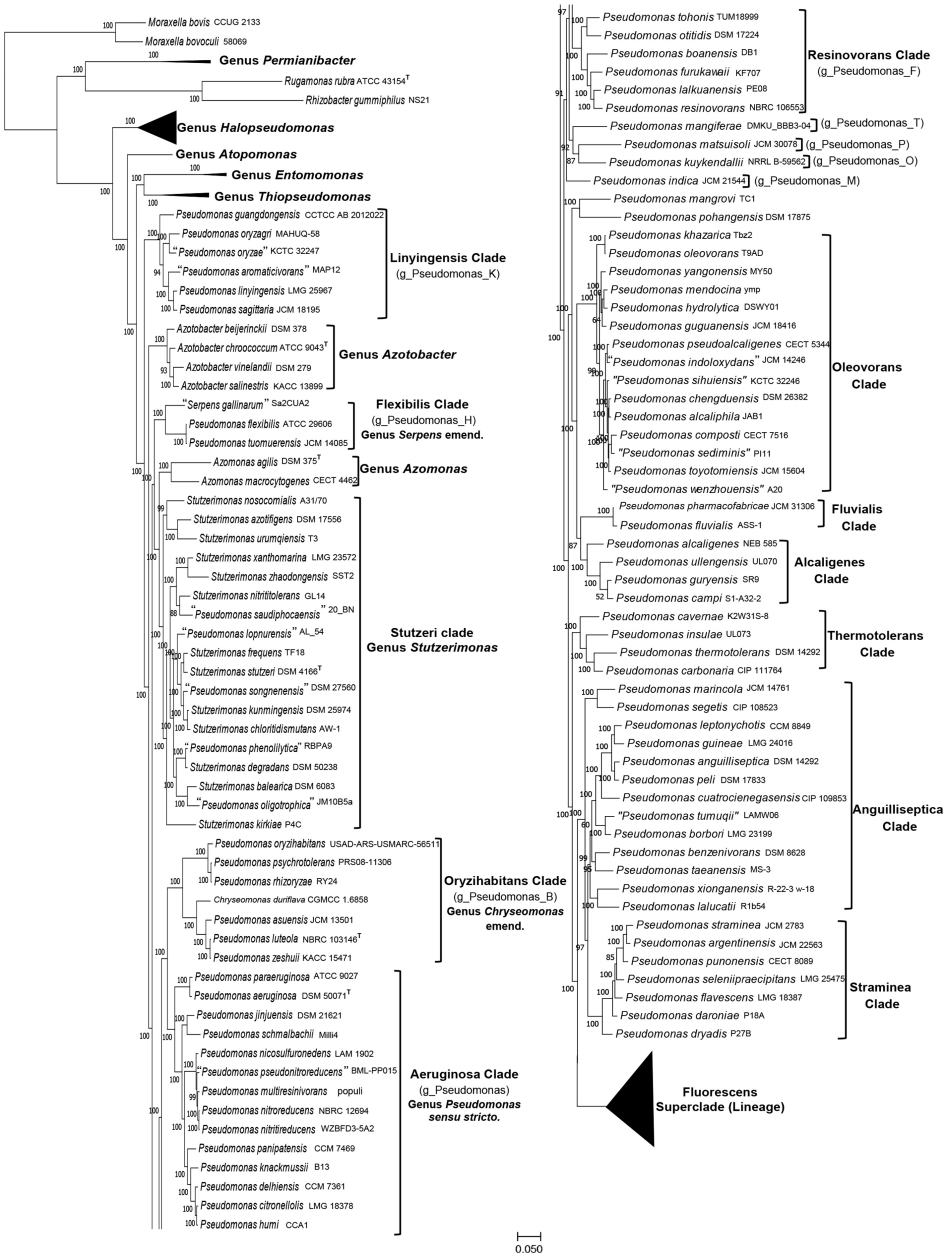
A maximum-likelihood tree for 388 genome-sequenced *Pseudomonadaceae* species based on concatenated sequences for 118 conserved proteins. The tree is shown into two halves, and species from the Fluorescens superclade (lineage) are compressed, so that the species compositions of other clades of interest can be seen. The species clades of interest are demarcated and labeled with the commonly used names and in some cases with the GTDB taxon assignment for the clade.

To understand the interspecies relationships among different *Pseudomonadaceae* species whose genomes were available in the NCBI as of December 16, 2022, two genome-scale phylogenetic trees were constructed. The first of these trees shown in Figure 1 (Supplementary Figure S1), which will be referred to as the phyloeco tree, is based on concatenated sequences for 118 conserved proteins, which comprise the phyloeco set for the class *Gammaproteobacteria* (Wang and Wu, 2013). Another comprehensive tree constructed is a core genome (protein) tree based on 1,503 proteins which are shared by at least 80% of the input *Pseudomonadaceae* species. This latter tree included only representative species (41) from the Fluorescens superclade (lineage), which is not the focus of this study. In both constructed trees, most observed nodes are supported with 100% SH values (like bootstrap scores) indicating that the observed evolutionary relationships are reliable.

The overall branching and grouping of *Pseudomonadaceae* species in different clusters in both the phyloeco (Figure 1) and the core protein tree (Supplementary Figure S2) is nearly identical, and it is similar to that observed in our earlier work (Rudra and Gupta, 2021), and other phylogenetic studies (Gomila et al., 2015; Hesse et al., 2018; Peix et al., 2018; Lalucat et al., 2020; Girard et al., 2021; Lalucat et al., 2022; Passarelli-Araujo et al., 2022). In both these trees, *Pseudomonas* species formed several distinct clades/groups, and species from the genera *Azomonas* and *Azotobacter* consistently branched between them (Hesse et al., 2018; Rudra and Gupta, 2021; Lalucat et al., 2022; Passarelli-Araujo et al., 2022). Additionally, species from the two recently proposed genera *Stutzerimonas* and *Chryseomonas* also branched within other *Pseudomonas* species, thus further contributing to the polyphyly of this genus. We have labeled different *Pseudomonas* species clades in Figure 1 and Supplementary Figure S2 by their commonly used clade/group names (Hesse et al., 2018; Girard et al., 2021; Lalucat et al., 2022). One distinct clade observed in all constructed trees is the “Aeruginosa clade,” which contains the type species *P. aeruginosa* and 13 other *Pseudomonas* species. As this clade contains the type species of the genus *Pseudomonas*, we have labeled it as the “Genus *Pseudomonas sensu stricto*.” Other species’ clades observed and labeled in Figure 1 (Supplementary Figure S2) include: the Alcaligenes, Anguilliseptica, Azomonas, Azotobacter, Flexibilis, Fluvialis, Linyingensis, Oleovorans, Oryzihabitans, Resinovorans, Straminea, Stutzeri (*Stutzerimonas*), Thermotolerans, and Fluorescens superclade (lineage).The Genome Taxonomy Database (GTDB),^2^ based on phylogenetic analysis of 120 ubiquitously conserved proteins, now provides an important resource for taxonomic inferences (Parks et al., 2018). The GTDB refers to the “Aeruginosa clade” as the genus *Pseudomonas* whereas most of the other observed species clades are referred to as distinct genera denoted by designations such as g_*Pseudomonas*_B, g_*Pseudomonas*_K, etc., which are also indicated in the tree in Figure 1.

Of these observed clades, the Fluorescens superclade (lineage) is the largest harboring 245 *Pseudomonas* species. It is separated from all other *Pseudomonas* species by a long branch in both constructed trees (Figure 1; Supplementary Figure S2). Due to the large number of species present in this clade, it is shown in a compressed form in Figure 1. However, detailed information for species comprising this clade is provided in Supplementary Figure S1. The Fluorescens superclade (lineage) is made up of multiple distinct clades and subclades (see Supplementary Figure S1) (Hesse et al., 2018; Peix et al., 2018; Lalucat et al., 2020; Rudra and Gupta, 2021; Lalucat et al., 2022). However, all species grouping within the Fluorescens superclade (lineage) are part of the GTDB taxon “g_*Pseudomonas*_E.” Although the *Pseudomonas*_E cluster in GTDB also encompasses the Alcaligenes, Anguilliseptica, Oleovorans and Thermotolerans clades, these clades in our phylogenomic trees (Figure 1; Supplementary Figure S1), and in several other published studies (Hesse et al., 2018; Girard et al., 2021; Lalucat et al., 2022; Passarelli-Araujo et al., 2022), branch separately from the Fluorescens superclade. This discrepancy in the branching positions of the Alcaligenes, Anguilliseptica, Oleovorans and Thermotolerans clades between the GTDB taxonomy and other phylogenomic trees, was also noted by Lalucat et al. (2022). However, in the present work, we will not be examining the evolutionary relationships of different species within the Fluorescens superclade. Besides the “Aeruginosa clade” and the Fluorescens superclade (lineage), the other clades marked in Figure 1 (Supplementary Figure S2) contain between 2–18 species. Except for the Anguilliseptica clade, which shows poor resolution and weak statistical support, all other clades in our phylogenetic trees are statistically strongly supported. Besides these species’ clades, a limited number of *Pseudomonas* species (*viz. P. indica, P. kuykendallii, P. mangiferae, P. mangrovi, P. matsuisoli* and *P. pohangensis*) are not part of any of the observed clades.

The analyzed genome sequences were also used for determination of percentage of conserved proteins (POCP) and average amino acid identity (AAI) between different pairs of genomes. The results of pairwise AAI and POCP values, for different *Pseudomonadaceae* genomes are presented in Supplementary Tables S2 and S3, respectively. Genome pairs exhibiting higher AAI or POCP values are shown by a darker shade of green/red, and different clades observed in our phylogenetic trees (Figure 1; Supplementary Figure S2) are outlined. In Table 1, we present a summary of the ranges of the AAI and POCP values for different *Pseudomonas* species clades for the ingroup and outgroup species. Based on the results in Table 1, the AAI and POCP values for species within different clades are higher (AAI values range: 0.70–1.00; POCP values range: 0.66–1.00) in comparison to these values for species from the other clades (AAI values range: 0.67–0.81; POCP values range: 0.42–0.77), which is an expected result. However, based on the AAI and POCP values (Table 1), only species from the Alcaligenes, Azotobacter, Flexibilis, Fluvialis, Lingyingensis, Oleovorans and Thermotolerans clades show no overlap with species from the other clades. In contrast, these values for several other clades (*viz.* “Aeruginosa.” Anguiliiseptica, Azomonas, Oryzihabitans, Resinovorans, Straminea, Stutzeri) either show significant overlap or are very close to those from the outgroup species. Thus, based on these genome similarity indices, species from different observed *Pseudomonadaceae* clades cannot be reliably demarcated. In Table 1, the highest overlap in the AAI and POCP values between the ingroup versus outgroup species is observed for the species from Anguilliseptica clade, which also shows poor resolution and weak statistical support in the phylogenetic trees.

**Table 1:** Range of AAI and POCP values among different *Pseudomonadaceae* species clades.

Clades	AAI values		POCP values	
	Ingroup	Outgroup	Ingroup	Outgroup
“Aeruginosa clade” (*Pseudomonas sensu stricto*)	0.75–1.00	0.67–0.75	0.66–1.00	0.42–0.73
Alcaligenes clade (*Aquipseudomonas* gen. nov.)	0.83–1.00	0.69–0.79	0.79–1.00	0.49–0.75
Anguilliseptica clade	0.77–1.00	0.68–0.81	0.68–1.00	0.45–0.75
Genus *Azomonas*	0.73–1.00	0.68–0.74	0.68–1.00	0.42–0.67
Genus *Azotobacter*	0.86–1.00	0.68–0.76	0.80–1.00	0.49–0.67
Flexibilis clade (Genus *Serpens* emend.)	0.79–1.00	0.69–0.76	0.83–1.00	0.51–0.69
Fluvialis clade (*Caenipseudomonas* gen. nov.)	1.00	0.70–0.77	1.00	0.48–0.71
Linyingensis clade (*Geopseudomonas* gen. nov.)	0.82–1.00	0.69–0.75	0.69–1.00	0.49–0.67
Oleovorans clade (*Ectopseudomonas* gen. nov.)	0.88–1.00	0.67–0.81	0.75–1.00	0.43–0.77
Oryzihabitans clade (Genus *Chryseomonas* emend.)	0.71–1.00	0.67–0.72	0.70–1.00	0.47–0.67
Resinovorans clade (*Metapseudomonas* gen. nov.)	0.79–1.00	0.68–0.77	0.70–1.00	0.44–0.74
Straminea clade (*Phytopseudomonas* gen. nov.)	0.76–1.00	0.67–0.81	0.69–1.00	0.47–0.76
*Stutzeri* clade (Genus *Stutzerimonas*)	0.77–1.00	0.68–0.76	0.72–1.00	0.49–0.66
Thermotolerans clade (*Zestomonas* gen. nov.)	0.81–1.00	0.70–0.79	0.75–1.00	0.48–0.74

Detailed information regarding the pairwise AAI and POCP values for species from different clades is provided in Supplementary Tables S2 and S3.

### Identification of molecular markers demarcating/distinguishing different *Pseudomonas* species clades

Although *Pseudomonadaceae* species form similar clades in different genome scale trees (Hesse et al., 2018; Parks et al., 2018; Girard et al., 2021; Lalucat et al., 2022; Figure 1; Supplementary Figure S2), branching of species in phylogenetic trees is influenced by large numbers of variables (Gupta, 1998; Baldauf, 2003; Felsenstein, 2004). Moreover, in phylogenetic trees for *Pseudomonas*, species from several clades are separated from each other by short branches (Figure 1; Supplementary Figure S2), which makes it difficult to reliably determine their boundaries. The POCP and AAI values for several clades also overlap or are very close to the other species (Table 1), thus they do not permit reliable determination of the boundaries of these clades. Hence, it was important to discover other reliable means for the demarcation of these clades. Molecular synapomorphies consisting of CSIs in genes/proteins sequences, which are uniquely shared characteristics of species from different clades, provide important means for the demarcation of taxa of different ranks in molecular terms (Gupta, 2014; Adeolu et al., 2016; Gupta et al., 2020; Patel and Gupta, 2020; Rudra and Gupta, 2021). Hence, detailed studies were conducted on protein sequences from *Pseudomonadaceae* species to identify CSIs which are specific for different observed clades. These analyses have identified 98 novel CSIs which are specific for different *Pseudomonadaceae* clades, providing independent evidence for the genetic distinctness of these clades and affording reliable means for their demarcation. Brief descriptions of the characteristics of these CSIs are given below.

### CSIs specific for the “Aeruginosa clade”

**Figure 2:**
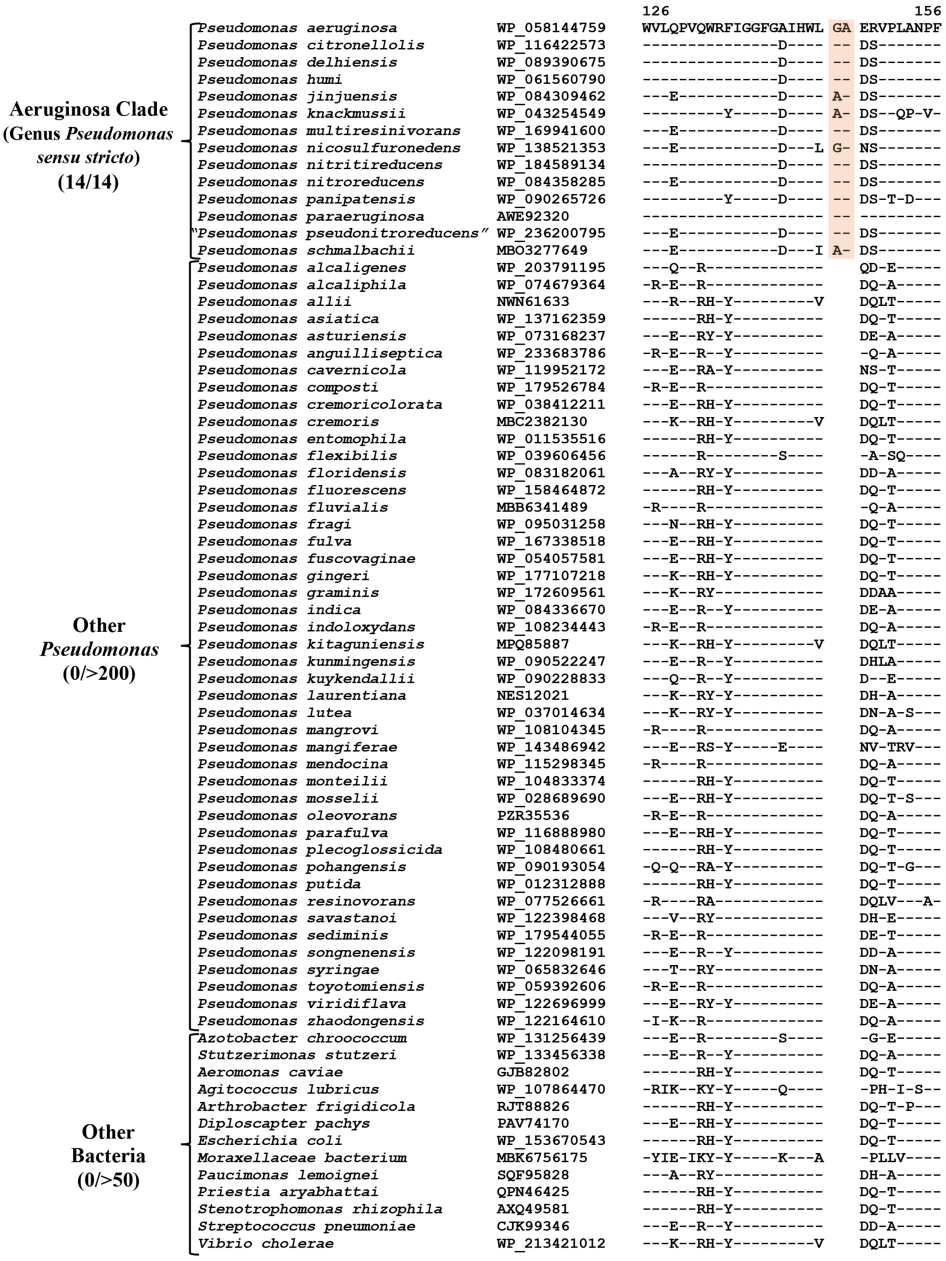
Partial sequence alignment of the HugZ family protein showing a two aa insertion (highlighted) that is exclusively present in all members of the “Aeruginosa clade.” The dashes (−) in this and all other sequence alignments indicate identity with the amino acids on the top line. Accession numbers for different sequences are indicated in the second column and the numbers at the top indicate the position of this sequence in the protein sequences. Detailed sequence information for this CSI and five other CSIs specific for this clade is provided in Supplementary Figures S3–S8.

The “Aeruginosa clade” representing the genus *Pseudomonas sensu stricto*, encompasses 14 named species (*viz.*, *P. aeruginosa, P. paraeruginosa, P. citronellolis, P. delhiensis, P. humi, P. jinjuensis, P. knackmussii, P. multiresinivorans, P. nicosulfuronedens, P. nitritireducens, P. nitroreducens, P. panipatensis, “P. pseudonitroreducens”* and *P. schmalbachii*) (Figure 1). Our analyses have identified six CSIs in proteins involved in different functions (Table 2), which are commonly and, in most cases, uniquely shared by different species from the “Aeruginosa clade.” Sequence information for one of these is presented in Figure 2. In the example shown, a two aa insertion (highlighted) in a conserved region of the HugZ family protein is commonly shared by all 14 species from the “Aeruginosa clade” but absent in all other *Pseudomonadaceae* species. Sequence information is shown in Figure 2 for only a limited number of species. However, more detailed information for this CSI is presented in Supplementary Figure S3. Like the CSI shown in Figure 2, we have identified five additional CSIs in other proteins which, except for an isolated occurrence, are uniquely shared by different species from the “Aeruginosa clade.” Sequence information for these CSIs is provided in Supplementary Figures S4–S8 and some of their characteristics are summarized in Table 2. Due to their unique shared presence in species from the “Aeruginosa clade,” genetic changes responsible for these CSIs likely occurred in a common ancestor of this clade and subsequently inherited by all members. Due to their specificities for the species from the “Aeruginosa clade,” these molecular synapomorphies provide robust means for the demarcation of this clade in molecular terms.

**Table 2:** Summary of CSIs specific for the “Aeruginosa,” Alcaligenes, and Oleovorans clades.

Protein name	Accession no	Figure number	Indel size	Indel location	Specificity
HugZ family protein	WP_058144759	Figure 2; Supplementary Figure S3	2 aa Ins	126–156	“Aeruginosa clade” (*Pseudomonas sensu stricto*)
TetR family transcriptional regulator	WP_162953821	Supplementary Figure S4	1 aa Ins	68–104	
Transglutaminase family protein^#^	WP_089389603	Supplementary Figure S5	1aa Ins	39–83	
Multidrug efflux RND transporter permease subunit	WP_038803172	Supplementary Figure S6	2 aa Ins	233–269	
Alginate O-acetyltransferase^#^	PXC05278	Supplementary Figure S7	1 aa Del	24–61	
23S rRNA (cytidine(2498)-2’-O)-methyltransferase RlmM^#^	OVZ41066	Supplementary Figure S8	1 aa Ins	54–98	
Ferric iron uptake transcriptional regulator	WP_110680887	Figure 3A; Supplementary Figure S9	2 aa Ins	6–52	Alcaligenes clade (*Aquipseudomonas* gen. nov.)
DUF1853 family protein	WP_061903990	Supplementary Figure S10	1 aa Del	55–93	
SCP2 sterol-binding domain-containing protein	WP_076424264	Supplementary Figure S11	1 aa Del	55–98	
Hypothetical protein^$^	GIZ66354	Supplementary Figure S12	4 aa Del	125–167	
Zinc ABC transporter substrate-binding protein	WP_061902889	Supplementary Figure S13	4 aa Del	261–297	
Hybrid sensor histidine kinase/response regulator	WP_203791762	Supplementary Figure S14	2 aa Del	130–170	
Cysteine synthase A	WP_150609166	Figure 3B; Supplementary Figure S15	1 aa Del	119–160	Oleovorans clade (*Ectopseudomonas* gen. nov.)
Lipopolysaccharide export system permease protein^$^	NYF64131	Supplementary Figure S16	1 aa Ins	19–61	
Succinylglutamate desuccinylase^$^	WP_125875007	Supplementary Figure S17	1 aa Ins	121–164	
Fe2 + −dependent dioxygenase	WP_206407640	Supplementary Figure S18	4 aa Del	124–155	
Osmoprotectant NAGGN system M42 family peptidase^#^	WP_206408901	Supplementary Figure S19	3 aa Ins	46–85	

^#^The CSIs listed here are specific for the indicated clades of bacteria, apart from an isolated exception present in some CSIs (#; see Supplementary Figures for details). ^$^The protein homologs were not found in some species.

### CSIs specific for the Alcaligenes clade

**Figure 3:**
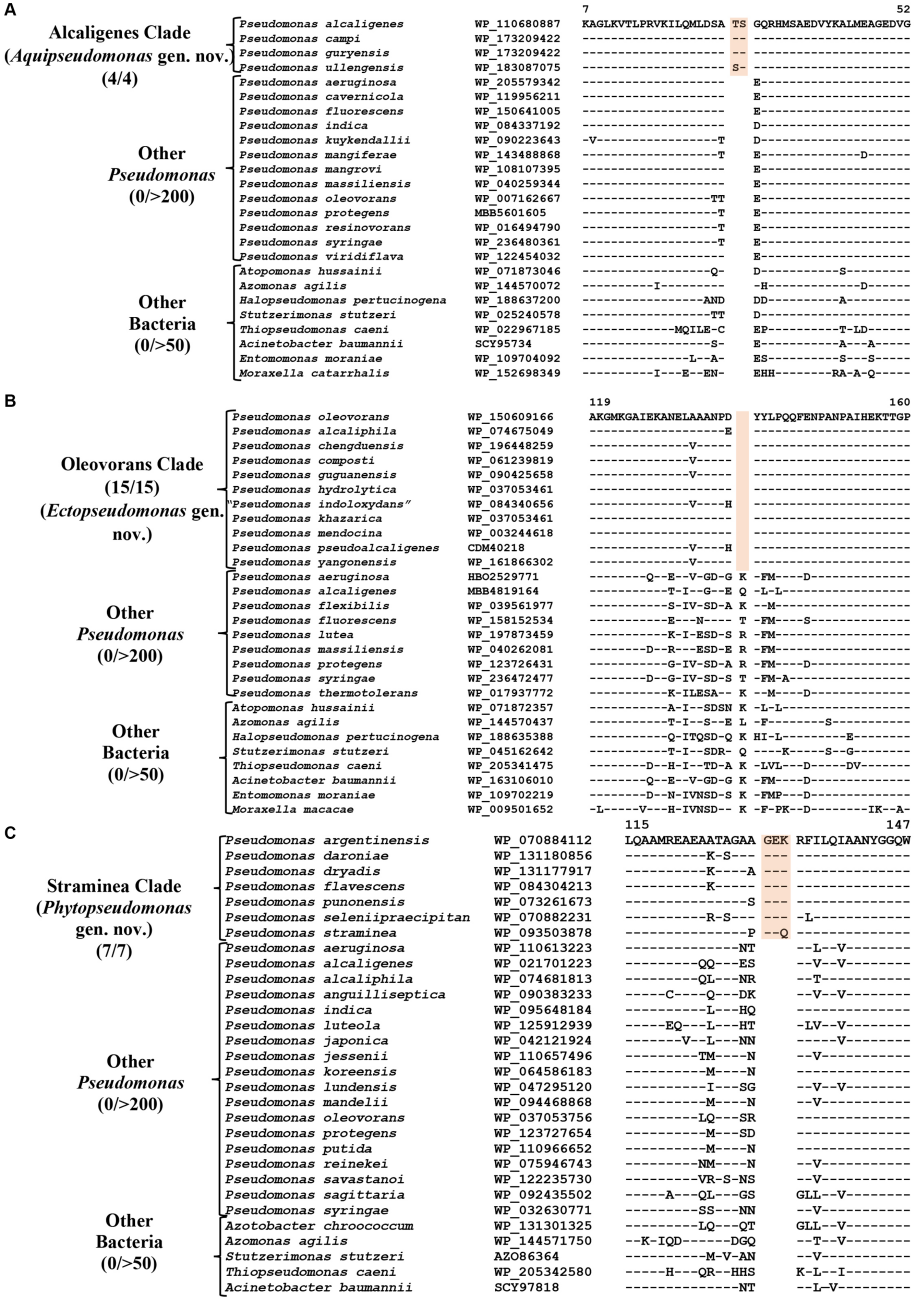
Partial sequence alignments of **(A)** Ferric iron uptake protein showing a two aa insertion within a conserved region that is a distinctive characteristic of all members of the Alcaligenes clade. **(B)** A one aa deletion in a conserved region of the protein Cysteine synthase A which is specific for the species from Oleovorans clade. **(C)** A three aa insertion within a conserved region in the protein Di-trans, poly-cis-decaprenylcistransferase, specific for the species from the Straminea clade. Detailed sequence information for these CSIs along with other CSIs specific for these clades are provided in Supplementary Figures S9–S31.

*P. alcaligenes* was indicated to branch separately from other clades in earlier studies (Hesse et al., 2018; Girard et al., 2021; Lalucat et al., 2022). In our phylogenetic trees (Figure 1; Supplementary Figure S2), three recently identified species (*viz.*, *P. campi, P. guryensis, P. ullengensis*) also reliably grouped with *P. alcaligenes*. Our analysis has identified six novel CSIs, which in most cases are exclusively shared by all four species from the Alcaligenes clade. Sequence information for one of these CSIs is presented in Figure 3A, where a two aa insertion in the protein ferric iron uptake transcriptional regulator is exclusively present in all four species from the Alcaligenes clade. Five additional CSIs in other proteins are also generally specific for the species from this clade. Detailed sequence information for these six CSIs is provided in Supplementary Figures S9–S14, and some of their characteristics are listed in Table 2. The identified CSIs provide reliable means for the demarcation of species from the Alcaligenes clade in molecular terms and we are proposing their transfer into *Aquipseudomonas* gen. nov.

### CSIs specific for the Oleovorans clade

Oleovorans clade is a strongly supported clade consisting of 15 *Pseudomonas* species (*viz.*, *P. alcaliphila, P. chengduensis, P. composti, P. guguanensis, P. hydrolytica, “P. indoloxydans,” P. khazarica, P. mendocina, P. oleovorans, P. pseudoalcaligenes, “P. sediminis,” “P. sihuiensis,” P. toyotomiensis, “P. wenzhouensis,” P. yangonensis*), which reliably group together in the constructed phylogenetic trees (Figure 1; Supplementary Figure S2). The genetic distinctness of this clade is also independently supported by five novel identified CSIs which, excepting an isolated occurrence, are uniquely shared by all species from this clade. Sequence information for one of these CSIs is provided in Figure 3B, where a one aa deletion (highlighted), within a conserved region of the protein cysteine synthase A, is exclusively shared by all species from the Oleovorans clade. More detailed sequence information for this CSI and four additional CSIs specific for the Oleovorans clade is provided in Supplementary Figures S15–S19 and some of their characteristics are listed in Table 2. Based on the strong evidence presented here demonstrating the distinctness of species from the Oleovorans clade, we are proposing the transfer of these species into *Ectopseudomonas* gen. nov.

In addition to the species with validly published names, Oleovorans clade also encompasses four species [*viz.*, *“P. indoloxydans”* (Manickam et al., 2008), *“P. sediminis”* (Behera et al., 2018), *“P. sihuiensis”* (Wu et al., 2014) and *“P. wenzhouensis”* (Zhang et al., 2021)], whose names have not been validly published. Because of their non-validly published status, new name combinations for these species are not proposed. However, in view of their reliable grouping with the Oleovorans clade, it is suggested that these species should also be recognized as members of the genus *Ectopseudomonas* with the names *“E. indoloxydans,” “E. sediminis,” “E. sihuiensis”* and *“E. wenzhouensis,”* respectively.

### CSIs specific for the Straminea clade

The Straminea clade is a strongly supported cluster encompassing seven *Pseudomonas* species (*P. argentinensis, P. daroniae, P. dryadis, P. flavescens, P. punonensis, P. seleniipraecipitans, P. straminea*) (Figure 1; Supplementary Figure S2). Species from this clade have also been found to group together in earlier studies (Hesse et al., 2018; Girard et al., 2021; Lalucat et al., 2022; Passarelli-Araujo et al., 2022). The members of this clade can be reliably distinguished from all other *Pseudomonadaceae* species by 12 novel CSIs identified in this study, which in most cases are exclusively shared by the species from this clade. Sequence information for one of these CSIs consisting of a three aa insertion in the protein Di-trans, poly-cis-decaprenylcistransferase is presented in Figure 3C. Detailed sequence information for this CSI and the 11 other CSIs specific for this clade are presented in Supplementary Figures S20–S31 and some of their characteristics are listed in Table 3. Based on the presented results showing the distinctness of this clade, we are proposing the transfer of species from this clade into *Phytopseudomonas* gen. nov.

**Table 3:** Summary of CSIs specific for members of the Straminea, Stutzeri, and Linyingensis clades.

Protein name	Accession no	Figure number	Indel size	Indel location	Specificity
Di-trans, poly-cis-decaprenylcistransferase	WP_070884112	Figure 3C; Supplementary Figure S20	3 aa Ins	110–150	Straminea clade (*Phytopseudomonas* gen. nov.)
Efflux RND transporter periplasmic adaptor subunit	WP_074886159	Supplementary Figure S21	2 aa Del	203–245	
Beta-ketoacyl-ACP synthase III	WP_093501944	Supplementary Figure S22	1 aa Ins	233–273	
Sugar ABC transporter ATPase^&^	WP_093502557	Supplementary Figure S23	2 aa Del	26–65	
DNA polymerase III subunit alpha^&^	WP_093503860	Supplementary Figure S24	4 aa Ins	818–855	
Polyprenyl diphosphate synthase^&^	WP_093503878	Supplementary Figure S25	3 aa Ins	110–153	
Ubiquinol-cytochrome c^&^ reductase cytochrome b subunit	SFD97069	Supplementary Figure S26	5 aa Ins	65–102	
GTP diphosphokinase^&^	WP_093502677	Supplementary Figure S27	1 aa Ins	108–150	
tRNA (adenosine(37)-N6)-dimethylallyltransferase MiaA^&^.	WP_093506440	Supplementary Figure S28	5 aa Del	167–203	
Transporter substrate-binding domain-containing protein^#^	WP_093500877	Supplementary Figure S29	1 aa Ins	112–152	
YIP1 family protein^#^	WP_074882567	Supplementary Figure S30	1 aa Del	48–87	
Methyltransferase^#^	WP_074882425	Supplementary Figure S31	1 aa Ins	55–85	
PAS domain-containing methyl-accepting chemotaxis protein	WP_084903134	Figure 4A; Supplementary Figure S32	1 aa Ins	83–127	Stutzeri clade (Genus *Stutzerimonas*)
DUF1329 domain-containing protein	WP_049338638	Supplementary Figure S33	1 aa Del	115–121	
Autotransporter assembly complex protein TamA^$^	WP_084904442	Supplementary Figure S34	1 aa Del	112–147	
2-octaprenyl-3-methyl-6-methoxy-1,4-benzoquinol hydroxylase^#^	WP_014818653	Supplementary Figure S35	1 aa Ins	105–149	
Rhomboid family intramembrane serine protease^#^	WP_218422476	Supplementary Figure S36	2 aa Ins	237–265	
RnfABCDGE type electron transport complex subunit D^#^	WP_106442915	Supplementary Figure S37	1 aa Del	165–212	
16S rRNA (uracil(1498)-N(3))-methyltransferase^$^	WP_221292728	Supplementary Figure S38	1 aa Del	142–170	
UDP-N-acetylmuramoyl-L-alanine--D-glutamate ligase	WP_090305970	Figure 4B; Supplementary Figure S39	5 aa Ins	372–421	Linyingensis clade (*Geopseudomonas* gen. nov.)
Septal ring lytic transglycosylase RlpA family protein	WP_090305376	Supplementary Figure S40	1 aa Ins	272–311	
Dephospho-CoA kinase	WP_090305710	Supplementary Figure S41	1 aa Ins	107–142	
ATP-dependent zinc metalloprotease FtsH	WP_090308457	Supplementary Figure S42	1 aa Del	413–445	
Penicillin-binding protein 1A	WP_090307056	Supplementary Figure S43	1 aa Ins	232–282	
bifunctional [glutamate--ammonia ligase]-adenylyl-L-tyrosine phosphorylase/[glutamate--ammonia-ligase] adenylyltransferase	WP_090307131	Supplementary Figure S44	1 aa Ins	672–718	
Repressor LexA	WP_090307764	Supplementary Figure S45	2 aa Ins	166–201	
Malate dehydrogenase	WP_090312804	Supplementary Figure S46	1 aa Ins	131–162	
Uridylyltransferase	WP_090313706	Supplementary Figure S47	1 aa Ins	629–676	
CHAD domain-containing protein^$^	WP_090307991	Supplementary Figure S48	3 aa Del	166–203	
Protocatechuate 3,4-dioxygenase subunit alpha^$^	WP_090309801	Supplementary Figure S49	4 aa Del; 1 aa Del	109–141	
Secretin	WP_090310373	Supplementary Figure S50	1 aa Del	194–231	
CDP-6-deoxy-delta-3,4-glucoseen reductase	WP_090312664	Supplementary Figure S51	3 aa Ins	236–276	
YkgJ family cysteine cluster protein	WP_090306967	Supplementary Figure S52	1 aa Ins	9–45	
tRNA preQ1(34) S-adenosylmethionine ribosyltransferase-isomerase QueA^#^	WP_090305582	Supplementary Figure S53	2 aa Ins	145–182	

^#^Isolated exception present in some CSIs (#; see Supplementary Figures for details). ^$^The protein homologs were not found in some species. ^&^CSI is not found in *P. dryadis*, which is the deepest branching member of the clade.

### CSIs specific for the genus *Stutzerimonas*

**Figure 4:**
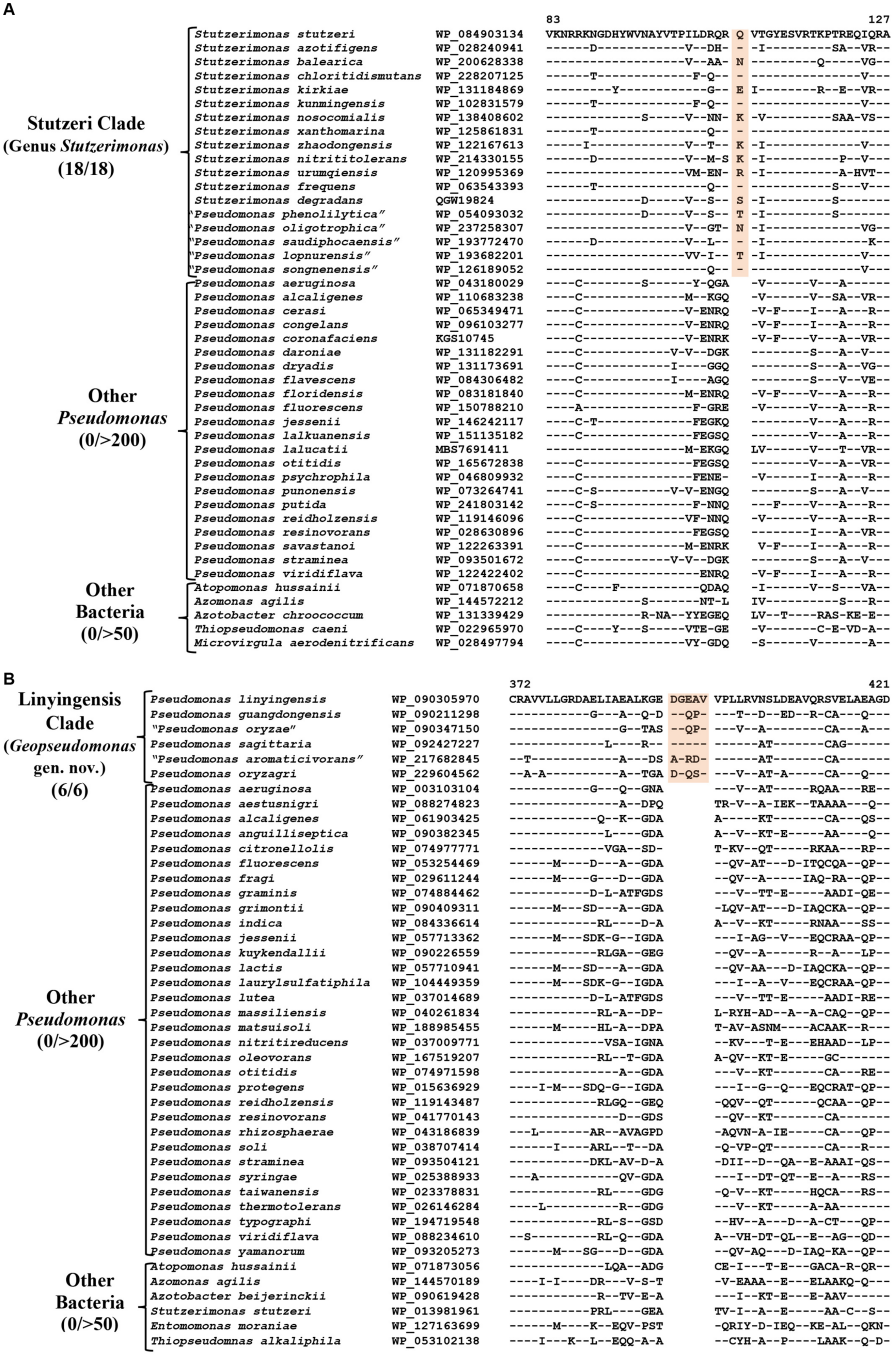
Partial sequence alignment of the protein **(A)** PAS domain containing Methyl-accepting chemotaxis protein showing a one aa insertion within a conserved region (highlighted) that is uniquely present in all members of the Stutzeri clade. **(B)** A five aa insertion within a conserved region of the protein UDP-N-acetylmuramoyl-L-alanine, which is specific for the species from Linyingensis clade. Detailed sequence information for these CSIs and other CSIs specific for Stutzeri and Linyingensis clades are provided in Supplementary Figures S32–S53.

The genus *Stutzerimonas* was recently described by Lalucat et al. (2022) by the transfer of several *Pseudomonas* species which branched distinctly in their phylogenetic tree. The clade labeled as *Stutzerimonas* in our phylogenetic tree (Figure 1) encompasses all 13 named *Stutzerimonas* species, whose genome sequences were available in the NCBI database at the time of analysis, as well as five non-validly published *Pseudomonas* species. Apart from their clustering in phylogenetic trees, there is no known reliable characteristic which is specific for the members of this genus. Our analyses have identified seven CSIs in different proteins, which in most cases are uniquely shared by all/most species from this clade. Sequence information for one of these CSIs is shown in Figure 4A. In this instance, a one aa insertion in a conserved region of the PAS domain-containing methyl-accepting chemotaxis protein is uniquely shared by all species from the *Stutzerimonas* clade. Detailed sequence information for this CSI and the six other CSIs specific for this clade/genus is provided in Supplementary Figures S32–S38 and some of their characteristics are summarized in Table 3. The identified CSIs provide reliable means for distinguishing *Stutzerimonas* species from all other *Pseudomonadaceae* species. Hence, we are emending the description of this genus to include these diagnostic characteristics.

Five species with non-validly published names [*viz.“P. lopnurensis”* (Mamtimin et al., 2021), *“P. phenolilytica”* (Kujur and Das, 2022)*, “P. oligotrophica”* (Zhang et al., 2022), *“P. saudiphocaensis”* (Azhar et al., 2017) and *“P. songnenensis”* (Zhang et al., 2015)], also group reliably within the *Stutzerimonas* clade and share CSIs specific for this clade. These species should also be recognized as members of this genus with the names *“S. lopnurensis,” “S. phenolilytica,” “S. oligotrophica,” “S. saudiphocaensis”* and *“S. songnenensis”* respectively.

### CSIs specific for the Linyingensis clade

The Linyingensis clade consists of six *Pseudomonas* species *viz.*, *P. aromaticivorans, P. guangdongensis, P. linyingensis, P. oryzagri,* “*P. oryzae*” and *P. sagittaria*, which form a strongly supported clade in our phylogenetic trees (Figure 1; Supplementary Figure S2). This clade is also denoted as g_Pseudomonas_K in the GTDB taxonomy (Parks et al., 2018). A specific evolutionary relationship among these species is supported by 15 CSIs (Table 3), which in most cases are uniquely shared by all species from this clade. In Figure 4B, we present one example of a CSI specific for this clade, where a five aa insertion in UDP-N-acetylmuramoyl-L-alanine--D-glutamate ligase protein is uniquely shared by all members of this clade. Detailed sequence information for this CSI and 14 other CSIs specific for this clade is presented in Supplementary Figures S39–S53. Based on these results, which robustly demarcate this species clade, we are proposing the transfer of these species into *Geopseudomonas* gen. nov.

### CSIs specific for the Resinovorans clade

**Figure 5:**
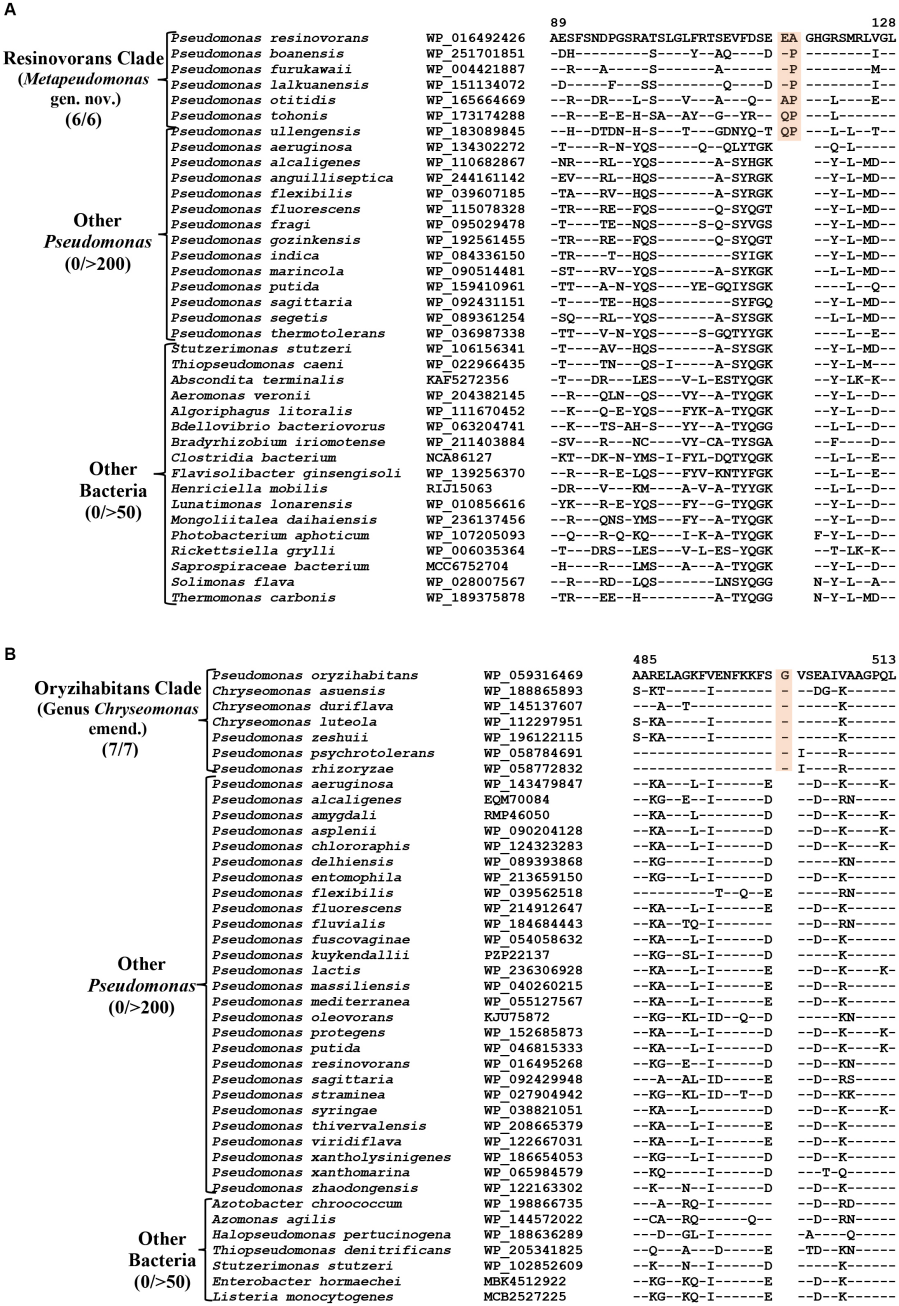
Partial sequence alignment of the protein **(A)** Murein L, D-transpeptidase catalytic domain family protein showing a two aa insertion within a conserved region that is commonly shared by all members of the Resinovorans clade. **(B)** A one aa insertion in the protein Cytochrome d ubiquinol oxidase subunit II which is specific for the species from the Oryzihabitans clade. Detailed sequence information for these CSIs and other CSIs specific for Resinovorans and Oryzihabitans clades are provided in Supplementary Figures S54–S69.

The Resinovorans clade (Figure 1; Supplementary Figure S2), which is denoted as the taxon g_*Pseudomonas*_F in GTDB taxonomy (Parks et al., 2018), consists of six species *viz. P. boanensis*, *P. furukawaii, P. lalkuanensis, P. otitidis, P. resinovorans* and *P. tohonis*. Species from this clade also formed a distinct clade in earlier studies (Girard et al., 2021; Lalucat et al., 2022; Passarelli-Araujo et al., 2022). The members of this clade can be reliably distinguished from all other *Pseudomonadaceae* species by five identified CSIs, which in most cases are exclusively shared by all/most species from this clade. One example of a CSI specific for this clade is presented in Figure 5A, where in the Murein L, D-transpeptidase catalytic domain family protein, a two aa insertion is exclusively present in all species from the Resinovorans clade. Detailed sequence information for this CSI and four other identified CSIs, specific for this clade, is presented in Supplementary Figures S54–S58 and some of their characteristics are listed in Table 4. Based on these results, we are proposing the transfer of species from Resinovorans clade into *Metapseudomonas* gen. nov.

**Table 4:** Summary of CSIs specific for members of the Resinovorans, Oryzihabitans, Thermotolerans, and Flexibilis clades.

Protein name	Accession no	Figure number	Indel size	Indel location	Specificity
Murein L, D-transpeptidase catalytic domain family protein^#^	WP_016492426	Figure 5A; Supplementary Figure S54	2 aa Ins	89–128	Resinovorans clade (*Metapseudomonas* gen. nov.)
Leucine--tRNA ligase^#^	WP_016490742	Supplementary Figure S55	5 aa Ins	260–304	
Alginate biosynthesis protein Alg44	WP_028628607	Supplementary Figure S56	1 aa Del	17–49	
YggL family protein	WP_051246415	Supplementary Figure S57	1 aa Ins	61–93	
Glycine--tRNA ligase subunit beta	WP_016489954	Supplementary Figure S58	3 aa Del	597–641	
Cytochrome d ubiquinol oxidase subunit II	WP_241809250	Figure 5B; Supplementary Figure S59	1 aa Ins	236–279	Oryzihabitans clade (Genus *Chryseomonas*)
Phosphoenolpyruvate carboxykinase	WP_059316469	Supplementary Figure S60	1 aa Ins	485–513	
GTPase HflX	WP_059316391	Supplementary Figure S61	1 aa Ins	317–385	
ATP-binding protein	WP_059313194	Supplementary Figure S62	1 aa Ins	192–230	
16S rRNA (adenine(1518)-N(6)/adenine(1519)-N(6))-dimethyltransferase RsmA	WP_059313310	Supplementary Figure S63	1 aa Del	77–115	
PTS fructose transporter subunit IIBC	HJE68896	Supplementary Figure S64	1 aa Del	36–75	
Glucokinase	WP_007158679	Supplementary Figure S65	1 aa Ins	164–201	
Dienelactone hydrolase family protein	WP_160922865	Supplementary Figure S66	1 aa Ins	40–77	
Bifunctional D-glycero-beta-D-manno-heptose-7-phosphate kinase/D-glycero-beta-D-manno-heptose 1-phosphate adenylyltransferase HldE	WP_059313726	Supplementary Figure S67	1 aa Ins	415–457	
Zinc transporter ZntB	WP_197850824	Supplementary Figure S68	1 aa Ins	209–245	
NADH-dependent 7-cyano-7-deazaguanine	WP_208691271	Supplementary Figure S69	1 aa Ins	180–220	
TerB family tellurite resistance protein	WP_017939833	Figure 6A; Supplementary Figure S70	6 aa Ins	27–75	Thermotolerans clade (*Zestomonas* gen. nov.)
TIGR02099 family protein	WP_119894903	Supplementary Figure S71	1 aa Del	175–206	
HAMP domain-containing histidine kinase	WP_187671317	Supplementary Figure S72	1 aa Ins	359–390	
23S rRNA (adenine(2030)-N(6))-methyltransferase RlmJ	WP_119895222	Supplementary Figure S73	1 aa Del	47–87	
Esterase-like activity of phytase family protein	WP_119895183	Supplementary Figure S74	1 aa Ins	261–299	
GTP diphosphokinase	WP_039562945	Figure 6B; Supplementary Figure S75	1 aa ins	464–500	Flexibilis clade (Genus *Serpens* emend.)
Zinc ABC transporter permease subunit ZnuB	WP_039607122	Supplementary Figure S76	1 aa Del	85–120	
LutB/LldF family L-lactate oxidation iron–sulfur protein	WP_039560866	Supplementary Figure S77	1 aa Del	433–469	

^#^Isolated exception present in some CSIs (#; see Supplementary Figures for details).

### CSIs specific for the Oryzihabitans clade (genus *Chryseomonas*)

Oryzihabitans clade (denoted as the taxon g_*Pseudomonas*_B in GTDB taxonomy) consists of seven named *Pseudomonas* species *viz. P. asuensis, P. duriflava, P. luteola, P. oryzihabitans, P. psychrotolerans, P. rhizoryzae* and *P. zeshuii*, which form a strongly supported clade in our phylogenetic trees (Figure 1; Supplementary Figure S2). These species also formed a distinct clade in earlier phylogenetic studies (Hesse et al., 2018; Girard et al., 2021; Saati-Santamaría et al., 2021; Passarelli-Araujo et al., 2022). The best-studied species from this clade is *P. luteola*, which was originally a member of the genus *Chryseomonas* (Holmes et al., 1986). However, in 1997, based on 16S rRNA gene sequence similarity, this species was transferred into the genus *Pseudomonas* (Anzai et al., 1997). More recently, based on genomic studies, this species along with two other *Pseudomonas* species (*P. asuensis* and *P. duriflava*) were transferred into the genus *Chryseomonas*. It should be noted that *C. luteola* is a synonym of *C. polytricha* (Holmes et al., 1986), which is the type species of genus *Chryseomonas* (Parte et al., 2020). The genetic distinctness of the clade formed by these seven species is strongly supported by 11 novel identified CSIs which are uniquely shared by these species. One example of a CSIs specific for this clade is shown in Figure 5B. In this case, a one aa insertion in the protein cytochrome d ubiquinol oxidase subunit II is exclusively shared by all members of this clade. Detailed sequence information for this CSI and 10 other CSIs specific for this clade are presented in Supplementary Figures S59–S69 and some of their characteristics are listed in Table 4. In addition to the three species which are presently assigned to the genus *Chryseomonas*, four additional *Pseudomonas* species *viz. P. oryzihabitans*, *P. psychrotolerans, P. rhizoryzae* and *P. zeshuii* reliably group within this clade and share different CSIs specific for this genus. Hence, we are proposing new name combinations of these species to transfer them into the genus *Chryseomonas*.

### CSIs specific for the Thermotolerans clade

**Figure 6:**
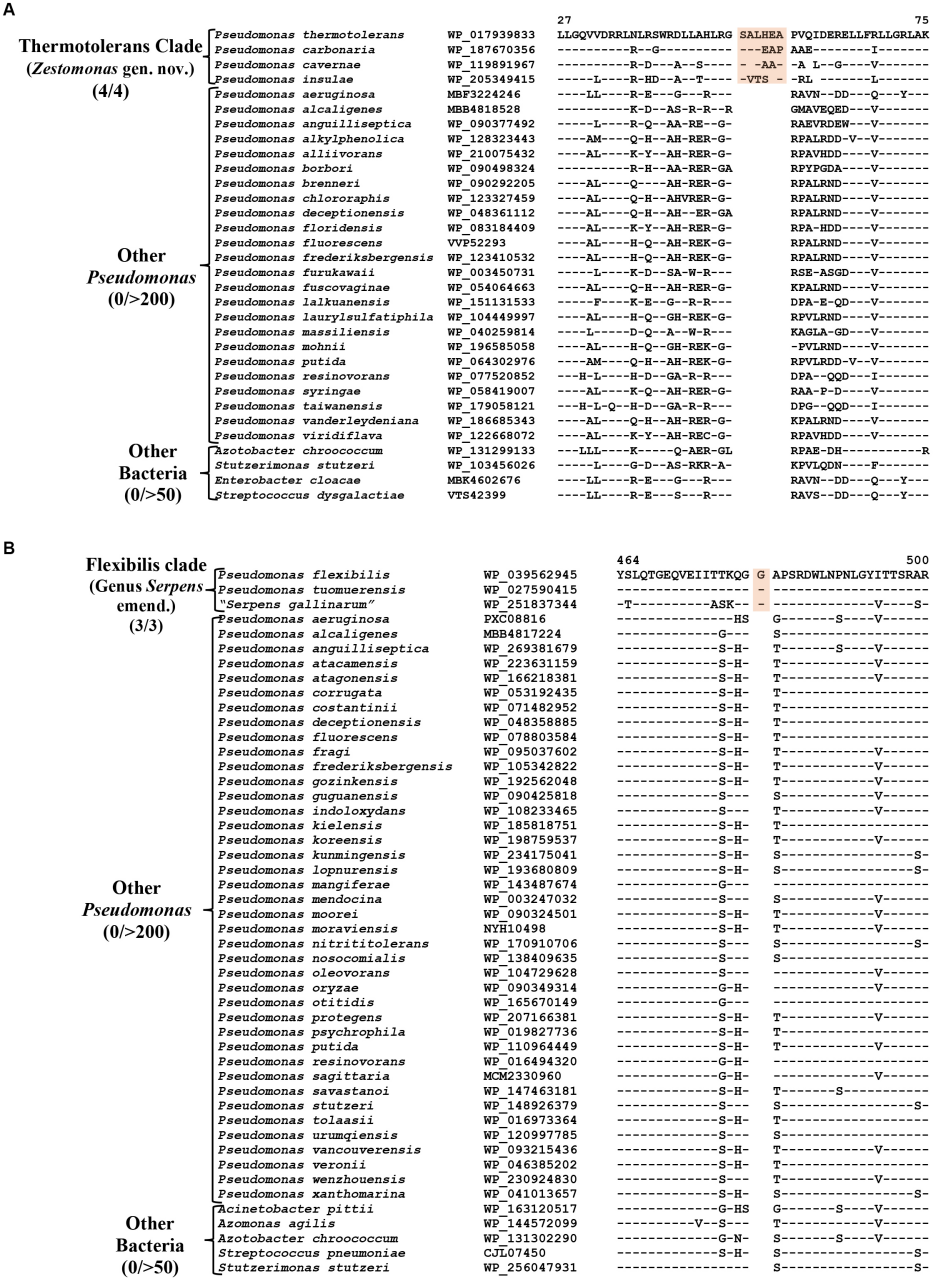
Partial sequence alignment of the protein **(A)** TerB family tellurite resistance protein showing a six aa insertion within a conserved region (highlighted) that is uniquely shared by members of the Thermotolerans clade. **(B)** A one aa insertion in a conserved region of the protein GTP diphosphokinase which is specific for the species from Flexibilis clade. Detailed sequence information for these CSIs and other CSIs specific for the Thermotolerans and Flexibilis clades are provided in Supplementary Figures S70–S77.

The Thermotolerans clade includes the species *P. carbonaria, P. cavernae*, *P. insulae* and *P. thermotolerans,* which form a distinct clade in our phylogenomic trees (Figure 1; Supplementary Figure S2). Species from this clade also formed a distinct cluster in earlier studies (Girard et al., 2021; Lalucat et al., 2022). A specific evolutionary relationship among these species is strongly supported by five CSIs, which are exclusively shared by all members of this clade. One example of a CSI specific for this clade is shown in Figure 6A, where a six aa insertion in the TerB family tellurite resistance protein is exclusively found in all four species from this clade. Detailed sequence information for the five CSIs specific for this clade are presented in Supplementary Figures S70–S74 and some of their characteristics are listed in Table 4. Based on these results, we are proposing the transfer of species from this clade into *Zestomonas* gen. nov.

### CSIs specific for the Flexibilis clade (genus *Serpens*)

*Pseudomonas flexibilis*, formerly known as *Serpens flexibilis* (Hespell, 1977) was recently transferred into the genus *Pseudomonas* based on 16S rRNA similarity with *P. pseudoalcaligenes* (Shin et al., 2015). In our phylogenomic tree (Figure 1), this species branches separately from other *Pseudomonas* species and forms a distinct clade together with a newly described non-validly published species “*Serpens gallinarum*” (Gilroy et al., 2021) and another species *P. tuomuerensis*, which according to Shin et al. (2015) is a heterotypic synonym of *P. flexibilis*. This clade is identified as the taxon g_*Pseudomonas*_H in the GTDB taxonomy (Parks et al., 2018). A close and specific relationship of *P. flexibilis* (*P. tuomuerensis*) to “*S. gallinarum*” is independently supported by three CSIs identified in this study, which are exclusively shared by these species. One example of a CSI specific for this clade is shown in Figure 6B, where a one aa insertion in the protein GTP diphosphokinase is specifically shared by these three species. Detailed sequence information for this CSI and the two other CSIs specific for this clade is presented in Supplementary Figures S75–S77 and some of their characteristics are summarized in Table 4. Based on these results we are presenting an emended description of the genus *Serpens* with *S. flexibilis* as its type species.

### CSIs specific for the Fluvialis clade

**Figure 7:**
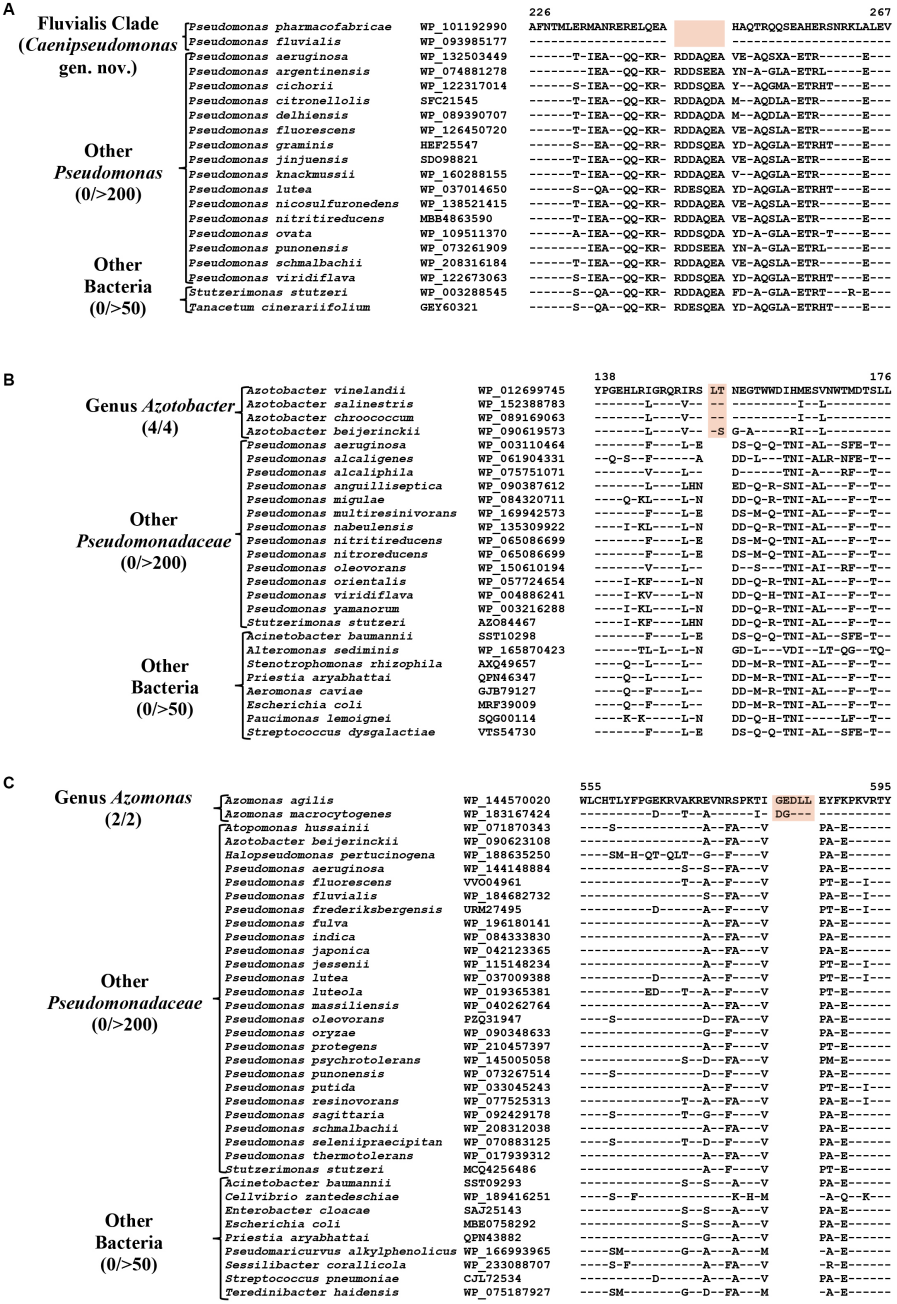
Partial sequence alignment of **(A)** ATP binding protein showing seven aa deletion within a conserved region (highlighted) that is uniquely shared by species from the Fluvialis clade. **(B)** A two aa insertion in a conserved region of the Alginate export family protein showing that is exclusively shared by species from the genus *Azotobacter*. **(C)** A five aa insertion in the protein Succinate dehydrogenase flavoprotein subunit which is specific for the species from genus *Azomonas*. Detailed sequence information for these CSIs and other CSIs specific for the Fluvialis clade and the *Azotobacter* and *Azomonas* genera are provided in Supplementary Figures S78–S100.

The *Fluvialis* clade consists of the species *P. fluvialis* and *P. pharmacofabricae*, which formed a strongly supported clade in different phylogenetic trees (Figure 1; Supplementary Figure S2). Our analyses have identified eight CSIs in different proteins that are uniquely shared by these two species. Figure 7A depicts an example of a CSI, consisting of a seven aa deletion within a conserved region of an ATP binding protein, which is exclusively shared by these two species. Detailed sequence information for this and the six other CSIs specific for the Fluvalis clade is presented in Supplementary Figures S78–S85 and a summary of some of their sequence characteristics is presented in Table 5. Based on the results presented here, we are proposing the transfer of species from this clade into *Caenipseudomonas* gen. nov.

**Table 5:** Summary of CSIs specific for members of the Fluvialis clade, and the genera *Azotobacter* and *Azomonas.*

Protein name	Accession no	Figure number	Indel size	Indel location	Specificity
ATP-binding protein	WP_101192990	Figure 7A; Supplementary Figure S78	7 aa Del	226–267	Fluvialis clade (*Caenipseudomonas* gen. nov.)
Hypothetical protein	WP_101193738	Supplementary Figure S79	5 aa Del, 1 aa Del	146–197	
DUF2868 domain-containing protein	WP_101193981	Supplementary Figure S80	5 aa Del	415–452	
Hypothetical protein	WP_093984635	Supplementary Figure S81	2 aa Del	101–143	
Putative chorismate pyruvate-lyase	GGH90722	Supplementary Figure S82	2 aa Del	72–119	
Bifunctional aminoglycoside phosphotransferase/ATP-binding protein	WP_093984289	Supplementary Figure S83	2 aa Ins	77–117	
RDD family protein	WP_101192354	Supplementary Figure S84	1 aa Ins	160–200	
Translocation/assembly module TamB	WP_093986880	Supplementary Figure S85	2 aa Ins	493–539	
Alginate export family protein	WP_012699745	Figure 7B; Supplementary Figure S86	2 aa Ins	138–176	Genus *Azotobacter*
DNA polymerase III subunit alpha	WP_012702399	Supplementary Figure S87	1 aa Ins	88–132	
Pyrroloquinoline quinone biosynthesis protein	WP_152387189	Supplementary Figure S88	1 aa Del	238–276	
Protein-export chaperone SecB	WP_012699152	Supplementary Figure S89	1 aa Ins	33–71	
Protein Ion transporter	WP_012701585	Supplementary Figure S90	1 aa Del	25–70	
Cysteine synthase A	WP_012701826	Supplementary Figure S91	1 aa Ins	268–309	
DUF2066 domain-containing protein	WP_012702209	Supplementary Figure S92	2 aa Del	156–195	
GGDEF domain-containing phosphodiesterase	WP_012702302	Supplementary Figure S93	1 aa Del	389–431	
Flagellar hook-associated protein FlgL	WP_012700992	Supplementary Figure S94	1 aa Ins	131–167	
LLM class flavin-dependent oxidoreductase	WP_012699059	Supplementary Figure S95	3 aa Del	87–130	
Succinate dehydrogenase flavoprotein subunit	WP_144570020	Figure 7C; Supplementary Figure S96	5 aa Ins	555–595	Genus *Azomonas*
Mechanosensitive channel MscK	WP_183165886	Supplementary Figure S97	4 aa Del	790–819	
SPOR domain-containing protein	WP_144571310	Supplementary Figure S98	2 aa del	73–110	
Bifunctional [glutamate--ammonia ligase]-adenylyl-L-tyrosine phosphorylase adenylyltransferase	WP_183165719	Supplementary Figure S99	1 aa Del	153–185	
Alkyl hydroperoxide reductase subunit F	WP_144571471	Supplementary Figure S100	1 aa Del	366–398	

### Identification of CSIs specific for the *Azotobacter* and *Azomonas* genera

The genus *Azotobacter* was described by Beijerinck (1901) and its members are known to branch in between *Pseudomonas* species (Young and Park, 2007; Özen and Ussery, 2012; Lalucat et al., 2022). Four *Azotobacter* species whose genome sequences were analyzed in this study (*viz. A. beijerinckii, A. chroococcum, A. salinestris,* and *A. vinelandii*), formed a distinct clade branching in the proximity of Stutzeri and Linyingensis clades (Figure 1; Supplementary Figure S2). Similar branching of *Azotobacter* species has been reported in earlier work (Jun et al., 2016; Hesse et al., 2018; Lalucat et al., 2022). Our analyses have identified 10 CSIs which are exclusively found in all four *Azotobacter* species providing reliable means for the demarcation of this clade. Partial sequence information for one of the CSIs specific for this genus, found in the alginate export family protein, is shown in Figure 7B. Detailed sequence information for this CSI and nine other CSIs specific for this genus is provided in Supplementary Figures S86–S95, and some of their sequence characteristics are listed in Table 5.

*Azomonas* is another genus whose members branch in between *Pseudomonas* species (Figure 1; Supplementary Figure S2; Young and Park, 2007; Kennedy and Rudnick, 2015; Rudra and Gupta, 2021; Lalucat et al., 2022). The two *Azomonas* species included in our analyses (*viz.*, *A. agilis* and *A. macrocytogenes*) formed a distinct cluster in our phylogenomic trees (Figure 1; Supplementary Figure S2). The distinctness of this clade is also supported by five CSIs identified in this work, which are exclusively shared by these two species. Sequence information for one of these CSIs, containing a five aa insertion within the protein succinate dehydrogenase flavoprotein, is shown in Figure 7C. Detailed sequence information for this CSI and the other four CSIs specific for this genus are provided in the Supplementary Figures S96–S100, and a summary of some of their sequence characteristics is listed in Table 5.

## Discussion

The genus *Pseudomonas* is one of the earliest known and largest prokaryotic genera encompassing a large assemblage of organisms exhibiting enormous genetic and metabolic diversity (Palleroni, 2005;Peix et al., 2009; Silby et al., 2011; Palleroni, 2015). The nomenclature type of this genus, *P. aeruginosa*, is an important human pathogen capable of causing a wide array of life-threatening acute and chronic diseases (Lund-Palau et al., 2016; Rossi et al., 2021). However, this genus also includes some animals and plant pathogenic species, as well as other economically and ecologically significant species (Desnoues et al., 2003; Silby et al., 2011; Xin et al., 2018). According to the LPSN (Parte et al., 2020), the genus *Pseudomonas* presently contains ≈310 species with validly published names. However, this number is increasing at a rapid pace (Girard et al., 2021), and in 2022 alone, more than 50 novel *Pseudomonas* species were listed in the LPSN server (Parte et al., 2020). As indicated in the introduction, and reviewed by others (Palleroni, 2010; Peix et al., 2018; Lalucat et al., 2022), evolutionary studies on the genus *Pseudomonas* have consistently shown that these species form multiple distinct clusters/clades, which are not specifically related to each other (Gomila et al., 2015; Hesse et al., 2018; Girard et al., 2021; Rudra and Gupta, 2021; Saati-Santamaría et al., 2021). Furthermore, it is generally recognized that of these species’ clades, circumscription of the genus *Pseudomonas* should be limited to the “Aeruginosa clade” harboring its type species, whereas species from the other observed clades should be reclassified into either novel or existing genera. In recent years, although several *Pseudomonas* species from deep branching clusters have been reclassified into novel genera (*viz. Atopomonas, Chryseomonas, Halopseudomonas* and *Stutzerimonas*) (Rudra and Gupta, 2021; Saati-Santamaría et al., 2021; Lalucat et al., 2022), the task of reliably reclassifying majority (>90%) of the *Pseudomonas* species into well-demarcated genera has proven challenging.

With the aim of reliably demarcating some of the observed *Pseudomonas* species clades, we have conducted here comprehensive phylogenomic and comparative analyses on the genome sequences of *Pseudomonadaceae* species. In our phylogenomic trees, *Pseudomonas* species formed multiple distinct clades (Figure 1; Supplementary Figure S2), which are similar to those reported in earlier studies (Gomila et al., 2015; Peix et al., 2018; Girard et al., 2021; Lalucat et al., 2022) excepting some differences resulting from the inclusion of several new species in our analysis. However, while similar species clusters are observed in different studies, based on their branching in phylogenetic trees (see Figure 1; Supplementary Figure S2), which is dynamic in nature and influenced by multiple variables including addition of new species (Gupta, 1998; Baldauf, 2003; Felsenstein, 2004), it is difficult to reliably demarcate the boundaries of different clades. Thus, a major focus of this study was to identify robust molecular markers, which independent of phylogenetic analyses, can confirm the existence of observed species clades and can provide reliable means for their demarcation.

Although genome sequence based indices such as average nucleotide identity (ANIb) and genome to genome DNA hybridization (GGDC) are now widely used for the delimitation of species level taxa (Goris et al., 2007; Kim et al., 2014; Yarza et al., 2014), such methods including AAI (Konstantinidis and Tiedje, 2007) or POCP (Qin et al., 2014) have shown limited usefulness for the delineation of genus level taxa (Parks et al., 2018; Gupta, 2019; Gupta and Kanter-Eivin, 2023). In the present work, while based on POCP and AAI values, some *Pseudomonas* species clades appear to be distinct (Table 1 and Supplementary Tables S2 and S3), for most of the observed clades these values generally show some overlap between the ingroup and outgroup species. Thus, based on these indices, it is difficult to reliably demarcate the boundaries of most of the clades. However, genome sequences are also enabling identification of highly specific molecular markers such as CSIs which are uniquely shared by different groups of organisms and provide dependable means for taxonomic and diagnostic studies (Gupta, 2014; Adeolu et al., 2016; Gupta, 2016; Gupta et al., 2020). As the CSIs in genes/proteins sequences result from rare genetic changes, their presence or absence in different species is generally not affected by most factors which can confound inferences from phylogenetic analyses (Baldauf and Palmer, 1993; Gupta, 1998; Rokas and Holland, 2000; Gupta, 2014, 2016). Furthermore, as the CSIs in different genes/proteins result from unrelated genetic changes, each of them provides independent evidence of a close and specific evolutionary relationship among a given group of species. In the present work, detailed analyses conducted on protein sequences from *Pseudomonadaceae* species, have identified 98 CSIs, which are specific for the species from 13 different *Pseudomonadaceae* species clades including the genera *Azomonas* and *Azotobacter*. Table 6 shows a summary of the CSIs that were identified for different *Pseudomonadaceae* clades along with the species that currently comprise these clades.

**Table 6:** Summary of different *Pseudomonadaceae* species clades reliably demarcated based on phylogenomic analyses and identified CSIs specific for these clades.

Clade name (Genus name)	Number of CSIs	Species composition of the clades
“Aeruginosoa clade” (*Pseudomonas sensu stricto*)	6	*P. aeruginosa, P. citronellolis, P. delhiensis, P. humi, P. jinjuensis, P. knackmussii, P. multiresinivorans, P. nicosulfuronedens, P. nitritireducens, P. nitroreducens, P. paraeruginosa, P. panipatensis, “P. pseudonitroreducens,” P. schmalbachii.*
Alcaligenes clade (*Aquipseudomonas* gen. nov.)	6	*P. alcaligenes, P. campi, P. guryensis, P. ullengensis*
Genus *Azomonas*	5	*A. agilis A. macrocytogenes*
Genus *Azotobacter*	10	*A. chroococcum, A. beijerinckii, A. salinestris, A. vinelandii.*
Flexibilis clade (Genus *Serpens* emend.)	3	*P. flexibilis, “Serpens gallinarum,” P. tuomuerensis*.
Fluvialis clade (*Caenipseudomonas* gen. nov.)	8	*P. fluvialis, P. pharmacofabricae*
Linyingensis clade (*Geopseudomonas* gen. nov.)	15	*P. guangdongensis, P. aromaticivorans, P. linyingensis, “P. oryzae,” P. oryzagri, P. sagittaria*
Oleovorans clade (*Ectopseudomonas* gen. nov.)	5	*P. alcaliphila, P. chengduensis, P. composti, P. guguanensis, P. hydrolytica, “P. indoloxydans,” P. khazarica, P. mendocina, P. oleovorans, P. pseudoalcaligenes, “P. sediminis,” “P. sihuiensis,” P. toyotomiensis, “P. wenzhouensis,” P. yangonensis*
Oryzihabitans clade (Genus *Chryseomonas* emend.)	11	*C. asuensis, C. duriflava, C. luteola, P. oryzihabitans, P. psychrotolerans, P. rhizoryzae, P. zeshuii*
Resinovorans clade (*Metapseudomonas* gen. nov.)	5	*P. boanensis, P. furukawaii, P. lalkuanensis, P. otitidis, P. resinovorans, P. tohonis*
Straminea clade (*Phytopseudomonas* gen. nov.)	12	*P. argentinensis, P. daroniae, P. dryadis, P. flavescens, P. punonensis, P. seleniipraecipitans, P. straminea.*
*Stutzeri* clade (Genus *Stutzerimonas*)	7	*S. azotifigens, S. balearica, S. chloritidismutans, S. decontaminans, S. degradans, S. frequens, S. kirkiae, S. kunmingensis, S. nitrititolerans, S. nosocomialis, S. perfectomarina, S. stutzeri, S. tarimensis, S. xanthomarina, S. zhaodongensis, S. urumqiensis, “P. lopnurensis,” “P. phenolilytica,” “P. oligotrophica,” “P. saudiphocaensis,” “P. songnenensis.”*
Thermotolerans clade (*Zestomonas* gen. nov.)	5	*P. carbonaria, P. cavernae, P. insulae, P. thermotolerans*

The results presented in Table 6 show that most of the *Pseudomonas* species clades, which are observed in our phylogenomic trees (Figure 1; Supplementary Figure S2), can now be robustly demarcated based on multiple identified CSIs, which are exclusively shared by the species from these clades. The genetic relatedness of the species from several of these clades is also supported by the results from AAI and POCP indices (Table 1). However, one clade for which CSIs were not identified is the Anguilliseptica clade. Species from this clade do not also form a well-resolved and strongly supported lineage in our phylogenetic trees (Figure 1; Supplementary Figure S2), and in earlier studies (Hesse et al., 2018; Busquets et al., 2021; Lalucat et al., 2022). In some phylogenetic trees [Supplementary Figure S2, unpublished results, and (Hesse et al., 2018)], one or more species from this clade (*viz. P. cuatrocienegasensis*) branch outside this clade. The results from AAI and POCP analyses (Table 1) also do not support the distinctness of this clade. All these observations indicate that the Anguilliseptica clade is not a trustworthy lineage and the cladistic relationships of species from this clade need to be further investigated. Of the CSIs identified by our analysis, six are uniquely shared by different species from the “Aeruginosa clade,” providing reliable molecular means for the demarcation/circumscription of this clade representing the genus *Pseudomonas sensu stricto*. Our analyses have also identified multiple CSIs reliably demarcating the species from Alcaligenes, Fluvialis, Linyingensis, Oleovorans, Resinovorans, Straminea, and Thermotolerans clades. Based on the strong and consistent evidence provided by phylogenomic analyses and identified molecular signatures supporting the distinctness of these clades, we are proposing that the species from the above noted clades should be reclassified into the following novel genera *Aquipseudomonas* gen. nov., *Caenipseudomonas* gen. nov., *Geopseudomonas* gen. nov., *Ectopseudomonas* gen. nov., *Metapseudomonas* gen. nov., *Phytopseudomonas* gen. nov., and *Zestomonas* gen. nov., respectively (Table 6). Our work has also identified 11 CSIs which are shared by all species from the Oryzihabitans clade providing robust means for the demarcation of species from this clade. Previously, only three species, which form a subclade of the Oryzihabitans clade, were reclassified into the genus *Chryseomonas* (Saati-Santamaría et al., 2021). Based on the results presented, we are proposing that the other species from this clade should also be transferred into the emended genus *Chryseomonas*. Species from the Flexibilis clade containing *P. flexibilis* are also transferred into the emended genus *Serpens*. Seven identified CSIs are commonly shared by all 13 species from the *Stutzerimonas* clade (Lalucat et al., 2022) providing robust molecular means for the demarcation of this genus. Lastly, multiple CSIs identified by our analyses are specific for the genera *Azomonas* and *Azotobacter* providing trustworthy means for the demarcation of these genera in molecular terms. As the identified CSIs provide important diagnostic characteristics of the above noted genera, we are also providing emended descriptions of these genera to include this information.

Although the present work represents a significant step toward clarifying the evolutionary relationships and classification scheme for *Pseudomonas* species, a vast majority of *Pseudomonas* species representing more than two thirds of the known species (see Supplementary Figure S1), are part of the Fluorescens superclade. As seen from Supplementary Figure S1, this large lineage is comprised of multiple clades and subclades (Palleroni, 2015; Hesse et al., 2018; Peix et al., 2018; Lalucat et al., 2020; Girard et al., 2021). To develop a reliable classification scheme for all *Pseudomonas* species, it will be necessary to reliably distinguish and demarcate different species clades within the Fluorescens superclade and reclassify them appropriately. In view of this consideration, despite our reliable demarcation of the genus *Pseudomonas sensu stricto*, an emended description of this genus is not proposed, until most other *Pseudomonas* species are reliably classified.

All newly proposed genera and other studied genera/clades in this work have been circumscribed based on their harboring multiple uniquely shared CSIs. One notable characteristic of the CSIs, which is of much importance for classification purposes, is that these markers exhibit high degree of predictive ability to be found in other (uncharacterized or unidentified) members of a given group/taxon (Bhandari et al., 2013; Gupta, 2014, 2016; Dobritsa and Samadpour, 2019; Patel and Gupta, 2020; Montecillo and Bae, 2022). Thus, the CSIs specific for the genus *Halopseudomonas* identified in our earlier work (Rudra and Gupta, 2021) are also present in all newly described species from this genus (Supplementary Figure S2). Similarly, the CSIs specific for the genus *Atopomonas* were also present in a newly described species from this genus (Li et al., 2023). Due to the demonstrated predictive abilities of the CSIs to be found in other members of specific taxa, we have recently developed a web-based tool/server,^3^ which can predict taxonomic affiliation based on the presence of known taxon-specific CSIs in a genome sequence (Gupta and Kanter-Eivin, 2023). Therefore, upon the addition of information for these newly identified CSIs to the AppIndels server, it should greatly facilitate the classification of both cultured and uncultured isolates related to the described taxa (Gupta and Patel, 2019). The CSIs specific for different taxa also provide useful means for the development of sensitive and specific diagnostic tests using *in silico* and experimental methods (Ahmod et al., 2011; Wong et al., 2014). Lastly, the earlier work on CSIs show that these molecular characteristics are functionally important for the group of organisms for which they are specific (Singh and Gupta, 2009; Khadka et al., 2020). Hence, genetic, and biochemical studies on the identified CSIs could lead to the discovery of novel biochemical and/or other characteristics of different groups of organisms.

The descriptions of different novel genera proposed and other emended genera are given below. The new name combinations for different species resulting from the proposed taxonomic changes are provided in Tables 7, 8. The names for the newly proposed genera are generally based on some characteristics of the proposed group of species.

**Table 7:** Descriptions of the new name combinations for different proposed genera.

New name combination and etymology	Basonym	Description	Type strain
*Aquipseudomonas* gen. nov.			
*Aquipseudomonas alcaligenes* comb. nov. (type species) (al.*ca.*li’ge.nes. N.L. n. *alcali*, alkali; from Arabic article *al*, the; from Arabic masc. n. *qaly*, ashes of saltwort; Gr. suff. *-genes*, producing; from Gr. ind. v. *gennaô*, to produce; N.L. part. adj. *alcaligenes*, alkali-producing)	*Pseudomonas alcaligenes* Monias, 1928 (Approved Lists 1980).	The description of this species is the same as provided by Monias (1928).	ATCC 14909; CCUG 1425; CCUG 1425 A; CFBP 2437; CIP 101034; DSM 50342; IFO 14159; JCM 5967; LMG 1224; NBRC 14159; NCCB 76044; NCTC 10367; VKM B-2171.
*Aquipseudomonas campi* comb. nov. (cam’pi. L. gen. n. *campi*, of a field, of grassland)	*Pseudomonas campi* Timsy et al., 2021	The description of this species is the same as provided by Timsy et al. (2021).	31,521; DSM 110222; LMG 31521; S1-A32-2
*Aquipseudomonas guryensis* comb. nov. (gu.ryen’sis. N.L. fem. adj. *guryensis*, pertaining to Gurye, a geographic location where the type strain was isolated)	*Pseudomonas guryensis* Kim et al., 2021.	The description of this species is the same as provided by Kim et al. (2021).	JCM 34509; KCTC 82228; SR9.
*Aquipseudomonas ullengensis* comb. nov. (ull.eng.en’sis. N.L. fem. adj. *ullengensis*, pertaining to Ulleng Island, a geographic location where the type strain was isolated)	*Pseudomonas ullengensis* Kim et al., 2021.	The description of this species is the same as provided by Kim et al. (2021).	JCM 34510; KCTC 82229; UL070.
*Caenipseudomonas* gen. nov.			
*Caenipseudomonas fluvialis* comb. nov. (type species) (flu.vi.a’lis. L. fem. adj. *fluvialis*, belonging to a river, the source of the isolate)	*Pseudomonas fluvialis* Sudan et al., 2018.	The description of this species is the same as provided by Sudan et al. (2018).	ASS-1; CCM 8778; KCTC 52437.
*Caenipseudomonas pharmacofabricae* comb. nov. (phar.ma.co.fa’bri.cae. N.L gen. n. pharmacofabricae from a pharmaceutical factory)	*Pseudomonas pharmafabricae* Yu et al., 2018.	The description of this species is the same as provided by Yu et al. (2018).	CGMCC 1.15498; JCM 31306; ZYSR67-Z.
*Ectopseudomonas* gen. nov.			
*Ectopseudomonas oleovorans* comb. nov. (type species) (o.le.o.vo.rans. L. neut. n. *oleum*, oil; L. pres. part. *vorans*, eating, devouring; N.L. part. adj. *oleovorans*, oil devouring)	*Pseudomonas oleovorans* Lee and Chandler, 1941 (Approved Lists 1980).	The description of this species is the same as provided by Lee and Chandler (1941).	ATCC 8062; CCUG 2087; CFBP 5589; CIP 59.11; DSM 1045; IFO 13583; JCM 11598; LMG 2229; NBRC 13583; NCIB 6576; NCIMB 6576; NCTC 10692; NRRL B-778; VKM B-1522.
*Ectopseudomonas alcaliphila* comb. nov. (al.*ca.*li.phi’la. N.L. n. *alcali*, alcali (from Arabic article al, the; Arabic n. qaliy, ashes of saltwort); N.L. fem. adj. suff. *-phila*, friend, loving; from Gr. fem. adj. *philê*, loving; N.L. fem. adj. *alcaliphila*, liking alkaline environments)	*Pseudomonas alcaliphila* Yumoto et al., 2001.	The description of this species is the same as provided by Yumoto et al. (2001).	AL15-21; DSM 17744; IAM 14884; JCM 10630; NBRC 102411.
*Ectopseudomonas chengduensis* comb. nov. (cheng.du.en’sis. N.L. fem. adj. *chengduensis*, pertaining to Chengdu, where the type strain was isolated)	*Pseudomonas chengduensis* Tao et al., 2014.	The description of this species is the same as provided by Tao et al. (2014).	CGMCC 2318; DSM 26382; MBR.
*Ectopseudomonas composti* comb. nov. (com.pos’ti. N.L. gen. n. *composti*, of compost, from which strains were first isolated)	*Pseudomonas composti* Gibello et al., 2011.	The description of this species is the same as provided by Gibello et al. (2011).	C2; CCUG 59231; CECT 7516; DSM 25648.
*Ectopseudomonas guguanensis* comb. nov. (gu.guan.en’sis. N.L. fem. adj. *guguanensis*, of or pertaining to Guguan, the location of a favorite hot spring attraction in Taiwan)	*Pseudomonas guguanensis* Liu et al., 2013.	The description of this species is the same as provided by Liu et al. (2013).	BCRC 80438; CC-G9A; JCM 18416.
*Ectopseudomonas hydrolytica* comb. nov. (hy.dro.ly’ti.*ca.* Gr. neut. n. *hydôr*, water; Gr. masc. adj. *lytikos*, dissolving, splitting; N.L. fem. adj. *hydrolytica*, splitting with water, referring to the hydrolytic enzymatic activity of the bacterium).	*Pseudomonas hydrolytica* Zhou et al., 2020.	The description of this species is the same as provided by Zhou et al. (2020).	CCTCC AB 2018053; DSM 106702; DSWY01.
*Ectopseudomonas khazarica* comb. nov. (kha.za’ri.*ca.* N.L. fem. adj. *khazarica*, pertaining to Khazar, a lake in the north of Iran as the largest lake in the world, from where the organism was isolated)	*Pseudomonas khazarica* Tarhriz et al., 2020.	The description of this species is the same as provided by Tarhriz et al. (2020).	KCTC 52410; LMG 29674; Tbz2.
*Ectopseudomonas mendocina* comb. nov. (men.do.ci.na. N.L. fem. adj. *mendocina*, pertaining to Mendoza (Argentina))	*Pseudomonas mendocina* Palleroni et al., 1970 (Approved Lists 1980).	The description of this species is the same as provided by Palleroni et al. (1970).	ATCC 25411; CCUG 1781; CFBP 2434; CIP 75.21; DSM 50017; IFO 14162; JCM 5966; LMG 1223; NBRC 14162; NCCB 76043; NCTC 10897; VKM B-972.
*Ectopseudomonas pseudoalcaligenes* comb. nov.(pseu.do.al.*ca.*li’ge.nes. Gr. masc. adj. *pseudes*, false; N.L. n. *alcali*, alkali; from Arabic article *al*, the; from Arabic masc. n. *qaly*, ashes of saltwort; Gr. suff. *-genes*, producing; from Gr. ind. v. *gennaô*, to produce; N.L. pres. part. *alcaligenes*, alkali-producing; N.L. part. adj. *pseudoalcaligenes*, false alkali producing)	*Pseudomonas pseudoalcaligenes* Stanier et al., 1966 (Approved Lists 1980).	The description of this species is the same as provided by Stanier et al. (1966).	ATCC 17440; CCUG 51525; CFBP 2435; CIP 66.14; DSM 50188; IFO 14167; JCM 5968; LMG 1225; NBRC 14167; NCCB 76045; NCTC 10860.
*Ectopseudomonas toyotomiensis* comb. nov. (to.yo.to.mi.en’sis. N.L. fem. adj. *toyotomiensis*, pertaining to Toyotomi, where the type strain was isolated)	*Pseudomonas toyotomiensis* Hirota et al., 2011.	The description of this species is the same as provided by Hirota et al. (2011).	DSM 26169; HT-3; JCM 15604; NCIMB 14511.
*Ectopseudomonas yangonensis* comb. nov. (yan.gon.en’sis. N.L. fem. adj. *yangonensis*, from or originating from Yangon, Myanmar, where the type strain was isolated)	*Pseudomonas yangonensis* Tohya et al., 2020.	The description of this species is the same as provided by Tohya et al. (2020).	JCM 33396; LMG 31602; MY50.
*Geopseudomonas* gen. nov.			
*Geopseudomonas sagittaria* comb. nov. (type species) (sa.git.ta’ria. L. fem. adj. *sagittaria*, pertaining to the constellation Sagittarius as the novel species was isolated during the month of November, the birthday of first author (Shih-Yao Lin) of the paper describing this species; from L. masc. adj. *sagittarius*, the constellation Sagittarius)	*Pseudomonas sagittaria* Lin et al., 2013.	The description of this species is the same as provided by Lin et al. (2013).	BCRC 80399; CC-OPY-1; DSM 27945; JCM 18195.
*Geopseudomonas aromaticivorans* comb. nov. (a.ro.ma.ti.ci.vo’rans. L. masc. adj. *aromaticus*, fragrant; L. pres. part. *vorans*, devouring; N.L. part. adj. *aromaticivorans*, devouring aromatic compounds)	*Pseudomonas aromaticivorans* Banerjee et al., 2022.	The description of this species is the same as provided by Banerjee et al. (2022).	LMG 32466; MAP12; NCAIM B.02668.
*Geopseudomonas linyingensis* comb. nov. (lin.ying.en’sis. N.L. fem. adj. *linyingensis*, pertaining to Linying, in Henan province, China, where the type strain was isolated).	*Pseudomonas linyingensis* He et al., 2012.	The description of this species is the same as provided by He et al. (2012).	CGMCC 1.10701; LMG 25967; LYBRD3-7
*Geopseudomonas guangdongensis* comb. nov. (guang.dong.en’sis. N.L. fem. adj. *guangdongensis*, of or pertaining to Guangdong, a province in south-east China, from where the type strain was isolated).	*Pseudomonas guangdongensis* Yang et al., 2013.	The description of this species is the same as provided by Yang et al. (2013).	CCTCC AB 2012022; DSM 100318; KACC 16606; SgZ-6.
*Geopseudomonas oryzagri* comb. nov. (o.ryz.a’gri. L. fem. n. *oryza*, rice; L. n. *ager*, a field; N.L. gen. n. *oryzagri*, of a rice field)	*Pseudomonas oryzagri* Huq et al., 2022.	The description of this species is the same as provided by Huq et al. (2022).	CGMCC 1.18518; KACC 22005; MAHUQ-58

**Table 8:** Descriptions of the new name combinations for different proposed and emended genera.

New name combination and etymology	Basonym	Description	Type strain
*Metapseudomonas* gen. nov.			
*Metapseudomonas resinovorans* comb. nov. (type species) (re.si.no.vo’rans. L. fem. n. *resina*, resin; L. pres. part. *vorans*, eating, devouring; N.L. part. adj. *resinovorans*, resin devouring)	*Pseudomonas resinovorans* Delaporte et al., 1961 (Approved Lists 1980).	The description of this species is the same as provided by Delaporte et al. (1961).	ATCC 14235; CCUG 2473; CCUG 4439; CFBP 5590; CIP 61.9; DSM 21078; LMG 2274; NRRL B-2649.
*Metapseudomonas boanensis* comb. nov. (bo.a.nen’sis. N.L. fem. adj. *boanensis*, pertaining to the Boane District in Mozambique)	*Pseudomonas boanensis* Nicklasson et al., 2022.	The description of this species is the same as provided by Nicklasson et al. (2022).	CCUG 62977; CECT 30359; DB1.
*Metapseudomonas furukawaii* comb. nov. (fu.ru.ka.wa’i.i. N.L. gen. masc. n. *furukawaii*, of Furukawa named after Kensuke Furukawa, a Japanese microbiologist who notably contributed to the understanding of microbial and molecular biological mechanisms involved in biphenyl/PCB degradation)	*Pseudomonas furukawaii* Kimura et al., 2018.	The description of this species is the same as provided by Kimura et al. (2018).	DSM 10086; KF707; NBRC 110670.
*Metapseudomonas lalkuanensis* comb. nov. (lal.ku.an.en’sis. N.L. fem. adj. *lalkuanensis*, pertaining to Lalkuan, a town in the Nainital district of Uttarakhand, India, where the type strain was isolated)	*Pseudomonas lalkuanensis* Thorat et al., 2020.	The description of this species is the same as provided by Thorat et al. (2020).	CCUG 73691; KCTC 72454; MCC 3792; PE08.
*Metapseudomonas otitidis* comb. nov. (o.ti’ti.dis. Gr. neut. n. *oûs* (gen. *ôtos*), ear; N.L. suff. *-itis -idis*, used in names of inflammations; N.L. gen. Fem. n. *otitidis*, of inflammation of the ear)	*Pseudomonas otitidis* Clark et al., 2006.	The description of this species is the same as provided by Clark et al. (2006).	ATCC BAA-1130; DSM 17224; MCC 10330.
*Metapseudomonas tohonis* comb. nov. (to.ho’nis. N.L. gen. n. *tohonis*, of Toho University, where the type strain was first isolated and analyzed)	*Pseudomonas tohonis* Yamada et al., 2021	The description of this species is the same as provided by Yamada et al. (2021)	GTC 22698; NCTC 14580; TUM18999
*Phytopseudomonas* gen. nov.			
*Phytopseudomonas straminea* comb. nov. (type species) (stra.mi.ne’a. L. fem. adj. *straminea*, made of straw)	*Pseudomonas straminea* corrig. Iizuka and Komagata, 1963 (Approved Lists 1980).	The description of this species is the same as provided by Iizuka and Komagata (1963).	ATCC 33636; CCUG 12539; CIP 106745; DSM 17727; IAM 1598; IFO 16665; JCM 2783; NBRC 16665; NRIC 164.
*Phytopseudomonas argentinensis* comb. nov. (ar.gen.ti.nen’sis. N.L. fem. adj. *argentinensis*, pertaining to the Argentine, of the Argentine)	*Pseudomonas argentinensis* Peix et al., 2005.	The description of this species is the same as provided by Peix et al. (2005).	CECT 7010; CH01; DSM 17259; LMG 22563.
*Phytopseudomonas daroniae* comb. nov. (da.ron.i’ae. N.L. gen. fem. n. *daroniae*, from Daron, the Celtic goddess of oak).	*Pseudomonas daroniae* Bueno-Gonzalez et al., 2019.	The description of this species is the same as provided by Bueno-Gonzalez et al. (2019).	FRB228; LMG 31088; NCPPB 4672.
*Phytopseudomonas dryadis* comb. nov. (dry.a’dis. L. gen. fem. n. *dryadis*, of a Dryad, of an oak tree nymph)	*Pseudomonas dryadis* Bueno-Gonzalez et al., 2019.	The description of this species is the same as provided by Bueno-Gonzalez et al. (2019).	FRB230; LMG 31087; NCPPB 4673.
*Phytopseudomonas flavescens* comb. nov. (fla.ves’cens. L. part. adj. *flavescens*, becoming golden yellow)	*Pseudomonas flavescens* Hildebrand et al., 1994.	The description of this species is the same as provided by Hildebrand et al. (1994).	ATCC 51555; B62; CCUG 49622; CFBP 5586; CIP 104204; DSM 12071; JCM 21586; LMG 18387; NBRC 103044; NCPPB 3063.
*Phytopseudomonas punonensis* comb. nov. (pu.no.nen’sis. N.L. fem. adj. *punonensis*, of or belonging to Puno, a region of Peru where the type strain was isolated)	*Pseudomonas punonensis* Ramos et al., 2013.	The description of this species is the same as provided by Ramos et al. (2013).	CECT 8089; DSM 27507; LMG 26839; LMT03.
*Phytopseudomonas seleniipraecipitans* comb. nov. (se.le.ni.i.prae.ci’pi.tans. N.L. neut. n. *selenium*, selenium; L. part. adj. *praecipitans*, precipitating; N.L. part. adj. *seleniipraecipitans*, selenium precipitating, referring to the organism’s ability to remove the selenium oxyanion selenite from aqueous solution)	*Pseudomonas seleniipraecipitans* corrig. Hunter and Manter, 2011.	The description of this species is the same as provided by Hunter and Manter (2011).	CA5; DSM 25106; LMG 25475; NRRL B-51283.
New name combination and etymology	Basonym	Description	Type strain
*Zestomonas* gen nov.			
*Zestomonas thermotolerans* comb. nov. (type species) (ther.mo.to’le.rans. Gr. masc. adj. *thermos*, hot; N.L. part. adj. *thermotolerans*, able to tolerate high temperatures)	*Pseudomonas thermotolerans* Manaia and Moore, 2002.	The description of this species is the same as provided by Manaia and Moore (2002).	CM3; DSM 14292; LMG 21284.
*Zestomonas carbonaria* comb. nov. (car.bo.na’ri.a. L. fem. adj. *carbonaria*, of or relating to charcoal, the source of isolation)	*Pseudomonas carbonaria* Kämpfer et al., 2021.	The description of this species is the same as provided by Kämpfer et al. (2021).	CCM 9017; CIP 111764; DSM 110367; Wesi-4.
*Zestomonas insulae* comb. nov. (in’su.lae. L. gen. fem. n. *insulae*, of an island, referring to the source of isolation of the type strain)	*Pseudomonas insulae* Lee et al., 2022.	The description of this species is the same as provided by Lee et al. (2022).	JCM 34511; KCTC 82407; UL073.
*Zestomonas cavernae* comb. nov. (*ca.*ver’nae. L. gen. n. *cavernae*, of a cave)	*Pseudomonas cavernae* Zhu et al., 2021	The description of this species is the same as provided by Zhu et al. (2021)	CGMCC 1.13586; K2W31S-8; KCTC 82191
Genus *Chryseomonas*			
*Chryseomonas oryzihabitans* comb. nov. (o.ry.zi.ha’bi.tans. L. fem. n. *oryza*, rice; L. pres. part. *habitans*, inhabiting; N.L. part. adj. *oryzihabitans*, rice inhabiting)	*Pseudomonas oryzihabitans* Kodama et al., 1985.	The description of this species is the same as provided by Kodama et al. (1985).	AJ 2197; ATCC 43272; CCUG 12540; CIP 102996; DSM 6835; IAM 1568; JCM 2952; KS0036; L-1; LMG 7040; NBRC 102199.
*Chryseomonas psychrotolerans* comb. nov. (psy.chro.to’le.rans. Gr. masc. adj. *psychros*, cold; L. pres. part. *tolerans*, tolerating; N.L. part. adj. *psychrotolerans*, cold-tolerating)	*Pseudomonas psychrotolerans* Hauser et al., 2004	The description of this species is the same as provided by Hauser et al. (2004).	C36; CCUG 51516; DSM 15758; LMG 21977.
*Chryseomonas rhizoryzae* comb. nov. (rhiz.o.ry’zae. Gr. fem. n. *rhiza*, root; L. fem. n. *oryza*, rice; N.L. gen. n. *rhizoryzae*, of rice root).	*Pseudomonas rhizoryzae* Wang et al., 2020.	The description of this species is the same as provided by Wang et al. (2020)	ACCC 61555; JCM 33201; RY24.
*Chryseomonas zeshuii* comb. nov. (ze.shu’i.i. N.L. gen. masc. n. *zeshuii*, of Ze-Shu, in honor of Ze-Shu Qian, a respected microbiologist, for his enormous contributions to promoting the development of soil microbiology in China)	*Pseudomonas zeshuii* Feng et al., 2012	The description of this species is the same as provided by Feng et al. (2012).	ACCC 5688; BY; BY-1; DSM 27927; KACC 15471.
Genus *Serpens*			
*Serpens flexibilis* comb. nov. (type species) (fle.xi’bi.lis. L. fem. adj. *flexibilis*, flexible, pliant)	*Pseudomonas flexibilis* Hespell, 1977; Shin et al., 2015.	The description of this species is the same as provided by Shin et al. (2015).	ATCC 29606; LMG 29034.
*Serpens tuomuerensis* comb. nov. (tuo.muer.en’sis. N.L. fem. adj. *tuomuerensis*, pertaining to the region of Tuomuer Peak of Tianshan Mountain, where the type strain was isolated)	*Pseudomonas tuomuerensis* Xin et al., 2009.	The description of this species is the same as provided by Xin et al. (2009).	78–123; CGMCC 1.1365; DSM 25351; JCM 14085.
Genus *Stutzerimonas*			
*Stutzerimonas marianensis* comb. nov. (ma.ri.an.en’sis. N.L. fem. adj. *marianensis*, pertaining to the Mariana Trench, the source of the type strain)	*Pseudomonas marianensis* Yang et al., 2022	The description of this species is the same as provided by Yang et al. (2022)	DSM 112238; MCCC 1 K05112; P S1

### Description of the genus *Aquipseudomonas* gen. nov.

*Aquipseudomonas* (A.qui.pseu.do.mo’nas. L. fem. n. *aqua*, water; N.L. fem. n. *Pseudomonas*, a bacterial genus; N.L. fem. n. *Aquipseudomonas, Pseudomonas*-like species isolated from water).

Cells are Gram-stain negative, motile and rod shaped. The species are aerobic in respiration and have been isolated from soil and swimming pool water. Optimum temperature for growth ranges from 30 – 37°C with <2% (w/v) NaCl and pH range from 4–10. Genome sizes for the species vary from 4.3 Mb to 4.6 Mb and the GC content ranges from 63.3 to 65.5%. Of the species from this genus, the type species *A. alcaligenes* can degrade polycyclic aromatic hydrocarbons and has been proven useful for bioremediation of oil pollution, pesticide substances, and certain chemical substances. Species from this genus form a strongly supported clade in phylogenomic tree based on large datasets of concatenated proteins. Additionally, species from this genus can be reliably distinguished from all other *Pseudomonadaceae* genera based on six CSIs (Table 2) which are exclusively found in the species from this genus. New name combinations for the species that are part of this genus are provided in Table 7.

The type species of this genus is *Aquipseudomonas alcaligenes.*

### Description of the genus *Caenipseudomonas* gen. nov.

*Caenipseudomonas* (Cae.ni.pseu.do.mo’nas. L. neut. n. *caenum*, mud; N.L. fem. n. *Pseudomonas*, a bacterial genus; N.L. fem. n. *Caenipseudomonas*, *Pseudomonas*-like organism(s) isolated from river sediments).

Cells are strictly aerobic, Gram-stain-negative, non-fluorescent and occur mostly as short rods. Cells are motile and contain a single polar flagellum. Chemoorganotrophic growth. Species have been isolated from river sediment, and wastewater sample from a pharmaceutical company. Growth occurs in the temperature range from 4-22°C with optimum growth occurring between 25-35^o^ C at pH between 7–8 in presence of 0–2% (w/v) NaCl concentration. Genome size range is from 3.3–3.4 Mb and the GC content is 62.6%. Species from this genus form a distinct lineage in phylogenomic trees based on large datasets of proteins, as well as in trees based on *rpoD* gene, or concatenated partial sequences for the 16S rDNA, *gyrB, rpoB,* and *rpoD* genes. In addition, species from this genus can be reliably distinguished based on eight exclusively shared CSIs listed in Table 5. The new name combinations for species from this genus are provided in Table 7.

The type species is *Caenipseudomonas fluvialis.*

### Description of the genus *Ectopseudomonas* gen. nov.

*Ectopseudomonas* (Ec.to.pseu.do.mo’nas. Gr. prep. *Ecto*, outside; N.L. fem. n. *Pseudomonas*, a bacterial genus; N.L. fem. n. *Ectopseudomonas*, a genus outside of *Pseudomonas*).

Cells are Gram-stain negative, motile and rod shaped. Excepting *E. chengduensis*, all other species from this genus are motile due to the presence of a polar flagellum. Species have been isolated from diverse sources including sea water, soil, hot spring, compost, and lake sediments, etc. Chemoorganotrophic life cycle. Most species grow aerobically; however, some are indicated to be facultatively anerobic. Colonies are generally brownish yellow. Growth can occur from 4^o^-42°C with optimum growth temperature between 30–37°C, with or without NaCl, in the pH range from 3.0–10.5 (optimum between pH 6–8). Genome sizes for known species vary from 4.5 Mb to 5.6 Mb and the GC content ranges from 62.2 to 65.0%. Of the species from this genus, *E. mendocina* can degrade toluene and it is indicated to cause opportunistic nosocomial infections. Members of this genus form a monophyletic clade in phylogenetic trees based on concatenated sequences of several large datasets of core genome proteins. Additionally, species from this genus also generally cluster together in phylogenetic trees based on *rpoD* gene, or concatenated partial sequences for the 16S rDNA, *gyrB, rpoB*, and *rpoD* genes. In addition of their distinct branching in phylogenetic trees, members of this genus can be reliably distinguished from other *Pseudomonadaceae* species based on five CSIs (Table 2) which in most cases are exclusively shared by the members of this genus. The new name combinations for species that are part of this genus are provided in Table 7.

The type species of this genus is *Ectopseudomonas oleovorans.*

### Description of the genus *Geopseudomonas* gen. nov.

*Geopseudomonas* (Ge.o.pseu.do.mo’nas. Gr. fem. n. *gê*, the Earth; N.L. fem. n. *Pseudomonas*, a bacterial genus; N.L. fem. n. *Geopseudomonas*, *Pseudomonas* like organisms isolated from soil).

Strictly aerobic to facultatively anaerobic, rod-shaped bacteria. Motile due to the presence of one or more polar or peritrichous flagella. Chemoorganotrophs, with cells exhibiting Gram-stain negative staining response. Cells generally do not produce fluorescent pigments. Members have been isolated from diverse sources including paddy soil, electroactive biofilm, herbicide applied wheat field and oil contaminated soil. Optimum growth occurs in the range of 30–37°C, between pH 7–8, in medium containing 1–2% NaCl (w/v). Genome lengths of the species vary from 3.2 to 4.7 Mb, and GC contents vary from 66.4 to 68.3%. Members of this genus form a monophyletic clade in phylogenetic tree based on concatenated sequences for several large datasets of proteins. Species from this genus also cluster together in phylogenetic trees based on *rpoD* gene, or concatenated partial sequences for the 16S rDNA, *gyrB, ropB,* and *rpoD* genes. In addition, the members of this genus can be reliably distinguished from all other *Pseudomonadaceae* genera by the 15 CSIs described in Table 3, which in most cases are exclusively shared by either all or most species from this genus. The new name combinations for species which are part of this genus are provided in Table 7.

The type species is *Geopseudomonas sagittaria*.

### Description of the genus *Metapseudomonas* gen. nov.

*Metapseudomonas* (Me.ta.pseu.do.mo’nas. Gr. adv. *Meta*, besides; N.L. fem. n. *Pseudomonas*, a bacterial genus; N.L. fem. n. *Metapseudomonas*, a genus beside *Pseudomonas*).

Species of this genus are Gram-negative, motile, aerobic and rod shaped. Chemoorganotrophic growth, cells do not produce fluorescent pigments. Members have been isolated from different sources such as clinical samples, soil or oil of wood mills and biphenyl contaminated soil. Optimum growth temperature is in the range of 30-37°C. Genome sizes for known species are in the range of 6.1 Mb to 6.8 Mb and GC content varies from 64.2 to 66.80%. Species from this genus form a strongly supported clade in phylogenomic trees based on large datasets of proteins. In addition, most of the species from this genus also cluster together in phylogenetic trees based on *rpoD* gene, or concatenated partial sequences for the 16S rDNA, *gyrB, ropB,* and *rpoD* genes. Importantly, the species from this genus can also be reliably distinguished from all other *Pseudomonadaceae* genera by the shared presence of five CSIs listed in Table 4. The new name combinations for the species of this genus are provided in Table 8.

The type species of this genus is *Metapseudomonas resinovorans.*

### Description of the genus *Phytopseudomonas* gen. nov.

*Phytopseudomonas* (Phy.to.pseu.do.mo’nas. Gr. neut. n. *phyton*, plant; N.L. fem. n. *Pseudomonas*, a bacterial genus; N.L. fem. n. *Phytopseudomonas*, *Pseudomonas*-like species isolated from plants).

Cells are Gram-stain negative, motile due to the presence of a polar flagellum, aerobic, and rod shaped. Chemoorganotrophs. Most species have been isolated from different plant sources such as *Quercus robur* stem tissues, straw grass, rice paddy, walnut blight cankers etc. All species produce a diffusible fluorescent pigment. Optimum temperature for growth is between 25-30°C, with <4% (w/v) or without NaCl in the pH range from 6–8. Genome sizes for the species vary from 4.5 Mb to 5.9 Mb and the GC content ranges from 61.5 to 65.0%. Members of this genus form a monophyletic clade in phylogenetic trees based on concatenated sequences of several large datasets of core genome proteins. Additionally, species from this genus also generally cluster together in phylogenetic trees based on *rpoD* gene, or concatenated partial sequences for the 16S rDNA, *gyrB, ropB*, and *rpoD* genes. Additionally, members of this genus can be reliably distinguished from other *Pseudomonadaceae* genera based on the presence of 12 CSIs summarized in Table 3. which in most cases are exclusively present in the species from this genus. The new name combinations for species that are part of this genus are provided in Table 8.

The type species of this genus is *Phytopseudomonas straminea.*

### Description of the genus *Zestomonas* gen. nov.

*Zestomonas* (Zes.to.mo’nas. Gr. masc. Adj. *zestos*, hot, boiling; L. fem. n. *monas*, a unit, monad; N.L. fem. n. *Zestomonas*, a monad that can grow at high temperature).

Aerobic, motile rods exhibiting Gram-negative staining response. Chemoorganotrophs. Species have been cultivated from different sources such as cooking water, forest soil, charcoal, and cave sediment. Temperature range for growth for species from this genus differs considerably. While the optimum growth of the type species *Zestomonas thermotolerans* occurs at 47°C (growth range 25–56°C), other species from this genus grow optimally at 28–30°C. Genome length ranges from 3.8 to 5.5 Mb and the GC content varies from 64.5 to 66.8%. Members of this genus form a monophyletic clade in phylogenomic tree based on concatenated sequences for several large datasets of proteins. In addition, members of this genus can be reliably distinguished from other *Pseudomonadaceae* genera by their uniquely sharing five CSIs listed in Table 4. New name combinations for the species from this genus are provided in Table 8.

The type species is *Zestomonas thermotolerans.*

### Emended description of the genus *Azomonas* Winogradsky, 1938 (Approved lists 1980)

*Azomonas* (A.zo.mo.nas. N.L. pref. *Azo-*, pertaining to nitrogen; L. fem. n. *monas*, a unit, monad; N.L. fem. n. *Azomonas*, nitrogen monad).

Description of this genus is in large part based on that provided by Kennedy and Rudnick (2015) in the Bergey’s Manual of Systematics of Archaea and Bacteria. Cells are Gram-stain variable or sometimes Gram-stain negative depending on the culture age, aerobic, ellipsoidal to rod shaped. Species are motile with peritrichous or lophotrichous polar flagella. Cells may occur singly, in pairs, or in clumps. All species fix atmospheric nitrogen under aerobic conditions. Alternative nitrogenases containing vanadium (nitrogenase-2) or iron (nitrogenase-3) may only be synthesized in Mo-deficient media. Cultures can grow both aerobically and microaerobically. Chemoorganotrophic. Sugars, alcohols, and organic acids are used as carbon sources. Ammonium salts and sometimes nitrate (*A. insignis* only) are used as nitrogen sources; amino acids are not used. Water-soluble and fluorescent pigments are produced by nearly all strains. Species are catalase positive. The optimum pH for nitrogen fixation is close to neutrality, but certain strains can also fix nitrogen at a pH of 4.6–4.8. Species isolated from water or soil. The G + C content of DNA from known species varies from 52.0–58.6% and their genome size ranges from 3.3 to 4.1 MB. Species belonging to this genus form a distinct clade in phylogenomic trees based on concatenated sequences of large number of proteins and in the tree based on 16S rRNA gene sequences. In addition, members of this genus can be reliably distinguished from *Azotobacter* as well as all other *Pseudomonadaceae* genera based on their exclusive sharing five CSIs described in this work (Table 5).

Type species is *Azomonas agilis* (Beijerinck, 1901) Winogradsky, 1938 (Approved Lists 1980).

### Emended description of the genus *Azotobacter* Beijerinck, 1901 (Approved lists 1980)

*Azotobacter* (A.zo.to.bac.ter. N.L. neut. n. *azotum*, nitrogen; N.L. masc. n. *bacter*, a rod; N.L. masc. n. *Azotobacter*, a nitrogen rod).

Description of this genus is in large part based on that provided by Kennedy et al. (2015) in the 2015 Bergey’s Manual of Systematics of Archaea and Bacteria. Cells range from straight rods with rounded ends to more ellipsoidal or coccoid. Motile with peritrichous flagella or nonmotile. Aerobic, having a strictly respiratory type of metabolism with oxygen as the terminal electron acceptor. Nitrogen is fixed under microaerobic conditions (2% oxygen), under full aerobiosis, or after adaptation in hyperbaric oxygen. N_2_ fixation uses Mo-, V-, or Fe-containing nitrogenase enzymes, depending on the environmental metal supply. Water-soluble and water-insoluble pigments are produced by some strains. Growth is heterotrophic; sugars, alcohols, and salts of organic acids are used as carbon sources. Ammonium salts, nitrate, and urea are used as sources of fixed nitrogen. The pH range for growth is from 4.8 to 8.5, with optimum pH for diazotrophic growth between 7.0–7.5. Most isolates are from soil, but a few are from water. The GC content of the DNA varies from 65.5–67.5%. Genome size ranges from 4.9–5.4 Mb. Species belonging to this genus group together in phylogenetic trees based on 16S rRNA gene sequences, and in phylogenomic trees based on concatenated sequences of large number of proteins. In addition, members of this genus can be reliably distinguished from all other *Pseudomonadaceae* genera by 10 uniquely shared CSIs listed in Table 5.

Type species is *Azotobacter chroococcum* Beijerinck, 1901 (Approved Lists 1980).

### Emended description of the genus *Chryseomonas* Holmes et al., 1986

*Chryseomonas* (Chry.se.o.mo’nas. Gr. masc. Adj. *chryseos*, golden; L. fem. n. *mona*s, a unit, monad; N.L. fem. n. *Chryseomonas*, a yellow unit).

The description of this genus is partially based on that given by Holmes et al. (1986) for the type species (*C. polytricha*) of this genus. The cells are rod-shaped, Gram-negative, aerobic, and exhibit chemoorganotrophic growth. Except for *C. duriflava* (and its synonym *C. zeshuii*), which do not exhibit motility, cells from the other species are motile by either a single or several polar or trichous flagella. Known species have been isolated from diverse sources including rice seeds and paddy, desert soil, herbicide-contaminated soil, grass rhizosphere, clinical specimens, and medical clinic for small animals. *C. oryzihabitans* has been reported as pathogenic to plants and animals. Some species (*C. luteola*) can reduce nitrate. Growth can occur in the temperature range from 4–42°C with optimum growth occurring between 30 to 37°C at pH 7.0 (pH range 6–8) in medium supplemented with 1–2% (w/v) NaCl. The cells are catalase positive but oxidase negative. The GC content of species varies from 53.6 to 66.2% and their genome lengths range from 4.3 to 5.4 Mb. Species from this genus form a distinct clade in the phylogenomic trees based on a large number of proteins. Additionally, these species also cluster together in phylogenetic trees based on *rpoD* gene, or concatenated partial sequences for the 16S rDNA, *gyrB, ropB,* and *rpoD* genes. Apart from their grouping together in phylogenetic trees, species from this genus can be reliably distinguished from all other *Pseudomonadaceae* genera by their 11 CSIs listed in Table 4, which in most cases are exclusively present in the species from this genus. New name combinations for four *Pseudomonas* species, which are transferred to this genus, are provided in Table 8.

Type species of this genus is *Chryseomonas polytrichia* (Holmes et al., 1986).

### Emended description of the genus *Serpens* Hespell, 1977 (Approved lists 1980)

Description of this genus is modified from that given by Hespell (1977). Gram-negative, aerobic, rod-shaped, non-spore forming, bacterial cells. Cells from the type species, *S. flexibilis*, are very flexible, and motile due to containing a flagellum, and exhibit serpentine-like movement in agar gels. Metabolism is respiratory, and molecular oxygen serves as the terminal electron acceptor. *S. flexibilis* mainly uses lactate as the energy and carbon source. Catalase and oxidase are produced. Temperature range for optimal growth is from 28 to 37°C. The G + C content of DNA ranges from 61.0–65.8 mol% and genome size varies from 3.8–3.9 Mb. Species from this genus form a monophyletic clade in the phylogenetic tree based on large dataset of proteins. The type species also forms a distinct lineage in phylogenetic trees based on *rpoD* gene, or concatenated partial sequences for the 16S rDNA, *gyrB, ropB,* and *rpoD* genes. Additionally, species from this genus can be reliably distinguished from other *Pseudomonadaceae* genera by the presence of three exclusively shared CSIs (Table 4). New name combinations for the two species which are part of this genus are provided in Table 8.

Type species of this genus is *Serpens flexibilis* Hespell, 1977 (Approved lists).

### Emended description of the genus *Stutzerimonas* Lalucat et al., 2022

*Stutzerimonas* (Stut.ze.ri.mo’nas. L. fem. n. *monas*, a unit, monad; N.L. fem. n. *Stutzerimonas*, monad of Stutzer, named in honor of Albert Stutzer, who in 1895 described the bacterium today known).

The description of this genus, especially in terms of its morphological, chemotaxonomic and growth characteristics, remains the same as provided by Lalucat et al. (2022). In addition to the genomic characteristics described by Lalucat et al. (2022), members of this genus can be reliably distinguished from other *Pseudomonadaceae* genera by seven novel CSIs identified in this study (listed in Table 3), which in most cases are exclusively found in the species from this genus. New name combination for *P. marianensis* (Table 8) is based on its branching in the 16S rRNA gene tree (Yang et al., 2022).

The type species is *Stutzerimonas stutzeri* (Lehmann and Neumann 1896) Lalucat et al. 2022.

## Data availability statement

The datasets presented in this study can be found in online repositories. The names of the repository/repositories and accession number(s) can be found in the article/Supplementary material.

## Author contributions

BR: Data curation, Formal analysis, Investigation, Methodology, Writing – original draft, Validation. RG: Conceptualization, Funding acquisition, Project administration, Resources, Software, Supervision, Validation, Writing – review & editing.

## Footnotes

^1^https://www.ncbi.nlm.nih.gov/genome/

^2^http://gtdb.ecogenomic.org/

^3^AppIndels.com
