# Supplementary material for: Phylogenomics studies and molecular markers reliably demarcate genus Pseudomonas sensu stricto and twelve other Pseudomonadaceae species clades representing novel and emended genera
Source: Front Microbiol. 2024 Jan 5;14:1273665. doi: 10.3389/fmicb.2023.1273665 (PMC10797017; doi:10.3389/fmicb.2023.1273665)
Supplement: Supplementary file 4 [file Data_Sheet_1.PDF]

Fluorescens Superclade

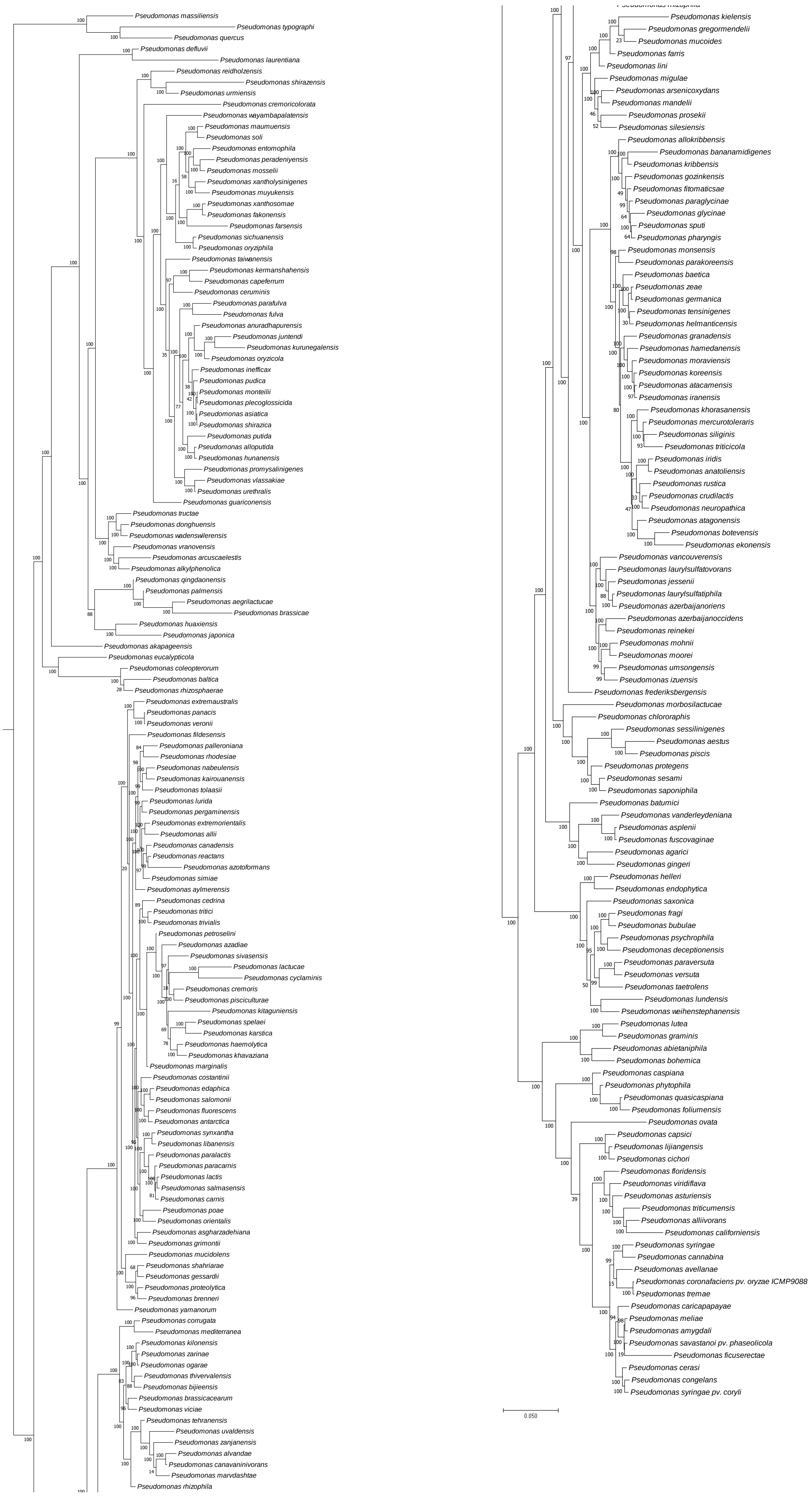

Figure-S1:

Uncompressed form of the different species from Fluorescens Superclade shown in Figure-1.

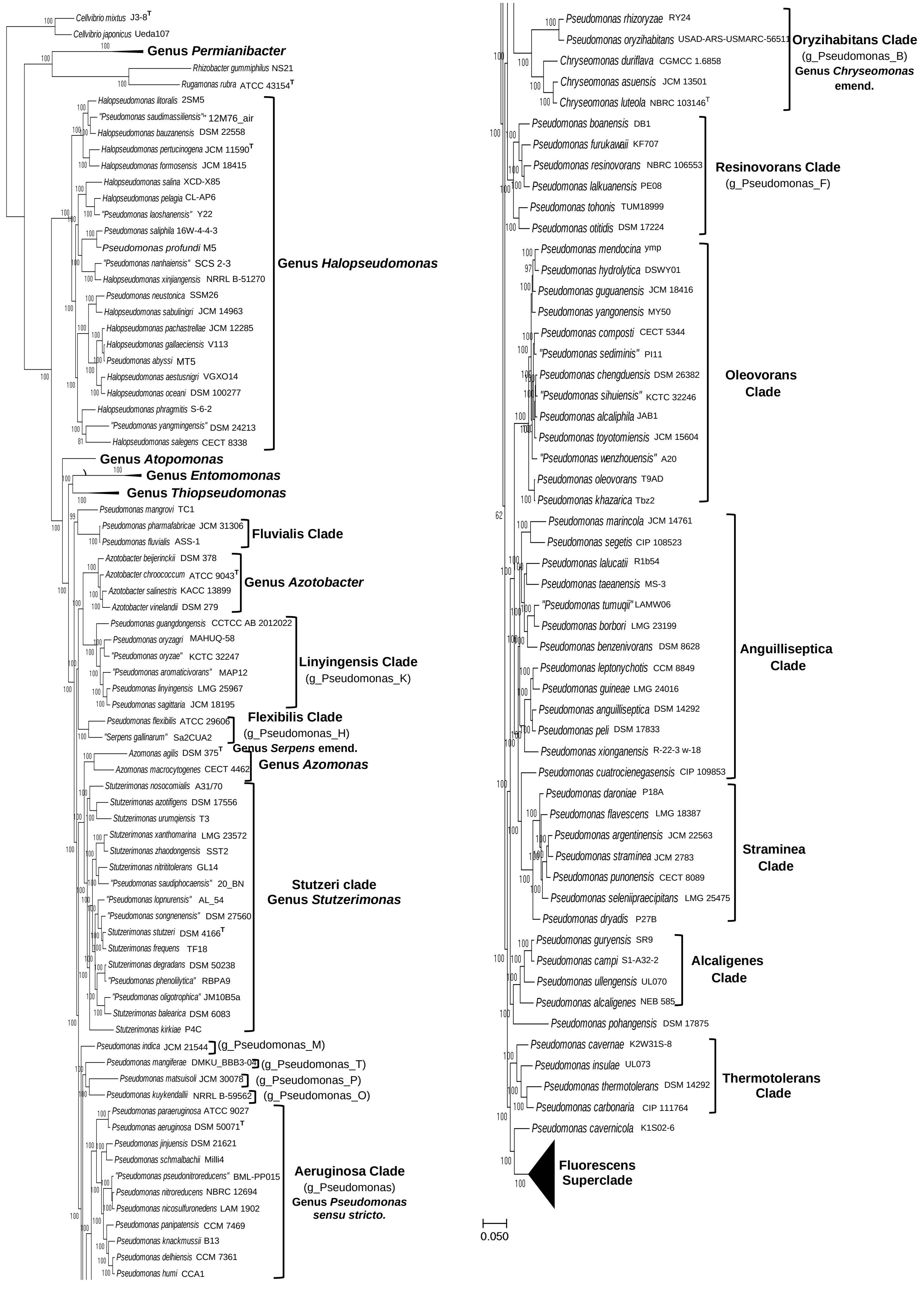

Cellvibrio mixtus J3-8T
Cellvibrio japonicus Ueda107
Genus Permianibacter
Rhizobacter gummiphilus NS21
Rugamonas rubra ATCC 43154T
Halopseudomonas litoralis 2SM5
"Pseudomonas saudimassiliensis" 12M76_air
Halopseudomonas bauzanensis DSM 22558
Halopseudomonas pertucinogena JCM 11590T
Halopseudomonas formosensis JCM 18415
Halopseudomonas salina XCD-X85
Halopseudomonas pelagia CL-AP6
"Pseudomonas laoshanensis" Y22
Pseudomonas saliphila 16W-4-4-3
Pseudomonas profundi M5
"Pseudomonas nanhaiensis" SCS 2-3
Halopseudomonas xinjiangensis NRRL B-51270
Pseudomonas neustonica SSM26
Halopseudomonas sabulinigri JCM 14963
Halopseudomonas pachastrellae JCM 12285
Halopseudomonas gallaeciensis V113
Pseudomonas abyssi MT5
Halopseudomonas aestusnigri VGXO14
Halopseudomonas oceani DSM 100277
Halopseudomonas phragmitis S-6-2
"Pseudomonas yangmingensis" DSM 24213
Halopseudomonas salegens CECT 8338
Genus Halopseudomonas
Genus Atopomonas
Genus Entomomonas
Genus Thiopseudomonas
Pseudomonas mangrovi TC1
Pseudomonas pharmafabricae JCM 31306
Pseudomonas fluvialis ASS-1
Fluvialis Clade
Azotobacter beijerinckii DSM 378
Azotobacter chroococcum ATCC 9043T
Azotobacter salinestris KACC 13899
Azotobacter vinelandii DSM 279
Genus Azotobacter
Pseudomonas guangdongensis CCTCC AB 2012022
Pseudomonas oryzagri MAHUQ-58
"Pseudomonas oryzae" KCTC 32247
"Pseudomonas aromaticivorans" MAP12
Pseudomonas linyingensis LMG 25967
Pseudomonas sagittaria JCM 18195
Linyingensis Clade
(g_Pseudomonas_K)
Pseudomonas flexibilis ATCC 29606
"Serpens gallinarum" Sa2CUA2
Flexibilis Clade
(g_Pseudomonas_H)
Genus Serpens emend.
Azomonas agilis DSM 375T
Azomonas macrocytogenes CECT 4462
Genus Azomonas
Stutzerimonas nosocomialis A31/70
Stutzerimonas azotifigens DSM 17556
Stutzerimonas urumqiensis T3
Stutzerimonas xanthomarina LMG 23572
Stutzerimonas zhaodongensis SST2
Stutzerimonas nitrititolerans GL14
"Pseudomonas saudiphocaensis" 20_BN
"Pseudomonas lopnurensis" AL_54
"Pseudomonas songnenensis" DSM 27560
Stutzerimonas stutzeri DSM 4166T
Stutzerimonas frequens TF18
Stutzerimonas degradans DSM 50238
"Pseudomonas phenolilytica" RBPA9
"Pseudomonas oligotrophica" JM10B5a
Stutzerimonas balearica DSM 6083
Stutzerimonas kirkiae P4C
Stutzeri clade
Genus Stutzerimonas
Pseudomonas indica JCM 21544
(g_Pseudomonas_M)
Pseudomonas mangiferae DMKU_BBB3-04
(g_Pseudomonas_T)
Pseudomonas matsuisoli JCM 30078
(g_Pseudomonas_P)
Pseudomonas kuykendallii NRRL B-59562
(g_Pseudomonas_O)
Pseudomonas paraeruginosa ATCC 9027
Pseudomonas aeruginosa DSM 50071T
Pseudomonas jinjuensis DSM 21621
Pseudomonas schmalbachii Milli4
"Pseudomonas pseudonitroreducens" BML-PP015
Pseudomonas nitroreducens NBRC 12694
Pseudomonas nicosulfuronedens LAM 1902
Pseudomonas panipatensis CCM 7469
Pseudomonas knackmussii B13
Pseudomonas delhiensis CCM 7361
Pseudomonas humi CCA1
Aeruginosa Clade
(g_Pseudomonas)
Genus Pseudomonas sensu stricto.
Pseudomonas rhizoryzae RY24
Pseudomonas oryzihabitans USAD-ARS-USMARC-56511
Chryseomonas duriflava CGMCC 1.6858
Chryseomonas asuensis JCM 13501
Chryseomonas luteola NBRC 103146T
Oryzihabitans Clade
(g_Pseudomonas_B)
Genus Chryseomonas emend.
Pseudomonas boanensis DB1
Pseudomonas furukawaii KF707
Pseudomonas resinovorans NBRC 106553
Pseudomonas lalkuanensis PE08
Pseudomonas tohonis TUM18999
Pseudomonas otitidis DSM 17224
Resinovorans Clade
(g_Pseudomonas_F)
Pseudomonas mendocina ymp
Pseudomonas hydrolytica DSWY01
Pseudomonas guguanensis JCM 18416
Pseudomonas yangonensis MY50
Pseudomonas composti CECT 5344
"Pseudomonas sediminis" PI11
Pseudomonas chengduensis DSM 26382
"Pseudomonas sihuiensis" KCTC 32246
Pseudomonas alcaliphila JAB1
Pseudomonas toyotomiensis JCM 15604
"Pseudomonas wenzhouensis" A20
Pseudomonas oleovorans T9AD
Pseudomonas khazarica Tbz2
Oleovorans Clade
Pseudomonas marincola JCM 14761
Pseudomonas segetis CIP 108523
Pseudomonas lalucatii R1b54
Pseudomonas taeanensis MS-3
"Pseudomonas tumuqii" LAMW06
Pseudomonas borbori LMG 23199
Pseudomonas benzenivorans DSM 8628
Pseudomonas leptonychotis CCM 8849
Pseudomonas guineae LMG 24016
Pseudomonas anguilliseptica DSM 14292
Pseudomonas peli DSM 17833
Pseudomonas xionganensis R-22-3 w-18
Pseudomonas cuatrocienegasensis CIP 109853
Anguilliseptica Clade
Pseudomonas daroniae P18A
Pseudomonas flavescens LMG 18387
Pseudomonas argentinensis JCM 22563
Pseudomonas straminea JCM 2783
Pseudomonas punonensis CECT 8089
Pseudomonas seleniipraecipitans LMG 25475
Pseudomonas dryadis P27B
Straminea Clade
Pseudomonas guryensis SR9
Pseudomonas campi S1-A32-2
Pseudomonas ullengensis UL070
Pseudomonas alcaligenes NEB 585
Alcaligenes Clade
Pseudomonas pohangensis DSM 17875
Pseudomonas cavernae K2W31S-8
Pseudomonas insulae UL073
Pseudomonas thermotolerans DSM 14292
Pseudomonas carbonaria CIP 111764
Thermotolerans Clade
Pseudomonas cavernicola K1S02-6
Fluorescens Superclade
0.050

Figure-S2:

A ML phylogenetic tree for 174 genome sequenced *Pseudomonadaceae* species based on concatenated sequences for 1503 core proteins. Species from the Fluorescens Superclade are shown in compressed form. The different species clades are demarcated and labelled as in Figure 1.

Group	Species	Accession	126		156
Aeruginosa Clade (Genus *Pseudomonas sensu stricto*) (14/14)	*Pseudomonas aeruginosa*	WP_058144759	WVLQPVQWRFIGGFGAIHWL	GA	ERVPLANPF
	Pseudomonas citronellolis	WP_116422573	---------------D----	--	DS-------
	Pseudomonas delhiensis	WP_089390675	---------------D----	--	DS-------
	Pseudomonas humi	WP_061560790	---------------D----	--	DS-------
	Pseudomonas jinjuensis	WP_084309462	---E-----------D----	A-	DS-------
	Pseudomonas knackmussii	WP_043254549	---------Y-----D----	A-	DS--QP-V-
	Pseudomonas multiresinivorans	WP_169941600	---E-----------D----	--	DS-------
	Pseudomonas nicosulfuronedens	WP_138521353	---E-----------D---L	G-	NS-------
	Pseudomonas nitritireducens	WP_184589134	---------------D----	--	DS-------
	Pseudomonas nitroreducens	WP_084358285	---E-----------D----	--	DS-------
	Pseudomonas panipatensis	WP_090265726	---------Y-----D----	--	DS-T-D---
	Pseudomonas paraeruginosa	AWE92320	--------------------	--	---------
	Pseudomonas pseudonitroreducens	WP_236200795	---E-----------D----	--	DS-------
	Pseudomonas schmalbachii	MBO3277649	---E-----------D---I	A-	DS-------
Other *Pseudomonas*	*Pseudomonas abietaniphila*	WP_074755438	---A--RY-Y----------		DD-A-----
	Pseudomonas agarici	WP_017131651	---K--RH-Y-----E----		DQ-T-----
	Pseudomonas alcaligenes	WP_203791195	---Q--R-------------		QD-E-----
	Pseudomonas alcaliphila	WP_074679364	-R-E--R-------------		DQ-A-----
	Pseudomonas allii	NWN61633	---R--RH-Y---------V		DQLT-----
	Pseudomonas asiatica	WP_137162359	------RH-Y----------		DQ-T-----
	Pseudomonas asturiensis	WP_073168237	---E--RY-Y----------		DE-A-----
	Pseudomonas batumici	WP_040064826	---K--RH-Y----------		DQ-T-----
	Pseudomonas borbori	WP_090503425	---E--R--Y----------		DQ-A-----
	Pseudomonas capeferrum	WP_181130252	---E-IRH-Y----------		DQ-T-----
	Pseudomonas caspiana	WP_087264829	---V--RY------------		DH-E-----
	Pseudomonas cavernicola	WP_119952172	---E--RA-Y----------		NS-T-----
	Pseudomonas chengduensis	WP_021489384	-R-E--R-------------		DQ-A-----
	Pseudomonas cichorii	WP_025261690	---T--RY-Y----------		DD-A-----
	Pseudomonas composti	WP_179526784	-R-E--R-------------		DQ-T-----
	Pseudomonas cremoricolorata	WP_038412211	---E--RH-Y----------		DQ-T-----
	Pseudomonas cremoris	MBC2382130	---K--RH-Y---------V		DQLT-----
	Pseudomonas entomophila	WP_011535516	------RH-Y----------		DQ-T-----
	Pseudomonas flexibilis	WP_039606456	------R--------S----		-A-SQ----
	Pseudomonas floridensis	WP_083182061	---A--RY-Y----------		DD-A-----
	Pseudomonas fluorescens	WP_158464872	------RH-Y----------		DQ-T-----
	Pseudomonas fluvialis	MBB6341489	-R----R-------------		-Q-A-----
	Pseudomonas fragi	WP_095031258	---N--RH-Y----------		DQ-T-----
	Pseudomonas fulva	WP_167338518	---E--RH-Y----------		DQ-T-----
	Pseudomonas fuscovaginae	WP_054057581	---E--RH-Y----------		DQ-T-----
	Pseudomonas gingeri	WP_177107218	---K--RH-Y----------		DQ-T-----
	Pseudomonas graminis	WP_172609561	---K--RY------------		DDAA-----
	Pseudomonas indica	WP_084336670	---E--R--Y----------		DE-A-----
	Pseudomonas indoloxydans	WP_108234443	-R-E--R-------------		DQ-A-----
	Pseudomonas kitaguniensis	MPQ85887	---K--RH-Y---------V		DQLT-----
	Pseudomonas kunmingensis	WP_090522247	---E--R--Y----------		DHLA-----
	Pseudomonas kuykendallii	WP_090228833	---Q--R--Y----------		D--E-----
	Pseudomonas laurentiana	NES12021	---K--RY-Y----------		DH-A-----
	Pseudomonas lutea	WP_037014634	---K--RY-Y----------		DN-A-S---
	Pseudomonas mangiferae	WP_143486942	---E--RS-Y-----E----		NV-TRV---
	Pseudomonas mangrovi	WP_108104345	-R----R-------------		DQ-A-----
	Pseudomonas mendocina	WP_115298345	-R----R-------------		DQ-A-----
	Pseudomonas monteilii	WP_104833374	------RH-Y----------		DQ-T-----
	Pseudomonas mosselii	WP_028689690	---E--RH-Y----------		DQ-T-S---
	Pseudomonas oleovorans	PZR35536	-R-E--R-------------		DQ-A-----
	Pseudomonas parafulva	WP_116888980	---E--RH-Y----------		DQ-T-----
	Pseudomonas plecoglossicida	WP_108480661	------RH-Y----------		DQ-T-----
	Pseudomonas pohangensis	WP_090193054	-Q----RA-Y----------		DQ-T-G---
	Pseudomonas pohangensis	WP_090193054	-Q-Q--RA-Y----------		DQ-T-G---
	Pseudomonas putida	WP_012312888	------RH-Y----------		DQ-T-----
	Pseudomonas putida	WP_070092365	---E--RH-Y----------		DQ-T-----
	Pseudomonas resinovorans	WP_077526661	-R----RA------------		DQLV---A-
	Pseudomonas savastanoi	WP_122398468	---V--RY------------		DH-E-----
	Pseudomonas sediminis	WP_179544055	-R-E--R-------------		DE-T-----
	Pseudomonas songnenensis	WP_122098191	---E--R--Y----------		DD-A-----
	Pseudomonas stutzeri	WP_133456338	---E--R--Y----------		DQ-A-----
	Pseudomonas syringae	WP_065832646	---T--RY------------		DN-A-----
	Pseudomonas toyotomiensis	WP_059392606	-R-E--R-------------		DQ-A-----
	Pseudomonas viridiflava	WP_122696999	---E--RY-Y----------		DE-A-----
	Pseudomonas zhaodongensis	WP_122164610	-I-K--R-------------		DQ-A-----
Other Bacteria	*Aeromonas caviae*	GJB82802	------RH-Y----------		DQ-T-----
	Agitococcus lubricus	WP_107864470	-RIK--KY-Y-----Q----		QPHPISS--
	Agitococcus sp.	MBP9216611	-R-TA-KY-Y-----R---A		-PSVIS---
	Arthrobacter frigidicola	RJT88826	------RH-Y----------		DQ-T-P---
	Azotobacter beijerinckii	WP_090619462	--ME--R--------S----		-G-E-----
	Azotobacter chroococcum	WP_131256439	---E--R--------S----		-G-E-----
	Azotobacter chroococcum	WP_131300250	---E--R--------S----		-G-E-----
	Azotobacter chroococcum	WP_169531529	---E--R--------S----		-G-E-----
	Azotobacter salinestris	WP_152386968	---E--R--------S----		-G-E-----
	Azotobacter vinelandii	WP_175555906	---E--R--------S----		-G-E-----
	Diploscapter pachys	PAV74170	---E--RH-Y----------		DQ-T-----
	Escherichia coli	WP_153670543	------RH-Y----------		DQ-T-----
	Moraxellaceae bacterium	MBK6756175	-YIE-IKY-Y-----K---A		-PLLV----
	Paucimonas lemoignei	SQF95828	---A--RY------------		DH-A-----

**Other Bacteria**	*Priestia aryabhattai*	QPN46425	------RH-Y----------	DQ-T-----
	Stenotrophomonas rhizophila	AXQ49581	------RH-Y----------	DQ-T-----
	Streptococcus pneumoniae	CJK99346	---E--R--Y----------	DD-A-----
	Vibrio cholerae	WP_213421012	---K--RH-Y---------V	DQLT-----

Figure-S3

Partial sequence alignments of HugZ family protein showing a 2aa Ins (highlighted), which is uniquely shared by all species from the Aeruginosa clade. The dashes (-) in all sequence alignments indicate identity with the amino acids on the top line..

Group	Species	Accession	68		104
**Aeruginosa Clade** (Genus *Pseudomonas sensu stricto*) (14/14)	*Pseudomonas aeruginosa*	WP_162953821	LEKELDRRQAKPEAQHA	T	LEDLLHLLVSQAMAVKPRS
	Pseudomonas citronellolis	WP_058488916	------------D-PR-	S	--E--E---V---------
	Pseudomonas delhiensis	KAF1056474	-----------SD-PR-	S	--E--E---L---------
	Pseudomonas humi	WP_009621360	------------D-PR-	S	--E--E---V---------
	Pseudomonas jinjuensis	WP_084310013	----------R-D-PR-	-	--E--E--AT-TL--T---
	Pseudomonas knackmussii	WP_043253481	-----------TD-PR-	S	--E--E---V---------
	Pseudomonas multiresinivorans	WP_169939736	------------D-PR-	-	--E--E---I---------
	Pseudomonas nicosulfuronedens	WP_138526002	------------D-PR-	-	--E--E---V-----K---
	Pseudomonas nitritireducens	WP_184586944	------------D-PR-	-	--E--E---V---------
	Pseudomonas nitroreducens	WP_088416908	------------D-PR-	-	--E--E---V---------
	Pseudomonas panipatensis	WP_090260612	------------D-PRP	-	--E--E---V---------
	Pseudomonas paraeruginosa	WP_003091195	-----------------	-	-------------------
	Pseudomonas pseudonitroreducens	WP_236170899	--------------PR-	-	--E--E---V---------
	Pseudomonas schmalbachii	WP_208314710	------------D-PR-	-	--E--E--AT-I---T---
**Other** ***Pseudomonas***	*Pseudomonas abietaniphila*	WP_074749674	-D---E----R--HKPT		--E--DM--E--LV-Q---
	Pseudomonas agarici	WP_017134132	-DR--EQ-L-R--VKSS		-----EI--E--LV-Q---
	Pseudomonas alcaligenes	TXI32737	--R-----M-N-DNKPQ		--AH-E---E--L------
	Pseudomonas alcaliphila	WP_074675259	-----------S-T-VS		--E--E---E--L------
	Pseudomonas alkylphenolica	WP_157191841	--R--E----R--HKPS		--E--EM--E--LV-Q---
	Pseudomonas amygdali	WP_122309510	-DR--E-------YKPS		--E--EI--E--LV-Q---
	Pseudomonas anguilliseptica	WP_090387339	--R----------TKVS		--E--E---E--L------
	Pseudomonas argentinensis	WP_074880311	--R---K--S--GVTFS		--E--E---E--L------
	Pseudomonas antarctica	WP_083359557	-DR--E---T---NKPS		-----EI--E--LV-Q---
	Pseudomonas azotifigens	WP_181069671	--------ESESDVPDT		--Q--EI--E-TI------
	Pseudomonas benzenivorans	WP_090442283	--R-----------KPS		--E--E---E--L------
	Pseudomonas balearica	WP_043220813	--R-----E-LAGKPDT		--E--GI--E--L------
	Pseudomonas borbori	WP_090497374	-DR--------Q--KAS		--E--E---D--L------
	Pseudomonas brassicae	WP_163945166	--R--E----R--QKPT		--E--EM--E--LV-Q---
	Pseudomonas brassicacearum	WP_123425847	-DR--E------DNRPS		--E--EI--E--LV-Q---
	Pseudomonas cedrina	WP_076950289	-DR--E-----ADHKPT		-----EI--E--LV-Q---
	Pseudomonas cichorii	WP_117187004	--R--E-------QKPS		--E--EM--E--LV-Q---
	Pseudomonas composti	WP_061240311	-----------S-T-VS		--E--E---E--L------
	Pseudomonas congelans	WP_096130866	-DR--E-------HKPS		--E--EI--E--LV-Q---
	Pseudomonas corrugata	WP_024777919	-DR--E------DVKPT		--E--EM--E--L--Q---
	Pseudomonas costantinii	WP_177012048	-DR--E---T---NKPS		-----EI--E--LV-Q---
	Pseudomonas cremoricolorata	WP_038414190	--R--E----R--LKPS		--E--EM--D--L--Q---
	Pseudomonas daroniae	WP_131178368	--R---K--S--GVTFS		--E--E---E--L------
	Pseudomonas dryadis	WP_131173740	--R------G--GTTFT		--E--E---E--L------
	Pseudomonas duriflava	WP_145142378	-DT-----YG-SGTRPD		--E-FY---D--L-I---H
	Pseudomonas edaphica	WP_138449563	-DR--E---T---NKPS		-----EI--E--LV-Q---
	Pseudomonas entomophila	WP_181091378	--R--EQ---R--R-DS		--A--EM--E--L--Q---
	Pseudomonas fildesensis	WP_048725717	-DR--E-----ADNKPS		-----EI--E--LV-Q---
	Pseudomonas fluorescens	WP_038442180	-DR--E------DNKPS		-----EI--E--LV-Q---
	Pseudomonas fluvialis	WP_184681323	--R------G---QAAN		--E--E---A--L------
	Pseudomonas fulva	WP_182138946	--R--E----R--HKPT		--E--EM--E--L--Q---
	Pseudomonas fuscovaginae	WP_054057091	-DR--E-------VKPT		--E--EI--E--LV-Q---
	Pseudomonas gingeri	WP_177086576	-DR--E-------VKPT		--E--EI--E--LV-Q---
	Pseudomonas grimontii	WP_090407767	-DR--E-----ADHKPS		-----EM--E--LV-Q---
	Pseudomonas guariconensis	WP_090343875	--R-------R--PKPS		--E--EM--E--L--Q---
	Pseudomonas guguanensis	WP_090426218	-----------A-T-AT		--E--E---E--L------
	Pseudomonas guineae	SFH76423	--R--------S-TKVD		--E--E---E--LT-----
	Pseudomonas haemolytica	WP_153870487	-DR--E-----ADHKPS		-----EI--E--LV-Q---
	Pseudomonas hydrolytica	WP_129481452	-----------ADT-AT		--E--E---E--L------
	Pseudomonas indica	WP_084334039	--R------T---SHAS		--E--EI--E--L------
	Pseudomonas japonica	WP_042124347	--R-------R--HKPN		--E--DM--E--LV-Q---
	Pseudomonas juntendi	WP_182388294	--R--E-------HKPS		--E--EM--E--L--Q---
	Pseudomonas khazarica	WP_134676241	-----------T-N-AT		--E--E---E--L------
	Pseudomonas kirkiae	WP_131184825	-------CE-QASQKDR		-----EM--V--LS-----
	Pseudomonas kitaguniensis	WP_152749728	-DR--E---T---NTPS		-----EI--E--LV-Q---
	Pseudomonas kairouanensis	WP_135288417	-DRD-E-----ADNKPT		-----EM--E--LV-Q---
	Pseudomonas kuykendallii	PZP22862	--R--E-------EKAS		--E--E---D--L------
	Pseudomonas leptonychotis	WP_136665088	--R----------SKVS		--E--E---E--L------
	Pseudomonas lundensis	WP_070415195	-DR--E----R-DSRPK		-----EM--E--LVIQ---
	Pseudomonas luteola	WP_019365906	--N-----YG-SGTKAD		--E--Y---D--L-I---H
	Pseudomonas mandelii	WP_133836763	-D---E-------NKPT		-----EI--E--LV-Q---
	Pseudomonas mangiferae	WP_143488169	--R------G-G-TRAS		--E--EI--E--L------
	Pseudomonas marginalis	WP_122252165	-DR--E---T---NTPS		-----EI--E--LV-Q---
	Pseudomonas massiliensis	WP_040260713	-DR--E--A-N-GHKAS		--E--EI--D--LS-Q---
	Pseudomonas matsuisoli	WP_188981458	--Q-----EGVA-VSYT		-----G-I-E-MLVI---N
	Pseudomonas mendocina	WP_047586418	-----------T-N-AT		--E--E---E--L------
	Pseudomonas monteilii	WP_060393279	--R--EQ---R--R-DS		--A--EM--E--L--Q---
	Pseudomonas nabeulensis	WP_135306775	-DR--E-----ADNKPS		-----EM--E--LV-Q---
	Pseudomonas nosocomialis	WP_138407221	--------E-ESDVPDT		--Q--GI--E-T-------
	Pseudomonas oleovorans	PZQ44202	-----------S-T-VS		--E--E---E--L------
	Pseudomonas otitidis	WP_160481169	--R---K--G-S-TPPS		--E--VM--E--L------

Other *Pseudomonas*	*Pseudomonas panacis*	WP_154843313	-DR--E------DNKPS	-----EI--E--LV-Q---
	Pseudomonas parafulva	WP_039578781	--R--E----Q---KPS	--E--EM--E--LV-Q---
	Pseudomonas poae	AZP73166	-DR--E-----ADSKPT	-----EM--E--LV-Q---
	Pseudomonas plecoglossicida	WP_016391291	--R--E-------QKPS	--E--EM--E--L--Q---
	Pseudomonas putida	WP_038409178	--R--E-------HKPS	--E--DM--E--L--Q---
	Pseudomonas punonensis	WP_073264591	--R---K--S--GVTFS	--E--E---E-TL------
	Pseudomonas resinovorans	WP_028629476	--R--------A-SKAS	--E--EM--E--L------
	Pseudomonas sediminis	WP_099526446	-----------SDT-VS	--E--E---E--L------
	Pseudomonas seleniipraecipitan	WP_092367554	--R---K--S--GITFS	--E--E---E--L------
	Pseudomonas stutzeri	WP_181095729	--R-----E-QSDRKES	--E--GI--E--L------
	Pseudomonas synxantha	WP_124376935	-DR--E-----ADH-PS	-----EI--E--LV-Q---
	Pseudomonas taeanensis	WP_025167381	--R-----------KIS	--E--E---E--LI-----
	Pseudomonas taiwanensis	WP_179060009	--R------S-A-SKAS	--E--EM--E--L------
	Pseudomonas thermotolerans	WP_017939223	---------ANADYKAS	--E--EI--E--M------
	Pseudomonas pohangensis	WP_090195149	--R------ANS-YKAS	--E--E---EH-L-I---I
	Pseudomonas trivialis	WP_057009564	-DR--E----RADHKPS	-----EM--E--LV-Q---
	Pseudomonas tolaasii	WP_177005916	-DR--E---T---HKPS	--E--EI--E--LV-Q---
	Pseudomonas xinjiangensis	WP_093392566	--A----QE-SGA-DPS	--E--EM--R--LQ-----
	Pseudomonas yamanorum	WP_177045651	-DR--E-------NKPS	-----EI--E--LV-Q---
	Pseudomonas viridiflava	WP_122551825	--R--E-------HKPT	-----EI--E--LV-Q---
Other Bacteria	*Azotobacter chroococcum*	WP_165892400	--R----H--A--PGPS	--E--EITIT-VQSAR---
	Azotobacter beijerinckii	WP_090898467	--R-------S--GGMG	--E--EVTIE-VQSARLC-

Figure-S4

Partial sequence alignments of TetR family transcriptional regulator protein showing a 1aa insertion (highlighted), which is uniquely shared by all species from the Aeruginosa clade.

Group	Species	Accession	39		83
Aeruginosa Clade (Genus *Pseudomonas sensu stricto*) (14/14)	*Pseudomonas delhiensis*	WP_089389603	LRVLDELERQLQATLPVATS	D	TEQAQLLLRRLNELGFAEDD
	Pseudomonas aeruginosa	VTM08501	Q-T----A--IG-S-DDRA-	A	S-R--G----MS-Q--C---
	Pseudomonas citronellolis	WP_058072759	-----D-Q----------AG	-	I-------------------
	Pseudomonas humi	WP_069863456	-----D-Q----------AG	-	I-------------------
	Pseudomonas jinjuensis	WP_084309796	RPTF-G-LQ-VRLG-AEGAD	-	A-R--L-----AS---S---
	Pseudomonas knackmussii	WP_160285872	M-M--D-AQR-F----LDGT	A	A-R------Q-S-Q------
	Pseudomonas multiresinivorans	WP_169936858	--LF-S-AH-VAVA--QG-G	-	A-R--F-----S--------
	Pseudomonas nicosulfuronedens	WP_138522052	--LF-S-AH-VAVA--QG--	A	A-R--F-----S--------
	Pseudomonas nitritireducens	WP_184593692	--LF-S-AH-VAVA-SQGAG	-	A-R--F-----S--------
	Pseudomonas nitroreducens	WP_138216087	--LF-S-AH-VAVA--QG-G	-	A-R--F-----S--------
	Pseudomonas panipatensis	WP_090261558	-HAV-N-AH-VT-G--LLEG	-	A-R--A-----S--------
	Pseudomonas paraeruginosa	WP_003138209	--E----V--IG-S-DDRA-	A	S-R--G----MS-Q--C---
	Pseudomonas pseudonitroreducens	WP_236172834	--LF-S-AH-VAVA--QGSG	-	A-R--F-----S-L--A---
	Pseudomonas schmalbachii	WP_208314353	RPA--S-L--VRID--EGN-	-	V-R--W----MSG-------
Other *Pseudomonas*	*Pseudomonas veronii**	WP_155677219	-QE-NV-QQ-VSLGM-LLPA	-	EVSG-P------D----Q--
	Pseudomonas alcaligenes	WP_043239938	--DVAS-TQ-VA-G--QLPA		I-L--P-----S--D-N---
	Pseudomonas antarctica	WP_064450834	-QE-IV-QQ-VSVGM-MLPA		D-LG-P----M-D----Q--
	Pseudomonas argentinensis	WP_070887560	MGE-EN-KQ-VG-G--NFPP		Q-L--P-----SD-H-Y---
	Pseudomonas asiatica	WP_137162395	-AK-HD-Q-EIS-N--MLPL		C-L--P---Q--A---QQ-E
	Pseudomonas asplenii	WP_090205192	--DFKD-QQRVS-G--MLPV		S-L--P----M-----QQ-E
	Pseudomonas azotoformans	WP_071494035	-QE-TL-HQ-VSIGM-MLPA		D-LG-P------D----Q-E
	Pseudomonas batumici	WP_040064350	--DFRD-QQRVS-G--MLPV		S-L--P----M-D---QQ-E
	Pseudomonas benzenivorans	WP_090448479	--E-GA-QQRVG-G--SLPA		A-L--P-----SA-D-H---
	Pseudomonas brassicacearum	WP_123582723	-EQFKD-QQRVS-G--MLPV		S-L--P------DM-YRQ-E
	Pseudomonas carnis	WP_127880855	-HD-GA-QQ-VSLGM-LLPA		D-LG-P----M-D----Q--
	Pseudomonas daroniae	WP_131179793	-HE--N-RH-VD-G--TYPP		Q-L--P------D-H-Y---
	Pseudomonas dryadis	WP_131176062	--D--S-QQ-VD-G--AFPL		Q-L--P------D-H-Y--E
	Pseudomonas extremaustralis	WP_010564041	-QA-TT-AQ-VSMGM-MLPA		D-LG-P------D----Q--
	Pseudomonas fildesensis	WP_048720374	-QDFTL-QQ-VSLGM-MLPA		D-LG-P----M-D----Q--
	Pseudomonas fluorescens	KAF1010123	-QDIKA-RQ-VGVG--MLPP		D-LG-P----M-D----Q--
	Pseudomonas frederiksbergensis	WP_071553456	-EQFK---QRVS-G--MLPV		S-L--P------DM-YQQ-E
	Pseudomonas fuscovaginae	WP_010445173	--DFKD-QQRVS-G--MLPV		S-L--P----M-----QQ-E
	Pseudomonas granadensis	WP_090281831	-QEFK--QQRISYG--MLPV		S-L--P----M-D----K--
	Pseudomonas grimontii	WP_090409125	-QD-KL--Q-VSLGM-LLPA		D-LG-P---CM-D----Q--
	Pseudomonas juntendi	WP_182390362	-ATVHD-QHAIS-H--MLPL		S-L--P---Q--A---VQ-E
	Pseudomonas kitaguniensis	WP_152745266	-QE-SV-QQ--STGM-LLPA		D-LG-P----M-D--Y-Q--
	Pseudomonas kuykendallii	PZP23736	--E-AS--QRVAVA--QLPA		R-L--P---Q--A-DYH---
	Pseudomonas lactis	WP_047712652	-HD-GA-QQ-VSLGM-LLPA		D-LG-P------D----Q--
	Pseudomonas leptonychotis	WP_136662948	C-Q-NS-QQHVNQG--ALPP		V-L--A--------D-HQ--
	Pseudomonas libanensis	WP_057013025	-QD-TA-QQ-VSLGM-LLPA		D-LG-P----M-D----Q--
	Pseudomonas marginalis	WP_074843988	MQD-KL--Q-VSLGM-LLPA		D-LG-P----M-D----Q--
	Pseudomonas monteilii	WP_028698128	-AA-HD-Q-EIS-N--MLPL		C-L--P---Q--A---QQ-E
	Pseudomonas mucidolens	WP_084379912	-ADINV-RQ-ISLDM-LLAP		D-LG-P----M-D----Q--
	Pseudomonas otitidis	WP_172434608	---F-H-L-EVA-G--E-PP		A-L--P----MA----C-E-
	Pseudomonas palleroniana	WP_060753835	-QDVTL-QQ-VALGM-MLPA		D-LG-P------D----Q--
	Pseudomonas plecoglossicida	WP_181107790	-AQVR--HQ-IN-S--MLPL		S-L--P---Q--T---QQ-E
	Pseudomonas protegens	WP_123473910	-EEFKQ-QQ-VGSS--TLPA		N-LG-P------D---QQ-E
	Pseudomonas punonensis	WP_073267139	MHE-EG-KH-ID-G--SVPP		K-L--PM----S--H-Q---
	Pseudomonas putida	WP_003251675	-AA-YD-Q-EIS-N--MLPL		C-L--P---Q--A---QQ-E
	Pseudomonas reactans	WP_177094888	-QEMTL-QQ-VSLGM-LLPA		D-LG-P------D----Q-E
	Pseudomonas reidholzensis	WP_119138416	-AR-QD-Q-EIN-S--MLPL		C-L--P---Q--A---QQ-E
	Pseudomonas resinovorans	WP_016494601	--DFGG-LQLVA-G--NLPP		V-L--P----M---D-H---
	Pseudomonas seleniipraecipitan	WP_092363544	-HE--G-KH-VDSG--SLPP		Q-L--P------D-D-H---
	Pseudomonas simiae	WP_128587148	-QE-IV-QQ-VSVGM-MLPA		D-LG-P----M-D----Q--
	Pseudomonas synxantha	WP_124399452	-QD-TA-QQ-VSLGM-MLPA		D-LG-P----M-D----Q--
	Pseudomonas taeanensis	WP_025164874	-GA-AS-QH-VS-G--QLPA		K-L--P-----S----Q--S
	Pseudomonas taiwanensis	WP_179060446	--DFGA-LQLVAVG--QLPP		A-L--P--------D-H---
	Pseudomonas trivialis	SDS40252	--DVAL-QQ-VSQAM-IVPM		D-LG-P----M-D----Q--
	Pseudomonas veronii	WP_169863738	-QE-NV-QQ-VSLGM-LLPT		D-LG-P------D----Q--
	Pseudomonas viridiflava	WP_122427980	-QE-TA-QQ-INLDM-LLPA		D-LG-P----M-D----Q--
	Pseudomonas yamanorum	WP_093201631	-QDFKV-QQLVSLS--MLPA		D-LG-P----M-D----Q--
Other Bacteria	*Stenotrophomonas rhizophila*	AXQ49692	-AT-QN-QG-IS-C--MLPL		S-L--P---Q--A---QQ-E

Figure-S5

Partial sequence alignments of Transglutaminase family protein showing a 1aa insertion (highlighted), which is uniquely shared by all species from the Aeruginosa clade. The presence of this CSI in *P. veroni* constitutes an isolated exception.

Group	Species	Accession	233		269
Aeruginosa Clade (Genus *Pseudomonas sensu stricto*) (14/14)	*Pseudomonas nitroreducens*	WP_038803172	NDQLRKASEYAPLIVHYN	AE	TGAAVRLKDVATVTDSV
	Pseudomonas aeruginosa	WP_053813836	-------R--Q--VI---	--	N------G---KIS---
	Pseudomonas citronellolis	WP_058070890	--------------I---	--	-N-----R---K-S---
	Pseudomonas delhiensis	WP_089392161	--------------I---	--	-N-----R---K-S---
	Pseudomonas humi	WP_069864867	--------------I---	--	-N-----R---K-S---
	Pseudomonas jinjuensis	WP_084312166	------------I-----	-A	-----------K-S---
	Pseudomonas knackmussii	WP_160287445	------------------	-Q	-----------K-S---
	Pseudomonas multiresinivorans	WP_169939381	------------------	--	---S-------K-----
	Pseudomonas nicosulfuronedens	WP_138522922	-----------S------	--	-----------------
	Pseudomonas nitritireducens	WP_184595353	------------------	--	-----------K-----
	Pseudomonas panipatensis	WP_090263844	----H---Q-----I---	--	--S--------K-S---
	Pseudomonas paraeruginosa	WP_033999185	-------R--Q--VI---	--	N------G---KIS---
	Pseudomonas pseudonitroreducens	WP_236175323	-----R------------	--	-----------------
	Pseudomonas schmalbachii	WP_208311700	-------A----I-----	--	-----------K-S---
Other *Pseudomonas*	*Pseudomonas viridiflava*	WP_122497031	----------L-V-----	--	-------G-I-E-SNA-
	Pseudomonas duriflava	WP_145143513	-SR-NR--D-R---IRTR		D-HVA--S----I----
	Pseudomonas lutea	WP_191943268	----KSPE---N--LA--		N-GPL------EIV-GA
	Pseudomonas luteola	WP_019365598	-SR-N---D-R---IRTR		N-QVA--D----I----
	Pseudomonas oleovorans	WP_119691384	S---A--AD-I-M-IR-Q		D------G-------G-
	Pseudomonas oryzihabitans	WP_059316878	----F--AD-----IR-Q		D---L--R---K-S-A-
	Pseudomonas psychrotolerans	WP_058790320	----F--AD-----IR-Q		D---L--R---K-S-A-
	Pseudomonas rhizoryzae	WP_137279207	----F--AD-----IR-Q		D--TL--R---K-S-A-
	Pseudomonas zeshuii	WP_181123566	----QS-D--KN-VIA-Q		N--P-------LAI-GA
Other Bacteria	*Atlantibacter hermannii*	HCC10005	--E-KT-A--Q---I--H		N----K-Q---------
	Cedecea davisae	WP_016537260	--E-K--A--M-------		N-S--------N-----
	Citrobacter sp. SNU WT2	WP_135321369	--E-KT----Q---I---		N------G---S-----
	Cronobacter malonaticus	WP_032980628	--E-KT-A--E-------		N----H-R---------
	Cronobacter sakazakii	PUW62476	--E-KT-A--E-------		N----H-R---------
	Cronobacter sakazakii	WP_105618278	--E-KT-A--E-------		N----H-R---------
	Cronobacter universalis	WP_038856773	--E-KT-A--E-------		N----H-R---------
	Enterobacter asburiae	WP_167819278	--E-KT----Q---I---		N------S---S-----
	EnteroBacteriaceae bacterium	WP_176919115	--E-KT-A--Q-------		N------G---------
	Escherichia coli	WP_180358716	--E-KT-A--Q---I---		N-G----G---------
	Escherichia fergusonii	WP_000667571	--E-KT-A--Q-------		N-G----G---------
	Klebsiella michiganensis	STW28513	--E-KT-A--Q-------		N------G---N-----
	Klebsiella pneumoniae	TNA34786	--E-KT-A--Q---I---		N-G----G---------
	Leclercia adecarboxylata	WP_139563225	--E-KT-A--Q---I---		N------S---S-----
	Leclercia sp. J807	WP_156239324	--E-KT-A--Q---I---		N------S---SI----
	Leclercia sp. W17	WP_114386126	--E-KT-A--Q---I---		N------S---SI----
	Leclercia sp. W6	WP_114314495	--E-KT-A--Q---I---		N------S---S-----
	Metakosakonia massiliensis	WP_044173106	--E-KT-A--Q---I---		N------S---------
	Mixta calida	WP_159378789	--E-KT-A--Q--V----		N------Q---------
	Mixta intestinalis	WP_160621567	--E-KT-A--R--V----		N------Q-----S---
	Shigella flexneri	PQN20110	--E-KT-A--Q---I---		N-G----G---------
	Shigella sonnei	WP_171556217	--E-KT-A--Q---I---		N-G----G---------
	Superficieibacter electus	WP_103674215	--E-KT-A--Q-------		N------G---------

Figure-S6

Partial sequence alignments of Multidrug efflux RND transporter permease subunit protein showing a 2aa Ins (highlighted), which is uniquely shared by all species from the Aeruginosa clade, except for its presence in *P. viridiflava*.

Group	Species	Accession	24		61
**Aeruginosa Clade (Genus *Pseudomonas sensu stricto*) (14/14)**	*Pseudomonas aeruginosa*	PXC05278	WLFTDEEFKPAPSGQQLED		NWALVRGVQRELNRRGVKL
	Pseudomonas alcaligenes	WP_187805625	-----------SNP---Q-		----IAA--Q-FK--HIE-
	Pseudomonas citronellolis	WP_082657060	--Y----------QT--D-		---------Q--E------
	Pseudomonas delhiensis	WP_089389585	--Y----------LT--D-		---------Q--E------
	Pseudomonas humi	WP_083280351	--Y----------QT--D-		---------Q--E------
	Pseudomonas jinjuensis	WP_084309528	-------------AT--D-		----I----Q--E------
	Pseudomonas knackmussii	WP_052355178	--Y---------TAT--D-		----L----K--E------
	Pseudomonas multiresinivorans	WP_169936916	------------TQ---D-		---------Q--E------
	Pseudomonas nicosulfuronedens	WP_138521990	------------TQ---D-		----V----Q--E------
	Pseudomonas nitritireducens	WP_184590989	------------TA---N-		---------Q--E------
	Pseudomonas nitroreducens	WP_017519035	------------TQ-----		---------Q--E------
	Pseudomonas paraeruginosa	WP_003092111	-------------------		-------------------
	Pseudomonas pseudonitroreducens	WP_236172804	------------TQ---D-		---------Q--E------
	Pseudomonas schmalbachii	WP_208312705	-------------AT--A-		----I----Q--E------
**Other *Pseudomonas***	*Pseudomonas migulae*	WP_182340602	---S-------SKPD--Q-		--Q--NA-RA-FE----T-
	Pseudomonas agarici	WP_017130743	--YS----N-IAN---NLQ	G	-Y---E--RQT-KAK--N-
	Pseudomonas alcaligenes	WP_021220911	---S----D-VAD-SRHLQ	E	-L--IQ--RD--AG---T-
	Pseudomonas alcaliphila	WP_074675781	--YS----D-VVD--RQLR	D	-L--I-----Q-QE-DIQ-
	Pseudomonas alkylphenolica	WP_038607093	---S------VANSD-F-Q	E	-L--I---RDT-QQ---Q-
	Pseudomonas brassicae	WP_163944778	---S------VAGAE-F-A	E	-L--I---RDT-QQQ--Q-
	Pseudomonas chengduensis	WP_017678178	--YS----D-VVD--RQLR	D	-L--I-----Q-EE-DIQ-
	Pseudomonas composti	WP_061240675	--YS----D-VAD--RQLR	D	-L--I-----Q-EE-DIQ-
	Pseudomonas donghuensis	WP_036996828	---S------VANSD-F-Q	E	-L--I---RDT-QQ---Q-
	Pseudomonas entomophila	WP_011535592	---S------TAGAE-QMQ	D	-L-----IRDA-Q-Q-TE-
	Pseudomonas fluorescens	WP_158157139	--YS----D-VAG---I-A	D	-F--IK--RDQ-KQH--Q-
	Pseudomonas fragi	NNG65069	--YS------W-DAE-HVA	D	-Y---E--R-T-EE--I--
	Pseudomonas furukawaii	WP_003448849	--YS----D-VAD-TRHLR	D	-L--IK--RD--A--D-Q-
	Pseudomonas fuscovaginae	WP_019360180	--YS------VAGQ--NLQ	D	-Y---E--RQT-KDK--N-
	Pseudomonas guariconensis	WP_090346836	---S------TAGAE--MQ	E	-L--I---RDT-Q-Q-TQ-
	Pseudomonas guguanensis	WP_090427053	--YS----A-LAD--RQMR	D	-L--I-----Q-QE-DIQ-
	Pseudomonas hydrolytica	WP_129483952	--YS----A-VADA-RQMQ	D	-L--I----QR-EQHD-H-
	Pseudomonas indica	WP_084336284	---S------VVN-E-NIA	D	-L------RDQ-A-QNI--
	Pseudomonas japonica	WP_181121837	----------VAN-D-I-K	E	-F--I---RDR-QQQ-I--
	Pseudomonas juntendi	WP_161892688	---S------TAGAEH-MQ	E	-L--I---RDT-QQH--H-
	Pseudomonas khazarica	WP_134678107	--YS----D-V-DA-RQMR	D	-L--I-----Q-EQ-DIH-
	Pseudomonas massiliensis	WP_040261260	----------VANRD-I-Q	E	-L--I---RDR-EEK-I--
	Pseudomonas mendocina	WP_147811291	--YS----D-VVD--RQLR	D	-L--I-----Q-EE-DIQ-
	Pseudomonas monteilii	WP_060478975	----------TANAE-QMQ	E	-L--IQ--RDT-RKH-SH-
	Pseudomonas mosselii	WP_138219831	---S------SVAAE-QMR	D	-L------RDT-Q-Q--Q-
	Pseudomonas oleovorans	WP_037046808	--YS----D-V-DA-RQMR	D	-L--I-----Q-EQ-DIH-
	Pseudomonas otitidis	WP_165675692	---S----D-VAD-ERHLR	D	-L--IQ--RD--DS--I--
	Pseudomonas parafulva	WP_028635242	--YS-------AGAE-RMQ	D	-L--I---RDT-QQQ-SQ-
	Pseudomonas plecoglossicida	WP_181107769	---S------TAGAD--MQ	D	-L--I---RDT-Q-Q--Q-
	Pseudomonas putida	WP_063422343	---S------TAGAE--MQ	E	-L------RDT-QQH-SQ-
	Pseudomonas reidholzensis	WP_119138617	---S------TAGAE--TE	E	-L--I---RDT-Q-Q-SQ-
	Pseudomonas resinovorans	WP_016491113	---S----D-VAD-SKQMR	D	-L--IQ--RD--A-HD-Q-
	Pseudomonas sediminis	WP_099522389	--YS----D-VVD--RQLR	D	-L--I-----Q-EE-DIQ-
	Pseudomonas sichuanensis	WP_110991672	---S------TVGA--QMQ	D	-L-----IRDS-Q-Q--Q-
	Pseudomonas soli	WP_110699978	--------N-TAGA--QMQ	D	-L------RDA-Q-Q--Q-
	Pseudomonas taiwanensis	WP_179061666	--YS----D-VAD-TRQVR	D	-L--IG--RD--A-HN-E-
	Pseudomonas toyotomiensis	WP_059390553	--YS----D-VVD--RQLR	D	-L--I-----Q-EE-DIQ-
	Pseudomonas versuta	WP_073509574	--YS------W-DAE-HVA	D	-Y---E--R-T-KE--I--
	Pseudomonas viridiflava	WP_122452127	---S------IAK-E-HQE	E	-L--IQ-AREQ-QKK----
	Pseudomonas wadenswilerensis	WP_115085387	---S------VANSD-F-Q	E	-L--I---RDT-QQ---Q-
	Pseudomonas weihenstephanensis	WP_048363226	---S------W-NA--NVA	D	-Y---E--RNT-KEQ----

Figure-S7

Partial sequence alignments of Alginate O-acetyltransferase protein showing a 1aa deletion (highlighted), which is uniquely shared by all species from the Aeruginosa clade.

Group	Species	Accession	54		98
Aeruginosa Clade (Genus *Pseudomonas sensu stricto*) (13/14)	*Pseudomonas aeruginosa*	OVZ41066	RLMRRLRFADLIFPRQWARG	P	GFIELPESQRIEVLLAELASYPVC
	Pseudomonas citronellolis	KAF1069931	-----V---E----------	S	--VA---QD--SA--E--SL----
	Pseudomonas delhiensis	WP_089393513	----H---SE----------	T	--VP---QD--GA--E--GP--L-
	Pseudomonas denitrificans	WP_151187646	----Q---PE----------	N	--TA---TD--GAI-EA--GFAQ-
	Pseudomonas humi	WP_043271865	----Q----E----------	T	--VQ---QD--GA--E--GP--L-
	Pseudomonas jinjuensis	WP_084310228	---Q----SE----------	T	-------QD--G---EQ--A----
	Pseudomonas multiresinivorans	WP_169939520	----Q---PE----------	T	--TT---QD--GAI-EA--GF-Q-
	Pseudomonas nicosulfuronedens	WP_138521661	---RQ---SE----------	N	D-TA---T---GAI-EA--AF-Q-
	Pseudomonas nitritireducens	WP_184586502	----Q---SE----------	A	--TA---TD--GAI-EA--DF-P-
	Pseudomonas nitroreducens	WP_017518618	----Q---SE----------	N	--TV---TD--GAI-EA--GF-A-
	Pseudomonas panipatensis	WP_090262294	---------E----------	G	--V----QN--G---EH-SL----
	Pseudomonas paraeruginosa	WP_003110971	--------------------	-	------------------------
	Pseudomonas pseudonitroreducens	WP_236170721	----Q---PE-I--------	S	--TA---TD--GAI-EA--GF-Q-
	Pseudomonas schmalbachii	WP_208313295	---H----------------	S	--V----Q---G---EH--P----
	Pseudomonas knackmussii	WP_043251550	-------SE----------		S-VP---QD--GA--EV--D--Q-
Other *Pseudomonas*	*Pseudomonas luteola*	WP_125889810	---K----------------	T	--VT---QD-VGAI-Q--Q-----
	Pseudomonas agarici	WP_060782782	---AG----E----------		S--D---TD--S-I--Q--TF-R-
	Pseudomonas alcaligenes	WP_184490796	----GQ---R----------		A-VA---TD--S----H--D----
	Pseudomonas asplenii	WP_102902009	----GQ---E----------		V--D---TD--S-I--HM-EF---
	Pseudomonas azotifigens	WP_181072274	---KA---S-----------		QYLA---R-------EAMSD--I-
	Pseudomonas benzenivorans	WP_090438010	----G---SQ----------		--V-----D--S----H--D----
	Pseudomonas brassicacearum	WP_123584629	----GQ---E----------		I--D---TD--S-I--H---F-T-
	Pseudomonas brassicae	WP_163949040	----E---KQ----------		S-V----TD--S---EH-------
	Pseudomonas chengduensis	WP_045735361	-M--SV--NS----------		--VM----D--S---DA--D----
	Pseudomonas chloritidismutans	WP_023446302	----K----E----------		EYLQ---TD--S----Y-------
	Pseudomonas chlororaphis	WP_045884489	----E----E----------		A-V----TD--S-I--Q---F-T-
	Pseudomonas coleopterorum	SEE09851	---SE---S-----------		D-VQ---TD--S---EQ-S-----
	Pseudomonas composti	WP_061237906	----SV--NS----------		--VM----D--S---EA--D----
	Pseudomonas deceptionensis	WP_048358472	----S----E----------		--VD---TD--S-I-EHM-DF---
	Pseudomonas endophytica	WP_055101539	---QQ----E----------		S-V----TD--S-I-EHM-DF---
	Pseudomonas entomophila	WP_011534848	---HE----Q----------		A-V----TD--S---EH--DL---
	Pseudomonas fluorescens	WP_019689563	----GQ---E----------		I--D---TD--S-I--H---F-T-
	Pseudomonas fragi	NNG61995	----G----E----------		-------TD--S-I-GHM-DF---
	Pseudomonas frederiksbergensis	WP_071550970	----GQ---E----------		I--D---TD--S-I--H---F-T-
	Pseudomonas fuscovaginae	WP_017905605	---AG----E----------		R-LD---TD--S-I-EH--E----
	Pseudomonas gingeri	WP_177084958	---AG---------------		S--D---TD--S-I--Q--TF-L-
	Pseudomonas granadensis	WP_090284772	----GQ---E----------		V--D---TD--S-I--HM-EF---
	Pseudomonas helleri	WP_048368838	---Q-----E----------		S-V----TD--S-I-EHM-DF-G-
	Pseudomonas hydrolytica	WP_129483425	-M--SV--NS----------		--VM----D--S---EA--D----
	Pseudomonas jessenii	WP_110720549	----GQ---E----------		L--D---TD--S-I--HM-EF---
	Pseudomonas koreensis	WP_077573848	----GQ---E----------		V--D---TD--S-I--HM-EF---
	Pseudomonas kribbensis	WP_114884403	----GQ---E----------		V--D---TD--S-I--HM-EF---
	Pseudomonas kunmingensis	WP_104097463	----K----E----------		DYLQ---TD--S----H--D----
	Pseudomonas laurentiana	WP_163935285	----E---SQ----------		S-V----TD--S---EA-VE----
	Pseudomonas lutea	WP_037011391	---NS---RE----------		M-VQ---TD--S-I-EHM-GFVP-
	Pseudomonas mendocina	WP_013716093	----SV--NQ----------		A-VS----D--S---EA--D----
	Pseudomonas moraviensis	WP_065615871	----GQ---E----------		F--D---ND--S-I--HM-DF---
	Pseudomonas oleovorans	KJU78420.1	-M--SV--NS----------		--VM----D--S---DA--D----
	Pseudomonas oryzihabitans	WP_145154710	---QQ-S--E-------S--		A-LS---QD--GP--EL-------
	Pseudomonas otitidis	WP_165665775	----GQ---R----------		--VA---TD--S---EQ-GA----
	Pseudomonas prosekii	WP_092270796	----GQ--------------		I--D---TD--S-I--HI-DF---
	Pseudomonas prosekii	WP_121757325	----GQ-----------S--		I--D---TD--S-I--HI-DF---
	Pseudomonas protegens	WP_110652568	---QG----E----------		-------TD--S-I--Y--GF-Q-
	Pseudomonas psychrophila	WP_019826660	---KG----E----------		--VD---TD--S-I-EHM-DF---
	Pseudomonas psychrotolerans	WP_140215710	----S----E-------S--		A-LD---QD--GP--EC--D----
	Pseudomonas putida	WP_033044623	----GQ---N----------		I--D----D--S-I--H--DF-T-
	Pseudomonas qingdaonensis	WP_100633160	----E---KQ----------		S-V----TD--S---EH--A----
	Pseudomonas resinovorans	WP_028630885	----KV--SQ----------		E-V-----D--G---EA--G----
	Pseudomonas rhizosphaerae	WP_043188043	---SA---S-----------		D-VQ---TD--S-V-EH--A----
	Pseudomonas saponiphila	WP_092316600	---QG---SQ----------		-------TD--S-I--Y--DF-Q-
	Pseudomonas saxonica	WP_122784072	----G---------------		-------TD--S-I-EHM-DF---
	Pseudomonas songnenensis	WP_122099142	----K----E----------		EYLQ---TD--S---EY--A----
	Pseudomonas stutzeri	WP_084903143	--------------------		DYLQ---TD--S----H--D----
	Pseudomonas taetrolens	WP_048382583	----G----E----------		S-V----TD--S-I-EHM-DF---
	Pseudomonas thivervalensis	WP_053120699	----GQ---E----------		T------TD--S-I--HMSAF-T-
	Pseudomonas toyotomiensis	WP_059391508	-M--SV--NS----------		--VM----D--S---DA--D----
	Pseudomonas umsongensis	WP_179054015	----GQ---N----------		I--D----D--S-I--H--DF-T-
	Pseudomonas versuta	WP_060691452	----G---VE----------		-------TD--S-I-QHM-DF---
	Pseudomonas viridiflava	WP_122607495	----GQ---E----------		V--D---TD--S-I--HM-DF---
	Pseudomonas vranovensis	WP_123565432	Q---E----Q----------		A-------D--S---EA--D--Q-
	Pseudomonas xanthomarina	WP_041014118	----K----E----------		EYLQ---TD--S---EY-------
Other Bacteria	*Thalassospira xiamenensis*	PTC01002	---GA---SE----------		-YV-----D--S----Q--EL-F-
	Azotobacter beijerinckii	SEI65163	----G----G----------		D-V-----E--G---ER--D----
	Azotobacter chroococcum	WP_131348765	---HG----G----------		--V-----E--G---ER--D--I-
	Azotobacter salinestris	WP_152389355	----E----A----------		--V-----D--G---EH--D----
	Azotobacter vinelandii	WP_012700621	----E----G-V--------		A-V-----E--G---EH--A----

Figure-S8

Partial sequence alignments of 23S rRNA (cytidine(2498)-2'-O)-methyltransferase RlmM protein showing a 1aa insertion (highlighted), which is uniquely shared by all species from the Aeruginosa clade except *P. knackmussii*. One outgroup species *P. luteola* also shares this CSI.

Group	Species	Accession	6		52
**Alcaligenes Clade (*Aquipseudomonas* gen. nov.) (4/4)**	*Pseudomonas alcaligenes*	WP_110680887	ELRKAGLKVTLPRVKILQMLDSA	TS	GQRHMSAEDVYKALMEAGEDVG
	Pseudomonas campi	WP_173209422	-----------------------	--	----------------------
	Pseudomonas guryensis	WP_173209422	-----------------------	--	----------------------
	Pseudomonas ullengensis	WP_183087075	-----------------------	S-	----------------------
**Other *Pseudomonas***	*Pseudomonas aeruginosa*	WP_205579342	-----------------------		E---------------------
	Pseudomonas akappageensis	WP_166364532	----V-----------------T		E---------------------
	Pseudomonas alkylphenolica	WP_038606723	----------------------T		E---------------------
	Pseudomonas anguilliseptica	WP_233686430	-----------------------		------------S-----D---
	Pseudomonas asiatica	WP_137188255	----------------------T		E---------------------
	Pseudomonas asuensis	WP_188868248	----V------------------		----------------------
	Pseudomonas azotifigens	WP_028241626	---------------------T-		E---------------------
	Pseudomonas baetica	WP_221730599	----------------------T		E---------------------
	Pseudomonas bananamidigenes	WP_065259963	----------------------T		E---------------------
	Pseudomonas batumici	WP_040064097	----------------------T		E---------------------
	Pseudomonas borbori	WP_090502261	-----------------------		E---------------------
	Pseudomonas brassicacearum	WP_230150441	-----------------------		E---------------------
	Pseudomonas capeferrum	WP_181130602	----------------------N		Q---------------------
	Pseudomonas carbonaria	WP_187669746	----------------------S		----------------------
	Pseudomonas cavernicola	WP_119956211	-----------------------		E---------------------
	Pseudomonas chlororaphis	WP_123337541	----------------------T		E---------------------
	Pseudomonas corrugata	WP_055135535	-----------------------		E---------------------
	Pseudomonas delhiensis	KAF1056945	-----------------------		E---------------------
	Pseudomonas duriflava	WP_145144653	-----------------------		E---------------------
	Pseudomonas endophytica	WP_055101465	-----------------------		E--------------Q------
	Pseudomonas entomophila	WP_060392466	---------------------LN		E---------------------
	Pseudomonas fluorescens	WP_150641005	-----------------------		E---------------------
	Pseudomonas fluvialis	WP_184682476	-----------------------		-L--------------------
	Pseudomonas fragi	NNG61403	-----------------------		E--------------Q------
	Pseudomonas frederiksbergensis	MCE6981992	----------------------T		E---------------------
	Pseudomonas fulva	WP_196178000	-----------------------		E---------------------
	Pseudomonas fuscovaginae	WP_026145177	----------------------T		E---------------------
	Pseudomonas gingeri	WP_177090314	-----------------------		E---------------------
	Pseudomonas gozinkensis	WP_192563468	----------------------T		E---------------------
	Pseudomonas guariconensis	WP_196155450	----------------------T		E---------------------
	Pseudomonas indica	WP_084337192	-----------------------		D---------------------
	Pseudomonas japonica	WP_181117716	-----------------------		E---------------------
	Pseudomonas jinjuensis	WP_084312609	----------------------T		E---------------------
	Pseudomonas juntendi	WP_236166407	-----------------------		E---------------------
	Pseudomonas knackmussii	WP_043255346	-----------------------		E---------------------
	Pseudomonas kuykendallii	WP_090223643	----V-----------------T		D---------------------
	Pseudomonas lalucatii	WP_213639351	-----------------------		E---------------------
	Pseudomonas leptonychotis	WP_136665255	-----------------------		------------------D---
	Pseudomonas lundensis	WP_047278445	-----------------------		D--------------Q------
	Pseudomonas mangiferae	WP_143488868	----------------------T		E--------------D------
	Pseudomonas mangrovi	WP_108107395	-----------------------		E---------------------
	Pseudomonas marincola	WP_150548514	----V------------------		------------S---------
	Pseudomonas massiliensis	WP_040259344	-----------------------		E---------------------
	Pseudomonas mosselii	WP_138221484	-----------------------		E---------------------
	Pseudomonas parafulva	WP_116887550	-----------------------		E---------------------
	Pseudomonas protegens	MBB5601605	----------------------T		E---------------------
	Pseudomonas psychrophila	WP_026014202	-----------------------		E--------------Q------
	Pseudomonas putida	WP_241803859	----------------------T		E---------------------
	Pseudomonas reidholzensis	WP_119137930	----------------------T		E---------------------
	Pseudomonas resinovorans	WP_016494790	----------------------T		E---------------------
	Pseudomonas saudiphocaensis	WP_037026398	---------------------TT		E---------------------
	Pseudomonas schmalbachii	WP_208314817	----------------------T		E---------------------
	Pseudomonas segetis	WP_089360435	----V------------------		------------S---------
	Pseudomonas stutzeri	WP_025240578	---------------------TT		D---------------------
	Pseudomonas syringae	WP_236480361	----------------------T		E---------------------
	Pseudomonas tohonis	BCG27100	----------------------T		E---------------------
	Pseudomonas typographi	WP_190423954	-----------------------		D-----------S---------
	Pseudomonas urmiensis	WP_186556509	----------------------T		E---------------------
	Pseudomonas urumqiensis	WP_120995936	---------------------TT		E---------------------
	Pseudomonas vanderleydeniana	WP_186682995	----------------------T		E---------------------
	Pseudomonas viciae	WP_135843635	-----------------------		E---------------------
	Pseudomonas viridiflava	WP_122454032	-----------------------		E---------------------
	Pseudomonas weihenstephanensis	WP_203307648	----------------------T		E--------------Q------
**Other Bacteria**	*Acinetobacter baumannii*	SCY95734	---------------------S-		E-----------A---A-----
	Azotobacter chroococcum	WP_039802036	-----------------------		-H----------S---------
	Atopomonas hussainii	WP_071873046	---------------------Q-		D-----------S---------
	Entomomonas moraniae	WP_109704092	------------------L--A-		ES----------S---S-----
	Escherichia coli	MRF42614	----------------------T		E-----------A---------
	Halopseudomonas pertucinogena	WP_188637200	--------------------AND		DD----------A---------
	Moraxella catarrhalis	WP_152698349	-------------I---E--EN-		EHH--------RA-A-Q-----
	Azomonas agilis	WP_144570072	-------------I---------		-H-------------D------
	Azotobacter	WP_012702790	-----------------------		-H--------------------
	Halopseudomonas salina	WP_150278796	----------------------S		PE--L-----------------
	Rhizobium laguerreae	WP_163883734	-----------------------		E---------------------

Figure-S9

Partial sequence alignments of Ferric iron uptake transcriptional regulator protein showing a 2aa insertion (highlighted), which is uniquely shared by all species from the Alcaligenes clade.

Group	Species	Accession	55		93
**Alcaligenes Clade (*Aquipseudomonas* gen. nov.) (4/4)**	*Pseudomonas alcaligenes*	WP_061903990	DWMLDQEAEPSALLAYLAGGS		SRLGRYYERLWQFALQAA
	Pseudomonas ullengensis	WP_183088600	--LHH-QRDSQ--QQF--A--		---------------EQ-
	Pseudomonas campi	WP_173207698	--L-GL-RDSHV-QQF--T--		------------------
	Pseudomonas guryensis	MBB1519443	--L-SL-RDSQ---QF-SS--		------------------
**Other *Pseudomonas***	*Pseudomonas aeruginosa*	PBD22594	A-LRE--RQ-QH-A-W--QWP	R	Q---L---H------EQ-
	Pseudomonas alcaliphila	WP_074681476	--LVQ-DN-AG--H-W--AKP	V	R---L----------H--
	Pseudomonas chengduensis	WP_021489728	--LVQ-DNDAG--H-W--AKP	V	R---L----------H--
	Pseudomonas citronellolis	WP_116425718	--LRE--RHSE--Q-W-QSSP	Q	Q---------------Q-
	Pseudomonas composti	WP_037000078	--LAQ-DSDA--VH-W--SKP	V	R---L----------H--
	Pseudomonas daroniae	WP_131192123	--L-R-DK--AS-Q-W-SRS-	V	R---L-------------
	Pseudomonas flexibilis	WP_039606392	--L-RLDEQ-AV--EW--R-P	L	R---L-------------
	Pseudomonas guguanensis	WP_090432858	--LMQ--RDA---Y-W--ERP	V	R---L----------H--
	Pseudomonas indoloxydans	WP_108233270	--LVQ-DNDAD--H-W--AKP	V	R---L----------H--
	Pseudomonas japonica	WP_042124165	H-L-CL-Q--AP--DW--RSA	S	R---L-----------H-
	Pseudomonas knackmussii	WP_160287759	--LRQ-DRA-E--VNQ-QAAP	Q	Q--------------EQ-
	Pseudomonas marincola	CAE6928405	--L-QLDQQ-D--RHW--ER-	V	R---L----------N--
	Pseudomonas mendocina	WP_209450995	--LAQ-DRD----HSW--EKP	V	H---L----------H--
	Pseudomonas nitritireducens	WP_193452171	--LRS--HS-GE---Q-EAAP	H	Q--------------EH-
	Pseudomonas nitroreducens	WP_024765112	--LRS--HS-GE---Q-EAAP	H	Q--------------ER-
	Pseudomonas oleovorans	RED06498	--LRQ-D-A-A--ATW--Q--	D	R----Q---------ER-
	Pseudomonas oryzihabitans	WP_144959216	--LRQ-D-A----ATW--Q--	D	R----Q---------ER-
	Pseudomonas panipatensis	WP_090260444	--L-AL-QA-EG-R-W-QAAP	Q	Q--------------GQ-
	Pseudomonas psychrotolerans	ONN70728	--LRQ-D-A-A--ATW--Q--	D	R----Q---------ER-
	Pseudomonas rhizoryzae	WP_240043595	--LRQ-D-A-EE-A-W--Q--	D	R----Q---------AR-
	Pseudomonas sagittaria	WP_092432758	--LQALDR--QP---Q-PAV-	G	N-------E------RQ-
	Pseudomonas schmalbachii	WP_208314642	--LQE-DRQ-GE-A-W-DAEP	R	Q--------------EQ-
	Pseudomonas sediminis	WP_099523627	--LAQ-DKD--P-HTW--AKP	V	R---L----------R--
	Pseudomonas seleniipraecipitan	WP_092364912	--L-R-DQ--AP---W-SRS-	I	R---L----------H--
	Pseudomonas stutzeri	WP_103457901	--L-R-D----I-H-W-SLH-	T	R---L-------Y--SQ-
	Pseudomonas toyotomiensis	WP_059391431	--LVQ-DNDAG--H-W--AKP	V	R---L----------H--
	Pseudomonas tructae	WP_130265609	Q-L-E-DRNSH---HW--QA-	T	R---L-----------H-
	Pseudomonas typographi	WP_190540851	A-LHTLD--------F--QR-	V	R---L---------VA--
	Pseudomonas vranovensis	WP_123567300	H-L-E-DRDSQ---HW--QA-	T	R---L--------V--H-
	Pseudomonas wadenswilerensis	WP_115085999	Q-L-E-DRDSH---HW--QA-	T	R---L--------V--H-
	Pseudomonas yangonensis	WP_161866942	--LAQ-DRD--P-HSW--EKP	V	R---L----------H--
**Other Bacteria**	*Azomonas macrocytogenes*	WP_183167635	--L-H--DI-QN-QGW--R--	S	R---I---Q------HQ-
	Azotobacter beijerinckii	WP_090942171	--LRALD-CSAP-D-W--QHP	T	R---L----------NQ-
	Azotobacter salinestris	WP_152389217	--LQALD-CSTP-D-W--QHP	T	R---L----------HQ-
	Azotobacter vinelandii	WP_012701938	--LRTLD-CSAP-D-W--RHP	T	R---I----------EQ-

Figure-S10

Partial sequence alignments of DUF1853 family protein showing a 1aa CSI (highlighted), which is uniquely shared by all species from the Alcaligenes clade.

Group	Species	Accession	55		98
**Alcaligenes Clade (*Aquipseudomonas* gen. nov.) (4/4)**	*Pseudomonas alcaligenes*	WP_076424264	GLQLASHWEGADCVLSAPASSLL		KLALAKDKTAILHRPEVSLSG
	Pseudomonas campi	WP_173209964	------Q-S----T-R---A---		R--------SV------E-D-
	Pseudomonas ullengensis	WP_183087378	--------DA---R-R-------		R-----------Q---A-D-
	Pseudomonas guryensis	WP_182832255	------Q-S----T-R---A---		R--------SV------E-E-
**Other *Pseudomonas***	*Pseudomonas alcaliphila*	WP_075745059	-----AQ-HAPADCTLTAPAAS-	L	R---SRE-----------D-
	Pseudomonas arsenicoxydans	WP_208670450	--M--A---TGADCTLRAPA-S-	L	---MS-----V--G---E-D-
	Pseudomonas azerbaijanoriens	WP_126048766	--M---Q--TGADCTLRAPA-S-	L	---TS----SV--A---E-D-
	Pseudomonas brassicacearum	WP_123435485	--M------TGADCTLRAPA-S-	L	---MS----SV--G---E-D-
	Pseudomonas chengduensis	WP_090336050	-----AQ-HAPADCTLTAPAAS-	L	R---SRE---V-------D-
	Pseudomonas composti	WP_061238453	------Q-HAPADCTLTAPAAS-	A	R---SR----V-------D-
	Pseudomonas corrugata	WP_152979042	--M--T---AEADCTLRAPA-S-	L	N---S-----V--S-D-E-E-
	Pseudomonas farris	WP_217855378	--M---Q--TGADCTLRAPA-S-	L	---TS----SV--A---E-D-
	Pseudomonas fluorescens	WP_123585573	--M------TGADCTLRAPA-S-	V	----S-----V--A---E-D-
	Pseudomonas frederiksbergensis	WP_076028949	--M---Q--TGADCTLRAPA-S-	L	---MS----S---A---E-D-
	Pseudomonas germanica	WP_220557392	--M------TGVDCTLRAPA-S-	I	----S-----V--A---E-D-
	Pseudomonas gregormendelii	WP_205893443	--M---Q--AGADCTLRAPA-S-	L	---MS-----V--G---E-D-
	Pseudomonas indoloxydans	WP_108234243	------Q-HAPTDCTLTAPA-S-	A	R---SRE-----------D-
	Pseudomonas izuensis	WP_160105299	--M---Q--TGADCTLRAPA-S-	L	---MS----SV--A-Q-E-D-
	Pseudomonas khorasanensis	WP_186531923	--M------TGVDCTLRAPA-S-	L	----S-----V--A---E-D-
	Pseudomonas kilonensis	WP_156331335	--M--A---AEADCTLRAPA-S-	L	N---G-----V----D-E-D-
	Pseudomonas koreensis	WP_127648616	--M------TGVDCTLRAPA-S-	V	---VS-----V--A---E-D-
	Pseudomonas laurylsulfativoran	WP_103397262	--M---Q--TGADCTLRAPA-S-	L	---MS----S---S---E-D-
	Pseudomonas lini	WP_048396068	--M---Q--SGADCTLRAPA-S-	L	---TS----SV--A---E-D-
	Pseudomonas mandelii	WP_019581841	--M------TGADCTLRAPA-S-	L	---TS----SV--G---E-D-
	Pseudomonas mediterranea	WP_055127135	--M------AEADCTLRAPA-S-	L	N---S-----V--S-D-E-E-
	Pseudomonas mendocina	WP_106737437	------Q-HAPADCTLTAPAAS-	A	R---SR----V-------D-
	Pseudomonas migulae	WP_084322541	--M------TGADCTLRAPA-S-	L	---MS----SV--E-Q-E-D-
	Pseudomonas moorei	WP_090328177	--M---Q--TGADCTLRAPA-S-	L	---MS----SV--G---E-E-
	Pseudomonas neuropathica	WP_194935811	--M--A---TGVDCTLRAPA-S-	I	---VS-----V--A---E-D-
	Pseudomonas oleovorans	WP_119692141	-----AQ-HADADCTLNAPA-S-	A	R---SR----V-------D-
	Pseudomonas putida	WP_225137175	--M------TGVDCTLRAPA-S-	V	----S-----V--A---E-D-
	Pseudomonas reinekei	WP_075947218	--M---Q--TGADCTLRAPA-S-	L	---MS-----V--A---E-D-
	Pseudomonas syringae	WP_236484765	--M---N--TGADCTLRAPAAS-	M	----S----GV--S---E-D-
	Pseudomonas toyotomiensis	WP_059390783	-----AQ-HAPADCTLTAPAAS-	L	R---SRE---V-------D-
	Pseudomonas vancouverensis	WP_093227511	--M---Q--TGADCTLRAPA-S-	L	----S--------G---E-D-
	Pseudomonas viridiflava	WP_122609125	--M------T-VDCTLHAPA-S-	I	---VS-----V--A---E-D-
	Pseudomonas yangonensis	WP_178101938	-----AQ-HAPADCTLTAPAAS-	A	R---SRE---V-------D-
	Pseudomonas zeae	WP_186621320	--M-----GTGVDCTLRAPA-S-	I	---VS-----V--A---E-D-

Figure-S11

Partial sequence alignments of SCP2 sterol-binding domain-containing protein showing a 1aa CSI (highlighted), which is uniquely shared by all species from the Alcaligenes clade.

Group	Species	Accession	125		167
Alcaligenes Clade (*Aquipseudomonas* gen. nov.) (3/3)	*Pseudomonas alcaligenes*	GIZ66354	EKAAWMLAQRRDAEGQPLLLSNE		GGNLSYAVEFGKITARLDLL
	Pseudomonas guryensis	WP_182831756	-------G----SR-R-------		-----F------MV------
	Pseudomonas campi	WP_173203855	-------G----SR-R-------		-----F------MV------
Other *Pseudomonas*	*Pseudomonas alcaliphila*	SDD63782	---G---S-----T---------	FSEQ	-N-I--------MV------
	Pseudomonas allokribbensis	WP_192558471	---T-I----QNK------F---	ISEE	-S---F-T----VV------
	Pseudomonas anatoliensis	WP_210699700	---T-I-S--QNK------F---	ISEE	-S---F-------V------
	Pseudomonas anguilliseptica	SEC24656	---S------T--G-K-W-----	ISAK	-H-----------V------
	Pseudomonas argentinensis	SFI57875	---T-L--T----Q-N-------	LSEN	-R---F-------V----Y-
	Pseudomonas arsenicoxydans	WP_090188821	---T-I-S--QNKN-----F---	ISEE	-S---F-------V------
	Pseudomonas azerbaijanoriens	WP_217835818	-I-T-I-S--Q-K------F---	ISEE	-S---F-------V------
	Pseudomonas baetica	WP_221730480	---T-I-S--QNK------F---	ISEE	-S---F-T-----V------
	Pseudomonas bananamidigenes	WP_065259452	---T-I----QNK------F---	ISEE	-S---F-T----VV------
	Pseudomonas botevensis	WP_217831693	---T-I-S--QNK------F---	ISEE	-S---F-T----VV------
	Pseudomonas chengduensis	WP_026088690	---G---S-----A-R-------	FSEQ	-N----------MV------
	Pseudomonas chlororaphis	MBP5144255	---T---S--QG-N-EL--F---	ISEE	-S---F-------V------
	Pseudomonas composti	EZH83543	---G---S-----M-R-------	FTEQ	-N----------MV------
	Pseudomonas daroniae	WP_242674772	---T----T----Q-N-------	ISEQ	-R---F-------V----Y-
	Pseudomonas dryadis	WP_131176908	-R-T-L--S----Q-R-------	ISDE	-R---F-------V----Y-
	Pseudomonas ekonensis	WP_217891340	---T-I----QNK------F---	ISEE	-S---F-T-----V------
	Pseudomonas flavescens	NYH74542	---T-L--T----Q-N-------	LSEQ	-R---F-------V----Y-
	Pseudomonas fluorescens	WP_033046551	---T-L-S--Q-K--K---F---	ISEE	-S---F-------V------
	Pseudomonas fulva	KIP90503	---T-L--T----Q-N-------	LSEQ	-R---F-------V----Y-
	Pseudomonas glycinae	WP_197867363	---T-I----QNK------F---	ISEE	-S---F-T-----V------
	Pseudomonas gozinkensis	WP_192561698	---T-I----QNK------F---	ISEE	-S---F-T----VV------
	Pseudomonas guguanensis	WP_090426896	-------G-----S---------	VSEQ	-S----------MV------
	Pseudomonas guineae	SFI91076	---S------T--G-T-W-----	ISAE	-R-----------V------
	Pseudomonas indoloxydans	WP_108234460	---G---S-----T-R-------	FSEQ	-N----------MA------
	Pseudomonas jessenii	SEC66593	-I-T-I-S--QNK------F---	ISEE	-S---F-------V------
	Pseudomonas khazarica	WP_037047410	-------SN----A-R-------	ISEQ	-S----------MV------
	Pseudomonas koreensis	WP_127652184	---T-I-S--QNK------F---	ISEE	-S---F-T-----V------
	Pseudomonas kribbensis	WP_114886742	---T-I----QNK------F---	ISEE	-S---F-T----VV------
	Pseudomonas laurylsulfatiphila	WP_104448080	-I-T-I-S--Q-K------F---	ISEE	-S---F-------V------
	Pseudomonas leptonychotis	TIH07801	---S------T--G-K-W-----	ISAE	-R-----------V------
	Pseudomonas mangrovi	WP_108104628	-------RN-T--L-R-----D-	ISAQ	-Q----------M-------
	Pseudomonas mendocina	ARS50348	-------G-----S---------	VSEQ	-S----------MV------
	Pseudomonas moraviensis	NYH10980	---T-I----QNK------F---	ISEE	-S---F-T----VV------
	Pseudomonas neuropathica	WP_194935577	---T-I-S--QNK------F---	ISEE	-S---F-T-----V-----I
	Pseudomonas oleovorans	MBN7118739	---G---S-----T-R-------	FSEQ	-N----------MV------
	Pseudomonas peli	SCW82069	---S------T--G-K-W-----	ISAE	-H-----------V------
	Pseudomonas pohangensis	WP_231975052	---S----N-K--Q---W-----	ISEH	-S------Q----V------
	Pseudomonas punonensis	WP_083593674	---T-L--T----Q-N-------	LSEN	-R---F-------V----Y-
	Pseudomonas seleniipraecipitan	SDE83238	---T-L--T----Q-N-------	ISEQ	-R---F-------V----Y-
	Pseudomonas sihuiensis	WP_231976562	---G---S-----T---M-----	FSEQ	-N----------MV------
	Pseudomonas syringae	WP_236485548	---T-I-S--QNK------F---	ISEE	-S---F-T-----V-----I
	Pseudomonas toyotomiensis	WP_072423743	---G---S-----T-R-------	FSEQ	-N----------MV------
	Pseudomonas vancouverensis	WP_093224488	-I-T-I----QTS------F---	ISEE	-S---F-------V------
	Pseudomonas viciae	WP_238346750	---T-L-S--QNKA-----F---	ISEE	-S---F-------V------
	Pseudomonas viridiflava	WP_122607425	---T-I-S--QNK------F---	ISEE	-S---F-T-----V-----I
	Pseudomonas wenzhouensis	UFQ99508	---S---S-----T-R-------	FSEQ	-N-I--------MV------
	Pseudomonas xionganensis	WP_233280134	--------N---EQ-N-W-----	MSEQ	-N-----------V------
	Pseudomonas yangonensis	WP_161867361	---G---S-----T---------	FTEQ	-A----------MV------
	Pseudomonas zeae	WP_186624135	---T-I-S--QNK------F---	ISEE	-S---F-T-----V-----I
Other Bacteria	*Azomonas agilis*	WP_144570681	-----L-S--K-VQ---------	ISED	-R---F-------V-----M

Figure-S12

Partial sequence alignments of a hypothetical protein showing a 4aa CSI (highlighted), which is uniquely shared by all species from the Alcaligenes clade. No homolog for this protein was found for the species *P. ullengensis*.

Group	Species	Accession	261		297
Alcaligenes Clade (*Aquipseudomonas* gen. nov.) (4/4)	*Pseudomonas alcaligenes*	WP_061902889	TLSAGLPVRLAELDALG		VEARSYEQLLDALAGQLAG
	Pseudomonas ullengensis	MBB2494185	---D----K--------		AQ-Q-------N-------
	Pseudomonas campi	WP_240008789	--------K---M----		A--Q------ED--NE--D
	Pseudomonas guryensis	WP_182832666	--------K---M----		AS-Q------EG--SE---
Other *Pseudomonas*	*Pseudomonas akappageensis*	WP_218584046	--T--------------		SN-Q------EN-GEG---
	Pseudomonas aeruginosa	AOX30251	---E----------D--	VNVS	-D-NG--N--NN---EF--
	Pseudomonas azotifigens	WP_028241074	--TR-------------	VGVE	TD--G--R---N--A----
	Pseudomonas capeferrum	KEY86483	--T--------------	GTDP	-DGKG--R--EK-G-D---
	Pseudomonas cavernicola	WP_238474206	--T--------------	GSLP	-N--G------N-GNG--D
	Pseudomonas coronafaciens	KGS10829	--------K-----G--	GYTP	AT-QG---V-EK--ND---
	Pseudomonas cremoricolorata	WP_028695806	--T--------------	GTDP	-D-TG--R---K-GSD---
	Pseudomonas entomophila	WP_240064120	--T--------------	GTDP	-D--G--R--EK-GSD-T-
	Pseudomonas fakonensis	WP_217841148	--T--------------	GTDP	-DGKG--R--ER-G-D---
	Pseudomonas fluorescens	VVN02795	--T--------------	GSEP	-DSKG--R--EN--NG---
	Pseudomonas fulva	WP_236166839	--T--------------	GTDP	-D-QG--R---K-G-D--S
	Pseudomonas guariconensis	MBF8732359	--T--------------	GNDP	-D-KG--R--EK-GNE-T-
	Pseudomonas huaxiensis	WP_110969913	--T-----K--------	GTLP	-T-QG------N-GNG---
	Pseudomonas indica	WP_084338549	--------T------M-	SGVA	-D-GG--R---N---E---
	Pseudomonas juntendi	WP_235768307	--T--------------	GTDP	-DGKG--R--EK-G-D---
	Pseudomonas monteilii	BBV94724	--T--------------	GTDP	-DGKG--R--EK-G-D---
	Pseudomonas mosselii	WP_096049016	--T--------------	GSDA	-D--G--R--EK-GND-T-
	Pseudomonas muyukensis	WP_217851472	--T--------------	GSDA	-DGKG--R--EK-G-D---
	Pseudomonas peradeniyensis	WP_186732112	--T--------------	GSDA	-D--G--R--EK-GSD-T-
	Pseudomonas plecoglossicida	EPB94603	--T--------------	GTDS	-D-KG--R--EK-G-D---
	Pseudomonas putida	KHL71713	--T--------------	GNEP	-D-KG--R--EK-G-E---
	Pseudomonas shirazica	WP_139657931	--T--------------	GTDP	-DGKG--H--EK-G-D-V-
	Pseudomonas sichuanensis	WP_110995016	--T--------------	GTDP	-D-KG--R--EK-G-D---
	Pseudomonas soli	SEQ55197	--T--------------	GGDK	-DDKG--R--EK-G-D---
	Pseudomonas syringae	WP_019717095	--------K--------	SYTP	AT-QG---V-EK--ND-T-
	Pseudomonas taiwanensis	ESW40671	--T--------------	GTDP	-DGKG--R--EK-G-D-V-
	Pseudomonas viridiflava	WP_122850137	--T-----K--------	ADIP	GT-TG-----N--GND---
	Pseudomonas xanthosomae	WP_217886879	--T--------------	GTDP	-DGKG--R--EK-G-D---
Other Bacteria	*Azomonas agilis*	WP_144570875	S-TQ----T--------	GSIP	-T-QG-----EQI-QS-SR
	Azomonas macrocytogenes	WP_183165952	----N---TM--I-PM-	TGIE	PH-QG--E--ER-TE--S-
	Azotobacter chroococcum	P_089166694	--------------P--	SAVP	-GPQG-----EG-GEG---
	Azotobacter salinestris	WP_152387870	--------------P--	SGVP	-GPQG-----ER-GEG---
	Azotobacter vinelandii	WP_012698778	--------------P--	SGIP	-GPQG--R--EN--EG---
	Escherichia coli	MQK16861	--------T--------		AS-K-------E-------

Figure-S13

Partial sequence alignments of Zinc ABC transporter substrate-binding protein showing a 4 aa CSI (highlighted), which is uniquely shared by all species from the Alcaligenes clade.

Group	Species	Accession	130–170		
**Alcaligenes Clade (*Aquipseudomonas* gen. nov.) (4/4)**	*Pseudomonas alcaligenes*	WP_203791762	PYIALQLKAIVLGVNLLVGT		TPSPTRSQDTALIVSLVLALF
	Pseudomonas guryensis	WP_182833254	--------G--------I-A		------G---------I----
	Pseudomonas campi	WP_173208052	--------G--------I-A		------G---------I----
	Pseudomonas ullengensis	WP_183089287	--------G--------I-A		------A--------------
**Other *Pseudomonas***	*Pseudomonas dryadis*	WP_131173937	--------G--------I-S		DSGG--A-----V---I----
	Pseudomonas alcaligenes	MBB4817296	--------G--------I-S	GA	ESTG--A--------------
	Pseudomonas antarctica	WP_083357464	--------G--------I-S	GP	DTTG--A--------------
	Pseudomonas argentinensis	WP_070886659	--------G--------I-S	ET	D-AG--A--------VI----
	Pseudomonas borbori	WP_090500359	--------G--------I-A	GP	DS-GI-A---------I----
	Pseudomonas brassicacearum	WP_123426855	--------G--------I-A	GP	DAMG--A---------I----
	Pseudomonas carnis	WP_237495031	--------G--------I-A	GP	DTTG--A--------------
	Pseudomonas cavernicola	WP_119955708	--------G--------I-A	GA	ESTG--A--------------
	Pseudomonas costantinii	WP_177015073	--------G--------I-S	GP	DTTG--A--------------
	Pseudomonas cremoris	WP_185703608	--------G--------I-S	GP	DTTG--A--------------
	Pseudomonas daroniae	WP_131190891	--------G--------I-S	ET	DADG--A--------VI----
	Pseudomonas edaphica	WP_177034579	--------G--------I-S	GP	DTTG--A--------------
	Pseudomonas fluorescens	WP_034117115	--------G--------I-A	GP	DTTG-----------------
	Pseudomonas frederiksbergensis	MCE6982545	--------G--------I-A	GA	DATGS-V-----V--------
	Pseudomonas haemolytica	WP_153871088	--------G--------I-A	GP	DTTG-----------------
	Pseudomonas karstica	WP_154744056	--------G--------I-A	GP	DTTG--A--------------
	Pseudomonas khavaziana	WP_217881745	--------G--------I-A	GP	DTTG--A--------------
	Pseudomonas lactis	WP_047711511	--------G--------I-A	GP	DTTG--A--------------
	Pseudomonas lalkuanensis	WP_151132154	--------G--------I-S	GA	ESAG--A--------------
	Pseudomonas libanensis	WP_059397773	--------G--------I-A	GP	DTTG--A--------------
	Pseudomonas marginalis	WP_122249539	--------G--------I-S	GP	DTTG--A--------------
	Pseudomonas marincola	WP_090510746	--------G--------I-S	GA	GATG--A--------------
	Pseudomonas moraviensis	WP_197892829	--------G--------I-A	GP	DAMG--A--------------
	Pseudomonas multiresinivorans	WP_169937419	--------G--------I-A	GP	DSTGI-A---------I----
	Pseudomonas nabeulensis	WP_135310074	--------G--------I-S	GP	DTTG--A--------------
	Pseudomonas nicosulfuronedens	WP_138526337	--------G--------I-A	GP	DSTGI-A---------I----
	Pseudomonas nitroreducens	WP_017522061	--------G--------I-A	GP	DSTGI-A--------------
	Pseudomonas orientalis	WP_124433551	--------G--------I-S	GP	DTTG--A-----V--------
	Pseudomonas otitidis	WP_165666853	--------G----------S	GA	DSTG--A--------------
	Pseudomonas palleroniana	WP_090369489	--------G--------I-S	GP	DTTG--A-----V--------
	Pseudomonas panacis	WP_154841532	--------G--------I-A	GT	DATG--A--------------
	Pseudomonas panipatensis	WP_090262756	--------G--------I-A	GP	DSGGS-A---------I----
	Pseudomonas paralactis	WP_057700935	--------G--------I-A	GP	DTTG--A--------------
	Pseudomonas poae	WP_123715555	--------G--------I-S	GP	DTTG--A-----V--------
	Pseudomonas prosekii	WP_092271241	--------G--------I-A	GP	DAMG--A--------------
	Pseudomonas punonensis	WP_073264186	--------G--------I-S	ET	D-DG--A--------VI----
	Pseudomonas putida	WP_065862199	--------G--------I-A	TP	DATG--V-----V--------
	Pseudomonas resinovorans	WP_016491184	--------G--------I-S	GA	ESAG--A--------------
	Pseudomonas salmasensis	WP_186606866	--------G--------I-A	GP	DTTG--A--------------
	Pseudomonas salomonii	WP_236370781	--------G--------I-S	GP	DTTG--A--------------
	Pseudomonas schmalbachii	WP_208315438	--------G--------I-A	GP	DSTG--------L---I----
	Pseudomonas segetis	WP_089358742	--------G----------S	VP	DSTG--A--------------
	Pseudomonas seleniipraecipitan	WP_070881785	--------G--------I-S	ET	D-DG--A-----V---I----
	Pseudomonas simiae	OAE11314	--------G--------I-S	GP	DTTG--A--------------
	Pseudomonas synxantha	WP_124378902	--------G--------I-A	GP	DTTG--A--------------
	Pseudomonas taiwanensis	WP_179061606	--------G--------I-P	SA	EA-G--A--------------
	Pseudomonas tohonis	WP_173171953	--------G--------I-S	GA	ESTG--A--------------
	Pseudomonas tolaasii	WP_017256311	--------G--------I-A	GV	DT-G--A--------------
	Pseudomonas viridiflava	WP_122717859	--------G--------I-S	GP	DTTG--A--------------
	Pseudomonas wayambapalatensis	MBC3426361	--------G--------I-A	TP	DATG--V-----V--------
	Pseudomonas xionganensis	WP_160344636	--------G----------A	GA	ESAG--A---------I----
	Pseudomonas yamanorum	WP_177044792	--------G--------I-A	SA	DTTG-----------------
**Other Bacteria**	*Acinetobacter pittii*	WP_163114574	--------G--------I-S	GA	DSTG--A--------------
	Escherichia coli	WP_214292197	--------G--------I-S	GA	DSTG--A--------------
	Xanthomonas citri pv. citri	MBD4392512	--------G--------I-S	GA	DSTG--A--------------

Figure-S14

Partial sequence alignments of Hybrid sensor histidine kinase/response regulator protein showing a 2 aa CSI (highlighted), which is uniquely shared by all species from the Alcaligenes clade.

			119		160
**Oleovorans Clade (*Ectopseudomonas* gen. nov.) (15/15)**	*Pseudomonas oleovorans*	WP_150609166	AKGMKGAIEKANELAAANPD		YYLPQQFENPANPAIHEKTTGP
	Pseudomonas alcaliphila	WP_074675049	-------------------E		----------------------
	Pseudomonas chengduensis	WP_196448259	--------------V-----		----------------------
	Pseudomonas composti	WP_061239819	--------------V-----		----------------------
	Pseudomonas guguanensis	WP_090425658	--------------V-----		----------------------
	Pseudomonas hydrolytica	WP_037053461	--------------------		----------------------
	Pseudomonas indoloxydans	WP_084340656	--------------V----H		----------------------
	Pseudomonas khazarica	WP_037053461	--------------------		----------------------
	Pseudomonas mendocina	WP_003244618	--------------------		----------------------
	Pseudomonas pseudoalcaligenes	CDM40218	--------------V----H		----------------------
	Pseudomonas sediminis	WP_099525312	--------------------		-------------S--------
	Pseudomonas sihuiensis	WP_092374648	--------------V-----		----------------------
	Pseudomonas toyotomiensis	WP_206417632	--------------V-----		----------------------
	Pseudomonas wenzhouensis	WP_230924787	--------------------		----------------------
	Pseudomonas yangonensis	WP_161866302	--------------V-----		----------------------
**Other *Pseudomonas***	*Pseudomonas aeruginosa*	HBO2529771	--------Q--E--V-GD-G	K	-FM----D--------------
	Pseudomonas alcaligenes	MBB4819164	-----------T-I--G--E	Q	-L-L------------------
	Pseudomonas alcaligenes	WP_187805311	-------------I-S-D-A	K	--Q----D--------------
	Pseudomonas alloputida	WP_232858615	-------------IV-SD-E	Q	-F--G-----------------
	Pseudomonas argentinensis	WP_070886374	-R---------AQI--S--E	Q	--M-------------------
	Pseudomonas asuensis	WP_188867593	-----------K-I-ESD-A	R	-FM-------------------
	Pseudomonas azotifigens	WP_028239901	-----------A-I-QSD-E	R	-FM----D--------------
	Pseudomonas azotifigens	WP_181071675	-----------S-I--SD--	R	-FM-------------------
	Pseudomonas bananamidigenes	WP_065258256	-----------A-IV-GD--	K	-FM-A--D--------------
	Pseudomonas benzenivorans	WP_090448237	--------D--A-I--SD-A	T	-------D--------------
	Pseudomonas borbori	WP_090503158	---------R-T-I-----A	K	-F-----N---------T----
	Pseudomonas brassicacearum	WP_123582228	-----------A-IL-SD-A	R	-FM-------------------
	Pseudomonas campi	WP_173204978	-------------I-S-D-S	K	-FQ-G--D--------------
	Pseudomonas chlororaphis	WP_096373321	-----------A-IL-SD-E	K	-FM----D--------------
	Pseudomonas chlororaphis	WP_123413049	-----------TQIL-SD--	K	-FM----D--------------
	Pseudomonas corrugata	WP_208555417	--------D--A-I--SD-S	S	-FM----D--------------
	Pseudomonas cremoricolorata	WP_038412534	-----------G-IV-SD--	A	-FM-G-----------------
	Pseudomonas deceptionensis	WP_048360682	-----------S-IQ-SD-A	R	-FM-------------------
	Pseudomonas dryadis	WP_131176355	--------D--A-I---DS-	R	--M-------------------
	Pseudomonas duriflava	WP_145141937	-----------K-I-ESD-S	L	-FM-------------------
	Pseudomonas entomophila	WP_181105043	-----------E--L-S---	Q	-FM-G--D--------------
	Pseudomonas eucalypticola	WP_176571863	-----------E---N----	T	-FM----S--------------
	Pseudomonas fakonensis	WP_217839814	-------------IV-SD-A	Q	-F--G-----------------
	Pseudomonas faucium	WP_176517843	-------------IV-SD-A	Q	-F--G--D--------------
	Pseudomonas flexibilis	WP_039561977	-----------S-IV-SD-A	K	--M-------------------
	Pseudomonas fluorescens	WP_158152534	-----------E---N----	T	-FM----S--------------
	Pseudomonas fragi	WP_086798009	--------D--A-IL-SD-V	N	-FM-------------------
	Pseudomonas furukawaii	WP_003454865	-----------G-----H-E	Q	-L-L------------------
	Pseudomonas guariconensis	WP_196164739	-------------IV-SD-A	T	-F--G-----------------
	Pseudomonas helleri	WP_153382575	--------D--A-IQ-TD--	T	FFM-------------------
	Pseudomonas huaxiensis	WP_110968523	-------------IV-SD-S	T	-FM-A--D--------------
	Pseudomonas juntendi	WP_236184215	--------D---QIV-SD-A	Q	-F--G-----------------
	Pseudomonas kirkiae	WP_131184130	-----------H-IV--D-E	R	FF--------------------
	Pseudomonas kuykendallii	WP_090229032	--------D--S-I--S--G	E	-L-L------------------
	Pseudomonas lalkuanensis	WP_151135429	--------------V----E	Q	FL-L------------------
	Pseudomonas laurentiana	WP_163932865	-------------IV-SD-A	L	-FM----D--------------
	Pseudomonas linyingensis	WP_090307597	--------D--S-------E	Q	-L-L-------------E----
	Pseudomonas lopnurensis	WP_193681460	-----------G-I--SD-Q	Q	--M----D--S------R----
	Pseudomonas lundensis	WP_048375799	--------D--A-IQ-SD-A	Q	-FM-------------------
	Pseudomonas lutea	WP_197873459	-----------K-I-ESD-S	R	-FM-------------------
	Pseudomonas luteola	WP_019365211	-----------K-I-ESD-S	R	-FM-------------------
	Pseudomonas luteola	WP_239382807	-----------K-I-ESD-S	R	-FM---------------S---
	Pseudomonas mangrovi	WP_108105359	-----------A--L-Q--G	E	--M----D--------------
	Pseudomonas massiliensis	WP_040262081	--------D--R---ESD-E	R	-FM-------------------
	Pseudomonas mediterranea	WP_047701731	--------D--A-I--SD-S	S	-FM----D--------------
	Pseudomonas mohnii	WP_206770401	-----------G-IV-SD-S	K	-FM-A-----------------
	Pseudomonas monteilii	KXK67703	-------------IV-SD-A	Q	-F--G-----------------
	Pseudomonas mosselii	WP_138218347	-------------IV-SD-A	L	-F--G-----------------
	Pseudomonas muyukensis	WP_217849252	-------------IV-S--A	Q	-F--G--D--------------
	Pseudomonas nitrititolerans	WP_122077740	-----------S-I--SD-N	L	--M-------S-----------
	Pseudomonas nosocomialis	WP_138408710	-----------S-I--SD-E	R	-FM-------------------
	Pseudomonas oryzicola	WP_186676128	-------------IV-SD-A	Q	-F--G-----------------
	Pseudomonas oryzihabitans	MBB4994915	-----------R-I-ESE--	R	-FM----D--------------
	Pseudomonas oryzihabitans	WP_059314553	--------D--R-I-ESE--	R	-FM----D--------------
	Pseudomonas parafulva	WP_078478935	-------------IV-S--E	Q	-FM-G-----------------
	Pseudomonas plecoglossicida	WP_016391884	-------------IV-SD-E	Q	-F--G--D--------------
	Pseudomonas protegens	WP_123726431	-----------G-IV-SD-A	R	-FM----D--------------
	Pseudomonas psychrophila	WP_019828721	-----------T-IQ-SD-A	Q	--M-------------------
	Pseudomonas punonensis	WP_073268215	--------D--A-I--SDTS	K	--M-------------------
	Pseudomonas putida	WP_176685595	-------------IV-SA--	Q	-F--G-----------------
	Pseudomonas resinovorans	WP_016491795	-R------D--S----S--E	Q	-L-L------------------
	Pseudomonas resinovorans	WP_028629023	--------------V-S--E	Q	-L-L------------------
	Pseudomonas sagittaria	WP_092431055	--------D--S--------	Q	-L-L-------------Q----
	Pseudomonas saponiphila	WP_092320449	-----------A-IL-SD-S	K	-FM----D--------------
	Pseudomonas saudiphocaensis	WP_193773457	-----------S-I--S--E	L	--M-------S-----------
	Pseudomonas seleniipraecipitan	WP_070882900	-----------T-I--SDTA	K	--M-------------------
	Pseudomonas stutzeri	WP_045162642	-----------T-I--SDR-	Q	----K-----S--G--------
	Pseudomonas stutzeri	WP_102852044	-----------S-I--S--E	Q	----------S-----------
	Pseudomonas syringae	WP_236472477	--------D--G-IV-SD-S	T	-FM-A-----------------
	Pseudomonas taiwanensis	WP_179058233	-----------S---N---E	Q	-L-L------------------
	Pseudomonas thermotolerans	WP_017937772	-----------K-ILESA--	K	--M----D--------------
	Pseudomonas thivervalensis	WP_208667154	-----------A-IL-SD-S	R	-FM----D--------------
	Pseudomonas ullengensis	WP_183088668	-------------I-S-D-A	K	-FQ----D--------------
	Pseudomonas urumqiensis	WP_120996513	-----------A-I-N-D-A	Q	-F--------------------

Other *Pseudomonas*	*Pseudomonas viridiflava*	WP_122488320	--------A--E--L-G--E Q -FM-G-----------------
	Pseudomonas wadenswilerensis	WP_115088246	-------------IV-SE-S A -F--G-----------------
	Pseudomonas xantholysinigenes	WP_186661142	-------------IV-SD-A Q -F--G--D--------------
	Pseudomonas xanthomarina	WP_065983903	-----------S-I--SD-N L --M-------S-----------
	Pseudomonas xanthosomae	WP_217886065	-------------IV-SD-A Q -F--G-----------------
Other Bacteria	*Atopomonas hussainii*	WP_071872357	-----------A-I--SDSN K -L-L------------------
	Halopseudomonas pertucinogena	WP_188635388	-----------Q-ITQSD-Q K HI-L---------E--------
	Entomomonas moraniae	WP_109702219	--------D--N-IVNSD-S K -FMP---D--------------
	Moraxella macacae	WP_009501652	-L-----V---H-IVNSD-- K -F-PK--D--------IK--A-
	Acinetobacter baumannii	WP_163106010	--------Q--E--V-GD-G K -FM----D--------------
	Azomonas agilis	WP_144570437	-----------T-I--S--E L -F--------S-----------
	Azomonas macrocytogenes	WP_183167707	-----------T-I----TE Q -FM-------S-----------
	Azotobacter beijerinckii	WP_090623147	-----------T-I--S--E L -FM----D--------------
	Azotobacter chroococcum	WP_039803481	-----------T-I--S--E L -FM----D--------------
	Azotobacter salinestris	WP_152386550	-----------T-I--S--- L -FM----D-----E--------
	Escherichia coli	MRF43351	-------------IV-SD-A Q -F--G-----------------
	Marinobacter xestospongiae	WP_248168622	----P---A--E-M---D-- K -F---------------E----

Figure-S15

Partial sequence alignments of Cysteine synthase A protein showing a 1 aa CSI (highlighted), which is uniquely shared by all species from the Oleovorans clade. No other *Pseudomonas* species or other bacterial species share this CSI.

Group	Species	Accession	19		61
**Oleovorans Clade (*Ectopseudomonas* gen. nov.) (14/14)**	*Pseudomonas oleovorans*	NYF64131	VLGIILGLALLFAFIDELGDL	N	KGDYGLGQALWYVLLTAPRRAY
	Pseudomonas alcaliphila	WP_075749301	---------------------	-	----------------------
	Pseudomonas chengduensis	WP_196448856	---------------------	-	----------------------
	Pseudomonas composti	WP_061241119	---------------------	-	----------------------
	Pseudomonas guguanensis	WP_090431949	------------------S--	-	------A---------------
	Pseudomonas khazarica	WP_134676976	---------------------	-	----------------------
	Pseudomonas indoloxydans	WP_108235217	------------------S--	-	--------V-------------
	Pseudomonas mendocina	WP_047593069	---------------------	-	----------------------
	Pseudomonas pseudoalcaligenes	WP_003459661	---------------------	-	----------------------
	Pseudomonas sediminis	WP_099526575	---------------------	-	----------------------
	Pseudomonas sihuiensis	MBA2833073	---------------------	-	----------------------
	Pseudomonas toyotomiensis	WP_206418480	---------------------	-	----------------------
	Pseudomonas wenzhouensis	WP_230925286	------------------S--	-	--------V-------------
	Pseudomonas yangonensis	WP_161867559	---V-----------------	-	------A---------------
**Other *Pseudomonas***	*Pseudomonas aeruginosa*	WP_121274441	---V--------------N-I		SAS--I-D--RFIF--------
	Pseudomonas alcaligenes	TXI27231	-----V---------------		SSN--TLD--A-----L-----
	Pseudomonas anguilliseptica	WP_233683917	-----V--------------V		E-G-S-LD-S----------L-
	Pseudomonas argentinensis	WP_070887972	-----V-------L------I		E-N--I-D------------I-
	Pseudomonas asuensis	WP_188865482	---V---------Y--Q----		DDN--AL---T-----------
	Pseudomonas azotifigens	WP_181069516	-----V--------------I		E-S---AD--L--FM-----L-
	Pseudomonas balearica	WP_061338676	---V-V--------------V		E-G---L---E-----T---L-
	Pseudomonas benzenivorans	SDG87303	-----V--------------V		E-G---QE-A---F--L---V-
	Pseudomonas borbori	WP_090503556	---V-VS----------M--V		E-G---QD-A--IF--L---V-
	Pseudomonas carbonaria	WP_187671359	---V-V--------------V		E-G---LD-A------T---L-
	Pseudomonas cavernae	WP_119892558	---V-V--------------I		E-G---FD-A------T---V-
	Pseudomonas cavernicola	WP_119955983	-----V---M--S-----P-I		E-G---SDVA---------KV-
	Pseudomonas chloritidismutans	WP_226930535	-----V------------S-V		E-S----D--Q-----S---L-
	Pseudomonas citronellolis	WP_074979046	---V------------Q--A-		DEN-T--V--K-------S---
	Pseudomonas cremoricolorata	WP_028696651	---------S-------M---		SNT-T--D--M---------V-
	Pseudomonas daroniae	WP_131180742	-----V--------------I		E-N----D-----F------I-
	Pseudomonas delhiensis	WP_089389522	---V------------Q--A-		DEN-T--V--K-------S---
	Pseudomonas dryadis	WP_131175095	-----V--------------I		E-S-S--D-----F------I-
	Pseudomonas duriflava	WP_145136574	---V---------Y--Q---F		DDN--AWE--RF----T-----
	Pseudomonas entomophila	WP_181092611	---------S-------M---		SDT-TVWE-G----------L-
	Pseudomonas flavescens	WP_084307693	-----V--------------I		E-N----D-----F------V-
	Pseudomonas fluorescens	WP_126450378	---V--------------N-I		SAS--I-D--RFIF--------
	Pseudomonas fluvialis	WP_184685646	-----VS-------------V		E-G--VLD------------L-
	Pseudomonas fulva	WP_013792403	-----V-------L------I		E-N--I-D-----F------I-
	Pseudomonas guangdongensis	WP_090213843	-----V--------V------		SAA--VAE-AT----S----V-
	Pseudomonas indica	WP_084335703	-----V------S-------V		E-G---AE-FY-----T---I-
	Pseudomonas japonica	WP_181117108	---------T---L-------		TDT-T--D-A----------L-
	Pseudomonas jessenii	WP_146242009	---------T-------M-EV		TDT-T-VD--S---------M-
	Pseudomonas juntendi	WP_212624503	---------S-------M---		SDT-TVM--GY---------L-
	Pseudomonas kirkiae	WP_131183022	---V-V--------------I		--N---FD-FE-----G---L-
	Pseudomonas kunmingensis	WP_104097009	-----V------------S-V		E-G----D--Q-----S---L-
	Pseudomonas kuykendallii	WP_090227069	---V-V--------------I		EDS---LD-A----------I-
	Pseudomonas lalucatii	MBS7689737	-----V--------------V		E-N-S--A-A---V--L---V-
	Pseudomonas lopnurensis	MBE7375480	-----V--------------V		E-S-S-FD--Q-----S---L-
	Pseudomonas lutea	WP_197870911	---V---------Y--Q---F		DDN--AL---T-----G-----
	Pseudomonas luteola	WP_239381384	---V---------Y--Q---F		DDN--AL---T-----G-----
	Pseudomonas mangiferae	WP_143486972	---V----------------I		SES---K-VI---V--------
	Pseudomonas mangrovi	WP_108106066	----VV--------------V		E-E-S-AE-VG-----T---I-
	Pseudomonas marincola	WP_150548457	---V-V--------------I		DDN--MA--A------T-S---
	Pseudomonas massiliensis	WP_040261240	---------T---L-------		SDT-T--N-A----------L-
	Pseudomonas matsuisoli	WP_188982863	---V-TA---V-----Q---V		------ME-A---V------I-
	Pseudomonas monteilii	AMA47018	---------S-------M---		SDT-T-WE-G----------L-
	Pseudomonas nosocomialis	WP_138409195	-----V--------------I		E-S---FD--Q--FM-----L-
	Pseudomonas oryzihabitans	WP_160923621	---V-V----------Q---F		DDN--VAE--KF----S-----
	Pseudomonas otitidis	WP_165664651	---V-IS-------------I		--N-T-MD-IC-----------
	Pseudomonas peli	WP_090248786	-----V--------------V		E-N---LD--G-----S---I-
	Pseudomonas psychrotolerans	WP_058767759	---V-V----------Q---F		DDN--VAE--KF----T-----
	Pseudomonas punonensis	WP_073261426	-----V--------------I		E-S--I-D------------I-
	Pseudomonas seleniipraecipitan	WP_070883337	-----V--------------I		E-S--I-D-----F------I-
	Pseudomonas songnenensis	WP_122098253	-----V--------------V		Q-G----D--Q-----S---L-
	Pseudomonas straminea	WP_093504258	-----V-------L------I		E-N--I-D-----F------I-
	Pseudomonas stutzeri	NRF47774	-----VC-------------V		Q-N----D--Q-----S---L-
	Pseudomonas taeanensis	WP_025165048	---V-V--------------V		E-N---LE-S---F--L---V-
	Pseudomonas thermotolerans	WP_027895949	---V-V--------------V		E-G---LD-A----------L-
	Pseudomonas viridiflava	WP_122452554	-----V--------------V		Q-S----D-A------S---V-
	Pseudomonas xionganensis	WP_160344035	-----V--G-----------I		ERN-RVQD-A------T---I-
**Other Bacteria**	*Stenotrophomonas maltophilia*	WP_164111571	---V------------G-N-I		SAS--I-D--RFIF--------
	Azomonas macrocytogenes	WP_183164887	---V-V--V-----------V		--S--TLD--I-----M---I-
	Azotobacter beijerinckii	WP_090939760	---V-V--------------V		R-N-D-AA-A---F--V-Q-I-
	Azotobacter chroococcum	WP_131300050	---V-V--T-----V-----V		R-N-D-AA-A---F--V-Q-T-
	Azotobacter salinestris	WP_152388839	---V-V--S-----V-----V		R-N-D-VA-A------V-Q-T-
	Streptococcus pneumoniae	CJK97195	-----V--------------V		Q-N----D--Q-----S---L-

Figure-S16

Partial sequence alignments of Lipopolysaccharide export system permease protein showing a 1aa Ins (highlighted), which is uniquely shared by all species from the Oleovorans clade. Homolog of this protein was not found in *P. hydrolytica*. No other *Pseudomonas* species or other bacterial species share this CSI.

			121		164
**Oleovorans Clade (*Ectopseudomonas* gen. nov.) (13/13)**	*Pseudomonas oleovorans*	WP_125875007	GFEAIRACDLEHLAATFFGK	Q	TGRTRLHYDLHTAIRGSKIEQFA
	Pseudomonas alcaliphila	WP_075746630	--------------------	-	-----------------------
	Pseudomonas chengduensis	WP_196447169	--------------------	-	-----------------------
	Pseudomonas composti	WP_037002626	-------------VT-----	-	S----------------------
	Pseudomonas guguanensis	WP_090426003	---------------N----	D	-----------------------
	Pseudomonas pseudoalcaligenes	WP_003461881	--------------------	-	-----------------------
	Pseudomonas khazarica	WP_134676192	-------------S------	E	-----------------------
	Pseudomonas mendocina	WP_047590898	-------------S------	E	-----------------------
	Pseudomonas sediminis	WP_179545256	--------------VN----	D	---------------A-------
	Pseudomonas sihuiensis	WP_092374495	--------------------	-	-----------------------
	Pseudomonas toyotomiensis	WP_074914060	--------------------	-	------------------V----
	Pseudomonas wenzhouensis	WP_230924913	-------------CVN----	D	S--------------A-------
	Pseudomonas yangonensis	WP_161866402	--------------VN----	D	---------------A-------
**Other *Pseudomonas***	*Pseudomonas abietaniphila*	WP_062389368	-S--L---E--R---S--SL		P--Y-------------------
	Pseudomonas aestus	WP_022640321	-H--L---E--R---S--SL		P--E-------------------
	Pseudomonas akappageensis	WP_166359759	-T--L---E--R------SM		P----------------------
	Pseudomonas alcaligenes	MBB4817518	----M--NE--R---A--I-		P----------------------
	Pseudomonas alcaligenes	WP_203791950	----L--NE--RF--A--S-		E--A-------------------
	Pseudomonas alkylphenolica	WP_197876233	-T--L---E--R--T---SV		P----------------------
	Pseudomonas amygdali	WP_005746299	-A--L---E------S--SL		PD-Y-------------------
	Pseudomonas anatoliensis	WP_210700338	-S--L---E--R---V--SQ		PD-Q-------------------
	Pseudomonas anguilliseptica	WP_233686403	-A--L-------------S-		PE---------------------
	Pseudomonas atacamensis	WP_206421707	-S--L---E--R---S--S-		PE-Q-------------------
	Pseudomonas bananamidigenes	WP_065261775	-Y--L---E--R---S--SV		PD-Q-------------------
	Pseudomonas batumici	WP_040070190	-S--L---E--R---S--NL		P--E-------------------
	Pseudomonas benzenivorans	WP_090447417	----M-------------S-		P----------------------
	Pseudomonas borbori	WP_090499498	-V--M-------------S-		PE-------------T-Q-----
	Pseudomonas brassicacearum	WP_025215102	-A--L---E--R---S--SR		PE-S-------------------
	Pseudomonas brassicae	WP_163945432	-V--L---A--R--T---SV		Q----------------------
	Pseudomonas bubulae	WP_218722804	-P--L---E-----E---SP		P----------------------
	Pseudomonas cavernae	WP_119892924	----L--AE--R------S-		PE---------------------
	Pseudomonas chlororaphis	WP_063432686	-S--L---E--R---S--SL		PD---------------------
	Pseudomonas corrugata	WP_053192401	-P--L---E--R---S--SR		PE-S-------------------
	Pseudomonas crudilactis	WP_180698584	-S--L---E--R---S--S-		PD-Q-------------------
	Pseudomonas deceptionensis	WP_048359771	-P--L---E-----E---SP		PE---------------------
	Pseudomonas dryadis	WP_131197885	-G--M---E----------D		P--S-----------A-------
	Pseudomonas ekonensis	WP_217890936	-C--L---E--R---S--SL		P--Q-------------------
	Pseudomonas endophytica	WP_055101130	-P--L---E-----E---NQ		PD-G---F---------------
	Pseudomonas farsensis	WP_186537066	----L--AE--QF-RV--S-		P----------------------
	Pseudomonas ficuserectae	WP_235813833	-A--L---E------S--SL		PD-Y-------------------
	Pseudomonas flavescens	WP_179538706	-A--M---E------S--AD		A--------------A-R-----
	Pseudomonas fluorescens	VVN41084	-S--L---E--R---S--S-		PD-Q-------------------
	Pseudomonas fragi	WP_095039364	-P--L---E-----E---SP		P----------------------
	Pseudomonas frederiksbergensis	WP_071553779	----L---E--R---S--NL		PD-D-------------------
	Pseudomonas fulva	WP_042553125	-A--M---E------S--AD		A--------------A-R-----
	Pseudomonas graminis	WP_133773359	-S--L---E--R---S--NI		P--Y-------------------
	Pseudomonas granadensis	WP_090285399	-S--L---E--R---I--S-		PE-Q-------------------
	Pseudomonas guineae	WP_090244426	-A--L---E------A--S-		PE-------------A-------
	Pseudomonas hamedanensis	WP_186551453	-S--L---E--R---S--S-		PD-Q-------------------
	Pseudomonas helleri	WP_048390131	-P--L---E-----E---KA		PE-----F-----M---------
	Pseudomonas huaxiensis	WP_110969530	-S--L---E--R--T---SL		P----------------------
	Pseudomonas indica	WP_084339114	----L--AE--R--TS--S-		P----------------------
	Pseudomonas japonica	WP_042124495	-S--L--G----------SV		P----------------------
	Pseudomonas japonica	WP_181122980	----M---E--R---S--NR		PD-Q-------------R-----
	Pseudomonas jessenii	WP_115146183	-S--L---E--R---V--SQ		PD-Q-------------------
	Pseudomonas khorasanensis	WP_186529814	-S--L---E--R---S--S-		PD-Q-------------------
	Pseudomonas koreensis	WP_064588778	-S------E--R---S--SL		P--Q-------------------
	Pseudomonas lalucatii	WP_213640552	----L---E---------SQ		P----------------------
	Pseudomonas leptonychotis	WP_136665809	-A--L----------N--S-		PE-------------A-------
	Pseudomonas lundensis	WP_169880269	-S--L---E-----TA--SL		A-----------------L----
	Pseudomonas meliae	WP_044344461	-A--L---E------S--SL		PD-Y-------------------
	Pseudomonas moraviensis	WP_065616777	-S--L---E--R---S--S-		PE-Q-------------------
	Pseudomonas otitidis	WP_165681179	-T--M--NE------A--AR		E-----------------V----
	Pseudomonas palleroniana	WP_151152781	-P--L---E--R---S--SR		PE-S-------------------
	Pseudomonas peli	WP_090251798	-A--L----------A--S-		PE-------------A-------
	Pseudomonas pharyngis	WP_236191290	-S--L---E--R---S--SL		P--Q-------------------
	Pseudomonas piscis	WP_152898627	-H--L---E--R---S--SL		P--E-------------------
	Pseudomonas protegens	QEN49543	----L---E--R---S--SQ		PD-Q-------------------
	Pseudomonas psychrophila	WP_019408683	-P--L---E-----K---NQ		PE---------------------
	Pseudomonas punonensis	WP_073264244	-A--M---E------S--AD		-E-------M-----A-R-----
	Pseudomonas putida	WP_064301974	-P--L--G----------SI		P----------------------
	Pseudomonas qingdaonensis	WP_213607202	-V--L---A--R--TA--Y-		V----------------------
	Pseudomonas savastanoi	RMS81522	-A--L---E------S--SL		PD-Y-------------------
	Pseudomonas segetis	WP_089359227	-------SE-----SA--RE		PE-S-------------------
	Pseudomonas shirazica	WP_217190721	----L--AE--QF-RV--S-		---S-------------------
	Pseudomonas siliginis	WP_217852946	-S--L---E--R---S--S-		PE-Q-------------------
	Pseudomonas syringae	WP_057441194	-A--L---E------S--SL		PD-Y-------------------
	Pseudomonas taeanensis	WP_025165374	-A--M---E------G--S-		P--------------A-------
	Pseudomonas tohonis	WP_173172312	----M--NE--R---A--I-		P----------------------
	Pseudomonas tolaasii	WP_017254719	-P--M--AE--Q--R---SQ		A--S-------------------
	Pseudomonas triticicola	WP_217862746	-S--L---E--R---S--S-		PE-D-------------------
	Pseudomonas tructae	WP_130263715	-A--L---E--R------SV		PE---------------------
	Pseudomonas urethralis	WP_176509059	----L--AE--QF-RV----		P----------------------
	Pseudomonas uvaldensis	WP_232776302	-P--L---E--R---S--N-		PE-S-------------------
	Pseudomonas versuta	WP_060694484	-P--L---E-----E---SL		PE---------------R-----
	Pseudomonas viciae	WP_135846802	-V--L---E--R---S--SR		PE-S-------------------
	Pseudomonas viridiflava	WP_025992929	-S--L---E--R------KL		PD-Y-------------------
	Pseudomonas vlassakiae	WP_186604497	----L--AE--QF-RV--S-		P----------------------
	Pseudomonas vranovensis	WP_123567960	-P--L---E--R------SV		PD---------------------

Group	Species	Accession	Sequence	
Other *Pseudomonas*	*Pseudomonas weihenstephanensis*	WP_048363483	-P--L---E-----QA--NA	PD---------------------
	Pseudomonas xionganensis	WP_160346455	-P--L-------------SQ	PE-S-------------------
	Pseudomonas zanjanensis	WP_186707066	-P--L---E--R---S--SR	PE-S-------------------
Other Bacteria	*Tanacetum cinerariifolium*	GEW94602	-S--L---E--R---S--SI	A--Y-------------------
	Aeromonas caviae	GJB78968	----L--AE--QF-RV--S-	---S-------------------

Figure-S17

Partial sequence alignments of a protein Succinylglutamate desuccinylase showing a 1 aa CSI (highlighted), which is uniquely shared by all species from the Oleovorans clade. Homologs were not found in the species *P. hydrolytica, P. indoloxydans*. No other *Pseudomonas* species or other bacterial species share this CSI.

Group	Species	Accession	124		155
Oleovorans Clade (*Ectopseudomonas* gen. nov.) (14/15)	*Pseudomonas oleovorans*	WP_206407640	PDEYDGGELVVQDQR		IKLPAGHLVLYSSGSLH
	Pseudomonas alcaliphila	WP_075745518	---------------		-----------------
	Pseudomonas chengduensis	WP_021488021	---------------		--------I--------
	Pseudomonas composti	WP_036999245	----E---------C		--------I--------
	Pseudomonas guguanensis	WP_090427504	-E--E----------		--------I--------
	Pseudomonas hydrolytica	MCF2122804	----E--------K-		--------I--------
	Pseudomonas indoloxydans	WP_108234333	---------------		-----------------
	Pseudomonas khazarica	WP_037046309	----------TRER-		-----------------
	Pseudomonas mendocina	WP_003242794	----E--------K-		--------I--------
	Pseudomonas pseudoalcaligenes	WP_004424912	---------------		-----------------
	Pseudomonas sediminis	WP_099522542	----E----------		-----------------
	Pseudomonas sihuiensis	WP_011920963	-E--E--------K-		--------I--------
	Pseudomonas toyotomiensis	WP_059390464	----E----------		-----------------
	Pseudomonas yangonensis	WP_161864892	-H--------M--R-		-------M------C--
	Pseudomonas wenzhouensis	WP_230926647	-----------H-HF	GSHR	A-------L--P-----
Other *Pseudomonas*	*Pseudomonas anguilliseptica*	WP_233683697	-----------E-SY	GSHR	V----------PGS---
	Pseudomonas arsenicoxydans	WP_140664107	-ED------EI--TF	GTQR	V-----DM---PGT---
	Pseudomonas balearica	MBC7199262	-------D--I--TY	GTQR	V-FA--DM---P-T---
	Pseudomonas brassicacearum	WP_123424230	---------EI--TF	GTQR	V-----D----PGT---
	Pseudomonas carbonaria	WP_187671303	----E-----IE-SY	GSHR	V----------P-----
	Pseudomonas carnis	WP_197880118	---------E---TF	GLQR	V-----DM---PGS---
	Pseudomonas cavernae	WP_119894936	--D-A-----I--TY	GSQR	V-----D-L--PAS---
	Pseudomonas cremoris	WP_185708320	---------EI--TF	GLQR	V-----DM---PGS---
	Pseudomonas flavescens	WP_179539897	-----------E-SY	GSHS	V-------I--PGT---
	Pseudomonas fluorescens	WP_019691846	---------EI--TF	GTQR	V-----D----PGT---
	Pseudomonas fragi	NNG65408	--D-------I--TY	GVQQ	V-----DM---PGT---
	Pseudomonas frederiksbergensis	WP_123516430	--D------EI--TF	GTQR	V-----DM---PGS---
	Pseudomonas furukawaii	WP_003452027	--D--------E-TY	GSHR	V----------PGS---
	Pseudomonas germanica	WP_220557780	--D------EI--TF	GTQR	V-----DM---PGT---
	Pseudomonas gessardii	WP_197881269	---------EI--TF	GLQR	V-----DM---PGS---
	Pseudomonas guineae	WP_090239998	-----------E-SY	GSHR	V----------PGS---
	Pseudomonas lundensis	WP_047292113	--D-------I--TY	GVQR	V-----DM---PGT---
	Pseudomonas mandelii	WP_033058090	--D-E----EI--TF	GTQR	V-----DM---PGT---
	Pseudomonas nabeulensis	WP_135307765	---------EI--TF	GLQR	V-----DM---PGS---
	Pseudomonas otitidis	WP_107331681	--D-E------E-TY	GSHR	V----------PGS---
	Pseudomonas peli	WP_090249244	----E------E-SY	GSHS	V----------PGS---
	Pseudomonas piscicultурae	WP_194936737	---------EI--TF	GLQR	V-----DM---PGS---
	Pseudomonas poae	WP_105697413	---------EI--TF	GLQR	V-----DM---PGS---
	Pseudomonas poae	WP_123714538	---------EI--TF	GLQR	V-----DM---PGS---
	Pseudomonas punonensis	WP_073262228	-E--------IE-TY	GSHS	V----------PGT---
	Pseudomonas resinovorans	WP_016490915	--D-Q------E-RY	GSHR	L----------------
	Pseudomonas schmalbachii	WP_208312946	----------I--TY	GTQQ	V-----D----PAT---
	Pseudomonas songnenensis	WP_122099383	----------I--TY	GTQR	V-FA--DM---P-T---
	Pseudomonas straminea	WP_093499808	----------IE-TY	GSHS	-----------PGT---
	Pseudomonas stutzeri	PZR54619	-------D--I--TY	GTQR	V-FA--DM---P-T---
	Pseudomonas syringae	WP_032624850	---------EI--TY	GTQR	V-----DM---P-T---
	Pseudomonas taeanensis	WP_025166921	----------I--TF	GTQR	------D-L--PAT---
	Pseudomonas thermotolerans	WP_027896444	-E---------E-TY	GSHR	V------M---TG----
	Pseudomonas viridiflava	WP_122427863	----E----EI--TF	GVQR	V-----DM---PGS---
	Pseudomonas xionganensis	WP_160347213	----E--------HF	GSHR	A-------L--P-----
Other Bacteria	*Achromobacter pestifer*	WP_173143083	--S-------ID-TY	GPRA	V------M---P-T---
	Achromobacter xylosoxidans	CUI50039	--S--------D-TY	GPRS	V------M---PGT---
	Acidobacterium capsulatum	WP_015896422	-E-------IIE-TY	GIHS	V------M---P-S---
	Advenella mimigardefordensis	WP_025373198	----E----II--TY	GEQR	V-M----M-V-P-T---
	Alcaligenes faecalis	WP_035271318	----E-----IE-VY	GTQS	-------M-V-PGT---
	Alcaligenes pakistanensis	WP_189391677	----E----IIE-VY	GTQS	-------M-V-PGT---
	Alteromonadaceae bacterium	NWO04663	--T-E----I-E-TY	GAHS	V-----D-I--P-S---
	Aquabacterium lacunae	WP_130968282	----E-----IE-TF	GPRR	V------M---P-T-V-
	Arenimonas composti	WP_026816856	----E-----I--VF	GDRR	V----------PGT-V-
	Azomonas agilis	WP_144572258	-N--E----II--TY	GVQP	V-----S----P-T-V-
	Azomonas macrocytogenes	WP_183167125	--A-------IE-TY	GSQR	V------MA--P-T---
	Azotobacter beijerinckii	WP_090622243	----------I--TY	GMQR	V-----D----PAT-I-
	Azotobacter chroococcum	WP_089167376	----------I--TY	GMQR	V-----D----PAT-I-
	Azotobacter salinestris	WP_152387063	----------I--TY	GVQR	V-----D----PAT-I-
	Azotobacter vinelandii	WP_012699881	----------IR-TY	GTQR	V-------A--PGT---
	Bacillus subtilis subsp. subti	MCF7749664	----Q-----IE-TY	GTQR	V------M---PGT---
	BetaproteoBacteria bacterium	MBI3716011	----------IE-TF	GSQS	V-----EMI--P-S---
	Bordetella bronchiseptica	WP_003820358	--S--------D-TY	GPRT	V------M---PGT---
	Bordetella pertussis	WP_050857271	--S--------D-TY	GPRT	V------M---PGT---
	Bowmanella denitrificans	WP_102798401	----------ME-TY	GSQR	V--A--DM---P-T---
	Brevundimonas lenta	WP_183205423	---------IIEEMY	GPQS	V-----D----P-K---
	Burkholderia cenocepacia	WP_124702786	----------IE-SY	GVHT	-------MI--PA----
	Burkholderia gladioli	WP_186038872	----------IE-SY	GSHT	V------M---PA----
	Burkholderia plantarii	WP_055140973	----------IE-SY	GSHT	V------M---PA----
	Candidatus Manganitrophaceae b	TAK05844	----E-----IE-TY	GVQR	---S-------P-T---
	Castellaniella caeni	WP_066459876	----E-----I--TY	GEQR	V------A-V-PGT---
	Castellaniella defragrans	WP_151024063	----E-----I--TY	GEQR	V------A-V-PGT---
	Cellvibrio polysaccharolyticus	MBE8718144	--D-------IE-TY	GSQS	-----------PGT---
	Chitinilyticum aquatile	WP_028453271	---------I-E-TY	GLHE	V-----DMI--P-S---
	Cupriavidus basilensis	AJG18432	-ED--------E-TY	GQQR	V------M---PAT---
	Cupriavidus gilardii	WP_174106347	----------IE-LY	GSHT	V----------PAS---
	Cupriavidus necator	WP_058696955	----------IE-TY	GQQR	V------M---PAT-I-
	Cupriavidus oxalaticus	WP_063237152	--Q--------E-TY	GQQR	V------M---PAT-V-
	Denitrificimonas caeni	WP_205342969	-----------E-SY	GAHS	V-----DMI--P-S---
	Dyella solisilvae	WP_114825249	---------I-E-TY	GSHT	V------M---P-S---
	Elstera cyanobacteriorum	WP_094408592	-E-------LIE-VY	GPQR	V-------I--P-T---
	Elstera litoralis	WP_045777626	-E-------IIE-VY	GPQR	V-------I--P-T---
	Entomomonas moraniae	WP_109704110	-E--E-----I--TY	GEQR	VR----D----P-T---

Group	Species	Accession	Sequence
Other Bacteria	*Fontimonas thermophila*	WP_091532331	----E----I-E-LY GIHR ---A-------PAS-V-
	Gluconobacter morbifer	WP_040507322	----------I--TY GHQS V------M---P-T---
	Granulicella tundricola	WP_013581066	-----------E-SY GEHK V------M---PAT---
	Haloferula luteola	WP_184020195	-E-------C-E-TY GVQK V------M---P-T---
	Klebsiella pneumoniae	SQC43604	-ES-E-----IE-TY GQHR V----------PAS---
	Klebsiella variicola	PLM82873	-ES-E-----IE-TY GQHR V----------PAS---
	Listeria monocytogenes	WP_149120575	-E--------I--TY GVQQ V-----DM---PGT---
	Luteimonas abyssi	WP_058833945	-----------E-TY GTQR ---R----A--PGT---
	Luteolibacter pohnpeiensis	WP_200272174	---------IIE-TY GAKS V------M---P-T---
	Luteolibacter yonseiensis	WP_200350904	-E-------CIE-TY GVQR V------M---PAT---
	Marinomonas gallaica	WP_067030119	----E-----IE-TF GTQQ ------DM---P-T-I-
	Marinomonas vulgaris	WP_211537206	----E-----IE-TF GQQK ------DM---P-T---
	Methylophilus methylotrophus	TXI38029	----E--------VF GDQR ---K--SI-I-P-S-V-
	Methyloradius palustris	WP_221763654	--D------TIE-VY GEQK V------M---P-T---
	Niveispirillum cyanobacterioru	WP_102115010	----E------E-LY GHHA V------M---P-S---
	Niveispirillum irakense	WP_029012732	----------IE-LF GTHQ V------M---P-S---
	Oceanimonas doudoroffii	WP_094200355	----E------E-TY GAHS V-----D-I--P-S---
	Oceanisphaera marina	WP_188628794	----E-----IE-SY GAHS V-----D-I--P-T---
	Oceanisphaera profunda	WP_087035110	----E------E-NY GAHS V-----D-I--P-T---
	Oleisolibacter albus	WP_114393923	---------RI--TY GTHQ V------M---P-T---
	Paenirhodobacter enshiensis	WP_036636151	-------------TY GSQR V------M-V-P-S---
	Paraburkholderia megapolitana	WP_091006072	----E------D-AH GERS -------MIV-PAST--
	Paraburkholderia phosphatilyti	WP_118178406	----------IE-TY GVQQ V-------IV-PAT---
	Parvibaculum lavamentivorans	WP_011995291	----E----I-E-TY GSHA -------M---PGT---
	Parvibium lacunae	WP_114402462	----E-----IE-TY GSQR ---A--DM---P-S-V-
	Pokkaliibacter plantistimulans	WP_110186879	----E------E-TY GTHQ V------MI--P-T---
	Pontibaca methylaminivorans	WP_076649881	-E--------IE--F GAQQ V------M---P-T---
	Prosthecobacter dejongeii	MBB5039531	-E--E----CIE-LY GVKT V------M---P-T---
	Pseudoxanthomonas suwonensis	WP_052630989	---------M---TY GEHR VR-----ML--PGT---
	Pusillimonas noertemannii	WP_026067789	-G--E----I---SY GAHT V------M-V-PGS---
	Rhizopus arrhizus	KAG1442905	--S-------ID-TY GPRA V------M---PGT---
	Rubritalea squalenifaciens	WP_143184632	----E--V-TIE-TY GTQE V------MI--PAT---
	Shewanella fodinae	WP_189617377	--S-E-----I--TY GQQR V--S--S----P-T---
	Solimonas aquatica	WP_093282633	-E--E----L-E-TY GVQR V-------I--PAS---
	Solimonas flava	WP_028007861	---------I-E-TY GAHS V----------P-S---
	Sphingomonadales bacterium	MBW8842927	--S-------IE--F GEQR V------M---PAS-V-
	Sphingomonas adhaesiva	WP_066708616	-AA-------IE-RF GEQR V------M---PAS---
	Sphingomonas aerophila	WP_184056912	--S--------E-VF GEQR V------M---PAS---
	Sphingomonas asaccharolytica	WP_066807740	--S-------IE--F GEQR V------M---PAS-V-
	Sphingomonas aurantiaca	WP_107956336	--S--------E-RF GPQS V------M---PAS---
	Sphingomonas carotinifaciens	WP_112383187	--S-------IEE-F GEQR V------M-V-PAS---
	Sphingomonas changnyeongensis	WP_160593617	--S-------IE-RF GEQR V------M---PAS-V-
	Sphingomonas corticis	WP_168133445	-ED-------IE--F GEHR V----------PAS---
	Sphingomonas hominis	WP_174192910	--A-------IE--F GEQR V------M---PAS---
	Sphingomonas laterariae	WP_089219514	--S-------IE--F GVQS V------MI--PAS---
	Sphingomonas mali	WP_066825920	--S-------IE--F GEQR V------M---PAS-V-
	Sphingomonas palmae	WP_093004815	--T-------IE--F GEQR V------M---PAS---
	Sphingomonas panacisoli	WP_146572694	--S-N-----IE-SF GEQR V------M---PAS-V-
	Sphingomonas pruni	WP_066593466	--S-------IE--F GEQR V------M---PAS-V-
	Sphingomonas sanxanigenens	PZO91450	--D-------IE-MF GEHR V-------IV-PGS---
	Sphingomonas solaris	WP_145155314	--S--------E-HY GVQA V-------L--PAS---
	Sphingosinicella flava	WP_200971500	--S-E------E-NL GAHK V------MI--PAS---
	Stella vacuolata	BBK42147	-ED------S-E-TY GVQQ -----------P-S---
	Stenotrophomonas humi	WP_057632812	----------IE-TY GNQR V------M---PGT---
	Stenotrophomonas maltophilia	VUK28671	----E-----IE-TF GTQR V------M---PGT---
	Stenotrophomonas pictorum	WP_054658277	----E-----IE-TY GNQR V------M---PGT---
	Stenotrophomonas rhizophila	WP_121043200	----E-----IE-TY GNQR V------M---PGT---
	Tatumella saanichensis	WP_029684513	----Q-----IK-TY GEHR V-----DML--P-----

Figure-S18

Partial sequence alignments of a protein Fe2+-dependent dioxygenase showing a 4 aa Del (highlighted), which is uniquely shared by all species from the Oleovorans clade except *P. wenzhouensis*. No other *Pseudomonas* species or other bacterial species share this CSI.

Group	Species	Accession	46		85
Oleovorans Clade (*Ectopseudomonas* gen. nov.) (13/13)	*Pseudomonas oleovorans*	WP_206408901	PFELTRRGTIRATLRGRQSEY	ERF	DRAVSVHLDTIGAIVR
	Pseudomonas alcaliphila	WP_074681510	---------------------	---	----------------
	Pseudomonas chengduensis	WP_021489747	---------------------	---	----------------
	Pseudomonas composti	WP_061238075	---------------------	---	----------------
	Pseudomonas guguanensis	WP_090431360	---------------------	---	----------------
	Pseudomonas khazarica	WP_037053167	---------------------	---	-----A----------
	Pseudomonas mendocina	WP_047584342	---------------------	---	-----A----------
	Pseudomonas pseudoalcaligenes	WP_003461497	---------------------	---	----------------
	Pseudomonas sediminis	WP_099523304	---------------------	---	----------------
	Pseudomonas sihuiensis	WP_017679030	---------------------	---	----------------
	Pseudomonas toyotomiensis	WP_196460482	---------------------	---	----------------
	Pseudomonas wenzhouensis	WP_230927275	---------------------	---	----------------
	Pseudomonas yangonensis	WP_161866954	---------------------	---	----------------
Other *Pseudomonas*	*Pseudomonas pohangensis*	WP_090198200	---------------------	D--	-----A-------S--
	Pseudomonas abietaniphila	WP_074752551	--------------K---NSP		-----A-------S--
	Pseudomonas aeruginosa	WP_132476508	--------------K---TSP		-----A-------S--
	Pseudomonas alcaligenes	WP_061904806	--------------K---TSP		-----A----------
	Pseudomonas alkylphenolica	WP_197877103	--------------K--K-SP		-----A-------S--
	Pseudomonas amygdali	WP_122393159	--------------K-K-NSP		-----A-------S--
	Pseudomonas anguilliseptica	WP_233684267	---M-------G--K-K--SP		-----A----------
	Pseudomonas arcuscaelestis	WP_203543007	--------------K--K-SP		-----A-------S--
	Pseudomonas argentinensis	KAB0545609	---M----------K-QKNSP		-----A-------A--
	Pseudomonas asplenii	WP_090206581	--------------K----SP		-----A-------A--
	Pseudomonas bananamidigenes	WP_065257185	---M----------K-K-NSP		-----A-------T--
	Pseudomonas benzenivorans	WP_090444628	---M-------G--K-Q--SP		-----A----------
	Pseudomonas bohemica	WP_110948276	--------------K-K-NSP		-----A-------S--
	Pseudomonas borbori	WP_090496823	---M-------G--K-K--SP		-----A----------
	Pseudomonas brassicacearum	ROM71287	---M----------K--K-SP		-----A-------S--
	Pseudomonas brassicae	WP_163940761	--------------K--K-SP		-----A-------S--
	Pseudomonas campi	WP_173204781	--------------K---TSP		-----A----------
	Pseudomonas cannabina	WP_055001305	--------------K-K-NSP		-----A-------S--
	Pseudomonas capeferrum	WP_181133284	--------------K----SP		-----A-------S--
	Pseudomonas carbonaria	WP_187672617	--------N-----K----SP		-----A----------
	Pseudomonas caspiana	WP_087265450	--------------K-K-NSP		-----A-------A--
	Pseudomonas cerasi	WP_065350362	--------------K-K-NSP		-----A-------S--
	Pseudomonas cichorii	WP_122314704	--------------K-K-NSP		-----A-------S--
	Pseudomonas coleopterorum	WP_192000118	---M----------K-K-NSP		-----A-------S--
	Pseudomonas coronafaciens	RMS32719	--------------K-K-NSP		-----A-------S--
	Pseudomonas corrugata	WP_039588825	---M----------K--K-SP		-----A-------S--
	Pseudomonas cremoricolorata	WP_028694825	-------------------SP		-----A-------S--
	Pseudomonas duriflava	WP_145140571	-Y------------K--KESP		-----A----------
	Pseudomonas entomophila	WP_011532752	-Y------------K----SP		-----A-------S--
	Pseudomonas fakonensis	WP_217842290	--------------K----SP		-----A-------S--
	Pseudomonas faucium	WP_236237448	--------------K----SP		-----A-------S--
	Pseudomonas ficuserectae	WP_235813751	--------------K-K-NSP		-----A-------S--
	Pseudomonas floridensis	WP_083184509	--------------K-K-NSP		-----A-------S--
	Pseudomonas fluorescens	WP_214913265	---M----------K----SP		-----A-------S--
	Pseudomonas frederiksbergensis	WP_123494544	---M----------K-K-NSP		-----A-------A--
	Pseudomonas fulva	WP_196181379	--------------K----SP		-----A-------S--
	Pseudomonas furukawaii	WP_003450334	-Y------------K----SP		-----A----------
	Pseudomonas fuscovaginae	WP_017906379	--------------K----SP		-----A-------A--
	Pseudomonas gingeri	WP_177065319	--------------K----SP		-----A-------A--
	Pseudomonas graminis	WP_172611183	--------------K-K-NSP		-----A-------S--
	Pseudomonas guariconensis	WP_196163965	--------------K---NTP		-----A-------S--
	Pseudomonas guineae	WP_090238376	---M-------G--K-K--SP		-----A----------
	Pseudomonas juntendi	WP_247732043	--------------K---TSP		-----A-------S--
	Pseudomonas koreensis	MBB4057235	---M----------K-KK-SP		-----A-------A--
	Pseudomonas lactis	MCF5082301	---M----------K-QKNSP		-----A-------A--
	Pseudomonas lalucatii	WP_213638282	--------------K-K--SP		-----T----------
	Pseudomonas lutea	WP_037013810	--------------K-K-NSP		-----A-------S--
	Pseudomonas mangrovi	WP_108108111	---M----------K--KPSP		-----A----------
	Pseudomonas maumuensis	WP_217866846	--------------K----SP		-----A-------S--
	Pseudomonas meliae	WP_044345472	--------------K-K-NSP		-----A-------S--
	Pseudomonas mohnii	WP_242070932	---M----------K-KKNSP		-----A-------A--
	Pseudomonas monteilii	AMA45193	-Y------------K----SP		-----A-------S--
	Pseudomonas moraviensis	WP_123418986	---M----------K-KK-SP		-----A-------A--
	Pseudomonas mosselii	WP_110736489	--------------K---NSP		-----A-------S--
	Pseudomonas oryzicola	WP_186678159	--------------K----SP		-----A-------S--
	Pseudomonas oryzihabitans	HJE70584	-------------IK-HKESP		-----A----------
	Pseudomonas oryziphila	WP_125857798	--------------K----SP		-----A-------S--
	Pseudomonas otitidis	WP_165666395	--------------A---NSP		----AA----------
	Pseudomonas parafulva	WP_029611958	--------------K----SP		-----A-------S--
	Pseudomonas peradeniyensis	WP_186733763	--------------K---NSP		-----A-------S--
	Pseudomonas plecoglossicida	WP_016391213	---------V----K----SP		-----A-------C--
	Pseudomonas promysalinigenes	WP_186476553	-Y------------K----SP		-----A-------S--
	Pseudomonas protegens	WP_210668797	--------------K----SP		-----A-------S--
	Pseudomonas psychrotolerans	WP_058793024	-------------IK-HKESP		-----A----------
	Pseudomonas putida	WP_049272834	--------------K---TSP		-----A-------S--
	Pseudomonas reidholzensis	WP_119139696	--------------K----SP		-----A-------S--
	Pseudomonas resinovorans	WP_077523911	-Y------------K---NSP		-----A----------
	Pseudomonas savastanoi	WP_122396829	--------------K-K-NSP		-----A-------A--
	Pseudomonas sichuanensis	WP_110992500	--------------K----SP		-----A-------S--
	Pseudomonas simiae	MCF5318205	---M----------K-QKNSP		-----A-------A--
	Pseudomonas syringae	MCF5223494	--------------K-K-NSP		-----A-------S--
	Pseudomonas taeanensis	WP_025165616	--------------K----SP		-----A-------S--
	Pseudomonas taiwanensis	WP_179062233	-Y------------K---NSP		-----A----------
	Pseudomonas tehranensis	WP_092397601	---M----------K----SP		-----A-------S--
	Pseudomonas thermotolerans	WP_027897752	--------N-----A----SP		-----A----------
	Pseudomonas ullengensis	WP_183090672	--------------Q---NSP		-----A-------S--

Other *Pseudomonas*	*Pseudomonas urethralis*	WP_176507167	---------------K----SP	-----A--------S--
	Pseudomonas urmiensis	WP_186554736	---------------K----SP	-----A--------S--
	Pseudomonas vanderleydeniana	WP_186677340	---------------K----SP	-----A--------A--
	Pseudomonas viciae	WP_178084302	---M-----------K--K-SP	-----A--------A--
	Pseudomonas viridiflava	WP_122664984	---------------K-K-NSP	-----A--------A--
	Pseudomonas vlassakiae	WP_186602075	---------------K----SP	-----A--------S--
	Pseudomonas weihenstephanensis	WP_048365316	----------L----K-Q--SP	-----A--------S--
	Pseudomonas xantholysinigenes	WP_186655818	---------------K---NSP	-----A--------S--
	Pseudomonas xanthosomae	WP_217888174	---------------K----SP	-----A--------S--
Other Bacteria	*Aeromonas caviae*	GJB81302	---------------K---TSP	-----A--------S--
	Paucimonas lemoignei	SQF97540	---------------K-K--SP	-----A--------A--

Figure-S19

Partial sequence alignments of a protein Osmoprotectant NAGGN system M42 family peptidase showing a 3 aa insertion (highlighted), which is uniquely shared by species from the Oleovorans clade, excepting its presence in *P. pohangensis.* No homologs were found for the species *P. hydrolytica* and *P. indoloxydans*.

Group	Species	Accession	110		150
Straminea Clade *(Phytopseudomonas* gen. nov.) (7/7)	*Pseudomonas argentinensis*	WP_070884112	RFHPELQAAMREAEAATAGAA	GEK	RFILQIAANYGGQWDIA
	Pseudomonas daroniae	WP_131180856	---------------K-S---	---	-----------------
	Pseudomonas dryadis	WP_131177917	---------------K----A	---	-----------------
	Pseudomonas flavescens	WP_084304213	---------------K-----	---	-----------------
	Pseudomonas punonensis	WP_073261673	--------------------S	---	-----------------
	Pseudomonas seleniipraecipitan	WP_070882231	---------------R-S---	---	-L---------------
	Pseudomonas straminea	WP_093503878	--------------------P	--Q	-----------------
Other *Pseudomonas*	*Pseudomonas abietaniphila*	WP_062383139	---------I-----S---NS		--V--------------
	Pseudomonas aeruginosa	WP_110613223	-------------------NT		--L--V-----------
	Pseudomonas agarici	WP_060782213	---------------V---NN		-----------------
	Pseudomonas alcaligenes	WP_021701223	--------------QQ---ES		--V--V-----------
	Pseudomonas alcaliphila	WP_074681813	--------------QL---NR		--T--------------
	Pseudomonas alkylphenolica	WP_038614002	---------------L---SN		-----------------
	Pseudomonas amygdali	WP_044317340	--------------VR-S-NS		--V--------------
	Pseudomonas anguilliseptica	WP_090383233	----------C----Q---DK		--V--V-----------
	Pseudomonas antarctica	WP_064451153	---------------I---SN		--V--------------
	Pseudomonas arsenicoxydans	WP_090187827	---------------M---TN		--V--------------
	Pseudomonas asplenii	WP_121137154	-------------------SN		-----------------
	Pseudomonas avellanae	WP_005615980	--------------VR-S-NS		--V--------------
	Pseudomonas baetica	WP_100847330	---------------M-V--N		--V--------------
	Pseudomonas batumici	WP_040064759	-----------------V-SN		-----------------
	Pseudomonas benzenivorans	WP_090443200	---------------L---EK		-----------------
	Pseudomonas bohemica	WP_110951341	---------I-----R---NT		--V--------------
	Pseudomonas borbori	WP_090500239	---------------M---DK		-----------------
	Pseudomonas brassicacearum	WP_025212115	--------------------D		--V--------------
	Pseudomonas canadensis	WP_028619202	---------------I----N		--V--------------
	Pseudomonas chlororaphis	WP_047736688	--------------------D		--V--------------
	Pseudomonas cichorii	WP_122318716	--------------MQ---NS		--V--------------
	Pseudomonas congelans	WP_096138769	--------------VR-S-NS		--V--------------
	Pseudomonas coronafaciens	WP_122334684	--------------TR-S-NS		--V--------------
	Pseudomonas cremoricolorata	WP_038412340	---------------L---NS		-----V-----------
	Pseudomonas endophytica	WP_055102578	----D---------LM----D		--V--------------
	Pseudomonas entomophila	WP_125859902	-----------------V-SN		-----------------
	Pseudomonas extremaustralis	WP_010563522	---------------I---VG		--V--------------
	Pseudomonas floridensis	WP_083183496	--------------LR---NN		--V--------------
	Pseudomonas fluorescens	AUM68472	--------------------D		--V--------------
	Pseudomonas frederiksbergensis	WP_123405754	---------------I----N		-----------------
	Pseudomonas fuscovaginae	WP_054058442	-------------------SN		-----------------
	Pseudomonas gingeri	WP_017127122	-------------------SN		-----------------
	Pseudomonas grimontii	WP_090408514	---------------I---TN		--V--------------
	Pseudomonas guguanensis	WP_090431219	--------------LL---SR		--T--------------
	Pseudomonas guineae	WP_090238874	--------------VQ---EK		--V--V-----------
	Pseudomonas japonica	WP_042121924	------------V--L---NN		-----V-----------
	Pseudomonas jessenii	WP_110657496	--------------TM----N		--V--------------
	Pseudomonas koreensis	WP_064586183	---------------M----N		-----------------
	Pseudomonas kribbensis	WP_114881638	---------------M---SN		-----------------
	Pseudomonas laurylsulfatovoran	WP_103394424	---------------M-V--N		--V--------------
	Pseudomonas lundensis	WP_047295120	---------------I---SG		--V--V-----------
	Pseudomonas mandelii	WP_094468868	---------------M----N		--V--------------
	Pseudomonas mandelii	AHZ69373	-------V-------M----N		--V--------------
	Pseudomonas mediterranea	WP_047702009	-----------D--------D		--V--------------
	Pseudomonas mendocina	WP_047591990	--------------LQ---GR		-----V-----------
	Pseudomonas migulae	WP_084322274	---------------M----N		--V--------------
	Pseudomonas monteilii	WP_060478347	---------------L---NN		-----------------
	Pseudomonas moorei	WP_090316929	---------------M----N		-----------------
	Pseudomonas mosselii	WP_110738399	-------------------SN		-----------------
	Pseudomonas oleovorans	WP_037053756	--------------LQ---SR		-----------------
	Pseudomonas orientalis	WP_124357342	---------------I---SN		--V--------------
	Pseudomonas parafulva	WP_029613421	---------------L---ND		-----V-----------
	Pseudomonas paralactis	WP_057701314	----D--V-------I---SN		--V--------------
	Pseudomonas peli	WP_090248390	---------------Q---DK		--V--V-----------
	Pseudomonas plecoglossicida	WP_016392308	---------------L---NN		-----------------
	Pseudomonas poae	WP_123714892	---------------M----N		--V--------------
	Pseudomonas prosekii	WP_121731106	---------------I---SN		-----------------
	Pseudomonas protegens	WP_123727654	---------------M---SD		-----------------
	Pseudomonas pseudoalcaligenes	WP_004424015	--------------QL---NR		--T--------------
	Pseudomonas psychrophila	WP_019411590	--------------QM---PD		--V--V-----------
	Pseudomonas putida	WP_110966652	---------------M----N		-----------------
	Pseudomonas reidholzensis	WP_119144998	----G--------------SN		-----V-----------
	Pseudomonas reinekei	WP_075946743	--------------NM----N		--V--------------
	Pseudomonas savastanoi	WP_122235730	--------------VR-S-NS		--V--------------
	Pseudomonas silesiensis	WP_064676103	---------------M---GN		GL---------------
	Pseudomonas simiae	WP_128586887	---------------I---SN		--V--------------
	Pseudomonas soli	WP_023629887	-------------------SN		-----------------
	Pseudomonas syringae	WP_032630771	--------------SS---NN		--V--------------
	Pseudomonas taeanensis	WP_025165205	---------------Q---DK		--V--------------
	Pseudomonas thivervalensis	WP_053118851	--------------T----SN		--V--------------
	Pseudomonas trivialis	WP_049709666	---------------I----D		--V--------------
	Pseudomonas vancouverensis	WP_093214881	---------------M-V--N		-----------------
	Pseudomonas viridiflava	WP_058391731	--------------VR-S-NR		--V--------------
	Pseudomonas wadenswilerensis	WP_115088384	---------------L---SN		-----------------

Group	Species	Accession	Sequence	Sequence
**Other Bacteria**	*Pseudomonas weihenstephanensis*	WP_048363356	--------------TI---PG	--V--------------
	Stenotrophomonas rhizophila	AXQ47080	---------------Q---NN	-----------------
	Priestia aryabhattai	QPN44390	---------------Q---NN	-----------------

Figure- S20

Partial sequence alignments of a protein Di-trans,poly-cis-decaprenylcistransferase showing a 3 aa CSI (insertion; highlighted), which is specific for all species from Straminea clade but not present in other *Pseudomonas* species.

Group	Species	Accession	203		245
**Straminea Clade** *(Phytopseudomonas* **gen. nov.)** **(7/7)**	*Pseudomonas argentinensis*	WP_074886159	AREGERDAVFNVFEALLVEP		GQPLRVSLLDNPEVSASGTVREV
	Pseudomonas daroniae	WP_131191884	------------Y-------		--A---------D---E-K----
	Pseudomonas flavescens	WP_084303700	--------------------		--A---------S---D-Q----
	Pseudomonas punonensis	WP_073263352	--------------------		-----------------------
	Pseudomonas seleniipraecipitan	WP_070882536	----A---------------		--A------E--AI--E-K----
	Pseudomonas straminea	WP_093500791	----A---------------		--AVQ---------T-R-V----
	Pseudomonas dryadis	WP_131176205	--D---------Y-S--AA-	S	--R-Q-T--ED-T---E------
**Other** ***Pseudomonas***	*Pseudomonas abietaniphila*	WP_074752446	--D---------Y-S-F-Q-	PT	D--VE-T-----TIK-T-K----
	Pseudomonas agarici	WP_060783195	--D---------Y-S--E--	PG	DRSIT-T-----TIK-G-K----
	Pseudomonas amygdali	WP_122310672	--D-D-------Y-S-F-Q-	PT	N-SVQ-T-----DIKV--K----
	Pseudomonas antarctica	WP_064450167	--D---------Y-S-----	PP	DA-IT-------S-K-V-K----
	Pseudomonas arsenicoxydans	WP_090177046	--D---------Y-S--T-R	PE	-ET-A-------AIKTT------
	Pseudomonas asplenii	WP_121135871	--D---------Y-S-----	PG	D-TIT-T-----SIQ-T-K----
	Pseudomonas asturiensis	WP_073167551	--D---------Y-S-F-Q-	PG	D--VQ-T-----AIKV--K----
	Pseudomonas avellanae	WP_005621332	--D---------Y-S-F-Q-	PT	D-AVQ-T-----KIKV--K----
	Pseudomonas azotoformans	WP_071493186	--D---------Y-S-----	PP	DA-IT-------SIK-V-K----
	Pseudomonas brassicacearum	WP_123487500	--D---------Y-S--MQ-	PG	QETIHI------K-TTT-K----
	Pseudomonas brenneri	WP_090292678	--D---------Y-S--A--	PP	DA-IT-------SIK-V-R----
	Pseudomonas caricapapayae	WP_122341501	--D-D-------Y-S-F-Q-	PT	N-SVQ-T-----AIKV--K----
	Pseudomonas cedrina	WP_076951555	--D---------Y-S-----	AP	DA-ITI------SIK-V-K----
	Pseudomonas cerasi	WP_065350845	--D-D-------Y-S-F-K-	PT	D-AVQ-T-----TIKV--K----
	Pseudomonas chlororaphis	WP_123328996	--D---------Y-S---D-	PK	DATIS-------SIK-T-----I
	Pseudomonas chlororaphis subsp	AZD58265	--D---------Y-S---A-	PK	EGAIT-------NIKTT-K----
	Pseudomonas cichorii	WP_122317665	--D-----------S-F-Q-	PT	D--VQ-T-----AIKVN-K----
	Pseudomonas cichorii JBC1	AHF65487	--D---------Y-S-F-Q-	PT	D--VQ-T-----AIKV--K----
	Pseudomonas coleopterorum	WP_090361906	--D-D-----D-Y-S-FAQ-	GG	I--IT-----D-AIKTT------
	Pseudomonas congelans	WP_096244558	--D-D-------Y-S-F-K-	PT	D-AVQ-T-----AIKV--K----
	Pseudomonas coronafaciens	KGS15150	--D---------Y-S-F-Q-	PV	N--VQ-T--E--NIKV--K----
	Pseudomonas corrugata	WP_055137036	--D--------IY-S--R-S	PP	DPAIVI------AIQTT-K---I
	Pseudomonas costantinii	WP_071482527	--D---------Y-S-----	PP	DA-IT-------SIK-V-K----
	Pseudomonas deceptionensis	WP_048361425	--D-A-------Y-S-FI--	PK	DPTVQ-T--S--AIKVN-QI--I
	Pseudomonas entomophila	WP_011536268	-VD-D-------Y-----K-	PS	DEGVV-------AIK-E-R---I
	Pseudomonas ficuserectae	WP_054996563	--D-D-------Y-S-F-Q-	PT	N--VQ-T-----DIKV--K----
	Pseudomonas floridensis	WP_083182575	--D---------Y-S-F-Q-	AS	D--VQ-T-----AIKVN-K----
	Pseudomonas fluorescens	WP_078822866	--D---------Y-S-----	PT	DA-IT-------AIT-Q-K----
	Pseudomonas frederiksbergensis	WP_123600073	--D---------Y-S--A--	PS	DKSVV-------AIKTT-----I
	Pseudomonas fuscovaginae	WP_054061989	--D---------Y-S-----	PQ	D--IT-T-----AIK-V-K----
	Pseudomonas gingeri	WP_042935073	--D---------Y-S-----	PD	D--IT-T-----AIK-V-K----
	Pseudomonas grimontii	WP_090401123	-TD---------Y-S-----	PP	DT-ITI------SIK-V-K----
	Pseudomonas kilonensis	WP_053177583	--D--------IY-S--S--	PS	DPAITI------AIKTT-----I
	Pseudomonas libanensis	WP_057010527	--D---------Y-S-----	PS	DA-IT-------SIK-V-K----
	Pseudomonas lundensis	WP_047279619	--D-A-------Y-S-FI--	PK	DPTVQ-T--S--QIKVK-QI--I
	Pseudomonas marginalis	WP_064054165	--D---------Y-S-----	PP	DA-IT-------SIKVQ-K----
	Pseudomonas mediterranea	WP_055129642	--D--------IY-S--S--	PS	DPAIVI------AIQTT-K---I
	Pseudomonas meliae	WP_044345052	--D-D-------Y-S-F-Q-	PT	N--VQ-T-----DIKV--K----
	Pseudomonas mucidolens	WP_084381802	--D-A-------Y----M--	PA	DL-IT-----K-T-K-V-R----
	Pseudomonas orientalis	WP_124431074	--D---------Y-S-----	PP	DA-IT-------SIK-V-K----
	Pseudomonas palleroniana	WP_090367544	--D---------Y-S-----	PP	EA-IT-------SIK-V-K----
	Pseudomonas paralactis	WP_057702377	--D---------Y-S-----	PP	DA-IT-------SIK-V-K----
	Pseudomonas poae	WP_105699297	-QD---------Y-S-----	PP	DA-IT-------SIK-Q-K----
	Pseudomonas protegens	WP_102862623	--D---------Y-S--MQ-	PG	QETIH-------K-TTT-K----
	Pseudomonas proteolytica	WP_092232702	--D---------Y-S--A--	AP	DV-IT-------SIK-V-R----
	Pseudomonas psychrophila	WP_019411805	----A-------Y-S-FI--	PK	DPTVQ-T--SD-TIKVK-QI--I
	Pseudomonas putida	WP_046815362	--D-D-------Y-S--A--	PS	DRAVV-------AIKTT------
	Pseudomonas reinekei	WP_075948590	-QD---------Y-S--A--	PS	DKAVV-------AIKTT-----I
	Pseudomonas rhodesiae	WP_040265269	--D---------Y-S--A--	ST	DT-IT-------SIQVQ-K----
	Pseudomonas saponiphila	WP_092318562	--D---------Y-S--MQ-	SG	QATIHI------A-TTT-K----
	Pseudomonas savastanoi	WP_010215900	--D-D-------Y-S-F-Q-	PT	N--VQ-T-----DIKV--K----
	Pseudomonas silesiensis	WP_064675359	--D---------Y-S--A--	PT	DKSVV----E--AIKTT------
	Pseudomonas simiae	WP_106119013	--D---------Y-S-----	PP	DA-IT-------SIK-V-K----
	Pseudomonas soli	WP_110697192	-VD-D-------Y-S---A-	SG	DKGVT---I---AIT-Q-H---I
	Pseudomonas synxantha	WP_005783768	--D---------Y-S-----	PP	DA-IT----E--SIK-V-K----
	Pseudomonas syringae	WP_122299811	--D---------Y-S-F-Q-	PT	D-AVQ-T-----AIKV--K----
	Pseudomonas thivervalensis	WP_053117229	--D--------IY-S--S--	PS	DPAITI------AIKTT-----I
	Pseudomonas tolaasii	WP_016972160	--D---------Y-S-----	PP	DA-IT-------SIK-V-K----
	Pseudomonas trivialis	WP_049710616	--D---------Y-S-----	PP	DA-IT-------SIK-V-K----
	Pseudomonas veronii 1YdBTEX2	SBW78170	----------D-Y-S-----	PA	DA-IT-------SIKVQ-H----
	Pseudomonas versuta	WP_073509948	--D-A-------Y-S-FI--	PK	DPTVQ-T--S--AIKVN-QI--I
	Pseudomonas viridiflava	WP_122210727	--D---------Y-S-F-Q-	AS	D--VQ-T-----AIKVL-K----
	Pseudomonas vranovensis	WP_123565210	--D---------Y-----A-	AT	REAIK---V---KIQ-Q-YI---
	Pseudomonas yamanorum	WP_063033608	--D---------Y-S-----	PP	DK-IT----E--QIT-V-K----
**Other Bacteria**	*Bacillus sp. TH86*	MBK5303075	--D---------Y-S--A--	PS	DRSIV-------KIKTT------
	Escherichia coli	MRF42492	-TD-D-------Y-S--VA-	AS	DAGVI-----D-K-Q-R-F---I
	Priestia aryabhattai	QPN45761	--D-D-------Y-S--VA-	PS	DAGVM-----D-K-Q-Q-F---I
	Rhodococcus equi	NKZ99195	--D---------Y-S--AGT	TS	DASVV-----D-K-Q-R-A---I
	Stenotrophomonas rhizophila	AXQ46342	--D-D-------Y-S--VA-	PS	DAGVI-----D-K-Q-H-F---I
	Tanacetum cinerariifolium	GEW38167	--D---------Y-S-FVN-	PT	DQPVD-------NIKAT-K----

Figure- S21

Partial sequence alignments of a protein Efflux RND transporter periplasmic adaptor subunit showing a 2 aa deletion (highlighted), which is specific for all species from Straminea clade but not present in other *Pseudomonas* species. The species *P. drysdis* constains 1 aa deletion instead of two.

Group	Species	Accession	233		273
Straminea Clade (*Phytopseudomonas* gen. nov.) (7/7)	*Pseudomonas straminea*	WP_093501944	SNNIRNNFGFLNRAAEESA	Q	TSRDKLFVQEGRKVFKEVCP
	Pseudomonas argentinensis	WP_070886775	---------------D-AG	A	VAEGR---------------
	Pseudomonas dryadis	WP_131174870	---------------QDGS	-	GT-------------RD---
	Pseudomonas daroniae	WP_131178456	----------------NGS	-	GAN-------------D---
	Pseudomonas flavescens	WP_084305643	---------------QDGS	-	AAG-----------------
	Pseudomonas punonensis	WP_073264396	---------------QDGN	-	AAG-----------------
	Pseudomonas seleniipraecipitans	WP_070881932	-----------------GG	P	LAEGR---------------
Other *Pseudomonas*	*Pseudomonas aeruginosa*	MBG6495724	----------------SGI		GK--------------D---
	Pseudomonas alcaligenes	TXI33368	-----------------GI		GAK-------------D---
	Pseudomonas alcaliphila	WP_074676732	-----------------GV		GA--------------D---
	Pseudomonas alvandae	WP_217860105	-------Y---------GI		GT-------------RD---
	Pseudomonas arsenicoxydans	WP_208671760	-----------------GI		GTK------------RD---
	Pseudomonas baetica	WP_100847558	-----------------GI		GT--------------D---
	Pseudomonas bananamidigenes	WP_065258264	-----------------GI		GA--------------D---
	Pseudomonas brassicacearum	WP_025212413	-----------------GI		G--------------RD---
	Pseudomonas brassicacearum	WP_230151103	---------------Q-GV		G--------------RD---
	Pseudomonas carbonaria	WP_187672025	-----------------GI		GT--------------D---
	Pseudomonas cavernae	WP_119894433	-----------------GI		GA--------------D---
	Pseudomonas chengduensis	WP_031306476	-----------------GI		GA--------------D---
	Pseudomonas chlororaphis	WP_038358456	-----------------GV		GA--------------D---
	Pseudomonas citronellolis	WP_061563214	-----------------GI		GQ--------------D---
	Pseudomonas composti	WP_061240404	-----------------GV		GA--------------D---
	Pseudomonas composti	WP_074941193	---------------Q-G-		GA--------------D---
	Pseudomonas corrugata	WP_024780430	-----------------GI		GT-------------RD---
	Pseudomonas delhiensis	WP_089391907	-----------------GI		GQ--------------D---
	Pseudomonas ekonensis	WP_217893231	-----------------GV		GA--------------D---
	Pseudomonas fluorescens	WP_043042757	---------------Q-G-		G--------------RD---
	Pseudomonas frederiksbergensis	WP_039593357	-----------------GI		GT-------------RD---
	Pseudomonas frederiksbergensis	WP_071551381	-----------------GV		GA-------------RD---
	Pseudomonas granadensis	WP_203421136	-----------------GI		GT--------------D---
	Pseudomonas gregormendelii	WP_205891897	-----------------GI		GA-------------RD---
	Pseudomonas guguanensis	WP_090426869	-----------------GI		GA--------------D---
	Pseudomonas hydrolytica	MCF2123150	-----------------GI		GA--------------D---
	Pseudomonas jinjuensis	WP_084313804	-----------------GI		GT------------------
	Pseudomonas khorasanensis	WP_186532149	-----------------GI		GT--------------D---
	Pseudomonas kilonensis	WP_024618557	-----------------G-		G--------------RD---
	Pseudomonas knackmussii	WP_043257037	-----------------GI		GQ--------------D---
	Pseudomonas lalkuanensis	WP_151132392	-----------------GV		GA--------------D---
	Pseudomonas mangiferae	WP_143488823	-----------------GP		GTD-------------D---
	Pseudomonas marincola	WP_090513599	-----------------GQ		GA--------------D---
	Pseudomonas mediterranea	WP_055134786	-----------------GI		GT-------------RD---
	Pseudomonas mendocina	WP_047589477	-----------------GV		GT--------------D---
	Pseudomonas moorei	WP_090317609	-----------------GI		GA-------------RD---
	Pseudomonas multiresinivorans	WP_169937211	-----------------GI		GT--------------D---
	Pseudomonas nicosulfuronedens	WP_138525098	-----------------GI		G---------------D---
	Pseudomonas nitritireducens	WP_221454957	-----------------GI		G---------------D---
	Pseudomonas nitroreducens	WP_205346023	-----------------GI		GT--------------D---
	Pseudomonas ogarae	WP_079305174	-----------------G-		G--------------RD---
	Pseudomonas oleovorans	WP_119693386	-----------------G-		GA--------------D---
	Pseudomonas otitidis	WP_182852085	-----------------GV		GA--------------D---
	Pseudomonas panipatensis	WP_090261137	-----------------GI		GQ--------------D---
	Pseudomonas piscis	WP_152897334	-------------T---GI		GA-------------RD---
	Pseudomonas prosekii	WP_092271655	-----------------GV		G-K------------RD---
	Pseudomonas protegens	WP_041116569	-----------------GI		GT-------------RD---
	Pseudomonas putida	WP_065861460	-----------------GE		GT------------------
	Pseudomonas reidholzensis	WP_119143769	-----------------GE		GTA------------R----
	Pseudomonas resinovorans	WP_077527842	-----------------GV		GA--------------D---
	Pseudomonas schmalbachii	WP_236032697	-----------------GI		GT------------------
	Pseudomonas sediminis	WP_099526522	-----------------GV		GA--------------D---
	Pseudomonas segetis	WP_089359267	-----------------GI		GA--------------D---
	Pseudomonas sihuiensis	WP_092380294	-----------------GV		GA--------------D---
	Pseudomonas syringae	WP_236485459	-----------------GI		GT--------------D---
	Pseudomonas taiwanensis	WP_218649422	-----------------GM		GA--------------D---
	Pseudomonas thivervalensis	WP_053120134	-------------T-Q-GI		G--------------RD---
	Pseudomonas tohonis	WP_173176421	-----------------GV		GA-------------RD---
	Pseudomonas toyotomiensis	WP_206417508	-----------------GI		GA--------------D---
	Pseudomonas urmiensis	MBV4535038	-----------------GE		GAA------------R----
	Pseudomonas uvaldensis	WP_232775504	-----------------GI		GT-------------RD---
	Pseudomonas viridiflava	WP_122566123	---------------Q-GV		G--------------RD---
	Pseudomonas wayambapalatensis	MBC3426145	-----------------GE		GT------------------
	Pseudomonas yangonensis	WP_161866620	-----------------GV		GA--------------D---
	Pseudomonas zanjanensis	WP_186704591	-------------T---GM		G--------------RD---
Other Bacteria	*Priestia aryabhattai*	QPN44179	-----------------GV		GT------------------
	Salinicola salarius	WP_110635064	--A----------L-D---		QTL-----------------
	Stenotrophomonas maltophilia	WP_204275055	--------------D-SGI		GK--------------D---

Figure- S22

Partial sequence alignments of a protein Beta-ketoacyl-ACP synthase III showing a 1aa insertion (highlighted), which is specific for all species from Straminea clade but not present in other *Pseudomonas* species.

Group	Species	Accession	26–65
Straminea Clade (*Phytopseudomonas* gen. nov.) (6/6)	*Pseudomonas straminea*	WP_093502557	RWLAKRDIVEALPTTCGRS GMAYAIAPGASRVVESAERLP
	Pseudomonas argentinensis	WP_070885551	------------------- ---------------A-GQ--
	Pseudomonas daroniae	WP_131181803	---------A--------- ---------------F-D---
	Pseudomonas flavescens	WP_084304853	---------A--------- A--------------F-G---
	Pseudomonas punonensis	WP_073265539	------------------- -------R-------A--W--
	Pseudomonas seleniipraecipitan	WP_092365268	------------------- ---------------------
Other *Pseudomonas*	*Pseudomonas abietaniphila*	WP_062383443	---V-L-V--ESL------ FN R---------R-F--NPDA--
	Pseudomonas alcalimarina	WP_041013213	----E-R-----------G VG --G----S--RS--QHPD-M-
	Pseudomonas alcaliphila	WP_074682426	---V--E----------QG GN ------G---R-IALRP-L--
	Pseudomonas alliivorans	WP_210070098	---I-LNV--PELS----T GN K----V----R---QNP-A--
	Pseudomonas amygdali	WP_044323876	---V-L-V--PEL-----T YN K-V--V----R---KHPDA--
	Pseudomonas anguilliseptica	WP_233683959	---V-QN-I-EQL-----T FK H-G-G--E--R----RP-L--
	Pseudomonas asturiensis	WP_024685908	---I-LNV--PEL-----A GN K----V----R---QNPDA--
	Pseudomonas avellanae	WP_005615795	---V-L-V--PEL-----T YN K----V----RK--KNP-A--
	Pseudomonas azotifigens	WP_181069916	----H-N---TV------T GN R--H-L----RQ-AQHPA---
	Pseudomonas bohemica	WP_110949974	---V-LGV--QNL------ FN R---------R-F--NPDA--
	Pseudomonas campi	WP_173209152	----QLNVI-EQ-S----- FN R---------R----LPQL--
	Pseudomonas cannabina	WP_055001799	---V-LNV--PEL-----A YN K----V----RA--KKPAA--
	Pseudomonas caricapapayae	WP_055007841	---V-L-M--PEL-----T YN K----V----K---KKPDA--
	Pseudomonas caspiana	WP_087264551	---V-LNV--PEL-----T YN K---------RA---NPDA--
	Pseudomonas chengduensis	WP_031306550	---V--E----------QG GN ------GS--R-IAQRP-L--
	Pseudomonas chloritidismutans	WP_023446528	----E-R-----------G VG --G----S--RS--QHP----
	Pseudomonas cichorii	WP_198726933	---V-LNV--PELS----T YN K---------R---QNPDA--
	Pseudomonas composti	WP_061241631	---V--E----------QG GN ------GA--R-IAQHS-L--
	Pseudomonas congelans	WP_032613723	---V-LNV--PEL-----T YN K----V----K---KNPDA--
	Pseudomonas coronafaciens	WP_055003359	---V-L-V--PEL-----T HN K----V----C---KNPDA--
	Pseudomonas duriflava	WP_145138757	---IH----QEQV-----G SR ----G-----E---HHPDQ--
	Pseudomonas floridensis	WP_083182849	---V-LNV--PEL-----A GN K----V----R---QNPDA--
	Pseudomonas fragi	NNG64505	---VRKN-I-QELS----G GS R---------AS--LHP-A--
	Pseudomonas graminis	HEF25360	---V-LNV--ESL------ FN R----V----R-F--NPDA--
	Pseudomonas guguanensis	WP_090432008	---V--E----------QG GN ------G---R-IAQHP-L--
	Pseudomonas guryensis	WP_182831866	----QLNVI-EQ-S----- FN R---------R----QPQL--
	Pseudomonas kunmingensis	NYF95910	----E-R-----------G VG --G----S--RS--QHP----
	Pseudomonas kuykendallii	WP_090227154	---VRL-V-AE------LG GA R-----GE--R----HP----
	Pseudomonas lalkuanensis	WP_151132063	----Q-EV-----S---LR GN R------R--RS---HP----
	Pseudomonas linyingensis	WP_090310630	N--V--E------S---QG GN -------R--Q----HP-L--
	Pseudomonas lopnurgenes	WP_187807511	---I-------Q-S----G AR ----------RA---HP----
	Pseudomonas lutea	KGF66530	---V-LNV--ESL------ FN R---TV----R----NPDA--
	Pseudomonas mendocina	WP_143505921	---V--E----------QG GN ------G---R-IARRP-L--
	Pseudomonas nosocomialis	WP_138406617	----Q-NV---V------T GN R--H-L----RQ-ARHPD---
	Pseudomonas oleovorans	PZQ39540	---V--E--A-------QG GN ------G---R-IAQRP-L--
	Pseudomonas oryzae	WP_090349414	N--VR-E--A-------QG GN ----------R----HP----
	Pseudomonas resinovorans	WP_077522609	----R-EV-----S---LR GN R------A--RE---NP----
	Pseudomonas savastanoi	EGH08910	---V-L-V--PEL-----T YN K----V----R---KHPDA--
	Pseudomonas sediminis	WP_099526548	---V--E----------QG GN ------G---R-IALRPDL--
	Pseudomonas segetis	WP_089358637	---VQQN-I--QM-----T FK K---------R----HP-L--
	Pseudomonas sihuiensis	WP_092377220	---V--E----------QG GN ------G---R-IAQRPDL--
	Pseudomonas songnenensis	WP_126190197	----E-R-----------G VG --G----S--RN--QHP----
	Pseudomonas stutzeri	WP_138300172	----E-R-----------G AR ----------R---QYP----
	Pseudomonas syringae	WP_103695997	---V-L-V--PEL-----T YN K----V----K---KNPDA--
	Pseudomonas taiwanensis	WP_179061685	----R-EV-----S---LR GN R------A--RE---NP----
	Pseudomonas toyotomiensis	WP_059392966	---V--E----------QG GN ------G---R-IAQRPDL--
	Pseudomonas triticumensis	WP_187518123	---I-LNV--PEL-----A GN K----V----R---QNPDA--
	Pseudomonas ullengensis	WP_183089147	---V--N---EQ------G GR ----------RQI-QRP-L--
	Pseudomonas viridiflava	WP_122433007	---I-LNV--PEL-----A GN K----V----R---Q-P-A--
	Pseudomonas wenzhouensis	WP_230925242	---V-----A-------QG GN ------G---RHIAQRP-L--
	Pseudomonas xanthoensis	WP_193682453	----E-R-----------G AS ----------RN--LRP-L--
	Pseudomonas xionganensis	WP_160344068	---V-QN-IQEQL-----T YK H-G-G--E--R----RP-L--
	Pseudomonas yangonensis	WP_161867585	---V--E----------QG GN ------G---R-IAQRP-L--
	Pseudomonas zhaodongensis	WP_122164449	----E-RVI---------G VG --G--V-Q--RN--KRP----
Other Bacteria	*Tanacetum cinerariifolium*	GEZ60859	---V-LNV--ESL------ FN R---------R----KPDA--

Figure- S23

Partial sequence alignments of a protein Sugar ABC transporter ATPase showing a 2 aa deletion (highlighted), which is specific for the species from Straminea clade. No homolog was found for the ingroup species *P. dryadis*, which is a deep-branching species in the clade.

Group	Species	Accession	818		855
**Straminea Clade** ***(Phytopseudomonas*** **gen. nov.) (6/7)**	*Pseudomonas straminea*	WP_093503860	PDVNASEFKFTVSHDA	NQTQ	RILYGLGAVKGVGEGPVE
	Pseudomonas argentinensis	WP_070884657	------------TR--	AGGE	------------------
	Pseudomonas daroniae	WP_131190293	----------------	-NVP	------------------
	Pseudomonas flavescens	WP_084304203	----------------	-NVP	------------------
	Pseudomonas punonensis	WP_073261653	-------------YE-	D VP	------------------
	Pseudomonas seleniipraecipitan	WP_070882335	-------Y--------	-NVP	------------------
	Pseudomonas dryadis	WP_131177985	----T-------ND-G		--V---------------
**Other** ***Pseudomonas***	*Pseudomonas alcaliphila*	WP_074681793	----V-------ND-G		--V---------------
	Pseudomonas alliivorans	WP_210070127	----T--------D-G		--------I---------
	Pseudomonas amygdali	WP_054077560	----T--------D-G		--------I---------
	Pseudomonas avellanae	WP_024698532	----T--------D-G		--------I---------
	Pseudomonas cannabina	KPW68157	----T--------D-G		--------I---------
	Pseudomonas carbonaria	WP_187671614	----I-------ND-G		--V---------------
	Pseudomonas caricapapayae	KPW55207	----T--------D-G		--------I---------
	Pseudomonas chengduensis	WP_021489802	----V-------ND-G		--V---------------
	Pseudomonas chloritidismutans	WP_228207284	----I-------ND-G		--V---------------
	Pseudomonas composti	NYG62220	----V-------ND-G		--V---------------
	Pseudomonas congelans	KPW83030	----T--------D-G		--------I---------
	Pseudomonas coronafaciens	WP_117216095	----T--------D-G		--------I---------
	Pseudomonas ficuserectae	RMS34133	----T--------D-G		--------I---------
	Pseudomonas floridensis	WP_083183487	----T--------D-G		--------I---------
	Pseudomonas fluorescens	WP_154971979	----T--------D-G		--------I---------
	Pseudomonas guguanensis	WP_090431236	----V-------ND-G		--V---------------
	Pseudomonas hydrolytica	MCF2123535	----V-------ND-G		--V---------------
	Pseudomonas indoloxydans	WP_108233761	----V-----S-ND-G		--V---------------
	Pseudomonas khazarica	WP_134677474	----V-------ND-G		--V---------------
	Pseudomonas khazarica	WP_210389439	----V-------ND-G		--V---------------
	Pseudomonas kunmingensis	NYF93247	----I-------ND-G		--V---------------
	Pseudomonas lopnurensis	WP_193679712	----V-------ND-G		--V---------------
	Pseudomonas meliae	KPX88884	----T--------D-G		--------I---------
	Pseudomonas mendocina	WP_147809593	----V-------ND-G		--V---------------
	Pseudomonas nitritireducens	WP_213909278	----V-------ND-G		--V---------------
	Pseudomonas oleovorans	PZR30108	----V-----S-ND-G		--V---------------
	Pseudomonas savastanoi	WP_057456222	----T--------D-G		--------I---------
	Pseudomonas sediminis	WP_099523459	----V-------ND-G		--V---------------
	Pseudomonas sihuiensis	WP_092376594	----V-------ND-G		--V---------------
	Pseudomonas songnenensis	WP_122099759	----V-------ND-G		--V---------------
	Pseudomonas stutzeri	WP_213663128	----V-------ND-G		--V---------------
	Pseudomonas syringae	WP_003377823	----T--------Y-G		--------I---------
	Pseudomonas toyotomiensis	WP_059391338	----V-------ND-G		--V---------------
	Pseudomonas tremae	WP_236534908	----T--------D-G		--------I---------
	Pseudomonas viridiflava	WP_099229710	----T--------D-G		--------I---------
	Pseudomonas wenzhouensis	WP_230927212	----V-------ND-G		--V---------------
	Pseudomonas xanthomarina	WP_041013557	----I-------ND-G		--V---------------
	Pseudomonas yangonensis	WP_161867641	----V-------ND-G		--V---------------
**Other Bacteria**	*Croceibacter atlanticus*	WP_219360707	------------NDEG		--I-----I---------
	Entomomonas moraniae	WP_127164536	------------GD-Q		--I-----I---------
	Escherichia coli	HAL1390276	------------NDEG		--I-----I---------
	Paucimonas lemoignei	SQF97377	----I--------D-G		--------I---------
	Streptococcus pneumoniae	CJK44307	----V-------ND-G		--V---------------

Figure- S24

Partial sequence alignments of a protein DNA polymerase III subunit alpha showing a 4 aa insertion (highlighted), which is specific for all species from Straminea clade except *P. dryadis*, which is a deep-branching species in the clade.

Group	Species	Accession	110	Ins	153
**Straminea Clade (*Phytopseudomonas* gen. nov.) (6/7)**	*Pseudomonas straminea*	WP_093503878	RFHPELQAAMREAEAATAGA	PGE	QRFILQIAANYGGQWDIAQAA
	Pseudomonas argentinensis	WP_070884112	--------------------	A--	K--------------------
	Pseudomonas daroniae	WP_131180856	---------------K-S--	A--	K--------------------
	Pseudomonas flavescens	WP_179537512	--------------SR-S-G	A--	K--------------------
	Pseudomonas punonensis	WP_073261673	--------------------	S--	K--------------------
	Pseudomonas seleniipraecipitan	WP_092365868	---------------R-S--	A--	K-L------------------
	Pseudomonas dryadis	WP_131177917	---------------K---E		K--------------------
**Other *Pseudomonas***	*Pseudomonas aeruginosa*	WP_134517482	-------------------N		T--L--V----------V---
	Pseudomonas aestus	ERO62051	---------------I---S		D--------------------
	Pseudomonas agarici	WP_060782213	---------------V---N		N--------------------
	Pseudomonas alkylphenolica	WP_038614002	---------------L---S		N--------------------
	Pseudomonas allokribbensis	WP_192559903	---------------M----		N--V-----------------
	Pseudomonas arsenicoxydans	WP_090187827	---------------M---T		N--V-----------------
	Pseudomonas atagonensis	WP_166223173	---------------M-V--		N--V-----------------
	Pseudomonas aylmerensis	WP_065903084	---------------I---V		N--V-----------------
	Pseudomonas baetica	WP_100847330	---------------M-V--		N--V-----------------
	Pseudomonas bananamidigenes	WP_065259189	---------------M---S		N--------------------
	Pseudomonas batumici	WP_040064759	-----------------V-S		N--------------------
	Pseudomonas benzenivorans	WP_090443200	---------------L---E		K--------------------
	Pseudomonas borbori	WP_090500239	---------------M---D		K--------------------
	Pseudomonas botevensis	WP_217832636	---------------M----		N--V-----------------
	Pseudomonas brassicacearum	WP_181289174	--------------S-----		D--V-----------------
	Pseudomonas campi	WP_173204182	--------------Q----E		N--V--V--------------
	Pseudomonas canadensis	WP_028619202	---------------I----		N--V-----------------
	Pseudomonas capeferrum	WP_181129006	---------------L---S		N-----V--------------
	Pseudomonas chlororaphis	WP_016705307	---------------M---S		N--------------------
	Pseudomonas costantinii	WP_177012235	---------------I---T		N--V-----------------
	Pseudomonas cremoricolorata	WP_028696805	---------------L---N		R-----V--------------
	Pseudomonas cremoris	WP_185707398	---------------I---T		N--V-----------------
	Pseudomonas donghuensis	MBF4207922	---------------L----		N--------------------
	Pseudomonas dryadis	WP_131177917	---------------K---E		K--------------------
	Pseudomonas ekonensis	WP_217893455	---------------M----		N--V-----------------
	Pseudomonas entomophila	WP_011535276	---------------L---N		N--------------------
	Pseudomonas fildesensis	WP_048719945	-------T-------I----		N--V-----------------
	Pseudomonas fluorescens	MBI3906723	---------------M----		N--V-----------------
	Pseudomonas frederiksbergensis	WP_074879695	---------------M----		N--V-----------------
	Pseudomonas fuscovaginae	WP_017906272	-------------------S		N--------------------
	Pseudomonas gessardii	WP_197880986	---------------I---S		N--V-----------------
	Pseudomonas gingeri	WP_177092974	-------------------S		N--------------------
	Pseudomonas grimontii	WP_090408514	---------------I---T		N--V-----------------
	Pseudomonas guineae	SFH82945	--------------VQ---E		K--V--V--------------
	Pseudomonas guryensis	WP_182834004	--------------Q----E		N--V--V--------------
	Pseudomonas izuensis	WP_160106802	---------------M----		N--V-----------------
	Pseudomonas koreensis	WP_150370366	---------------M----		N--------------------
	Pseudomonas kribbensis	WP_114881638	---------------M---S		N--------------------
	Pseudomonas lactis	WP_236313393	-------T-------I---S		N--V-----------------
	Pseudomonas laurylsulfatiphila	WP_104450680	---------------M----		N--V-----------------
	Pseudomonas laurylsulfativoran	WP_103394424	---------------M-V--		N--V-----------------
	Pseudomonas leptonychotis	WP_136662669	--------------TQ---D		K-----V--------------
	Pseudomonas lurida	WP_192095508	---------------I----		D--V-----------------
	Pseudomonas mandelii JR-1	AHZ69373	-------V-------M----		N--V-----------------
	Pseudomonas mediterranea	WP_047702009	-----------D--------		D--V-----------------
	Pseudomonas moorei	WP_090316929	---------------M----		N--------------------
	Pseudomonas mosselii	WP_062361392	-----------------V-N		N--------------------
	Pseudomonas ogarae	WP_014336792	-------------------S		D--V-----------------
	Pseudomonas orientalis	WP_124357342	---------------I---S		N--V-----------------
	Pseudomonas oryzicola	WP_186679217	---------------L---N		N--------------------
	Pseudomonas oryziphila	WP_125859902	-----------------V-S		N--------------------
	Pseudomonas palleroniana	WP_198762321	---------------M----		N--V-----------------
	Pseudomonas plecoglossicida	WP_028625232	---------------L---N		N--------------------
	Pseudomonas poae	WP_060548630	---------------I----		D--V-----------------
	Pseudomonas prosekii	WP_121731106	---------------I---S		N--------------------
	Pseudomonas protegens	WP_123727654	---------------M---S		D--------------------
	Pseudomonas proteolytica	WP_169904915	---------------I---S		N--V-----------------
	Pseudomonas putida	WP_046816513	---------------M----		N--V-----------------
	Pseudomonas reactans	WP_177003306	---------------I----		N--V-----------------
	Pseudomonas rhodesiae	WP_198712580	---------------I----		D--V-----------------
	Pseudomonas sichuanensis	WP_110992420	-------------------S		N--------------------
	Pseudomonas simiae	OAE14043	---------------I---S		N--V-----------------
	Pseudomonas soli	WP_023629887	-------------------S		N--------------------
	Pseudomonas taeanensis	WP_025165205	---------------Q---D		K--V-----------------
	Pseudomonas thivervalensis	WP_053118851	--------------T----S		N--V-----------------
	Pseudomonas trivialis	WP_049709666	---------------I----		D--V-----------------
	Pseudomonas tructae	WP_130263456	---------------L----		N--------------------
	Pseudomonas urmiensis	WP_186555434	-------------------S		N-----V--------------
	Pseudomonas vancouverensis	WP_093214881	---------------M-V--		N--------------------
	Pseudomonas vanderleydeniana	WP_186682122	-------------------S		N--------------------
	Pseudomonas veronii	WP_232591346	---------------I---S		N--V-----------------
	Pseudomonas viciae	WP_135843919	-------------------T		D--V-----------------
	Pseudomonas viridiflava	WP_116628370	---------------I----		N--V-----------------
	Pseudomonas xantholysinigenes	WP_186660047	---------------I---N		N--------------------
	Pseudomonas xionganensis	WP_160346584	---------------Q---D		K-----V--------------
	Pseudomonas yamanorum	WP_177042013	---------------I---S		D--V-----------------
	Pseudomonas zeae	WP_186622028	---------------M----		D--------------------
**Other Bacteria**	*Escherichia coli*	MRF43523	---------------L---N		N--------------------
	Priestia aryabhattai	QPN44390	---------------Q---N		N--------------------
	Stenotrophomonas rhizophila	AXQ47080	---------------L---S		N--------------------

Figure- S25

Partial sequence alignments of a protein Polyprenyl diphosphate synthase showing a 3aa Ins (highlighted), which is specific for all species from Straminea clade except *P. dryadis*, which is a deep-branching species in the clade.

Group	Species	Accession	65		102
Straminea Clade (*Phytopseudomonas* gen. nov.) (6/7)	*Pseudomonas straminea*	SFD97069	EGAFASVEGLMRDVR	TGDTP	HGAILRYMHSTGASAFF
	Pseudomonas argentinensis	WP_070887234	-A-------I-----	--N--	-----------------
	Pseudomonas daroniae	WP_131180211	---------I-----	-----	-----------------
	Pseudomonas flavescens	SDI03473	---------------	-----	-----------------
	Pseudomonas punonensis	WP_073268058	---------------	-----	-----------------
	Pseudomonas seleniipraecipitan	WP_070882729	-A-------I-----	-----	-----------------
	Pseudomonas dryadis	WP_131175438	-E------YI----E		Y-W--------------
Other *Pseudomonas*	*Pseudomonas aeruginosa*	KAB0758612	-E------YI----D		Y-W-I------------
	Pseudomonas caspiana	WP_087264863	-E-------I----P		Y-S---LL---------
	Pseudomonas entomophila	WP_181092684	------I-SI----E		Y--L---L---------
	Pseudomonas floridensis	WP_083182589	------I-YI----E		Y-----LL---------
	Pseudomonas foliumensis	WP_187520902	-E-------I----P		Y-S---LL---------
	Pseudomonas fulva	WP_196181304	-E------YI----E		Y-W----L---------
	Pseudomonas graminis	WP_172609583	--------SI----E		Y-S---LL---------
	Pseudomonas mangrovi	WP_108104425	D-------YI----E		Y---I--L---------
	Pseudomonas monteilii	AMA47109	--------SI----E		Y--L---L---------
	Pseudomonas oleovorans	PZQ25676	--------YI----E		Y-W----L---------
	Pseudomonas palleroniana	WP_151153041	-E------YI----E		Y-S---LL---------
	Pseudomonas savastanoi	WP_122394956	-E-------I----P		Y-S---LL---------
	Pseudomonas stutzeri	WP_038657351	--------YI----E		Y-W----L---------
	Pseudomonas syringae	WP_027900226	-E-------I----P		Y-S---LL---------
	Pseudomonas viridiflava	WP_122535924	--------SI----E		Y-S---LL---------
Other Bacteria	*Aeromonas australiensis*	WP_040096310	------I-YI----E		Y-WL-------------
	Aeromonas bivalvium	WP_042001529	--------YI----D		Y-WL-------------
	Aeromonas veronii	WP_064335277	--------YI----E		Y-WL-------------
	Agaribacterium haliotis	WP_096085241	--------YI----P		Y-W-I--L---------
	Algibacillus agarilyticus	WP_111978273	--------YI----E		Y-WL-------------
	Alteromonadaceae bacterium	MAA66729	--------YI----Q		W-WL-------------
	Amphritea atlantica	WP_091362069	--------YI----D		Y-WL-------------
	Cellvibrio mixtus	WP_039918249	--------YI----D		F-WL-------------
	Cellvibrio zantedeschiae	WP_189415738	--------YI----D		Y-WL-------------
	Enterovibrio baiacu	WP_129494462	------I-YI----E		Y-WL-------------
	Enterovibrio norvegicus	WP_016960768	--------YI----E		Y-WL-------------
	Francisellaceae bacterium	MBT6206436	--------FI---LP		M-WL----------F--
	Gallaecimonas xiamenensis	WP_008485246	--------YI----D		Y-WL---L---------
	Grimontia celer	WP_062665984	------I-YI----E		Y-WL-------------
	Hahella ganghwensis	WP_020409293	------I-YI----E		Y-WL---L---------
	Halieaceae bacterium	HBQ42206	-Q------YI----D		Y-W--------------
	Halomonas anticariensis	WP_016414568	--------YI----E		Y-WLI------------
	Halomonas saliphila	WP_104201790	--------YI----E		W-WLI------------
	Halomonas urmiana	WP_138181538	--------YI----E		W-WLI------------
	Klebsiella pneumoniae	WP_228680857	-E------YI----D		Y-W-I------------
	Mangrovitalea sediminis	WP_097460968	-------QYI----Q		W-WLI--L---------
	Maribrevibacterium harenarium	WP_140588238	--------YI----E		Y-WLI------------
	Marinimicrobium koreense	WP_123636898	--------YI----E		Y-W--------------
	Marinobacter adhaerens	HBF93710	--------YI----E		W-WL---L---------
	Marinobacterium stanieri	WP_010321798	--------YI----E		Y-WL---L---------
	Marinobacterium zhoushanense	WP_188746334	--------YI----E		Y-WL---L---------
	Marinomonas aquimarina	SBS27313	--------YI----E		Y-WLI------------
	Motiliproteus sediminis	WP_210396724	------I-YI----E		Y-WL---L---------
	Neptunicella marina	WP_186505739	--------YI--E-D		Y-Y--------------
	Nitrincola alkalilacustris	WP_151703871	------I-YI----E		Y-WL-------------
	Nitrincola iocasae	WP_151053800	------I-YI----E		Y-WL-------------
	Oceanospirillum beijerinckii	WP_051228113	--------YI----E		Y-WL-------------
	Oceanospirillum linum	WP_077242876	--------YI----E		Y-WL-------------
	Oleiphilus messinensis	ARU58709	------I-YI----E		Y-WL---L---------
	Paraglaciecola aquimarina	WP_235313464	--------YI----E		Y-WL-------------
	Parahaliea mediterranea	WP_116368583	--------YI----D		Y-W--------------
	Paraperlucidibaca baekdonensis	WP_116208148	----D---YI----E		Y-WL-------------
	Pistricoccus aurantiacus	WP_147185312	--------YI----E		Y-WLI--------T---
	Pleionea mediterranea	WP_109764509	-N------YI----P		Y-WL-------------
	Pleionea sediminis	WP_144394041	--------YI----P		Y-WL-------------
	Pokkaliibacter plantistimulans	WP_110186909	--------YI----D		M-W-I--L---------
	Pseudomonadales bacterium	MCC6530136	--------YI----E		W-WL-------------
	Pseudospirillum japonicum	WP_093308209	--------YI----E		Y-WLI------------
	Psychromonas antarctica	WP_238740249	------I-YI----P		Y-WL-------------
	Psychromonas ossibalaenae	WP_019615810	------I-YI----P		Y-WL-------------
	Salinivibrio socompensis	WP_025674465	--------YI----E		Y-WL-------------
	Stenotrophomonas maltophilia	WP_204273223	-E------YI----D		Y-W-I------------
	Terasakiispira papahanaumokuak	WP_068997081	--------YI----N		Y-WL-------------
	Thiopseudomonas alkaliphila	WP_053100178	--------YI----E		Y-W----L---------
	Thiopseudomonas denitrificans	WP_101497803	--------YI----E		Y-W----L---------
	Veronia nyctiphanis	WP_129123514	------I-YI----E		Y-WL-------------
	Vibrio parahaemolyticus	TOP17414	------I-YI----E		Y-WL-------------

Figure- S26

Partial sequence alignments of a protein Ubiquinol-cytochrome c reductase cytochrome b subunit showing a 5aa Ins (highlighted), which is specific for the species from Straminea clade except in the deep-branching *P. dryadis*.

Group	Species	Accession	108		150
**Straminea Clade (*Phytopseudomonas* gen. nov.) (6/7)**	*Pseudomonas straminea*	WP_093502677	EGVLRMAAISASLNPR	G	ESTVLGSQAQVDNLRKM
	Pseudomonas argentinensis	WP_070885465	----------------	-	-----------------
	Pseudomonas daroniae	WP_131181137	----------------	-	--M--------------
	Pseudomonas seleniipraecipitan	WP_070881722	----------------	-	--L--------------
	Pseudomonas punonensis	WP_073265652	----------------	-	--L--------------
	Pseudomonas flavescens	WP_084305768	----------------	-	--L--------------
	Pseudomonas dryadis	WP_131198471	----------------		D-L--------------
**Other *Pseudomonas***	*Pseudomonas aeruginosa*	WP_134632125	----------------		H-M---T----E-----
	Pseudomonas alcaligenes	WP_187807243	----------------		D-L--------E-----
	Pseudomonas anguilliseptica	WP_233684568	------------M---		D-L--------E-----
	Pseudomonas asuensis	WP_188864190	----------------		Q-M---T--------R-
	Pseudomonas azotifigens	WP_028241835	----------------		D-L--------E-----
	Pseudomonas benzenivorans	SDG69778	D---------------		D-L--------E-----
	Pseudomonas borbori	WP_090497674	D---------------		D-L--------E-----
	Pseudomonas brassicacearum	ADW54434	----------------		H-M---T----E-----
	Pseudomonas cavernae	WP_119892772	----------------		--L--------E-----
	Pseudomonas citronellolis	WP_061562541	----------------		Q-M---T---IE-----
	Pseudomonas composti	WP_061239955	------------I---		--V-----T--E-----
	Pseudomonas delhiensis	WP_089394062	----------------		Q-M---T---IE-----
	Pseudomonas entomophila	WP_181096610	D---------------		Q-L--------E-----
	Pseudomonas fulva	WP_196179542	D---------------		Q-L--------E-----
	Pseudomonas guguanensis	WP_090425818	------------I---		--V-V---T--E-----
	Pseudomonas guryensis	WP_182833198	----------------		D-L--------E-----
	Pseudomonas khazarica	WP_210390875	------------I---		--V-V---T--E-----
	Pseudomonas kirkiae	WP_131183246	----------------		D-L-----T--------
	Pseudomonas kunmingensis	WP_090518912	----------------		--L--------E-----
	Pseudomonas kuykendallii	PZP25767	----------------		Q-L---T----E-----
	Pseudomonas lalucatii	WP_213640750	D---------------		D-L--------E-----
	Pseudomonas leptonychotis	WP_136665991	------------M---		D-L--------E-----
	Pseudomonas lopnurensis	WP_193680809	----------------		--L--------E-----
	Pseudomonas luteola	WP_125888791	----------------		Q-M---T--------R-
	Pseudomonas mendocina	NYK07311	------------I---		--V-----T--E-----
	Pseudomonas monteilii	AMA46889	D---------------		Q-L--------E-----
	Pseudomonas nitrititolerans	WP_214329221	----------------		--L-----T--E-----
	Pseudomonas nosocomialis	WP_138411449	----------------		--L---T----E-----
	Pseudomonas oryzae	WP_090349314	----------------		--L--------E-----
	Pseudomonas oryzihabitans	WP_197851269	----------E-----		K-M-----T------R-
	Pseudomonas peli	WP_090254073	----------------		D-L--------E-----
	Pseudomonas psychrotolerans	WP_133861114	----------E-----		K-M-----T------R-
	Pseudomonas putida	WP_009397967	D---------------		Q-L--------E-----
	Pseudomonas resinovorans	WP_077527740	----------------		Q-M---T----E-----
	Pseudomonas schmalbachii	WP_208314573	----------------		H-M---T----E-----
	Pseudomonas stutzeri	NIU62364	----------------		--L--------E-----
	Pseudomonas taeanensis	WP_025165882	D---------------		D-L--------E-----
	Pseudomonas taiwanensis	WP_179060236	----------------		Q-M---T----E-----
	Pseudomonas ullengensis	WP_183090813	----------------		D-L--------E-----
	Pseudomonas urumqiensis	WP_120997785	----------D-----		D-L--------E-----
	Pseudomonas xanthomarina	WP_041013657	----------------		--L--------E-----
	Pseudomonas xionganensis	WP_160344314	----------------		D-L--------E-----
**Other Bacteria**	*Escherichia coli*	MBE1189404	----------------		H-M---T----E-----
	Klebsiella pneumoniae	SVJ72918	----------------		H-M---T----E-----
	Stenotrophomonas maltophilia	WP_164247760	----------------		H-M---T----E-----
	Streptococcus pneumoniae	CJL07450	----------------		--L--------E-----
	Streptomyces diastaticus	WP_146045982	----------------		--L--------E-----

Figure- S27

Partial sequence alignments of a protein GTP diphosphokinase showing a 1aa insertion (highlighted), which is specific for all species from Straminea clade except *P. dryadis*, which is a deep-branching species in the clade.

			167		203
**Straminea Clade** *(Phytopseudomonas* **gen. nov.)** **(6/7)**	*Pseudomonas straminea*	WP_093506440	LEVFRVSGMTMTQLREQQSE		AATEGGGLPYTVAQLAI
	Pseudomonas argentinensis	WP_070885211	--------V------A----		--AQD-----------
	Pseudomonas daroniae	WP_131178096	------------E--LR---		VGS-E-RF---------
	Pseudomonas flavescens	WP_084308395	----K---T--SE--LR---		ERSGA-CF---------
	Pseudomonas punonensis	WP_073266341	---------------A----		VNSQQP-----------
	Pseudomonas seleniipraecipitan	WP_092368846	--------V---E--QR-N-		-SSSA-HF------I-V
	Pseudomonas dryadis	WP_131173335	--------S---EH-LR-A-	QNPGN	D-P-A-RF--------V
**Other** ***Pseudomonas***	*Pseudomonas abietaniphila*	WP_062379037	---Y-----S--AH-----A	QSAQA	GGSGRHQ------N---
	Pseudomonas amygdali	WP_057433835	---Y-----S--AH----TA	QSTEA	--SGRQQ------N---
	Pseudomonas arcuscaelestis	WP_203478762	---Y---------H-QR--A	ESSAA	GPSAA-H------S---
	Pseudomonas asplenii	WP_102899449	---Y----QS--A--Q---A	QSTEA	--SGLQQ------N---
	Pseudomonas asturiensis	WP_073167970	---Y-----S--AH----TA	QSTEA	--SGRQQ------S---
	Pseudomonas atagonensis	WP_166218777	---Y----QS--A--Q---A	QSTEA	--SGLQQ------N---
	Pseudomonas azerbaijanoccidens	WP_217872660	---Y----QS--A--L---A	QSTEA	--SGRQQ------N---
	Pseudomonas baetica	WP_100846428	---Y----QS--A--Q---A	QSTEA	--SGLQQ------N---
	Pseudomonas baltica	WP_185794991	---Y----SS--E--AR--A	QSTEA	G-LAS-Q------H---
	Pseudomonas cannabina	WP_054999689	---Y-----S--AH----TA	QSTEA	--SGQQQ------N---
	Pseudomonas carbonaria	WP_187669910	--------RS--EH--L-AR	ESATA	G-SAV-Q----------
	Pseudomonas carnis	WP_197882866	---W----Q---EH-LK--A	QSTDA	G-SGQSQ------N---
	Pseudomonas cerasi	WP_065350749	---Y-----S--AH----TA	QSTEA	--SGRQQ------N---
	Pseudomonas chlororaphis	WP_038360356	---Y----QS--E--Q---A	QSTEA	--SGLQQ------N---
	Pseudomonas cichorii	WP_221556829	---Y-----S--AH------	QTAQA	T-SGRQQ------S---
	Pseudomonas coleopterorum	WP_090355880	---Y----SS--E--AR--A	QSTEP	G-LAS-Q------N---
	Pseudomonas composti	WP_061237442	---Y----LS--EH-RR-AA	GNPDT	GTSGV-Q----------
	Pseudomonas congelans	WP_032615408	---Y-----S--AH----TA	QSTEA	--SGRQQ------N---
	Pseudomonas coronafaciens	WP_122359690	---Y-----S--AH----TA	QSTEA	--SGRQQ------N---
	Pseudomonas deceptionensis	WP_048360334	---Y-----SV-EH-QK---	QTTKA	--SGHSQ------N---
	Pseudomonas endophytica	WP_055102340	---Y----QS--AH-QK---	ESTQA	--SG--Q----------
	Pseudomonas extremaustralis	WP_010563324	---W----L---EH-QR--A	QSADA	G-SGQSQ------N---
	Pseudomonas ficuserectae	WP_005752767	---Y-----S--AH----TA	QSTEA	--SGRQQ------N---
	Pseudomonas floridensis	WP_083181010	---Y-----S--AH----TA	QSTEA	--SGRQQ------S---
	Pseudomonas fluorescens	WP_034151641	---Y----QS--A--Q---A	QSTEA	--SGLQQ------N---
	Pseudomonas frederiksbergensis	WP_076028858	---Y----QS--A--L---A	QSTEA	--SGLQQ------N---
	Pseudomonas granadensis	WP_090280764	---Y----RS--D--Q---A	QSTEA	--SGLQQ------N---
	Pseudomonas gregormendelii	WP_205892008	---Y----QS--A--L---A	QSTEA	--SGRQQ------N---
	Pseudomonas guguanensis	WP_090427964	---Y----LS--EH-RR-AA	GNPDA	GTSGA-Q----------
	Pseudomonas helleri	WP_048366601	---Y----LS--AH-HK---	ESTKA	--SG--Q----------
	Pseudomonas indica	WP_084332808	---Y--------AH-QR-AA	QKPAL	DGSGA-H------H---
	Pseudomonas jessenii	WP_115145935	---Y----QS--A------A	QSTEA	--SGLQQ------N---
	Pseudomonas khorasanensis	WP_186531727	---Y----RS--A------A	QSTEA	--SGRQQ------N---
	Pseudomonas koreensis	CAH0282581	---Y----QS--T------A	QSTEA	--SGRQQ------N---
	Pseudomonas laurylsulfatiphila	WP_104451560	---Y----QS--A--L---A	QSTEA	--SGQQQ------N---
	Pseudomonas lundensis	WP_047291653	---Y----LS--AH-QK---	QSTKA	--SGASQ------H---
	Pseudomonas mandelii	WP_033055542	---Y----QS--A--L---A	QSTEA	--SGLQQ------N---
	Pseudomonas meliae	WP_044343248	---Y-----S--AH----TA	QSTEA	--SGRQQ------N---
	Pseudomonas mendocina	WP_003246511	---Y-----S-SEH-LR-AA	GNPEG	GTSAS-Q----------
	Pseudomonas moorei	WP_090328517	---Y----QS--E--L---A	QSTEA	--SGRQQ------N---
	Pseudomonas moraviensis	WP_133337184	---Y----QS--A------A	QSTEA	--SGRQQ------N---
	Pseudomonas neuropathica	WP_194935474	---Y----QS--E--Q---A	QSTEA	--SGLQQ------N---
	Pseudomonas oleovorans	NYF61105	---Y----LS-SEH-RR-AA	GNSDT	GTSA--Q----------
	Pseudomonas psychrophila	WP_019411153	---Y-----S--AH-QK---	QSTKA	--SGR-Q----------
	Pseudomonas putida	WP_046819839	--------QS--E--L---A	QSTEA	--SGRQQ------N---
	Pseudomonas reinekei	WP_075947743	---Y----QS--D--L---A	QSTEA	--SGRQQ------N---
	Pseudomonas rhodesiae	WP_094065107	---W----L---EH-QR--A	QSADA	G-SGQSQ------N---
	Pseudomonas savastanoi	KPB12928	---Y-----S--AH----TA	QSTEA	--SGRQQ------N---
	Pseudomonas savastanoi	WP_004666406	---Y-----S--AH----TA	QSTEA	--SGRQQ------N---
	Pseudomonas saxonica	WP_146384774	---Y----L---AH-QK---	QSTKA	--SGCSQ------H---
	Pseudomonas sediminis	WP_179545907	---Y-L---S--EH-LR-AA	GNPDA	GTSGA-Q----------
	Pseudomonas sivasensis	WP_181644095	---W----Q---E--LK--A	QSADA	G-SGQSQ------N---
	Pseudomonas stutzeri	MBM7380001	---Y----QS--A--L---A	QSTEA	--SGRQQ------N---
	Pseudomonas syringae	SDY11465	---Y-----S--AH----TA	QSTEA	--SGCQQ------N---
	Pseudomonas taetrolens	WP_048382756	---Y-----S--AH-QK---	ETTKA	--SGQ-Q------N---
	Pseudomonas toyotomiensis	WP_074913805	---Y----LS--EH-LR-AA	GNPDA	GTSGT-Q----------
	Pseudomonas tremae	WP_024690929	---Y-----S--AH----TA	QSTEA	--SGRQQ------N---
	Pseudomonas triticicola	WP_217864508	---Y----QS--A------A	QSTEA	--SGRQQ------N---
	Pseudomonas triticumensis	WP_187518709	---Y-----S--AH----TA	QSTEA	--SGRQQ------S---
	Pseudomonas vancouverensis	WP_093227204	---Y----QS--A--Q---A	QSTEA	--SGRQQ------N---
	Pseudomonas versuta	WP_060695770	---Y-----S--DH-QK---	ESTKA	--SGCSQ------N---
	Pseudomonas viridiflava	WP_122609371	---Y----QS--E--Q---A	QSTEA	--SGLQQ------N---
	Pseudomonas yangonensis	WP_161864729	---Y----LS--EH-RR-AA	GNPEA	GTSG--Q----------
	Pseudomonas zeae	WP_186621144	---Y----QS--E--Q---S	QSTEA	--SGLQQ------N---
	Pseudomonas zhaodongensis	WP_122163787	---Y--------EH-SR-RQ	QKAGD	G-PD--V--------SV
**Other Bacteria**	*Tanacetum cinerariifolium*	GFC27077	---Y-----S--AH-----A	QSAQA	GGSGRHQ------N---

Figure- S28

Partial sequence alignments of a protein tRNA (adenosine(37)-N6)-dimethylallyltransferase MiaA showing a 5aa Del (highlighted), which is specific for all species from Straminea clade except *P. dryadis*, which is a deep-branching species in the clade.

Group	Species	Accession	112		152
Straminea Clade (*Phytopseudomonas* gen. nov.) (7/7)	*Pseudomonas straminea*	WP_093500877	FKWVGPIAKVERVLLAAPGSNI	P	NLTGLEQARQYRIGSYK
	Pseudomonas argentinensis	WP_074886291	--------------------T-	-	-----------------
	Pseudomonas daroniae	WP_131179300	----------------------	-	--KD-------------
	Pseudomonas dryadis	WP_131198063	----------------------	-	--KD-NE-S--------
	Pseudomonas flavescens	WP_179539522	----------------------	-	--KD-------------
	Pseudomonas punonensis	WP_073263461	----------------G-----	-	---S-------------
	Pseudomonas seleniipraecipitan	WP_070882504	--------------------T-	-	---S-------------
Other *Pseudomonas*	*Pseudomonas delhiensis*	WP_089394555	------L--TGW-V--P---K-	T	T-KDVKE-GK-KL-A--
	Pseudomonas citronellolis	GBL58561	------L--TGW-V--P---K-	T	A-KDVKE-GK-KL-A--
	Pseudomonas alcaligenes	WP_076582481	------L--S-W--------T-		KVGS--E-AK-K--A--
	Pseudomonas alcaliphila	WP_074680203	---------TGW-------ND-		KVSS-QD-AK----A--
	Pseudomonas anguilliseptica	WP_233685466	------L--TGW-------N-L		SVAN-KD-A--Q--A--
	Pseudomonas asuensis	WP_188867454	------LG-TRK--MSTTA---		K--N--G-NK-K--T--
	Pseudomonas azotifigens	WP_181069992	------LSTTGW--------DV		R--S----------A--
	Pseudomonas benzenivorans	WP_090448394	------L--TGW-------N-L		SVAS-KE-A-----A--
	Pseudomonas brenneri	WP_133713980	-------GPDDW----KDD-K-		Q--E-----H-KV-A--
	Pseudomonas bubulae	WP_235572330	------LGPDDW----RGD-S-		T--S--------V-A--
	Pseudomonas carbonaria	WP_235978870	------L--TSW---S---N-L		RVNS--E-GK--V-A-R
	Pseudomonas cavernicola	WP_119954913	------L--TGW-------NTL		KVPS-KE-G--T--A--
	Pseudomonas chloritidismutans	WP_228207969	------L-SISW----PAD-PL		R--------DL---A--
	Pseudomonas chlororaphis	WP_075117405	-------GPDDW-M--RED-K-		T-QS----------A--
	Pseudomonas cichorii	RMQ42174	-------GP--W-M--RED-T-		A--D----KA-QV-A--
	Pseudomonas composti	NYG61643	-Q----LSSTR--FV-----TL		T--R--E---------R
	Pseudomonas flavescens	WP_084308360	------L--TSW--------RL		TVNS-KE-G--S--A--
	Pseudomonas fluorescens	WP_191955949	-------GPDDW----KAD-K-		Q-D------R----A--
	Pseudomonas fragi	WP_237147497	------LGPDDW----RGD-S-		T--S--------V-A--
	Pseudomonas furukawaii	ELS27174	--------RT-W-------N--		AVSD-KS-S-----A--
	Pseudomonas guguanensis	WP_090429579	------L--TGW-------ND-		KVSS-QD-AK----A--
	Pseudomonas guineae	WP_090244270	------L--TGW-------N-L		SVAN-KD-A--Q--A--
	Pseudomonas guryensis	WP_182832961	------L--SGW--------T-		TVAT-KD-A--NV-A--
	Pseudomonas indica	WP_084335862	---------TSW---S---NKL		QIKD-KD-G-----A--
	Pseudomonas indoloxydans	WP_108233816	---------TGW-------ND-		KVST-QD-AK----A--
	Pseudomonas kitaguniensis	WP_152751559	-------GPDDW----KAD-T-		Q-SD-----R-K--A--
	Pseudomonas kunmingensis	WP_102832365	------L-SISW----PAD-PL		R--------DL---A--
	Pseudomonas kuykendallii	WP_090225558	------L--TGW---S----T-		KVAS-KE-EK-KL-A--
	Pseudomonas lalucatii	WP_213639674	---------SGW-------N-L		SVGS-KE-A--K--A--
	Pseudomonas leptonychotis	WP_136664140	------L--TGW-------N--		TVAN-KD-A--Q--A--
	Pseudomonas linyingensis	WP_090312421	--------QYDS--V---ERRF		E-GS-----A----A--
	Pseudomonas lutea	WP_197871286	------LG-TRK--VSTTA-T-		K--S--G-NK----T--
	Pseudomonas luteola	WP_019367291	------LG-TRK--VSTTA-T-		K--S--G-NK----T--
	Pseudomonas matsuisoli	WP_188981724	------LSST----V----KS-		TVKD--A-K--KV-A--
	Pseudomonas mendocina	WP_106738350	------L--TGW--------D-		KVS--QD-AK--V-A--
	Pseudomonas mendocina	WP_147811717	---------TGW-------ND-		KVST-QD-AK----A--
	Pseudomonas multiresinivorans	WP_169942521	---------THW----P---S-		KVKD-KD-AK-KL-A--
	Pseudomonas nitritireducens	WP_184588177	---------TSW----P---S-		KVKD-KD-AK-KL-A--
	Pseudomonas nitrititolerans	WP_170910407	------L-STGW----P---PL		R-SS--------V-A--
	Pseudomonas nitroreducens	WP_138213858	---------THW----P---S-		KVKD-KD-AK-KL-A--
	Pseudomonas nosocomialis	WP_138406696	------LSNTGW--------D-		R--S----------A--
	Pseudomonas oleovorans	WP_125874379	---------TGW-------ND-		KVST-QE-AK----A--
	Pseudomonas orientalis	WP_181282901	-------GPDDW----KDD-K-		Q-D------R----A--
	Pseudomonas otitidis	WP_074973848	------L--SGW--------TL		SVKD-KS-G-----A--
	Pseudomonas peli	WP_090254754	------L--TGW-------N-V		SVAS-KD-A--Q--A--
	Pseudomonas protegens	WP_169892965	-------GPDDWIM--KA--T-		S-ES-----K-K--A--
	Pseudomonas psychrophila	WP_164675414	------LGPDDW----RGD-P-		P--S--------V-A--
	Pseudomonas resinovorans	WP_016495209	---------T-W-------N-L		AVPD-KS-S-----A--
	Pseudomonas resinovorans	WP_028629877	---------T-W-------N-L		SVKD-KS-S-----A--
	Pseudomonas sagittaria	WP_092433309	-Q------EYDS--V----RRF		E-NS-----A----A--
	Pseudomonas saudiphocaensis	WP_125837421	------L-ETGW----PSD-SL		R--S-----S--V-A--
	Pseudomonas saudiphocaensis	WP_193772232	------L-ETGW----PSD-PL		R--S-----S--V-A--
	Pseudomonas sediminis	WP_099526021	---------TGW-------ND-		KVSS-QD-AK----A--
	Pseudomonas seleniipraecipitan	WP_092370190	------L--TGW-------N-L		TVNS-KE-G--T--A--
	Pseudomonas sihuiensis	MBA2833056	-Q----LSSTR--FV-----TL		T--R--E-HN------R
	Pseudomonas songnenensis	WP_126188701	-------GPDDW----RGD-P-		---S-------NV-A--
	Pseudomonas stutzeri	MCF6780305	------L-ETGW----PSD-SL		R--S-----S--V-A--
	Pseudomonas taeanensis	WP_025164133	------L--TGW-------N--		SVGS-KE-G-----A--
	Pseudomonas tohonis	BCG27510	-------GPDDW----RGD-T-		S--S----K--KV-A--
	Pseudomonas urumqiensis	WP_120997311	--------HYDS--------G-		A-DS-D--------G--
	Pseudomonas viridiflava	WP_025993339	-------GP-DW----PAN-TV		A-SS-----G----A--
	Pseudomonas wenzhouensis	WP_230925936	------L--TGW--------D-		KVSS-QD-AK--V-A--
	Pseudomonas xanthomarina	SEH51147	--------QY-S---S----TL		K--S----KG-T--A--
	Pseudomonas xionganensis	WP_160343448	------L--TGW-------N--		SVAS-KD-A-----A--
	Pseudomonas zhaodongensis	WP_122163332	--------QY-S---S----SL		K--S----KG-T--A--
Other Bacteria	*Alteromonas australica*	HAW76475	--------QY-S---S-----L		K--S----QG-T--A--

Figure- S29

Partial sequence alignments of Transporter substrate-binding domain-containing protein showing a 1aa Ins (highlighted), which is commonly shared by all species from the Straminea clade. Except for its presence in *P. delhiensis* and *P. citronellolis,* this CSI is not found in any other *Pseudomonas* species.

Group	Species	Accession	48		87
**Straminea Clade** ***(Phytopseudomonas*** **gen. nov.)** **(7/7)**	*Pseudomonas argentinensis*	WP_074882567	GTTQIGWHLGGEAYKVAVPTAA		ALGVVFYILMLIGVAIMG
	Pseudomonas straminea	WP_178109882	-------Q---D--Q-------		V--I--------------
	Pseudomonas punonensis	WP_073267125	----------------------		---I--------------
	Pseudomonas flavescens	WP_084306284	----------------------		---I--------------
	Pseudomonas seleniipraecipitan	WP_083328635	-----------------M----		---I--------------
	Pseudomonas daroniae	WP_131179779	-----------------M----		---IA------V------
	Pseudomonas dryadis	WP_207390738	------------VT---M--G-		V--IT------L------
**Other** ***Pseudomonas***	*Pseudomonas matsuisoli*	WP_188983829	-------RVDT--DR---S-GL		-MALT--V-I-VA-G---
	Pseudomonas fulva	WP_196179513	----V--VIADR-AVMLTLES-	F	WMSLMS-LA--A---V--
	Pseudomonas stutzeri	WP_019341561	-------SI--GEAVKLTESS-	L	Q-TILS-LA--A---V--
**Other Bacteria**	*Buttiauxella brennerae*	WP_064557361	-------NF-DGT-VQLSMFTG	F	Y---L--A---A---V--
	Candidatus Pantoea persica	MBA2815094	----L--N--EGQLVQLNLLT-	I	G--IL--LII-G---V--
	Cedecea davisae	WP_039898013	-------NF-DGS-VQLSMFTG	L	Y--IL--A---A---V--
	Cedecea neteri	WP_061276834	-------NF-DGT-VQLSMFTG	L	Y--IL--A---A---V--
	Citrobacter telavivensis	WP_152403606	-------NF-DGNVLKLSLFTG	L	--A-L--AV--A------
	Cronobacter sakazakii	WP_085045109	----L--LS-EGHAIRLDMFT-	F	YTA-A--L---A---A--
	Enterobacter cloacae	CAF9433258	-------NF-DGTVVQLSWFTG	L	Y-AIL--C---A---V--
	Enterobacter dykesii	WP_214576666	-------NF-DGTVVQLSWFTG	L	Y-AI---G---A------
	Enterobacter hormaechei	WP_046619131	-------NF-DGTVFQLSWFTG	L	Y-AIL--G---A---V--
	Enterobacter ludwigii	QCR93801	-------NF-DGTVVQLSWFTG	L	S-AIL--G---A---V--
	Enterobacter roggenkampii	WP_063435772	-------NF-DGTVVQLSWFTG	L	Y-AII--G---V---V--
	Erwinia gerundensis	WP_187486113	-------N--SGQNIPVNLMTG	L	G---L--L-I-GA--V--
	Erwinia iniecta	WP_052896727	----L--N--AGRTIQLSLFTG	I	--A-L--A-I-G---V--
	Erwinia toletana	WP_017799099	----L--D--A-RTIQLSMFTG	I	---IL--L-I-G---V--
	Erwinia typographi	WP_034896467	-------D--AGQTIALSMLN-	F	--A-L--A-I-G---V--
	Escherichia coli	MBN6334453	----L--NF-DGTVIKLSMLTG	L	--A-L--AV--A------
	Ewingella americana	WP_140470404	----L--NF-DGQNLMVSMFT-	A	YIA-I--V---GA--L--
	Gibbsiella quercinecans	WP_121552958	----L--LS--GHAIRLGIP--	L	FAALL--V---A---V--
	Halomonas boliviensis	WP_211595356	----V--TY--PETYRVSLANG	I	----A--A-I-MA--VV-
	Halomonas campaniensis	WP_170938517	----V--TY--SETYRVSLANG	I	----A--A-I-MA--VV-
	Halomonas olivaria	BBI51652	----V--TY--PETYRVSIANG	I	----A--A-I-MA--VV-
	Halomonas populi	WP_126981420	---RL--TY--IDTYKVSI-NG	L	----A--A---VA-GLV-
	Halomonas sedimenti	WP_180094025	----V--TY--S-TYKVSVANG	I	----A--A---VA-G-V-
	Halomonas titanicae	TVU88625	----V--TY--PETYQVSLANG	I	----A--A-I-MA--VV-
	Jejubacter calystegiae	WP_138098403	-------NF-ADN-LQLSLGTG	I	G-A-L--A---A---V--
	Kalamiella piersonii	MBZ6403112	----L--NF-DGTVIKLSMLTG	L	--A-L--AV--A------
	Klebsiella aerogenes	STR15603	----L--NF-DGTVIKLSMLTG	L	--A-L--AV--A------
	Klebsiella michiganensis	PLM52108	----L--NF-DGTVVKLSLMTG	L	--A-L--AV--A---V--
	Klebsiella pneumoniae	AVK34553	----L--NF-DGTVIKLSMLTG	L	--A-L--AV--A------
	Leclercia adecarboxylata	WP_077226262	-------DFDDGNIVKLSW-TG	L	--A----A---A---V--
	Lelliottia amnigena	WP_015959733	-------NF-DGNIVKLSWVTG	L	--A-I--GI--A------
	Lelliottia aquatilis	WP_095282657	-------NF-DGTIVKLSWMTG	L	--AI---G---A---V--
	Marinomonas arctica	WP_111606812	----F--TF--HEAIKVSLMDG	L	G---L--A-I-VA--MV-
	Marinomonas shanghaiensis	WP_111637663	----F--TF--DEAIKVSLMDG	L	G---L--A-I-VA--MV-
	Mycobacterium tuberculosis	MBZ4280478	----L--NF--GTVIRLSMLTG	L	--A-L--AV--A------
	Oceanidesulfovibrio indonesien	WP_144304773	-------NF-DGTVVQLSWFTG	L	Y-AI---G---A------
	Pantoea agglomerans	PEI03279	----L--N--EGR-VQLNLATG	I	G--IL--LII-G---V--
	Pantoea alhagi	WP_085067515	----M--Y---GRSVPLALSTG	L	M-A-L--L-I-G---V--
	Pantoea allii	WP_136198295	----L--N--EGQ-VQLNLATG	I	G--I---LII-G---V--
	Pantoea ananatis	WP_058706128	---NS--N--EGQ-VQLNFPT-	L	G--IL--L-I-G---V--
	Pantoea conspicua	WP_094119400	----L--N--EGQFVQLNIATG	I	G--IL--LII-G--TV--
	Pantoea vagans	WP_033731934	----L--N--EGQ-VQLNLATG	I	G--IL--LII-G---V--
	Pantoea wallisii	WP_128601460	----L--N--EGQ-IQLNTLTG	I	G--IL--LII-G---V--
	Plautia stali symbiont	BAN95660	----L--N--EGQFVQLNMLTG	I	G--IL--LII-G---V--
	Pluralibacter gergoviae	WP_043082870	-------DF-DGNVVRLSLVTG	L	M---L--A---A---V--
	Proteus mirabilis	WP_231316831	----L--LS-EGHAIRLDMFT-	L	YTA-A--L---A---A--
	Pseudescherichia vulneris	WP_154058138	----L---F-EGVFSRLSLST-	L	L--IL--G---A---V--
	Pseudomonas xanthomarina	WP_073300596	-------SI--GEAVKLTESS-	L	Q-TILS-LA--A---V--
	Rahnella bruchi	WP_120506186	----L--NF-DGQKLMVSMIT-	S	YIA-I--V---AA--L--
	Rouxiella chamberiensis	WP_045046258	----V--NF-DGQQLQISLLT-	A	SIAAA--V-I-CA--V--
	Salmonella enterica	EAQ1117534	-------NF-DGNVLQLSLFT-	F	--A-L--GV--A---V--
	Scandinavium goeteborgense	WP_110509453	-------NF-DGTTVKLSLFTG	F	--A-L--G---A---V--
	Serratia entomophila	WP_234589920	----L--LS-EGHAIRLDMFT-	L	YTA-A--L---A------
	Serratia fonticola	VTR52503	----L--LS-QGQAIRLDMFT-	F	YTA-A--L---A------
	Serratia rubidaea	VTP61048	----L--LS-AGHAIRLDMPT-	L	YTAAA--L-I-A---V--
	Shewanella avicenniae	WP_207353459	----F--SF--DESYKVSIENG	I	---LA--A---AA-W-V-
	Shewanella dokdonensis	WP_213682447	----F--TY--DESFKVSMLNG	I	---IA--A-I-LA-SVV-
	Shewanella mangrovi	WP_037440547	----F--TF---ESYKVSIENG	I	----A--A---GA-W-V-
	Shewanella yunxiaonensis	WP_212595734	----F--TF---ESYKVSLLNG	F	---IA--A-I-LA-GVV-
	Shimwellia blattae	WP_002439883	----L--NF-SGTSVRLSL-TG	F	V------A---A---V--
	Shimwellia pseudoproteus	WP_199015320	----L--SF--G-TVRLSI-TG	F	V------A---A---V--
	Tenebrionibacter intestinalis	WP_238712172	-------NF-DG-VVKLSLWTG	L	G-A-L--A---G---V--
	Vibrio mangrovi	WP_087480986	----V--TF---ETYKVSLMNG	I	----A--A-I-MA-G-V-
	Vibrio ruber	WP_077337595	----V--TF---ETYKVSLMNG	L	----A--A-I-LA-GLV-
	Vibrio viridaestus	WP_124935662	----F--SF---ESFKVSLLNG	L	----A--A-I-LA-VVV-
	Yersinia enterocolitica	ALG80730	----V--NF-DGQSIKLA-LT-	L	YFA-I--V-I-AA--L--
	Yersinia similis	WP_025384007	----V--RF-NGQAIKLDTLT-	L	YSAII----I-AA--L--

Figure- S30

Partial sequence alignments of YIP1 family protein showing a 1aa Del (highlighted), which is specific for all species from Straminea clade. Apart from the Straminea clade, this CSI is present in one outgroup species *P. matsuisoli*.

Group	Species	Accession	55		85
Straminea Clade *(Phytopseudomonas* gen. nov.) (7/7)	*Pseudomonas argentinensis*	WP_074882425	WLRSRTLGQAEASHNQPTTL	D	SAPAPFPELA
	Pseudomonas punonensis	WP_073262582	-------E---------AA-	-	G---------
	Pseudomonas straminea	WP_093500107	-------E----------A-	S	D---------
	Pseudomonas flavescens	WP_179539197	---G---E----A-----A-	Q	A---------
	Pseudomonas daroniae	WP_131179675	---G---EE---A-----A-	Q	A---------
	Pseudomonas seleniipraecipitan	WP_070880520	---N---E----------L-	E	D----Y----
	Pseudomonas dryadis	WP_131176657	---A-S-E---------A--	H	D------S--
Other *Pseudomonas*	*Pseudomonas alcaligenes*	WP_187808015	---A-S-E----N----HL-		E---------
	Pseudomonas amygdali	KPW36770	---Q---E----A--H-EQ-		D--F---Q--
	Pseudomonas anguilliseptica	WP_090378352	---AQS-EH---A----YL-		P------Q--
	Pseudomonas azotoformans	WP_061434926	---Q-S-AD--------HD-		L------Q--
	Pseudomonas borbori	SFP60160	---A-S-E---V-----HL-		D-----AG--
	Pseudomonas campi	WP_173211756	---A-S-E---------HL-		D------Q--
	Pseudomonas canadensis	WP_123475274	---Q-S-DD--------FD-		P------Q--
	Pseudomonas caspiana	WP_087268533	---Q---SE---A--H-EQ-		A--F---Q--
	Pseudomonas chengduensis	WP_064495046	---G-S-E-----D-S-F--		-------D--
	Pseudomonas chloritidismutans	WP_023447029	---A-S-AD---A----GE-		A------Q--
	Pseudomonas cichorii	GFM87597	---Q---D---TA--H-ER-		D--Y--KQ--
	Pseudomonas citronellolis	KAF1067686	---Q---E--DGC----D--		D----Y-Q--
	Pseudomonas coleopterorum	WP_090355332	---A---AE---V--SLLD-		D------A--
	Pseudomonas coronafaciens	KGS11099	---Q---E--D-A--H-ER-		D--F--SQ--
	Pseudomonas cyclaminis	WP_193862909	---Q-S-AE------R-HD-		A------Q--
	Pseudomonas edaphica	WP_138453342	---Q-S-AD------H-YD-		P------Q--
	Pseudomonas extremaustralis	WP_150292087	---Q-S-AD--S-----HD-		P------Q--
	Pseudomonas ficuserectae	WP_054996710	---Q---E----A--H-EQ-		D--F---Q--
	Pseudomonas flavescens	WP_084308270	---G---E----A-T---A-	Q	A-----A---
	Pseudomonas fluorescens	WP_003218066	---Q-S-AE--S-----HH-		P---------
	Pseudomonas foliumensis	WP_187521451	---Q-S-D----A--H-EQ-		T--F---Q--
	Pseudomonas fragi	NNG61222	---G-S-QD--------QY-		-------Q--
	Pseudomonas grimontii	WP_090401049	---Q-S-SD--T---H-H--		P------T--
	Pseudomonas guryensis	WP_182832931	---A-S-E---------HL-		D------Q--
	Pseudomonas haemolytica	WP_153838629	---Q-S-AE--------HE-		P------Q--
	Pseudomonas karstica	WP_154744803	---Q-S-AD--S-----HA-		-------Q--
	Pseudomonas kunmingensis	WP_090523329	---A-S-AD---A----GE-		A------Q--
	Pseudomonas lopnurensis	WP_193682282	---A-SSAD---A--R--D-		A------Q--
	Pseudomonas lurida	WP_181080263	---Q-S-DD------N-YD-		A------Q--
	Pseudomonas marginalis	WP_064054120	---R-S-AA--------HD-		A------Q--
	Pseudomonas mendocina	WP_143506951	-----S-E-----D-S-FA-		----------
	Pseudomonas multiresinivorans	WP_169935794	-----S-ED-D-V--A-EQ-		D------T--
	Pseudomonas nitritireducens	WP_170858986	-----S-EE-D-V--A-QQ-		D------T--
	Pseudomonas nitrititolerans	HJE27648	---H-S-TD--TA----AR-		T---------
	Pseudomonas nitroreducens	WP_223186977	-----S-EE-D-V--A-QQ-		D------T--
	Pseudomonas oleovorans	MBA2827720	-----S-E-----D-S-FA-		----------
	Pseudomonas paralactis	WP_198707568	---R-S-AE--------HD-		P------Q--
	Pseudomonas paraversuta	WP_202211142	---G-S-QD--------QY-		-------Q--
	Pseudomonas poae	WP_060548397	---Q----E-QD-----HD-		P------Q--
	Pseudomonas pseudoalcaligenes	CDM42623	-----S-E-----D-S-F--		-----Y-D--
	Pseudomonas psychrophila	WP_019411772	---Q-S-QD--D-----HA-		D---------
	Pseudomonas reactans	WP_177001728	---Q-S-DD--------HD-		P------Q--
	Pseudomonas rhizosphaerae	WP_148308565	---A-P-AE---V--SLLD-		D------A--
	Pseudomonas salomonii	WP_069786753	---Q-S-AD--TC--H-YD-		P------Q--
	Pseudomonas savastanoi	KPB16220	---Q---E----A--H-EQ-		D--F---Q--
	Pseudomonas schmalbachii	WP_236032687	---G---E--D-V----HE-		P------Q--
	Pseudomonas sivasensis	WP_181640265	---Q-S-AD---N----HD-		P------QF-
	Pseudomonas songnenensis	WP_122098918	---A-S-AD---A----A--		A------Q--
	Pseudomonas spelaei	WP_155582972	---Q-S-A---S-----YD-		L------Q--
	Pseudomonas stutzeri	NIU61917	-----S-AD---A-------		G---------
	Pseudomonas synxantha	WP_005783887	---R-S-AE--------YD-		P------Q--
	Pseudomonas syringae	WP_027899501	---H-S-D----A--H-EH-		A--F---Q--
	Pseudomonas ullengensis	WP_183090376	---A-S-E---S-----HL-		D------Q--
	Pseudomonas veronii	WP_046385014	---R-S-AD--S---R-HE-		P------Q--
	Pseudomonas viridiflava	WP_122538011	---Q-S-AE--T---H-HA-		H------Q--
	Pseudomonas xanthomarina	WP_041015331	---A-S-AD---A----GE-		A------Q--
	Pseudomonas xionganensis	WP_160343427	---A-S-E---VT----YQ-		H---------
	Pseudomonas yamanorum	WP_177025835	---G-S-AE--S-----HY-		P---------
	Pseudomonas zhaodongensis	WP_128119863	-----S-ED---A----AQ-		L------Q--
Other Bacteria	*Halopseudomonas formosensis*	WP_090538789	-------E----A----HL-	E	G--R--AQ--
	Halopseudomonas pertucinogena	WP_188636733	-----S-E----A----QW-		D--E--AR--
	Halopseudomonas xiamenensis	WP_185267247	-------E----A----HV-		D--E--AS--

Figure- S31

Partial sequence alignments of a protein Methyl transferase showing a 1aa Ins (highlighted), which is commonly shared by all species from Straminea clade. Apart from the Straminea clade, this CSI is also present present in two other species viz. *P. flavescens* and *Halopseudomonas formosensis*.

Stutzeri Clade (Genus *Stutzerimonas*) (18/18)

```
                                                             83                                           127
Stutzerimonas stutzeri               WP_084903134   VKNRRKNGDHYWVNAYVTPILDRQR Q VTGYESVRTKPTREQIQRA
Stutzerimonas azotifigens            WP_028240941   ------D------------V--DH- - -I---------SA--VR--
Stutzerimonas balearica              WP_200628338   -------------------V--AA- N ---------Q-----VG--
Stutzerimonas chloritidismutans      WP_228207125   ------T-------------F-Q-- - ---------------V---
Stutzerimonas kirkiae                WP_131184869   ---------Y------------G-- E I--------R--E--VR--
Stutzerimonas kunmingensis           WP_102831579   ------T-------------F-Q-- - ---------------V---
Stutzerimonas xanthomarina           WP_125861831   ------T---------------Q-- - -------------------
"Stutzerimonas zhaodongensis"        WP_122167613   -----I-------------V--T-- K -I-------------V---
"Stutzerimonas nosocomialis"         WP_138408602   -------------S-----V--NN- K -V---------SAA-VS--
"Pseudomonas lopnurensis"            WP_193682201   -------------------VV-I-- T -I-------------V---
Pseudomonas nitrititolerans          WP_214330155   ------D------------V--M-S K -I----------P--V---
"Pseudomonas saudiphocaensis"        WP_193772470   ------D------------V--L-- - -I--------------K--
"Pseudomonas songnenensis"           WP_126189052   ----------------------Q-- - ---------------V---
Pseudomonas urumqiensis              WP_120995369   -------------------VM-EN- R -I----------A-HVT--
Stutzerimonas frequens               WP_063543393   ------T---------------Q-- - -----------S---V---
Stutzerimonas degradans              QGW19824       -------------D-----V--S-- S -I---------S-------
Pseudomonas phenolilytica            WP_054093032   -------------D-----V--S-- T -I---------S-------
"Pseudomonas oligotrophica"          WP_237258307   -------------------V--GT- N -I-------------VG--
```

Other *Pseudomonas*

```
Pseudomonas aeruginosa               WP_043180029   ----C--------S------Y-QGA   -V------V---A------
Pseudomonas alcaligenes              WP_110683238   ----C--------------M--KGQ   -V------V--SA--VR--
Pseudomonas alliivorans              WP_184323203   ----C--------------M-ENRQ   -V-F----I---A---R--
Pseudomonas amygdali                 WP_005738334   ----C--------------V-ENRQ   -V-F----I---A---R--
Pseudomonas argentinensis            WP_074880143   ----C-S----------V-V--DGK   --------S---A--V---
Pseudomonas asplenii                 WP_090209510   ----S--------------VFEGNQ   -V------V---A--V---
Pseudomonas asturiensis              WP_073171670   ----C--------------M-ENRQ   -V-F----I---A---R--
Pseudomonas avellanae                RMU37699       ----C--------------V-ENRQ   -V-F----I---A---R--
Pseudomonas bohemica                 WP_110947150   ----C---------------F-NKQ   -V-F----I---A---R--
Pseudomonas cerasi                   WP_065349471   ----C--------------V-ENRQ   -V-F----I---A---R--
Pseudomonas congelans                WP_096103277   ----C--------------V-ENRQ   -V-F----I---A---R--
Pseudomonas coronafaciens            KGS10745       ----C--------------V-ENRK   -V-F----V---A--VR--
Pseudomonas daroniae                 WP_131182291   ----C------------V-V--DGK   --------S---A--V---
Pseudomonas dryadis                  WP_131173691   ----C------------I----GGQ   --------S---A--VG--
Pseudomonas flavescens               WP_084306482   ----C------------I----AGQ   --------S---V--VE--
Pseudomonas floridensis              WP_083181840   ----C--------------M-ENRQ   -V-F----V---A---R--
Pseudomonas fluorescens              WP_150788210   ----A---------------F-GRE   -V------V---A---R--
Pseudomonas fulva                    WP_013791941   ----C------------V-V--DGK   --------S---A--V---
Pseudomonas fuscovaginae             WP_010451001   ----S--------------VFEGNQ   -V------V---A--V---
Pseudomonas gingeri                  WP_218176262   ----C-S------------VF-GNQ   -I------I---A---R--
Pseudomonas guryensis                WP_182833715   ----S--------------V--NNQ   -V------V---S--VK--
Pseudomonas jessenii                 WP_146242117   ----C-T-------------FEGKQ   --------V---A---R--
Pseudomonas lalkuanensis             WP_151135182   ----C---------------FEGSQ   --------V---A---R--
Pseudomonas lalucatii                MBS7691411     -------------------M-EKGQ   LV------V---T--VR--
Pseudomonas mandelii                 WP_033056568   ----S---------------FEGRE   -V------V---A---R--
Pseudomonas mangrovi                 WP_108106702   ----CQ---F---S------WEDG-   MA-F----SL-SQA-K---
Pseudomonas oleovorans               WP_104729736   ----C--------------VTENNQ   -V------V---A--VR--
Pseudomonas otitidis                 WP_165672838   ----C---------------FEGSQ   --------V---A--VR--
Pseudomonas psychrophila             WP_046809932   ----C---------------FENE-   -V------I---A---R--
Pseudomonas punonensis               WP_073264741   ----C-S----------V-V-ENGQ   --------S---A--V---
Pseudomonas putida                   WP_241803142   ----C-S-------------F-NNQ   ---F----V---A---R--
Pseudomonas reidholzensis            WP_119146096   ----C--------------VF-NNQ   -V------V---A---R--
Pseudomonas resinovorans             WP_028630896   ----C---------------FEGSQ   --------I---A--VR--
Pseudomonas savastanoi               WP_122263391   ----C--------------M-ENRK    V-F----I---A---R--
Pseudomonas seleniipraecipitan       WP_092370889   ----C------------V-V--GGK   --------S---T--V---
Pseudomonas silesiensis              WP_064678614   ----S---------------FEGRE   -V------G---A---R--
Pseudomonas straminea                WP_093501672   ----C------------V-V--DGK   --------S---A--V---
Pseudomonas viridiflava              WP_122422402   ----C----------------ENRQ   -V-F----I---A---R--
```

Other Bacteria

```
Azomonas agilis                      WP_144572212   -------------S-------NT-L   IV----------S---R--
Microvirgula aerodenitrificans       WP_028497794   ----C--------------VY-GDQ   -S------V-------A--
Pararheinheimera texasensis          WP_031566987   ----C----Y-----------QQSK   -V------V---A--V---
```

Figure- S32

Partial sequence alignments of a PAS domain-containing Methyl-accepting chemotaxis protein showing a 1aa Ins(highlighted), which is specific for all species from the genus *Stutzerimonas*.

Group	Species	Accession	115		121
Stutzeri Clade (Genus *Stutzerimonas*) (18/18)	*Stutzerimonas stutzeri*	WP_049338638	YDIISGRYIAIGMSNEEK		PHEYGYRASARDFTPAALRNAGVR
	Stutzerimonas azotifigens	WP_028239682	------------------		-F----K-----------------
	Stutzerimonas frequens	WP_102839750	------------------		------------------------
	Pseudomonas oligotrophica	WP_237256673	------------------		-F-W--K-----------------
	Stutzerimonas degradans	WP_008567041	------------------		-FDW--K-----------------
	Pseudomonas phenolilytica	WP_234302733	------------------		-FDW--K-----------------
	Stutzerimonas balearica	WP_237146211	------------------		-FDW--K-----------------
	Stutzerimonas chloritidismutans	WP_221102701	------------------		------------------------
	Stutzerimonas kirkiae	WP_131186217	-------------N----		-QQF-H------------------
	Stutzerimonas kunmingensis	NYF95753	------------------		------------------------
	"Pseudomonas lopnurensis"	WP_193682220	------------------		------------------------
	Pseudomonas nitrititolerans	HJE27828	--V------VN-------		---F--K-----------------
	"Stutzerimonas nosocomialis"	WP_138487342	------------------		--Q---K-----------------
	"Pseudomonas saudiphocaensis"	WP_193773843	-------------A----		-WA--H------------------
	"Pseudomonas songnenensis"	WP_122100060	------------------		------------------------
	Pseudomonas urumqiensis	WP_120994574	----A---L--------E		-F----E-----------------
	Stutzerimonas xanthomarina	WP_065983628	--V------VN-------		-Q-F--K-----------------
	"Stutzerimonas zhaodongensis"	WP_122168950	--V---------------		A--F--K-----------------
Other *Pseudomonas*	*Pseudomonas aeruginosa*	WP_003122200	--V-A---L-L--N----	R	GY-F-FK--SA-Y-----------
	Pseudomonas alcaligenes	WP_061902949	----A-----V--A----	S	AVQ--IQ---V-------------
	Pseudomonas alcaliphila	WP_075751082	--V-A---V-V--A----	M	SIQ--TQ---K-------------
	Pseudomonas campi	WP_173210503	---VV---V----A----	H	SV---AH---K-------------
	Pseudomonas chengduensis	MBG0844087	--V-A---V-V--A----	M	SIQ--TQ---K-------------
	Pseudomonas composti	WP_061242156	--V-A--------A----	S	AVQ--TQ---K-------------
	Pseudomonas guguanensis	SDQ02345	--V-A---V-V--A----	M	SIQ--TQ---K-------------
	Pseudomonas indica	WP_084338660	----A-----L--K----	R	GI-F-FQ---A-Y-----------
	Pseudomonas khazarica	WP_134677722	----A--------A----	T	AVQ--TQ---K-------------
	Pseudomonas kuykendallii	WP_090226938	----A---LS---N----	S	AIR--TK---N-------------
	Pseudomonas mangiferae	WP_143490309	----G---L-L--K----	H	SF-F-FK---A-Y---------I-
	Pseudomonas mangrovi	WP_108106445	--L-A---LV--LN----	S	AFQ--FK---A-------------
	Pseudomonas marincola	WP_090515012	--L-A----VT-------	E	FVQF-VK---A-------------
	Pseudomonas mendocina	WP_013714325	--V-A--------A---R	T	AVQ--TQ---K-------------
	Pseudomonas oleovorans	HIQ41114	----A--------A----	T	AVQ--TQ---K-------------
	Pseudomonas sediminis	WP_099522295	--V-A--------A---R	T	AVQ--TQ---K-------------
	Pseudomonas sihuiensis	MBA2830784	--V-A--------A---R	T	AVQ--TQ---K-------------
	Pseudomonas toyotomiensis	WP_093048992	--V-A---V-V--A----	M	SIQ--TQ---K-------------
	Pseudomonas yangonensis	WP_161864615	--V-A--------A----	T	AVQ--TQ---K-------------
Other Bacteria	*Acinetobacter baumannii*	SCY29583	--V-A---L-L--N----	R	GY-F-FK--SA-Y-----------
	Listeria monocytogenes	MCB2475021	--V-A---L-L--N----	R	GY-F-FK--SA-Y-----------
	Escherichia coli	MBE1189359	--V-A---L-L--N----	R	GY-F-FK--SA-Y-----------

Figure- S33

Partial sequence alignments of a protein DUF1329 domain-containing protein showing a 1aa Del(highlighted), which is specific for all species from the genus *Stutzerimonas*.

Group	Species	Accession	112		147
**Stutzeri Clade (Genus *Stutzerimonas*) (15/16)**	*Stutzerimonas stutzeri*	WP_084904442	TVRLEGPAAEFSGFRVAQR		SLRQGDVLNHGRYEDVK
	Stutzerimonas azotifigens	WP_028237862	E--V-----SLRE---PH-		Q--S-A-----T-----
	Stutzerimonas balearica	WP_083674897	Q---Q---TGLPA-HTPDQ		L--P-----------EA-
	Stutzerimonas chloritidismutans	ESQ99265	----D------------E-		L-KE----D------A-
	Stutzerimonas kunmingensis	SFJ20102	----D------------E-		T-------------E--
	Stutzerimonas kirkiae	WP_131184185	VLG-D-E--DLAA---PRG		T-------------E--
	Stutzerimonas xanthomarina	WP_192871748	----D------------E-		T-------------E--
	"Stutzerimonas zhaodongensis"	WP_241531154	K-QVN-----LQT---PRN		T-KP-A-----Q--A--
	"Stutzerimonas nosocomialis"	WP_138409020	-L------S--RS-T-PR-		V-VA-E--------E--
	"Pseudomonas lopnurensis"	WP_235989079	E---D----Q-PA-Q--G-		T-------------E--
	Pseudomonas nitrititolerans	WP_213908848	R-GV------LKS-QNP-G		A-EP-TT-------E--
	"Pseudomonas saudiphocaensis"	MBE7927799	---VD----QL-A-E-PRN		T-KP----H-----E--
	"Pseudomonas songnenensis"	WP_122099796	----D--------------		-----------------
	Stutzerimonas frequens	WP_267932886	-------------------		-----------------
	"Pseudomonas oligotrophica"	WP_237257420	----D---RDQP--ELPAK		L--P-----------EA-
	Pseudomonas urmiensis	WP_186555216	---I----T-LKA--IPSS	S	A--A-EP----H---A-
**Other *Pseudomonas***	*Pseudomonas anatoliensis*	WP_210699739	-I-VD----S-KS---PKA	D	V-KP-A---------A-
	Pseudomonas arsenicoxydans	WP_140667796	-I-ID----SLKS---P-G	A	--KT-A-----H---A-
	Pseudomonas atacamensis	WP_223631083	---VD----SLKS---PKS	D	L-KS-A---------A-
	Pseudomonas baetica	WP_100845700	-I-VD----S-KS---PKA	D	V-KP-A---------A-
	Pseudomonas bananamidigenes	WP_065258315	---VD----TLKS---PKS	D	V-KP-A---------A-
	Pseudomonas brassicacearum	WP_181287837	-I-I-----SLK----PDN	A	G-KS-A---------A-
	Pseudomonas brassicae	NER64841	---I----S-MKA--IPNS	R	A--T-E-----N---A-
	Pseudomonas capeferrum	WP_181132818	---V----S-LKS---PDS	K	A--P-E-----N---A-
	Pseudomonas defluvii	WP_065758521	---IQ---S-LKA--IPNS	R	Y--S-E-----H---A-
	Pseudomonas ekonensis	WP_217891402	---ID----SLKA---PKS	D	V-KP-S---------A-
	Pseudomonas entomophila	WP_213661143	---I----T-LKA--IPDS	K	A--P-EQ----H---A-
	Pseudomonas fakonensis	WP_217839003	---I----S-LKA--IPDS	R	A--P-EP----L---A-
	Pseudomonas farris	WP_217857196	---ID----S-KS---PKS	D	L-KP-A---------A-
	Pseudomonas farsensis	WP_186537284	I--I----S-LKA--IPDS	K	A--P-TP--------A-
	Pseudomonas fluorescens	MBM7763104	-I-VD----SLKS---PSS	D	T-KS-A---------A-
	Pseudomonas frederiksbergensis	WP_123358311	---ID----S-KS---PKS	D	L-KP-A---------A-
	Pseudomonas gingeri	WP_177061171	---I-----SLKA---PKS	D	T-KP-A-----H---A-
	Pseudomonas gozinkensis	WP_192561630	---ID----SLKS---PKS	D	V-KP-A---------A-
	Pseudomonas granadensis	SDT28010	---VD----SLKS---PKS	D	L-KS-A---------A-
	Pseudomonas guariconensis	WP_196170656	---I----S-LKA--IP-S	K	A--P-EP----L---A-
	Pseudomonas iranensis	WP_186566362	---VD----SLKS---PKS	D	L-KS-A---------A-
	Pseudomonas jessenii	RDL18171	-I-VD----S-KS---PKA	D	V-KP-A---------A-
	Pseudomonas juntendi	WP_182389287	---I----SDMKA---PDS	R	A--P-EQ----T---A-
	Pseudomonas koreensis	MBB4054751	---VD----SLKS---PKS	D	L-KS-A---------A-
	Pseudomonas kurunegalensis	WP_186723351	---ID---S-MKA---PDS	K	A--A-EQ----H---A-
	Pseudomonas laurentiana	WP_189396084	---I----SQLKA--IPNS	R	Y--S-E-----H-G-A-
	Pseudomonas lini	WP_048396887	---ID----S-KS---PKS	D	L-KP-A---------A-
	Pseudomonas mandelii	WP_221108975	-I-VD----SLKS---PKS	A	A-KT-A---------A-
	Pseudomonas monsensis	WP_186744396	-I-VD----ALKS---PNS	D	V-KS-A---------A-
	Pseudomonas monteilii	WP_235200565	---ID---S-MKA---PDS	K	A--A-EQ----H---A-
	Pseudomonas moraviensis	WP_073471315	---VD----SLKS---PKS	D	L-KS-S---------A-
	Pseudomonas mosselii	WP_138217410	---IN---S-LKA--IPNS	K	A--P-EP----H---A-
	Pseudomonas parafulva	WP_039583126	Q--I----S-LKA---PDS	R	A----EQ----A---A-
	Pseudomonas peradeniyensis	WP_186733264	---ID---S-LKA--IPDS	K	A--P-EQ----I---A-
	Pseudomonas plecoglossicida	WP_016394943	---I----G-MKA---PDS	R	A--A--P----L-G-A-
	Pseudomonas putida	WP_086979770	---I----S-MKA---PDS	K	A--A-EQ----H---A-
	Pseudomonas qingdaonensis	WP_196933148	---V----S-MTA---PSN	K	A--P-A-----N---A-
	Pseudomonas reidholzensis	WP_119142749	---I----S-LKA--LPDS	K	A--A-ER----T---A-
	Pseudomonas sichuanensis	WP_110994465	---I----S-MKA--IPDS	K	A--P-EQ----H---A-
	Pseudomonas syringae	WP_052967726	---I-----S-KS---P-S	D	L-KP-A---------A-
	Pseudomonas triticicola	WP_217863017	---VD----SLKS---PKS	D	L-KS-A---------A-
	Pseudomonas tructae	WP_130264046	-I-ID---S-LKA--IPDS	K	A--T-A-----H---A-
	Pseudomonas viridiflava	WP_122451273	---ID----QLPA---PAS	S	E--S-A-----H---A-
	Pseudomonas wayambapalatensis	MBC3424118	---I----S-MKA--IPDS	K	A----EQ----V---A-
	Pseudomonas xanthosomae	WP_217884975	---ID---S-LKA--IPDS	R	A--P-EP----H---A-
	Pseudomonas yangonensis	WP_161867821	DI-I----S-L-A---P-A	S	R-QP-S---------A-
**Other Bacteria**	*Arthrobacter stackebrandtii*	PYG98147	---I----S-MKA--MPDS	R	A--A-EQ----H---A-
	Priestia aryabhattai	QPN48012	---I----S-MKA---PDS	K	A--P-EQ----L---A-
	Stenotrophomonas rhizophila	AXQ50514	---I----S-MK----PDS	K	A--E-EQ----V---A-
	Thalassospira xiamenensis	PTC00228	---I----S-MKA--MPDS	R	A--A-EQ----H---A-

Figure- S34

Partial sequence alignments of a protein Autotransporter assembly complex protein TamA showing a 1aa Del(highlighted), which is specific for all/most of the species from the genus *Stutzerimonas* except *P. urumqiensis*. Homologs are absent within two ingroup species.

Group	Species	Accession	105		149
**Stutzeri Clade (Genus *Stutzerimonas*) (17/17)**	*Stutzerimonas stutzeri*	WP_014818653	LGHIVENRVVQDSLLDAMQR	R	GGLQLIGDARVEQLARTPDGWQLT
	Stutzerimonas azotifigens	WP_181070012	------------A-VERLRE	Q	ASVR-LA---L---R-SG-E-L--
	Stutzerimonas balearica	WP_043217932	------------A----LRA	G	-EIE-L-N-SLQT-E-DGT---IE
	Stutzerimonas chloritidismutans	WP_221103572	--------------------	-	------------------------
	Stutzerimonas kirkiae	WP_131188750	-------TL---G--E-A-K	-	DDIE-L-G----R-Q-DGEC-H-G
	Stutzerimonas kunmingensis	WP_090520717	--------------------	-	------------------------
	Stutzerimonas xanthomarina	WP_125876042	--------------------	-	------------------------
	"Stutzerimonas zhaodongensis"	WP_128120471	------------A--E-LKS	-	-SVE-K-N--L---QQNDN--T--
	"Stutzerimonas nosocomialis"	WP_138406717	------------A-VERLRE	-	-RVR-LA---L---R-SG-E-L--
	"Pseudomonas lopnurensis"	WP_193680104	------------A--ETL-A	-	-AVE--AA--L-R-H-SDE--L-S
	Pseudomonas nitrititolerans	WP_122075081	------------A---SL-A	S	-SVEM-AG--L-R-DPLDE--E-V
	"Pseudomonas saudiphocaensis"	WP_125837441	------------A--E-LKA	-	-SVE--AG--L---H-DDA--H--
	"Pseudomonas songnenensis"	WP_122097725	------------A--ET--S	H	-QVR-L-N------L--T---R--
	Pseudomonas urumqiensis	WP_120994302	------------A-IEQLR-	D	DKVR--EG--L--VR-SG---L--
	Stutzerimonas frequens	WP_110778754	------------A--ET---	G	-RLQ----------TQ-AE--R--
	Stutzerimonas degradans	WP_155550899	------------A--E-L-A	-	-TAE--A---L---HEVE------
	Pseudomonas phenolilytica	WP_234302965	------------A--E-L-A	-	-TAE--A---L---REVE------
**Other *Pseudomonas***	*Pseudomonas alcaligenes*	MBB4818162	---------I-EA--EPLHD		S-VG-LPN--L-N-R-SG-D-L--
	Pseudomonas amygdali pv. eriob	RMO62762	------------A---RLHE		SDIG-LAN--L--MRHSG-D-L--
	Pseudomonas asplenii	PNG42226	------------A---RLHD		CD-GMLAN--L--MR-SG-D-L--
	Pseudomonas bananamidigenes	WP_176704468	------------A---RLHD		CD-G-LAN--L--MR-SG-D-L--
	Pseudomonas borbori	WP_170862186	------------A--ERLHD		-PVG-LNH--L---R-SG-D-L--
	Pseudomonas bubulae	WP_235572440	------------A--ECL-G		SSIEMLAN--M--MR-SG-D-L--
	Pseudomonas campi	WP_173210210	------------A--ERLHD		SR-G-L-N--L---R-SG---L-K
	Pseudomonas cavernae	WP_119895404	---------I--A--ERLHA		SE-G-L-N--L---R-SG-D-L--
	Pseudomonas chlororaphis	KHA74388	------------A---RLHD		CD-GMLAN--L--MR-SG-D-L--
	Pseudomonas flexibilis	WP_039560943	------------A---CLRD		-PVR-L-Q----R-QQGEA--RVS
	Pseudomonas fluorescens	WP_191625169	------------A---RLHE		CD-GMLAN--L--MR-SG-D-L--
	Pseudomonas fragi	WP_179291710	------------A--ESL-G		SSIEMLAN--M--MR-SG-D-L--
	Pseudomonas fuscovaginae	WP_239688183	------------A---RLHD		CDMG-LAN--L--MR-SG-D-L--
	Pseudomonas gingeri	WP_177098343	------------A---RLHD		CDMG-LAN--L--MR-SG-D-L--
	Pseudomonas guangdongensis	WP_090212925	------------A--ERL-A		STVE-WPQ--L---R-SA---L--
	Pseudomonas guineae	WP_090241449	------------A--ERLHD		SPVG-LANT-----R-SG---L-S
	Pseudomonas helleri	KMN11694	------------A--ERL-D		S-I-MLAN--L--MR-SA-D-L--
	Pseudomonas helmanticensis	WP_166674967	------------A---RLHD		CD-GMLAN--L--MR-SG-D-L--
	Pseudomonas indica	MBU3055032	------------A---RLHD		S-IG-LPN--L---R-SG-D-L--
	Pseudomonas jessenii	PYC26451	------------A---RLHD		CD-GMLAN--L--MR-SG-D-L--
	Pseudomonas koreensis	ANI00619	------------A---RLHD		CD-GMLAN--L--MR-SG-D-L--
	Pseudomonas kribbensis	TFH76732	------------A---RLHD		CD-G-LAN--L--MR-SG-D-L--
	Pseudomonas lalkuanensis	WP_178119451	------------A-MEPLHD		S-IG-MPG--L-R-R-SG-D-L--
	Pseudomonas linyingensis	WP_090312671	------------A--ERL-D		S-IE-LAS--L---R-SGA--L-Q
	Pseudomonas lundensis	NNA16678	------------A--ECL-G		SNIE-LAN--L--MR-SG-E-L--
	Pseudomonas lutea	KGF64131	------------A-VERL-Q		SSIG-LA---L--MR-SG-D-L--
	Pseudomonas mangiferae	WP_143487926	------------G----LAG		SPVE-MANV-L---R-SG-D-L--
	Pseudomonas monsensis	WP_186744127	------------A---RLHD		CD-GMLAN--L--MR-SG-D-L--
	Pseudomonas neuropathica	WP_194936173	------------A---RLHD		CD-GMLAN--L--MR-SG---L--
	Pseudomonas ogarae	OPG74919	------------A---RLHD		CD-G-LAN--L--MR-SG-D-L--
	Pseudomonas orientalis	KRP65358	------------A---RLHE		CD-G-LAN--L--MR-SG-D-L--
	Pseudomonas oryzae	WP_090350397	------------A--ERL-D		S-IE-LAH--L---R-SAG--L-Q
	Pseudomonas panacis	WP_186511889	------------A--ERLHD		CD-G-LAN--L--MR-SG---L--
	Pseudomonas paraversuta	WP_202211331	---------I--A--ECL-A		TNIE-LAN--L--MR-SA-D-L--
	Pseudomonas pohangensis	WP_090197422	---------I--A--EQLLA		S-V-VL-E--L-A-R-NAE----K
	Pseudomonas prosekii	RLU07202	------------A---RLHE		CD-G-LAN--L--MR-SG-D-L--
	Pseudomonas psychrophila	EPJ94480	------------A--ECL-A		S-IEMLAN--L--MR-SG-E-L--
	Pseudomonas sagittaria	WP_092431526	------------A--ERL-G		C-IE-LAA--L---R-SGE--L-Q
	Pseudomonas savastanoi	RMV23587	------------A---RLHE		SDIG-LAN--L--MRHSG-D-L--
	Pseudomonas saxonica	TWR94225	------------A--ECV-G		ADIEMLAN--L--MR-SG-D-L--
	Pseudomonas syringae	MCF5700845	------------A---RLHD		CD-GMLAN--L--MR-SG-D-L--
	Pseudomonas taetrolens	KMM82530	------------A---CLHG		TAIEMLSN--L--MR-SG-D-L--
	Pseudomonas tensinigenes	WP_186613425	------------A---RLHD		CD-GMLAN--L--MR-SG-D-L--
	Pseudomonas tohonis	WP_173176642	---------I-EA--EPLHD		S-VG-LPN--L-R-R-SG-D-L--
	Pseudomonas ullengensis	WP_183087552	------------A---RLHD		SQ-G-LAN--L---R-SG---L-K
	Pseudomonas versuta	ALE90215	---------I--A--ECL-V		TNIE-LAN--L--MR-SG-D-L--
	Pseudomonas viridiflava	WP_163004684	------------A---RLHD		CD-GMLAN--L--MR-SG-D-L--
	Pseudomonas weihenstephanensis	WP_173426988	------------A---SL-G		VDIE-LAN--L--MR-SG-D-L--
	Pseudomonas xionganensis	WP_160342791	------------A--ECLHE		SPIG-LPG--L---R-SG---L--
	Pseudomonas zeae	WP_186620423	------------A---RLHD		CD-GMLAN--L--MR-SG-D-L--
**Other Bacteria**	*Azomonas agilis*	WP_144571457	-------Q-------EQLET		TKVR--SN-HLSA-T-LDN--CV-
	Azomonas macrocytogenes	WP_183165613	------------A--ERLKT	C	AAVE-VAGT-L---Q-KGND----
	Enterobacter hormaechei	MBX8630705	------------A---RLHD		STTG-LAN--L--MRHSG-D-L--

Figure- S35

Partial sequence alignments of a protein 2-octaprenyl-3-methyl-6-methoxy-1,4-benzoquinol hydroxylase showing a 1aa Ins(highlighted), which is specific for all species from the genus *Stutzerimonas* but not shared by any other *Pseudomonas* species or other bacterial species. Homolog is absent for one ingroup species. One distant outgroup species *Azomonas macrocytogenes* also shares this CSI.

Group	Species	Accession	237		265
Stutzeri Clade (Genus *Stutzerimonas*) (18/18)	*Stutzerimonas stutzeri*	WP_218422476	MLVWLVICLTGVIEVLSFG	AL	AIANAAHV
	Stutzerimonas azotifigens	WP_028239322	--A--L-------D-V---	T-	--------
	Stutzerimonas balearica	MBD3737582	------------V------	--	--------
	Stutzerimonas chloritidismutans	WP_031297604	-------------------	--	--------
	Stutzerimonas degradans	EKM95807	---------S---D-----	T-	--------
	Pseudomonas phenolilytica	WP_234302268	---------S---D-----	T-	--------
	Stutzerimonas frequens	WP_200595239	-------------------	--	--------
	Stutzerimonas kirkiae	WP_131185459	--A--LV--S-I-DLA---	T-	S-------
	Stutzerimonas kunmingensis	WP_181066948	-------------------	--	--------
	Stutzerimonas xanthomarina	WP_125861665	-------------------	--	--------
	“*Stutzerimonas zhaodongensis*”	WP_122164117	--I-----------MV---	--	--------
	“*Stutzerimonas nosocomialis*”	WP_241663873	--A------------V---	--	--------
	“*Pseudomonas lopnurensis*”	WP_193681510	------V------------	--	--------
	Pseudomonas nitritiitolerans	WP_213909238	--I---V--S-A-------	TM	--------
	“*Pseudomonas saudiphocaensis*”	WP_037022898	--I---V----A-------	T-	--------
	“*Pseudomonas songnenensis*”	WP_122098614	-----------------S-	--	--------
	Pseudomonas oligotrophica	WP_237257712	-----LV--S-LV------	--	--------
	Pseudomonas urumqiensis	WP_120996629	--A--L--------LV---	SV	--------
Other *Pseudomonas*	*Pseudomonas aeruginosa*	WP_227389036	--I--LV--S---DL-G--		S---G---
	Pseudomonas alcaliphila	WP_074681472	--A--L--M--IF-L-Q--		--------
	Pseudomonas anguilliseptica	WP_090384513	--A--L--M---F-L-Q--		--------
	Pseudomonas argentinensis	WP_070886442	-----L--M---F-L-Q--		--------
	Pseudomonas benzenivorans	WP_090442133	--A--L-----IF-L-Q--		--------
	Pseudomonas chengduensis	WP_021489727	--A--L--M--IF-L-Q--		--------
	Pseudomonas composti	WP_037004307	--A--A--M---F-L-Q--		--------
	Pseudomonas daroniae	WP_131192121	--I--L--M---F-L-Q--		--------
	Pseudomonas duriflava	WP_145143165	--A--A-------D--G--		----G---
	Pseudomonas flavescens	WP_084304068	-----L--M---F-L-Q--		--------
	Pseudomonas fulva	WP_013790929	--I--L--M---F-L-Q--		--------
	Pseudomonas guguanensis	WP_090432830	--A--A--M--IF-L-Q--		--------
	Pseudomonas guineae	WP_090242944	--A--L--M--IF-L-Q--		--------
	Pseudomonas hydrolytica	MCF2123485	--A--L--M--IF-L-Q--		--------
	Pseudomonas leptonychotis	WP_136662514	--A-----M--IF-L-Q--		--------
	Pseudomonas marincola	WP_150549561	--A--L--M--IF-L-Q--		S-------
	Pseudomonas matsuisoli	WP_188982492	------V-MS-LVSA-G--		--------
	Pseudomonas mendocina	WP_017364042	--I---V-M--IF-T-GV-		--------
	Pseudomonas oleovorans	NYF63633	-----L--M--IF-LRQ--		--------
	Pseudomonas otitidis	WP_236212966	------V--S--VSL-G--		----G---
	Pseudomonas peli	WP_090252316	--A--L--M--IF-L-Q--		--------
	Pseudomonas psychrotolerans	WP_058776968	--I---------FG--G--		----G---
	Pseudomonas sediminis	WP_099523279	--I---V-M--IF-T-GV-		----G---
	Pseudomonas segetis	WP_089358830	--A--L--M---F-L-Q--		S-------
	Pseudomonas seleniipraecipitan	WP_070883916	--I--L--M---F-L-Q--		--------
	Pseudomonas straminea	WP_093503268	-----L--M---F-L-Q--		--------
	Pseudomonas taeanensis	WP_025165551	--A--L--M--IF-L-Q--		--------
	Pseudomonas toyotomiensis	WP_074916244	--I---V-M--IF-T-GI-		--------
	Pseudomonas wenzhouensis	WP_230927287	--I---V-M--IF-T-GI-		--------
	Pseudomonas xionganensis	WP_160347907	--A--L--M--IF-L-Q--		--------
	Pseudomonas yangonensis	WP_161866940	--A--A--M--IF-L-Q--		--------
Other Bacteria	*Acinetobacter baumannii*	WP_146179407	--I--LV--S---DL-G--		S---G---
	Azomonas agilis	WP_144571696	--I--LV--S-LV-T----	-M	S-------
	Azomonas macrocytogenes	MBB3104730	--A--L---S-I-T-V-L-	-M	--------
	Escherichia coli	WP_160340500	--I---V-M--IF-T-GL-		--------
	Klebsiella pneumoniae	WP_159334094	--I--LV--S---DL-G--		S---G---

Figure- S36

Partial sequence alignments of a protein Rhomboid family intramembrane serine protease showing a 2aa Ins (highlighted), which is specific for all/most of the species from the genus *Stutzerimonas*. This CSI is also shared by two distant outgroups *Azomonas macrocytogenes* and *Azomonas agilis* but not by any other *Pseudomonas* species or other bacterial species.

Group	Species	Accession	165		212
**Stutzeri Clade (Genus *Stutzerimonas*) (17/18)**	*Stutzerimonas stutzeri*	WP_106442915	DGWTQATALDVLKVNKSLTIDELW		RNPAFGHFGGIGSEVVNLAFLAGG
	Stutzerimonas azotifigens	WP_028240449	---AR--------T-R---VE---		Q---------M-A-I---------
	Stutzerimonas balearica	WP_200291116	---S---------------V----		------------------------
	Stutzerimonas chloritidismutans	WP_023444738	------------------------		S-----------------------
	Stutzerimonas kirkiae	TBU94613	---S-------AH-DR---LE---		QA-V--LV-AA---L--F------
	Stutzerimonas kunmingensis	WP_102831927	------------------------		T-----------------------
	“*Stutzerimonas nosocomialis*”	WP_138408199	-A-----------N-Q---V----		QG----------------------
	Stutzerimonas xanthomarina	WP_125862264	------------------------		S-----------------------
	“*Stutzerimonas zhaodongensis*”	WP_128121525	-------------------V----		A-----------------------
	“*Pseudomonas lopnurensis*”	WP_193681805	-----------M---N---V----		------------------------
	Stutzerimonas frequen	WP_200594556	------------------------		------------------------
	Pseudomonas phenolilytica	WP_043295925	---S---------L-NR---E---		------------------------
	Stutzerimonas degradans	WP_102827501	---S---------L-NR---E---		------------------------
	Pseudomonas oligotrophica	WP_237258212	---S------A--I-N---VA--W		------------------------
	Pseudomonas nitrititolerans	WP_214331764	---S-------M-L-R---VE---		T-----------------------
	“*Pseudomonas saudiphocaensis*”	WP_193773661	---S-------M-L------E---		A-----------------------
	“*Pseudomonas songnenensis*”	WP_122099484	------------------------		S-----------------------
	Pseudomonas urumqiensis	WP_245961991	---S---L--I--H-QR---E--F	E	-V----A--AR-L-W---------
**Other *Pseudomonas***	*Pseudomonas flexibilis*	WP_039605626	---S---------T----------		KQ-----Y----------------
	Pseudomonas mangrovi	WP_108106418	---S---------I-Q---VA---		Q------Y----------------
	Pseudomonas alcaligenes	WP_110682371	---S------A-------------	A	Q-----S---KAV-W---------
	Pseudomonas alcaliphila	WP_045734444	---A------A------------R	A	S-A-------A---A---------
	Pseudomonas allokribbensis	WP_192558875	-A-V------S-RI---------F	A	S-----R---H-V-W---------
	Pseudomonas anatoliensis	WP_210700255	-A-V------S-RI---------F	A	A-----R---H-M-W---------
	Pseudomonas anguilliseptica	WP_090384729	---A------A-------------	A	Q-----YL--AAT-A---------
	Pseudomonas arsenicoxydans	WP_140668343	-A-A------S-RI-----M---F	A	G---------R-V-W---------
	Pseudomonas arsenicoxydans	WP_208669677	-A-V------S-RI-----M---F	A	G---------R-V-W---------
	Pseudomonas asplenii	WP_102901036	-A-V------T-RI---------F	A	A-----Q---H-V-W------V--
	Pseudomonas azerbaijanoccidens	WP_217872800	-A-VR-----S-RI-----M---F	S	A---------R-V-WL--------
	Pseudomonas azerbaijanoriens	WP_217836272	-A-V------S-R------M---F	A	G------Y--R-V-W---------
	Pseudomonas baetica	WP_100845210	-A-V------S-RI---------F	A	A-----R---R-A-W------V--
	Pseudomonas batumici	WP_040070335	-A-AT-----A-RI-----L---F	T	S---------K---WL--------
	Pseudomonas benzenivorans	WP_090448160	----------A--T-----M----	A	QS----R---AAV-----------
	Pseudomonas borbori	WP_090498606	----------AI-I-----M----	A	QS----R---TAV-A---------
	Pseudomonas brassicacearum	WP_123368473	-A-V------S-RI-----M---F	A	A---------R-M-W---------
	Pseudomonas caspiana	WP_140893494	-A-V------S-RI-----M---F	A	A---------R-M-W---------
	Pseudomonas chengduensis	WP_017677834	---A------A------------R	A	T---------A---A---------
	Pseudomonas chlororaphis	WP_047736600	-A-AG-----S-RL-R-------F	A	H---------R-A-W---------
	Pseudomonas composti	NYG64055	---A------A------------R	A	A---------A---A---------
	Pseudomonas extremaustralis	WP_150292756	-A-A------A-RI---------F	A	-S--------RA--W-G--Y-M--
	Pseudomonas fildesensis	WP_048720486	-A-A------A-RI---------F	A	-S----F---N---W---------
	Pseudomonas fluorescens	WP_150779443	-A-A------S-RI-----M---F	A	G---------R-V-W---------
	Pseudomonas frederiksbergensis	WP_076028465	-A-V------S-RI-----M---F	A	A---------R-M-W---------
	Pseudomonas gingeri	WP_177102762	-A-V------T-RI-----M---F	A	G---------K---WL--------
	Pseudomonas granadensis	WP_203419315	-A-A------T-R--A-------F	A	-H--------RAA-W---------
	Pseudomonas guangdongensis	WP_090214298	-A--------A---------A---	A	S-----SL--Y-A-A---------
	Pseudomonas guguanensis	WP_090426695	---A------A------------Y	A	V---------T---A---------
	Pseudomonas guineae	WP_090242774	---A------A-------------	T	Q-----R---A---A---------
	Pseudomonas indoloxydans	WP_108235146	---A------A------------R	A	S-A-------A---A---------
	Pseudomonas izuensis	WP_160106336	-A-VR-----S-RI-----M---F	S	A---------R-V-W---------
	Pseudomonas jessenii	WP_110662580	-A-V------S-RI-----M---F	A	A------Y--R-V-WM--------
	Pseudomonas koreensis	AVX90817	-A-V------S-RI-----M---F	A	A-----R---R-V-LI--------
	Pseudomonas lalucatii	MBS7691805	-------------I-Q---V----	A	QH----R---AAV-A------T--
	Pseudomonas laurylsulfatiphila	WP_104449287	-A-V------S-RI-----M---F	A	G------Y--R-V-W---------
	Pseudomonas leptonychotis	WP_136662481	---A------A--I----------	A	QS----R---A---A---------
	Pseudomonas linyingensis	WP_090313420	-A--------A---------A---	E	S--S--SV--Y---A---------
	Pseudomonas mandelii	WP_083375853	-A-A------S-RI-----M---F	A	S---------H-V-W---------
	Pseudomonas marincola	WP_150547310	---V------T--I----------	T	Q-----YI--A-------------
	Pseudomonas mendocina	WP_115297868	---A------A------------H	A	S---------A-------------
	Pseudomonas moorei	WP_090324887	-A-V------S-RI-----M---F	A	T-----Q---R-V-W---------
	Pseudomonas oleovorans	MBA2826043	---A------A------------R	A	S---------A-------------
	Pseudomonas oryzae	WP_090349065	-A--------A--------MA---	A	G-----N---Y---A---------
	Pseudomonas peli	WP_090252373	---A------A-------------	A	QH----YL--AAT-A---------
	Pseudomonas protegens	WP_123472912	-A-A------S-RI-----V---F	A	G---------R-T-W---G-----
	Pseudomonas reinekei	WP_075945526	-A-VR-----S-R------M---F	S	AS--------R-V-W---------
	Pseudomonas sagittaria	WP_092432787	-A--------A---------A---	A	S--S--SL--Y-A-A---------
	Pseudomonas sediminis	WP_099522103	---A------A------------H	A	S-----Y---A-------------
	Pseudomonas segetis	WP_089358586	---A------A--I----------	M	Q-----R---A-A--------L--
	Pseudomonas sihuiensis	WP_092374315	---A------A------------R	A	S-V-------A---A---------
	Pseudomonas syringae	KFE52522	-A-S--------RI-----V---F	A	-S----SL--R-V-W---------
	Pseudomonas taeanensis	WP_025165939	----------A--I----------	A	QH----R---ASV-A---------
	Pseudomonas tensinigenes	WP_186615383	-A-V------T-RI---------F	A	A-----Q---H-V-W------V--
	Pseudomonas toyotomiensis	WP_196455802	---A------A------------R	A	S-V-------A---A---------
	Pseudomonas vancouverensis	WP_093226458	-A-A------S-RI-----M---F	A	S---------R-A-WL--------
	Pseudomonas veronii	RWA28755	-A-A------A-RL---------F	A	-------V--K---W---------
	Pseudomonas wenzhouensis	WP_230925082	---A------A------------R	A	A---------A---A---------
	Pseudomonas xionganensis	WP_160346125	---A------A----N---M---R	A	A---------A---A---------
	Pseudomonas yangonensis	WP_161864426	---A------A------------Y	A	G---------A---A---------
**Other Bacteria**	*Azotobacter beijerinckii*	WP_090625317	---S-------R-A-R---M----	A	Q-----YL--R---W---------
	Azotobacter chroococcum	WP_089168605	---S---------T-R---ME---	A	Q-----YL--R-A-W---------
	Azotobacter vinelandii	WP_061288598	---S------A--T-R---M---R	A	Q------L-SR-A-W---------

Figure- S37

Partial sequence alignments of a protein RnfABCDGE type electron transport complex subunit D showing a 1aa Del(highlighted), which is specific for all/most of the species from the genus *Stutzerimonas* except *P. urumqiensis*. One exception of this CSI is that it is also shared by two outgroup species *P. flexibilis* and *P. mangrovi* but not by any other *Pseudomonas* species or other bacterial species.

Group	Species	Accession	142		170
**Stutzeri Clade (Genus *Stutzerimonas*) (16/18)**	*Stutzerimonas stutzeri*	WP_221292728	GRSVLPIIHAPVTLRDWL		DVEADLKLVLH
	Stutzerimonas azotifigens	WP_028238441	------K-----A-E---		G-Q--------
	Stutzerimonas balearica	WP_200290538	------TL---QP-AQ--		GI------L--
	Stutzerimonas chloritidismutans	WP_045665856	---A-----P-MS-----		P----------
	Stutzerimonas kirkiae	WP_131185296	----V-K--P-QA-AQ--		PLQ-E---L--
	Stutzerimonas kunmingensis	WP_181065987	------L-----S-----		TL---------
	Stutzerimonas xanthomarina	WP_125875963	------V--P-IS-----		P----------
	"Stutzerimonas zhaodongensis"	WP_241529441	----V-T--P-MP-Q---		GLD--------
	"Pseudomonas lopnurensis"	WP_193680988	------V----RS-DG--		S---E------
	Pseudomonas nitrititolerans	WP_014851236	----I----P-QL-A---		AI---------
	"Pseudomonas saudiphocaensis"	WP_125837567	----I-L--P-AP-AQ--		A---Q------
	Stutzerimonas frequens	WP_110778841	---------S--A-----		A----------
	"Pseudomonas oligotrophica"	WP_237255679	------V----MA-G---		A---T------
	Pseudomonas phenolilytica	WP_054094373	----V-T--P-QQ-G---		P---E------
	Stutzerimonas degradans	OOE11112	----V-T--P-QP-G---		P---E------
	"Pseudomonas songnenensis"	WP_122097794	-----------TS-----		T---E------
	"Stutzerimonas nosocomialis"	WP_138412179	------R-Q---S-E---	Q	RT------L--
	Pseudomonas urumqiensis	WP_120994840	------V----QP-DQ--	Q	GAD--------
**Other *Pseudomonas***	*Pseudomonas alcaligenes*	MBB4818337	------V----IN-A---	K	Q-D--------
	Pseudomonas alliivorans	WP_210073883	----V-V--P-MP-A---	K	QT---------
	Pseudomonas amygdali	WP_010195539	----V-V----MP-AE-I	K	HT---------
	Pseudomonas anguilliseptica	WP_090380424	---------P-IS-T---	T	Q-S--------
	Pseudomonas argentinensis	WP_070885879	----I-V--P-IA-N---	Q	QTQ--------
	Pseudomonas asiatica	WP_182328867	---T--V--P----AE--	N	SAK--------
	Pseudomonas asuensis	WP_188865446	---T--V-------TE-I	E	RA---------
	Pseudomonas cannabina	WP_055001322	----V-V--P-MP-S--I	K	LT---------
	Pseudomonas carbonaria	WP_187669794	------V-------D---	A	Q----------
	Pseudomonas cichorii	WP_201005162	----V-V--P-MP-A---	K	RT---------
	Pseudomonas congelans	WP_096101448	----V-V----MP-TE-I	K	HT---------
	Pseudomonas daroniae	WP_131180031	----I-M----I--D---	Q	QS--E------
	Pseudomonas entomophila	WP_125465829	---T--V--P-L--AE--	K	AC---------
	Pseudomonas flexibilis	WP_039561127	------V-----S-D--Q	R	Q-Q-E------
	Pseudomonas fluorescens	WP_046069903	---TV-V-----L-A---	K	Q----------
	Pseudomonas fulva	WP_196175841	------V--P-LA-G--I	K	AS---------
	Pseudomonas furukawaii	WP_003455553	------V-----A-A--Q	R	Q----------
	Pseudomonas graminis	WP_172610002	----V-V-------A---	K	QT---------
	Pseudomonas guariconensis	WP_196155209	---TV-V--P-L--A---	K	ETD--------
	Pseudomonas haemolytica	WP_153837466	---TV-V----LL-A---	K	Q----------
	Pseudomonas indica	MBU3057402	------V--P-LL-S---	R	QT---------
	Pseudomonas khavaziana	WP_217880878	---TV-V----LL-A---	K	Q----------
	Pseudomonas knackmussii	WP_160285653	------Q-N--I--AE--	Q	G---E------
	Pseudomonas lalkuanensis	WP_151131360	------V----IA-A--Q	R	Q----------
	Pseudomonas lalucatii	WP_213641692	-------L-P-I--SE--	Q	R---Q------
	Pseudomonas libanensis	WP_057014142	---TV-V----LL-A---	K	Q----------
	Pseudomonas lutea	KGF62267	----V-V-N-----A---	K	QTD--------
	Pseudomonas luteola	WP_125887757	---T----------AE--	G	K----------
	Pseudomonas maumuensis	WP_217867445	---T--V--P----AE--	K	ST---------
	Pseudomonas mendocina	WP_147809463	------L----LE-EA--	Q	QI---------
	Pseudomonas monteilii	KXK67533	---T--V--P----AE--	N	SAN--------
	Pseudomonas mosselii	WP_138219235	---T--V--P-L--AE--	K	AT---------
	Pseudomonas nosocomialis	WP_138406839	------R-Q---S-E---	Q	RT------L--
	Pseudomonas oryzicola	WP_186672379	---T--V--P----AE--	N	SAR--------
	Pseudomonas otitidis	WP_107329851	------V--P-----E-Q	K	Q----------
	Pseudomonas parafulva	WP_116887416	---S--T-------AE--	K	ATD--------
	Pseudomonas peli	WP_090250070	---------P-IS-N---	G	Q-T-E------
	Pseudomonas peradeniyensis	WP_186733328	---T--V--P----AE--	K	GT---------
	Pseudomonas plecoglossicida	WP_016395331	---T--V--P----AE-I	K	TS---------
	Pseudomonas poae	WP_197626512	---TV-V--P-LL-A---	K	Q----------
	Pseudomonas punonensis	WP_073263016	---M--L--S-I--N---	Q	QSR--------
	Pseudomonas putida	WP_049272966	---T--V--P----AE--	G	STQ--------
	Pseudomonas resinovorans	WP_028629559	------V--S----A--Q	R	--Q--------
	Pseudomonas savastanoi	RMT80434	----V-V----MP-AE-I	K	HT---------
	Pseudomonas seleniipraecipitan	WP_092367235	----I-V--S-I--EE--	Q	HSQ--------
	Pseudomonas shirazica	WP_217189982	---T--V--P----AE--	S	SAK--------
	Pseudomonas synxantha	WP_068965661	---TV-V-----L-A---	K	Q----------
	Pseudomonas syringae	WP_020307112	----V-V--P-MP-A--I	N	LT---------
	Pseudomonas taiwanensis	WP_179058474	------V-------V--Q	R	--Q--------
	Pseudomonas tohonis	WP_173175042	------V----I--A---	V	QAR--------
	Pseudomonas urmiensis	WP_186557377	---T--V--P-L--AE--	K	AT---------
	Pseudomonas viridiflava	MBB5990906	----V-V--P-MP-A---	K	QT---------
	Pseudomonas vlassakiae	WP_186601720	---T--V--P----AE--	K	GT--E------
	Pseudomonas wayambapalatensis	MBC3425233	---T--V--P----AE--	K	-S--E------
**Other Bacteria**	*Azomonas macrocytogenes*	WP_183166787	------V----QA-A---	Q	RT--E-R----
	Halopseudomonas pachastrellae	HIQ53075	----V-V--P-AS-Q--M	A	-LQ-EV-----
	Salinibius halmophilus	WP_119394844	---DV----P-IS-N---		NI---C-----
	Tanacetum cinerariifolium	GEX30933	----V-T-N-----A---	K	QTD--------

Figure- S38

Partial sequence alignments of a protein 16S rRNA (uracil(1498)-N(3))-methyltransferase showing a 1aa Del(highlighted), which is specific for all the species from the genus *Stutzerimonas* except *P. urumqiensis* and *P. nosocomialis*.

Group	Species	Accession	372		421
**Linyingensis Clade (*Geopseudomonas* gen. nov.) (6/6)**	*Pseudomonas linyingensis*	WP_090305970	CRAVVLLGRDAELIAEALKGE	DGEAV	VPLLRVNSLDEAVQRSVELAEAGD
	Pseudomonas guangdongensis	WP_090211298	-----------G---A--Q-D	--QP-	---T--D--ED--R-CA---Q---
	Pseudomonas oryzae	WP_090347150	---------------G--TAS	--QP-	---V--A--------CA---A---
	Pseudomonas sagittaria	WP_092427227	-------------L----R--	-----	------AT-------CAG------
	Pseudomonas aromaticivorans	WP_217682845	--T------------A---DS	A-RD-	------AT-------CA-------
	Pseudomonas oryzagri	WP_229604562	--A-A----------A--TGA	D-QS-	---V--AT-A-----CA---Q---
**Other *Pseudomonas***	*Pseudomonas aeruginosa*	WP_003103104	-----------G---Q--GNA		---V--AT-----RQAA---RE--
	Pseudomonas aestusnigri	WP_088274823	---------------A--DPQ		TR-V--A-IEK--TAAAA---Q--
	Pseudomonas alcaligenes	WP_061903425	------------Q--K--GDA		A-----KT-------CA---QS--
	Pseudomonas anguilliseptica	WP_090382345	-------------L----GDA		A--V--KT-E-----AA----Q--
	Pseudomonas asplenii	WP_090202101	-------------L-Q--GD-		---I--QT--------A---HS--
	Pseudomonas brassicae	WP_178116374	------------RL----GDA		---I--A-------QCAT--Q---
	Pseudomonas chlororaphis	WP_009050718	----I-M---SDK-G--IGDA		---I--G--V---AQCRAT-Q---
	Pseudomonas citronellolis	WP_074977771	-------------VGA--SD-		T-KV--QT-----RKAA---RP--
	Pseudomonas fluorescens	WP_053254469	------M----D---A--GDA		--QV-AT---D-ITQCQA--QP--
	Pseudomonas fragi	WP_029611244	------M----G---D--GDA		--QV--A-----IAQ-RA--QP--
	Pseudomonas graminis	WP_074884462	-----------D-L-ATFGDS		---V--TT-E-----AADI-QE--
	Pseudomonas grimontii	WP_090409311	------M---SD---A--GDA		-LQV-AT---D-IAQCKA--QP--
	Pseudomonas indica	WP_084336614	------------RL----D-A		A--V--KT-----RNAA---SS--
	Pseudomonas jessenii	WP_057713362	------M---SDK-G--IGDA		---I-AG--V---EQCRAA-QP--
	Pseudomonas kuykendallii	WP_090226559	------------RLGA--GEG		--QV--A------R--A---LP--
	Pseudomonas lactis	WP_057710941	------M---SD---A--GDA		--QV-AA---D-IAQCKA--QP--
	Pseudomonas laurylsulfatiphila	WP_104449359	------M---SDK-G--IGDA		---I--A--V---EQCRAA-QP--
	Pseudomonas lutea	WP_037014689	-----------D-L-ATFGDS		---V--TT-E-----AADI-RE--
	Pseudomonas massiliensis	WP_040261834	------------RL-A--DP-		L-RYH-AD--A--A-CAQ--QP--
	Pseudomonas matsuisoli	WP_188985455	------M-----HL-A--DPA		T-AV-ASNM----ACAAK--R---
	Pseudomonas nitritireducens	WP_037009771	-------------VSA-IGNA		--KV---T-E---EHAAD--LP--
	Pseudomonas oleovorans	WP_167519207	------------RL--T-GDA		A-QV--KT-E-----GC-------
	Pseudomonas otitidis	WP_074971598	---------------A--GDA		---V--KT-------CA---RE--
	Pseudomonas protegens	WP_015636929	----I-M---SDQ-G--IGDA		---I--G--Q---EQCRAT-QP--
	Pseudomonas reidholzensis	WP_119143487	------------RLGQ--GEQ		-QQV--QT------QCAA--QP--
	Pseudomonas resinovorans	WP_041770143	---------------D--GDS		--QV--KT-------CA-------
	Pseudomonas rhizosphaerae	WP_043186839	---L-------AR--AVAGPD		-AQVN-A-IE-----CA---QP--
	Pseudomonas soli	WP_038707414	------I----ARL--T--DA		-Q-VP-QT-------CA---R---
	Pseudomonas straminea	WP_093504121	-----------DKL-AV-D-A		-DII--D--QA--E-AAAI-QS--
	Pseudomonas stutzeri	WP_013981961	-----------PRL----GEA		TV-I--A--E-----AA-C--S--
	Pseudomonas syringae	WP_025388933	---A-----------QV-GDA		---I--DT-QT--E--A---RS--
	Pseudomonas taiwanensis	WP_023378831	------------RL----GDG		-Q-V--KT-----HQCA---RS--
	Pseudomonas thermotolerans	WP_026146284	----L----------R--GDG		---V--KT-----A-AA-------
	Pseudomonas typographi	WP_194719548	------------RL-S--GSD		--HV--A---D--A-CT---QP--
	Pseudomonas viridiflava	WP_088234610	--S---------RL-Q--GDA		A--VH-DT-QL--E--AG--QD--
	Pseudomonas yamanorum	WP_093205273	------M---SG---D--GDA		--QV-AQ---D-IAQ-KA--QP--
**Other Bacteria**	*Azomonas agilis*	WP_144570189	----I-I----DR---V-S-T		-V-EAAA--E---ELAAKQ-Q---
	Azotobacter beijerinckii	WP_090619428	------------R--TV-E-A		---I--KT-E---E-AAV------
	Entomomonas moraniae	WP_127163699	------M----K--EQV-PST		-QRIY-D-IEQ--KE-AL--QKN-
	Oblitimonas alkaliphila	WP_053102138	----I---K--L--EQQ-A-A		--CYH-A--P----LAAK--Q-D-

Figure-S39

Partial sequence alignments of UDP-N-acetylmuramoyl-L-alanine--D-glutamate ligase showing a 5aa Ins (highlighted), which is uniquely shared by all species from the Linyingensis clade..

Group	Species	Accession	272		311
**Linyingensis Clade (*Geopseudomonas* gen. nov.) (6/6)**	*Pseudomonas linyingensis*	WP_090305376	AFANPDAAELLKAKLNG	L	VGTAPVFISSVVHNQQVLHRVR
	Pseudomonas guangdongensis	WP_090213654	-------------R---	M	--A------P--R---------
	Pseudomonas oryzae	WP_090349479	---------------I-	V	------------R---------
	Pseudomonas sagittaria	WP_092427653	-----------------	-	----------------------
	"Pseudomonas aromaticivorans"	MBV2133688	-----------------	-	----------------------
	Pseudomonas oryzagri	WP_229602992	---------------I-	V	----------------M-----
**Other *Pseudomonas***	*Pseudomonas abietaniphila*	WP_074757008	-----------RS--S-		MVR----V--IAR---T-Y---
	Pseudomonas aeruginosa	WP_003100310	---------------S-		-TA---------R---I-----
	Pseudomonas aestus	WP_022641203	-----------RS--SS		MVS-------I-R---T-----
	Pseudomonas agarici	WP_017131415	-----------RS--SS		MVR----V--I-R---T-----
	Pseudomonas alcaligenes	WP_021701846	---------------SE		TVP-K-------R---I-----
	Pseudomonas alcaliphila	WP_075749750	------------S--SQ		TSSV--------RD--I-----
	Pseudomonas alkylphenolica	WP_038606553	-----------RS--S-		MVS-------I-R---T-----
	Pseudomonas amygdali	WP_044318451	-----------RS--S-		MVR----V--IAR---T-Y---
	Pseudomonas anguilliseptica	WP_090381363	-----------RS--SE		TVA----V----R---I-----
	Pseudomonas antarctica	WP_064454281	-----------RS--S-		MVN-------I-R---T-----
	Pseudomonas argentinensis	WP_074881348	------------S--SE		SVS-----N---R---I-----
	Pseudomonas arsenicoxydans	WP_090179468	-----------RS--S-		MVS-------I-R---T-----
	Pseudomonas asiatica	WP_013974354	-----------RS--SS		MVS-------I-R---T-----
	Pseudomonas asplenii	WP_090202352	-----------RS--SS		MVK----V--I-R---T-----
	Pseudomonas asturiensis	WP_073168657	-----------RS--S-		MVR----V--IAR---T-Y---
	Pseudomonas asuensis	WP_188864515	------------D--SE		LTA---------R---T-----
	Pseudomonas atacamensis	WP_016773027	-----------RS--S-		MVS-------I-R---T-----
	Pseudomonas avellanae	WP_003376554	-----------RS--S-		MVR----V--IAR---T-Y---
	Pseudomonas azotifigens	WP_028238631	------------D--AS		LVS----V---T----------
	Pseudomonas azotoformans	WP_033896438	-----------RS--S-		MVN-------I-R---T-----
	Pseudomonas baetica	WP_095188447	-----------RS--S-		MVS-------I-R---T-----
	Pseudomonas balearica	WP_043222159	------------D--A-		-V-----V--------I-----
	Pseudomonas batumici	WP_040071178	-----------RS--SS		MVN----V-PI-R---T-----
	Pseudomonas bauzanensis	WP_074780345	---SDQ---Q-RSQ-Q-		MVS----VTPIEQDSRT-----
	Pseudomonas benzenivorans	WP_090445217	---------------SE		TVS---------R---I-----
	Pseudomonas bohemica	WP_110947857	-----------RS--S-		MVR----V--IAR---T-Y---
	Pseudomonas borbori	WP_090499020	---------------SD		TVA---------R---I-----
	Pseudomonas brassicacearum	WP_025215646	-----------RS--SS		MVS-------I-R---T-----
	Pseudomonas brenneri	WP_032861700	-----------RS--S-		MVS-------I-R---T-----
	Pseudomonas bubulae	WP_094999435	-----------RS--S-		MVST------I-R---T-----
	Pseudomonas caeni	WP_022966384	--------Q--RD--ST		LTS-----N---R---------
	Pseudomonas canadensis	WP_028618072	-----------RS--S-		MVN-------I-R---T-----
	Pseudomonas cannabina	WP_007250891	-----------RS--S-		MVR----V--IAR---T-Y---
	Pseudomonas capeferrum	WP_033701718	-----------RS--ST		MVS-------I-R---T-----
	Pseudomonas caricapapayae	WP_055007673	-----------RS--S-		MVR----V--IAR---T-Y---
	Pseudomonas carnis	WP_032895156	-----------RS--S-		MVS-------I-R---T-----
	Pseudomonas caspiana	WP_087267634	-----------RS--S-		MVR----V--IAR---T-Y---
	Pseudomonas cedrina	WP_076952667	-----------RS--S-		MVN-------I-R---T-----
	Pseudomonas cerasi	WP_065350613	-----------RS--S-		MVR----V--IAR---T-Y---
	Pseudomonas chengduensis	WP_079782756	------------S--SQ		TSSV--------RD--I-----
	Pseudomonas chloritidismutans	WP_014818958	--------Q---D--S-		-VS-------------T-----
	Pseudomonas chlororaphis	WP_028683489	-----------RS--S-		MVS-------I-R---T-----
	Pseudomonas cichorii	WP_025261968	-----------RS--SA		MVR----V--IAR---T-Y---
	Pseudomonas citronellolis	WP_074981965	-----------RD-IS-		MTS-Q-------V-K-T-----
	Pseudomonas coleopterorum	WP_056843999	-----------RS--SS		MVS--A-T-QIAV---TFY---
	Pseudomonas composti	WP_074941456	------------S--SQ		TSSV--------RD--I-----
	Pseudomonas congelans	WP_010427251	-----------RS--S-		MVR----V--IAR---T-Y---
	Pseudomonas coronafaciens	WP_005888728	-----------RS--S-		MVR----V--IAR---T-Y---
	Pseudomonas corrugata	WP_024776644	-----------RS--SS		MVS-------I-R---T-----
	Pseudomonas costantinii	WP_071485439	-----------RS--S-		MVN-------I-R---T-----
	Pseudomonas cremoricolorata	WP_038411854	-----------RS--ST		MVS-------I-R---T-----
	Pseudomonas cremoris	WP_185708610	-----------RS--S-		MVN-------I-R---T-----
	Pseudomonas cuatrocienegasensi	WP_069521177	---------------SD		TVS---------R---I-----
	Pseudomonas daroniae	WP_131190445	---------------SE		SV------N---R---I-----
	Pseudomonas deceptionensis	WP_048359373	-----------RS--S-		MVS-------I-R---T-----
	Pseudomonas delhiensis	WP_089391245	-----------RD-IS-		MTS-Q-------V-R-T-----
	Pseudomonas denitrificans	WP_023115084	---------------S-		-TA---------R---I-----
	Pseudomonas donghuensis	WP_010222899	-----------RS--SS		MVS-------I-R---T-----
	Pseudomonas dryadis	WP_131174921	---------------SE		SVN-----N---R---I-----
	Pseudomonas duriflava	WP_145141396	------------D--SD		LTA---------R---T-----
	Pseudomonas edaphica	WP_169987391	-----------RS--S-		MVS-------I-R---T-----
	Pseudomonas endophytica	WP_055102801	-----------RS--SS		MVS-------I-R---T-----
	Pseudomonas entomophila	WP_011535841	-----------RS--ST		MVS-------I-R---T-----
	Pseudomonas extremaustralis	WP_010562458	-----------RS--SS		MVN-------I-R---T-----
	Pseudomonas extremorientalis	WP_071489892	-----------RS--SS		MVN-------I-R---T-----
	Pseudomonas ficuserectae	WP_002555332	-----------RS--S-		MVR----V--IAR---T-Y---
	Pseudomonas fildesensis	WP_048724654	-----------RS--S-		MVN-------I-R---T-----
	Pseudomonas flavescens	WP_084304401	---------------SE		SVS-----N---R---I-----
	Pseudomonas flexibilis	WP_039559435	-----------R-Q-SE		TVSV----------R-------
	Pseudomonas floridensis	WP_083184912	-----------RS--S-		MVR----V--IAR---T-Y---
	Pseudomonas fluorescens	WP_053258175	-----------RS--S-		MVN-------I-R---T-----
	Pseudomonas fluvialis	WP_093984339	---------------SA		AVS---L-----R---I-----
	Pseudomonas formosensis	WP_090538306	--SSDQ---Q-RSRVQ-		MVQT---V-P-QVDSRT-----
	Pseudomonas fragi	WP_016779264	-----------RS--S-		MVST------I-R---T-----
	Pseudomonas frederiksbergensis	WP_071552056	-----------RS--S-		MVS-------I-R---T-----
	Pseudomonas fulva	WP_013792708	------------D--SQ		TVS-----N--AR---I-----
	Pseudomonas furukawaii	WP_004420348	---------------S-		TVS---------R---I-----
	Pseudomonas fuscovaginae	WP_010446719	-----------RS--SS		MVK----V--I-R---T-----
	Pseudomonas gessardii	WP_076964195	-----------RS--S-		MVS-------I-R---T-----
	Pseudomonas gingeri	WP_017123350	-----------RS--SS		MVK----V--I-R---T-----

Other *Pseudomonas*

Pseudomonas graminis	WP_074892713	-----------RS--S-	MVR----V--IAR---T-Y---
Pseudomonas granadensis	WP_090286611	-----------RS--S-	MVS-------I-R---T-----
Pseudomonas grimontii	WP_090401913	-----------RS--SS	MVS-------I-R---T-----
Pseudomonas guariconensis	WP_090346794	-----------R---ST	MVS-------I-R---T-----
Pseudomonas guguanensis	WP_090433060	------------S--SQ	TSSV--------RD--I-----
Pseudomonas guineae	WP_090243335	-----------RS--SE	TVA----V----R---I-----
Pseudomonas haemolytica	WP_153872569	-----------RS--S-	MVS-------I-R---T-----
Pseudomonas helleri	WP_048367643	-----------RS--S-	MVS-------I-R---T-----
Pseudomonas helmanticensis	WP_134177808	-----------RS--S-	MVS-------I-R---T-----
Pseudomonas humi	WP_069862115	-----------RD-IS-	MTS-Q-------V-K-T-----
Pseudomonas hunanensis	WP_003249731	-----------R---ST	MVN-------I-R---T-----
Pseudomonas hussainii	WP_083394245	-----------R---SQ	QLNTN-LVAPLNQSV-T-----
Pseudomonas hydrolytica	WP_012019716	---------------TQ	TTSV--------RD--I-----
Pseudomonas hydrolytica	WP_017361987	------------S--TQ	TSSV--------RD--I-----
Pseudomonas indica	WP_090257200	------------D--SQ	AVN---------R---I-----
Pseudomonas indoloxydans	WP_108234600	------------S--SQ	TSSV--------RD--I-----
Pseudomonas inefficax	WP_133975649	-----------RS--SS	MVS-------I-R---T-----
Pseudomonas japonica	WP_042123723	-----------RS--S-	MVS-------I-R---T-----
Pseudomonas jessenii	WP_057713159	-----------RS--S-	MVS-------I-R---T-----
Pseudomonas jilinensis	WP_080049759	--SSNQ---Q-RQR-Q-	-VN-T--V-P-EQ-A-T-----
Pseudomonas jinjuensis	WP_084314240	--------------IGS	LTG--A-V----R---T-Y---
Pseudomonas juntendi	WP_054906037	-----------RS--ST	MVS-------I-R---T-----
Pseudomonas kairouanensis	WP_135291346	-----------RS--S-	MVN-------I-R---T-----
Pseudomonas khazarica	WP_080756950	------------D--SQ	TTSV--------RD--I-----
Pseudomonas kilonensis	WP_024619121	-----------RS--SS	MVS-------I-R---T-----
Pseudomonas kirkiae	WP_131185882	------------D--ST	MVS----V----L-E-------
Pseudomonas kitaguniensis	WP_058412897	-----------RS--S-	MVK-------I-R---T-----
Pseudomonas knackmussii	WP_052355161	-----------RE--S-	LTG-Q-------V-K-T-----
Pseudomonas koreensis	WP_016773027	-----------RS--S-	MVS-------I-R---T-----
Pseudomonas kribbensis	WP_085732950	-----------RS--S-	MVS-------I-R---T-----
Pseudomonas kunmingensis	WP_014818958	--------Q---D--S-	-VS-------------T-----
Pseudomonas kuykendallii	WP_090225959	------------E--ST	-V--------IAR---I-----
Pseudomonas lactis	WP_014720084	-----------RS--S-	MVS-------I-R---T-----
Pseudomonas laurentiana	WP_163936409	-----------RS--ST	MVN-------I-R---T-----
Pseudomonas laurylsulfatiphila	WP_104451329	-----------RS--S-	MVS-------I-R---T-----
Pseudomonas laurylsulfatovoran	WP_103396546	-----------RS--S-	MVS-------I-R---T-----
Pseudomonas leptonychotis	WP_136665471	---------------SE	TVA----V----R---I-----
Pseudomonas libanensis	WP_043047982	-----------RS--S-	MVS-------I-R---T-----
Pseudomonas lini	WP_038981381	-----------RS--S-	MVS-------I-R---T-----
Pseudomonas litoralis	WP_090273441	---STQ---Q-RRQ-QS	MVS----V-P-QL-SNT-----
Pseudomonas lundensis	WP_047273500	-----------RS--S-	MVS-------I-R---T-----
Pseudomonas lurida	WP_034109431	-----------RS--SS	MVN-------I-R---T-----
Pseudomonas lutea	WP_037017970	-----------RS--S-	MVR----V--IAR---T-Y---
Pseudomonas luteola	WP_074823020	------------D--SE	LTA---------R---T-----
Pseudomonas mandelii	WP_042932530	-----------RS--S-	MVS-------I-R---T-----
Pseudomonas marginalis	WP_064052193	-----------RS--S-	MVN-------I-R---T-----
Pseudomonas marincola	WP_090514702	------------D--ST	TVA---------Q--KI-----
Pseudomonas massiliensis	WP_040261586	-----------RS--ST	MVK--A-T-PTIR---T-Y---
Pseudomonas matsuisoli	WP_188983739	------------D--SQ	TTQ-----D--IR---T-----
Pseudomonas mediterranea	WP_047702697	-----------RS--SS	MVS-------I-R---T-----
Pseudomonas meliae	WP_002555332	-----------RS--S-	MVR----V--IAR---T-Y---
Pseudomonas mendocina	WP_012019716	---------------TQ	TTSV--------RD--I-----
Pseudomonas migulae	WP_084319709	-----------RS--S-	MVS-------I-R---T-----
Pseudomonas mohnii	WP_047533725	-----------RS--S-	MVST------I-R---T-----
Pseudomonas monteilii	WP_013974354	-----------RS--SS	MVS-------I-R---T-----
Pseudomonas moorei	WP_090325480	-----------RS--S-	MVST------I-R---T-----
Pseudomonas moraviensis	WP_065615618	-----------RS--S-	MVS-------I-R---T-----
Pseudomonas mosselii	WP_028690389	-----------RS--ST	MVS---------R---T-----
Pseudomonas mucidolens	WP_084376488	-----------RS--S-	MVS-------I-R---T-----
Pseudomonas multiresinivorans	WP_024763990	------------E-VG-	LTG-KS------V---T-Y---
Pseudomonas nabeulensis	WP_105698777	-----------RS--SS	MVN-------I-R---T-----
Pseudomonas nitritireducens	WP_193452651	------------E-VG-	LTG-KS------V---T-Y---
Pseudomonas nitrititolerans	WP_014854277	--------Q---D--S-	MTA----------Q--------
Pseudomonas nitroreducens	WP_024763990	------------E-VG-	LTG-KS------V---T-Y---
Pseudomonas nosocomialis	WP_138407055	------------S--SS	MVS--------T----------
Pseudomonas oleovorans	WP_150606631	------------D--SQ	TTSV--------RD--I-----
Pseudomonas orientalis	WP_057724899	-----------RS--S-	MVS-------I-R---T-----
Pseudomonas oryzihabitans	WP_059313892	------------D--SD	LTG-------T-R---T-----
Pseudomonas otitidis	WP_044410755	------------D--SS	MVA--------AR---I-----
Pseudomonas ovata	WP_056841868	-----------RS--ST	MVR----V--IAR---T-Y---
Pseudomonas palleroniana	WP_090365644	-----------RS--S-	MVR-------I-R---T-----
Pseudomonas panacis	WP_017848342	-----------RS--SS	MVN-------I-R---T-----
Pseudomonas panipatensis	WP_090261832	-----------RE-IS-	LTG-Q-------V-R-T-----
Pseudomonas parafulva	WP_028632754	-----------RS--SS	MVS-------I-R---T-----
Pseudomonas paralactis	WP_014720084	-----------RS--S-	MVS-------I-R---T-----
Pseudomonas peli	WP_090255590	---------------SE	TVA----V----R---I-----
Pseudomonas pertucinogena	WP_188635977	---SDQ---Q-RREIQ-	LVQ----V-P-EVDART-----
Pseudomonas pharmafabricae	WP_093984339	---------------SA	AVS---L-----R---I-----
Pseudomonas piscis	WP_022641203	-----------RS--SS	MVS-------I-R---T-----
Pseudomonas plecoglossicida	WP_013974354	-----------RS--SS	MVS-------I-R---T-----
Pseudomonas poae	WP_003237032	-----------RS--S-	MVN-------I-R---T-----
Pseudomonas pohangensis	WP_090192716	------------D--SQ	IAS--------TL---------
Pseudomonas prosekii	WP_092277631	-----------RS--S-	MVS-------I-R---T-----
Pseudomonas protegens	WP_011063667	-----------RS--S-	MVS-------I-R---T-----
Pseudomonas proteolytica	WP_029292258	-----------RS--S-	MVS-------I-R---T-----
Pseudomonas psychrophila	WP_019824215	-----------RS--S-	MVST------I-R---T-----
Pseudomonas psychrotolerans	WP_099049438	------------D--SD	LTG-------T-R---T-----
Pseudomonas psychrotolerans	WP_099049438	------------D--SD	LTG-------T-R---T-----
Pseudomonas pudica	WP_046613225	-----------RS--SS	MVS-------I-R---T-----
Pseudomonas punonensis	WP_073262046	------------D--SQ	AVS-----N--AR---I-----

Other *Pseudomonas*	*Pseudomonas putida*	WP_016501805	-----------RS--ST	MVS-------I-R---T-----
	Pseudomonas qingdaonensis	WP_043863902	-----------RS--S-	MVS-------I-R---T-----
	Pseudomonas reactans	WP_177004239	-----------RS--S-	MVN-------I-R---T-----
	Pseudomonas reidholzensis	WP_119138064	-----------RS--ST	MVS-------I-R---T-----
	Pseudomonas reinekei	WP_075949064	-----------RS--S-	MVS-------I-R---T-----
	Pseudomonas resinovorans	WP_016490734	---------------S-	TV----------R---I-----
	Pseudomonas rhizoryzae	WP_099049438	------------D--SD	LTG-------T-R---T-----
	Pseudomonas rhizosphaerae	WP_043192657	-----------RS--SS	MVS--A-T-QIAV---TFY---
	Pseudomonas rhodesiae	WP_034139421	-----------RS--SS	MVN-------I-R---T-----
	Pseudomonas salomonii	WP_065911238	-----------RS--S-	MVS-------I-R---T-----
	Pseudomonas saponiphila	WP_092309658	-----------RS--SS	MVS-------I-R---T-----
	Pseudomonas saudimassiliensis	WP_044500446	---SDQ---Q-RSQ-Q-	MVS----V-PIEVESRT-----
	Pseudomonas saudiphocaensis	WP_037023862	--------Q--RD--S-	MTG----V-----RE-------
	Pseudomonas savastanoi	WP_002555332	-----------RS--S-	MVR----V--IAR---T-Y---
	Pseudomonas saxonica	WP_122783226	-----------RS--S-	MVS---------R---T-----
	Pseudomonas sediminis	WP_099526365	------------S--SQ	TSSV--------RD--I-----
	Pseudomonas segetis	WP_089360537	------------D--S-	TV----------Q--RT-----
	Pseudomonas seleniipraecipitan	WP_070880765	---------------SE	SV------N---L---I-----
	Pseudomonas shirazica	WP_013974354	-----------RS--SS	MVS-------I-R---T-----
	Pseudomonas sichuanensis	WP_110993395	-----------RS--ST	MVS---------R---T-----
	Pseudomonas sihuiensis	WP_092377715	------------S--SQ	TSSV--------RD--I-----
	Pseudomonas silesiensis	WP_064679957	-----------RS--S-	MVS-------I-R---T-----
	Pseudomonas simiae	WP_010206787	-----------RS--S-	MVN-------I-R---T-----
	Pseudomonas sivasensis	WP_032884596	-----------RS--S-	MVN-------I-R---T-----
	Pseudomonas soli	WP_038707279	-----------RS--ST	MVS---------R---T-----
	Pseudomonas songnenensis	WP_122097915	--------Q---D--S-	-VS-------------T-----
	Pseudomonas straminea	WP_093504165	------------D--SQ	TVS-----N--AR---I-----
	Pseudomonas stutzeri	WP_014597832	--------Q---D--S-	-VS-------------T-----
	Pseudomonas synxantha	WP_057024454	-----------RS--S-	MVS-------I-R---T-----
	Pseudomonas syringae	WP_024647028	-----------RS--S-	MVR----V--IAR---T-Y---
	Pseudomonas taeanensis	WP_025167621	--------Q---D--S-	TV--------------I-----
	Pseudomonas taetrolens	WP_048378359	--V--------RS--S-	MVS---------R---T-----
	Pseudomonas taiwanensis	WP_023382526	-----------RS--ST	MVS-------I-R---T-----
	Pseudomonas thermotolerans	WP_026146327	---------------SE	TVN---------R---I-----
	Pseudomonas thivervalensis	WP_053125661	-----------RS--SS	MVS-------I-R---T-----
	Pseudomonas tolaasii	WP_016973915	-----------RS--S-	MVS-------I-R---T-----
	Pseudomonas toyotomiensis	WP_074916578	------------S--TQ	TSSV--------RD--I-----
	Pseudomonas tremae	WP_005888728	-----------RS--S-	MVR----V--IAR---T-Y---
	Pseudomonas trivialis	WP_049711373	-----------RS--S-	MVK-------I-R---T-----
	Pseudomonas tuomuerensis	WP_039606552	-----------R-Q-SE	TVSV----------R-------
	Pseudomonas typographi	WP_190423706	-----------RS--GS	MVK--A-T-P--R---T-Y---
	Pseudomonas umsongensis	WP_033044785	-----------RS--S-	MVKT------I-R---T-----
	Pseudomonas vancouverensis	WP_093229873	-----------RS--S-	MVS-------I-R---T-----
	Pseudomonas veronii	WP_017848342	-----------RS--SS	MVN-------I-R---T-----
	Pseudomonas versuta	WP_060696815	-----------RS--S-	MVS---------R---T-----
	Pseudomonas viridiflava	WP_004885844	-----------RS--S-	MVR----V--IAR---T-Y---
	Pseudomonas vranovensis	WP_028944404	-----------RS--S-	MVS---------R---T-----
	Pseudomonas wadenswilerensis	WP_115085160	-----------RS--SS	MVS---------R---T-----
	Pseudomonas weihenstephanensis	WP_048362374	-----------RS--S-	MVS-------I-R---T-----
	Pseudomonas xanthomarina	WP_073298983	--------Q--RD--SS	MTG----V-----QE-I-----
	Pseudomonas yamanorum	WP_063026906	-----------RS--S-	MVS-------I-R---T-----
	Pseudomonas zeshuii	WP_010797098	------------D--SE	LTA---------R---T-----
	Pseudomonas zhaodongensis	WP_128120702	--------Q--RD--SS	LTE----V-----QE-I-----
Other Bacteria	*Azomonas agilis*	WP_144572037	--------Q---DR-S-	MV-----------RE-------
	Azomonas macrocytogenes	WP_183165511	------------D--S-	MVS---------R----F----
	Azotobacter beijerinckii	WP_090623378	------------D--SR	ISAT-A-V----RDR-TF----
	Azotobacter chroococcum	WP_089169195	------------D--SR	ISAT-A-V-P--RDR-TF----
	Azotobacter salinestris	WP_152388632	------------D--SR	ISST---V-P--RDR-TF----
	Azotobacter vinelandii	WP_012699512	------------D--SR	ISST---V-P--RDR-TF----
	Candidatus Pseudomonas adelges	WP_129210840	----Q---K--RF--S-	IVN-----GPIMR---I-YC--
	Entomomonas moraniae	WP_127164036	-----------RQR-M-	-T-T----NA-AQGKTQ-Y---
	Oblitimonas alkaliphila	WP_053102212	--------Q--RD--AQ	HVEV--EV--IARDN-------
	Thiopseudomonas denitrificans	WP_101496445	--------Q--RD--GA	-TASQ-KV----RD--------

Figure-S40

Partial sequence alignments of Septal ring lytic transglycosylase RlpA family protein showing 1aa Ins (highlighted), which are uniquely shared by all species from the Linyingensis clade.

			107		142
**Linyingensis Clade (*Geopseudomonas* gen. nov.) (6/6)**	*Pseudomonas linyingensis*	WP_090305710	PYAILVSPLLVES	A	AQRQMSQRVLVVDVPEQVQIQR
	Pseudomonas guangdongensis	WP_090211535	-------------	E	Q--R-VE-I--I----AL-L--
	Pseudomonas oryzae	WP_090351171	-------------	V	P----T-----------L-VE-
	Pseudomonas sagittaria	WP_092427422	-------------	-	--------------------E-
	"Pseudomonas aromaticivorans"	WP_217682927	-------------	T	---E-AR-------------H-
	Pseudomonas oryzagri	WP_229605567	-------------	-	P--Q-AQ------A---L--E-
**Other *Pseudomonas***	*Pseudomonas profundi*	WP_150299527	A--L---------		GRY--T----------EI--A-
	Pseudomonas abietaniphila	WP_074752124	----------L-T		S-H--V-----I----A---E-
	Pseudomonas abyssi	WP_096006520	---L---------		G-NNLTS--I------AM-LA-
	Pseudomonas aeruginosa	WP_003094654	-------------		G----T-------T--HL-L--
	Pseudomonas aestus	WP_022641060	-------------		G-AR-T--I--I-A-Q-L--E-
	Pseudomonas aestusnigri	WP_088277638	---L---------		G-NTLTK-II------AM-LA-
	Pseudomonas agarici	WP_060783270	----------I--		G-YATT--L-II-A-QTL-V--
	Pseudomonas alcaligenes	WP_061905376	-------------		G-HRIT-------A---L-L--
	Pseudomonas alcaliphila	WP_075745449	-------------		G----T--I----T-AEL-V--
	Pseudomonas alkylphenolica	WP_038606931	---VF----M---		G-Y-RT--L--I-A-QEL----
	Pseudomonas allii	WP_058423062	----------I--		G-HS-T--I--I---QSL----
	Pseudomonas amygdali	WP_044319939	----------I--		G-YR-V--L--I---Q----E-
	Pseudomonas anguilliseptica	WP_090381713	-------------		G-HKLT--I----A---L-V--
	Pseudomonas antarctica	WP_064450652	----------I--		G-YS-T--I--I---QSL----
	Pseudomonas argentinensis	WP_074881499	----T-----I--		G-YKLA--I--------L--E-
	Pseudomonas arsenicoxydans	WP_090179852	----------I--		G-YA-T-----I-A---L----
	Pseudomonas asiatica	WP_100413056	---VY-----I--		G-Y-KT-----I-A-QEL-VA-
	Pseudomonas asplenii	WP_090202192	----------I--		G-YATT-----I-A-QAL-M--
	Pseudomonas asturiensis	WP_073168487	----------I--		G-YRTV--L--I---QSL--E-
	Pseudomonas asuensis	WP_188864422	----------I--		G-TKLT-----I----TL-LE-
	Pseudomonas atacamensis	WP_130887103	----------I--		G-YA-T--I--I-A-Q-L--E-
	Pseudomonas avellanae	WP_005614790	----------I--		G-SR-V--L--I---Q----E-
	Pseudomonas azotifigens	WP_028240304	---V------I--		G-YR-T----------HL-L--
	Pseudomonas azotoformans	WP_033903342	----------I--		G-YS-T--I--I---QSL----
	Pseudomonas baetica	WP_095189302	----------I--		G-YA-T--I--I-A-Q-L--E-
	Pseudomonas balearica	WP_043221694	---V---------		--HEQVD---------DL-L--
	Pseudomonas batumici	WP_040071044	-------------		G-YKNT-----I-A-QAL----
	Pseudomonas bauzanensis	WP_074777786	---L---------		G-Y--T----------AL--A-
	Pseudomonas benzenivorans	WP_090448068	----------I--		G-HRLA-------A--EL-V--
	Pseudomonas bohemica	WP_110946413	----------L-T		S-DR-V-----I---QAL--E-
	Pseudomonas borbori	WP_090498766	-------------		G-HRLA-----I-A--HL-V--
	Pseudomonas brassicacearum	WP_025215528	----------I--		G-YT-T--I--I-A---L--E-
	Pseudomonas brassicae	WP_163940975	---VF----M---		G-YKRC--L--I-A-QAL--E-
	Pseudomonas brenneri	WP_065944237	----------I--		G-HT-T--I--I---QST----
	Pseudomonas bubulae	WP_095018262	----------I--		G-YTTT-----I-A-QAL-M--
	Pseudomonas caeni	WP_022966049	----------I--		G-YKTV--I------SEL-VA-
	Pseudomonas canadensis	WP_028618793	----------I--		G-YS-T--I--I---QSL----
	Pseudomonas cannabina	WP_055000554	---V------I--		G-SR-V--L--I---R----E-
	Pseudomonas capeferrum	WP_033701427	---VY-----I--		G-YRKT-----I-A-QAL-ME-
	Pseudomonas caricapapayae	WP_055007785	-------------		G-YR-V--L--I-A-AHL--E-
	Pseudomonas carnis	WP_057976744	----------I--		G-YS-T--I--I---QSL----
	Pseudomonas caspiana	WP_087274558	-------------		G-WKTT-----I---Q---V--
	Pseudomonas cedrina	WP_076951107	----------I--		G-YS-T--I--I---QSL----
	Pseudomonas cerasi	WP_058397527	-------------		G-YR-V--L--I-A-PHL--E-
	Pseudomonas chengduensis	WP_059390436	-------------		G----T--I----T-AEL-V--
	Pseudomonas chlororaphis	WP_009045669	----------I--		G-YA-T--I--I---Q-L--E-
	Pseudomonas cichorii	WP_025261833	----------I--		G-YK-V-----I-A--NL--E-
	Pseudomonas citronellolis	WP_074977502	-------------		G-HR-A-------T--HL-LE-
	Pseudomonas composti	WP_074936159	-------------		G----T-------T-AEL-V--
	Pseudomonas congelans	WP_054992648	-------------		G-YR-V--L--I-A-AHL--E-
	Pseudomonas coronafaciens	WP_005895649	----------I--		G-SR-VK-L--I-A-QRL--E-
	Pseudomonas corrugata	WP_055135082	----------I--		G-YA-TH-I--I-A--PL--E-
	Pseudomonas costantinii	WP_071485901	----------I--		G-HS-T-----I---QSL----
	Pseudomonas cremoricolorata	WP_038410888	---VY-----I--		G-YAKT--I--I-A-HEL-L--
	Pseudomonas cremoris	WP_185708301	-------------		G-HS-T--I--I---QSL----
	Pseudomonas cuatrocienegasensi	WP_069522555	-------------		G-QALT-------A--HL-L--
	Pseudomonas daroniae	WP_131190543	----------I--		G-YKLT-----------L--E-
	Pseudomonas deceptionensis	WP_082136237	----------I--		G-YTTT-----I-A-QAL----
	Pseudomonas delhiensis	WP_089390517	-------------		G-HR-A-------T--HL-LE-
	Pseudomonas denitrificans	WP_003094654	-------------		G----T-------T--HL-L--
	Pseudomonas dryadis	WP_131175949	--S-------I--		G-YA-T-------A--HL-V--
	Pseudomonas duriflava	WP_145141599	----------I--		G-I--T-----I-A--AL-LE-
	Pseudomonas edaphica	WP_017134508	----------I--		G-YS-T--I--I---QSL----
	Pseudomonas endophytica	WP_055101753	----------I--		G-YTTT-----I-A-QTL----
	Pseudomonas entomophila	WP_011535704	---VY-----I--		G-FSKT--I--I-A-TEL-V--
	Pseudomonas extremaustralis	WP_010565575	----------I--		G-SS-T--I--I---QSL----
	Pseudomonas extremorientalis	WP_071491080	----------I--		G-YS-T--I--I---QSL----
	Pseudomonas ficuserectae	WP_054995405	----------I--		G-YR-V--L--I-T-AHL--E-
	Pseudomonas fildesensis	WP_048724165	----------I--		G-YS-T--I--I---QSL----
	Pseudomonas flavescens	WP_084305073	----------I--		G-YKLT----------PL--E-
	Pseudomonas flexibilis	WP_027591057	-----E----I--		G-S-LC-----I----A---S-
	Pseudomonas floridensis	WP_083182529	----------I--		G-YR-V-----I-A-Q-L--E-
	Pseudomonas fluorescens	WP_053254338	----------I--		G-YS-T--I--I---QSL----
	Pseudomonas fluvialis	WP_093985944	-----E-------		G-LR-T--L----S--AL--E-
	Pseudomonas formosensis	WP_090540472	---L---------		G-Y--TR---------AL--A-
	Pseudomonas fragi	WP_016779445	----------I--		G-YTTT-----I-A-QAL----
	Pseudomonas frederiksbergensis	WP_071553272	----------I--		G-YA-T-----I-A--RL--E-

Other *Pseudomonas*

Pseudomonas fulva	WP_013792873	----M-----I--	G-YKLT--I--------L--E-
Pseudomonas furukawaii	WP_003452481	----------I--	G-HR-T----L--A--SA----
Pseudomonas fuscovaginae	WP_019361534	----------I--	G-YATT-----I-A-QAL-V--
Pseudomonas gallaeciensis	WP_096006520	---L---------	G-NNLTS--I------AM-LA-
Pseudomonas gessardii	WP_076963598	----------I--	G-SA-T--I--I---QAT----
Pseudomonas gingeri	WP_017123508	----------I--	G-YATT-----I-A-QAL-V--
Pseudomonas graminis	WP_074888356	A---------L-T	S-H--V-----I----S---A-
Pseudomonas granadensis	WP_090286375	----------I--	G-YS-T--I--I-A-QPL--E-
Pseudomonas grimontii	WP_090409548	----------I--	G-YS-T--I--I---QSL----
Pseudomonas guariconensis	WP_043209638	----Y--------	G-IAKT-----I-A-QEL----
Pseudomonas guguanensis	WP_090427571	-------------	G----T-------T-AEL----
Pseudomonas guineae	WP_090240047	-------------	G-H-LT--I----A--HL--E-
Pseudomonas haemolytica	WP_153870150	----------I--	G-YS-T--I--I---QSL----
Pseudomonas helleri	WP_048367327	----------I-A	G-HKTT-----I-T-QAL----
Pseudomonas helmanticensis	WP_134177320	----------I--	G-YA-T--I--I-A-Q-L--E-
Pseudomonas humi	WP_009617624	-------------	G-HR-A-------T--HL-LE-
Pseudomonas hunanensis	WP_004575721	---VY-----I--	G-HHKT-----I-A-QDL--A-
Pseudomonas hussainii	WP_074869438	----------L-T	S-H--T----------AM--T-
Pseudomonas hydrolytica	WP_041976792	-------------	G----T-------T-VEL----
Pseudomonas indica	WP_084339420	-------------	G-HRLA-------A--HL-LA-
Pseudomonas indoloxydans	WP_108234288	-------------	G----T--I----T-AEL-V--
Pseudomonas inefficax	WP_112251900	---VY-----I--	G-Y-KT-----I-A-QEL-VA-
Pseudomonas jessenii	WP_046041800	----------I--	G-YA-T-----I-A---L--E-
Pseudomonas jilinensis	WP_119700444	---L---------	G-H--T--I-------AL--A-
Pseudomonas jinjuensis	WP_084313611	-------------	G-KR-TR------T--HL-LE-
Pseudomonas juntendi	WP_161892068	----Y-----I--	G-HHKT-----I-A-QAL-VA-
Pseudomonas kairouanensis	WP_135287351	----------I--	G-SG-T-----I---QSL----
Pseudomonas khazarica	WP_134676930	-------------	G--H-T--I----T-ADL-V--
Pseudomonas kilonensis	WP_033062721	S---------I--	G-YT-T--I--I-A---L--E-
Pseudomonas kirkiae	WP_131185710	----M-------A	GLHRRVD-L-------SL-LE-
Pseudomonas kitaguniensis	WP_058412748	-------------	G-HS-T--I--I-A-QSL----
Pseudomonas knackmussii	WP_043254953	-------------	G----TK-I----T--HL-LE-
Pseudomonas koreensis	WP_041477816	----------I--	G-YA-T--I--I-A-Q-L--E-
Pseudomonas kribbensis	WP_114885599	----------I--	G-YS-T--I--I-A-Q-L--E-
Pseudomonas kuykendallii	WP_090226288	----------I--	G-HRLT-------A---L-L--
Pseudomonas lactis	WP_003188438	----------I--	G-YS-T--I--I---QSL----
Pseudomonas laurylsulfatiphila	WP_104451417	----------I--	G-YA-T-----I-A---L--E-
Pseudomonas laurylsulfatovoran	WP_103396431	----------I--	G-YA-T-----I-A---L--E-
Pseudomonas leptonychotis	WP_136663131	----------I--	G-HKLTH------A---L----
Pseudomonas libanensis	WP_057013845	----------I--	G-YS-T--I--I---QSL----
Pseudomonas lini	WP_038981519	S---------I--	G-YA-T-----I-A---L--E-
Pseudomonas litoralis	WP_090271886	---L---------	G-Y--TR---------AL--A-
Pseudomonas lundensis	WP_047281317	-------------	G-HATT-----I-A-QAL----
Pseudomonas lurida	WP_069021704	----------I--	G-YS-T--I--I---QSL----
Pseudomonas lutea	WP_037015346	A---------L-T	S-H--V-----I----S---E-
Pseudomonas luteola	WP_074822820	---V------I--	G-T-LT----------AL-LE-
Pseudomonas mandelii	WP_042932554	----------I--	G-YA-T-----I-A---L--E-
Pseudomonas marginalis	WP_012722158	----------I--	G-YS-T--I--I---QSL----
Pseudomonas marincola	WP_090509568	-------------	G-SKLT--I----AS-TL--E-
Pseudomonas matsuisoli	WP_188983441	-------------	G-WRNV-------T--HL-L--
Pseudomonas mediterranea	WP_047698307	S---------I--	G-YA-T--I--I-A---L-VE-
Pseudomonas meliae	WP_005732328	----------I--	G-YR-V--L--I-T-AHL--E-
Pseudomonas mendocina	WP_011920935	----------I--	G--HLT-------T-VEL-V--
Pseudomonas migulae	WP_084319373	----------I--	G-HA-T-----I-A---L--E-
Pseudomonas mohnii	WP_047533366	----------I--	G-YA-T-----I-A---L--E-
Pseudomonas monteilii	WP_024086216	---VY-----I--	G-Y-KT-----I-A-QEL-VA-
Pseudomonas moorei	WP_090325229	----------I--	G-HA-T-----I-A---L--E-
Pseudomonas moraviensis	WP_042610481	----------I--	G-YA-T--I--I-A-Q-L--E-
Pseudomonas mosselii	WP_062575177	---VY-----I--	G-FSKT--I--I-A-TEL-V--
Pseudomonas mucidolens	WP_084376377	----------I--	G-YS-T--I--I---QSL----
Pseudomonas multiresinivorans	WP_169941843	-------------	G-HR-T-------T--HL-LE-
Pseudomonas nabeulensis	WP_105697432	----------I--	G-YS-T--I--I---QSL----
Pseudomonas nitritireducens	WP_170859556	-------------	G-HR-T-------T--HL-LE-
Pseudomonas nitrititolerans	WP_058075604	----------I-A	G-YRQAD---------SL-L--
Pseudomonas nitroreducens	WP_037011470	-------------	G-HR-T-------T--HL-LE-
Pseudomonas nosocomialis	WP_138407344	-------------	G-HRL---I--I----A--LE-
Pseudomonas oceani	WP_104737399	---L---------	G-NALTK--I------A--LS-
Pseudomonas oleovorans	WP_037046229	-------------	G--H-T--I----T-ADL-V--
Pseudomonas orientalis	WP_057726137	----------I--	G-YR-T--I--I---QSL----
Pseudomonas otitidis	WP_074973059	----------I--	G-HR-T----------AL-V--
Pseudomonas ovata	WP_109513926	----------I--	G-YKLAR----I---PE--LA-
Pseudomonas pachastrellae	WP_083725330	---L---------	G-NSLTS--I------TM-LA-
Pseudomonas palleroniana	WP_060754868	----------I--	G-YS-T--I--I---QSL----
Pseudomonas panacis	WP_017845471	----------I--	G-HS-T--I--I---QSL----
Pseudomonas panipatensis	WP_090267725	-------------	G--RLA--I----T--HL-LE-
Pseudomonas parafulva	WP_028631768	---VY-----I--	G-Y-KT-----I-A-HHL-VS-
Pseudomonas paralactis	WP_057701655	----------I--	G-YS-T--I--I---QSL----
Pseudomonas pelagia	WP_022962867	---L---------	G-HA-TE--I------AM--L-
Pseudomonas peli	WP_090249317	-------------	G-HKLT--I----AS-KL-V--
Pseudomonas pharmafabricae	WP_101194237	-----E-------	G-LR-T--L----S--AL--E-
Pseudomonas piscis	WP_085597063	-------------	G-AR-T--I--I-A-Q-L--E-
Pseudomonas plecoglossicida	WP_023660627	---VY-----I--	G-H-KT-----I-A-QEL-VA-
Pseudomonas poae	WP_004372354	----------I--	G-YS-T--I--I---QSL----
Pseudomonas pohangensis	WP_090192813	----------I--	G-H-LT--I----T--HL-V--

Other *Pseudomonas*	*Pseudomonas prosekii*	WP_092277879	----------I--	G-YA-T-----I-A-Q-L--E-
	Pseudomonas protegens	WP_011063523	----------I--	G-SR-T--I--I---Q-L--E-
	Pseudomonas proteolytica	WP_029291995	----------I--	G-YT-T--I--I---QST----
	Pseudomonas pseudoalcaligenes	WP_004425004	-------------	G----T--I----T-AEL-V--
	Pseudomonas psychrophila	WP_019823957	----------I--	G-YTTT-----I-A-QA-----
	Pseudomonas psychrotolerans	WP_074528524	----------F--	G--ALV--G--I----EL-LE-
	Pseudomonas psychrotolerans	WP_074528524	----------F--	G--ALV--G--I----EL-LE-
	Pseudomonas pudica	WP_112251900	---VY-----I--	G-Y-KT-----I-A-QEL-VA-
	Pseudomonas punonensis	WP_073262325	----TM----I--	G-YKLA--I-------H---E-
	Pseudomonas putida	WP_016501578	---VY-----I--	G-YHKT-----I-A-QEL--A-
	Pseudomonas qingdaonensis	WP_078479476	---VF----MI--	G-Y-RT--L--I-A-QAL--E-
	Pseudomonas reactans	WP_177002350	----------I--	G-YS-T--I--I---QSL----
	Pseudomonas reidholzensis	WP_119138316	---VY-----I--	G-HKKT-----I-A-QAL-V--
	Pseudomonas reinekei	WP_075948420	----------I--	G-YA-T-----I-A---L--E-
	Pseudomonas resinovorans	WP_016490894	----------I--	G-YR-T----L--A--AA-L--
	Pseudomonas rhizoryzae	WP_058777710	----------F--	G--ALV--G--I-A--EL-LE-
	Pseudomonas rhodesiae	WP_034111714	----------I--	G-YS-T--I--I---QSL--E-
	Pseudomonas sabulinigri	WP_092285479	---L---------	G-HRLTS--I------EL-LA-
	Pseudomonas salegens	WP_092384055	A--L---------	T--NLVR-I--I-A--AI--S-
	Pseudomonas salina	WP_150278172	---L---------	G-HK-TD-------S-ET-LM-
	Pseudomonas salomonii	WP_069786042	----------I--	G-YS-T--I--I---QSL----
	Pseudomonas saponiphila	WP_092309990	----------I--	G-SR-T--I--I---Q-L--E-
	Pseudomonas saudimassiliensis	WP_044501438	---L---------	G-Y--TR---------AL--A-
	Pseudomonas savastanoi	WP_011167725	----------I--	G-YR-V--L--I-T-AHL--E-
	Pseudomonas saxonica	WP_146384321	----------I--	G-HTTT-----I---QAL----
	Pseudomonas sediminis	WP_099522570	-------------	G----T--I----T-AEL-V--
	Pseudomonas segetis	WP_010489721	------T-----T	G-HALT-----I-A--TL--E-
	Pseudomonas seleniipraecipitan	WP_092370001	----M-----I--	G-YKLA-----I----H---E-
	Pseudomonas shirazica	WP_100413056	---VY-----I--	G-Y-KT-----I-A-QEL-VA-
	Pseudomonas sichuanensis	WP_110991742	---VY-----I--	G-FNKV--I----A-TEL-V--
	Pseudomonas sihuiensis	WP_004425004	-------------	G----T--I----T-AEL-V--
	Pseudomonas silesiensis	WP_064675851	----------I--	G-YA-T-----I-A---L--E-
	Pseudomonas simiae	WP_010213235	----------I--	G-YS-T--I--I---QSL----
	Pseudomonas sivasensis	WP_032886194	----------I--	G-YS-T--I--I---QS-----
	Pseudomonas soli	WP_038706809	---VY-----I--	G-H-KT--I--I-A-TEL-V--
	Pseudomonas songnenensis	WP_126189502	----M--------	G-YRQVG---------SL--A-
	Pseudomonas straminea	WP_093499899	----M-----I--	G-YKLT--I-------KL--E-
	Pseudomonas synxantha	WP_057021608	----------I--	G-YS-T--I--I---QSL----
	Pseudomonas syringae	WP_025390696	----------I--	G-SR-V--L--I---P-L--E-
	Pseudomonas taeanensis	WP_025166961	----------I--	G-HKLT-------A---L----
	Pseudomonas taetrolens	WP_048378221	----------I--	G-YSTT--I--I-A-QAL-V--
	Pseudomonas taiwanensis	WP_027907761	---VY-----I--	G-H-KT-----I-A-QAL-ME-
	Pseudomonas thermotolerans	WP_017939799	----------I--	G-HRTT-------A--AL----
	Pseudomonas thivervalensis	WP_053125330	S---------I--	G-YA-T-----I-A---L--E-
	Pseudomonas tolaasii	WP_080520061	----------I--	G-YS-T--I--I---QSL----
	Pseudomonas toyotomiensis	WP_021487990	-------------	G----T--I----T-AEL-V--
	Pseudomonas tremae	WP_005895649	----------I--	G-SR-VK-L--I-A-QRL--E-
	Pseudomonas trivialis	WP_049710056	----------I--	G-DS-T--I--I---QSL----
	Pseudomonas tuomuerensis	WP_039605990	-----E----I--	G-S-LC-----I----A---S-
	Pseudomonas typographi	WP_190417701	----YA------T	G--RGA-----I----AL-LA-
	Pseudomonas umsongensis	WP_083349764	----------I--	G-YA-T-----I-A---L--E-
	Pseudomonas vancouverensis	WP_093230163	----------I--	G-YA-T-----I-A---L--E-
	Pseudomonas veronii	WP_079442837	----------I--	G-HS-T--I--I---QSL----
	Pseudomonas versuta	WP_060690685	----------I--	G-YATT-----I-A-QAL--E-
	Pseudomonas vranovensis	WP_028944619	---VF----M---	G-F-RT--L--I-A-QEL----
	Pseudomonas weihenstephanensis	WP_048362482	-------------	G-HATT-----I-A-QAL----
	Pseudomonas xanthomarina	WP_073298748	----M--------	G-HHQVA---------AL-LE-
	Pseudomonas xinjiangensis	WP_093391479	---L------I--	G-HR-TE--I------SI-LA-
	Pseudomonas yamanorum	WP_017477788	----------I--	G-YT-T-----I---QSL----
	Pseudomonas yangmingensis	WP_093475169	---L---------	G-HRLTR----I---QAL--S-
	Pseudomonas zeshuii	WP_010797205	---V------I--	G-T-LT----------AL-LE-
	Pseudomonas zhaodongensis	WP_128120170	----M--------	G-Y-QVA---------AL-LE-
Other Bacteria	*Azomonas macrocytogenes*	WP_183167510	----------I--	G-Y-RM-------T--SL----
	Azotobacter beijerinckii	WP_090622202	-----E------T	D-HT-T--I-------SL-LE-
	Azotobacter chroococcum	WP_089167358	------------T	D-HK-T--I--I----PL-LE-
	Azotobacter salinestris	WP_152387088	------------T	D-HK-T--I--I----SL-LE-
	Azotobacter vinelandii	WP_012699851	----------L-T	D-HR-T--I--I----SL-LE-
	Candidatus Pseudomonas adelges	WP_129210992	--V-------I-L	G-YK-T--I--I-A-QSL----
	Oblitimonas alkaliphila	WP_053102855	----------I-T	Q-T-LC-----I----AL--S-

Figure-S41

Partial sequence alignments of the protein Dephospho-CoA kinase showing a 1aa Ins (highlighted), which are uniquely shared by all species from the Linyingensis clade.

Group	Species	Accession	413		445
Linyingensis Clade (*Geopseudomonas* gen. nov.) (6/6)	*Pseudomonas linyingensis*	WP_090308457	TAYHESGHAILGCLFP		SDPVHKVTIIPRGRALG
	Pseudomonas guangdongensis	WP_090213238	----------------		-----------------
	Pseudomonas oryzae	WP_090351638	----------------		-----------------
	Pseudomonas sagittaria	WP_092429387	----------------		-----------------
	"Pseudomonas aromaticivorans"	WP_217683414	----------------		-----------------
	Pseudomonas oryzagri	WP_229604111	----------------		-----------------
Other *Pseudomonas*	*Pseudomonas profundi*	WP_150301543	----------V-R-V-	E	H---Y--S---------
	Pseudomonas abyssi	WP_096006130	----------V-R-V-	E	H---Y------------
	Pseudomonas aestusnigri	WP_088276379	----------V-R-V-	E	H---Y------------
	Pseudomonas alcaligenes	WP_081693959	-----A----V-R-V-	E	H---Y--S---------
	Pseudomonas argentinensis	WP_074881317	-----A----V-R-V-	E	H---Y------------
	Pseudomonas azotifigens	WP_028241614	-----A----V-RVV-	E	H---Y--S---------
	Pseudomonas balearica	WP_043218753	-----A----V-RVV-	E	H---Y--S---------
	Pseudomonas bauzanensis	WP_074779735	----------V-R-V-	E	H---Y--S---------
	Pseudomonas caeni	WP_022965146	-----A----V-R-V-	E	H---Y--S---------
	Pseudomonas chloritidismutans	WP_023446165	-----A----V-RVV-	E	H---Y--S---------
	Pseudomonas cissicola	WP_007972066	-----A----V-R-V-	E	H---Y------------
	Pseudomonas duriflava	WP_145144760	-----A----V-R-V-	E	H---Y--S---------
	Pseudomonas flexibilis	WP_039560882	-----A----V-R-V-	E	H---Y--S---------
	Pseudomonas floridensis	WP_083182400	-----A----V-R-V-	E	H---Y--S---------
	Pseudomonas fluvialis	WP_093986051	-----A----V-R-V-	E	H---Y--S---------
	Pseudomonas formosensis	WP_090539830	-----A----V-R-V-	E	H---Y--S---------
	Pseudomonas furukawaii	WP_003457301	-----A----V-R-V-	E	H---Y--S---------
	Pseudomonas gallaeciensis	WP_118131723	----------V-R-V-	E	H---Y------------
	Pseudomonas geniculata	WP_057502978	-----A----V-R-V-	E	H---Y------------
	Pseudomonas guariconensis	WP_043210103	-----A----V-R-V-	E	H---Y--S---------
	Pseudomonas guguanensis	WP_090433515	-----A----V-R-V-	E	H---Y--S---------
	Pseudomonas guineae	WP_090243749	--F--A----V-R-V-	E	H---Y--S---------
	Pseudomonas hunanensis	WP_003249950	-----A----V-R-V-	E	H---Y--S---------
	Pseudomonas hussainii	WP_074867105	-----A----V-RIV-	E	H---Y--S---------
	Pseudomonas hydrolytica	WP_013716999	-----A----V-R-V-	E	H---Y--S---------
	Pseudomonas indica	WP_084337216	-----A----V-R-V-	E	H---Y--S---------
	Pseudomonas indoloxydans	WP_108235181	-----A----V-R-V-	E	H---Y--S---------
	Pseudomonas jilinensis	WP_119701786	----------V-R-V-	E	H---Y--S---------
	Pseudomonas juntendi	WP_009685343	-----A----V-R-V-	E	H---Y--S---------
	Pseudomonas khazarica	WP_037050830	-----A----V-R-V-	E	H---Y--S---------
	Pseudomonas kirkiae	WP_131183671	--F--A----V-R-V-	E	H---Y--S---------
	Pseudomonas kunmingensis	WP_041016119	-----A----V-RVV-	E	H---Y--S---------
	Pseudomonas kuykendallii	WP_090223623	-----A----V-R-V-	E	H---Y--S---------
	Pseudomonas litoralis	WP_090276090	----------V-R-V-	E	H---Y--S---------
	Pseudomonas luteola	WP_019366086	-----A----V-R-V-	E	H---Y--S---------
	Pseudomonas marincola	WP_069897982	-----A----V-N-V-	E	H---Y--S---------
	Pseudomonas matsuisoli	WP_188983793	-----A----V-R-V-	E	H---Y--S---------
	Pseudomonas mendocina	WP_036986839	-----A----V-R-V-	E	H---Y--S---------
	Pseudomonas nitrititolerans	WP_014853922	-----A----V-RVV-	E	H---Y--S---------
	Pseudomonas nosocomialis	WP_138409434	-----A----V-RVV-	E	H---Y--S---------
	Pseudomonas oceani	WP_104739303	----------V-R-V-	E	H---Y------------
	Pseudomonas oleovorans	WP_037050830	-----A----V-R-V-	E	H---Y--S---------
	Pseudomonas otitidis	WP_044413721	-----A----V-RIV-	E	H---Y--S---------
	Pseudomonas ovata	WP_056834018	-----A----V-R-V-	E	H---Y--S---------
	Pseudomonas pachastrellae	WP_083726912	----------V-R-V-	E	H---Y------------
	Pseudomonas pelagia	WP_022964567	----------V-R-V-	E	H---Y--S---------
	Pseudomonas pertucinogena	WP_188637188	----------V-R-V-	E	H---Y--S---------
	Pseudomonas pharmafabricae	WP_101194221	-----A----V-R-V-	E	H---Y--S---------
	Pseudomonas pohangensis	WP_090192989	-----A----V-R-V-	E	H---Y--S---------
	Pseudomonas punonensis	WP_073261985	-----A----V-R-V-	E	H---Y------------
	Pseudomonas resinovorans	WP_016494778	--F--A----V-R-V-	E	H---Y--S---------
	Pseudomonas sabulinigri	WP_092288518	----------V-R-V-	E	H---Y------------
	Pseudomonas salegens	WP_092389390	----------V-R-V-	D	H---Y--S---------
	Pseudomonas salina	WP_150278779	----------I-R-V-	E	H---Y--S---------
	Pseudomonas saudimassiliensis	WP_044501693	----------V-R-V-	E	H---Y--S---------
	Pseudomonas saudiphocaensis	WP_037026426	-----A----V-R-V-	E	H---Y--S---------
	Pseudomonas segetis	WP_089360431	-----A----V-R-V-	E	H---Y--S---------
	Pseudomonas songnenensis	WP_126188728	-----A----V-RVV-	E	H---Y--S---------
	Pseudomonas straminea	WP_093504138	-----A----V-R-V-	E	H---Y------------
	Pseudomonas stutzeri	WP_043942352	-----A----V-RVV-	E	H---Y--S---------
	Pseudomonas taeanensis	WP_025164688	--F--A----V-R-V-	E	H---Y--S---------
	Pseudomonas thermotolerans	WP_017937463	-----A----I-R-M-	E	H---Y------------
	Pseudomonas tuomuerensis	WP_039560882	-----A----V-R-V-	E	H---Y--S---------
	Pseudomonas xanthomarina	WP_073301293	-----A----V-RVV-	E	H---Y--S---------
	Pseudomonas xinjiangensis	WP_093397736	----------V-R-V-	E	H---Y--S---------
	Pseudomonas yangmingensis	WP_093477887	----------V-R-M-	E	H---Y--S---------
	Pseudomonas zeshuii	WP_073450668	-----A----V-R-V-	E	H---Y--S---------
Other Bacteria	*Atlantibacter hermannii*	WP_040459606	-----A----I-R-V-	E	H----------------
	Atlantibacter subterranea	WP_040459606	-----A----I-R-V-	E	H----------------
	Azomonas macrocytogenes	WP_183166947	-----A----V-RMV-	E	H---Y-IS---------
	Azotobacter beijerinckii	WP_090618842	-----A----V-R-V-	E	H---Y--S---------

Other Bacteria

Azotobacter chroococcum	WP_089167226	-----A----V-R-V- E H---Y--S---------
Azotobacter salinestris	WP_152389828	-----A----V-R-V- E H---Y--S---------
Azotobacter vinelandii	WP_012702779	-----A----V-R-V- E H---Y--S---------
Buttiauxella agrestis	WP_034498824	-----A----I-R-V- E H----------------
Buttiauxella brennerae	WP_064560989	-----A----I-R-V- E H----------------
Buttiauxella ferragutiae	WP_064543152	-----A----I-R-V- E H----------------
Buttiauxella gaviniae	WP_064514062	-----A----I-R-V- E H----------------
Buttiauxella izardii	WP_034459698	-----A----I-R-V- E H----------------
Buttiauxella noackiae	WP_034459698	-----A----I-R-V- E H----------------
Buttiauxella warmboldiae	WP_124023057	-----A----I-R-V- E H----------------
Candidatus Blochmannia chromai	WP_015344396	-----A--V-I-R-V- E H----------------
Candidatus Blochmannia florida	WP_041568935	-----A----I-R-V- E H----------------
Candidatus Blochmannia pennsyl	WP_011282650	-----A--V-I-R-V- E H----------------
Candidatus Blochmannia vafer	WP_044026117	-----A----I-R-V- E H----------------
Candidatus Doolittlea endobia	WP_067565459	-----A----I-R-L- E H----------------
Candidatus Hamiltonella defens	WP_100103563	-----A----I-R-V- E H----------------
Candidatus Hoaglandella endobi	WP_067567443	-----A----I-R-V- E H-----A----------
Candidatus Mikella endobia	WP_067568985	-----A----I-R-V- E H----------------
Candidatus Purcelliella pentas	WP_115956220	-----A----I-R-M- E H----------------
Candidatus Regiella insecticol	WP_176487847	-----A----I-R-V- E H------------H---
Candidatus Riesia	ARC54813.1	----------V-RVV- D H-------------T--
Candidatus Riesia pediculischa	WP_080626694	-----A----V-KVV- E Q--I--I-------S--
Candidatus Symbiopectobacteriu	WP_196906618	-----A----I-R-L- E H----------------
Candidatus Tachikawaea gelatin	WP_082018176	-----A----V-RIV- E H--I-------------
Cedecea colo	WP_167610067	-----A----I-R-V- E H----------------
Cedecea davisae	WP_016538369	-----A----I-R-V- E H----------------
Cedecea lapagei	WP_126354391	-----A----I-R-V- E H----------------
Cedecea neteri	WP_061276175	-----A----I-R-V- E H----------------
Citrobacter amalonaticus	WP_046488025	-----A----I-R-V- E H----------------
Citrobacter braakii	WP_019077783	-----A----I-R-V- E H----------------
Citrobacter cronae	WP_003025013	-----A----I-R-V- E H----------------
Citrobacter europaeus	WP_043017790	-----A----I-R-V- E H----------------
Citrobacter farmeri	WP_042325087	-----A----I-R-V- E H----------------
Citrobacter freundii	WP_003025013	-----A----I-R-V- E H----------------
Citrobacter gillenii	WP_115257388	-----A----I-R-V- E H----------------
Citrobacter koseri	WP_012135310	-----A----I-R-V- E H----------------
Citrobacter murliniae	WP_045448044	-----A----I-R-V- E H----------------
Citrobacter pasteurii	WP_005121306	-----A----I-R-V- E H----------------
Citrobacter portucalensis	WP_003025013	-----A----I-R-V- E H----------------
Citrobacter rodentium	WP_012908535	-----A----I-R-V- E H----------------
Citrobacter sedlakii	WP_042291851	-----A----I-R-V- E H----------------
Citrobacter werkmanii	WP_003025013	-----A----I-R-V- E H----------------
Citrobacter youngae	WP_006687880	-----A----I-R-V- E H----------------
Cosenzaea myxofaciens	WP_066749887	-----A----I-R-V- E H----------------
Cronobacter condimenti	WP_032984433	-----A----I-R-V- E H----------------
Cronobacter dublinensis	WP_004385065	-----A----I-R-V- E H----------------
Cronobacter malonaticus	WP_004385065	-----A----I-R-V- E H----------------
Cronobacter muytjensii	WP_038865499	-----A----I-R-V- E H----------------
Cronobacter sakazakii	WP_004385065	-----A----I-R-V- E H----------------
Cronobacter turicensis	WP_004385065	-----A----I-R-V- E H----------------
Cronobacter universalis	WP_004385065	-----A----I-R-V- E H----------------
Dickeya zeae	WP_029456646	-----A----I-R-V- E H----------------
Enterobacter aerogenes	YP_004591073	-----A----I-R-V- E H----------------
Enterobacter asburiae	WP_029739626	-----A----I-R-V- E H----------------
Enterobacter bugandensis	WP_013098921	-----A----I-R-V- E H----------------
Enterobacter cancerogenous	WP_006178765	-----A----I-R-V- E H----------------
Enterobacter chengduensis	WP_014833437	-----A----I-R-V- E H----------------
Enterobacter chuandaensis	WP_013098921	-----A----I-R-V- E H----------------
Enterobacter cloacae	WP_013098921	-----A----I-R-V- E H----------------
Enterobacter dykesii	WP_010436002	-----A----I-R-V- E H----------------
Enterobacter hormaechei	WP_014833437	-----A----I-R-V- E H----------------
Enterobacter huaxiensis	WP_119936841	-----A----I-R-V- E H----------------
Enterobacter kobei	WP_013098921	-----A----I-R-V- E H----------------
Enterobacter lignolyticus	WP_044611874	-----A----I-R-V- E H----------------
Enterobacter ludwigii	WP_025202925	-----A----I-R-V- E H----------------
Enterobacter massiliensis	WP_044179628	-----A----I-R-V- E H----------------
Enterobacter mori	WP_010436002	-----A----I-R-V- E H----------------
Enterobacter oligotrophicus	WP_014833437	-----A----I-R-V- E H----------------
Enterobacter quasihormaechei	WP_003861810	-----A----I-R-V- E H----------------
Enterobacter roggenkampii	WP_013098921	-----A----I-R-V- E H----------------
Enterobacter sichuanensis	WP_013098921	-----A----I-R-V- E H----------------
Enterobacter soli	WP_041162135	-----A----I-R-V- E H----------------
Enterobacter timonensis	WP_061707090	-----A----I-R-V- E H----------------
Enterobacter vonholyi	WP_111964963	-----A----I-R-V- E H----------------
Enterobacter wuhouensis	WP_010436002	-----A----I-R-V- E H----------------
Enterobacter xiangfangensis	WP_003861810	-----A----I-R-V- E H----------------
Erwinia teleogrylli	WP_058910840	-----A----I-R-V- E H----------------
Escherichia alba	WP_155107347	-----A----I-R-V- E H----------------

**Other Bacteria**	*Escherichia albertii*	WP_001107461	-----A----I-R-V- E H----------------
	Escherichia coli	NP_417645.1	-----A----I-R-V- E H----------------
	Escherichia fergusonii	WP_001107467	-----A----I-R-V- E H----------------
	Escherichia marmotae	WP_038355868	-----A----I-R-V- E H----------------
	Franconibacter daqui	WP_024559287	-----A----I-R-V- E H----------------
	Franconibacter helveticus	WP_024561924	-----A----I-R-V- E H----------------
	Franconibacter pulveris	WP_024559287	-----A----I-R-V- E H----------------
	Hafnia alvei	WP_025798844	-----A----I-R-V- E H----------------
	Klebsiella aerogenes	WP_015369514	-----A----I-R-V- E H----------------
	Klebsiella grimontii	WP_004854549	-----A----I-R-V- E H----------------
	Klebsiella huaxiensis	WP_112213564	-----A----I-R-V- E H----------------
	Klebsiella indica	WP_138362943	-----A----I-R-V- E H----------------
	Klebsiella michiganensis	WP_039288095	-----A----I-R-V- E H----------------
	Klebsiella oxytoca	WP_004854549	-----A----I-R-V- E H----------------
	Klebsiella pneumoniae	YP_005229027	-----A----I-R-V- E H----------------
	Klebsiella quasipneumoniae	WP_004206183	-----A----I-R-V- E H----------------
	Klebsiella quasivariicola	WP_004206183	-----A----I-R-V- E H----------------
	Klebsiella variicola	WP_008806637	-----A----I-R-V- E H----------------
	Kluyvera ascorbata	WP_035891115	-----A----I-R-V- E H----------------
	Kluyvera crysocrescens	WP_061281661	-----A----I-R-V- E H----------------
	Kluyvera georgiana	WP_064541920	-----A----I-R-V- E H----------------
	Kluyvera intermedia	WP_047372957	-----A----I-R-V- A H----------------
	Kluyvera intestini	WP_039077472	-----A----I-R-V- E H----------------
	Kluyvera sichuanensis	WP_185667810	-----A----I-R-V- E H----------------
	Kosakonia arachidis	WP_090121604	--F--A----I-R-V- E H----------------
	Kosakonia cowanii	WP_054802973	-----A----I-R-V- E H----------------
	Kosakonia oryzae	WP_043956395	-----A----I-R-V- E H----------------
	Kosakonia oryzendophytica	WP_061493015	-----A----I-R-V- E H----------------
	Kosakonia oryziphila	WP_090134842	-----A----I-R-V- E H----------------
	Kosakonia pseudosacchari	WP_086870090	-----A----I-R-V- E H----------------
	Kosakonia quasisacchari	WP_131407785	-----A----I-R-V- E H----------------
	Kosakonia radicincitans	WP_007369921	-----A----I-R-V- E H----------------
	Kosakonia sacchari	WP_017457344	-----A----I-R-V- E H----------------
	Leclercia adecarboxylata	WP_032614434	-----A----I-R-V- E H----------------
	Lelliottia amnigena	WP_064327572	-----A----I-R-V- E H----------------
	Lelliottia jeotgali	WP_095283561	-----A----I-R-V- E H----------------
	Lelliottia nimipressuralis	WP_013098921	-----A----I-R-V- E H----------------
	Mangrovibacter phragmitis	WP_064600601	-----A----I-R-V- E H----------------
	Mangrovibacter plantisponsor	WP_036104857	-----A----I-R-V- E H----------------
	Metakosakonia massiliensis	WP_044179628	-----A----I-R-V- E H----------------
	Moellerella wisconsensis	WP_047257512	-----A----I-H-M- E H------------Q---
	Morganella	WP_004234548	-----A----I-R-V- E H----------------
	Oblitimonas alkaliphila	WP_053101353	-----A----V-R-V- E H---Y--S---------
	Perlucidibaca aquatic	WP_068857439	-----A----V-R-T- E H---Y--S---------
	Perlucidibaca piscinae	WP_040568103	--F--A----V-R-V- E H---Y--S---------
	Permianibacter aggregans	WP_133593495	-----A----V-Y-L- K T---Y------------
	Photorhabdus asymbiotica	WP_015836111	-----A----I-R-V- E H----------------
	Photorhabdus heterorhabditis	WP_054480218	-----A----I-R-V- E H----------------
	Photorhabdus luminescens	WP_049585097	-----A----I-R-V- E H----------------
	Photorhabdus temperata	WP_021323302	-----A----I-R-V- E H----------------
	Phytobacter diazotrophicus	WP_039077472	-----A----I-R-V- E H----------------
	Phytobacter palmae	WP_085005585	-----A----I-R-V- A H----------------
	Phytobacter ursingii	WP_047372957	-----A----I-R-V- A H----------------
	Plautia stali symbiont	WP_010617343	-----A----I-R-V- E H----------------
	Pluralibacter gergoviae	WP_043080874	-----A----I-R-V- E H----------------
	Proteus hauseri	WP_064718628	-----A----I-R-V- E H----------------
	Proteus vulgaris	WP_036939358	-----A----I-R-V- E H----------------
	Providencia alcalifaciens	WP_006658017	-----A----V-Y-M- E H----------------
	Providencia rustigianii	WP_006814513	-----A----V-Y-M- E H----------------
	Pseudescherichia vulneris	WP_042390103	-----A----I-R-V- E H----------------
	Pseudocitrobacter faecalis	WP_108474971	-----A----I-R-V- E H----------------
	Pseudomonas fulva	WP_013792677	-----A----V-R-V- E H---Y------------
	Raoultella electrica	WP_141963303	-----A----I-R-V- E H----------------
	Raoultella ornithinolytica	WP_015585660	-----A----I-R-V- E H----------------
	Raoultella planticola	WP_015585660	-----A----I-R-V- E H----------------
	Raoultella terrigena	WP_041147400	-----A----I-R-V- E H----------------
	Rhizobacter gummiphilus NS21	WP_085751617	-----A---LVAR-L- K T-------V--------
	Salmonella bongori	WP_001107481	-----A----I-R-V- E H----------------
	Salmonella enterica	NP_462207.1	-----A----I-R-V- E H----------------
	Scandinavium goeteborgense	WP_125352451	-----A----I-R-V- E H----------------
	Shigella boydii	WP_001107475	-----A----I-R-V- E H----------------
	Shigella dysenteriae	YP_404841.1	-----A----I-R-V- E H----------------
	Shigella flexneri	NP_708977.1	-----A----I-R-V- E H----------------
	Shigella sonnei 53G	WP_001107467	-----A----I-R-V- E H----------------
	Shimwellia blattae	WP_014715748	-----A----I-R-V- E H----------------
	Shimwellia pseudoproteus	WP_199014680	-----A----I-R-V- E H----------------
	Siccibacter colletis	WP_031519030	-----A----I-R-V- E H----------------
	Siccibacter turicensis	WP_024562577	-----A----I-R-V- E H----------------

Other Bacteria	*Superficieibacter electus*	WP_103676716	-----A----I-R-V- E H----------------
	Thiopseudomonas denitrificans	WP_101497992	-----A----V-R-V- E H---Y--S---------
	Trabulsiella guamensis	WP_038160907	-----A----I-R-V- E H----------------
	Trabulsiella odontotermitis	WP_038160907	-----A----I-R-V- E H----------------
	Xenorhabdus bovienii	WP_012986971	-----A----I-R-V- E H----------------
	Xenorhabdus cabanillasii	WP_038261483	-----A----I-R-V- E H----------------
	Xenorhabdus doucetiae	WP_045968315	-----A----I-R-V- E H----------------
	Xenorhabdus eapokensis	WP_074022676	-----A----I-R-V- E H----------------
	Xenorhabdus hominickii	WP_069317204	-----A----I-R-V- E H----------------
	Xenorhabdus khoisanae	WP_047962390	-----A----I-R-V- E H----------------
	Xenorhabdus nematophila	WP_013183297	-----A----I-R-V- E H----------------
	Xenorhabdus poinarii	WP_045959949	-----A----I-R-V- E H----------------
	Xenorhabdus szentirmaii	WP_038237667	-----A----I-R-V- E H----------------
	Xenorhabdus thuongxuanensis	WP_074019935	-----A----I-R-V- E H----------------
	Yokenella regensburgei	WP_006818123	-----A----I-R-V- E H----------------

Figure- S42

Partial sequence alignments of a protein ATP-dependent zinc metalloprotease FtsH showing a 1aa Del (highlighted), which is uniquely shared by all species from the Linyingensis clade.

Group	Species	Accession	232		282
**Linyingensis Clade (*Geopseudomonas* gen. nov.) (6/6)**	*Pseudomonas linyingensis*	WP_090307056	LGKIDQQRYQSALAEAVEVRQQAASP	P	ELEAPYVAEMARAEIVGRFGSDAY
	Pseudomonas sagittaria	WP_092430157	--------------------------	-	--------------------G---
	Pseudomonas oryzae	WP_090349618	---------------T-DAH--VV--	-	----------------S---G---
	Pseudomonas guangdongensis	WP_090213444	--R--RL--EQ----T----A--IA-	-	QI-----------L--QY-G---
	"Pseudomonas aromaticivorans"	WP_217681990	----------T----T--AH---VR-	-	------------------------
	Pseudomonas oryzagri	WP_229606236	---------------T--AH--VVT-	-	----------------S-------
**Other *Pseudomonas***	*Pseudomonas saudiphocaensis*	WP_037025463	--T--EA-F-Q----E-DAAYHG---		------I-------M---------
	Pseudomonas xanthomarina	WP_073303057	-----ET---Q----V-DASYHGS--		--N---I-------M------T--
	Pseudomonas tuomuerensis	WP_039559170	---------LD----P----LHIPT-		--N-------V---M--LY--E--
	Pseudomonas flexibilis	WP_039559170	---------LD----P----LHIPT-		--N-------V---M--LY--E--
	Pseudomonas azotifigens	WP_028238203	--H---A---A----P-NA-YHV---		--H---I-------L---Y--E--
	Pseudomonas caspiana	WP_087271119	------SA-----S-PLNASYHVPT-		-VN---I-------M---Y-----
	Pseudomonas capeferrum	WP_033702010	------AS--T----PLNASYHVPT-		-VN---I-------M---Y-----
	Pseudomonas indica	WP_084332915	-----EAS--A----PIDASYHVPT-		--S-----------M---Y-----
	Pseudomonas putida	WP_041167931	------AS--T----PLNASYHVPT-		-VN---I-------M---Y-----
	Pseudomonas alcaligenes	WP_061903205	--A--KA--DA----PIDA-HHVQPI		--N---I-------M---Y-----
	Pseudomonas zhaodongensis	WP_128120279	---L-ET--HQ----L-DASYHGS--		--D---I-------M------A--
	Pseudomonas abietaniphila	WP_074751548	------NT--T----PINASYHVQ--		-VN---I-------M---Y--E--
	Pseudomonas bohemica	WP_110950926	------NT--T----PINASYHVQ--		-VN---I-------M---Y--E--
	Pseudomonas straminea	WP_093500336	--R---A---A----P-DASYHV-T-		-VS-----------M---Y-----
	Pseudomonas punonensis	WP_073262843	--R---A--ET----P-NASYH-PT-		-VQ-----------M---Y-----
	Pseudomonas segetis	WP_089359860	-----E----E----P-NASYHV-A-		--A---I-------M---Y-----
	Pseudomonas entomophila	WP_011531749	-----ESS--A----PLNASYHVPT-		-VN-----------M---Y-----
	Pseudomonas qingdaonensis	WP_100634294	-----EAS--A----PINASYHVPT-		-VN---I-------M---Y-----
	Pseudomonas donghuensis	WP_010222226	-----EAS--A----PINASYHVPT-		-VN---I-------M---Y-----
	Pseudomonas wadenswilerensis	WP_115089390	-----EAS--A----PINASYHVPT-		-VN---I-------M---Y-----
	Pseudomonas weihenstephanensis	WP_048364551	------AS--A-I--P-NASYHVPT-		-VN---I-------M---Y--E--
	Pseudomonas kuykendallii	WP_090225664	-----ET--KA----PIDA-YHVQT-		--A---I-------M------A--
	Pseudomonas gessardii	WP_076961718	------SA-E--V--PLNASYHVPT-		-IS---I-------M---Y-----
	Pseudomonas vranovensis	WP_028943925	-----EAS--A----PINASYHVPT-		-VS---I-------M---Y-----
	Pseudomonas otitidis	WP_074973680	------A--EA-IS-PIDASYHVPT-		-VN---I-------M---Y-----
	Pseudomonas mosselii	WP_084942062	-----EAS--A----PLNASYHVPT-		-VN-----------M---Y-----
	Pseudomonas alkylphenolica	WP_038615547	-----EAS--A----PINASYHVPT-		-VR---I-------M---Y-----
	Pseudomonas brassicae	WP_163950361	-----DAS--A----PINASYHVPT-		-VN---I-------M---Y-----
	Pseudomonas fragi	WP_016783184	------AS-EA-I--P-NASYHVPT-		-VS---I-------M---Y-----
	Pseudomonas cremoricolorata	WP_038411273	------AS--N----PLNASYHVPT-		-VY-----------M---Y-----
	Pseudomonas peli	WP_090249974	--R------KE----PIN-SYHVPT-		--V---I-------M---Y--E--
	Pseudomonas japonica	WP_042121211	-----EAS--A----PLNASYHVPT-		-VN---I-------M---Y-----
	Pseudomonas asturiensis	WP_073167463	------TA--A--S-PLNASYHVPT-		-VN---I-------M---Y-----
	Pseudomonas extremaustralis	WP_010563208	------AA-E--V--PLNASYHVPT-		-VN---I-------M---Y-----
	Pseudomonas rhodesiae	WP_034136580	------AA-E--V--PLNASYHVPT-		-VN---I-------M---Y-----
	Pseudomonas parafulva	WP_028632033	-----EAS--A----PLNASYHVPT-		-VN---I-------M---Y-----
	Pseudomonas synxantha	WP_057010652	------AA-E--V--PLNASYHVPT-		-VN---I-------M---Y-----
	Pseudomonas libanensis	WP_057010652	------AA-E--V--PLNASYHVPT-		-VN---I-------M---Y-----
	Pseudomonas paralactis	WP_057702496	------AA-E--V--PLNASYHVPT-		-VN---I-------M---Y-----
	Pseudomonas taiwanensis	WP_027907909	-----EAS--A----PLNASYHVPT-		-VN---I-------M---Y-----
	Pseudomonas hunanensis	WP_003249135	------AS-EA----PLNASYHVPT-		-VN---I-------M---Y-----
	Pseudomonas palleroniana	WP_090367275	------AA-E--V--PLNASYHVPT-		-VN---I-------M---Y-----
	Pseudomonas guineae	WP_090240346	--R--K---EE----PIN-SYHVST-		--A---I-------M---Y--E--
	Pseudomonas inefficax	WP_025340961	-----EAS--A----PLNASYHVPT-		-VN---I-------M---Y-----
	Pseudomonas guariconensis	WP_090343362	------AS-EA----PLNASYHVPT-		-VN---I-------M---Y-----
	Pseudomonas saxonica	WP_122785197	------AS-EA-I--P-DASYHVPT-		-VS---I-------M---Y--E--
	Pseudomonas shirazica	WP_139657893	-----EAS--A----PLNASYHVPT-		-VN---I-------M---Y-----
	Pseudomonas pudica	WP_046613531	-----EAS--A----PLNASYHVPT-		-VN---I-------M---Y-----
	Pseudomonas plecoglossicida	WP_023662592	-----EAS--A----PLNASYHVPT-		-VN---I-------M---Y-----
	Pseudomonas monteilii	WP_023662592	-----EAS--A----PLNASYHVPT-		-VN---I-------M---Y-----
	Pseudomonas lutea	WP_037016031	------NT--T--V-PINASYHVQ--		-VN---I-------M---Y--E--
	Pseudomonas sichuanensis	WP_110996379	-----DAS--A----PLNASYHVTPE		-VN-----------M---Y-----
	Pseudomonas brenneri	WP_090292793	------NA-ET-V--PLNASYHVPT-		-VS---I-------M---Y-----
	Pseudomonas laurentiana	WP_163937224	-----EAS-L-----PINASYHVPT-		-VN---I-------M---Y-----
	Pseudomonas nitrititolerans	WP_170910519	--S--EA-W-Q----P-DASYHG-T-		--N---I-------M------A--
	Pseudomonas anguilliseptica	WP_090380555	--R------EE----PIN-SYHVPT-		--V---I-------M---Y--E--
	Pseudomonas proteolytica	WP_029296059	------NA-ET-V--PLNASYHVPT-		-VS---I-------M---Y-----
	Pseudomonas bubulae	WP_130872295	------AS--A-I--P-NASYHVPT-		-VS---I-------M---Y--E--
	Pseudomonas viridiflava	WP_088236116	------TA--A--N-PLNASYHVPT-		-VN---I-------M---Y-----
	Pseudomonas argentinensis	WP_074882003	--R---A--EA----P-DASYHV-T-		-VS-----------M---Y-----
	Pseudomonas fuscovaginae	WP_010453639	-----ESA--T----PINASYHVPT-		-VN---I-------M---Y--E--
	Pseudomonas asplenii	WP_090202887	-----ESA--T----PINASYHVPT-		-VN---I-------M---Y--E--
	Pseudomonas massiliensis	WP_040260076	------AS-DT----PINASYHVPT-		-VN---I-------M------A--
	Pseudomonas helleri	WP_048366717	------AS-EA-I--P-NASYHVPT-		-VN---I-------M---Y--E--
	Pseudomonas furukawaii	WP_003453196	--R--EA---L-IN-PIDASYHVPT-		--A-----------M---Y-----
	Pseudomonas lundensis	WP_047281947	------AS-EA-I--P-NASYHVPT-		-VN---I-------M---Y--E--
	Pseudomonas endophytica	WP_055101873	------AS-EA-I--P-NASYHVPT-		-VN---I-------M---Y--E--
	Pseudomonas soli	WP_094011029	-----EAS--A--G-PLNASYHVPT-		-VN-----------M---Y-----
	Pseudomonas resinovorans	WP_016495124	--R--EA---Q-IN-PINASYHVPT-		--A---I-------M---Y-----
	Pseudomonas taetrolens	WP_048382892	------AS-EA-I--P-NATYHVPT-		-VS---I-------M---Y--E--
	Pseudomonas balearica	WP_043218296	--Y------EQ-I--SETA-YHG---		------I-------M---Y--A--
	Pseudomonas thermotolerans	WP_026146340	-----EA--RA--N-PIDASYHVP--		-IS-----------M---Y-----
	Pseudomonas marincola	WP_090510174	------A--DE-V--PIDATYHVQ--		--N-----------M---Y-----
	Pseudomonas tolaasii	WP_080520643	------AA-E--V--PLNASYHVTP-		-VN---I-------M---Y--E--
	Pseudomonas lurida	WP_098465622	------TA-E--I--PLNASYHVPT-		-VN---I-------M---Y--E--
	Pseudomonas graminis	WP_074890784	------NT--T--V-PINASYHVQ--		-VN---I-------M---Y--A--
	Pseudomonas versuta	WP_060695991	-----KAS-ET-I--P-NASYHVTP-		-VS---I-------M---Y--E--
	Pseudomonas allii	WP_179029687	------AA-E--I--PLNASYHVPT-		-VN---I-------M---Y--E--
	Pseudomonas extremorientalis	WP_071492369	------AA-E--I--PLNASYHVPT-		-VN---I-------M---Y--E--
	Pseudomonas canadensis	WP_028615417	------AA-E--I--PLNASYHVPT-		-VN---I-------M---Y--E--
	Pseudomonas kirkiae	WP_131185855	-----ET---Q----P-DATYHG-T-		--D-A-I------DM------E--
	Pseudomonas deceptionensis	WP_048360444	------AS-EA-I--P-NASYHVPT-		-VS---I-------M---Y--E--
	Pseudomonas sivasensis	WP_095186127	------AA-E--I--PLNASYHVPT-		-VN---I-------M---Y--E--
	Pseudomonas batumici	WP_040063664	-----EAA--A-IV-PINASYHVPT-		-VN---I-------M---Y-----
	Pseudomonas juntendi	WP_161892175	------AS-RA--D-PLNASYHVPT-		-VN-----------M---Y-----
	Pseudomonas psychrophila	WP_046809120	------AS-EA-I--P-NASYHVPT-		-VS---I-------M---Y--E--
	Pseudomonas floridensis	WP_083182549	------TA--A--S-PLNASYHVPT-		-VN---I-------M---Y--E--

**Other** ***Pseudomonas***	*Pseudomonas nitritireducens*	WP_170858832	--F-------A-IQ-P-GASYHVQT-	--A---I-------L-----G---
	Pseudomonas simiae	WP_010213762	------AA-E--I--PLNASYHVPT-	-VN---I-------M---Y--E--
	Pseudomonas orientalis	WP_057724425	------TA-E--V--PLNASYHVPT-	-VN---I-------M---Y--E--
	Pseudomonas reactans	WP_177001685	------AA-E--I--PLNASYHVPT-	-VN---I-------M---Y--E--
	Pseudomonas azotoformans	WP_061434985	------AA-E--I--PLNASYHVPT-	-VN---I-------M---Y--E--
	Pseudomonas fulva	WP_041705686	--R---A--EA----PMNASYHV-T-	-VS-----------M---Y-----
	Pseudomonas knackmussii	WP_043255943	--F---A---A-I--P-AASYHVP--	--N---I-------M---Y--N--
	Pseudomonas marginalis	WP_012721813	------AA-E--V--PLNASYHVPT-	-VN---I-------M---Y--E--
	Pseudomonas frederiksbergensis	WP_071552306	-----EAA--T-IN-PLNASYHVPT-	-VN---I-------M---Y-----
	Pseudomonas savastanoi	WP_011167464	------TA--T--N-PLNASYHVPT-	-VN---I-------M---Y--E--
	Pseudomonas flavescens	WP_084304633	--R---A--ET----P-DASYHVPT-	-VP-----------M---Y-----
	Pseudomonas ficuserectae	WP_010195507	------TA--T--N-PLNASYHVPT-	-VN---I-------M---Y--E--
	Pseudomonas meliae	WP_044343319	------TA--T--N-PLNASYHVPT-	-VN---I-------M---Y--E--
	Pseudomonas amygdali	WP_044317419	------TA--T--N-PLNASYHVPT-	-VN---I-------M---Y--E--
	Pseudomonas cannabina	WP_007248062	------TA--T--N-PLNASYHVPT-	-VN---I-------M---Y--E--
	Pseudomonas fluorescens	WP_053253951	------AA-E--V--PLNASYHVPT-	-VN---I-------M---Y--E--
	Pseudomonas salomonii	WP_065930761	------AA-E--V--PLNASYHVPT-	-VN---I-------M---Y--E--
	Pseudomonas haemolytica	WP_153871580	------AA-E--V--PLNASYHVPT-	-VN---I-------M---Y--E--
	Pseudomonas antarctica	WP_064450305	------AA-E--V--PLNASYHVPT-	-VN---I-------M---Y--E--
	Pseudomonas leptonychotis	WP_136663374	--R--K---EE----PIN-SYHVPT-	--A---I-------M---Y--E--
	Pseudomonas grimontii	WP_090400976	------GA-E--V--PLNASYHVPT-	-VN---I-------M---Y--E--
	Pseudomonas kairouanensis	WP_135289186	------AA-E--V--PLNASYHVPT-	-VN---I-------M---Y--E--
	Pseudomonas edaphica	WP_176991728	------AA-E--V--PLNASYHVPT-	-VN---I-------M---Y--E--
	Pseudomonas nosocomialis	WP_138406551	--S---A--EE----P-DAKYHF-A-	--N-A-I-------M---Y-----
	Pseudomonas yamanorum	WP_026078066	------AA-E--V--PLNASYHVPT-	-VN---I-------M---Y--E--
	Pseudomonas cremoris	WP_185704858	------AA-E--V--PLNASYHVPT-	-VN---I-------M---Y--E--
	Pseudomonas lactis	WP_047712396	------AA-E--V--PLNASYHVPT-	-VN---I-------M---Y--E--
	Pseudomonas nabeulensis	WP_135308064	------AA-E--V--PLNASYHVPT-	-VN---I-------M---Y--E--
	Pseudomonas trivialis	WP_049710408	------AA-E--V--PLNASYHVPT-	-VN---I-------M---Y--E--
	Pseudomonas carnis	WP_034125573	------AA-E--V--PLNASYHVPT-	-VN---I-------M---Y--E--
	Pseudomonas luteola	WP_074823154	--Y---RA-ET-V---IDAYHHV-V-	--A-----------M---Y-----
	Pseudomonas kitaguniensis	WP_152747478	------AA-E--V--PLNASYHVTP-	-VN---I-------M---Y--E--
	Pseudomonas cedrina	WP_076951745	------AA-E--V--PLNASYHVPT-	-VN---I-------M---Y--E--
	Pseudomonas poae	WP_015373578	------AA-E--V--PLNASYHVPT-	-VN---I-------M---Y--E--
	Pseudomonas mucidolens	WP_084379838	------NT-ET-V--PLNASYHVQT-	-VS---I-------M---Y--E--
	Pseudomonas caricapapayae	WP_055008878	------SA--A--N-PLNASYHVPT-	-VN---I-------M---Y--E--
	Pseudomonas borbori	WP_090501081	--R------EA----PGDASHH-P--	-VT---I---V---M---Y--E--
	Pseudomonas syringae	WP_024646249	------TA--A--N-PLNASYHVPT-	-VN---I-------M---Y--E--
	Pseudomonas cerasi	WP_065350825	------TA--A--N-PLNASYHVPT-	-VN---I-------M---Y--E--
	Pseudomonas congelans	WP_010438754	------TA--A--N-PLNASYHVPT-	-VN---I-------M---Y--E--
	Pseudomonas seleniipraecipitan	WP_092367061	--R---A--ET----P-DASYHVQT-	-VP-----------M---Y-G---
	Pseudomonas costantinii	WP_071482661	------AA-D--V--PLNASYHVPT-	-VN---I-------M---Y--E--
	Pseudomonas multiresinivorans	WP_169935939	--F-------V-IQ-P-GASYHVQTE	--A---I-------L-----G---
	Pseudomonas sihuiensis	WP_092379590	--R---AS-EE----V-DA-YHVPT-	--S---I-------M---Y--E--
	Pseudomonas alcaliphila	WP_075745084	--R---AS-EE----V-DA-YHVPT-	--S---I-------M---Y--E--
	Pseudomonas pseudoalcaligenes	WP_003459020	--R---AS-EE----V-DA-YHVPT-	--S---I-------M---Y--E--
	Pseudomonas chengduensis	WP_017678538	--R---AS-EE----V-DA-YHVPT-	--S---I-------M---Y--E--
	Pseudomonas indoloxydans	WP_108233406	--R---AS-EE----V-DA-YHVPT-	--S---I-------M---Y--E--
	Pseudomonas mendocina	WP_011920759	--R---AS-ET----E-DA-YHVPA-	--S---I-------M---Y--E--
	Pseudomonas hydrolytica	WP_129481891	--R---AS-ET----E-DA-YHVPA-	--S---I-------M---Y--E--
	Pseudomonas asuensis	WP_188864576	--Y---KA-ET-V--SIDAYHHV-V-	--A-----------M---Y-----
	Pseudomonas taeanensis	WP_025164206	---------ED----PINASYHVPT-	-IA-----------M---Y--E--
	Pseudomonas fildesensis	WP_048723238	------AA-E--VS-PLNASYHVPT-	-VN---I-------M---Y--E--
	Pseudomonas daroniae	WP_131191345	--R---A--ET----PMDASYHVPT-	-VP-----------M---Y-----
	Pseudomonas sediminis	WP_099522790	--R---AS-EE----V-DA-YHVPA-	--S---I-------M---Y--E--
	Pseudomonas composti	WP_074936587	--R---AS-EE----E-DA-YHVPT-	--S---I-------M---Y--E--
	Pseudomonas chlororaphis	WP_016704444	----SEAD--A-V--PLNASYHVPT-	-VN-----------M---Y-----
	Pseudomonas guguanensis	WP_090430272	--R---TS-EQ----E-DA-YHVPA-	--S---I-------M---Y--E--
	Pseudomonas delhiensis	WP_089389906	--F---A---E-V--P-GASYHV-T-	--A---I-------M---Y--N--
	Pseudomonas reidholzensis	WP_119146626	-----DAS--T----PLNASYHVPT-	-VN---I-------M---Y--E--
	Pseudomonas avellanae	WP_005621209	------TA--A--N-PLNASYHVPT-	-VN---I-------M---Y--E--
	Pseudomonas cichorii	WP_025258131	------NA--A--N-PLNASYHVPT-	-VY---I-------M---Y--E--
	Pseudomonas oleovorans	WP_150607759	--R---AS-E-----E-DA-YHVPA-	-IS---I-------M---Y--E--
	Pseudomonas hydrolytica	WP_041976365	--R---AS-EQ----E-DA-YHVPA-	--S---I-------M---Y--E--
	Pseudomonas toyotomiensis	WP_074913719	--R---AS-EQ----E-DA-YHVPA-	--S---I-------M---Y--E--
	Pseudomonas khazarica	WP_134676115	--R---AS-E-----E-DA-YHVPA-	-IS---I-------M---Y--E--
	Pseudomonas zeshuii	WP_010797029	--Y---RA-ET-V--PIDAYHHV-V-	--A-----------M---Y-----
	Pseudomonas tremae	WP_005888202	------SA--A--S-PLNASYHVPT-	-VN---I-------M---Y--A--
	Pseudomonas coronafaciens	WP_005888202	------SA--A--S-PLNASYHVPT-	-VN---I-------M---Y--A--
	Pseudomonas humi	WP_061562996	--F---A---E-V--P-GASYHV---	--A---I-------M---Y-GN--
	Pseudomonas gingeri	WP_017126916	-----EAA--A-IV-PINASYHVPT-	-VN---I-------M---Y--E--
	Pseudomonas veronii	WP_079442681	----S-SA-E--V--PLNASYHVPT-	-VN---I-------M---Y--E--
	Pseudomonas panacis	WP_046384970	----S-SA-E--V--PLNASYHVPT-	-VN---I-------M---Y--E--
	Pseudomonas aeruginosa	WP_003103276	--F-------A-VD-PINASYHVQT-	--N---I-------M---Y--E--
	Pseudomonas nitroreducens	WP_024764149	--F--L----A-IQ-P-GASYHVQT-	--A---I-------L-----G---
	Pseudomonas dryadis	WP_131174421	--R---E---A----PLGASYHVPT-	-VP-----------M---Y-----
	Pseudomonas denitrificans	WP_003095841	--F-------A-VE-PINASYHVQT-	--N---I-------M---Y--E--
	Pseudomonas benzenivorans	WP_090440822	--R--R---EA----P-DASYHVPK-	-VV-------V---M---Y-----
	Pseudomonas pharmafabricae	WP_101194012	------A--LA-IE-PIAASYHVPK-	--S---I-------M---Y--E--
	Pseudomonas citronellolis	WP_074985602	--F---A---E-V--P-GASYHV---	--A---I------DM---Y-GN--
	Pseudomonas typographi	WP_190423463	------AA--A-VS-PINASYHVTP-	-VN--WI-------M---Y--E--
	Pseudomonas duriflava	WP_145141262	--Y-N-AA-ET-V--PIDAYHHV-T-	--A-----------M---Y-----
	Pseudomonas cuatrocienegasensi	WP_069516282	-----KA-HDA-I--GIDA-YHVQA-	-VQ-----------M---Y--E--
	Pseudomonas oryzihabitans	WP_059313505	--Y---AA--AGI--PL-AYHHV-V-	--N---I-------M---Y-G---
	Pseudomonas agarici strain	WP_060783380	-----EAT--ASIV-PINASYHVPT-	-VN---I-------M---Y-GE--
	Pseudomonas lini	WP_038978606	----TEAD-TT-IN-LNASYHVPT E	VN-----------M---Y-----
	Pseudomonas ovata	WP_109512101	----N-AA--A-IV-PMNASYHVQ--	-VY---I-------M---Y--E--
	Pseudomonas mohnii	WP_047536461	----SEAD-TT-IN-PLNASYHVTP-	-VN---I-------M---Y-----
	Pseudomonas kilonensis	WP_053177811	----SEAD-TT-IN-PLNASYHVPT-	-VN---I-------M---Y-----
	Pseudomonas arsenicoxydans	WP_090176587	----TEAD-TT-IN-PLNASYHVPT-	-VN---I-------M---Y-----
	Pseudomonas prosekii	WP_092274489	----TEAD-TT-IN-PLNASYHVPT-	-VN---I-------M---Y-----
	Pseudomonas mandelii	WP_010464433	----TEAD-TT-IN-PLNASYHVPT-	-VN---I-------M---Y-----
	Pseudomonas umsongensis	WP_033042561	----SEAD-TT-VN-PLNASYHVPT-	-VN---I-------M---Y-----
	Pseudomonas helmanticensis	WP_134175933	----SEAD-TA-IN-PLNASYHVPT-	-VN---I-------M---Y-----
	Pseudomonas reinekei	WP_075947235	----SEAD-TA-IN-PLNASYHVPT-	-VN---I-------M---Y-----
	Pseudomonas moraviensis	WP_065615442	----SEAD-TA-IN-PLNASYHVPT-	-VN---I-------M---Y-----
	Pseudomonas moorei	WP_090328220	----SEAD-TA-IN-PLNASYHVPT-	-VN---I-------M---Y-----

Group	Species	Accession	Sequence 1	Sequence 2
Other *Pseudomonas*	*Pseudomonas laurylsulfatovoran*	WP_103397275	----SEAD-TA-IN-PLNASYHVPT-	-VN---I-------M---Y-----
	Pseudomonas vancouverensis	WP_093227480	----SEAD-TA-IN-PLNASYHVPT-	-VN---I-------M---Y-----
	Pseudomonas thivervalensis	WP_053117651	----SEAD-TA-IN-PLNASYHVPT-	-VN---I-------M---Y-----
	Pseudomonas baetica	WP_100846510	----SEAD-TA-IN-PLNASYHVPT-	-VN---I-------M---Y-----
	Pseudomonas corrugata	WP_024779910	----SEAD-TA-IN-PLNASYHVPT-	-VN---I-------M---Y-----
	Pseudomonas jinjuensis	WP_084314517	--F---A--EA-VN-PIAASYHVQT-	--I---I-------M---Y-----
	Pseudomonas brassicacearum	WP_025211412	----TEAD-TA-IN-PLNASYHVPT-	-VN---I-------M---Y-----
	Pseudomonas pohangensis	WP_090196805	--R--KE-LEQ-I--PIDASYHGPQ-	----H-I---V---M---Y-----
	Pseudomonas kribbensis	WP_085711054	----TEAD-TA-IN-PLNASYHVPT-	-VN---I-------M---Y-----
	Pseudomonas migulae	WP_084323157	----TEAD-TA-IN-PLNASYHVPT-	-VN---I-------M---Y-----
	Pseudomonas laurylsulfatiphila	WP_104451107	----TEAD-TA-IN-PLNASYHVPT-	-VN---I-------M---Y-----
	Pseudomonas mediterranea	WP_047700493	----SEAD-TA-IN-PLNASYHVPT-	-VN---I-------M---Y-----
	Pseudomonas silesiensis	WP_064675504	----TEAD-TA-IN-PLNASYHVPT-	-VN---I-------M---Y-----
	Pseudomonas granadensis	WP_090280415	----TEAD-TA-IN-PLNASYHVPT-	-VN---I-------M---Y-----
	Pseudomonas jessenii	WP_090452669	----SEAD-TA-VN-PLNASYHVPT-	-VN---I-------M---Y-----
	Pseudomonas stutzeri	WP_043942093	--SL-ESS-RQ----PETA-YHG-T-	--D-S-I-------M------A--
	Pseudomonas panipatensis	WP_090265445	--F---A-F-A-VN-P-GASYHVP--	--V---I-------L---Y-GN--
	Pseudomonas protegens	WP_011058818	----TEAA-TE-IN-PLNASYHVPT-	-VN---I-------M---Y-----
	Pseudomonas saponiphila	WP_092318886	----TEAA-TE-IN-PLNASYHVPT-	-VN---I-------M---Y-----
	Pseudomonas piscis	WP_152899053	----SEAD-TE-VN-PLNASYHVPT-	-VN---I-------M---Y-----
	Pseudomonas matsuisoli	WP_188983698	--R--EA--KA-IN-P-GASYHYQ--	--Y-----------M---Y--E--
	Pseudomonas fluvialis	WP_093984922	------AS-LA-IE-PIAASYHVPK-	--S---I-------M---Y--E--
	Pseudomonas songnenensis	WP_126189121	--SL-ESS-RQ----PETA-YHG-A-	--D-S-I-------M------A--
	Pseudomonas atacamensis	WP_136492395	----SEAD-TA-IN-PLNASYHVTP-	-VN---I-------M---Y--E--
	Pseudomonas koreensis	WP_041477567	----SEAD-TA-IN-PLNASYHVPT-	-VN---I-------M---Y--E--
	Pseudomonas rhizoryzae	WP_058773820	--Y-N-AT--ESVK-PL-AYHHV-V-	--N---I-------M---Y-----
	Pseudomonas psychrotolerans	WP_074529256	--Y-N-AT--ESVK-PL-AYHHV-V-	--N---I-------M---Y-----
	Pseudomonas pertucinogena	WP_188636766	--S---AT-EE-I-AP-SA-NHG-N-	------------L-MIE---TA--
	Pseudomonas chloritidismutans	WP_023446116	--SL-ETS-RQ----PETA-YHG-A-	--D-A-I-------M------A--
	Pseudomonas kunmingensis	WP_090522024	--SL-ETS-RQ----PETA-YHG-A-	--D-A-I-------M------A--
	Pseudomonas coleopterorum	WP_090355684	-----EAS-NA-IN-PLNASYHVPT-	-VS--WI-------M---Y-----
	Pseudomonas cremoris	WP_185709939	--S--AAD--A--N-D-DA-YH-PE-	-V----I------Q--R-L-E---
	Pseudomonas aestus	WP_031320214	--S--AA---A----PLTA-YH-PE-	-VD----------QM-E---EQ--
	Pseudomonas piscis	WP_152897583	--S--AA---A----PLTA-YH-PE-	-VD----------QM-E---EQ--
	Pseudomonas rhizosphaerae	WP_043191357	-----EAS-NA-IG-PLNASYHVPT-	-VS--WI-------M---Y--E--
	Pseudomonas salina	WP_150276144	--N----T-ED-V-KPIKATKYE-KA	------------L-M-E---D---
	Pseudomonas saudimassiliensis	WP_044498866	--S---AA-DE-I-APITA-NHG-N-	------------L-MLE---TN--
	Pseudomonas formosensis	WP_090538752	--S--RAA-DE-I-TP-TA-YHGSN-	------------L-MIE---TS--
	Pseudomonas hussainii	WP_074865200	--Y--EPSL-V--SAP-DA-YHVP--	-VS---I-------M---Y--A--
	Pseudomonas caeni	WP_028244225	--R-SDEQ--E-VS-PLTASLHINM-	D-N---I---V---M-----GQ--
	Pseudomonas litoralis	WP_090274397	--S---AAHDE-V-APITA-NHG-N-	------------L-MIE---TE--
	Pseudomonas oceani	WP_104738103	--Y--EAS-RD-V-TPITA-NHG-N-	-M----I-----L-M-E-Y-D---
Other Bacteria	*Azotobacter beijerinckii*	WP_090620478	--Y--EA---Q----PADASYHVPT-	--N---I-------L---Y-----
	Azotobacter chroococcum	WP_089167128	--H--EAS--Q----PA-ASYHV-T-	--N---I-------L---Y-----
	Azotobacter vinelandii	WP_012703012	--H--EA---Q----PADASYHVQT-	--N---I--L----L---Y-----
	Azotobacter salinestris	WP_152387374	--H--ET--LQ----PADASYHVPA-	--N---I--L----L---Y-----
	Azomonas macrocytogenes	WP_183165871	--TL-DA--RQ----P-DAHYHI-Q-	--D---I-------M---Y--A--
	Azomonas agilis	WP_144571317	--MLSDE---L----P-NA-YHV-Q-	--D---I-------M---Y-AT--

Figure-S43

Partial sequence alignments of Penicillin-binding protein 1A showing a 1aa Ins (highlighted), which are uniquely shared by all species from the Linyingensis clade.

			672		718
**Linyingensis Clade** (***Geopseudomonas* gen. nov.**) **(6/6)**	*Pseudomonas linyingensis*	WP_090307131	ILEQVLQLGWRQVAQRHGYPRRAD	G	SVASEPDFIIVGYGKVGGIELG
	Pseudomonas guangdongensis	WP_090213546	-------------T-K--F-LCE-	-	-L-R--E-V------L------
	"Pseudomonas oryzae"	WP_090349593	--------A----GLK------E-	-	-SV---E-V-------------
	Pseudomonas sagittaria	WP_092430205	--------A----G-K--------	-	-----A----------------
	Pseudomonas aromaticivorans	WP_217682016	------------------------	-	----------------------
	Pseudomonas oryzagri	WP_229606265	--------A----GL---------	-	-SV---E-V-------------
**Other *Pseudomonas***	*Pseudomonas profundi*	WP_150299567	--Q---R-A--ELT----A-K-R-		GSDC-L--V-------------
	Pseudomonas abietaniphila	WP_074751727	------A-A--HTVA---A---P-		GTVCD-G-V-------------
	Pseudomonas abyssi	WP_096003386	--Q---L-A--EMVA---R-A---		GSRC-L---V------------
	Pseudomonas aeruginosa	WP_003146359	--VE--E-A---LV----R-L---		GTPCD---V---------L-F-
	Pseudomonas aestus	WP_022641017	------A-A---TVA---A-L-T-		GTLCD-G---------------
	Pseudomonas aestusnigri	WP_088275707	--Q---M-A--EMVA---R-A---		GSRC-L----------------
	Pseudomonas agarici	WP_060783598	------A-A---TVGKY-S-----		GSLCD-G---------------
	Pseudomonas alcaligenes	WP_061903260	--D---A-A---TVAKY-N-----		GSLCD-------------L-F-
	Pseudomonas alcaliphila	WP_075745130	--D---A-A-QHTV----R-Q---		GTPCD-------------L-F-
	Pseudomonas alkylphenolica	WP_038605898	--N---A-A---TVA---Q-K-S-		GSLCD-G---I---------F-
	Pseudomonas allii	WP_179029566	------A-A---TVA---S-Q-L-		GTLCD-G---------------
	Pseudomonas anguilliseptica	WP_090380706	--D---A-A--HTVSKY-A---T-		GSICD---V-------------
	Pseudomonas antarctica	WP_064450356	------A-A---TVA---S-Q-V-		GSLCD-G---------------
	Pseudomonas argentinensis	WP_074882208	--D---A-A---MVN---T-L---		GSPCA-----------------
	Pseudomonas arsenicoxydans	WP_090176410	------A-A---TVAKY-T-L-T-		GTLCD-G-----------L---
	Pseudomonas asiatica	WP_061303841	--D---A-A---TVA---Q-K-S-		GSLCD-G---I-------L---
	Pseudomonas asplenii	WP_090202763	------A-A---TVSKY-S-Q---		GTLCD-G---------------
	Pseudomonas asturiensis	WP_073168080	------A-A--YSVA---T-S-P-		GTLCD-G-V-------------
	Pseudomonas asuensis	WP_188864557	--D---E-A---MV----R-Q---		GSPCD---V-------------
	Pseudomonas atacamensis	WP_136492369	------A-A---TVAKY-T-L-T-		GTLCD-G---------------
	Pseudomonas avellanae	WP_081591690	--D---A-A--YSVA---T-F-P-		GTLCD-G-V-------------
	Pseudomonas azotifigens	WP_028241553	------AIA--YMVA---R-Q---		GSPCD-A-V---------L---
	Pseudomonas azotoformans	WP_061435042	------A-A---TVA---S-Q-V-		GTLCD-G---------------
	Pseudomonas baetica	WP_100846458	------A-A---TVAKY-T-L-T-		GTLCD-G---------------
	Pseudomonas balearica	WP_043222255	--NE--T-A--HMVA---Q---S-		GSPCD-A-V-------------
	Pseudomonas batumici	WP_040063771	------A-A---TVA---S---V-		gSLCD-G---------------
	Pseudomonas benzenivorans	WP_090440662	--D---A-A---TVA---A---L-		GGPCA-----------------
	Pseudomonas bohemica	WP_110950982	------A-A--HTVA---A---P-		GTLCD-G-V-------------
	Pseudomonas borbori	WP_090501018	--D---A-A--HTVSKY-V-L-V-		GSPCD-----------------
	Pseudomonas brassicacearum	WP_025211490	------A-A---TVAK--V-L-T-		GSLCD-G-----------L---
	Pseudomonas brassicae	WP_163941381	--N---A-A---TVA---Q-K-S-		GSLCD-G---------------
	Pseudomonas brenneri	WP_032862118	------A-A---TVA---S-Q-L-		GTLCD-G---------------
	Pseudomonas bubulae	WP_095025241	------A-A---TVAKY-T-Q-T-		GSLCD-G---------------
	Pseudomonas caeni	WP_022965947	------H-V-NEMVRK--L-Q-L-		GSP--L--VV-----L------
	Pseudomonas canadensis	WP_028615467	------A-A---TVA---S-Q-L-		GTLCD-G---------------
	Pseudomonas capeferrum	WP_033703118	--D---A-A---TVA---Q-K-S-		GSLCD-G---I-------L---
	Pseudomonas carnis	WP_146469866	------A-A---TVA---S-Q-L-		GTLCD-G---------------
	Pseudomonas caspiana	WP_087271361	---E--A-A--HTVA---T-S-P-		GSLCD-G-V-------------
	Pseudomonas cedrina	WP_076951798	------A-A---TVA---S-Q-L-		GTLCD-G---------------
	Pseudomonas chengduensis	WP_055984344	--D---A-A-QHTV----R-Q---		GTLCD-------------L-F-
	Pseudomonas chloritidismutans	WP_023445088	--QE--T-A--HTVA---Q---S-		GTLCD-A-V-------------
	Pseudomonas chlororaphis	WP_038630433	------A-A---TVAKY-A-Q-T-		GTLCD-G-----------L---
	Pseudomonas cichorii	WP_025258248	------A-A--HTVA---T-S-P-		GSLCD-S-V-------------
	Pseudomonas citronellolis	WP_074983466	------A-A---LV----R-L---		GTPCDT------------L---
	Pseudomonas coleopterorum	WP_090362423	------A-A---TVA---V-K-P-		GSDCD-A-V-------------
	Pseudomonas composti	WP_074936486	--D---A-A-QHTV----R-Q---		GSLCD-------------L-F-
	Pseudomonas coronafaciens	WP_024668636	--D---A-A--YSVA---T-S-P-		GSLCD-G-V-------------
	Pseudomonas corrugata	WP_055136032	------A-A---T-AK--V-L-T-		GSLCD-G-----------L---
	Pseudomonas costantinii	WP_071482714	------A-A---TVA---S-Q-V-		GTLCD-G---------------
	Pseudomonas cremoricolorata	WP_038411176	--D---A-A---TVA---Q-MGN-		GALCD-G---------------
	Pseudomonas cremoris	WP_185704989	------A-A---TVA---S-Q-L-		GTLCD-G---------------
	Pseudomonas cuatrocienegasensi	WP_069521880	--D---A-A---TVA---V-Q---		GSLCD-----------------
	Pseudomonas daroniae	WP_131190772	--D---A-A---MVA---T-Q-L-		GSLCA-----------------
	Pseudomonas deceptionensis	WP_048360383	------A-A---TVAKY-T-Q--N		GSLCD-G---------------
	Pseudomonas delhiensis	WP_089389932	------A-A-H-LV----R-L---		GTPCDT------------L---
	Pseudomonas denitrificans	WP_003146359	--VE--E-A---LV----R-L---		GTPCD---V---------L-F-
	Pseudomonas donghuensis	WP_010221379	--N---A-A---TVS---Q-K-S-		GSLCD-G---------------
	Pseudomonas dryadis	WP_131173387	--D---A-A---MVA---T-Q-V-		GSLCA---V-------------
	Pseudomonas duriflava	WP_145141309	------A-A---MV----H-L-T-		GSPCD-----------------
	Pseudomonas edaphica	WP_138450287	------A-A---TVA---S-Q-V-		GSLCD-G---------------
	Pseudomonas endophytica	WP_055104870	------A-A---TVAKY-T-Q-PN		GSLCD-G---------------
	Pseudomonas entomophila	WP_011536131	--D---A-A---T-A---Q---S-		GSLCD-G-----------L---
	Pseudomonas extremaustralis	WP_010563262	------A-A---TVA---S-Q-L-		GTLCD-G---------------
	Pseudomonas extremorientalis	WP_071492321	------A-A---TVA---S-Q-L-		GTLCD-G---------------
	Pseudomonas fildesensis	WP_048727704	------A-A---TVA---S-Q-L-		GTLCD-G---------------
	Pseudomonas flavescens	WP_084308605	--D---A-A---MVT---T-Q-P-		GSLCA-----------------
	Pseudomonas flexibilis	WP_039559235	------A-A--HTVAK--T-K---		GSDCD-----------------
	Pseudomonas floridensis	WP_083184095	------A-A--YSVA---T-T---		GTLCD-G-V-------------
	Pseudomonas fluorescens	WP_053253998	------A-A---TVA---S-Q-V-		GSLCN-G---------------
	Pseudomonas fluvialis	WP_093984643	--Q-----A---LTSK--V---S-		GSLCDG----------------
	Pseudomonas formosensis	WP_090538376	--Q-----A-QDMVR---V-K-H-		GTPC-L----------------
	Pseudomonas fragi	WP_016783246	------A-A---TVAKY-T-Q-T-		GSLCD-G---------------
	Pseudomonas frederiksbergensis	WP_071552252	------T-A---TVAKY-S-Q-T-		GTLCA-G-V---------L---
	Pseudomonas fulva	WP_013793266	--D---A-A---MVS---T-L-V-		GSPCS-----------------
	Pseudomonas furukawaii	WP_003453150	--D---A-A---TVA---T-K---		GSLCD-------------L-F-
	Pseudomonas fuscovaginae	WP_019360963	------A-A---TVSKY-S-Q---		GTLCD-G---------------
	Pseudomonas gallaeciensis	WP_118129210	--Q---L-A--EMVA---R-A---		GSRC-L---V------------
	Pseudomonas gessardii	WP_076961770	------A-A---TVA---T-Q-L-		GTLCD-G---------------
	Pseudomonas gingeri	WP_017125127	------A-A---TVGKY-S-Q---		GTLCD-G---------------
	Pseudomonas graminis	WP_074892181	------A-A--HTVA---A---P-		GTLCD-G-V-------------
	Pseudomonas granadensis	WP_090280598	------A-A---TVAKY-T-L-T-		GTLCD-G-----------L---
	Pseudomonas grimontii	WP_090400917	------A-A---TVA---S-Q-L-		GTLCD-G---------------
	Pseudomonas guariconensis	WP_090346299	--D---A-A---TVA---Q-K-S-		GSLCD-G---------------
	Pseudomonas guguanensis	WP_090430332	--D---A-A-QHTV----R-Q-V-		GTPCD-------------L-F-
	Pseudomonas guineae	WP_090240291	-----MA-A--HTVSKY-A-Q-V-		GSLCD---V-------------
	Pseudomonas haemolytica	WP_153871555	------A-A---TVA---S-Q-L-		GTLCD-G---------------
	Pseudomonas helleri	WP_048366654	------A-A---TVAKY-T-Q---		GSLCD-G---------------
	Pseudomonas helmanticensis	WP_134175884	------A-A---TVAKY-T-L-T-		GTLCD-G-----------L---
	Pseudomonas humi	WP_069863136	------A-A-H-LV----R-L---		GTPCDT------------L---
	Pseudomonas hunanensis	WP_003255650	--D---A-A---TVA---Q-K-S-		GSLCD-G---I----M--L---

Other *Pseudomonas*

Pseudomonas hussainii	WP_074865031	--RE--N-A-Q-MVL---R-----	GGVCD-----------------
Pseudomonas hydrolytica	WP_041976490	--D---A-A-QHTV----R-Q-V-	GTPCD-------------L-F-
Pseudomonas hydrolytica	WP_129481869	--D---A-A-QHTV----R-F-T-	GTPCD-------------L-F-
Pseudomonas indica	WP_084332862	------A-A---SVAKY-T---P-	GSLCD-----------------
Pseudomonas indoloxydans	WP_108233385	--D---A-A-QHTV----R-F---	GTPCD-------------L-F-
Pseudomonas inefficax	WP_133970004	--D---A-A---TVA---Q-K-S-	GSLCD-G---I-------L---
Pseudomonas japonica	WP_042124061	--N---A-S---TVA---R-Q-S-	GSPCD-G-----------L---
Pseudomonas jessenii	WP_090452766	------A-A---TVAKY-T-L-T-	GTLCD-G-----------L---
Pseudomonas jilinensis	WP_119700462	--Q-----A--EMSL---V-----	GSPC-L----------------
Pseudomonas jinjuensis	WP_084313936	--SE--E-A-H-LV----R-L---	GTPCD----V--------L-F-
Pseudomonas kairouanensis	WP_135291946	------A-A---TVA---S-Q-L-	GTLCD-G---------------
Pseudomonas khazarica	WP_134676059	--D---A-A-QHTV----R-S---	GTPCA-------------L-F-
Pseudomonas kilonensis	WP_018614083	------A-A---TTAK--V-L-T-	GSLCD-G-----------L---
Pseudomonas kirkiae	WP_131184301	------A-A-HHTVA---R-K-V-	GSLCD-A---------------
Pseudomonas knackmussii	WP_043255871	------A-A-H-LV-K--R-L---	GTPCD-------------L---
Pseudomonas koreensis	WP_041477617	------A-A---TVAKY-T-L-T-	GTLCD-G---------------
Pseudomonas kribbensis	WP_114881169	------A-A---TVAKY-T-L-T-	GTLCD-G---------------
Pseudomonas kunmingensis	WP_044313855	--QE--T-A--HTVA---Q-Q-S-	GTLCD-A-V-------------
Pseudomonas kuykendallii	WP_090225752	--D---A-A--HTVA---A-K---	GSPCD---V-------------
Pseudomonas lactis	WP_057709600	------A-A---TVA---S-Q-L-	GTLCD-G---------------
Pseudomonas laurylsulfatiphila	WP_104451068	------A-A---TVAKY-T-L-T-	GTLCD-G-----------L---
Pseudomonas laurylsulfatovoran	WP_103397333	------A-A---TVAKY-T-L-T-	GTLCD-G-----------L---
Pseudomonas leptonychotis	WP_136663319	--D---A-A-HHTVS-Y-A---V-	GSLCA---V-------------
Pseudomonas libanensis	WP_057010694	------A-A---TVA---A-Q-L-	GTLCD-G---------------
Pseudomonas lini	WP_048396114	------A-A---TVAKY-T-L-T-	GSLCD-G-----------L---
Pseudomonas litoralis	WP_090273566	--Q---E-A-SDMVR---V---H-	GSLC-L---------------A
Pseudomonas lundensis	WP_047281980	------A-A---TVAKY-T-Q-T-	GSLCD-G---------------
Pseudomonas lurida	WP_069021307	------A-A---TVA---S-Q-L-	GTLCD-G---------------
Pseudomonas lutea	WP_037015901	------A-A--HTVA---A---P-	GTLCD-G-V-------------
Pseudomonas luteola	WP_074823122	--D---E-A---MV----R-Q---	GSPCD---V-I-----------
Pseudomonas marginalis	WP_064054014	------A-A---TVA---S-Q-L-	GTLCD-G---------------
Pseudomonas marincola	WP_090510256	---E--A-A--HTQ-KY-T-K-I-	GTYCD-G---------------
Pseudomonas massiliensis	WP_040259621	--A---A-A---TVA---T---P-	GSPCD-G--------M--L---
Pseudomonas matsuisoli	WP_188985895	--Q---A-A----TA-Y-A-K---	GSVC-L----------------
Pseudomonas mediterranea	WP_047700432	------A-A---T-AK--V-L-T-	GSLCD-G-----------L---
Pseudomonas meliae	WP_044343223	--D---A-A--YSVA---T-S-P-	GTLCD-G-V-------------
Pseudomonas mendocina	WP_011920793	--D---A-A-QHTV----R-F-T-	GTPCD-------------L-F-
Pseudomonas migulae	WP_084320960	------A-A---TVAKY-T-Q-T-	GTLCD-G-----------L---
Pseudomonas mohnii	WP_090465518	------A-A---TVAKY-T-L-T-	GTLCD-G-----------L---
Pseudomonas monteilii	WP_024088011	--D---A-A---TVT---Q-K-S-	GSLCD-G---I-------L---
Pseudomonas moorei	WP_090328374	------A-A---TVAKY-T-L-S-	GTLCD-G-----------L---
Pseudomonas moraviensis	WP_083354054	------A-A---TVAKY-T-L-T-	GTLCD-G---------------
Pseudomonas mosselii	WP_096049185	--D---A-A---TVA---Q-K-S-	GSLCD-G-----------L---
Pseudomonas mucidolens	WP_084379700	------A-A---TVA---A-L-M-	GTLCD-G---------------
Pseudomonas multiresinivorans	WP_169935988	------V-T-D-LV----R-T-V-	GTPCD-----I----S--L---
Pseudomonas nabeulensis	WP_135308016	------A-A---TVA---S-Q-V-	GTLCD-G---------------
Pseudomonas nitritireducens	WP_170858850	------S-T-D-LV----R-T-V-	GTPCD-----I----S--L---
Pseudomonas nitrititolerans	WP_170910558	--KE--A-A--HTVA---Q-K-S-	GSPCD-A---L-----------
Pseudomonas nitroreducens	WP_084358652	------S-T-D-LV----R-T-V-	GTPCD-----I----S--L---
Pseudomonas oceani	WP_104737350	--Q---V-A--EMVA---R-A---	GSRCDL----------------
Pseudomonas oleovorans	WP_150607825	--D---A-A-QHTV----R-S---	GTPCD-------------L-F-
Pseudomonas orientalis	WP_057724372	------A-A---TVA---S-Q-L-	GSLCN-G---------------
Pseudomonas oryzihabitans	WP_059313712	---E-FG-A---LV----T-L-R-	GSPCQ-----------------
Pseudomonas otitidis	WP_074970667	--D---A-A--HTVA---T-----	GS-CD-------------L-F-
Pseudomonas ovata	WP_109513503	--Q---A-A--HTVA---T-L-P-	GTPCD-A-V-------------
Pseudomonas pachastrellae	WP_083725406	--Q---L-A--EMVA---R-A---	GSRC-L---V------------
Pseudomonas palleroniana	WP_090367214	------A-A---TVA---S-Q-V-	GTLCD-G---------------
Pseudomonas panacis	WP_057003653	------A-A---TVA---A-Q-L-	GSVCD-G---------------
Pseudomonas panipatensis	WP_090262920	------A-A-N-LV----R-L---	GTPCD-------------L---
Pseudomonas parafulva	WP_028635265	--D---A-A---TVA---Q-V-S-	GSPCD-G---I-------L---
Pseudomonas paralactis	WP_057702538	------A-A---TVA---S-Q-L-	GTLCD-G---------------
Pseudomonas pelagia	WP_022961423	--Q---L-A--EMT----F-K---	GSLC-M--V-L-----------
Pseudomonas peli	WP_090249837	--D---A-A--HTVSKY-A-----	GSICD---V-------------
Pseudomonas pertucinogena	WP_188634919	--Q-----A--DMVR---L---H-	GSPC-L--T-------------
Pseudomonas pharmafabricae	WP_101193832	--Q-----A---LTSK--V---S-	GSLCDG----------------
Pseudomonas piscis	WP_022641017	------A-A---TVA---A-L-T-	GTLCD-G---------------
Pseudomonas plecoglossicida	WP_041506298	--D---A-A---TVT---Q-K-S-	GSLCD-G---I-------L---
Pseudomonas poae	WP_060548464	------A-A---TVA---S-Q-L-	GSLCN-G---------------
Pseudomonas pohangensis	WP_090196727	------A-A---MVA---T---V-	GSLCN----V------------
Pseudomonas prosekii	WP_092274307	------A-A---TVAKY-T-L-N-	GTLCD-G-----------L---
Pseudomonas protegens	WP_019093029	------A-A---TVSK--A-L-T-	GTLCD-G---------------
Pseudomonas proteolytica	WP_092232978	------A-A---TVA---T-Q-L-	GTLCD-G---------------
Pseudomonas pseudoalcaligenes	WP_003459084	--D---A-A-QHTV----R-F---	GTPCD-------------L-F-
Pseudomonas psychrophila	WP_019825358	------A-A---TVAKY-T-Q-T-	GSLCD-G---------------
Pseudomonas psychrotolerans	WP_074529374	--AE-FG-A---LV----T-Q-R-	GSPCQ-----------------
Pseudomonas pudica	WP_108481139	--D---A-A---TVA---Q-K-T-	GSLCD-G---I-------L---
Pseudomonas punonensis	WP_073266506	--D---A-A---MVS---T-Q-S-	GSPCA-----------------
Pseudomonas putida	WP_016497646	--D---A-A---TVA---Q---S-	GSLCD-G---I-------L---
Pseudomonas qingdaonensis	WP_100634797	--N---A-A---TVA---Q-K-S-	GSLCD-G---------------
Pseudomonas reactans	WP_177001628	------A-A---TVA---S-Q-L-	GTLCD-G---------------
Pseudomonas reidholzensis	WP_119137613	--D---A-A---TVA---Q---S-	GSPCD-----------------
Pseudomonas reinekei	WP_075947290	------A-A---TVAKY-T-L-T-	GSLCD-G-----------L---
Pseudomonas resinovorans	WP_016495097	--D---A-T---TVAKY-T-K---	GSLCD---V---------L-F-
Pseudomonas rhizoryzae	WP_058773358	--AE-FG-A---LV----T-Q-R-	GSPCQ-----------------
Pseudomonas rhizosphaerae	WP_084139897	------A-A---TVA---V-K-P-	RSDCD-A-V-------------
Pseudomonas rhodesiae	WP_034136614	------A-A---TVA---S-Q-L-	GTLCD-G---------------
Pseudomonas sabulinigri	WP_092285398	--Q---K-A--EMVS---Q-A---	GSRC-L---V------------
Pseudomonas salegens	WP_092384161	--Q-----A--EMV----R-Q-V-	GSRC-L----------------
Pseudomonas salina	WP_150278250	--Q-----A--EMTL---K-K-E-	GSDC-M--V-L-----------
Pseudomonas salomonii	WP_069786817	------A-A---TVA---S-Q-V-	GSLCD-G---------------
Pseudomonas saponiphila	WP_092318991	------A-A---TVAKY-A-Q-S-	GTLCD-G---------------
Pseudomonas saudimassiliensis	WP_044500043	--Q---E-A--DMVR---T---H-	GSPC-L---V-----------S
Pseudomonas savastanoi	WP_011167523	--D---A-A--YSVA---T-L-P-	GTLCD-G-V-------------
Pseudomonas saxonica	WP_122785135	------A-A---TVAKY-T-L-S-	GSLCD-G---------------
Pseudomonas sediminis	WP_099522763	--D---A-A-QHTV----R-Y---	GTPCD-------------L-F-
Pseudomonas segetis	WP_089359835	--D---A-S--HTCAKY-T-K-V-	GSDCD-G-------------M-
Pseudomonas seleniipraecipitan	WP_092368669	--D---A-A---MVT---T-Q-P-	GSLCA-----------------

Other *Pseudomonas*	*Pseudomonas shirazica*	WP_061303841	--D---A-A---TVA---Q-K-S-	GSLCD-G---I-------L---
	Pseudomonas sichuanensis	WP_110992294	--D---A-A---TVS---Q-K-S-	GSLCD-G---------------
	Pseudomonas sihuiensis	WP_092379695	--D---A-A-QHTV----R-F---	GTPCD------------L-F-
	Pseudomonas silesiensis	WP_064675558	------A-A---TVAKY-T-L-T-	GTLCD-G-V---------L---
	Pseudomonas simiae	WP_069672812	------A-A---TVA---S-Q-L-	GTLCD-G---------------
	Pseudomonas sivasensis	WP_181640236	------A-A---TVA---S-Q-L-	GTLCD-G---------------
	Pseudomonas soli	WP_094011547	--D---A-A---TVA---Q-K-S-	GSLCD-G-----------L---
	Pseudomonas songnenensis	WP_126188545	--QE--T-A--HTVA---Q-Q-S-	GTLCD-A-V-------------
	Pseudomonas straminea	WP_093506292	--D---A-A---MVS---T-L---	GSPCS-----------------
	Pseudomonas stutzeri	WP_014597898	--QE--T-A--HTVA---Q-Q-S-	GTLCD-A-V-------------
	Pseudomonas synxantha	WP_057023464	------A-A---TVA---A-Q-L-	GTLCD-G---------------
	Pseudomonas taeanensis	WP_025164255	--D---A-A---TVAK--T-----	GSPCD-----------------
	Pseudomonas taetrolens	WP_048382826	------A-S---TVAKY-T-Q-S-	GSLCD-G---------------
	Pseudomonas taiwanensis	WP_023382741	--D---A-A---TVS---Q-K-S-	GSLCD-G-V-I-------L---
	Pseudomonas thermotolerans	WP_017939365	--D---A-A---MVS-Y-T---S-	GSLCD-----------------
	Pseudomonas thivervalensis	WP_053117795	------A-A---T-AK--V-L-T-	GSLCD-G-----------L---
	Pseudomonas tolaasii	WP_016972664	------A-A---TVA---S-Q-V-	GTLCD-G---------------
	Pseudomonas toyotomiensis	WP_074913765	--D---A-A-QHTV----R-Q---	GTLCD------------L-F-
	Pseudomonas tremae	WP_054997483	--D---A-A--YSVA---T-S-P-	GSLCD-G-V-------------
	Pseudomonas trivialis	WP_049710356	------A-A---TVA---S-Q-L-	GTLCD-G---------------
	Pseudomonas tuomuerensis	WP_039606612	------A-A--HTVAK--T-K---	GSDCD-----------------
	Pseudomonas typographi	WP_190416723	------A-A---TTA---T-L---	GSLCA-A-V---------V---
	Pseudomonas umsongensis	WP_020796213	------A-A---TVAKY-T-L-T-	GTLCD-G-----------L---
	Pseudomonas vancouverensis	WP_093227318	------A-A---TVAKY-T-L-T-	GTLCD-G-----------L---
	Pseudomonas veronii	WP_079442703	------A-A---TVA---A-Q-L-	GSVCD-G---------------
	Pseudomonas versuta	WP_060695869	------A-A---TVAKY-T-Q---	GSLCD-G---------------
	Pseudomonas viridiflava	WP_088236064	------A-A--HSVA---T-S-P-	GTLCD-G-V-------------
	Pseudomonas vranovensis	WP_028942211	--N---A-A---TVA---Q-K-S-	GSLCD-G---------------
	Pseudomonas wadenswilerensis	WP_115084959	--N---A-A---TVS---Q-K-S-	GSLCD-G---------------
	Pseudomonas weihenstephanensis	WP_048364502	------A-A---TVAKY-T-Q-T-	GSLCD-G---------------
	Pseudomonas xanthomarina	WP_073299051	---E--A-A--HTVA---Q-Q---	GSLCD-A-V-------------
	Pseudomonas xinjiangensis	WP_093391514	--Q-----A--EMT----F----N	GSICDL--V-------------
	Pseudomonas yamanorum	WP_093202462	------A-A---TVA---S-Q-V-	GTLCD-G---------------
	Pseudomonas yangmingensis	WP_093477111	--Q-----A--ELV----R---Q-	GTPC-L--V-L-----------
	Pseudomonas zeshuii	WP_010797050	--D---E-A---MV----R-Q---	GSPCD---V-------------
	Pseudomonas zhaodongensis	WP_128121671	---E--A-A--HTVA---Q-----	GSLCD-A---L-----------
Other Bacteria	*Azomonas agilis*	WP_144571373	------A-A-KHTTA---Q-K-R-	GTLCN----------L------
	Azotobacter salinestris	WP_152387346	------N-A---MVA---N---G-	GSPCDL----------------
	Perlucidibaca piscinae	WP_051144595	V-QE--N-S-H-LTT---R---E-	GTPCD----------L--L---
	Azomonas macrocytogenes	WP_183165719	------A-A---LVA-Y-Q--CS-	GSPCD-G-LVL-----------
	Azotobacter beijerinckii	WP_090620425	--D---T-A---MVV---N---G-	GSPCDL----------------
	Azotobacter chroococcum	WP_089167153	--D---T-A---MVA---N---G-	GSPCDL----------------
	Azotobacter vinelandii	WP_012702973	--D-A-R-A---MVV---N---S-	GSPC-L--L-------------
	Thiopseudomonas denitrificans	WP_101497837	V-----R-V-HEMTHK--T---L-	GSTG-L--V------L------

Figure-S44

Partial sequence alignments of a protein bifunctional [glutamate--ammonia ligase]-adenylyl-L-tyrosine phosphorylase/[glutamate--ammonia-ligase] adenylyltransferase showing a 1aa Ins (highlighted), which is uniquely shared by all species from the Linyingensis clade.

			166		201
**Linyingensis Clade** (*Geopseudomonas* gen. nov.) (6/6)	*Pseudomonas linyingensis*	WP_090307764	GKRVQLLAENPEFAPIVIDL	DK	PDQEFVIEGLSVGV
	Pseudomonas guangdongensis	WP_090214146	-----------D-----V--	-S	-E-D----------
	"Pseudomonas oryzae"	WP_090348737	--Q-----------------	EH	--------------
	Pseudomonas sagittaria	WP_092431202	--------------------	--	--------------
	Pseudomonas aromaticivorans	WP_217679256	--------------------	EQ	--------------
	Pseudomonas oryzagri	WP_229607073	--Q-----------------	ER	--------------
**Other** ***Pseudomonas***	*Pseudomonas profundi*	WP_150298971	-RK-S-I----D----D---		AE--LI--------
	Pseudomonas abietaniphila	WP_062382291	-SK-W-I----D----EVN-		K--DL---------
	Pseudomonas aeruginosa	WP_003091196	-SK-W-----------EV--		KE--LI--------
	Pseudomonas aestus	WP_031321324	-SK-W-I---------EVN-		K---L---------
	Pseudomonas aestusnigri	WP_088276506	-RK-S-M----D-R--E---		AE--LT--------
	Pseudomonas agarici	WP_026013432	-SK-W-----------EVN-		K--D----------
	Pseudomonas alcaliphila	WP_021488554	-NK-W-I---------EV--		EH-DL---------
	Pseudomonas alkylphenolica	WP_038608892	-SK-W-I---------EVN-		K---L---------
	Pseudomonas allii	WP_003172575	-SK-W-----------EVN-		K--DL---------
	Pseudomonas amygdali	WP_044317788	-SK-W------D----EV--		K---L---------
	Pseudomonas antarctica	WP_017137423	-SK-W-M---------EVN-		K--DL---------
	Pseudomonas argentinensis	WP_074880314	-N--W-----------EVN-		EE-DL---------
	Pseudomonas arsenicoxydans	WP_090182100	-SK-W-----------EVN-		K---L---------
	Pseudomonas asiatica	WP_004376420	-SK-W-----------EV--		KE--L---------
	Pseudomonas asplenii	WP_010449606	-SK-W-----------EVN-		KE-D----------
	Pseudomonas asturiensis	WP_024689271	-SK-W-----------EV--		K--DL---------
	Pseudomonas atacamensis	WP_016775490	-SK-W-I---------EV--		K---L---------
	Pseudomonas avellanae	WP_005619251	-SK-W------D----EV--		K---L---------
	Pseudomonas azotifigens	WP_028239581	-SK-W-----------EV--		EH--L---------
	Pseudomonas azotoformans	WP_003172575	-SK-W-----------EVN-		K--DL---------
	Pseudomonas baetica	WP_016775490	-SK-W-I---------EV--		K---L---------
	Pseudomonas batumici	WP_040063186	-SK-W-----------EVN-		K--D----------
	Pseudomonas bauzanensis	WP_036989643	-RK-S-I----D----EV--		RE--LI--------
	Pseudomonas benzenivorans	WP_090442280	-N--W-I---------EVN-		E--DL---------
	Pseudomonas bohemica	WP_110947538	-SK-W-----------EVN-		K--DL---------
	Pseudomonas borbori	WP_090497376	-NK-W-I---------EVN-		EE--LI--------
	Pseudomonas brassicacearum	WP_003199651	-SK-W-I---------EVN-		K--DL---------
	Pseudomonas brenneri	WP_029300096	-SK-W--------S--EVN-		K--DL---------
	Pseudomonas canadensis	WP_028619420	-SK-W-----------EVN-		K--DL---------
	Pseudomonas cannabina	WP_007251282	-SK-W------D----EV--		K---L---------
	Pseudomonas capeferrum	WP_033700412	-TK-W-----------EV--		KE--L---------
	Pseudomonas caricapapayae	WP_003315796	-SK-W------D----EV--		K---L---------
	Pseudomonas carnis	WP_003172575	-SK-W-----------EVN-		K--DL---------
	Pseudomonas caspiana	WP_087265098	-SK-W-I----D----EVN-		K---L---------
	Pseudomonas cedrina	WP_003172575	-SK-W-----------EVN-		K--DL---------
	Pseudomonas cerasi	WP_003315796	-SK-W------D----EV--		K---L---------
	Pseudomonas chengduensis	WP_021488554	-NK-W-I---------EV--		EH-DL---------
	Pseudomonas chloritidismutans	WP_014820593	--K-W-----------EV--		EQ--L---------
	Pseudomonas chlororaphis	WP_007921301	-SK-W-I---------EVN-		K--DL---------
	Pseudomonas cichorii	WP_025260736	-SK-W-I---------EV--		K---L---------
	Pseudomonas citronellolis	WP_074979919	-SK-W-I---------EV--		KE--LI--------
	Pseudomonas composti	WP_037000359	-NK-W-I---------EV--		EH-DL---------
	Pseudomonas congelans	WP_003315796	-SK-W------D----EV--		K---L---------
	Pseudomonas coronafaciens	WP_003315796	-SK-W------D----EV--		K---L---------
	Pseudomonas corrugata	WP_024777918	-SK-W-I---------EV--		QE-DL---------
	Pseudomonas costantinii	WP_003172575	-SK-W-----------EVN-		K--DL---------
	Pseudomonas cremoricolorata	WP_038414192	-SK-W-I---------EV--		K---L---------
	Pseudomonas cremoris	WP_003172575	-SK-W-----------EVN-		K--DL---------
	Pseudomonas cuatrocienegasensi	WP_069518967	-NT-W-----E------V--		EQ--LI--------
	Pseudomonas deceptionensis	WP_048358706	-NK-W-I----D----EVN-		K---L---------
	Pseudomonas delhiensis	WP_089392630	-SK-W-I---------EV--		EE--LI--------
	Pseudomonas denitrificans	WP_003091196	-SK-W-----------EV--		KE--LI--------
	Pseudomonas donghuensis	WP_010226967	-SK-W-I---------EVN-		K---L---------
	Pseudomonas edaphica	WP_017137423	-SK-W-M---------EVN-		K--DL---------
	Pseudomonas entomophila	WP_011534818	-SK-W-I---------EV--		KE--L---------
	Pseudomonas extremaustralis	WP_003172575	-SK-W-----------EVN-		K--DL---------
	Pseudomonas extremorientalis	WP_003172575	-SK-W-----------EVN-		K--DL---------
	Pseudomonas ficuserectae	WP_002554100	-SK-W------D----EV--		K---L---------
	Pseudomonas fildesensis	WP_003172575	-SK-W-----------EVN-		K--DL---------
	Pseudomonas flavescens	WP_084306533	-NT-W-----------EVN-		EE--LI--------
	Pseudomonas flexibilis	WP_039562054	--H-W-----------KV--		EH-QLT--------
	Pseudomonas floridensis	WP_004883801	-SK-W-----------EV--		K--DL---------
	Pseudomonas fluorescens	WP_017137423	-SK-W-M---------EVN-		K--DL---------
	Pseudomonas formosensis	WP_090538688	-RK-S-I---------E---		GE--LI--------
	Pseudomonas frederiksbergensis	WP_019692743	-SK-W-I---------EVN-		K--DL---------
	Pseudomonas fulva	WP_013792002	-NT-W-----------KVN-		EE-DL---------
	Pseudomonas fuscovaginae	WP_010449606	-SK-W-----------EVN-		KE-D----------
	Pseudomonas gessardii	WP_029300096	-SK-W--------S--EVN-		K--DL---------
	Pseudomonas gingeri	WP_042937141	-SK-W-----------EVN-		K--D----------
	Pseudomonas granadensis	WP_090284744	-SK-W-I---------EV--		K---L---------
	Pseudomonas grimontii	WP_003172575	-SK-W-----------EVN-		K--DL---------
	Pseudomonas guariconensis	WP_043215752	-SK-W-I---------EV--		KE--L---------
	Pseudomonas guguanensis	WP_013714775	-NK-W-I---------EV--		EH-DL---------
	Pseudomonas guineae	WP_090238241	-NK-W-I---------EVN-		EE-DLI--------
	Pseudomonas haemolytica	WP_003172575	-SK-W-----------EVN-		K--DL---------
	Pseudomonas helmanticensis	WP_003226590	-SK-W-I---------EV--		K---L---------
	Pseudomonas humi	WP_043267317	-SK-W-I---------EV--		EE--LT--------
	Pseudomonas hunanensis	WP_004577098	-SK-W-----------EV--		KE--L---------
	Pseudomonas hydrolytica	WP_003245255	-NK-W-I---------EV--		EQ-DL---------
	Pseudomonas hydrolytica	WP_013714775	-NK-W-I---------EV--		EH-DL---------
	Pseudomonas indica	WP_084334040	-NK-W-I---------EV--		EQ-NLT--------
	Pseudomonas indoloxydans	WP_021488554	-NK-W-I---------EV--		EH-DL---------
	Pseudomonas inefficax	WP_004376420	-SK-W-----------EV--		KE--L---------
	Pseudomonas japonica	WP_042124345	-SK-W-I---------EVN-		KE-DL---------
	Pseudomonas jessenii	WP_007995278	-SK-W-I---------EVN-		Q---L---------
	Pseudomonas jilinensis	WP_080050464	-RK-S-I---------EV--		GE--LT--------
	Pseudomonas jinjuensis	WP_084310012	-NK-W-I---------EV--		KE--LT--------
	Pseudomonas juntendi	WP_029887015	-SK-W-----------EV--		KE--L---------
	Pseudomonas kairouanensis	WP_003172575	-SK-W-----------EVN-		K--DL---------
	Pseudomonas khazarica	WP_037048649	-NK-W-I---------EV--		EH-DL---------

Other *Pseudomonas*

Pseudomonas kilonensis	WP_003199651	-SK-W-I---------EVN-	K--DL---------
Pseudomonas kirkiae	WP_131184824	-SK-W-I---------EV--	QR--LT--------
Pseudomonas kitaguniensis	WP_003172575	-SK-W-----------EVN-	K--DL---------
Pseudomonas knackmussii	WP_043253482	-SK-W-I---------EV--	QE--LI--------
Pseudomonas koreensis	WP_041477344	-SK-W-I---------EV--	K---L---------
Pseudomonas kribbensis	WP_085713140	-SK-W-I---------EV--	K---L---------
Pseudomonas kunmingensis	WP_014820593	--K-W-----------EV--	EQ--L---------
Pseudomonas kuykendallii	WP_090231288	-NK-W-I----D----EV--	EQ-SL---------
Pseudomonas lactis	WP_003172575	-SK-W-----------EVN-	K--DL---------
Pseudomonas laurylsulfatiphila	WP_007974043	-SK-W-I---------EVN-	Q---L---------
Pseudomonas laurylsulfatovoran	WP_103395017	-SK-W-I------D--EVN-	Q---L---------
Pseudomonas libanensis	WP_003172575	-SK-W-----------EVN-	K--DL---------
Pseudomonas lini	WP_007907770	-SK-W-I---------EVN-	K---L---------
Pseudomonas litoralis	WP_090273035	-RK-S-I----D----EV--	RE--LI--------
Pseudomonas lurida	WP_003172575	-SK-W-----------EVN-	K--DL---------
Pseudomonas luteola	WP_010797701	-NK-W-I----D-Q--EV--	NK-DL---------
Pseudomonas mandelii	WP_007944149	-SK-W-I---------EVN-	K---L---------
Pseudomonas marginalis	WP_003172575	-SK-W-----------EVN-	K--DL---------
Pseudomonas marincola	WP_069898845	-NT-W-I------Q--EV--	EQ--L---------
Pseudomonas massiliensis	WP_040260714	-HI--------D----EV--	RE-DL---------
Pseudomonas mediterranea	WP_024777918	-SK-W-I---------EV--	QE-DL---------
Pseudomonas meliae	WP_044343382	-SK-W------D----EV--	K---L---------
Pseudomonas mendocina	WP_003245255	-NK-W-I---------EV--	EQ-DL---------
Pseudomonas migulae	WP_007944149	-SK-W-I---------EVN-	K---L---------
Pseudomonas mohnii	WP_047531364	-SK-W-I---------EVN-	K---L---------
Pseudomonas monteilii	WP_004376420	-SK-W-----------EV--	KE--L---------
Pseudomonas moorei	WP_090318547	-SK-W-I----D----EVN-	K---L---------
Pseudomonas moraviensis	WP_016775490	-SK-W-I---------EV--	K---L---------
Pseudomonas mosselii	WP_028692736	-SK-W-----------EV--	KE--L---------
Pseudomonas mucidolens	WP_003172575	-SK-W-----------EVN-	K--DL---------
Pseudomonas multiresinivorans	WP_026079098	-SK-W-----------EV--	KE--L---------
Pseudomonas nabeulensis	WP_003172575	-SK-W-----------EVN-	K--DL---------
Pseudomonas nitritireducens	WP_026079098	-SK-W-----------EV--	KE--L---------
Pseudomonas nitrititolerans	WP_170910016	--K-W-----------EV--	EH--L---------
Pseudomonas nitroreducens	WP_024766859	-SK-W-----------EV--	KE--L---------
Pseudomonas nosocomialis	WP_138407220	--K-W-----------EV--	EQ--L---------
Pseudomonas oleovorans	WP_037048649	-NK-W-I---------EV--	EH-DL---------
Pseudomonas orientalis	WP_003172575	-SK-W-----------EVN-	K--DL---------
Pseudomonas oryzihabitans	WP_059315859	-NQ-T-----------EV--	SS---A--------
Pseudomonas otitidis	WP_044405045	-DK-W-I------S--EV--	HE--L---------
Pseudomonas ovata	WP_056838972	-NK-W-I----D----E---	KE--L---------
Pseudomonas palleroniana	WP_090366159	-SK-W-I---------EVN-	K--DL---------
Pseudomonas panacis	WP_003172575	-SK-W-----------EVN-	K--DL---------
Pseudomonas panipatensis	WP_090260610	-SK-W-I---------EV--	EE--LI--------
Pseudomonas parafulva	WP_028632476	-NK-W-----------EVN-	KE--L---------
Pseudomonas paralactis	WP_003172575	-SK-W-----------EVN-	K--DL---------
Pseudomonas pelagia	WP_028614745	-RK-S-F----D----EV--	GE--LT--------
Pseudomonas peli	WP_090248056	-NK-W-I---------EV--	EE-DLI--------
Pseudomonas pertucinogena	WP_188635396	-RK-S-I---------E---	AE--LI-------I
Pseudomonas piscis	WP_031321324	-SK-W-I---------EVN-	K---L---------
Pseudomonas plecoglossicida	WP_004376420	-SK-W-----------EV--	KE--L---------
Pseudomonas poae	WP_003172575	-SK-W-----------EVN-	K--DL---------
Pseudomonas pohangensis	WP_090195152	-NK-W-I---------EVN-	Q---L---------
Pseudomonas prosekii	WP_092270751	-SK-W-I---------EVN-	K---L---------
Pseudomonas protegens	WP_011060269	-SK-W-I---------EVN-	K--DL---------
Pseudomonas proteolytica	WP_029300096	-SK-W--------S--EVN-	K--DL---------
Pseudomonas pseudoalcaligenes	WP_039964828	-NK-W-I---------EV--	EH-DLI--------
Pseudomonas psychrophila	WP_019827486	-NK-W-I---------EVN-	K---L---------
Pseudomonas psychrotolerans	WP_058759960	-NQ-T-----------EV--	SS---A--------
Pseudomonas psychrotolerans	WP_058759960	-NQ-T-----------EV--	SS---A--------
Pseudomonas pudica	WP_004376420	-SK-W-----------EV--	KE--L---------
Pseudomonas putida	WP_016500740	-SK-W-----------EV--	K---L---------
Pseudomonas reactans	WP_003172575	-SK-W-----------EVN-	K--DL---------
Pseudomonas reidholzensis	WP_119139938	-SK-W-----------EV--	KQ-DL---------
Pseudomonas reinekei	WP_056857020	-SK-W-I---------EVN-	K---L---------
Pseudomonas rhizoryzae	WP_058759960	-NQ-T-----------EV--	SS---A--------
Pseudomonas rhizosphaerae	WP_043188277	-NK-F-V----D----EV--	K--DL---------
Pseudomonas rhodesiae	WP_003172575	-SK-W-----------EVN-	K--DL---------
Pseudomonas salegens	WP_092384924	-RK-S-I------S--EV--	GEE-LA--------
Pseudomonas salomonii	WP_017137423	-SK-W-M---------EVN-	K--DL---------
Pseudomonas saponiphila	WP_092316567	-SK-W-I----D----EVN-	K---L---------
Pseudomonas saudimassiliensis	WP_044499830	-RK-S-I---------EV--	RE--LI--------
Pseudomonas saudiphocaensis	WP_037021848	--K-W-----------EV--	EQ--L---------
Pseudomonas savastanoi	WP_002554100	-SK-W------D----EV--	K---L---------
Pseudomonas sediminis	WP_099526447	-NK-W-I---------EV--	EN-DL---------
Pseudomonas segetis	WP_089358864	-SK-W-I---------EVN-	EE--L---------
Pseudomonas shirazica	WP_004376420	-SK-W-----------EV--	KE--L---------
Pseudomonas sichuanensis	WP_110993543	-SK-W-I---------EV--	KE--L---------
Pseudomonas sihuiensis	WP_021488554	-NK-W-I---------EV--	EH-DL---------
Pseudomonas silesiensis	WP_064676764	-NK-W-----------EVN-	K---L---------
Pseudomonas simiae	WP_003172575	-SK-W-----------EVN-	K--DL---------
Pseudomonas sivasensis	WP_003172575	-SK-W-----------EVN-	K--DL---------
Pseudomonas soli	WP_023632613	-SK-W-I---------EV--	KE--L---------
Pseudomonas songnenensis	WP_106155227	--K-W-----------EV--	EQ--L---------
Pseudomonas stutzeri	WP_014596417	--K-W-----------EV--	EH--L---------
Pseudomonas synxantha	WP_003172575	-SK-W-----------EVN-	K--DL---------
Pseudomonas syringae	WP_024644070	-SK-W------D----EV--	K---L---------
Pseudomonas taeanensis	WP_025167380	-TK-W-I---------EV--	EK-DL---------
Pseudomonas taetrolens	WP_048382332	-SK-W-I----D----EVN-	K---L---------
Pseudomonas taiwanensis	WP_023379578	-SK-W-I---------EV--	KE--L---------
Pseudomonas thivervalensis	WP_003199651	-SK-W-I---------EVN-	K--DL---------
Pseudomonas tolaasii	WP_003172575	-SK-W-----------EVN-	K--DL---------
Pseudomonas toyotomiensis	WP_021488554	-NK-W-I---------EV--	EH-DL---------
Pseudomonas tremae	WP_003315796	-SK-W------D----EV--	K---L---------
Pseudomonas trivialis	WP_003172575	-SK-W-----------EVN-	K--DL---------
Pseudomonas tuomuerensis	WP_027589311	--H-W-----------KV--	EH-QLT--------
Pseudomonas typographi	WP_190425327	-SK-R-----------EV--	RE--L---------

Group	Species	Accession		
Other *Pseudomonas*	*Pseudomonas umsongensis*	WP_008064450	-SK-W-----------EVN-	K---L---------
	Pseudomonas vancouverensis	WP_093217749	-SK-W-I---------EVN-	Q---L---------
	Pseudomonas veronii	WP_003172575	-SK-W-----------EVN-	K--DL---------
	Pseudomonas versuta	WP_003446624	-NK-W-I----D----EVN-	K---L---------
	Pseudomonas viridiflava	WP_004883801	-SK-W-----------EV--	K--DL---------
	Pseudomonas vranovensis	WP_028945927	-NK-W-I---------EVN-	K---L---------
	Pseudomonas wadenswilerensis	WP_115086083	-SK-W-I---------EVN-	K---L---------
	Pseudomonas xanthomarina	WP_073301604	-S--W-----------E---	EQ--L---------
	Pseudomonas xinjiangensis	WP_093392568	-RK-S-I----D----E---	SE--LI--------
	Pseudomonas yamanorum	WP_003172575	-SK-W-----------EVN-	K--DL---------
	Pseudomonas yangmingensis	WP_093473769	-RK-S-I----D----EV--	GE--LT--------
	Pseudomonas zeshuii	WP_010797701	-NK-W-I----D-Q--EV--	NK-DL---------
	Pseudomonas zhaodongensis	WP_045428414	-SK-W-----A-----EV--	EQ--L---------
Other Bacteria	*Azomonas macrocytogenes*	WP_183165106	-DK-M--------D---V--	KT-DL---------
	Azotobacter beijerinckii	WP_090621954	-DK-W-I--------LE---	NE-DLT--------
	Azotobacter chroococcum	WP_089167512	-DK-W-I--------LE---	NE-DLI--------
	Azotobacter salinestris	WP_152386893	-DK-W-I--------LE---	NE-DLT--------
	Klebsiella cf. planticola	WP_042712642	-NT-E-----SD-S---V--	RK-N-T----A---
	Kosakonia cowanii	WP_023478391	-NT-E-----SD-S---V--	RK-N-T----A---
	Moellerella wisconsensis	WP_047257458	-NK-E-I----D-E---V--	RE-S-T----A---
	Photorhabdus asymbiotica	WP_015836008	-NK-E--------K------	REKNLT----A---
	Photorhabdus heterorhabditis	WP_054478017	-NK-E-----S--K---V--	R--NLT----A---
	Photorhabdus luminescens	WP_049582985	-N--E--------K---V--	R--SLT----A---
	Proteus hauseri	WP_064721284	-NKIE-H------S--IV--	RE-S-TV---A---
	Proteus penneri	WP_006536371	-NKIE-H------S--IV--	RE-S-TV---A---
	Proteus vulgaris	WP_036934582	-NKIE-H------S--IV--	RE-N-TV---A---
	Providencia alcalifaciens	WP_006658577	-N--E-I----------V--	RE-N-T----A---
	Providencia burhodogranariea	WP_008913740	-N--E-I---S-----I---	RQ-N-T----A---
	Providencia heimbachae	WP_068437338	-N-IE-I---------IV--	RQ-N-T----A---
	Providencia rettgeri	WP_042848518	-N--E-I---------IV--	RE-S-T----A---
	Providencia rustigianii	WP_006816063	-N--E-I----------V--	RQ-N-T----A---
	Providencia sneebia	WP_008916952	-N--E-I---------I---	RQ-N-T----A---
	Providencia stuartii	WP_004925920	-N--E-I---------IV--	RQ-N-T----A---
	Xenorhabdus bovienii	WP_012990285	-NKIE-I------E---V--	CE-S-T----A--I
	Xenorhabdus cabanillasii	WP_038260255	-N-IE-I------E---V--	RK-N-T----A---
	Xenorhabdus doucetiae	WP_045972439	-NTIE-I------K---V--	SE-N-T----A---
	Xenorhabdus eapokensis	WP_074024836	-NKIE-I------E---V--	SE-N-T----A---
	Xenorhabdus hominickii	WP_069318005	-N-IE-I------E--IV--	SE-N-T----A--I
	Xenorhabdus khoisanae	WP_047963669	-N-IE-I------K---V--	SE-S-T----A---
	Xenorhabdus nematophila	WP_010848769	-N-IE-I------EL--V--	SK-N-T----A---
	Xenorhabdus szentirmaii	WP_038235614	-NKIE-I------E---V--	SK-S-T----A--I
	Xenorhabdus thuongxuanensis	WP_074020812	-NKIE-I------K---V--	SE-N-T----A---
	Osenzaea myxofaciens	WP_066749065	-NKIE-H------S--IV--	RE-N-TV---A---

Figure-S45

Partial sequence alignments of a protein Repressor LexA showing a 2aa Ins (highlighted), which is uniquely shared by all species from the Linyingensis clade.

Group	Species	Accession	131		162
**Linyingensis Clade** (***Geopseudomonas*** **gen. nov.**) **(6/6)**	*Pseudomonas linyingensis*	WP_090312804	FLRKRFDALKAHHCF	R	DDMEYSEDRGTIGDWAPL
	Pseudomonas guangdongensis	WP_090211472	------ELM------	A	-----T---NL-------
	"Pseudomonas oryzae"	WP_090349235	Y---------S----	S	-----T---E---Q----
	Pseudomonas sagittaria	WP_092432270	---------------	S	---------A--------
	Pseudomonas oryzagri	WP_229606090	Y-------L-S----	S	---------K---S----
	Pseudomonas aromaticivorans	WP_217679375	--------M-A----	S	--L--T---Q---Q----
**Other** ***Pseudomonas***	*Pseudomonas abietaniphila*	WP_074753562	Y-K--YE--RK--A-		AS---T--KA-LAE-M--
	Pseudomonas aeruginosa	WP_003161400	Y-K---ES-TK--A-		ET-V----KT-LAE-M--
	Pseudomonas aestus	WP_022640868	Y-K--HEV-SQ--A-		A----T--KAKMAE-M--
	Pseudomonas agarici	WP_017132909	--K---E--HQ--A-		AE---T---AKMSE-M--
	Pseudomonas alcaligenes	WP_061905156	--K--HEL--Q--A-		EG---T--KA-MAE-M--
	Pseudomonas alcaliphila	WP_075749591	--K---E--RQ--A-		AE---T---A--AE----
	Pseudomonas alkylphenolica	WP_038614712	--K----L--Q--A-		SE---T--KALMNE-M--
	Pseudomonas amygdali	WP_044319548	--KT--E-MSK--A-		SS---T--KAKLAE-M--
	Pseudomonas anguilliseptica	WP_090375934	Y-K---E--T---A-		AE---T---A-MAE-M--
	Pseudomonas antarctica	WP_064450734	--K---ET-SQ--A-		S--H-T---NEMAE-M--
	Pseudomonas argentinensis	WP_074881147	Y-K---E--HV--A-		E--V-----A-MTE-M--
	Pseudomonas arsenicoxydans	WP_090180159	--KE--KT-SK--A-		S----T--KA-MAE-M--
	Pseudomonas asiatica	WP_085721527	--K--YE--Q-SPL-		RG-Q----PAQ-AK-V--
	Pseudomonas asiatica	WP_100413025	--K--YEL--Q--A-		A----T--KAVMK--M--
	Pseudomonas asplenii	WP_090202160	--K---EV-HQ--A-		A----T---AKMAE-M--
	Pseudomonas asturiensis	WP_073172401	--KT--E-MRK--A-		SS---T--KA-LAE-M--
	Pseudomonas asuensis	WP_188864844	------E--S-NPL-		AG---T---AKLEE-M--
	Pseudomonas atacamensis	WP_136492723	--KE--NV-SK--A-		A----T--K-KMAE-M--
	Pseudomonas avellanae	WP_005615230	--KT--E-MSK--A-		SS---T--KA-LAE-M--
	Pseudomonas azotifigens	WP_028240666	Y--R-H---SR--A-		A--A----HV-LA--M--
	Pseudomonas azotoformans	WP_033899397	--K---ES-SQ--A-		S--H-T---SEMAE-M--
	Pseudomonas baetica	WP_095190596	--KD---V-SK--A-		S----T--KAKMAE-M--
	Pseudomonas balearica	WP_074519932	Y-K---E-ITQ--A-		A--T-----A-LAE-M--
	Pseudomonas batumici	WP_040070963	--K---ES-HP--A-		AE---T---AKLAE-M--
	Pseudomonas benzenivorans	WP_090448717	Y-K---K--T---A-		AA-------A-LAK-M--
	Pseudomonas bohemica	WP_110948186	Y-K--YE--RK--A-		AS---T--KAKLAE-M--
	Pseudomonas borbori	WP_090504108	--KT--K--TQ--A-		A----T---A-MAK-M--
	Pseudomonas brassicacearum	WP_025215436	--K---ELMSQ--A-		A----T--KAKMAE-M--
	Pseudomonas brassicae	WP_163949075	--K---ES--P--A-		AE---T--KALMNE-M--
	Pseudomonas brenneri	WP_032860070	--K---ET-SQ--A-		S--H-T---AEMAE-M--
	Pseudomonas bubulae	WP_095001565	--K----V-SK--A-		A----T--KAKMTE-M--
	Pseudomonas canadensis	WP_028618871	--K---ES-SQ--A-		S--H-T---SEMAE-M--
	Pseudomonas cannabina	WP_055000621	--KT--E-MSK--A-		SS---T--KARLAE-M--
	Pseudomonas capeferrum	WP_033699799	--K--YE--Q-SPL-		RG-Q----PAQ-AK-V--
	Pseudomonas capeferrum	WP_033701303	--K---EL--Q--A-		TE---T--KAVMK--M--
	Pseudomonas caricapapayae	WP_055009809	--KT--E-MSR--A-		SS---T--KVKLAE-M--
	Pseudomonas carnis	WP_034126319	--K---ET-SQ--A-		S--H-T---SEMAE-M--
	Pseudomonas caspiana	WP_087272997	--KT--E-MRT--A-		SS---T--KA-LAE-M--
	Pseudomonas cedrina	WP_076951199	--K---ET-SR--A-		S--H-T---SEMAE-M--
	Pseudomonas cerasi	WP_065349120	--KT--E-MSK--A-		SS---T--KAKLAE-M--
	Pseudomonas chengduensis	WP_021489382	--K---E--RQ--A-		TE---T------AE----
	Pseudomonas chloritidismutans	WP_080950556	Y-KR------T--A-		A--------A-LAE-M--
	Pseudomonas chlororaphis	WP_009050773	--K--YEV-SK--A-		A----T--KAKMAE-M--
	Pseudomonas cichorii	WP_025258629	--KT--E-MHK--A-		SS---T--KAKLAE-M--
	Pseudomonas citronellolis	WP_074982295	-----YE--Q-SPL-		RP-Q----PAQ-AK-V--
	Pseudomonas coleopterorum	WP_090360243	--K--YEL--T--A-		A----T--KAKMAE-M--
	Pseudomonas composti	WP_036999034	--K---E--RQ--A-		TE---T---A--AE----
	Pseudomonas congelans	WP_054993142	--KT--E-MSK--A-		SS---T--KA-LAE-M--
	Pseudomonas coronafaciens	WP_005891221	--KT--E-MSR--A-		SS---T--KAKLAE-M--
	Pseudomonas corrugata	WP_053191812	--K---EL-SQ--A-		A----T--K-KMAE-M--
	Pseudomonas costantinii	WP_071485992	--K---ET-SQ--A-		S--H-T---SEMAE-M--
	Pseudomonas cremoricolorata	WP_038412066	--K---EL--Q--A-		AE-Q-T--KAVMQQ-M--
	Pseudomonas cremoris	WP_185708377	--K---ES-SQ--A-		S--H-T---SEMAE-M--
	Pseudomonas cuatrocienegasensi	WP_069520091	Y-K---A--SV--A-		S----T---A-MAE-M--
	Pseudomonas daroniae	WP_131180826	Y-K---E--RV--A-		E--V-----D-MAQ-M--
	Pseudomonas deceptionensis	WP_048359206	--K---EV-SK--A-		S----T--KAKMTE-M--
	Pseudomonas denitrificans	WP_003099345	Y-K---ES-TK--A-		ET-V----KA-LAE-M--
	Pseudomonas donghuensis	WP_010225762	--K---EL--Q--A-		AE---T--KAVMNE-M--
	Pseudomonas dryadis	WP_131175211	Y-KR--E--T---A-		A----T---A-MTQ-M--
	Pseudomonas edaphica	WP_099548402	--K---ET-SQ--A-		SE-H-T---NEMAE-M--
	Pseudomonas endophytica	WP_055104190	--K--YEV-SQ--A-		T----T--KAVMNE-M--
	Pseudomonas entomophila	WP_011532228	--K---EL--Q--A-		A----T--KAVMNE-M--
	Pseudomonas extremaustralis	WP_010565724	Y-----ET-SK--A-		S--R-T---NQMAE-M--
	Pseudomonas extremorientalis	WP_071491160	--K---ET-SQ--A-		S--H-T---SEMAE-M--
	Pseudomonas ficuserectae	WP_002552176	--KT--E-MSK--A-		SS---T--KAKLAE-M--
	Pseudomonas fildesensis	WP_048723921	--K---ET-SQ--A-		S--H-T---SEMAE-M--
	Pseudomonas flavescens	WP_084307772	Y-K---E--R---A-		A--V-----D-MAQ-M--
	Pseudomonas flexibilis	WP_039559666	-----YE---D-A--		-G------HAKLRE-M--
	Pseudomonas floridensis	WP_083185502	--KT--E-MRK--A-		SS---T--KT-LAE-M--
	Pseudomonas fluorescens	WP_053254438	--K---ET-SQ--A-		S--H-T---NEMAE-M--
	Pseudomonas fluvialis	WP_093986028	Y----YA--RD----		AQ-D---E-A-LQQ-M--

Other *Pseudomonas*	*Pseudomonas fragi*	WP_016779489	--K---EV-SK--A-	A----T--KARMTE-M--
	Pseudomonas frederiksbergensis	WP_071553369	--KE-YKV-SK--A-	A----T--KAKMA--M--
	Pseudomonas fulva	WP_013792483	Y-K---E--HV--A-	E--V-----A-MNE-M--
	Pseudomonas furukawaii	WP_003456653	--K---EL-RQ--A-	SE---T---A-MAK----
	Pseudomonas fuscovaginae	WP_081354380	--K---EV-HQ--A-	A----T---AKMAE-M--
	Pseudomonas gessardii	WP_076963531	--K---ET-SQ--A-	S--H-T---AEMAE-M--
	Pseudomonas graminis	WP_074884008	Y-K--YE--RP--A-	SS---T--KARLAE-M--
	Pseudomonas granadensis	WP_090286193	--KE--NV-SK--A-	A----T--K-KMAE-M--
	Pseudomonas grimontii	WP_090409388	--K---ET-SQ--A-	S--H-T---NEMAE-M--
	Pseudomonas guariconensis	WP_070576955	--K---EL--Q--A-	SE---T--KAVMEQ-M--
	Pseudomonas guguanensis	WP_090433618	--K---E--RQ--A-	TE---T---A--AE----
	Pseudomonas guineae	WP_090243687	--K---A--T---A-	SE-------A-MAE-M--
	Pseudomonas haemolytica	WP_153870195	--K---ET-SQ--A-	S--H-T---SEMAE-M--
	Pseudomonas helleri	WP_048367240	--K----V-SK--A-	A----T--KAKMAE-M--
	Pseudomonas helmanticensis	WP_134177960	--KS---V-SK--A-	A----T--K-KMAE-M--
	Pseudomonas hunanensis	WP_003247357	--K---EL--Q--A-	AE---T--KAVMN--M--
	Pseudomonas hydrolytica	WP_129482950	--K---E--RQ--A-	TEI--T---A--AE----
	Pseudomonas indica	WP_084335469	Y-K---A--SQ--A-	AE-------A-LAE-M--
	Pseudomonas indoloxydans	WP_108232931	--K---E--RQ--A-	AE---T---A--AE----
	Pseudomonas inefficax	WP_025337698	--K--YEL--Q--A-	A----T--KAVMN--M--
	Pseudomonas japonica	WP_042127104	--K--YEL--Q--A-	AE---T--QSVMKE-M--
	Pseudomonas jessenii	WP_057713331	--KE--KV-SK--A-	A----T--KAKMAE-M--
	Pseudomonas jinjuensis	WP_090416175	--K--Y---Q-SPL-	RP-Q----HAQ-AK-V--
	Pseudomonas juntendi	WP_054881708	--K---EL--Q--A-	SE---T--KAVMN--M--
	Pseudomonas kairouanensis	WP_135287443	Y-K---ET-SQ--A-	S--H-T---NQMAE-M--
	Pseudomonas khazarica	WP_134675675	--K---E--RQ--A-	T----T---A--AE----
	Pseudomonas kilonensis	WP_018606499	--K---ELMHQ--A-	A----T--KARMAE-M--
	Pseudomonas kirkiae	WP_131185553	Y-K----V-TQ--A-	A--V-----QVLAE-M--
	Pseudomonas knackmussii	WP_043251585	--K--Y---Q-SPL-	RP-Q----HAQ-AK-V--
	Pseudomonas koreensis	WP_024014277	--KE--NV-SK--A-	A----T--K-KMAE-M--
	Pseudomonas kribbensis	WP_114885473	--KE--NT-HK--A-	A----T--KAKMAE-M--
	Pseudomonas kunmingensis	WP_080950556	Y-KR------T--A-	A--------A-LAE-M--
	Pseudomonas kuykendallii	WP_090228757	Y-K---A--TG--A-	SS------KA-LAR-M--
	Pseudomonas lactis	WP_057710936	--K---ET-SQ--A-	S--H-T---SEMAE-M--
	Pseudomonas laurylsulfatiphila	WP_104449388	--KE--KV-SK--A-	A----T--KA-MAE-M--
	Pseudomonas laurylsulfatovoran	WP_103396208	--KE--KV-SK--A-	-----T--KAKMAE-M--
	Pseudomonas leptonychotis	WP_136665275	--K---A---T--A-	TE---T---A-MAE-M--
	Pseudomonas libanensis	WP_057012943	--K---ET-SQ--A-	S--H-T---SEMAE-M--
	Pseudomonas lini	WP_048397770	--KE--KT-SK--A-	S----T--KA-MAE-M--
	Pseudomonas lundensis	WP_047273723	--K----V-SQ--A-	A----T--KAKMAE-M--
	Pseudomonas lurida	WP_034102224	--K---ET-SQ--A-	S--H-T---SEMAE-M--
	Pseudomonas lutea	WP_037018294	Y-K--YE--RT--A-	SS---T--KARLAE-M--
	Pseudomonas luteola	WP_073450725	------E--S-NPL-	AG---T---AKLEE-M--
	Pseudomonas mandelii	WP_010458221	--KE--KT-SK--A-	S----T--KA-MA--M--
	Pseudomonas marginalis	WP_012722258	--KQ--ET-SQ--A-	S--H-T---NEMAE-M--
	Pseudomonas marincola	WP_090512915	--KN--N--T---A-	SE---T---A-MAE-M--
	Pseudomonas mediterranea CFBP	WP_047701389	--K---EL-SQ--A-	A----T--K-KMAE-M--
	Pseudomonas meliae	WP_005733240	--KT--E-MSK--A-	SS---T--KAKLAE-M--
	Pseudomonas mendocina	WP_003240435	--K---E--RQ--A-	TE---T---A--AE----
	Pseudomonas migulae	WP_084320438	--KE--KV-SK--A-	S----T--KAKMA--M--
	Pseudomonas mohnii	WP_047533102	--KE--KV-SK--A-	SS---T--KAKMAE-M--
	Pseudomonas monteilii	WP_013973787	--QQ--RQMS----Y	QN----D-LKQ-DE----
	Pseudomonas moorei	WP_090325096	--KE--KV-SK--A-	A----T--KA-MAE-M--
	Pseudomonas moraviensis	WP_064362137	--KE--NV-SK--A-	A----T--K-KMAE-M--
	Pseudomonas mosselii	WP_062360541	--K--Y---Q-SPL-	RP-Q----HAQ-AK-V--
	Pseudomonas mucidolens	WP_084376279	--K---ET-SQ--A-	S--H-T---AQMAE-M--
	Pseudomonas nabeulensis	WP_135307826	--K---ES-SQ--A-	S--H-T---SEMAE-M--
	Pseudomonas nitrititolerans	WP_083237780	--KR------T--A-	E--V-T---A-MAE-M--
	Pseudomonas nosocomialis	WP_138407269	Y-H--YE--RS--A-	A--V-T---A-MA--M--
	Pseudomonas oleovorans	WP_037050955	--K---E--RQ--A-	T----T---A--AE----
	Pseudomonas orientalis	WP_057724589	--K---ET-SR--A-	S--H-T---SEMAE-M--
	Pseudomonas oryzihabitans	WP_059314954	-----YE---D-A--	-G------HAKLRE-M--
	Pseudomonas otitidis	WP_074973469	--K--YE--Q-SPL-	RG-Q----PAQ-AK-V--
	Pseudomonas ovata	WP_056841687	--HQ-YRQMS-N--Y	EN-Q----KQQ-E-----
	Pseudomonas palleroniana	WP_090366724	--K---ET-SQ--A-	S--H-T---SEMAE-M--
	Pseudomonas panacis	WP_057004261	------ET-SQ--A-	S--R-T---NEMVE-M--
	Pseudomonas panipatensis	WP_090261626	--KT-QQQ-S---A-	ET---T---EL-AK----
	Pseudomonas parafulva	WP_028631670	--K--YEL--Q--A-	A----T--KAVMNE-M--
	Pseudomonas paralactis	WP_057701579	--K---ET-SQ--A-	S--H-T---SEMAE-M--
	Pseudomonas peli	WP_090255240	Y-K---Q--TV--A-	A----T--KA-LAE-M--
	Pseudomonas pharmafabricae	WP_101193070	Y----YA--RD----	AQ-D---E-A-LQQ-M--
	Pseudomonas piscis	WP_053132011	Y-K--HEV-SQ--A-	A----T--KAKMAE-M--
	Pseudomonas plecoglossicida	WP_013973787	--K--Y---Q-SPL-	RP-Q----HAQ-AK-V--
	Pseudomonas plecoglossicida	WP_023662527	--K--YEL--Q--A-	A----T--KAVMK--M--
	Pseudomonas poae	WP_060548331	--K----T-VQ--A-	S--H-T----EMAE-M--
	Pseudomonas pohangensis	WP_090192945	--K---E--------	SN-----E-S--AG----
	Pseudomonas prosekii	WP_092278111	--KS--EV-SK--A-	A----T--KAKMAE-M--
	Pseudomonas protegens	WP_011063381	--K---EV-SQ--A-	A----T--KAKMAE-M--

Other *Pseudomonas*	*Pseudomonas proteolytica*	WP_029291742	--K---ET-SQ--A-	S--H-T---AEMAE-M--
	Pseudomonas pseudoalcaligenes	WP_004424538	--K---E--RQ--A-	AE---T---A--AE----
	Pseudomonas psychrophila	WP_048351164	--K---EV-SK--A-	S----T--KAKMTE-M--
	Pseudomonas psychrotolerans	WP_058782805	--HQ-YRQMS-N--Y	EN------KKQ-E-----
	Pseudomonas pudica	WP_196173999	--K--YEL--Q--A-	AE---T--KAVMK--M--
	Pseudomonas punonensis	WP_073261610	Y-K------RV--A-	E--V-----D-MHQ-M--
	Pseudomonas putida	WP_016498351	--K--Y---Q-SPL-	RP-Q----HAQ-AK-V--
	Pseudomonas qingdaonensis	WP_043864347	--K---ES--P--A-	AE---T--KALMNE-M--
	Pseudomonas reactans	WP_177002418	--K---ET-SQ--A-	S--H-T---SEMAE-M--
	Pseudomonas reidholzensis	WP_119143974	--K--Y---Q-SPL-	RP-Q----HAQ-AK-V--
	Pseudomonas reinekei	WP_075945671	--KE--KV-SK--A-	A----T--KA-MAE-M--
	Pseudomonas resinovorans	WP_016494666	--K---EL-RQ--A-	SE---T-----VAK----
	Pseudomonas rhizoryzae	WP_058771943	-----YE---D-A--	-G------HAKLRE-M--
	Pseudomonas rhizosphaerae	WP_043192940	--K--YEL-----A-	A----T--KTKMAE-M--
	Pseudomonas rhodesiae	WP_034136998	--K---ET-SQ--A-	S--H-T---SEMAE-M--
	Pseudomonas salomonii	WP_069786082	--K---ET-SQ--A-	S--H-T---NEMAE-M--
	Pseudomonas saponiphila	WP_092310406	--K---EV-SQ--A-	A----T--KAKMAE-M--
	Pseudomonas saudiphocaensis	WP_074436848	Y-KR------Q--A-	EN-V-T---A-MQE-M--
	Pseudomonas savastanoi	WP_002552176	--KT--E-MSK--A-	SS---T--KAKLAE-M--
	Pseudomonas saxonica	WP_146384276	--K----V-SQ--A-	A----T--KAVMNE-M--
	Pseudomonas sediminis	WP_099526152	--K---E--RQ--A-	TE---T---A--AE----
	Pseudomonas segetis	WP_010486306	--KT--E--SK--A-	EQ---T---E-MAQ-M--
	Pseudomonas seleniipraecipitan	WP_092365819	Y-K---E--RV--A-	A--V-----D-MAQ-M--
	Pseudomonas shirazica	WP_013970927	--K--YEL--Q--A-	A----T--KAVMK--M--
	Pseudomonas sichuanensis	WP_110994789	--K---EL--Q--A-	SE---T--KAVMN--M--
	Pseudomonas sihuiensis	WP_092377521	--K---E--RQ--A-	TE---T------AE----
	Pseudomonas silesiensis	WP_064675930	--KE--ELMSK--A-	A----T--KA-MAE-M--
	Pseudomonas simiae	WP_021491877	--K---ET-SQ--A-	S--H-T---SEMAE-M--
	Pseudomonas sivasensis	WP_095183855	--K---ET-SQ--A-	S--H-T---SEMAE-M--
	Pseudomonas soli	WP_023630753	--K---EL--Q--A-	AE---T--KAVMK--M--
	Pseudomonas songnenensis	WP_106155672	Y-KR------T--A-	A--------A-L-E-M--
	Pseudomonas straminea	WP_093503826	Y-K---E--HV--A-	E--V-----A-MSE-M--
	Pseudomonas stutzeri	WP_080505043	Y-KR------T--A-	A--------A-LAE-M--
	Pseudomonas synxantha	WP_057021547	--K---ET-SQ--A-	S--H-T---SEMAE-M--
	Pseudomonas syringae	WP_024643630	--KT--E-MSK--A-	SS---T--KARLAE-M--
	Pseudomonas taeanensis	WP_025166732	--K---A--TQ--A-	AA---T---A-MAE-M--
	Pseudomonas taetrolens	WP_048378186	--K---EV-SK--A-	S----T--KAKMAE-M--
	Pseudomonas taiwanensis	WP_023381787	--K--Y---Q-SPL-	RP-Q----HAQ-AK-V--
	Pseudomonas thermotolerans	WP_017936908	--KT-QQQ-S---A-	ET---T---EL-AK----
	Pseudomonas thivervalensis	WP_053125164	--K---ELMRQ--A-	A----T--KDQMAE-M--
	Pseudomonas tolaasii	WP_080520114	--K---ES-SQ--A-	S--H-T---SEMAE-M--
	Pseudomonas toyotomiensis	WP_074916394	--K---E--RQ--A-	TE---T---A--AE----
	Pseudomonas tremae	WP_005891221	--KT--E-MSR--A-	SS---T--KAKLAE-M--
	Pseudomonas trivialis	WP_049709961	--K---ET-SQ--A-	S--H-T---SEMAE-M--
	Pseudomonas tuomuerensis	WP_039559666	-----YE---D-A--	-G------HAKLRE-M--
	Pseudomonas typographi	WP_190418580	--KA--ET-RQ--A-	AA-Q-T--KA-LAE-M--
	Pseudomonas umsongensis	WP_020799762	--KE--NV-HK--A-	A----T--KA-MAE-M--
	Pseudomonas vancouverensis	WP_093213773	--KE--NV-SK--A-	A----T--KAKMAE-M--
	Pseudomonas veronii	WP_057004261	------ET-SQ--A-	S--R-T---NEMVE-M--
	Pseudomonas versuta	WP_060690720	--K---EV-SK--A-	S----T--KAKMAE-M--
	Pseudomonas viridiflava	WP_058430999	--KT--E-MRK--A-	SS---T--KAKLAE-M--
	Pseudomonas vranovensis	WP_028944113	--K---EL--Q--A-	SE---T--KALMSE-M--
	Pseudomonas wadenswilerensis	WP_115088737	--K---EM--Q--A-	AE-D-T--KALMNE-M--
	Pseudomonas weihenstephanensis	WP_048362525	--K---EV-SK--A-	A----T--KAKMAE-M--
	Pseudomonas xanthomarina	WP_073301326	--KR------T--A-	E--V-----A-MEE-M--
	Pseudomonas yamanorum	WP_093205211	--K---ET-SQ--A-	S--Y-T---SEMAE-M--
	Pseudomonas zeshuii	WP_073450725	------E--S-NPL-	AG---T---AKLEE-M--
	Pseudomonas zhaodongensis	WP_128121018	Y-KR--N---T--A-	E--V-----A-MEE-M--
Other Bacteria	*Azomonas agilis*	WP_144570032	--KT-YE--RQ--A-	AA------FA-LKA-M--
	Azomonas macrocytogenes	WP_183167807	Y-K--HEL-S---A-	A--V-----A--A--V--
	Azotobacter beijerinckii	WP_090622571	--KT--E---T----	A----T--QA-LNE-I--
	Azotobacter chroococcum	WP_089167302	--KT--EV--K----	A----T--KA-LEE-M--
	Azotobacter salinestris	WP_152387161	--KT---V--K----	A----T--KA-LDE-M--
	Azotobacter vinelandii	WP_012702639	Y-KT--EV--K----	A----T--QA-LDE-M--
	Proteus mirabilis	WP_004243955	--KQ--KQMS----Y	HN--FTD-PKQ-NE-T--
	Proteus penneri	WP_006537409	--QQ-YRQMS----Y	QN----D-PKQ-NE----
	Providencia burhodogranariea	WP_008912252	--QQ-YRQMS----Y	KN----D-PKQ-NE----
	Providencia heimbachae	WP_068908556	--QQ--RQMS----Y	HN----D-PKQ-NE----
	Providencia rettgeri	WP_042845787	--QQ--RQMS----Y	HN----D-PKQ-D-----
	Providencia sneebia	WP_008914004	--K--Y---Q-SPL-	RP-Q----HAQ-AK-V--
	Providencia stuartii	WP_004915691	------H--QKSTL-	RG------PQQ-AQ-V--
	Shimwellia blattae	WP_002443436	--K--Y---Q-SPL-	RP-Q----HAQ-AK-V--

Figure- S46

Partial sequence alignments of a protein Malate dehydrogenase (quinone) showing a 1aa Ins (highlighted), which is uniquely shared by all species from the Linyingensis clade.

Group	Species	Accession	629		676
**Linyingensis Clade (*Geopseudomonas* gen. nov.) (6/6)**	*Pseudomonas linyingensis*	WP_090313706	REEQIRENQHAALDLL	R	ARDVVDSDAAEQLWTQLGDD
	Pseudomonas guangdongensis	WP_090215645	---L---H------R-	-	Q-GT--P--V----S-----
	"Pseudomonas oryzae"	WP_090348882	---R------------	-	S-GQ--G-------A-----
	Pseudomonas sagittaria	WP_092435482	----------------	-	--N-I---------S-----
	Pseudomonas oryzagri	WP_229606915	---R------------	-	G-GQV-G-------A-----
	Pseudomonas aromaticivorans	WP_217681373	---R---------K--	H	--EDI-A--V----A-----
**Other *Pseudomonas***	*Pseudomonas abietaniphila*	WP_074752737	------RT-S----I-		V-NGT-P-DV----S-----
	Pseudomonas abyssi	WP_096002971	---H--QT-Q----I-		V-NG--E-D--A--DE----
	Pseudomonas aeruginosa	WP_003098610	------QT-T----Q-		V-NGI-Q-D-----S-----
	Pseudomonas aestus	WP_022642572	------QT-S----I-		V-NGT-P-EV----S-----
	Pseudomonas aestusnigri	WP_088273391	---H--QT-Q----I-		V-NGIEEED--D--SE--E-
	Pseudomonas agarici	WP_060782210	------QT-S----I-		V-AGT-P-DV----S-----
	Pseudomonas alcaligenes	WP_061903633	------QT-S--I-I-		V-GGI-Q-D-----S-----
	Pseudomonas alcaliphila	WP_075748738	------QT-S----T-		V-GGT-P-D-----S-----
	Pseudomonas alkylphenolica	WP_038614015	------QT-G----I-		I-EGT-P-DV----A-----
	Pseudomonas allii	WP_179029378	------RT-S----I-		V-GGN-P-DV----S-----
	Pseudomonas amygdali	WP_002554732	------RT-T----I-		V-NGT-P-DV----SA----
	Pseudomonas anguilliseptica	WP_090383216	------LT-I----I-		V-GGT-P-D-----S-----
	Pseudomonas antarctica	WP_064451151	------RT-S----I-		V-GGN-P-DV----S-----
	Pseudomonas argentinensis	WP_074881177	------LT-S----T-		INGGT-P-DV----S-----
	Pseudomonas arsenicoxydans	WP_090187840	--M---QT-S----I-		V-GGT-P-DV----S-----
	Pseudomonas asiatica	WP_054573194	------QT-S----I-		V-EGT-P-DV----S-----
	Pseudomonas asplenii	WP_010444748	------QT-S----I-		V-TGT-P-DV----S-----
	Pseudomonas asturiensis	WP_073163993	------RT-I----I-		V-NGT-P-DV----SA----
	Pseudomonas asuensis	WP_188864261	------QT-SS-I-I-		V-NGI-Q-D--H--G-----
	Pseudomonas atacamensis	WP_016771478	------QT-S----I-		V-GGT-P-DV----A-----
	Pseudomonas avellanae	WP_081591433	------RT-T----I-		V-SGT-P-DV----SA----
	Pseudomonas azotifigens	WP_028240001	------RT-Q----Q-		V-SGT-P-D-----A-----
	Pseudomonas azotoformans	WP_061435544	------RT-S----I-		V-GGN-P-DV----S-----
	Pseudomonas baetica	WP_100847328	------QT-S----I-		V-GGT-P-DV----A-----
	Pseudomonas balearica	WP_041104466	------QS-R----T-		V-NGT-P-D-----A-----
	Pseudomonas batumici	WP_040064751	------QT-S----I-		V-AGT-P-DV----S-----
	Pseudomonas benzenivorans	WP_090443179	------LT-SS---I-		V-SGT-P-D-----S-----
	Pseudomonas bohemica	WP_110951346	------RT-S----I-		V-NGT-P-DV----S-----
	Pseudomonas borbori	WP_090500227	------LT-S----I-		V-GGT-P-E-----SK----
	Pseudomonas brassicacearum	WP_025212112	------RT-S----I-		V-GGT-P-DV----S-----
	Pseudomonas brassicae	WP_163940222	------QT-S----I-		I-EGT-P-DV----S-----
	Pseudomonas brenneri	WP_032858169	------RT-S----I-		V-GGN-P-DV----S-----
	Pseudomonas bubulae	WP_095001306	------QT-S----I-		V-GGT-Q-DV----S-----
	Pseudomonas canadensis	WP_028619200	------RT-S----I-		V-GGN-P-DV----S-----
	Pseudomonas cannabina	WP_055001132	------RT-I----I-		V-NGT-P-DV----SA----
	Pseudomonas capeferrum	WP_033700887	------QT-S----I-		I-EGT-P-DV----S-----
	Pseudomonas caricapapayae	WP_055010694	------RT-T----I-		V-NGT-P-DV----SA----
	Pseudomonas carnis	WP_034126830	------RT-S----I-		V-GGN-P-DV----S-----
	Pseudomonas caspiana	WP_087274045	------RT-I----I-		V-NGT-P-DV----S-----
	Pseudomonas cedrina	WP_076950022	------RT-S----I-		V-GGN-P-DV----S-----
	Pseudomonas cerasi	WP_003364865	------RT-T----I-		V-NGT-P-DV----SA----
	Pseudomonas chengduensis	WP_017674793	------QT-S----T-		V-GGT-P-D-----S-----
	Pseudomonas chloritidismutans	WP_023443743	------QT-R----D-		V-QGT-P-D-----S-----
	Pseudomonas chlororaphis	WP_038630713	------QT-S----I-		V-AGT-P-DV----A-----
	Pseudomonas cichorii	WP_025258942	------RT-I----I-		V-NGI-P-DV----SA----
	Pseudomonas citronellolis	WP_074985303	------QT-S--I-I-		V--GI-Q-D-----S-----
	Pseudomonas coleopterorum	WP_056843854	------RT-S---EI-		V-GGT-Q-DI----S-----
	Pseudomonas composti	WP_074937023	------QT-N----T-		V-GGT-P-D-----S-----
	Pseudomonas congelans	WP_032616020	------RT-T----I-		V-NGT-P-DV----SA----
	Pseudomonas coronafaciens	WP_005891811	------RT-I----I-		V-NGT-P-DV----SA----
	Pseudomonas corrugata	WP_055135913	------RT-S----I-		V-GGT-P-DV----S-----
	Pseudomonas costantinii	WP_071487454	------RT-S----I-		V-GGN-P-DV----S-----
	Pseudomonas cremoricolorata	WP_038412338	---L--QT-SS---I-		I-EGT-P-DV----S-----
	Pseudomonas cremoris	WP_185707402	------RT-S----I-		V-GGN-P-DV----S-----
	Pseudomonas cuatrocienegasensi	WP_069516098	------VT-S----I-		V-GGT-P-D-----S-----
	Pseudomonas daroniae	WP_131190298	------LT-S----T-		V-SGT---EV----A-----
	Pseudomonas deceptionensis	WP_048359910	------QT-S----I-		V-GGT-Q-DV----S-----
	Pseudomonas delhiensis	WP_089391577	------QT-S--I-I-		V--GI-Q-D-----S-----
	Pseudomonas denitrificans	WP_003113862	------QT-T----Q-		V-NGI-Q-D-----S-----
	Pseudomonas donghuensis	WP_010224852	------QT-S----I-		I-EGT-P-DV----S-----
	Pseudomonas dryadis	WP_131177923	------LT-S----I-		V-GGT---EV----A-----
	Pseudomonas duriflava	WP_145140064	------QT-AS-I-T-		V-NGI-Q-D-----N-----
	Pseudomonas edaphica	WP_017137139	------RT-S----I-		V-GGN-P-DV----S-----
	Pseudomonas endophytica	WP_055102583	------QT-R----I-		V-NGN-Q-DV----S-----
	Pseudomonas entomophila	WP_011535282	------QT-S----I-		V-EGT-P-DV----S-----
	Pseudomonas extremaustralis	WP_010563518	------RT-S----I-		V-GGN-P-DV----S-----
	Pseudomonas extremorientalis	WP_071488797	------RT-S----I-		V-GGN-P-DV----S-----
	Pseudomonas ficuserectae	WP_005760201	------RT-T----I-		V-NGT-P-DV----SA----
	Pseudomonas fildesensis	WP_048719958	------RT-S----I-		V-GGN-P-DV----S-----
	Pseudomonas flavescens	WP_084304217	------LT-S----T-		V-GGT---DV----A-----
	Pseudomonas floridensis	WP_083183500	------RT-I----I-		V-NGT-P-DV----SA----
	Pseudomonas fluorescens	WP_053254734	------RT-S----I-		V-GGN-P-DV----S-----
	Pseudomonas fluvialis	WP_093986270	------QT-DS-RE--		IAAEQ-A-Q--L--S-----
	Pseudomonas fragi	WP_016781979	------QT-S----I-		V-GGT-Q-DV----S-----

	Pseudomonas frederiksbergensis	WP_071551645	------QT-S----I-	V-GGT-P-DV----S-----
	Pseudomonas fulva	WP_013792520	------LT-A----I-	TAAST-A-DV----S-----
	Pseudomonas furukawaii	WP_004421071	------QT-S--I-I-	V-NGI-Q-E-----S-----
	Pseudomonas fuscovaginae	WP_019361581	------QT-S----I-	V-TGT-P-DV----S-----
	Pseudomonas gallaeciensis	WP_118130868	---H--QT-Q----I-	V-NG--E-D--A--DE----
	Pseudomonas gessardii	WP_076962167	------RT-S----I-	V-GGN-P-DV----S-----
	Pseudomonas gingeri	WP_017127117	------QT-S----I-	V-AGT-P-DV----S-----
	Pseudomonas graminis	WP_074891306	------RT-T----I-	V-SGT-P-DV----S-----
	Pseudomonas granadensis	WP_090281998	------QT-S----I-	V-GGT-P-DV----A-----
	Pseudomonas grimontii	WP_090408521	------RT-S----I-	V-GGN-P-DV----S-----
	Pseudomonas guariconensis	WP_043208884	------QT-T----I-	V-EGT-P-DV----A-----
	Pseudomonas guguanensis	WP_090431212	------QT-S----I-	V-GGT-P-D-----S-----
	Pseudomonas guineae	WP_090238892	------LT-GT---I-	V-GGT-A-V-----A-----
	Pseudomonas haemolytica	WP_153870359	------RT-S----I-	V-GGN-P-DV----S-----
	Pseudomonas helleri	WP_048368103	------QT-S----V-	V-NGN-Q-DV----S-----
	Pseudomonas helmanticensis	WP_134176912	------QT-S----I-	V-GGT-P-DV----A-----
	Pseudomonas humi	WP_069863653	------QT-S--I-I-	V--GI-Q-D-----S-----
	Pseudomonas hunanensis	WP_003252283	------QT-SS---I-	V-EGT-P-DV----A-----
	Pseudomonas hussainii	WP_074865771	---S---T-L------	Q-AGIEA-T-----A-----
	Pseudomonas hydrolytica	WP_003244188	------QT-S----I-	V-GGT-P-D-----S-----
	Pseudomonas indica	WP_084336965	------QT-S----I-	V-SGI-P-D-----S-----
	Pseudomonas indoloxydans	WP_004424004	------QT-S----I-	V-GGT-P-D-----S-----
	Pseudomonas inefficax	WP_133970537	------QT-S----I-	V-EGT-P-DV----S-----
	Pseudomonas japonica	WP_042121930	------QT-S----I-	V-EGN-Q-DV----S-----
	Pseudomonas jessenii	WP_090453382	------QT-S----I-	V-GGT-P-DV----A-----
	Pseudomonas jilinensis	WP_119701034	-----HQT-Q----I-	V-NGI-E-DVQA--------
	Pseudomonas jinjuensis	WP_084310026	------QT-S--I-I-	V-SGT-Q-D-----S-----
	Pseudomonas juntendi	WP_161892724	------QT-S----I-	V-EGT-P-DV----S-----
	Pseudomonas kairouanensis	WP_135287992	------RT-S----I-	V-GGN-P-DV----S-----
	Pseudomonas khazarica	WP_134677466	------QT-S------	V-GGI-P-D-----S-----
	Pseudomonas kilonensis	WP_053185895	------QT-S----I-	V-GGT-P-DV----S-----
	Pseudomonas knackmussii	WP_043249890	-----LQT-N--I-I-	V-EGI-Q-D-----S-----
	Pseudomonas koreensis	WP_041478131	------QT-S----I-	V-GGT-P-DV----A-----
	Pseudomonas kribbensis	WP_114881637	------QT-S----I-	V-GGT-P-DV----A-----
	Pseudomonas kunmingensis	WP_090518998	------QT-R----D-	V-QGT-P-D-----A-----
	Pseudomonas kuykendallii	WP_090226628	------QT-S----I-	--AGI-P-D-----S-----
	Pseudomonas lactis	WP_034134914	------RT-S----I-	V-GGN-P-DV----S-----
	Pseudomonas laurentiana	WP_163934210	------QT-S----I-	V-EGT-P-DV----S-----
	Pseudomonas laurylsulfatiphila	WP_104450682	------QT-S----I-	V-GGT-P-DV----A-----
	Pseudomonas laurylsulfatovoran	WP_103394421	------QT-S----I-	V-GGT-P-DV----A-----
	Pseudomonas leptonychotis	WP_136662675	------LT-GT---I-	V-GGT-A-I-----A-----
	Pseudomonas libanensis	WP_057012058	------RT-S----I-	V-GGN-P-DV----S-----
	Pseudomonas lini	WP_048393762	------QT-S----I-	V-GGN-P-DV----A-----
	Pseudomonas lundensis	WP_047282996	------QT-S----T-	V-NGN-Q-DV----S-----
	Pseudomonas lurida	WP_069022118	------RT-S----I-	V-GGN-P-DV----S-----
	Pseudomonas lutea	WP_037014268	------RT-S----I-	V-SGT-P-DV----S-----
**Other *Pseudomonas***	*Pseudomonas luteola*	WP_019364434	------QT-SS-I-I-	V-NGI-Q-D--H--G-----
	Pseudomonas mandelii	WP_010462838	--M---QT-S----I-	V-GGT-P-DV----S-----
	Pseudomonas marginalis	WP_012722593	------RT-S----I-	V-GGN-P-DV----S-----
	Pseudomonas marincola	WP_090508901	------LT-T----I-	V-TGT---E--H--S-----
	Pseudomonas massiliensis	WP_040261139	------QT-A----I-	V-GGT-P-DV----S-----
	Pseudomonas mediterranea	WP_047702013	------RT-T----I-	V-GGT-P-DV----S-----
	Pseudomonas meliae	WP_044344084	------RT-T----I-	V-NGT-P-DV----SA----
	Pseudomonas mendocina	WP_003244188	------QT-S----I-	V-GGT-P-D-----S-----
	Pseudomonas migulae	WP_084322270	--M---QT-S----I-	V-GGN-P-DV----S-----
	Pseudomonas mohnii	WP_090467442	------QT-S----I-	V-GGT-P-DV----A-----
	Pseudomonas monteilii	WP_023662319	------QT-S----I-	V-EGT-P-DV----S-----
	Pseudomonas moorei	WP_090316919	------QT-S----I-	V-GGT-P-DV----A-----
	Pseudomonas moraviensis	WP_083352097	------QT-S----I-	V-GGT-P-DV----A-----
	Pseudomonas mosselii	WP_062574941	------QT-S----I-	V-EGT-P-DV----S-----
	Pseudomonas mucidolens	WP_084377185	------RT-S----I-	V-GGN-P-DV----S-----
	Pseudomonas multiresinivorans	WP_169940936	------QT-S--I-I-	V-GGI-Q-D-----S-----
	Pseudomonas nabeulensis	WP_135308357	------RT-S----I-	V-GGN-P-DV----S-----
	Pseudomonas nitritireducens	WP_065084286	------QT-S--I-I-	V-GGI-Q-D-----S-----
	Pseudomonas nitrititolerans	WP_170911428	------QT-R----S-	V-NGT-P-E-----A-----
	Pseudomonas nitroreducens	WP_065084286	------QT-S--I-I-	V-GGI-Q-D-----S-----
	Pseudomonas nosocomialis	WP_138410020	------QT-R----D-	V-NGT-P-D-----A-----
	Pseudomonas oleovorans	WP_037053731	------QT-S------	V-GGI-P-D-----S-----
	Pseudomonas orientalis	WP_057723205	------RT-S----I-	V-GGN-P-DV----S-----
	Pseudomonas oryzihabitans	WP_059314042	------ST-QS---T-	M-RGI-A-D-----S----T
	Pseudomonas otitidis	WP_074971953	------QT-V--I-I-	V-NGI-Q-D-----A-----
	Pseudomonas ovata	WP_109511188	------RT-T----I-	V-NGT-P-DV----SG----
	Pseudomonas pachastrellae	WP_083724567	---H--QT-Q----I-	V-NG--E-D--A--VE----
	Pseudomonas palleroniana	WP_060754034	------RT-S----I-	V-GGN-P-DV----S-----
	Pseudomonas panacis	WP_057004716	------RT-S----I-	V-GGN-P-DV----S-----
	Pseudomonas panipatensis	WP_090261220	------QT-VS-I-I-	V--GI-Q-D-----S-----
	Pseudomonas parafulva	WP_028634977	------QT-S----I-	V-QGI-P-DV----S-----
	Pseudomonas paralactis	WP_057701315	------RT-S----I-	V-GGN-P-DV----S-----
	Pseudomonas peli	WP_090248399	------LT-I----I-	V-GGT-P-D-----S-----
	Pseudomonas pharmafabricae	WP_101193281	------QT-DS-RE--	IAAEQ-A-Q--L--S-----
	Pseudomonas piscis	WP_022642572	------QT-S----I-	V-NGT-P-EV----S-----
	Pseudomonas plecoglossicida	WP_023662319	------QT-S----I-	V-EGT-P-DV----S-----
	Pseudomonas poae	WP_015371407	------RT-S----I-	V-GGN-P-DV----S-----
	Pseudomonas pohangensis	WP_090195844	------QT-AE--E--	DETEI-E-K-----N-M---

Other *Pseudomonas*	*Pseudomonas prosekii*	WP_092272460	------QT-S----I-	V-GGT-P-DV----A-----
	Pseudomonas protegens	WP_015634331	------QT-S----I-	V-NGT-P-EV----S-----
	Pseudomonas proteolytica	WP_092234000	------RT-S----I-	V-GGN-P-DV----S-----
	Pseudomonas pseudoalcaligenes	WP_004424004	------QT-S----I-	V-GGT-P-D-----S-----
	Pseudomonas psychrophila	WP_048351380	------QT-S----T-	V-NGN-Q-DV----S-----
	Pseudomonas psychrotolerans	WP_074529529	------ST-QS---T-	I-RGI-A-------S----H
	Pseudomonas pudica	WP_046616138	------QT-T----I-	V-EGT-P-DV----S-----
	Pseudomonas punonensis	WP_073261683	------LT-S----T-	V-GGT-P-DV----S-----
	Pseudomonas putida	WP_016501169	------QT-S----I-	V-EGT-P-DV----S-----
	Pseudomonas qingdaonensis	WP_043863250	------QT-S----I-	V-EGT-P-DV----S-----
	Pseudomonas reactans	WP_061435544	------RT-S----I-	V-GGN-P-DV----S-----
	Pseudomonas reidholzensis	WP_119145008	---L--QT-SS---I-	I-EGT-P-DV----S-----
	Pseudomonas reinekei	WP_075946739	------QT-S----I-	V-GGT-P-DV----A-----
	Pseudomonas resinovorans	WP_016494397	------QT-S--I-I-	V-NGT-Q-D-----S-----
	Pseudomonas rhizoryzae	WP_058761978	------ST-QS---T-	I-RGI-A-------S----H
	Pseudomonas rhizosphaerae	WP_043185077	------RT-S---EI-	V-GGT-Q-DI----S-----
	Pseudomonas rhodesiae	WP_034096237	------RT-S----I-	V-GGN-P-DV----S-----
	Pseudomonas sabulinigri	WP_092288037	-D-H--QT-Q----I-	V-NGIEEED--A--SE----
	Pseudomonas salegens	WP_092384001	---HV-QT-Q----I-	V-SGI-E-D--E--V---E-
	Pseudomonas salomonii	WP_065928974	------RT-S----I-	V-GGN-P-DV----S-----
	Pseudomonas saponiphila	WP_092320025	------QT-S----I-	V-NGT-P-EV----S-----
	Pseudomonas saudiphocaensis	WP_037023328	------QT-R----S-	V-NGT-P-D-----A-----
	Pseudomonas savastanoi	WP_004667439	------RT-T----I-	V-NGT-P-DV----SA----
	Pseudomonas saxonica	WP_146385284	------QT-R----I-	V-NGN-Q-DV----S-----
	Pseudomonas sediminis	WP_099523492	------QT-S----A-	V-GGT-P-D-----S-----
	Pseudomonas segetis	WP_010486759	------LT-TS---T-	V-SGT-P-E-----A-----
	Pseudomonas seleniipraecipitan	WP_092365874	------LT-S---EM-	V--GT-A-DI----S-----
	Pseudomonas shirazica	WP_054573194	------QT-S----I-	V-EGT-P-DV----S-----
	Pseudomonas sichuanensis	WP_110992425	------QT-T----I-	V-EGT-P-DV----S-----
	Pseudomonas sihuiensis	WP_021489811	------QT-S----T-	V-GGT-P-D-----S-----
	Pseudomonas silesiensis	WP_064676099	------QT-S----I-	V-GGT-P-DV----S-----
	Pseudomonas simiae	WP_021492430	------RT-S----I-	V-GGN-P-DV----S-----
	Pseudomonas sivasensis	WP_032891658	------RT-S----I-	V-GGN-P-DV----S-----
	Pseudomonas soli	WP_094011638	------QT-S----I-	I-EGT-P-DV----S-----
	Pseudomonas songnenensis	WP_122099766	------QT-R----D-	V-HGT-P-D-----A-----
	Pseudomonas straminea	WP_093503884	------LT-A----I-	TAAGT-A-DV----S-----
	Pseudomonas stutzeri	WP_011912698	------QT-R----D-	V-HGT-P-D-----A-----
	Pseudomonas synxantha	WP_046071284	------RT-S----I-	V-GGN-P-DV----S-----
	Pseudomonas syringae	WP_024644384	------RT-I----I-	V-NGT-P-DV----SA----
	Pseudomonas taeanensis	WP_025165199	------L--S---EI-	V-GGT-P-H-----A-----
	Pseudomonas taetrolens	WP_048378862	------QT-S----I-	V-GGN-P-DV----S-----
	Pseudomonas taiwanensis	WP_023381976	------QT-T----I-	V-EGT-P-DV----S-----
	Pseudomonas thermotolerans	WP_017938794	------RT-S----I-	V-NGT-Q-D-----A-----
	Pseudomonas thivervalensis	WP_053118841	------RT-S----I-	V-GGT-P-DV----S-----
	Pseudomonas tolaasii	WP_016974401	------RT-S----I-	V-GGN-P-DV----S-----
	Pseudomonas toyotomiensis	WP_021489811	------QT-S----T-	V-GGT-P-D-----S-----
	Pseudomonas tremae	WP_054997858	------RT-I----I-	V-NGT-P-DV----SA----
	Pseudomonas trivialis	WP_049709671	------RT-S----I-	V-GGN-P-DV----S-----
	Pseudomonas typographi	WP_190418022	--G---QT------T-	V-SGI-P-EV----A-----
	Pseudomonas umsongensis	WP_033040572	------QT-S----I-	V-GGT-P-DV----A-----
	Pseudomonas vancouverensis	WP_093214868	------QT-S----I-	V-GGT-P-DV----A-----
	Pseudomonas veronii	WP_046384468	------RT-S----I-	V-GGN-P-DV----S-----
	Pseudomonas versuta	WP_060694750	------QT-S----I-	V-NGN-Q-DV----S-----
	Pseudomonas viridiflava	WP_088235803	------RT-I----I-	V-NGT-P-DV----SA----
	Pseudomonas vranovensis	WP_028942985	------QT-S----I-	I-EGT-P-DV----A-----
	Pseudomonas wadenswilerensis	WP_115088388	------QT-G----I-	I-EGT-P-DV----S-----
	Pseudomonas weihenstephanensis	WP_048363351	------QT-S----T-	V-NGN-Q-DV----S-----
	Pseudomonas xanthomarina	WP_073300844	------QT-R----N-	V-NGT-P-D-----S-----
	Pseudomonas xinjiangensis	WP_093394475	------QT-Q--V-I-	V-AG--E-D--A--SE--E-
	Pseudomonas yamanorum	WP_093201131	------RT-S----I-	V-GGN-P-DV----S-----
	Pseudomonas yangmingensis	WP_093473341	------QT-Q----I-	V-SGI-E-DV-A------E-
	Pseudomonas zeshuii	WP_010797375	------QT-SS-I-I-	V-NGI-Q-D--H--G-----
	Pseudomonas zhaodongensis	WP_128119480	------QT-R----N-	V-NGT-P-D-----S-----
Other *Bacteria*	*Azomonas agilis*	WP_144571744	-----QQT-Q------	V-GGT-P-DV-H--K-----
	Azomonas macrocytogenes	WP_183166625	------HT-I----Q-	V-SGT-P-D--I--S-----
	Azotobacter beijerinckii	WP_090620039	-----LQT-S----I-	V-SGT-P-D-----S-----
	Azotobacter chroococcum	WP_089168901	-----LQT-N----I-	V-SGT-P-D-----S-----
	Azotobacter salinestris	WP_152388962	------QT-N----I-	V-SGT-P-D--H--------
	Azotobacter vinelandii	WP_012702416	------QT-K----I-	V-SGT-P-D--H--------
	Entomomonas moraniae	WP_127163310	-Q---LQT-R----I-	T-TGI-E-EV-T----M-E-
	Oblitimonas alkaliphila	WP_053102561	-----STR-ST--S--	CSEG--A--VKK--K---EE

Figure- S47

Partial sequence alignments of a protein Uridylyltransferase showing a 1aa Ins (highlighted), which is uniquely shared by all species from the Linyingensis clade. No other *Pseudomonas* species or other bacterial species shares this CSI.

			166		203
**Linyingensis Clade (*Geopseudomonas* gen. nov.) (4/4)**	*Pseudomonas linyingensis*	WP_090307991	HRLRLLVKRVRYAAEVW		PHQLSVPPDALKAAQSALGD
	Pseudomonas guangdongensis	WP_090216024	----I----------A-		-R--Q-QGEG--L--A----
	Pseudomonas sagittaria	WP_092429214	-----------------		-----A--A-----------
	Pseudomonas aromaticivorans[*]	WP_217679170	-----------------		-Q-LTLE-EL----------
**Other *Pseudomonas***	*Pseudomonas abietaniphila*	WP_074753377	------I--------AY	PEL	S--PQGMQKR--S--G----
	Pseudomonas abyssi	WP_096005458	-K--I----T--ITDAF	PSL	SPLPA-ATTS--K------G
	Pseudomonas aeruginosa	WP_003114744	-P---RI-----GL-AY	PHD	CSIPGSLLAP----------
	Pseudomonas aestus	WP_022640098	------I--------SY	PEL	DRLPGPAIKR-----A----
	Pseudomonas agarici	WP_017132950	------I--------AY	PEL	DRLPAPALKH--T--Q----
	Pseudomonas alcaligenes	WP_061904209	-------------G-AY	PQL	IGLPRRSLGL--TT------
	Pseudomonas alcaliphila	WP_075747369	------I--------TY	PAQ	SRLSKAAQLR--R-------
	Pseudomonas alkylphenolica	WP_038609146	------I--------AY	PQL	E-AGRRLQCL--R---D--S
	Pseudomonas allii	WP_179028944	------I--------AY	PEL	DKLPAKAVSH--K--G----
	Pseudomonas antarctica	WP_064451512	------I--------AY	PEL	DKLPAKAMSR--Q--G----
	Pseudomonas argentinensis	WP_074888562	--V---I-----T--AY	PQL	SALSAAQLKL--KG-AV---
	Pseudomonas arsenicoxydans	WP_090186483	------I-----GI-AY	PEL	DRLPKAAMPR--S--A----
	Pseudomonas asiatica	WP_015269700	------I--A--GD-AY	PQL	D-AGKQLRRL--Q--GD---
	Pseudomonas asplenii	WP_090210826	------I--------AY	PEL	DRLPASALRR-----E----
	Pseudomonas asturiensis	WP_073169397	-D----I--------AY	PEL	S--PKNMRAR--S---E---
	Pseudomonas atacamensis	WP_136492983	------I-----GI-AY	PEL	DRLPKPAYKR--S--G----
	Pseudomonas avellanae	WP_005617325	-D----I--------AY	PEL	S--PKNMQAR--S--GE---
	Pseudomonas azotoformans	WP_061435820	------I--------AY	PEL	DKLPANAMSR--K--G----
	Pseudomonas baetica	WP_100847758	------I-----GI-AY	PEL	DRLPEAALPR--S--G----
	Pseudomonas batumici	WP_040065494	------I--------AY	PQL	NRLPAAAIKG--S--E----
	Pseudomonas batumici	WP_040068295	-Q--I----T--LT-AF	PEL	SPLSRDAALS--EL-----S
	Pseudomonas benzenivorans	WP_090438256	------I-----G--AY	PQS	SGLSAEIQAH--Q--A----
	Pseudomonas bohemica	WP_110949603	------I--------AY	PEL	S--PKGMQKR--S--G----
	Pseudomonas borbori	WP_090500482	------I-----G--AY	PQL	SGLSANTLAR----------
	Pseudomonas brassicacearum	WP_025212598	------I------I-AY	PEL	DRLPEATMPR--S--GT---
	Pseudomonas brenneri	WP_032863095	------I--------AY	PQL	GQLPAKAMSR--S--G----
	Pseudomonas bubulae	WP_095019094	--V---I--------AY	PEL	NQLPPLVLAR--G--Q---E
	Pseudomonas canadensis	WP_028619503	------I--------AY	PEL	DKLPANAMSR--K--G----
	Pseudomonas capeferrum	WP_033699101	------I--A--GD-AY	PQL	E-AGKKLQRL--K---D--A
	Pseudomonas caricapapayae	WP_083493226	-D----I--------AY	PQL	S--PKNMQAR--S--GE---
	Pseudomonas carnis	WP_034126714	------I--------AY	PEL	DKLPTNALSH--K------N
	Pseudomonas caspiana	WP_087266172	------I-------DAY	PEL	S--PKKMQSR-----G----
	Pseudomonas cedrina	WP_076950389	------I--------AY	PEL	SKLPANAMSR--K--G----
	Pseudomonas cerasi	WP_083188422	-D----I--------AY	PQL	S--RKNMQAR--S--GE---
	Pseudomonas chengduensis	WP_017677118	------I--------TY	PAQ	SRLSKAAQLR--L-------
	Pseudomonas chengduensis	WP_090336822	-E----A--L--C--AF	PRL	SPLP-ATFQ---QM--V---
	Pseudomonas chlororaphis	WP_009050147	------I--------AY	PEL	AELSPKAVSH-----G----
	Pseudomonas chlororaphis	WP_038630895	-Q--I----T--LNDAF	PQL	SPLSGEAAAS--TV-----S
	Pseudomonas cichorii	WP_025260597	-D----I--------AY	PQL	S--PKKLQAR------E--N
	Pseudomonas citronellolis	WP_074979762	------I------L-AY	PKD	SPLPQKVLAP--E---T--T
	Pseudomonas composti	WP_074937640	------I--------TY	PAQ	SRLNKAAQLR--R-------
	Pseudomonas congelans	WP_074594294	-D----I--------AY	PQL	S--PKNMRAR--S--GE---
	Pseudomonas coronafaciens	WP_024691216	-D----I--------AY	PEL	S--PKNMQAR--S---E---
	Pseudomonas corrugata	WP_069018506	------I------I-AY	PEL	DRLPESAMPR--S--G----
	Pseudomonas costantinii	WP_071483531	------I--------AY	PEL	DKLPANAMSR--N--G----
	Pseudomonas cremoricolorata	WP_038412866	------I-----GD-AY	PQL	D-AGKKLRSV--R--GD---
	Pseudomonas cremoris	WP_185708769	------I--------AY	PEL	DKLPANAMSR--K--G----
	Pseudomonas daroniae	WP_131192271	--I---I-----T--AY	PQL	SPLSAEQVRL--KG-AV---
	Pseudomonas deceptionensis	WP_048358660	--V---I--------AY	PEL	D-LPPLVLAR-----K---E
	Pseudomonas delhiensis	WP_089391717	------I--M--SL-AY	PKD	SPLPQKLLAP--E---T--T
	Pseudomonas denitrificans	WP_003114744	-P---RI-----GL-AY	PHD	CSIPGSLLAP----------
	Pseudomonas donghuensis	WP_010222561	------I--------AY	PQL	Q-SNPRLQK---R--GD--T
	Pseudomonas dryadis	WP_131174119	------I-----T--AY	PDL	AAVPAAQIRE--K-------
	Pseudomonas edaphica	WP_099549084	------I--------AY	PEL	DKLPAKAMQR--Q--G----
	Pseudomonas endophytica	WP_055101173	-QV--MI-----G--AY	PAL	SSVPTTMVKH--D--K---Q
	Pseudomonas entomophila	WP_011533121	------I-----GD-AY	PQL	E-ARRSLQRL--R--GD---
	Pseudomonas extremaustralis	WP_010566538	------I--------AY	PEL	DKLPAKSLLH--K--A----
	Pseudomonas extremorientalis	WP_071490043	------I--------AY	PEL	DKLPANAMSH--K--G----
	Pseudomonas ficuserectae	WP_057423048	-D----I--------AY	PEL	S--PKNMQAR-----GE---
	Pseudomonas fildesensis	WP_048731123	------I--------AY	PEL	DKLPANAMSR--N--A----
	Pseudomonas flavescens	WP_084303461	--V---I-----T--AY	PQL	SPLSAGQVRL--K--AV---
	Pseudomonas floridensis	WP_083181976	-D----I--------AY	PEL	S--PKNMQAR----------
	Pseudomonas fluorescens	WP_053255126	------I--------AY	PAL	DKLPAKAMSR--Q--G----
	Pseudomonas fragi	WP_016780106	--V---I--------AY	PEL	NQLPPLVLAR-----Q---E
	Pseudomonas frederiksbergensis	WP_071554279	------I------I-AY	PEL	DRLPEAAMPR--S--E----
	Pseudomonas frederiksbergensis	WP_081427266	-E--I--R-T--LT-AF	PEL	SPLSRDAAKS--GL-----A
	Pseudomonas fulva	WP_013791660	-QV---I-----T--AY	PQL	SPLSAAQLKL--KG-A----
	Pseudomonas furukawaii	WP_003452420	-A-------M---H-AY	PRL	SPLPAPAVAS---V------
	Pseudomonas fuscovaginae	WP_019360337	------I--------AY	PEL	DRLPASALRR-----E----
	Pseudomonas gessardii	WP_076963897	------I--------AY	PQL	GQLPAKAMSR--S--G----
	Pseudomonas gingeri	WP_017128013	------I--------AY	PEL	DRLPAPALKQ-----E----
	Pseudomonas graminis	WP_074884879	------I------G-AY	PEL	S--PKNMQRR-----G----
	Pseudomonas granadensis	WP_090285005	------I-----GI-AY	PEL	DRLPAAAYKR--S--G----
	Pseudomonas grimontii	WP_090407365	------I--------AY	PQL	DKLPAKAMSR--K--A----
	Pseudomonas guariconensis	WP_090346868	------I-----GD-AY	PQL	E-AGNKLQRL--RT-AD---
	Pseudomonas guguanensis	WP_090428914	------I--------AY	PRQ	SCLPK-AQQR--R--G----
	Pseudomonas haemolytica	WP_034118773	------I--------AY	PEL	DKLPPNAMSR--K-------
	Pseudomonas helleri	WP_048368638	-KV---I-----G--AY	PEL	DRVPKAVMKH--E--K---E
	Pseudomonas helmanticensis	WP_134176339	------I-----GI-AY	PEL	DRLPEAALAR--S--G----
	Pseudomonas humi	WP_069861088	------I------L-AY	PKD	SPLPQKVLAP--E---T--T
	Pseudomonas hunanensis	WP_003250256	------I--A--GD-AY	PQL	D-AGKKLQRL--K--GN---
	Pseudomonas hydrolytica	WP_041979386	------I--------TY	PAQ	SRLSKAAQLR--R-------
	Pseudomonas hydrolytica	WP_100219155	------I--------TY	PAH	GGLGKAAYAR--R---E---
	Pseudomonas indica	WP_084333767	------I-----GFDAY	PEL	IPVGGDE-AR----------

Other *Pseudomonas*

Pseudomonas indoloxydans	WP_108233789	------I--------TY PAQ SRLSKAAQLR--R-------
Pseudomonas inefficax	WP_133971005	------I--A--GD-AY PQL D-ASKQLRRL--Q--GD---
Pseudomonas japonica	WP_042128922	------I--A--G--AY PQL SRIGKSMRAE--R--DD--H
Pseudomonas jessenii	WP_090454548	-E--I----T--LT-AF PQL SPLSTKAAKS---V-----S
Pseudomonas jessenii	WP_090457091	------I-----GIDAY PEL DRLPKAAMPR--S--G----
Pseudomonas jilinensis	WP_165443794	--V-I----L--LLQAF APA LAMSPELLT--QQC--S---
Pseudomonas jinjuensis	WP_084310825	------I-----GL-AY PDD SPLPRKLLAP--D-------
Pseudomonas juntendi	WP_161892882	------I--A--GD-AY PQF Q-ASQKLQRL--K--AD---
Pseudomonas kairouanensis	WP_135287798	------I--------AY PEL DKLPTHAMPR--K-------
Pseudomonas khazarica	WP_134675904	------I------G-TY PLQ SGLDRAALAR-RR-------
Pseudomonas kilonensis	WP_053186696	------I------I-AY PEL DRLPEAAMPR--S--A----
Pseudomonas knackmussii	WP_043253403	------I------L-AY PKE SPLPRQVLGP--T--D---V
Pseudomonas knackmussii	WP_084166639	-SI------A---Q-AY PQL LPLPFDLAAR--RL--S--S
Pseudomonas koreensis	WP_041478383	------I-----GI-AY PEL DRLPKPAYKR--S--G----
Pseudomonas kribbensis	WP_114884594	------I-----GI-AY PEL DRLPKAALAR--S--G----
Pseudomonas lactis	WP_057710892	------I--------AY PEL DKLPANALSH--K-------
Pseudomonas laurentiana	WP_163931591	------------G--AY PQL ERLTDRLQQQ--CV-GD---
Pseudomonas laurylsulfatiphila	WP_104448936	------I-----GIDAY PEL DRLPKAAMPR--S--G----
Pseudomonas laurylsulfatovoran	WP_103396970	-Q----I-----GIDAY PEL DRLPKAAMPR--S--G----
Pseudomonas libanensis	WP_057013410	------I--------AY PEL DKLPANAMSR--K--G----
Pseudomonas lini	WP_038982192	------I-----GI-AY PEL DRLPEAALPN--S-------
Pseudomonas litoralis	WP_090272027	-Q--I----T--LTDAF PSL SPLPNDAAAS--K--A---A
Pseudomonas litoralis	WP_090272627	-Q--I----T--LTDAF PKL SPLPAAAANS--K--A---V
Pseudomonas lundensis	WP_047281713	--V---I--------AY PEL DRLPPRVLVR--E--K----
Pseudomonas lurida	WP_034102705	------I--------AY PEL DTLPAKAMSH--K--A----
Pseudomonas lutea	WP_037009459	------I--------AY PEL S--PQNMQRR-----G----
Pseudomonas mandelii	WP_010461622	------I-----GIDAY PEL DRLPDAAMPR--S--G---E
Pseudomonas marginalis	WP_064052354	------I--------AY PEL DKLPANALSR--K--G----
Pseudomonas marincola	WP_090508511	-E----------G-QAY PQL CGLSTRQQAT--R---S--T
Pseudomonas massiliensis	WP_040262590	-Q--I----T--LT-AF PRL SPLSAKGAK----V-G---S
Pseudomonas mediterranea	WP_047701558	------I------I-AY PEL DRLPEAAMPR--S--G----
Pseudomonas meliae	WP_044344881	-D----I--------AY PEL S--PKNIQAR-----GE---
Pseudomonas mendocina	WP_012018754	------I--------TY PAH GGLGKAAYAR--R---E---
Pseudomonas migulae	WP_084318810	------I-----GI-AY PEL DRLPKPALPK--S--A----
Pseudomonas mohnii	WP_090468041	------I-----GI-AY PEL DRLPKAALPR-RS--A----
Pseudomonas monteilii	WP_013971887	------I--A--GD-AY PQL D-AGKQLRRL--Q--GD---
Pseudomonas moorei	WP_090317996	------I-----GI-AY PEL DRLPAKALPR--S-------
Pseudomonas moraviensis	WP_042607905	------I-----GI-AY PQL DRLPKPAYKQ--S--G----
Pseudomonas mosselii	WP_096049817	------I-----GDQAY PQL E-AG-KLQRL-----GD---
Pseudomonas mucidolens	WP_084378161	------I--------AY PQL GKLPAKAMSR--S--G----
Pseudomonas multiresinivorans	WP_169939674	------I-----SL-AY PKE SAVPQRLLAP--D-------
Pseudomonas nabeulensis	WP_135309367	------I--------AY PEL DKLPANAMSR--K--G----
Pseudomonas nitritireducens	WP_038803745	------I-----SL-AY PKE SAVPQRLLAP--D-------
Pseudomonas nitroreducens	WP_084357354	------I-----SL-AY PKE SAVPQRLLAP--DS------
Pseudomonas oleovorans	WP_150609487	------I------G-TY PLQ SGLDRAALAR--R-------
Pseudomonas orientalis	WP_057722866	------I--------AY PEL DKLPVRAMA---K--G----
Pseudomonas otitidis	WP_044413820	-A----I--A--G-DAY PGV VEVPERTRRL--G--D---K
Pseudomonas ovata	WP_109513623	------I------S-AY PAL S--PKGMHAR--Q--G----
Pseudomonas palleroniana	WP_090366047	------I--------AY PNL NKLPAKAMSR--R-------
Pseudomonas panacis	WP_046384253	------I--------AY PEL DKLPVKAVLR--Q--G----
Pseudomonas panipatensis	WP_090260772	------I-----SL-AY PDD SPLPQRLLAP--D--AT--E
Pseudomonas parafulva	WP_028634537	------I--A--GD-AY PQL N-AGKRLQQV--R--GD---
Pseudomonas paralactis	WP_057703798	------I--------AY PEL DKLPANAMPR--K-------
Pseudomonas pelagia	WP_022963347	-Q--IR---T--LTDAF PSL SPLPNDAAAS--K--A---A
Pseudomonas pelagia	WP_022964707	-Q--I----T--LTDAF PQL SPLPTAATKS--K--A---A
Pseudomonas piscis	WP_152898788	------I--------SY PEL DRLPGPAIKR-----A----
Pseudomonas plecoglossicida	WP_023661056	------I--A--GD-AY PQL D-AGKQLRRL--Q--GD---
Pseudomonas poae	WP_060549192	------I--------AY PNL DKLPAKAVPR--K--G----
Pseudomonas prosekii	WP_092279481	------I-----GI-AY PEL DRLPKAAMPR--S--A----
Pseudomonas protegens	WP_015636552	------I-------DAY PEL DRLP-QAMKR----------
Pseudomonas proteolytica	WP_070995441	------I--------AY PQL GQLPAKAMSR--S--G----
Pseudomonas pseudoalcaligenes	WP_003460513	------I--------TY PAQ SRLSKAVQLR--R-------
Pseudomonas psychrophila	WP_019827567	--V---I--------AY PEL NQLPPLVVTR--G--Q---E
Pseudomonas pudica	WP_085600184	------I--A--GD-AY PQL D-AGKQLRRL--Q--GD---
Pseudomonas punonensis	WP_073267586	-QV---I-----T--AY PHL SPFKADQLRL--KG------
Pseudomonas putida	WP_016500555	------I--A--GD-AY PQL D-AGKKLQRL--K--GD--N
Pseudomonas qingdaonensis	WP_100634710	------I-----G--AY PQL ERLRPALRRG--R--GD---
Pseudomonas reactans	WP_177003543	------I--------AY PEL NKLPANAMSR--K--G----
Pseudomonas reidholzensis	WP_119140207	------I-----GDDAY PQL D-AG-KLQRL--R--GD---
Pseudomonas reinekei	WP_075949321	------I-----GIDAY PEL DRLPKAAMPR--S--G----
Pseudomonas resinovorans	WP_016492610	-A-------M---H-AY AGL SPITDEVAR----V------
Pseudomonas rhodesiae	WP_034096562	------I--------AY PQL DKLPANAVPR--K--AV---
Pseudomonas salomonii	WP_069788687	------I--------AY PEL DKLPAKAMQR--Q--G----
Pseudomonas saponiphila	WP_092312102	------I-------DAY PEL DRLPALAMKR-----G----
Pseudomonas savastanoi	WP_011168341	-D----I--------AY PEL S--PKNMQAR-----GE---
Pseudomonas saxonica	WP_122784467	--V---I-----G--AY PQL NELSARVLSR--Q--Q---E
Pseudomonas sediminis	WP_099523843	------I--------TY PAQ SRLSKAAQLR--R--G----
Pseudomonas segetis	WP_089359026	-D----------N--AY PKV ARLDRPGQK---Q--E---Q
Pseudomonas seleniipraecipitan	WP_092369735	--V---I-----T--AY PQL SPLSNQQLKL--RG-AV---
Pseudomonas shirazica	WP_015269700	------I--A--GD-AY PQL D-AGKQLRRL--Q--GD---
Pseudomonas sichuanensis	WP_110994349	------I-----GD-AY PQL D-AGKQLQRL--K--GD---
Pseudomonas sihuiensis	WP_074857198	------I--------TY PAQ SRLSKAVQLR--R-------
Pseudomonas silesiensis	WP_064678947	------I-----GI-AY PEL DRLPKAAMPG-----G----
Pseudomonas simiae	WP_069673329	------I--------AY PEL DKLPANAMSR--N--G----
Pseudomonas sivasensis	WP_181639709	------I--------AY PEL DKLPANAMSR--K--G----
Pseudomonas soli	WP_094011287	------I-----GD-AY PQL E-AGKKLQRL--K--GD---
Pseudomonas straminea	WP_093504361	-QV---I-----T--AY PQL SSLSAAQLKL--KG-A----
Pseudomonas synxantha	WP_057022162	------I--------AY PEL DKLPANAMSR--K--G----
Pseudomonas syringae	WP_025389405	-D----I--------AY PEL S--PKNMQAR--S--GE---
Pseudomonas taeanensis	WP_025167871	-E----T-SA---TDAY PQL SPISVETATS--RV------

Group	Species	Accession	Sequence
Other *Pseudomonas*	*Pseudomonas taetrolens*	WP_048382397	--V---I------G-AY PEL D-LPEQVVTR--T--K---Q
	Pseudomonas taiwanensis	WP_049818698	------I--A--GD-AY PQL G-TGKKLRRL--R--GD---
	Pseudomonas thermotolerans	WP_017938368	------I-----GD-AY PRL SPVGASGWA----------
	Pseudomonas thivervalensis	WP_053120446	------I------I-AY PEL NRLPEAATPR--S--G----
	Pseudomonas tolaasii	WP_016968890	------I--------AY PEL DKLPANAMSR--K--G----
	Pseudomonas toyotomiensis	WP_074914820	------I--------TY PAH SRLSKAAQLR--R-------
	Pseudomonas tremae	WP_024691216	-D----I--------AY PEL S--PKNMQAR--S---E---
	Pseudomonas trivialis	WP_049709327	------I--------AY PEL DKLPANAMSR--K--A----
	Pseudomonas typographi	WP_190419992	------I--A-----AY PRR Q-LPAALAK---R--RS---
	Pseudomonas umsongensis	WP_020796125	------I-----GIDAY PEL DRLPKAAMPR--S--G----
	Pseudomonas vancouverensis	WP_093216485	------I-----GIDAY PEL DRLPKAAMPR-----G----
	Pseudomonas veronii	WP_046384253	------I--------AY PEL DKLPVKAVLR--Q--G----
	Pseudomonas versuta	WP_060691284	--V---I--------AY PQL DRLPVLVLGR-----K---E
	Pseudomonas viridiflava	WP_029243934	-D----I--------AY PEL S--PKNMQAR--S---E---
	Pseudomonas vranovensis	WP_028941417	------I-----G-DAY PQL E-AGKRLQR---R--GD---
	Pseudomonas wadenswilerensis	WP_115086165	------I--------AY PQL Q-SNPRLQK--RR--GD--S
	Pseudomonas weihenstephanensis	WP_048365213	--V---I-----G--AY PEL ERLPALVAAR-N---K----
	Pseudomonas yamanorum	WP_093200170	------I--------AY PEL DKLPPKAVSR-----G----
Other Bacteria	*Azotobacter beijerinckii*	WP_090618905	------I-----G--AY PDL VDFSGAE-EL----------
	Azotobacter chroococcum	WP_089169686	----V-I------D-AY PEL SALPAGTAKR----------
	Azotobacter salinestris	WP_152389543	------I-----G--AY PDL VELSKDGAKR--T-------
	Azotobacter vinelandii	WP_012700409	----VRI---------Y PDL SDLPDKA-KR--E-------

Figure-S48

Partial sequence alignments of a protein CHAD domain-containing protein showing a 3aa Del (highlighted), which is uniquely shared by all species from the Linyingensis clade. No homolog was found for the ingroup species *P. oryzae* and *P. oryzagri*.

Group	Species	Accession	109				141
**Linyingensis Clade (*Geopseudomonas* gen. nov.) (5/5)**	*Pseudomonas linyingensis*	WP_090309801	TVKPG		AVG		KQAPHINVSLFARGINIQLQTRIYF
	"Pseudomonas oryzae"	WP_090348518	-----		---		-----------------H-------
	Pseudomonas sagittaria	WP_092428246	-----		--		--------A-------------L--
	Pseudomonas oryzagri	WP_229605151	-----		---		-------------------------
	Pseudomonas aromaticivorans	WP_217679541	-----		---		-------------------------
**Other *Pseudomonas***	*Pseudomonas abietaniphila*	WP_074751232	-----	VVLN	-A-	V	PM---V-----------H----L--
	Pseudomonas aeruginosa	WP_003118582	-----	VVRN	-Q-	V	PM---------------H----L--
	Pseudomonas aestus	WP_022641145	-----	IVNN	TA-	L	PM------M--------H-H--L--
	Pseudomonas agarici	WP_017129860	-I---	VVNN	-A-	V	PM---------------H----L--
	Pseudomonas alcaliphila	WP_075749937	-----	VVHN	-A-	V	PM---V--A--------H-N--L--
	Pseudomonas alkylphenolica	WP_032495632	-----	VVNA	-D-	R	P------------------A-----
	Pseudomonas alkylphenolica	WP_038614920	-----	VVRN	-A-	V	PM---------------H----L--
	Pseudomonas amygdali	WP_004665140	-I---	SAHN	-A-	V	PM-A-------------H----L--
	Pseudomonas argentinensis	WP_074879973	-I---	VVNN	-A-	V	PM------A--------H-H--L--
	Pseudomonas asiatica	WP_015271805	-----	VVNN	-A-	V	PM-----I---------H-H--L--
	Pseudomonas asplenii	WP_026007535	-I---	VVNN	-A-	V	PM---------------H----L--
	Pseudomonas asturiensis	WP_073161750	-I---	TVRN	-A-	V	PM-S-------------H----L--
	Pseudomonas asuensis	WP_188865363	-I---	KVTD	SQ-	R	PM-----LT--------H-------
	Pseudomonas avellanae	WP_005617434	-----	SVRN	-A-	V	PM-A--S----------H----L--
	Pseudomonas batumici	WP_040068865	-I---	TLNN	-A-	V	PM-A-------------H----L--
	Pseudomonas benzenivorans	WP_090446804	-----	VVRN	-A-	V	PM---------------H----L--
	Pseudomonas brassicae	WP_163950993	-----	VVAN	-A-	V	PM-----I---------H-H--L--
	Pseudomonas bubulae	WP_094999387	-I---	VVNN	-A-	V	PM---------------H----L--
	Pseudomonas cannabina	WP_055001809	-I---	SVRN	-A-	V	PM-A-V-----------H----L--
	Pseudomonas capeferrum	WP_033702952	-----	VVNN	-A-	V	PM-----I---------H-H--L--
	Pseudomonas caricapapayae	WP_055009931	-I---	TVRN	-A-	V	PM-A-V-----------H----L--
	Pseudomonas caspiana	WP_087266210	-I---	VVRN	-A-	V	PM-A-------------H----L--
	Pseudomonas cerasi	WP_003366941	-I---	SARN	-A-	V	PM-A-------------H----L--
	Pseudomonas chlororaphis	WP_038635294	-I---	VVKN	-A-	V	PM------A--------H-H--L--
	Pseudomonas cichorii	WP_025260529	-I---	VVHN	-A-	V	PM-A-------------H----L--
	Pseudomonas citronellolis	WP_074983014	-I---	VTKA	-D-	R	P----------------H----L--
	Pseudomonas coleopterorum	WP_049860902	-I---	VVNN	-A-	V	PM-----I------V--H-H--L--
	Pseudomonas composti	WP_074941616	-----	VVNN	-A-	V	PM------A--------H-N--L--
	Pseudomonas congelans	WP_054993019	-I---	SARN	-A-	V	PM-A-------------H----L--
	Pseudomonas coronafaciens	WP_053932232	-I---	SACN	-A-	V	PM-A-------------H----V--
	Pseudomonas cremoricolorata	WP_038413715	-----	VVNN	-A-	V	PM-----L---------H----L--
	Pseudomonas cuatrocienegasensi	WP_069519246	-----	VVNN	-A-	V	PM---V-----------H-H--L--
	Pseudomonas daroniae	WP_131191159	-----	VVDN	-A-	V	PM---------------H-H-----
	Pseudomonas deceptionensis	WP_048359323	-I---	VVNN	-G-	V	PM---------------H----L--
	Pseudomonas delhiensis	WP_009618058	-I---	VTKA	-D-	R	P----------------H----L--
	Pseudomonas denitrificans	WP_003102189	-----	VVRN	-Q-	V	PM---------------H----L--
	Pseudomonas donghuensis	WP_010226894	-I---	VAKN	-A-	V	PM---------------H----L--
	Pseudomonas dryadis	WP_131177543	-----	VLHN	-A-	V	PM---------------H-H--L--
	Pseudomonas duriflava	WP_145136187	-----	KVLD	SL-	R	PM-----LA--------H-------
	Pseudomonas endophytica	WP_055101740	-I---	VVSN	-A-	V	PM------T--------H----L--
	Pseudomonas entomophila	WP_011535803	-----	VVNN	-A-	V	PM-----L---------H-H--L--
	Pseudomonas extremaustralis	WP_010565184	-----	TVKN	-A-	V	PM---------------H----V--
	Pseudomonas ficuserectae	WP_004665140	-I---	SAHN	-A-	V	PM-A-------------H----L--
	Pseudomonas flavescens	WP_084307148	-I---	VVDN	-A-	V	PM---V-----------H-H-----
	Pseudomonas floridensis	WP_083182039	-I---	SMRN	-A-	V	PM-S-------------H----L--
	Pseudomonas formosensis	WP_090538728	-----	VVKN	-A-	V	PM------A----------------
	Pseudomonas fragi	WP_016779341	-I---	VVNN	-A-	V	PM---------------H----L--
	Pseudomonas	WP_013791872	-----	VVNN	-A-	V	PM---V-IA--------H-H--L--
	Pseudomonas furukawaii	WP_003456008	-I---	VVKN	-A-	V	PM-----L---------H----V--
	Pseudomonas fuscovaginae	WP_026007535	-I---	VVNN	-A-	V	PM---------------H----L--
	Pseudomonas gingeri	WP_017123401	-I---	VVNN	-A-	V	PM---------------H----L--
	Pseudomonas guariconensis	WP_090345144	-----	VVNN	-A-	V	PM-----L---------H-H--L--
	Pseudomonas guguanensis	WP_090433141	-----	VVKN	-A-	V	PM---V--A--------H-N--L--
	Pseudomonas helleri	WP_048367476	-I---	VVKN	-A-	V	PM---------------H----L--
	Pseudomonas humi	WP_009618058	-I---	VTKA	-D-	R	P----------------H----L--
	Pseudomonas hunanensis	WP_003251601	-----	VVNN	-A-	V	PM-----I---------H-H--L--
	Pseudomonas hydrolytica	WP_017362044	-----	VVKR	PA-	V	PM------A--------H-N--L--
	Pseudomonas indica	WP_084337765	-----	VANN	-A-	V	PM------A--------H-------
	Pseudomonas inefficax	WP_133975437	-----	VVNN	-A-	V	PM-----I---------H-H--L--
	Pseudomonas japonica	WP_042124861	-----	VVNN	-A-	V	PM-----I---------H-------
	Pseudomonas jinjuensis	WP_084309633	-I---	VVNN	-A-	V	PM---------------H-------
	Pseudomonas juntendi	WP_009682256	-----	VVNN	-A-	V	PM-----I---------H-H--L--
	Pseudomonas knackmussii	WP_043251891	-I---	VVKN	-A-	Q	D----------------H----L--
	Pseudomonas laurentiana	WP_163932306	-I---	VALN	-A-	V	PM---------------H----L--
	Pseudomonas laurylsulfatovoran	WP_103392910	-----	LVKN	-S-	V	PM-A-------------H----L--
	Pseudomonas luteola	WP_074825087	-I---	KVMD	SQ-	R	PM-----LA--------H-------
	Pseudomonas marincola	WP_090511685	-I---	VVNN	-A-	V	AM-----L---------H----V--
	Pseudomonas massiliensis	WP_040261622	-I---	VVTT	TA-	I	PM---------------H-------
	Pseudomonas meliae	WP_044343773	-I---	SAHN	-A-	V	PM-A-------------H----L--
	Pseudomonas mohnii	WP_047530326	-----	VVKN	-A-	V	AM-S-------------H----L--
	Pseudomonas monteilii	WP_013974224	-----	AVNN	-A-	V	PM-----I---------H-H--L--
	Pseudomonas moorei	WP_090319973	-----	VVKN	-A-	V	AM-A-V-----------H----L--
	Pseudomonas mosselii	WP_096049297	-----	VVNN	-A-	V	PM-----I---------H-H--L--
	Pseudomonas multiresinivorans	WP_169937772	-I---	VVKN	-A-	V	PM------L-------LH----L--
	Pseudomonas nitritireducens	WP_037015695	-I---	VVKN	-A-	V	PM--------------LH----L--
	Pseudomonas nitroreducens	WP_084359034	-I---	VVKN	-A-	V	PM--------------LH----L--
	Pseudomonas nosocomialis	WP_138410308	-I---	VVKN	-A-	V	PM---V----------------L--
	Pseudomonas oryzihabitans	WP_059313397	-I---	QVAD	QR-	Q	PM------T--------H----L--
	Pseudomonas otitidis	WP_074972083	-I---	VVKN	-A-	V	PM---------------H----L--
	Pseudomonas ovata	WP_109514519	-----	VVTN	GA-	V	PM---------------H-H--L--
	Pseudomonas panacis	WP_057005844	-----	TVKN	-A-	V	PM---V-----------H----V--
	Pseudomonas panipatensis	WP_090265835	-----	VVKN	-A-	V	PM---------------H----L--
	Pseudomonas parafulva	WP_028633331	-----	VVNN	-A-	V	PM-----L---------H-H--L--
	Pseudomonas piscis	WP_022641145	-----	IVNN	TA-	L	PM------M--------H-H--L--
	Pseudomonas plecoglossicida	WP_013974224	-----	AVNN	-A-	V	PM-----I---------H-H--L--
	Pseudomonas protegens	WP_015637137	-----	VVNN	-A-	V	PM-----L---------H-H--L--
	Pseudomonas psychrophila	WP_048351205	-I---	VVNN	-G-	V	PM---------------H----L--
	Pseudomonas psychrotolerans	WP_074529186	-----	QVTD	QR-	Q	PM------T--------H-------
	Pseudomonas psychrotolerans	WP_074529186	-----	QVTD	QR-	Q	PM------T--------H-------
	Pseudomonas pudica	WP_108479966	-----	VVKN	-A-	V	PM-----I---------H-H--L--
	Pseudomonas punonensis	WP_073264907	-----	VVNN	-A-	V	PM---V--A--------H-H--L--

Group	Species	Accession					
Other *Pseudomonas*	*Pseudomonas putida*	WP_003251601	-----	VVNN	-A-	V	PM-----I---------H-H--L--
	Pseudomonas qingdaonensis	WP_043860697	-----	VVAN	-A-	V	PM---------------H-H--L--
	Pseudomonas reidholzensis	WP_119145918	-----	VVNN	-A-	V	PM-----I-----------H--L--
	Pseudomonas resinovorans	WP_016492979	-I---	VVKN	-A-	V	PM-----L---------H----L--
	Pseudomonas rhizoryzae	WP_058764260	-----	QVSD	QR-	Q	PM------T--------H-------
	Pseudomonas rhizosphaerae	WP_043192757	-I---	VVNN	-A-	V	PM-----I------V--H-H--L--
	Pseudomonas saponiphila	WP_092309774	-----	VVNN	-A-	V	PM-----L---------H-H--L--
	Pseudomonas savastanoi	WP_004665140	-I---	SAHN	-A-	V	PM-A-------------H----L--
	Pseudomonas saxonica	WP_146384384	-I---	VVNT	-G-	V	PM---------------H----L--
	Pseudomonas sediminis	WP_099526308	-----	VVKN	-A-	V	PM------A--------H-N--L--
	Pseudomonas segetis	WP_089358994	-I---	VVNN	-A-	V	PM---------------H----L--
	Pseudomonas shirazica	WP_015271805	-----	VVNN	-A-	V	PM-----I---------H-H--L--
	Pseudomonas sichuanensis	WP_110994677	-----	VVNN	-A-	V	PM-----I---------H-H--L--
	Pseudomonas sihuiensis	WP_092377874	-----	VVRN	-A-	V	PM---V--A--------H-N--L--
	Pseudomonas silesiensis	WP_064676961	-----	VVKN	-L-	L	PM-A-----V-------H----L--
	Pseudomonas soli	WP_094012490	-----	VVNN	-A-	V	PM-----L---------H-H--L--
	Pseudomonas straminea	WP_093501545	-----	VVNN	-A-	V	PM---V-IA--------H-H--L--
	Pseudomonas stutzeri	WP_014596062	-----	VVKS	RA-	I	PM---------------H-H-----
	Pseudomonas syringae	WP_025389439	-----	SVRN	-A-	V	PM-S-------------H----L--
	Pseudomonas taeanensis	WP_025164614	-----	VVKN	-A-	V	PM---V-I---------H----L--
	Pseudomonas taetrolens	WP_048378282	-----	VVKN	-A-	L	PM------T--------H----L--
	Pseudomonas taiwanensis	WP_023378693	-----	VVKN	-A-	V	PM-----I---------H-H--V--
	Pseudomonas thermotolerans	WP_017939982	-I---	VVKT	-A-	L	P----------------H-------
	Pseudomonas toyotomiensis	WP_074918582	-----	VVHN	-A-	V	PM---V--A--------H-N--L--
	Pseudomonas tremae	WP_024669822	-I---	SACN	-A-	V	PM-A-------------H----V--
	Pseudomonas umsongensis	WP_083348604	-----	RLKN	-S-	V	TM-A----A--------H----L--
	Pseudomonas veronii	WP_057005844	-----	TVKN	-A-	V	PM---V-----------H----V--
	Pseudomonas versuta	WP_060690629	-I---	VVNN	-A-	V	PM---------------H----L--
	Pseudomonas viridiflava	WP_029243668	-I---	SMRN	-A-	V	PM-S-------------H----L--
	Pseudomonas vranovensis	WP_028944010	-I---	VVCN	-A-	V	PM---------------H----L--
	Pseudomonas wadenswilerensis	WP_115088828	-I---	VANN	-A-	V	PM-----I---------H----L--
	Pseudomonas zeshuii	WP_010795479	-I---	KVMD	SQ-	R	PM-----LA--------H-------
Other Bacteria	*Azotobacter beijerinckii*	WP_090622042	-----	VVKN	-T-	K	PM----S---------------L--
	Azotobacter chroococcum	WP_089168859	-----	VVKN	-A-	K	PM----S------------------
	Azotobacter salinestris	WP_152389024	-----	MVKN	-A-	K	PM-----------------------
	Azotobacter vinelandii	WP_012702342	-----	VVKN	-A-	K	PM----S----------H-------

Figure-S49

Partial sequence alignments of the protein Protocatechuate 3,4-dioxygenase subunit alpha showing 4aa and 1aa Deletions (highlighted), which are uniquely shared by all species from the Linyingensis clade. No homolog was found for the ingroup species *P. guangdongensis.*

Group	Species	Accession	194		231
**Linyingensis Clade (*Geopseudomonas* gen. nov.) (5/5)**	*Pseudomonas linyingensis*	WP_090310373	GDSVQLSIRSQQD		RLGNRSGVIDTQSTDTRISGRLGEW
	Pseudomonas sagittaria	WP_092434201	-------------		----HA------G----V-------
	"Pseudomonas oryzae"	WP_090352097	-EE-----Y----		--ASPR-A-A--R----V-------
	Pseudomonas aromaticivorans	WP_217679058	-EM----------		--DS--------D----VN------
	Pseudomonas oryzagri	WP_264758846	-EH---N-Y----		--AG---A-A--H---QV-----D-
Other *Pseudomonas*	*Pseudomonas kuykendallii*	WP_090230943	--T-H---S-NR-	R	LSQRQP-A--I--------------
	Pseudomonas resinovorans	WP_016492428	-EL-HV--S-NN-	R	LNNS-P----V------V-------
	Pseudomonas saudiphocaensis	WP_037021974	--R--I--S----	R	MSSS-AD-V-I-E----V-------
	Pseudomonas pseudoalcaligenes	WP_003460463	-EL-HVA-S--R-	R	VSNS-P----V------V-------
	Pseudomonas sediminis	WP_099523917	-EL-HVA-S--R-	R	VSNS-P----V------V-------
	Pseudomonas indoloxydans	WP_108234110	-EL-HVA-S--R-	R	VSNS-P----V------V-------
	Pseudomonas toyotomiensis	WP_074914868	-EL-HVA-S--R-	R	VSNS-P----V------V-------
	Pseudomonas composti	WP_074937691	-EL-HV--N--R-	R	INDH-P----V------V-------
	Pseudomonas hydrolytica	WP_041979346	-EL-HV--S--R-	R	MNDH-P--V-V------V-------
	Pseudomonas khazarica	WP_134675881	-EL-HVT-S--R-	R	VSS---N---V------V-------
	Pseudomonas oleovorans	WP_037055885	-EL-HVT-S--R-	R	VSS---N---V------V-------
	Pseudomonas alcaligenes	WP_061904550	--R-HV--S-NN-	R	VNQYQP-----------V-------
	Pseudomonas sihuiensis	WP_017677083	-EL-HVA-S--R-	R	VSNSHP----V------V-------
	Pseudomonas chengduensis	WP_017677083	-EL-HVA-S--R-	R	VSNSHP----V------V-------
	Pseudomonas taeanensis	WP_025166341	--I-HI--S-NR-	R	LNPSQP----V------V-------
	Pseudomonas alcaliphila	WP_064494735	-EL-HVA-S--R-	R	VSNSHP----V------V-------
	Pseudomonas indica	WP_084335255	--I-HV--S-NR-	R	LSQ-MP-A--I------V-------
	Pseudomonas guguanensis	WP_090429005	-EL-HVA-S--R-	R	VNS--PD---V------V-------
	Pseudomonas hydrolytica	WP_129483593	-EL-HVA-S--R-	R	VNS--PD---V------V-------
	Pseudomonas mendocina	WP_003240668	-EL-HVA-S--R-	R	VNS--PD---V------V-------
	Pseudomonas benzenivorans	WP_090438833	-EI-HI--S-NR-	R	LSQSQP----V------V-------
	Pseudomonas stutzeri	WP_013982949	--R--I--S-SR-	R	LAQG----VEV-NA---V---V---
	Pseudomonas songnenensis	WP_126188742	--R--I--S-TR-	R	LAQG----VEV-NA---V---V---
	Pseudomonas pohangensis	WP_090193564	--R-N-----NN-	S	LSRSQP---NV------VN------
	Pseudomonas aeruginosa	WP_003108752	-NL-HVT-S-SN-	R	LSQSHP-----------V--K----
	Pseudomonas leptonychotis	WP_136664625	-ER-HI--S-NN-	R	LSQSQP-A--V------V-------
	Pseudomonas borbori	WP_090504569	-EI-HIN-S-NR-	R	LSSSQP----I------V-------
	Pseudomonas caeni	WP_022966790	--I---EVN--N-	R	-SQQYGN-------SS-V-------
	Pseudomonas denitrificans	WP_023115286	-NL-HVT-S-SN-	R	LSQSHP------N----V--K----
Other *Bacteria*	*Azomonas agilis*	WP_144570590	--W--INLST-R-	R	VNH--PE-L-L---E--V-------
	Azomonas macrocytogenes	WP_183164656	--A--VTLS--N-	R	VNR-HRN-L-L-N-E--V--K----

Figure- S50

Partial sequence alignments of a protein Secretin showing a 1aa Del (highlighted), which is uniquely shared by all species from the Linyingensis clade. No homolog of this protein was found for the deeper branching *P. guangdongensis*.

Group	Species	Accession	236		276
**Linyingensis Clade (*Geopseudomonas* gen. nov.) (5/5)**	*Pseudomonas linyingensis*	WP_090312664	YRLPVWEEWQRRPNLFLHPVVSD	PQP	DDGWSGRCGLLHQAV
	"Pseudomonas oryzae"	WP_090350417	------Q--RQL------QIA--	A--	---------------
	Pseudomonas sagittaria	WP_092431542	-----------L-----------	---	---------------
	Pseudomonas aromaticivorans	WP_217679723	---------RQ-------Q----	---	---------------
	Pseudomonas oryzagri	WP_229604902	----A-A---H-------Q----	---	-E-------------
**Other *Pseudomonas***	*Pseudomonas abietaniphila*	WP_074754541	-E-KH-D--KTL---H--K----		LC--E----M--E--
	Pseudomonas abyssi	WP_096004495	-NA-H-QR-EQA---Y--R----		QAD----Q-------
	Pseudomonas aeruginosa	WP_003096331	----H-T--EGL------R----		LC--E-------E--
	Pseudomonas aestus	WP_022641762	-Q-EH-DQ--QL---Y--K----		LC--E----M--E--
	Pseudomonas aestusnigri	WP_088276205	-EA-H-QR--QA---Y--R-I--		QPD-PE-Q-M-----
	Pseudomonas agarici	WP_060782043	-E-ER----KTL------K----		PC--D----M--E--
	Pseudomonas alcaligenes	WP_061903021	-Q--H-QD-EQQAG-Q--R---E		A-A-G-------E--
	Pseudomonas alcaliphila	WP_075744862	-E--H-A---QLD--H--Q----		QC--Q-------E--
	Pseudomonas alkylphenolica	WP_038615728	-QIEH-A--EQL------K----		LC--E-------E-I
	Pseudomonas amygdali	WP_004655176	-QVSH-D--TAL------K----		LC--E-------E--
	Pseudomonas anguilliseptica	WP_090378005	-E-EH-AQ--QV---L--Q----		QC--Q-------E--
	Pseudomonas antarctica	WP_064454654	-EIEH-AQ--QL------K----		LC--E-------E--
	Pseudomonas argentinensis	WP_074881930	-QV-H-D---AL------Q----		QC--Q-------E--
	Pseudomonas arsenicoxydans	WP_090178254	-AIEH-D--LKL------K----		QC--Q----M--E--
	Pseudomonas asiatica	WP_013974732	-QIEH-D----L------Q----		LC--E----M--E--
	Pseudomonas asplenii	WP_090204048	-E-EH-D--KKL------K---E		LC--E----M--E--
	Pseudomonas asturiensis	WP_073170838	-AISH-D---KL------K----		LC--E----M--E--
	Pseudomonas atacamensis	WP_122705605	-QIEH-D--LKL------K----		QC--E----M--E--
	Pseudomonas avellanae	WP_005621392	-QVSH-D--ATL------K----		LY--E-------E--
	Pseudomonas azotifigens	WP_028241473	---EH-AD--AQS-----Q----		VC--D-------E--
	Pseudomonas azotoformans	WP_033897532	-EIEH-DQ-LQL------K----		LC--E-------E--
	Pseudomonas baetica	WP_100846874	-DIEH-D--LKL------K----		QC--E----M--E--
	Pseudomonas balearica	WP_043218281	-Q-RH-D--R-M---H--R----		VCD-G-------E-I
	Pseudomonas batumici	WP_040071616	-T-DH-D--AQL------K----		LC--E-------E--
	Pseudomonas benzenivorans	WP_090443942	-Q--S-A---SL---L--Q----		QCA-P-------E--
	Pseudomonas bohemica	WP_110948769	-E-KH-D--KTL---Y--KI---		LC--E----M--EV-
	Pseudomonas borbori	WP_090500042	-E--C-A---SL------R----		QC--Q-------E--
	Pseudomonas brassicacearum	WP_025216058	-EIEH-D--KKL------K----		VC--E----M--E--
	Pseudomonas brassicae	WP_163942152	-T-EH-D--AQL------K----		LC--E-------E--
	Pseudomonas brenneri	WP_090292378	-QVEH--Q--QL------K----		LC--E-------E--
	Pseudomonas bubulae	WP_095000702	-T-DE-A--EKT------K----		LC--E----M--E--
	Pseudomonas canadensis	WP_028618404	-QIEH-DQ-LQL------K----		LC--E-------E--
	Pseudomonas cannabina	WP_055001765	-QVSH----TKL------K----		LC--E-------E--
	Pseudomonas capeferrum	WP_033702191	-QIEH-DQ--HL------K----		LC--E----M--E--
	Pseudomonas caricapapayae	WP_055008954	-QVSH-D--ATL------K----		LC--E-------E--
	Pseudomonas carnis	WP_034129017	-EIEH-DQ-LQL------K----		LC--E-------E--
	Pseudomonas caspiana	WP_087271924	-EIKH----KKL------K----		LC--E----M--E--
	Pseudomonas cedrina	WP_076951972	-QIEH-DQ-LQL------K----		VC--E-------E--
	Pseudomonas cerasi	WP_065350860	-QVSH-D--AKL------K----		LC--E-------E--
	Pseudomonas chengduensis	WP_017678347	-E--H-A---QLD--H--Q----		QC--Q-------E--
	Pseudomonas chloritidismutans	WP_031298059	-Q-SH-D---GMS--H--R----		VC--E----M--E-I
	Pseudomonas chlororaphis	WP_038635977	-QIEH-D--KKL------K----		LC--E----M--E--
	Pseudomonas cichorii	WP_025257999	-EIRQ-D--ATL------K----		LC--E----M--E-
	Pseudomonas citronellolis	WP_024128814	-A--H-D--KGLA--H--K----		LC--Q-------E--
	Pseudomonas coleopterorum	WP_090361240	-A-EH--Q-KQL-----NK----		LC--E-------E--
	Pseudomonas composti	WP_074941425	-A--H-AD--QLH--R--Q----		QC--Q-------E--
	Pseudomonas congelans	WP_054994425	-QVSH-D--AKL------K----		LC--E-------E--
	Pseudomonas coronafaciens	WP_024669146	-QVSH-D--AKL------M----		VC--E-------E--
	Pseudomonas corrugata	WP_069018356	-EIEH----KKL------K----		LC--E----M--E--
	Pseudomonas costantinii	WP_071485120	-EIEH--Q-LQL------K----		LC--E-------E--
	Pseudomonas cremoricolorata	WP_038411377	-QIEH-D--AQ-------K----		LC--D-------E--
	Pseudomonas cremoris	WP_185709803	-DIEH-DQ--QL------K----		LC--E-------E--
	Pseudomonas cuatrocienegasensi	WP_069516443	-A--H-AD--QVA--T--Q---E		SC--Q-------E--
	Pseudomonas daroniae	WP_131191401	-S--H-Q---AL------Q----		QC--K-------E-I
	Pseudomonas deceptionensis	WP_048360572	-T-EE-A--EKI------K----		LC--E----M--E--
	Pseudomonas delhiensis	WP_089391028	-A--H-D--KGLA--H--K----		LC--Q-------E--
	Pseudomonas denitrificans	WP_003096331	----H-T--EGL------R----		LC--E-------E--
	Pseudomonas donghuensis	WP_010221161	-QIEH-A---HL------K----		LC--E-------E--
	Pseudomonas dryadis	WP_131174350	-A--H-D--RQL------Q----		QC--Q-------E-I
	Pseudomonas duriflava	WP_145137699	-A--H-A--EQD-R-H--K----		VC--E-------E--
	Pseudomonas edaphica	WP_056860765	-EIEH--Q--QL------K----		LC--E-------E--
	Pseudomonas endophytica	WP_055103087	-T-EE-DA--QI------K----		VC--E-------E--
	Pseudomonas entomophila	WP_011536306	-EIEH-D----L------K----		LC--E-------E--
	Pseudomonas extremaustralis	WP_010564502	-EIQE-DQ-LKL------K----		LC--D-------E--
	Pseudomonas extremorientalis	WP_071490462	-EIEH-DQ-LQL------K----		LC--E-------E--
	Pseudomonas ficuserectae	WP_054995567	-QVSH-D--TAL------K----		LC--E-------E--
	Pseudomonas fildesensis	WP_048729322	-EIEH-DQ-LQL------K----		LC--E-------E--
	Pseudomonas flavescens	WP_084304569	----H-----AL------Q----		QC--A-------E-I
	Pseudomonas flexibilis	WP_039560096	-A--Q-D----T---I--R----		LC--E-------E--
	Pseudomonas floridensis	WP_083181806	-EIAH-D--AKL-----NK----		LC--E----M--E--
	Pseudomonas fluorescens	WP_053258590	-EIEH-DQ--QL------K----		LC--E-------E--
	Pseudomonas fragi	WP_016783022	-T-DE-A--EKT------K----		LC--E----M--E--
	Pseudomonas frederiksbergensis	WP_071552856	-QIEH-D--LKL------K----		LC--E----M--E--
	Pseudomonas fulva	WP_013789611	-Q--H-D---AL---S--Q----		QC--Q-------E--
	Pseudomonas furukawaii	WP_003457520	-E-AH-D--K-LE--V--Q----		AC--K-------E--
	Pseudomonas fuscovaginae	WP_019360268	-E-EH-D--KKL------K---E		LC--E----M--E--
	Pseudomonas gallaeciensis	WP_096004495	-NA-H-QR-EQA---Y--R----		QAD----Q-------
	Pseudomonas gessardii	WP_076963618	-QVEH--Q--QL------K----		LC--E-------E--
	Pseudomonas gingeri	WP_017125451	-E-EH-D--KTL------K----		LC--E----M--E--
	Pseudomonas graminis	WP_074892575	-Q-KH-D---TL------K----		LC--E----M--E--
	Pseudomonas granadensis	WP_090287361	-QIEH-D--LKL------K----		QC--E----M--E--
	Pseudomonas grimontii	WP_090401554	-EIEH-DQ-LQL------K----		LC--E-------E--
	Pseudomonas guariconensis	WP_043212136	-QIEH-S--EQL------K----		LC--E-------E--
	Pseudomonas guguanensis	WP_090434652	-E--H-AQ--QLD--H--Q----		QC--Q-------E--

Other *Pseudomonas*	*Pseudomonas guineae*	WP_090241431	-EVGH-AQ-LQV------Q----	QC--Q-------E--
	Pseudomonas haemolytica	WP_153871316	-AIDH-DQ-LQL---Y--K----	LC--E-------E--
	Pseudomonas helleri	WP_048366888	-A-EE-DA--QI------K----	LC--E-------E--
	Pseudomonas helmanticensis	WP_134176000	-EIEH-D--LKL------K----	QC--E----M--E--
	Pseudomonas humi	WP_024128814	-A--H-D--KGLA--H--K----	LC--Q-------E--
	Pseudomonas hunanensis	WP_003253665	-QIEH-D----L------Q----	LC--E----M--E--
	Pseudomonas hydrolytica	WP_041976110	-E--H-A---QLD--H--Q----	QC--Q-------E--
	Pseudomonas hydrolytica	WP_129481997	-A--H-TD---LG--H--Q----	QR--P-------E--
	Pseudomonas indica	WP_084333132	-Q-SH-D--RSL---H--K----	AC--D----M--E--
	Pseudomonas indoloxydans	WP_108233338	-E--H-A---QLD--H--Q----	QC--Q-------E--
	Pseudomonas inefficax	WP_133976243	-QIEH-D----L------Q----	LC--E----M--E--
	Pseudomonas japonica	WP_042121075	-EIEE-AQ---L------K----	LC--E-------E--
	Pseudomonas jessenii	WP_090452076	-QIEH-D--LKL------K----	QC--E----M--E--
	Pseudomonas jilinensis	WP_080049253	-QA-Y--Q-RAH---Y-EQ----	QPD-P--Q-M-----
	Pseudomonas jinjuensis	WP_084313852	-E--H-QQ--GV---H--Q----	PCD-P--H----E--
	Pseudomonas juntendi	WP_009684149	-QIEH-D----L---Y--Q----	LC--E----M--E--
	Pseudomonas kairouanensis	WP_135290719	-DIEH-DQ--QL------K----	LC--E-------E--
	Pseudomonas khazarica	WP_134677854	-Q--H-A---QLA--H--Q----	QC--Q-------E--
	Pseudomonas kilonensis	WP_053190057	-EIEH-D--KKL------K----	LC--E----M--E--
	Pseudomonas kirkiae	WP_131182698	-Q--H-N--RQV-G-V--Q---E	LC----------E--
	Pseudomonas knackmussii	WP_043248469	-A--H-D--KSL---H--K----	PVE-D--R----E--
	Pseudomonas koreensis	WP_041476904	-QIEH-D--LKL------K----	QC--E----M--E--
	Pseudomonas kribbensis	WP_085708962	-EIEH-D--LKL------K----	QC--E----M--E--
	Pseudomonas kunmingensis	WP_090522018	-Q-SH-D---GMS--H--R----	VC--D----M--E-I
	Pseudomonas kuykendallii	WP_090224116	-HV-H-Q---TVD-----Q----	LC--E-------E-I
	Pseudomonas lactis	WP_003195327	-QIEH-DQ-LQL------K----	LC--E-------EV-
	Pseudomonas laurentiana	WP_163938335	-S-EH-D---QL---Y--K----	LC--E-------E--
	Pseudomonas laurylsulfatiphila	WP_104450229	-QIEH-N--LKL------K----	QC--E----M--E--
	Pseudomonas laurylsulfatovoran	WP_103395878	-QVEH-G--LKL------K----	QC--E----M--E--
	Pseudomonas leptonychotis	WP_136663512	-A-EH-AK--QV------Q----	QC--Q-------E--
	Pseudomonas libanensis	WP_057011367	-QIEH--Q--QL------K----	LC--E-------E--
	Pseudomonas lini	WP_048396295	-GIEH-D--LKL---Y--K----	QC--G----M--E--
	Pseudomonas lundensis	WP_047281889	-T-EE-DA--SI---H--K----	LC--E-------E--
	Pseudomonas lurida	WP_034110159	-EIEH-DQ-LQL------K----	LC--E-------E--
	Pseudomonas lutea	WP_037016522	-Q-KH-D---TL------KI---	LC--E----M--E--
	Pseudomonas luteola	WP_074821551	-A--H----EQD-R-H-NK----	LC--E-------E--
	Pseudomonas marginalis	WP_064054384	-QIEH-DQ-LQL------K----	LC--E-------E--
	Pseudomonas marincola	WP_090510057	-A-D--NS--QLD--T--Q----	QC--K-------E--
	Pseudomonas massiliensis	WP_040260249	--IEH-D----L---Y--R----	LC--E-------E--
	Pseudomonas matsuisoli	WP_188984358	-TIER-A--ESLA--H--K----	LC--E-------E--
	Pseudomonas mediterranea	WP_047702521	-EIEH-D--KKL------K----	LC--E----M--E--
	Pseudomonas meliae	WP_044343821	-QVSH-D--TAL------K----	LC--E-------E--
	Pseudomonas mendocina	WP_011920593	-A--H-TD---LG--H--Q----	QR--P-------E--
	Pseudomonas migulae	WP_084320294	-EIEH-D--LKL------K----	QC--P----M--E--
	Pseudomonas mohnii	WP_047534833	-QIEH-D--LKL------K----	QC--Q----M--E--
	Pseudomonas monteilii	WP_013974732	-QIEH-D----L------Q----	LC--E----M--E--
	Pseudomonas moorei	WP_090326441	-QIEH-D--LKL------K----	QC--Q----M--E--
	Pseudomonas mosselii	WP_084942101	-EIEH-D--L-L------K----	LC--E-------E--
	Pseudomonas mucidolens	WP_084378674	--IEH-QQ--QL------K----	LC--E-------E--
	Pseudomonas nabeulensis	WP_135310803	-DIEH-DQ--QL------K----	LC--E-------E--
	Pseudomonas nitritireducens	WP_170858044	-E--H--Q-KSV---H--KI---	LC--Q-------E--
	Pseudomonas nitrititolerans	WP_125863243	-Q--H-D---GMA--H--R----	VC--E----M--E--
	Pseudomonas nitroreducens	WP_084358387	-E--H--Q-ESV---H--KI---	LC--Q-------E--
	Pseudomonas nosocomialis	WP_138408057	--IAS--Q-LQT---T--Q----	VC--E-------E-I
	Pseudomonas oceani	WP_104738043	-EA-H-QS--QAS--Y--R----	QPD-PE-Q-V-----
	Pseudomonas oleovorans	WP_134677854	-Q--H-A---QLA--H--Q----	QC--Q-------E--
	Pseudomonas orientalis	WP_057723380	-EIEH-DQ-RQL------K----	LC--E-------E--
	Pseudomonas ovata	WP_109512188	-AIDH-DQ--QL---Y--K----	LCA-E-------E--
	Pseudomonas palleroniana	WP_060753578	-EIEH-PQ--QL------K----	LC--E-------E--
	Pseudomonas panacis	WP_057003993	-EIEH-DQ--QL------K----	LC--E-------E--
	Pseudomonas panipatensis	WP_090264314	-E--H-S--KTL---Y--K----	LC--D-------E--
	Pseudomonas parafulva	WP_028632946	-QIEH-D--LQL---Y--Q----	LC--E----M-----
	Pseudomonas paralactis	WP_057702030	-QIEH-DQ-LQL------K----	LC--E-------EV-
	Pseudomonas peli	WP_090254682	-E-EH-AQ--QV------Q----	QC--Q-------E--
	Pseudomonas piscis	WP_022641762	-Q-EH-DQ--QL---Y--K----	LC--E----M--E--
	Pseudomonas plecoglossicida	WP_013974732	-QIEH-D----L------Q----	LC--E----M--E--
	Pseudomonas poae	WP_003234827	-EIEH-DQ-LQL------K----	LC--E-------E--
	Pseudomonas pohangensis	WP_090197446	-TV-H-D---QMS--H--Q----	A-D-A--K----E--
	Pseudomonas profundi	WP_150301764	-QA-Y-QQ--AE--IY--K----	-P-----Q---PE--
	Pseudomonas prosekii	WP_092276442	-EIEH-D--LKL------K----	QC--E----M--A--
	Pseudomonas protegens	WP_011064154	-Q-EH-D---QL---Y--K----	LC--E----M--E--
	Pseudomonas proteolytica	WP_029289554	-QVEH--Q--QL------K----	LC--E-------E--
	Pseudomonas pseudoalcaligenes	WP_039964254	-E--H-A---QLD--H--Q----	QC--Q-------E--
	Pseudomonas psychrophila	WP_019824470	-T-EE--Q-EQI------K----	LC--E----M--E--
	Pseudomonas pudica	WP_013974732	-QIEH-D----L------Q----	LC--E----M--E--
	Pseudomonas punonensis	WP_073262718	-A--H-D---AL------Q----	QC--Q-------E--
	Pseudomonas putida	WP_016502173	-QIEH-D----L------Q----	LC--E----M--E--
	Pseudomonas qingdaonensis	WP_043863393	-QIEH-D--ATL------K----	LC--E-------E--
	Pseudomonas reactans	WP_177002552	-EIEH-DQ-LQL------K----	LC--E-------E--
	Pseudomonas reidholzensis	WP_119146921	-AIEH-D----L------K----	LC--E----M--E--
	Pseudomonas reinekei	WP_075946423	-EVEH-D--LKL------K----	QC--P----M--E--
	Pseudomonas resinovorans	WP_016495308	-E-AH-D--R-L------Q----	QC--Q-------E--
	Pseudomonas rhizosphaerae	WP_043192258	-E-EH--Q-KLL-----NK----	LC--E----M--E--
	Pseudomonas rhodesiae	WP_034139746	-EIEH-TQ--QL------K----	LC--E-------E--
	Pseudomonas sabulinigri	WP_092286303	-QA-H-QR--AA---Y--K----	EAQ----D-------
	Pseudomonas salegens	WP_092383374	-QA-Y----ADM----M-K----	-A--A--Q---PA--
	Pseudomonas salomonii	WP_065931850	-EIEH--Q--QL------K----	LC--E-------E--
	Pseudomonas saponiphila	WP_092317426	-Q-EH-D---QL---Y--K----	LC--E----M--E--
	Pseudomonas saudimassiliensis	WP_044498994	-QA-H--H--QQA-IY--Q----	APD----Q-M-PE--
	Pseudomonas saudiphocaensis	WP_037025448	-Q--H-D--LDM---Y--R----	IC--E----M-----

Other *Pseudomonas*	*Pseudomonas savastanoi*	WP_004655176	-QVSH-D--TAL------K----	LC--E-------E--
	Pseudomonas saxonica	WP_122785338	-G-QE--A--NT------K----	LC--E-------E--
	Pseudomonas sediminis	WP_099524626	-Q--H-A---QLD--H--Q----	QC--Q-------E--
	Pseudomonas segetis	WP_089360373	-D--H-AD--KLE--T--K----	QC--Q-------E--
	Pseudomonas seleniipraecipitan	WP_092366945	-S--H-----AL------Q----	QC--K-------E-I
	Pseudomonas shirazica	WP_013974732	-QIEH-D----L------Q----	LC--E----M--E--
	Pseudomonas sichuanensis	WP_110995704	-QIEH-A--E-L------K----	LC--E-------E--
	Pseudomonas sihuiensis	WP_017678347	-E--H-A---QLD--H--Q----	QC--Q-------E--
	Pseudomonas silesiensis	WP_064680365	-AIEH-D--LKL------K----	QC--Q----M--E--
	Pseudomonas simiae	WP_010207400	-QIEH-DQ-LQL------K----	LC--E-------E--
	Pseudomonas sivasensis	WP_122681938	-QIEH-DQ-LQL------K----	LC--E-------E--
	Pseudomonas soli	WP_038707150	-QIEH-A--E-L------K----	LC--E-------E--
	Pseudomonas songnenensis	WP_126189124	-Q--H-----GMS--H--R----	VC--E----M--E-I
	Pseudomonas straminea	WP_093500232	-Q--H-D---AL---S--Q----	QC--Q-------E--
	Pseudomonas stutzeri	WP_014595697	----H-----GMS--H--R----	VC--E----M--E-I
	Pseudomonas synxantha	WP_057022755	-QIEH--Q--QL------K----	LC--E-------E--
	Pseudomonas syringae	WP_025390902	-QVSH-D--AKL------K----	VC--E-------E--
	Pseudomonas taeanensis	WP_025164007	-Q--N-S---QLT--S--R---E	ACV-E-------E--
	Pseudomonas taetrolens	WP_048383810	-T-EE-DA--QI------K----	LC--E-------E--
	Pseudomonas taiwanensis	WP_023382976	-AIEH-D--E-L---Y--K----	LCD-E----M--E--
	Pseudomonas thivervalensis	WP_053126434	-EIEH-D--KKL------K----	LC--E----M--E--
	Pseudomonas tolaasii	WP_016973079	-EIEH-DQ--QL---Y--K----	LC--E-------E--
	Pseudomonas toyotomiensis	WP_074913556	-E--H-A---QLD--H--Q----	QC--Q-------E--
	Pseudomonas tremae	WP_005888035	-QVSH-D--AKL------M----	VC--E-------E--
	Pseudomonas trivialis	WP_049711004	-DIEH-DQ-LQL------K----	LC--E-------E--
	Pseudomonas tuomuerensis	WP_027590982	-A--Q-D----T---I--R----	LC--E-------E--
	Pseudomonas umsongensis	WP_020797261	-EVEH-D--LKL------K----	QC--Q----M--E--
	Pseudomonas vancouverensis	WP_093228787	-QIEH-DQ-LKL------K----	QC--E----M--E--
	Pseudomonas veronii	WP_017845123	-EIEH-DQ-LQL------K----	LC--E-------E--
	Pseudomonas versuta	WP_060696230	-N-EE-DA--KI------K----	LC--E-------E--
	Pseudomonas viridiflava	WP_088234319	-DISH-D--AKL------K----	LC--E----M--E--
	Pseudomonas vranovensis	WP_028942378	-EIEH-D--E-L------K----	LC--E----M--E--
	Pseudomonas wadenswilerensis	WP_115084775	-QIEH-A--EHL------K----	LC--E----------
	Pseudomonas weihenstephanensis	WP_048364700	-T-EE-DA--KI---Y--K----	LC--E-------E--
	Pseudomonas xinjiangensis	WP_093396732	-QA-F-NS-ANQ---Y--Q----	-AA-P--Q---P---
	Pseudomonas yamanorum	WP_003213981	-DIEH-DQ-LQL------K----	LC--E-------E--
	Pseudomonas zeshuii	WP_010796697	-A--H----EQD-R-H-NK----	LC--E-------E--
	Pseudomonas zhaodongensis	WP_128120274	-Q-TH-Q---QM------QI---	ACE-A--R-M--E--
Other Bacteria	*Azotobacter beijerinckii*	WP_090621619	----YG----HL---H--R----	LC--E-------E--
	Azotobacter chroococcum	WP_089169452	----YA-----L---H--R----	LC--E-------E--
	Azotobacter vinelandii	WP_012703206	----CE---RHL---H--R----	LC--E-------E--

Figure- S51

Partial sequence alignments of a protein CDP-6-deoxy-delta-3,4-glucoseen reductase showing a 3aa Ins (highlighted), which is uniquely shared by all species from the Linyingensis clade. No homolog was found in the deeper-branching ingroup species *P. guangdongensis*.

Group	Species	Accession	9		45
**Linyingensis Clade** (***Geopseudomonas*** **gen. nov.) (5/6)**	*Pseudomonas linyingensis*	WP_090306967	ADPERTETWIKF	T	GNDLCHSCRATCCSLPVEVRLKDL
	Pseudomonas sagittaria	WP_092430090	------------	-	------T-----------------
	"Pseudomonas oryzae"	WP_090349655	-----L---L--	-	--PM-LD-S---------------
	Pseudomonas guangdongensis	WP_090211831	-----L-----Y		RSG--RD-Q----G------IA--
	Pseudomonas aromaticivorans	WP_217681960	-----L------	P	--S--RD-------------I---
	Pseudomonas oryzagri	WP_229606199	---D-L---LR-	-	--PM-LD-N------------P--
**Other** ***Pseudomonas***	*Pseudomonas balearica*	WP_041108652	-----L---VRY		S-G--RD-Q----T------ID--
	Pseudomonas nitrititolerans	WP_014854434	---D-L---ARY		S-G--RD------T------ID--
	Pseudomonas saudiphocaensis	WP_037025228	---D-L-S-ARY		RSG---D------T------ID--
	Pseudomonas kunmingensis	WP_031298455	-----L---VRY		SAG--RD-H----T------ID--
	Pseudomonas chloritidismutans	WP_031298455	-----L---VRY		SAG--RD-H----T------ID--
	Pseudomonas stutzeri	WP_014595577	-----L---VRY		SAG--RD-H----T------ID--
	Pseudomonas songnenensis	WP_003280999	-----L---VRY		SAG--RD-H----T------ID--
	Pseudomonas sediminis	WP_099522825	-EID-L---A-Y		TA-M----MS---TM------N--
	Pseudomonas composti	WP_037004502	-EID-L---A-Y		TA-M----MS---TM------N--
	Pseudomonas toyotomiensis	WP_021487773	-EID-L---A-Y		TA-M----MS---TM------N--
	Pseudomonas sihuiensis	WP_021487773	-EID-L---A-Y		TA-M----MS---TM------N--
	Pseudomonas indoloxydans	WP_021487773	-EID-L---A-Y		TA-M----MS---TM------N--
	Pseudomonas chengduensis	WP_021487773	-EID-L---A-Y		TA-M----MS---TM------N--
	Pseudomonas alcaliphila	WP_021487773	-EID-L---A-Y		TA-M----MS---TM------N--
	Pseudomonas pseudoalcaligenes	WP_003458937	-EID-L---A-Y		TA-M----MS---TM------N--
	Pseudomonas xanthomarina	WP_019340514	---D-L---ARY		S-G--RD-Q----T------FD--
	Pseudomonas hydrolytica	WP_013713689	-EID-L---A-Y		TA-M----MSS--T-------N--
	Pseudomonas leptonychotis	WP_136664182	-ELD-LD--A-Y		TA-M----HSS--T-------N--
	Pseudomonas mendocina	WP_011920733	-EID-L---A-Y		TA-M----ISS--T-------N--
	Pseudomonas hydrolytica	WP_011920733	-EID-L---A-Y		TA-M----ISS--T-------N--
	Pseudomonas guguanensis	WP_090430215	-EID-L---A-Y		TA-M----ISS--T-------N--
	Pseudomonas peli	WP_090254835	-ELD-L---A-Y		TA-M----MSS--T----A--N--
	Pseudomonas putida	WP_003249225	-EVD-L---QRY		TSNM--G-HS---T-----KI---
	Pseudomonas pudica	WP_046613485	-EVD-L---QRY		TSNM--G-HS---T-----KI---
	Pseudomonas juntendi	WP_003249225	-EVD-L---QRY		TSNM--G-HS---T-----KI---
	Pseudomonas hunanensis	WP_060538663	-EVD-L---QRY		TSNM--G-HS---T-----KI---
	Pseudomonas asiatica	WP_046613485	-EVD-L---QRY		TSNM--G-HS---T-----KI---
	Pseudomonas shirazica	WP_003259873	-EVD-L---QRY		TSNM--G-HS---T-----KI---
	Pseudomonas plecoglossicida	WP_003259873	-EVD-L---QRY		TSNM--G-HS---T-----KI---
	Pseudomonas monteilii	WP_003259873	-EVD-L---QRY		TSNM--G-HS---T-----KI---
	Pseudomonas inefficax	WP_003259873	-EVD-L---QRY		TSNM--G-HS---T-----KI---
	Pseudomonas guineae	WP_090244227	-ELD-LD--A-Y		TA-M----MSS--T-------G--
	Pseudomonas marincola	WP_069898417	-EID-LD--A-Y		TS-M----NSS--T------IS--
	Pseudomonas segetis	WP_089359885	-EID-LD--A--		TR-M----NSS--Q------IS--
	Pseudomonas jinjuensis	WP_084313326	--V--L---A-Y		TSG--RD-H----T------IG--
	Pseudomonas oleovorans	WP_037049937	-EID-L---A-Y		SA-M----ISS--T-------N--
	Pseudomonas khazarica	WP_037049937	-EID-L---A-Y		SA-M----ISS--T-------N--
	Pseudomonas cuatrocienegasensi	WP_069516330	-ELD-LD--A-Y		TA-M----NSS--T----A--S--
	Pseudomonas borbori	WP_090501182	-ELD-LD--A-Y		TS-M----MSS--T------IG--
	Pseudomonas azotifigens	WP_028238234	---D-LD--ARY		AKG--RD------------GIE--
	Pseudomonas pharmafabricae	WP_093984599	--LD------RY		EKSM----ISS--TM---A--S--
	Pseudomonas fluvialis	WP_093984599	--LD------RY		EKSM----ISS--TM---A--S--
	Pseudomonas aeruginosa	WP_003135287	---D-LD--V-Y		REG--GE-N----T------ID--
	Pseudomonas denitrificans	WP_023115615	---D-LD--V-Y		REG--GE-N----T------ID--
	Pseudomonas fulva	WP_013792958	-EID-L---ARY		TA-M----ISS--Q------IN--
	Pseudomonas pohangensis	WP_090197078	-ELD--D---RY		ESNM----ASS--TM-----FN--
	Pseudomonas pachastrellae	WP_083724988	--VD-LD--Q-Y		RKG--D-------T-------D--
	Pseudomonas benzenivorans	WP_090440977	-ELD-LD--A-Y		TS-M--G-ISS--T-------G--
	Pseudomonas punonensis	WP_073262489	-EID-L---ARY		TA-M----ISS--Q------IT--
	Pseudomonas furukawaii	WP_036993297	--LD--D----Y		DKTM--A-VSS--TM---A--N--
	Pseudomonas straminea	WP_093500035	-EID-LD--ARY		TA-M----ISS--Q------IN--
	Pseudomonas resinovorans	WP_041770460	--LD--D----Y		EKTM--A-VSS--TM---T--S--
	Pseudomonas taeanensis	WP_025164150	-EID-LD--A-Y		TA-M----ISS--T----A-IG--
	Pseudomonas daroniae	WP_131179614	-EID-LD--ARY		TS-M----ISS--Q------IA--
	Pseudomonas dryadis	WP_131176723	-EID-LD--ARY		TS-M----ISS--Q------IA--
	Pseudomonas argentinensis	WP_074881582	-EID-LD--ARY		TA-M----ISS--Q------IG--
	Pseudomonas indica	WP_084335839	--ID-L---V-Y		KDS--RD-M-S--T------IG--
	Perlucidibaca aquatic	WP_068857664	--ID-L---HRY		HKG--ND---S--T-------D--
	Pseudomonas panipatensis	WP_090263084	-ELD-L---A-Y		TAG--KD-H----TM-----ID--
	Pseudomonas nosocomialis	WP_138406510	--LD-L---PRY		S-GM-R--D----T----T-ID--
	Pseudomonas kuykendallii	WP_090225581	--VD-L---V-Y		QKG--ND-H----T----A-IG--
	Pseudomonas alcaligenes	WP_021698918	-EID-V---T-Y		NK-M-T--MS---TM-----IA--
	Pseudomonas flavescens	WP_084305251	-EID-LD--ARY		HS-M----ISS--Q-------A--
	Pseudomonas typographi	WP_190425084	-ELD-L---Q-Y		ASH--GN-M-S--T------I---
	Pseudomonas delhiensis	WP_089390109	--LD-L---A-Y		SSG--RD-H-S--TM-----IG--
	Pseudomonas jilinensis	WP_119701601	--ID--D--LRY		RKG--D--HGS--T------ID--
	Pseudomonas seleniipraecipitan	WP_070880791	-EID-LD--ARY		NS-M----ISS--Q------VA--
	Pseudomonas asuensis	WP_188866122	-ELD-LD--A-Y		YKG--RE-M----T------I---
	Pseudomonas zeshuii	WP_010796460	-ELD-LD--A-Y		YKG--RD-M----T------I---
	Pseudomonas luteola	WP_010796460	-ELD-LD--A-Y		YKG--RD-M----T------I---
	Pseudomonas oceani	WP_104736493	--ID-LD--Q-Y		RKG--D--N-S--T-------D--
	Pseudomonas citronellolis	WP_074982816	-ELD-L---A-Y		SSG--RD-H----TM-----IG--
	Pseudomonas humi	WP_058488268	-ELD-L---A-Y		SSG--RD-H----TM-----IG--
	Pseudomonas hussainii	WP_071873278	--LA--D---RY		EKG--KG-M-A--N----A--P--
	Pseudomonas japonica	WP_042120523	-ELD-L---Q-Y		ASHM-GG-MS---T-----K----
	Pseudomonas knackmussii	WP_043255593	-ELD-L---A-Y		TSG--SD-N----TM-A---IG--

Other *Pseudomonas*	*Pseudomonas massiliensis*	WP_040260532	-ELD-L---Q-Y	ASHM-GG-NSS--T------I---
	Pseudomonas oryzihabitans	WP_059313197	--LD------RY	DKAM-LD-ASS--T----A--S--
	Pseudomonas aestusnigri	WP_088276086	--ID-LD--Q-Y	RKG--DT-N-S--T-------D--
	Pseudomonas rhizoryzae	WP_058761639	--LD------RY	DKAM-LD-VSS--T----A--S--
	Pseudomonas psychrotoleran	WP_058761639	--LD------RY	DKAM-LD-VSS--T----A--S--
	Pseudomonas lundensis	WP_047272954	-EID-LD--A-Y	TAPM-G--ISS--T-----KV---
	Pseudomonas weihenstephanensis	WP_047281788	-EID-LD--A-Y	TAPM-G--ISS--T-----KV---
	Pseudomonas sichuanensis	WP_016712459	-EVD-L---QRY	ASHM-GG-HS---T-----KI---
	Pseudomonas parafulva	WP_016712459	-EVD-L---QRY	ASHM-GG-HS---T-----KI---
	Pseudomonas capeferrum	WP_016712459	-EVD-L---QRY	ASHM-GG-HS---T-----KI---
	Pseudomonas mosselii	WP_096048883	-EVD-L---QRY	ASHM-GG-HS---T-----KI---
	Pseudomonas reinekei	WP_075947183	-EID-LD--A-Y	SAPM-G--MSS--T-----KI---
	Pseudomonas soli	WP_023628812	-EVD-L---QRY	ASHM-GG-HS---T-----KI---
	Pseudomonas entomophila	WP_011536098	-EVD-L---QRY	ASHM-GG-HS---T-----KI---
	Pseudomonas guariconensis	WP_043212564	-EVD-L---QRY	ASHM-GG-HS---T-----KI---
	Pseudomonas piscis	WP_031320092	-EID-LD--A-Y	SAPM-G--MSS--T-----KI---
	Pseudomonas saponiphila	WP_016965546	-EID-LD--A-Y	SAPM-G--MSS--T-----KI---
	Pseudomonas aestus	WP_022639516	-EID-LD--A-Y	SAPM-G--MSS--T-----KI---
	Pseudomonas chlororaphis	WP_009046550	-EID-LD--A-Y	SAPM-G--MSS--T-----KI---
	Pseudomonas vancouverensis	WP_008018944	-EID-LD--A-Y	SAPM-G--MSS--T-----KI---
	Pseudomonas laurylsulfatiphila	WP_007970630	-EID-LD--A-Y	SAPM-G--MSS--T-----KI---
	Pseudomonas batumici	WP_040063517	-ELD-L---Q-Y	SSHM-GG-MSS--T-----KI---
	Pseudomonas jessenii	WP_090452613	-EID-LD--A-Y	SAPM-G--MSS--T-----KI---
	Pseudomonas fuscovaginae	WP_010453530	-ELD-L---Q-Y	SSHM-GG-MSS--T-----KI---
	Pseudomonas asplenii	WP_010453530	-ELD-L---Q-Y	SSHM-GG-MSS--T-----KI---
	Pseudomonas gingeri	WP_017126968	-ELD-L---Q-Y	SSHM-GG-MSS--T-----KI---
	Pseudomonas trivialis	WP_049710458	-ELD-L---Q-Y	SAHM-GG-VSS--T-----KI---
	Pseudomonas duriflava	WP_145138395	-EID-LD--A-Y	YKG--SG-V----TM-A---I---
	Pseudomonas antarctica	WP_064450261	-ELD-L---Q-Y	SAHM-GG-VSS--T-----KI---
	Pseudomonas veronii	WP_017849920	-ELD-L---Q-Y	SAHM-GG-VSS--T-----KI---
	Pseudomonas simiae	WP_010213857	-ELD-L---Q-Y	SAHM-GG-VSS--T-----KI---
	Pseudomonas panacis	WP_017849920	-ELD-L---Q-Y	SAHM-GG-VSS--T-----KI---
	Pseudomonas orientalis	WP_057724473	-ELD-L---Q-Y	SAHM-GG-VSS--T-----KI---
	Pseudomonas extremorientalis	WP_071492414	-ELD-L---Q-Y	SAHM-GG-VSS--T-----KI---
	Pseudomonas costantinii	WP_071482617	-ELD-L---Q-Y	SAHM-GG-VSS--T-----KI---
	Pseudomonas synxantha	WP_003187815	-ELD-L---Q-Y	SAHM-GG-VSS--T-----KI---
	Pseudomonas sivasensis	WP_003187815	-ELD-L---Q-Y	SAHM-GG-VSS--T-----KI---
	Pseudomonas paralactis	WP_003187815	-ELD-L---Q-Y	SAHM-GG-VSS--T-----KI---
	Pseudomonas libanensis	WP_003187815	-ELD-L---Q-Y	SAHM-GG-VSS--T-----KI---
	Pseudomonas lactis	WP_003187815	-ELD-L---Q-Y	SAHM-GG-VSS--T-----KI---
	Pseudomonas kitaguniensis	WP_152745942	-ELD-L---Q-Y	SAHM-GG-VSS--T-----KI---
	Pseudomonas haemolytica	WP_003187815	-ELD-L---Q-Y	SAHM-GG-VSS--T-----KI---
	Pseudomonas cedrina	WP_003171132	-ELD-L---Q-Y	SAHM-GG-VSS--T-----KI---
	Pseudomonas carnis	WP_003187815	-ELD-L---Q-Y	SAHM-GG-VSS--T-----KI---
	Pseudomonas arsenicoxydans	WP_090188351	-EID-LD--A-Y	SAPM-G--VSS--T-----KI---
Other Bacteria	*Perlucidibaca piscinae*	WP_022955167	--LD-L---VRY	R-G--GD------TM------D--
	Azotobacter vinelandii	WP_012703045	-----L---V-Y	RQS--QD-------------S--
	Azomonas macrocytogenes	WP_183165846	---D-L---VRY	R-G--RD-Q----T-------D--
	Azomonas agilis	WP_144571388	-----L---ARY	Y-G--QG-Q-I--T--A-I--N--
	Azotobacter salinestris	WP_152387406	---D-L---V-Y	RQS--LD-H----T-------P--
	Azotobacter chroococcum	WP_039801775	---D-L---V-Y	RQS--RD-HS---T-------A--
	Azotobacter beijerinckii	WP_090624902	-E-D-L---V-Y	RQS--RD-HS---T-------T--
	Entomomonas moraniae	WP_127164525	---D-L---A-Y	HKR--DT-Q----Q----IK-D--

Figure-S52

Partial sequence alignments of YkgJ family cysteine cluster protein showing a 1aa Ins (highlighted), which are uniquely shared by all species from the Linyingensis clade, except the deeper-branching *P. guangdongensis*.

Group	Species	Accession	145		182
**Linyingensis Clade** (*Geopseudomonas* **gen. nov.**) **(6/6)**	*Pseudomonas linyingensis*	WP_090305582	LPPYIDRPDEDADRERYQTVYS	DQ	AKAGAVAAPTAGLH
	Pseudomonas guangdongensi	WP_090213778	----------------------	-K	--------------
	"Pseudomonas oryzae"	WP_090347044	----------A-----------	-K	G-------------
	Pseudomonas sagittaria	WP_092427498	----------------------	--	S-------------
	Pseudomonas aromaticivoran	WP_217682279	----------A-----------	-R	D-------------
	Pseudomonas oryzagri	WP_229604670	----------A-----------	-R	D-------------
**Other** ***Pseudomonas***	*Pseudomonas alcaligenes*	WP_021700365	----------A-----------	-R	S-------------
	Pseudomonas fulva	WP_013792401	---------------------A		Q-------------
	Pseudomonas yangmingensi	WP_093474805	----------T----------A		D-------------
	Pseudomonas nitritireduc	WP_170859361	---------------------A		ER------------
	Pseudomonas nitroreducens	WP_084359507	---------------------A		ER------------
	Pseudomonas punonensis	WP_073263800	---------------------A		Q-------------
	Pseudomonas hydrolytica	WP_013716870	----------E----------A		E-------------
	Pseudomonas multiresinivorans	WP_169937040	---------------------A		ER------------
	Pseudomonas panipatensis	WP_090261463	---------------------A		ER------------
	Pseudomonas delhiensis	WP_089389517	---------------------A		ER------------
	Pseudomonas mucidolens	WP_084380017	----------------------		ERL-----------
	Pseudomonas peli	WP_090251192	----------Q----------A		DR------------
	Pseudomonas guineae	WP_090239403	----------Q----------A		DR------------
	Pseudomonas anguillisepti	WP_090375542	----------Q----------A		DR------------
	Pseudomonas leptonychotis	WP_136662876	----------Q----------A		DR------------
	Pseudomonas cedrina	WP_076949884	----------------------		QRL-----------
	Pseudomonas jilinensis	WP_080049549	----------T----------A		E-------------
	Pseudomonas tolaasii	WP_016972005	----------------------		QRL-----------
	Pseudomonas chlororaphis	WP_038634902	-----------S----------		ERL-----------
	Pseudomonas rhodesiae	WP_034139237	----------------------		QRL-----------
	Pseudomonas paralactis	WP_057703925	----------------------		QRL-----------
	Pseudomonas carnis	WP_034128030	----------------------		QRL-----------
	Pseudomonas veroni	WP_017846191	----------------------		QRL-----------
	Pseudomonas orientalis	WP_057723970	----------------------		QRL-----------
	Pseudomonas gessardii	WP_076964514	-----------S----------		ERL-----------
	Pseudomonas extremaustral	WP_010565219	----------------------		QRL-----------
	Pseudomonas panacis	WP_057004585	----------------------		QRL-----------
	Pseudomonas fluorescens	WP_053257886	----------------------		QRL-----------
	Pseudomonas lactis	WP_057710519	----------------------		QRL-----------
	Pseudomonas duriflava	WP_145136581	----------E----------A		R-------------
	Pseudomonas mendocina	WP_012019514	---------DE----------A		E-------------
	Pseudomonas sivasensis	WP_181639821	-----------S----------		QRL-----------
	Pseudomonas hydrolytica	WP_129481750	---------DE----------A		E-------------
	Pseudomonas fildesensis	WP_048722810	-----------S----------		QRL-----------
	Pseudomonas antarctica	WP_064454048	----------------------		QRP-----------
	Pseudomonas trivialis	WP_049711625	----------------------		QRP-----------
	Pseudomonas otitidis	WP_074970855	---------------------A		QH------------
	Pseudomonas pertucinoge	WP_188636249	----------K----------A		Q-------------
	Pseudomonas bauzanensis	WP_036989357	----------T----------A		Q-------------
	Pseudomonas palleroniana	WP_060754329	----------------------		QRP-----------
	Pseudomonas costantinii	WP_071485701	-----------S----------		QRL-----------
	Pseudomonas litoralis	WP_090273964	-----------S---------A		H-------------
	Pseudomonas canadensis	WP_028617855	-----------S----------		QRL-----------
	Pseudomonas edaphica	WP_138453875	-----------S----------		QRL-----------
	Pseudomonas kairouanensis	WP_135291174	-----------S----------		QRL-----------
	Pseudomonas cremoris	WP_185708191	-----------S----------		QRL-----------
	Pseudomonas reactans	WP_177004679	-----------S----------		QRL-----------
	Pseudomonas haemolytica	WP_153839144	-----------S----------		QRL-----------
	Pseudomonas nabeulensis	WP_135309838	-----------S----------		QRL-----------
	Pseudomonas libanensis	WP_057013163	-----------S----------		QRL-----------
	Pseudomonas grimontii	WP_090400685	-----------S----------		QRL-----------
	Pseudomonas brenneri	WP_090290542	-----------S----------		QRL-----------
	Pseudomonas azotoforma	WP_033898643	-----------S----------		QRL-----------
	Pseudomonas allii	WP_058423383	-----------S----------		QRL-----------
	Pseudomonas synxantha	WP_043046810	-----------S----------		QRL-----------
	Pseudomonas pudica	WP_085600669	----------G----------A		KR------------
	Pseudomonas meliae	WP_002552466	----------G----------A		QR------------
	Pseudomonas congelans	WP_054992948	----------G----------A		QR------------
	Pseudomonas hunanensi	WP_004576301	----------G----------A		QR------------
	Pseudomonas balearica	WP_043219081	----------GS---------A		QR------------
	Pseudomonas syringae	WP_025390480	----------GS---------A		ER------------
	Pseudomonas simiae	WP_010207886	-----------S----------		QRL-----------
	Pseudomonas donghuensi	WP_010223475	----------G----------A		ER------------
	Pseudomonas yamanorum	WP_093201386	-----------S----------		QRL-----------
	Pseudomonas proteolytica	WP_092233827	-----------S----------		QRL-----------
	Pseudomonas extremorientalis	WP_071489241	-----------S----------		QRL-----------
	Pseudomonas salomonii	WP_056858741	-----------S----------		QRL-----------
	Pseudomonas marginalis	WP_064053768	-----------S----------		QRL-----------
	Pseudomonas entomophil	WP_011532328	----------G----------A		ER------------
	Pseudomonas matsuiso	WP_188982852	---------D-----------A		QR------------
	Pseudomonas cichorii	WP_025258823	----------G----------A		ER------------

Group	Species	Accession	Region 1	Region 2
Other *Pseudomonas*	*Pseudomonas capeferrum*	WP_033701201	----------G----------A	ER------------
	Pseudomonas guariconensi	WP_090345272	----------G----------A	ER------------
	Pseudomonas reidholzensis	WP_119145663	----------G----------A	ER------------
	Pseudomonas wadenswilerensis	WP_115088649	----------G----------A	ER------------
	Pseudomonas marincola	WP_090514044	----------A----------A	ER------------
	Pseudomonas lutea	WP_037012517	---------D-----------A	QR------------
	Pseudomonas shirazica	WP_013971008	----------G----------A	ER------------
	Pseudomonas plecoglossicida	WP_013971008	----------G----------A	ER------------
	Pseudomonas asiatica	WP_013971008	----------G----------A	ER------------
	Pseudomonas alkylphenolica	WP_038614529	----------G----------A	ER------------
	Pseudomonas argentinensis	WP_074881070	----------A----------A	Q-------------
	Pseudomonas laurentiana	WP_163935052	----------G----------A	ER------------
	Pseudomonas inefficax	WP_112250695	----------G----------A	ER------------
	Pseudomonas xinjiangensis	WP_093391396	----------SS---------A	ER------------
	Pseudomonas caricapapayae	WP_055007826	----------S----------A	QR------------
	Pseudomonas viridiflava	WP_029241755	----------G----------A	ER------------
	Pseudomonas graminis	WP_074886366	---------D-----------A	QR------------
	Pseudomonas composti	WP_074938076	---------DE----------A	Q-------------
	Pseudomonas pseudoalcaligenes	WP_003459669	---------DE----------A	Q-------------
	Pseudomonas japonica	WP_042120851	----------G----------A	ER------------
	Pseudomonas knackmussii	WP_043249552	----------A----------A	ER------------
	Pseudomonas ovata	WP_109512601	----------G----------A	ER------------
	Pseudomonas gallaeciensis	WP_096003940	---------------------A	KRE-----------
	Pseudomonas abyssi strai	WP_096003940	---------------------A	KRE-----------
	Pseudomonas saudimassiliensis	WP_044498600	----------S----------A	QR------------
	Pseudomonas pachastrella	WP_083727537	---------------------A	KRE-----------
	Pseudomonas oleovorans	WP_150606211	---------DE----------A	Q-------------
	Pseudomonas nosocomialis	WP_138409197	----------A----------A	ER------------
	Pseudomonas formosensis	WP_090541058	----------S----------A	QR------------
	Pseudomonas sihuiensis	WP_092377259	---------DE----------A	Q-------------
	Pseudomonas indoloxydans	WP_074859261	---------DE----------A	Q-------------
	Pseudocitrobacter faecali	WP_108474806	---------------L------	SRP-----------
	Pseudomonas chengduensis	WP_017675192	---------DE----------A	QR------------
	Pseudomonas agarici	WP_060783357	----------G----------A	ERL-----------
	Pseudomonas kribbensis	WP_114885363	----------GS---------A	E-L-----------
	Pseudomonas laurylsulfatovora	WP_056721282	----------G----------A	ERL-----------
	Pseudomonas jessenii	WP_090453232	----------G----------A	ERL-----------
	Pseudomonas qingdaonensis	WP_043864453	----------G----------A	QRS-----------
	Pseudomonas helmanticensis	WP_134177631	----------GS---------A	E-L-----------
	Pseudomonas fuscovaginae	WP_010452737	----------G----------A	ERL-----------
	Pseudomonas asplenii	WP_010452737	----------G----------A	ERL-----------
	Pseudomonas moorei	WP_090316477	----------G----------A	ERL-----------
	Pseudomonas tremae	WP_024670465	----------GS---------A	QR------------
	Pseudomonas coronafacien	WP_024670465	----------GS---------A	QR------------
	Pseudomonas mosselii	WP_028689579	----------G----------A	ER------------
	Pseudomonas sediminis	WP_099526572	---------DE----------A	Q-------------
	Pseudomonas toyotomiensis	WP_074917871	---------DE----------A	Q-------------
	Pseudomonas humi	WP_043273034	----------A----------A	ER------------
	Pseudomonas citronellolis	WP_074978996	----------A----------A	ER------------
	Pseudomonas amygdali	WP_044318418	----------G----------A	QR------------
	Pseudomonas juntendi	WP_054911410	----------G----------A	QR------------
	Pseudomonas savastanoi	WP_011167997	----------G----------A	QR------------
	Pseudomonas avellanae	WP_005615739	----------G----------A	QR------------
	Pseudomonas coleopterorum	WP_090356402	---------DT----------A	E-------------
	Pseudomonas massiliensis	WP_040261915	----------T----------A	QH------------
	Pseudomonas caspiana	WP_087264576	---------DS----------A	QR------------
	Pseudomonas benzenivorans	WP_090442845	---------DA----------A	ER------------
	Pseudomonas tuomuerensis	WP_039606075	---------DT----------A	ER------------
	Pseudomonas indica	WP_084335706	---------DA----------A	ER------------
	Pseudomonas azotifigens	WP_028240042	---------D-----------A	QR------------
	Pseudomonas flexibilis	WP_039559736	---------DT----------A	ER------------
	Pseudomonas asuensis	WP_188865485	---------DE----------A	RN------------
	Pseudomonas profundi	WP_150299436	----------T----------A	QRE-----------
	Pseudomonas nitrititoler	WP_014853415	---------DS----------A	QR------------
	Pseudomonas typographi	WP_190425843	---------DS----------A	EH------------
	Pseudomonas soli	WP_094011727	----------G----------A	QR------------
	Pseudomonas vranovensis	WP_028942751	----------G----------A	QR------------
	Pseudomonas asturiensis	WP_073163633	----------G----------A	QR------------
	Pseudomonas sichuanensis	WP_110993513	----------G----------A	ER------------
Other Bacteria	*Plautia stali symbiont*	WP_010618344	----------E----L------	-RP-----------
	Azotobacter chroococcum	WP_089169003	----------A----------A	DR------------
	Escherichia marmotae	WP_001266487	---------------L------	E-P-----------
	Citrobacter koseri	WP_012133575	---------------L------	E-P-----------
	Citrobacter amalonaticus	WP_046476180	---------------L------	E-P-----------
	Shigella sonnei 53G	WP_001266503	---------------L------	E-P-----------
	Shigella flexneri	NP_706293.1	---------------L------	E-P-----------
	Shigella dysenteriae	YP_402037.1	---------------L------	E-P-----------
	Escherichia coli K12	NP_414939.1	---------------L------	E-P-----------

	Escherichia albertii	WP_001266503	---------------L------	E-P-----------
	Lelliottia jeotgali	WP_095280971	---------------L------	E-P-----------
	Kluyvera crysocrescens	WP_061284652	---------------L------	E-P-----------
	Pluralibacter gergoviae	WP_043085673	---------------L------	E-P-----------
	Enterobacter timonensis	WP_061708182	---------------L------	E-P-----------
	Kluyvera georgiana	WP_064547853	---------------L------	E-P-----------
	Kluyvera ascorbata	WP_035895142	---------------L------	E-P-----------
	Citrobacter farmeri	WP_042324571	---------------L------	E-P-----------
	Shigella boydii	WP_001266490	---------------L------	E-P-----------
	Escherichia fergusonii	WP_001266495	---------------L------	E-P-----------
	Citrobacter rodentium	WP_012904807	---------------L------	E-P-----------
	Kluyvera sichuanensis	WP_185667177	---------------L------	E-P-----------
	Klebsiella oxytoca	WP_032694142	---------------L------	E-P-----------
	Trabulsiella odontotermitis	WP_054180490	---------------L------	E-P-----------
	Citrobacter gillenii	WP_096758094	---------------L------	E-P-----------
	Trabulsiella guamensis	WP_038155157	---------------L------	E-P-----------
	Citrobacter freundii	WP_003835985	---------------L------	E-P-----------
	Citrobacter braakii	WP_003021529	---------------L------	E-P-----------
	Citrobacter werkmanii	WP_038638182	---------------L------	E-P-----------
	Citrobacter murliniae	WP_045440477	---------------L------	E-P-----------
	Citrobacter youngae	WP_006685742	---------------L------	E-P-----------
	Pseudescherichia vulneris NBRC	WP_042391570	---------------L------	E-P-----------
	Citrobacter portucalensis	WP_032942525	---------------L------	E-P-----------
	Citrobacter pasteurii	WP_005125124	---------------L------	E-P-----------
	Lelliottia amnigena	WP_064325010	---------------L------	Q-P-----------
	Citrobacter cronae	WP_085047646	---------------L------	E-P-----------
	Citrobacter europaeus	WP_043015380	---------------L------	E-P-----------
	Salmonella enterica	NP_459399.1	---------------L------	E-P-----------
	Klebsiella indica	WP_138358176	---------------L------	S-P-----------
	Metakosakonia massiliensis	WP_044184523	----------E----L------	--P-----------
	Enterobacter massiliensis	WP_044184523	----------E----L------	--P-----------
	Scandinavium goeteborgense	WP_125355344	---------------L------	Q-P-----------
	Leclercia adecarboxylata	WP_032616799	---------------L------	Q-P-----------
**Other Bacteria**	*Buttiauxella brennerae*	WP_064556983	---------------L------	Q-P-----------
	Superficieibacter electus	WP_103674869	---------------L------	Q-P-----------
	Yokenella regensburgei	WP_038254217	---------------L------	Q-P-----------
	Enterobacter lignolyticus	WP_062740478	---------------L------	Q-P-----------
	Kosakonia arachidis	WP_090118362	---------------L------	Q-P-----------
	Kosakonia oryziphila	WP_090135585	---------------L------	Q-P-----------
	Azotobacter beijerinckii	WP_090623920	----------A----------A	ER------------
	Klebsiella cf. planticola B43	WP_042712175	---------------L------	Q-P-----------
	Phytobacter diazotrophicus	WP_041852535	---------------L------	Q-P-----------
	Kluyvera intestini	WP_071195844	---------------L------	Q-P-----------
	Kosakonia oryzae	WP_064568216	---------------L------	Q-P-----------
	Kosakonia radicincitans	WP_007373483	---------------L------	Q-P-----------
	Kosakonia oryzendophytica	WP_061497243	---------------L------	Q-P-----------
	Hafnia alvei	WP_025800038	---------------L------	SRP-----------
	Buttiauxella ferragutiae	WP_064549191	-----------S---L------	Q-P-----------
	Buttiauxella izardii	WP_120066279	-----------S---L------	Q-P-----------
	Cedecea colo	WP_167612041	----------E----L------	Q-P-----------
	Edaphovirga cremea	WP_114191987	---------D-----L------	E-P-----------
	Klebsiella huaxiensis	WP_112214113	----------E----L------	T-P-----------
	Shimwellia blattae	WP_002444722	---------------L------	KRP-----------
	Photorhabdus temperata	WP_046973803	----------E----L------	ERP-----------
	Photorhabdus asymbiotica	WP_015835609	----------E----L------	ERP-----------
	Providencia rettgeri	WP_042846951	----------E--K-L------	E-P-----------
	Cedecea lapagei	WP_126357337	----------E----L------	Q-P-----------
	Klebsiella michiganensis	WP_008454534	----------E----L------	Q-P-----------
	Phytobacter ursingii	WP_047371609	----------E----L------	Q-P-----------
	Kluyvera intermedia	WP_047371609	----------E----L------	Q-P-----------
	Cedecea neteri	WP_061275365	----------E----L------	Q-P-----------
	Kosakonia sacchari	WP_017458843	----------E----L------	Q-P-----------
	Kosakonia pseudosacchari	WP_193821886	----------E----L------	Q-P-----------
	Kosakonia quasisacchari	WP_131410043	----------E----L------	Q-P-----------
	Siccibacter turicensis	WP_024550837	-----E---------L------	Q-P-----------

Figure- S53

Partial sequence alignments of the protein tRNA preQ1(34) S-adenosylmethionine ribosyltransferase-isomerase QueA showing a 2aa Ins (highlighted), which are uniquely shared by all species from the Linyingensis clade. This CSI is also shared by one outgroup species *Pseudomonas alcaligenes*.

Group	Species	Accession	89		128
**Resinovorans Clade** (***Metapeudomonas*** **gen. nov.) (6/6)**	*Pseudomonas resinovorans*	WP_016492426	AESFSNDPGSRATSLGLFRTSEVFDSE	EA	GHGRSMRLVGL
	Pseudomonas boanensis	WP_251701851	-DH---------S----Y--AQ----D	-P	--------I--
	Pseudomonas furukawaii	WP_004421887	--R---A-----S-------A------	-P	--------M--
	Pseudomonas lalkuanensis	WP_151134072	-D-----F---SS--------Q----D	-P	--------I--
	Pseudomonas otitidis	WP_165664669	--R--DR---L-S---V---A---Q--	AP	---L----E--
	Pseudomonas tohonis	WP_173174288	--R---E-E-H-SA--AY--G--YR--	QP	---L-------
**Other** ***Pseudomonas***	*Pseudomonas ullengensis*	WP_183089845	--H--DTDN-H-S---T---GDNYQ-T	QP	---L-L--T--
	Pseudomonas aeruginosa	WP_134302272	-T----R-N-YQS-----Q--QLYTGK		--Q-L-----
	Pseudomonas alcaligenes	WP_110682867	-NR---RL--YQS-------A-SYHGK		--Y-L-MD--
	Pseudomonas anguilliseptica	WP_244161142	-EV---RL--HQS-------A-SYRGK		--Y-L-MD--
	Pseudomonas flexibilis	WP_039607185	-TA---RV--HQS-------A-SYRGK		--Y-L-MD--
	Pseudomonas fluorescens	WP_115078328	-TR---RE--FQS-------Q-SYQGT		--Y-L-MD--
	Pseudomonas fluorescens	WP_191955686	-TA---LE--HQS-------Q-SYLGT		--Y-L-MD--
	Pseudomonas fragi	WP_095029478	-T----TE--NQS-----S-Q-SYVGS		--Y-L-MD--
	Pseudomonas gozinkensis	WP_192561455	-TR---RE--FQS-------Q-SYQGT		--Y-L-MD--
	Pseudomonas indica	WP_084336150	-TR----T--HQS---------SYIGK		--Y-L-MD--
	Pseudomonas marincola	WP_090514481	-ST---RV--YQS-------A-SYKGK		--L-L-MD--
	Pseudomonas putida	WP_159410961	-TT---A-N-YQS----YE-GQIYSGK		----L--Q--
	Pseudomonas sagittaria	WP_092431151	-T----TE--HQS---------SYFGQ		--Y-L-MD--
	Pseudomonas segetis	WP_089361254	-SQ---RL--YQS-------A-SYSGK		--L-L-MD--
	Pseudomonas stutzeri	WP_106156341	-T----AV--HQS-------A-SYSGK		--Y-L-MD--
	Pseudomonas thermotolerans	WP_036987338	-TT---V-N-YQS-----S-GQTYYGK		----L--E--
**Other Bacteria**	*Abscondita terminalis*	KAF5272356	-T---DR---LES---V-L-ESTYQGK		--Y-LK-K--
	Aeromonas australiensis	WP_235461966	--R---QID--QS---VY--A-TYQGK		--Y-L--D--
	Aeromonas dhakensis	WP_242762443	-RQ---QFN-MKS---VY--A-TYQGK		--Y-L--D--
	Aeromonas diversa	WP_005348364	-H---DR-D-LQS----Y--A-TYLGK		Y-Y-L--D--
	Aeromonas enteropelogenes	WP_225633246	-RQ---Q-N-LKS---VY--A-TYQGK		--Y-L--D--
	Aeromonas hydrophila	WP_194759080	-NQ---QFN-MKS---VY--A-TYQGK		--Y-L--D--
	Aeromonas jandaei	WP_201911216	-HQ---Q-N-MKS---VY--A-TYLGK		--Y-L--D--
	Aeromonas piscicola	WP_042866755	-RQ---QLN-MQS---VY--A-TYQGK		--Y-L--D--
	Aeromonas salmonicida	WP_139696219	-RQ---QLN--QS---VY--A-TYQGK		--Y-L--D--
	Aeromonas veronii	WP_204382145	--R---QLN--QS---VY--A-TYQGK		--Y-L--D--
	Algoriphagus aquaeductus	WP_111392113	--K---R-E-YQS---FYQ-A-TYQGK		--Y-L--D--
	Algoriphagus litoralis	WP_111670452	--K---Q-E-YQS---FYK-A-TYQGK		--Y-L--D--
	Algoriphagus marincola	WP_026945130	-TN---K-S-YKS---FYK-A-TYHGK		--L-L--D--
	Algoriphagus pacificus	WP_206586246	-DK---Q-E-YQS---FYK-A-TYQGK		--Y-L--D--
	Algoriphagus sanaruensis	WP_067545085	--K---R-E-YQS---FYM-A-TYHGK		--Y-L--D--
	Bdellovibrio bacteriovorus	WP_063204741	--K---TS-AH-S---YY--A-TYQGG		--L-L--D--
	Bradyrhizobium iriomotense	WP_211403884	-SV---R---NC----VY-CA-TYSGA		--F----D--
	Candidatus Solibacter sp.	HHI54496	-SR--DQ----MS---TYV-AGTYQGK		--Y-L--K--
	Clostridia bacterium	NCA86127	-KT--DK-N-YMS-I-FYL-DQTYQGK		--L-L--E--
	CycloBacteriaceae bacterium	NDE60889	--Q---R-E-HQS---F-K-A-TYQGK		--A-L--D--
	Flavisolibacter ginsengisoli	WP_139256370	--R---R-E-LQS---FYV-KNTYFGK		--L-L--E--
	Henriciella mobilis	RIJ15063	-DR---V---KM----A-V-A-TYYGK		--L-L--D--
	Lunatimonas lonarensis	WP_010856616	-YK---R-E-YQS---FY--G-TYQGK		--Y-L--D--
	Lunatimonas salinarum	WP_209330679	--R---R-E-YQS---FY--A-TYQGK		--Y-L--D--
	Mongoliitalea daihaiensis	WP_236137456	--R---QNS-YMS---FY--A-TYQGK		--Y-L--D--
	Photobacterium aphoticum	WP_107205093	--Q---R-Q-KQ----I-K-A-TYQGK		F-Y-L--D--
	Rickettsiella grylli	WP_006035364	-T---DRS--LES---V-L-ES-YQGK		--T-LK-K--
	Saprospiraceae bacterium	MCC6752704	-H----R---LMS---A---A-TYQGK		--Y-L--D--
	Solimonas flava	WP_028007567	--R---RD--LQS-------LNSYQGG		N-Y-L--A--
	Solimonas variicoloris	WP_051101783	--R---RD--LQS-------LNSYQGG		N-Y-L--A--
	Thermomonas carbonis	WP_189375878	-TR---EE--H---------A-TYQGG		N-Y-L-MD--

Figure- S54

Partial sequence alignments of Murein L, D-transpeptidase catalytic domain family protein showing a 2aa Ins (highlighted), which is uniquely shared by all species from the Resinovorans clade. The only exception of this CSI is that it is shared by one outgroup species *P. ullengensis*.

Group	Species	Accession	260		304
Resinovorans Clade (*Metapeudomonas* gen. nov.) (6/6)	*Pseudomonas resinovorans*	WP_016490742	ATYVAVAAEHPLATLAVQQLP	ADKAA	ELQAFIDECKRGGVAEADI
	Pseudomonas boanensis	WP_215371069	-------------A-------	T----	---T---------------
	Pseudomonas furukawaii	ELS24726	------------------R--	-----	-------------------
	Pseudomonas lalkuanensis	WP_151131702	-------------A----K--	-----	-------------------
	Pseudomonas otitidis	WP_074973169	-------------AI-LTK--	-----	-------------------
	Pseudomonas tohonis	WP_173178486	-------------AI-L-K--	-----	-------------------
Other *Pseudomonas*	*Pseudomonas linyingensis*	WP_090305227	-------------CI-LS---	--Q--	-------------------
	Pseudomonas taeanensis	WP_025167612	----------H--SV-LAR--	S----	-------------------
	Pseudomonas thermotolerans	WP_017937503	-------------AI-LSK--	PEQ--	-------------------
	Pseudomonas aeruginosa	WP_134553211	--------------Q-A-ND-		Q------------------
	Pseudomonas alcaligenes	WP_076428358	-------------QR-AESN-		AIA---A---S-------M
	Pseudomonas balearica	MBC7199850	----------------A-NN-		-------------------
	Pseudomonas cavernicola	WP_119952454	----------------AKGNA		----------H--------
	Pseudomonas chloritidismutans	WP_221103377	----------------A-DN-		-------------------
	Pseudomonas citronellolis	WP_074981928	----------------A-RE-		A------------------
	Pseudomonas delhiensis	WP_089391230	----------------A-RE-		A------------------
	Pseudomonas guangdongensis	WP_090211579	----------------A-GNA		-------------------
	Pseudomonas humi	WP_069862104	----------------A-RE-		A------------------
	Pseudomonas indica	MBU3055495	----------------AKDN-		------E------------
	Pseudomonas kunmingensis	WP_102832175	----------------A-NN-		-------------------
	Pseudomonas lopnurensis	WP_193682687	----------------A-ND-		-------------------
	Pseudomonas mangrovi	WP_108107510	----------------A-NN-		-------------------
	Pseudomonas marincola	WP_090514715	--------------Q-A-GN-		-------------------
	Pseudomonas multiresinivorans	WP_169942190	----------------AERD-		S------------------
	Pseudomonas nicosulfuronedens	WP_138520111	----------------AERD-		S------------------
	Pseudomonas nitritireducens	WP_184597571	----------------AERDG		S------------------
	Pseudomonas nitrititolerans	WP_213909508	----------------A-GN-		-------------------
	Pseudomonas nitroreducens	WP_138216928	----------------AERD-		S------------------
	Pseudomonas oryzihabitans	MBB4995666	--------------Q-A-GNA		-------------------
	Pseudomonas panipatensis	WP_090261818	----------------A-RD-		S-----------------L
	Pseudomonas pohangensis	WP_090196636	----------------A-NN-		-------------------
	Pseudomonas psychrotolerans	WP_058767784	--------------Q-A-GNA		-------------------
	Pseudomonas sagittaria	WP_092427742	-------------SI-A-GNA		-------------------
	Pseudomonas saudiphocaensis	WP_125839383	----------------A-NN-		-------------------
	Pseudomonas songnenensis	WP_122097921	----------------A-NN-		-------------------
	Pseudomonas stutzeri	WP_019405405	----------------A--N-		-------------------
	Pseudomonas taiwanensis	WP_023378552	--------------Q-A-GN-		A---------S-S-----M
	Pseudomonas viridiflava	WP_122624009	----------------A-NN-		------N---S-S-----V
	Pseudomonas xanthomarina	WP_065984388	----------------A-NN-		-------------------
	Pseudomonas zhaodongensis	WP_128120709	----------------A-NN-		-------------------
Other Bacteria	*Halopseudomonas aestusnigri*	WP_233048311	--------------Q-A-NN-		-------------------
	Halopseudomonas oceani	WP_104737242	--------------Q-A-NN-		-------------------

Figure- S55

Partial sequence alignments of a protein Leucine--tRNA ligase showing a 5aa Ins (highlighted), which is uniquely shared by all species from the Resinovorans clade. The only exception of this CSI is that it is shared by three outgroup species *P. linyingensis, P. taeanensis, P. thermotolerans*.

Group	Species	Accession	17		49
Resinovorans Clade (*Metapeudomonas* gen. nov.) (5/5)	*Pseudomonas resinovorans*	WP_028628607	RQHARVKIPGKVRYIA		RGERFEASLLDVSAGGF
	Pseudomonas boanensis	WP_215378116	-----------I---L		-N--M-QA-I-------
	Pseudomonas lalkuanensis	WP_151132090	----------------		-----------------
	Pseudomonas tohonis	WP_236205440	---------A-I--L-		NN--V-HR---L-----
	Pseudomonas otitidis	WP_165681623	-------L-A----LV		GK--V-HR---L-----
Other *Pseudomonas*	*Pseudomonas aeruginosa*	RCI70610	--F----L-ARI---G	A	NR-GVD-R---L-----
	Pseudomonas akappageensis	WP_166365501	------R--A-L----	A	DRQTL--RIE-L-----
	Pseudomonas alcaligenes	WP_061904329	--Y--L-L-A-I--RT	P	Q-QEL--Q---L-V---
	Pseudomonas alcaliphila	WP_074675774	-------L--RL-FTN	A	K---ID-R-Q-------
	Pseudomonas benzenivorans	WP_090442681	---------ARL--LT	K	QR--V--RT--L-----
	Pseudomonas carbonaria	WP_187671808	-----IR--A---F-G	R	NR-LV-QQ---I-----
	Pseudomonas cavernae	WP_119892508	---------A-L-FVG	K	NR-VA-QT---I-----
	Pseudomonas cavernicola	WP_119956020	-------L-A---FFG	K	SR-VV-QA---L-----
	Pseudomonas chengduensis	WP_196446808	-------L--RL-FTN	A	K---ID-R-Q-I-----
	Pseudomonas citronellolis	WP_058072972	-------L-ARI---G	A	NR-GVD-R---L-----
	Pseudomonas composti	WP_074935904	-------L--RL-FTN	A	K---ID-R-Q-------
	Pseudomonas delhiensis	WP_089389579	-------L-ARI---G	A	NR-GVD-R---L-----
	Pseudomonas guguanensis	WP_090427033	-------L--RL-FTN	A	K---ID-R-Q-I-----
	Pseudomonas helleri	WP_153382387	---------A-L-FLT	A	ERAPM----I-L----L
	Pseudomonas jinjuensis	WP_084309535	-------L-ARI---G	A	NR-GVD-R---I-----
	Pseudomonas khazarica	WP_210390503	-------L--RL--SN	A	QR--ID-R-Q-------
	Pseudomonas knackmussii	WP_043249469	-------L-ARI---G	A	NR-GVD-R---L-----
	Pseudomonas mendocina	WP_147811285	-------L--RL-FTN	A	K---ID-R---------
	Pseudomonas nicosulfuronedens	WP_138521980	-------L-ARI---G	G	NR-GVD-R---L-----
	Pseudomonas nitroreducens	WP_024765842	-------L-ARI---G	S	NR-GVD-R---L-----
	Pseudomonas oleovorans	WP_037046820	-------L--RL--SN	A	QR--ID-R-Q-------
	Pseudomonas sediminis	WP_099522382	-------L--RL-FTN	A	K---ID-R-Q-------
	Pseudomonas segetis	WP_089359437	------R--ATL----	K	NR-QV-VRV--L-----
	Pseudomonas taeanensis	WP_025164989	-------L-A---F-G	K	SR-VA-QN---L-----
	Pseudomonas toyotomiensis	WP_074913458	-------L--RL-FTN	A	K---ID-R-Q-------
	Pseudomonas ullengensis	WP_183089171	---------AVL--LT	K	GR--V-QRI--L----L
	Pseudomonas wenzhouensis	WP_230926786	-------L--RL-FTN	A	K---ID-R-Q-I-----
	Pseudomonas yangonensis	WP_161865047	-------L--RL-FTN	A	K---ID-R-Q-I-----
Other Bacteria	*Azotobacter beijerinckii*	WP_090619576	-------L---I-FLG	A	NR-TI-QR-I-------
	Azotobacter chroococcum	WP_131340640	-------L---I-FLG	P	NR-TI-QR---I-----
	Azotobacter chroococcum	WP_165891401	-------L---I-FLG	P	NR-TI-QR---I-----
	Azotobacter salinestris	WP_152388785	-------L---I-FLG	A	NR-TI-QR-I-------
	Azotobacter vinelandii	WP_012699747	-------L---I-FLG	P	NR-TI-QR-I-I-----

Figure- S56

Partial sequence alignments of Alginate biosynthesis protein Alg44 showing a 1aa Del (highlighted), which is uniquely shared by all species from the Resinovorans clade. No homolog was found for the ingroup species *P. furukawaii*.

Group	Species	Accession	61		93
**Resinovorans Clade (*Metapeudomonas* gen. nov.) (4/5)**	*Pseudomonas resinovorans*	WP_051246415	SVTAEQRAQVEAWL	Q	KGHAELAGSTVSPLLDVW
	Pseudomonas furukawaii	WP_003456365	--------------	G	Q-Q-----Y-----I---
	Pseudomonas lalkuanensis	WP_151132943	-------T------	-	--------------I---
	Pseudomonas boanensis	WP_215370058	--SDV--SE--S--	-	---D-V-SFNL---M---
	Pseudomonas otitidis	WP_222901412	--NE---TAL----		--RS---EFNL---V---
**Other *Pseudomonas***	*Pseudomonas abyssi*	WP_096003925	--SE---SA--S--		--RD---SF-----Q-A-
	Pseudomonas aeruginosa	WP_126394543	---E----T-----		--RS-VTKVE------A-
	Pseudomonas akappageensis	WP_166366617	--SE----K-----		-ARS--TSVE--------
	Pseudomonas alcaligenes	WP_110683068	--SE---T-L----		--RK--TEFNL---M---
	Pseudomonas alkylphenolica	WP_128326127	---E----A--S--		--RN--TKIE------A-
	Pseudomonas amygdali	WP_122302891	--SE---VA-K---		--RS--TEV-----I---
	Pseudomonas anguilliseptica	WP_090387418	--NE----A-----		--RD--VDF-A-------
	Pseudomonas arcuscaelestis	WP_203476801	---E---SA-----		--R---TNIEI-----A-
	Pseudomonas arsenicoxydans	WP_090181922	--SE----A-----		--RT--TTAE--------
	Pseudomonas atacamensis	WP_136493913	--NE----A-----		-NRT--TKYE--------
	Pseudomonas azotifigens	WP_043238129	---E---QR-QQ--		E-RP---EFQL---I---
	Pseudomonas balearica	WP_165563228	--SE---Q---S--		-ART---EFQ----M---
	Pseudomonas batumici	WP_040065437	--S-----S--T--		-ARN--TSVE----I---
	Pseudomonas benzenivorans	WP_090444824	--SE----K-----		--RS--VDFSLT--I---
	Pseudomonas borbori	WP_090497455	--SE----------		--RD--VDI-----M---
	Pseudomonas botevensis	WP_217834018	--NE----A-----		--RT--TNVEI-------
	Pseudomonas brassicacearum	WP_123369294	--SE----A-----		--RS--TEA-----I---
	Pseudomonas campi	WP_173205783	--S----DKL----		--RSD-T-F-L---M---
	Pseudomonas cannabina	WP_054999549	--SE----A-----		--RS--TEV-----I---
	Pseudomonas capeferrum	WP_181129852	--SE----T-----		--RS--TKVE------A-
	Pseudomonas caspiana	WP_087265050	---E----I-----		--RS--TKVE----M-A-
	Pseudomonas cavernae	WP_119894133	--SE---V------		--RN--VDFS----I---
	Pseudomonas cavernicola	WP_119954292	--SE---VA--T--		--RS-VTDF-A---I---
	Pseudomonas chloritidismutans	WP_221104080	--SE----K-D---		--CS---EYS----M---
	Pseudomonas chlororaphis	WP_218225162	--S-----N-----		-TRS--TSVE----M---
	Pseudomonas congelans	WP_236274035	--SE----A-----		--RS--TEV-----I---
	Pseudomonas coronafaciens pv.	KPW33666	--SE---VA-----		--RS--TEV-----I---
	Pseudomonas corrugata	RMM46097	--SE----A-----		--RS--TNM-----I---
	Pseudomonas endophytica	WP_055101726	--S-----A-----		-ARS--TSVE--------
	Pseudomonas entomophila	WP_011534862	--SE----A-----		--RS--TNIE------A-
	Pseudomonas eucalypticola	WP_176570244	---E----A-----		--RD--TKIE------A-
	Pseudomonas farsensis	WP_186535625	---E----A-----		--RS-VTQV-------A-
	Pseudomonas flavescens	WP_084304095	--SE---TA-----		--RS--VDFS--------
	Pseudomonas floridensis	WP_083182057	--SE----A-----		--RS--TEV---------
	Pseudomonas fluorescens	WP_039763008	--NE----A-----		-NRS--TKYE--------
	Pseudomonas frederiksbergensis	WP_205889190	---EA---A-----		--RS--TSAE----I---
	Pseudomonas fulva	WP_182140577	---E----A--S--		--RS--TKVE------A-
	Pseudomonas fuscovaginae	WP_017901935	--SE----A-----		--RS--TSVE--------
	Pseudomonas gingeri	WP_177094613	--S-----N-----		-TRS--TSAE----M---
	Pseudomonas guangdongensis	WP_090211437	--------A-----		--RS----AK-GE-V-A-
	Pseudomonas guineae	WP_090238362	--SE----A-----		--RD--VEF-A-------
	Pseudomonas guryensis	WP_182833598	--S----DKL----		-SRS--T-F-L---M---
	Pseudomonas hydrolytica	MCF2123521	--NE---TA-----		--RD--QNFEI-------
	Pseudomonas khazarica	WP_210389430	--AE---TA-----		--RD--Q-FEI-------
	Pseudomonas kunmingensis	WP_090523266	--SE----K-D---		--RG---EYS----M---
	Pseudomonas kuykendallii	PZP22819	--NE---T------		--RS--TEYKL---M---
	Pseudomonas lalucatii	WP_213638353	--SEA---------		--RS--IDFSA---M---
	Pseudomonas leptonychotis	WP_136665134	--NE----A-----		--RD--VDF-A---I---
	Pseudomonas lopnurensis	WP_193679605	--SE---EK-D---		--RS---EY-----M---
	Pseudomonas lutea	KGF65649	---E----A-----		N-RD--TKV-------A-
	Pseudomonas mandelii	WP_094471468	--SE----A-----		--RS--TEA-----I---
	Pseudomonas mangiferae	WP_143488327	--SE---QA--T--		-SRKD---F-I---M---
	Pseudomonas mangrovi	WP_108106784	--SE-------T--		-ARS--KD-SI-------
	Pseudomonas massiliensis	WP_040260742	--SE----T-----		--RS--TKVE----V-A-
	Pseudomonas meliae	WP_044343704	--SE---VA-----		--RS--TEV-----I---
	Pseudomonas mendocina	MBB5526852	--NE----A-----		--RD--QNYEI-------
	Pseudomonas monsensis	WP_186744795	--NE----A-----		--RS--T-M---------
	Pseudomonas monteilii	AMA46457	---E----A-----		--RS--TKIEI-----A-
	Pseudomonas moraviensis	WP_065614916	--SE----A-----		-TRS--TEM---------
	Pseudomonas mosselii	WP_096049716	--SE----A-----		--RS--TKIE------A-
	Pseudomonas multiresinivorans	WP_169939771	--SN---EL--G--		-QQP---EF-----I-A-
	Pseudomonas muyukensis	WP_217853650	--SE----A-----		--RS--TNIE------A-
	Pseudomonas nicosulfuronedens	WP_138525904	--S----ELIDR--		-QQP----FSL---I-A-
	Pseudomonas nitroreducens	WP_138214972	--S----EWIDG--		-QQP----FS----I-A-
	Pseudomonas nosocomialis	WP_138407567	---E---GA--S--		--RD--TEA-----M---
	Pseudomonas otitidis	WP_208276086	--NE---TAL----		--RS---EFNL---V---
	Pseudomonas pohangensis	WP_090193205	--S-----T-----		--RS--SSFEI---M---
	Pseudomonas psychrophila	WP_019408000	--S-----A-----		-ART--TNAE----I---
	Pseudomonas punonensis	WP_073268310	--SE----A-----		-SRS--VDFN--------
	Pseudomonas putida	WP_110967141	---E----A-----		--RS--TEV-----I---
	Pseudomonas reidholzensis	WP_119145990	--SE----A-----		--RS--TEIK------A-
	Pseudomonas reinekei	WP_075946045	--SEA---A-----		--RS--TEA-----I---
	Pseudomonas saudiphocaensis	WP_037022272	--SE---G------		--RS---EFS----M---
	Pseudomonas savastanoi	WP_004644496	--SE---VA-----		--RS--TEV-----I---

Group	Species	Accession	Sequence	Sequence
Other *Pseudomonas*	*Pseudomonas schmalbachii*	WP_208314675	--SE----L-DG--	-QQS--EKF-I---M-A-
	Pseudomonas seleniipraecipitan	WP_070882849	--SE----A-----	--RS--VDF-I---I---
	Pseudomonas soli	WP_023631687	---E----A-----	--RS--TNIE------A-
	Pseudomonas stutzeri	WP_102840345	---E----K-D---	--RS---EYS----M---
	Pseudomonas syringae	WP_024680835	--SE----A-----	--RS--TEV-----I---
	Pseudomonas taeanensis	WP_025165583	--ND----A-----	--RS--VDFN----M---
	Pseudomonas tolaasii	WP_016973199	--S-----A--S--	--RS--KSAE--------
	Pseudomonas tructae	WP_130265543	---E----A-----	--RS--TKVE------A-
	Pseudomonas ullengensis	WP_183089393	--N----DK-----	--RS--T-F-L---M---
	Pseudomonas umsongensis	MBB2885064	---EA---A-----	--RS--TSAE----I---
	Pseudomonas urumqiensis	WP_120996442	---E---QA-QT--	E-RQ----F-L---I---
	Pseudomonas vancouverensis	WP_093217547	--------A--T--	--RT--TSVE--------
	Pseudomonas vanderleydeniana	WP_186682466	--SE----A-----	--RS--TSVE--------
	Pseudomonas viridiflava	WP_122453126	--SE----A-----	--RS-VTDY-A---I---
	Pseudomonas vranovensis	WP_123567341	---E----A-----	--RS--TKIE------A-
	Pseudomonas xanthomarina	WP_073301986	--NE---GK-----	--RN--SEFN----M---
	Pseudomonas xionganensis	WP_160345192	--SE----A-----	--RN--VDF-A-------
	Pseudomonas yangonensis	WP_161897933	--NE----A-----	--RD--QNYEI-------
	Pseudomonas zhaodongensis	WP_122164088	--NE---G------	--RK--SDF-----M---
Other Bacteria	*Denitrificimonas caeni*	WP_205340342	---E----A-D---	-ARK--SSFEI-------
	Enterobacter hormaechei	MCE1690346	--SE----A-----	--RS--TNIE------A-
	Halopseudomonas gallaeciensis	WP_118129102	---E---SA--N--	--RD---SF-----Q-A-
	Halopseudomonas pachastrellae	WP_083727513	---E---NA--N--	--RD---SF-----Q-A-

Figure- S57

Partial sequence alignments of YggL family protein showing a 1aa Ins (highlighted), which is uniquely shared by the species from the *Resinovorans* clade except *P. otitidis*.

			597		641
**Resinovorans Clade (*Metapeudomonas* gen. nov.) (4/5)**	*Pseudomonas resinovorans*	WP_016489954	DSALLQEDAEKVLARAVTEAEQA		LANAGSYRETLERLASLREPVD
	Pseudomonas boanensis	WP_215375833	-------P--QA--K--A-----		--V-------------------
	Pseudomonas lalkuanensis	WP_151131060	-N-----A--RA--K--AD----		--S-S-----------------
	Pseudomonas furukawaii	WP_003458045	-G-----P--QA-----AA--T-		V-AG-----------A------
	Pseudomonas otitidis	WP_165673344	-----------A--A--A---R-	VAP	M-A-RC-S-A-G----------
**Other *Pseudomonas***	*Pseudomonas asuensis*	WP_188866218	EAT-FEH---R--LE-I-D---N	VEP	-SL-RN---A-N----------
	Pseudomonas benzenivorans	WP_090446126	-N-----P--QA--K--AA----	VLP	M-A-RC---A--H---------
	Pseudomonas campi	WP_173210753	-N---EAG--QT--A--AA----	VAP	--A-RR---A--Q---------
	Pseudomonas duriflava	WP_145138599	-AK-FEH---R--LE-I-----N	VEP	ISLSRN---A-N---R------
	Pseudomonas indica	WP_095648620	-GS----P------Q-LAA-DE-	VAP	--S-RC---A------------
	Pseudomonas lalucatii	WP_213640026	-A-----P--QA--E--AH----	VLP	M-A-RC---A--H---------
	Pseudomonas lutea	WP_197872466	-AK-FEH---R--LE-I-D---N	VEP	-SL-RN---A-N----------
	Pseudomonas luteola	WP_125888035	-AK-FEH---R--LE-I-D---N	VEP	-SL-RN---A-N----------
	Pseudomonas nanhaiensis	WP_223655906	-PS----G--QA--TSIQH---S	IAP	--QQRE-QR---T---------
	Pseudomonas oryzihabitans	WP_144961082	-A--FET---RT--A-IAD--RE	AAP	--E-RR---A--------A---
	Pseudomonas otitidis	WP_165665548	-----------A--A--A---R-	VAP	M-A-RC-S-A-G----------
	Pseudomonas psychrotolerans	WP_074583337	-A--FET---RT--A-IAD--RE	AAP	--E-RR---A--------A---
	Pseudomonas taeanensis	WP_025163710	--T--H-P--QM--Q--AD--L-	VMP	M-A-RC---A--H---------
	Pseudomonas taiwanensis	WP_023377954	EPKYFDNAN-FS-YS-IQQAD-A	VQP	M-A-RQ-S-A-A---A--D---
	Pseudomonas yangmingensis	WP_093474660	NAS----S--QA--S-ISA----	NAP	--ASRD-QRS--Q-----A---
**Other Bacteria**	*Aliivibrio finisterrensis*	WP_130045773	-L------------EK-EILAE-	LEP	VFI--N-Q-A-S---E------
	Aliivibrio fischeri	WP_233940769	-L---------A--EK-EILAE-	LEP	VFA--N-Q-A-S---E------
	Aliivibrio logei	WP_017020773	-L------------EK-EILAE-	LEP	VFI--N-Q-A-S---E------
	Aliivibrio sifiae	WP_105056629	-L------------EK-EILAE-	LEP	VFM--N-Q-A-S---E------
	Aliivibrio wodanis	VVV02692	-L------------EK-EILAE-	LEP	VFI--N-Q-A-S---E------
	Ferrimonas balearica	WP_222545501	NP--------RA--EV-SKVTAQ	VQP	MF-S-D-QDA-LA--Q------
	Halomonas alkaliphila	WP_198348723	-QS----E---T-FN---ASQ-Q	VAP	-FSS-D-QQA-DA--T------
	Halomonas humidisoli	WP_095603339	-QS----E---T-FN---ASQ-Q	VAP	-FS--D-QQA-DA--T------
	Halomonas venusta	WP_206048469	-QS--------T-FN--AASQ-Q	VAP	-FS--D-QQA-DA--T------
	Halopseudomonas sabulinigri	HDY97274	--N----A--QS--S-IEA--KS	VAP	--ADRQ-KDA-----A------
	Marinobacterium alkalitolerans	WP_209288620	-TS--T-SG--A--E-I-SI-AE	VQP	-CEQ-D--S---K-----TVI-
	Marinobacterium halophilum	WP_106591809	-TD----A--QA--S-L-A--HD	LQP	-FEQ-D--NA--------DVI-
	Marinobacterium stanieri	WP_076465592	-ES--S-----A--T-I-NI-SE	IQP	-CEQ-D--S---K-----A-I-
	Oceanospirillales bacterium	MBR9827075	-TD----A--QA--S-L-A--HD	LQP	-FEQ-D--NA--------DVI-
	Plesiomonas shigelloides	WP_039046352	--S----P---T--EQ-QSLSAK	LQP	-FAE-R-Q-A-TE--A------
	Pseudidiomarina marina	WP_126759792	NDD-------RA--N-IK--REV	SAA	AL---N-TAA-AS----QQ---
	Pseudidiomarina maritima	WP_092858004	NAS--T----CA--S-ISS-QK-	SDS	ALQ--N-T-A-SS----Q----
	Vibrio alginolyticus	WP_086048500	-L---------A--EN-EVMTE-	LEP	AFAT-N-Q-A-SK--D------
	Vibrio atlanticus	WP_012602954	-L---------A--EN-EVMTE-	LEP	AFAT-N-Q-A-SK--D------
	Vibrio bathopelagicus	WP_192889678	-LT--------A--EN-EVMTE-	LEP	AFAT-N-Q-A-SK--D------
	Vibrio brasiliensis	WP_006880037	-LS--------A--ES--VMTE-	LEP	AFAT-N-Q-A-SK--D------
	Vibrio campbellii	WP_228175603	-L------------ES-EVMTE-	LEP	AFAT-N-Q-A-SK--D------
	Vibrio cidicii	WP_196386933	-LS--------A--EN-EVLTE-	LEP	AFAM-N-QQA-SQ--A------
	Vibrio coralliirubri	WP_052880516	-L---------A--EN-EVMTE-	LEP	AFAT-N-Q-A-SK--D------
	Vibrio crassostreae	WP_210449763	-LT--------A--EN-EVMTE-	LEP	AFAT-N-Q-A-SK--D------
	Vibrio cyclitrophicus	WP_016767549	-L---------A--EN-EVMTE-	LEP	AFAT-N-Q-A-SK--D------
	Vibrio diabolicus	NTU35182	-L---------A--ES-EIMTE-	LEP	AFAT---Q-A-SK--D------
	Vibrio gallaecicus	WP_137375439	-LT--------A--EN-EVMTE-	LEP	AFAT-N-Q-A-SK--D------
	Vibrio ishigakensis	GAM54871	-L------------ES-EVLTE-	LEP	AFET-N-QQA-SK--E------
	Vibrio kanaloae	WP_017055489	-L---------A--EN-EVMTE-	LEP	AFAT-N-Q-A-SK--D------
	Vibrio lentus	WP_102268695	-LT--------A--EN-EVMTE-	LEP	AFAT-N-Q-A-SK--D------
	Vibrio marinisediminis	WP_182110215	-L------------QS-EVMTE-	LEP	AFAT-N-Q-A-SK--D------
	Vibrio mediterranei	WP_088876472	-L------------EN-EVMTE-	LEP	AFAT-N-Q-A-SK--D------
	Vibrio navarrensis	WP_172565061	-L---------A--EN-EVLTE-	LEP	AFAT-N-QQA-SQ--A------
	Vibrio parahaemolyticus	WP_141133728	-L---------A--ES-EVMTE-	LEP	AFAT-N-Q-A-SK--D------
	Vibrio ponticus	GAK87172	-L------------EN-EVMTE-	LEP	AFAT-N-Q-A-SK--D------
	Vibrio splendidus	WP_146453669	-L---------A--EN-EVMTE-	LEP	AFAT-N-Q-A-SK--D------
	Vibrio taketomensis	WP_162062249	-L------------EN-EVMTE-	LEP	AFAT-N-Q-A-SK--D------
	Vibrio tasmaniensis	WP_135445394	-L---------A--EN-EVMTE-	LEP	AFAT-N-Q-A-SK--D------
	Vibrio toranzoniae	WP_161674715	-LT--------A--EN-EVMTE-	LEP	AFAT-N-Q-A-SK--D------
	Vibrio vulnificus	EHG1330721	-L------------EN-EVMTE-	LEP	AFAT-N-Q-A-SK--D------
	Vibrio xiamenensis	WP_093278252	-L---------T--QN-EVMTE-	LEP	AFAT-N-Q-A-SK--D------
	Vibrio ziniensis	WP_165309920	-LS--------A--EN-EVLTE-	LEP	AFAT-N-QQA-SQ--A------

Figure- S58

Partial sequence alignments of a protein Glycine--tRNA ligase subunit beta showing a 3aa Del (highlighted), which is uniquely shared by the species from the Resinovorans clade except *P. otitidis*.

Group	Species	Accession	236		279
**Oryzihabitans Clade (Genus *Chryseomonas* emend.) (7/7)**	*Pseudomonas oryzihabitans*	WP_241809250	WFLPVPILVGVVTLALLRAILR	E	DSHHAPFLLTLALIFLGYSGL
	Chryseomonas asuensis	WP_188864475	---------L-TVVF------K	-	-A-VS--V---I---------
	Chryseomonas duriflava	WP_145141483	--M------V-TAVG--KS-Q-	-	EI-ST--V---I---------
	Chryseomonas luteola	WP_010797144	---------L-TVVF------K	-	EA-VS--V---I---------
	Pseudomonas psychrotolerans	WP_145010416	----------------------	-	---------------------
	Pseudomonas rhizoryzae	WP_058765116	----------------------	-	---------------------
	Pseudomonas zeshuii	WP_010797144	---------L-TVVF------K	-	EA-VS--V---I---------
**Other *Pseudomonas***	*Pseudomonas akappageensis*	WP_166360383	---------L-TLYG----VA-		NA-YT------V---------
	Pseudomonas alcaliphila	WP_074676058	--M---V--LLC-W-----VAN		NANYS----------------
	Pseudomonas alkylphenolica	WP_128322855	---------L-TLYG--K-VA-		NA-YT----------------
	Pseudomonas arcuscaelestis	WP_203480415	---------L-TLYG----VA-		NA-YT----------------
	Pseudomonas azerbaijanoriens	WP_217836421	--M------L-TMYG-I--VA-		NA-YT------V---------
	Pseudomonas balearica	WP_043220680	--M------VLC--G-----K-		YANYQ----------------
	Pseudomonas batumici	WP_040071116	---------L-TLYG----VA-		NA-YT----------------
	Pseudomonas borbori	WP_090498820	--I---L--LLC-W-----VAG		NA-YS----------------
	Pseudomonas brassicacearum	WP_123423562	---------L-TMYG----VA-		NA-YT------V---------
	Pseudomonas chlororaphis	WP_023969449	---------L-TLYG----VA-		NA-YT------V---------
	Pseudomonas cichorii	WP_221536250	--I------L-TFYG----VA-		-A-YT------V---------
	Pseudomonas cremoricolorata	WP_028696182	--M------M-TFY-----VAQ		-T-YT------V---------
	Pseudomonas deceptionensis	WP_048359306	---------L-TMYG--K--A-		KA-YT------V---------
	Pseudomonas farris	WP_217858117	---------L-TLYG-I--VA-		NA-YT------V---------
	Pseudomonas flexibilis	WP_039605665	---------LLCSW--Y---Q-		YA-YS----------------
	Pseudomonas fluorescens	WP_057400510	--M------L-TMYG-I--VA-		NA-YT------V---------
	Pseudomonas fragi	WP_095037904	---------L-TMYG--K--A-		KA-YT------V---------
	Pseudomonas frederiksbergensis	WP_123509185	---------L-TMYG-I--VA-		NA-YT------V---------
	Pseudomonas furukawaii	WP_004420210	---------L-C-W-----VAN		NAE----I---V---------
	Pseudomonas guariconensis	WP_196164933	--M------L-TFYG--C-VA-		NA-YT----------------
	Pseudomonas karstica	WP_154745017	---------L-TLYG----VA-		NA-YT------V---------
	Pseudomonas lalkuanensis	WP_226283508	--M------LIC-W-----VAN		NA-----V---V---------
	Pseudomonas laurentiana	WP_163932343	---------L-TLYG--K-VA-		NA-YT------V---------
	Pseudomonas lundensis	WP_047295647	---------L-TLY---K--A-		NA-YT------V---------
	Pseudomonas lutea	WP_191945816	--M---A--VLTMYG-FK-VA-		NANYT------V---------
	Pseudomonas mandelii	WP_140680983	---------L-TMYG-I--VA-		NA-YT------V---------
	Pseudomonas mendocina	WP_096827407	--M---V--LLC-W---KSVAE		NA-YS------V---------
	Pseudomonas mosselii	WP_096049780	--I------L-TFYG----VA-		NA-YT----------------
	Pseudomonas oleovorans	WP_206422373	--M---V--LLC-W-----VAN		NANYS----------------
	Pseudomonas oryziphila	WP_125859191	--M------L-TFYG----VA-		NA-YT----------------
	Pseudomonas otitidis	WP_236210010	---------L-C-W-----VAN		NAN-L--V---V---------
	Pseudomonas poae	WP_169896612	---------L-TMYG-I--VA-		QA-YT------V---------
	Pseudomonas protegens	WP_123724447	---------L-TMYG----VA-		NA-YT------V---------
	Pseudomonas putida	WP_020193615	--M------L-TCYG----VA-		NA-YT----------------
	Pseudomonas resinovorans	WP_077525132	--M------L-C-W-----VAD		NA-----V---V---------
	Pseudomonas saponiphila	WP_092309841	---------L-TMYG----VA-		NA-YT------V---------
	Pseudomonas synxantha	QUW66235	--M------L-TCYG----VA-		NA-YT----------------
	Pseudomonas syringae	WP_052967964	---------L-TLYG-I--VA-		NA-YT------V---------
	Pseudomonas taiwanensis	WP_179061126	--M------L-C-W-----VAD		NA-----V---V---------
	Pseudomonas toyotomiensis	WP_074913904	--M---V--LLC-W-----VAS		NANYS----------------
	Pseudomonas tructae	WP_130263027	---------L-TLYG----VA-		NA-YT------V---------
	Pseudomonas vancouverensis	WP_093230025	--M------L-TMYG-I--VA-		NA-YT------V---------
	Pseudomonas vanderleydeniana	WP_186682644	---------L-TLFG-I--VA-		NA-YT------V---------
	Pseudomonas wayambapalatensis	MBC3424552	---------L-TCYG----VA-		NA-YT----------------
**Other Bacteria**	*Acyrthosiphon pisum*	XP_016664730	------L--VLCSWGIV---K-		EA-YS------------F---
	Dickeya zeae	WP_168362798	----------LCAAGIV-SVR-		QA-Y-------G-----FT--
	Ewingella americana	WP_140470038	------V--FL-S-WM--TVNK		K--------------------
	Hafnia psychrotolerans	WP_188470620	------V--LF-S-WM---VNK		R--------------------
	Klebsiella pneumoniae	WP_171483065	--M------L-TFYG--K-VA-		NA-YT------V---------
	Pantoea ananatis	WP_028715777	------V--VLSAWG-R---K-		EA-YS------------F---
	Pantoea ananatis	BAK12549	------V--VLSAWG-R---K-		EA-YS------------F---
	Salmonella enterica	EAN8120997	--M------L-TFYG----VA-		NA-YT------V---------

Figure-S59

Partial sequence alignments of a protein Cytochrome d ubiquinol oxidase subunit II showing a 1aa Ins(highlighted), which is specific for all species from the Oryzihabitans clade.

Group	Species	Accession	485		513
**Oryzihabitans Clade (Genus *Chryseomonas* emend.) (7/7)**	*Pseudomonas oryzihabitans*	WP_059316469	AARELAGKFVENFKKFS	G	VSEAIVAAGPQL
	Chryseomonas asuensis	WP_188865893	S-KT-----I-------	-	--DG-K------
	Chryseomonas duriflava	WP_145137607	---A--T----------	-	-----K------
	Chryseomonas luteola	WP_112297951	S-KA-----I-------	-	-----K------
	Pseudomonas psychrotolerans	WP_058784691	-----------------	-	I----R------
	Pseudomonas rhizoryzae	WP_058772832	-----------------	-	I----R------
	Pseudomonas zeshuii	WP_196122115	S-KA-----I-------	-	-----K------
**Other *Pseudomonas***	*Pseudomonas abietaniphila*	WP_062379528	--KA---L-I------D		--D--K----K-
	Pseudomonas aeruginosa	WP_143479847	--KA---L-I------E		--D--K----K-
	Pseudomonas amygdali	RMP46050	--KA---L--------D		--D--K----K-
	Pseudomonas asplenii	WP_090204128	--KA---L-I------D		--D--K----K-
	Pseudomonas bananamidigenes	WP_065257789	--KA---L-I------D		--D--K----K-
	Pseudomonas batumici	WP_040071571	--KA---L-I------D		--D--K----K-
	Pseudomonas brassicacearum	WP_181290235	--KA---L-I------E		--D--K------
	Pseudomonas canadensis	WP_123475322	--KA---L-I------D		--D--K----K-
	Pseudomonas caspiana	WP_087271874	--KA---L-I------E		--D--K------
	Pseudomonas cedrina	WP_076951592	--KA---L--------E		-----K----K-
	Pseudomonas chlororaphis	WP_124323283	--KA---L-I------D		--D--K----K-
	Pseudomonas citronellolis	WP_074978142	--KG-----I------E		-----KN-----
	Pseudomonas delhiensis	WP_089393868	--KG-----I------D		-----KN-----
	Pseudomonas entomophila	WP_213659150	--KG---L-I------D		--D--K------
	Pseudomonas fluorescens	WP_214912647	--KA---L-I------E		--D--K------
	Pseudomonas fuscovaginae	WP_054058632	--KA---L-I------D		--D--K------
	Pseudomonas gingeri	WP_177100931	--KA---L-I------D		--D--K----K-
	Pseudomonas gozinkensis	WP_192563127	--KA---L-I------E		--D--K----K-
	Pseudomonas graminis	WP_172610342	--KA---L-I------E		--D--K----K-
	Pseudomonas gregormendelii	WP_205893588	S-KA---L--------D		--D--K----KM
	Pseudomonas grimontii	WP_090401092	--KA---L-I------E		--D--K----K-
	Pseudomonas kuykendallii	PZP22137	--KG--SL-I------D		-----K------
	Pseudomonas lactis	WP_236306928	--KA---L-I------E		--D--K----K-
	Pseudomonas massiliensis	WP_040260215	--KA---L-I------E		--D--R------
	Pseudomonas mediterranea	WP_055127567	--KA---L-I------E		--D--K------
	Pseudomonas mohnii	WP_198132094	--KA---L-I------D		--D--K------
	Pseudomonas moorei	WP_090327873	--KA---L-I------D		--D--K------
	Pseudomonas protegens	WP_152685873	--KA---L-I------D		--D--K----K-
	Pseudomonas putida	WP_046815333	--KA---L-I------D		--D--K----K-
	Pseudomonas saponiphila	WP_092318624	--KA---L-I------D		--D--K----K-
	Pseudomonas silesiensis	WP_064675396	S-KA---L--------D		--D--K----K-
	Pseudomonas stutzeri	WP_102852609	--K---N--I------D		-----K------
	Pseudomonas syringae	WP_038821051	--KA---L--------D		--D--K----K-
	Pseudomonas thivervalensis	WP_208665379	--KA---L-I------E		--D--K------
	Pseudomonas trivialis	WP_057008618	--KA---L--------E		-----K----T-
	Pseudomonas tructae	WP_130261893	--KA---L-I------E		--D--K------
	Pseudomonas urumqiensis	WP_120994275	--KA--E--I------D		-----R------
	Pseudomonas uvaldensis	WP_232777067	--KA---L-I------E		--D--K------
	Pseudomonas vancouverensis	WP_093227728	--KA---L-I------E		--D--K------
	Pseudomonas veronii	WP_169863827	--KA---L-I------D		--D--K----K-
	Pseudomonas viciae	WP_135843182	--KA---L-T------D		--D--K------
	Pseudomonas viridiflava	WP_122667031	--KA---L-I------E		--D--K------
	Pseudomonas xantholysinigenes	WP_186654053	--KG---L-I------D		--D--K------
	Pseudomonas xanthomarina	WP_065984579	--KQ-----I------D		---T-Q------
	Pseudomonas zhaodongensis	WP_122163302	--K---N--I------D		--D--K------
**Other Bacteria**	*Enterobacter hormaechei*	MBK4512922	--KG--KQ-I------E		--D--K------
	Listeria monocytogenes	MCB2527225	--KG--KQ-I------E		--D--K------

Figure- S60

Partial sequence alignments of a protein Phosphoenolpyruvate carboxykinase showing a 1aa Ins(highlighted), which is specific for all species from the Oryzihabitans clade but not for any other *Pseudomonas* species.

Group	Species	Accession	317		358
**Oryzihabitans Clade (Genus *Chryseomonas* emend.) (7/7)**	*Pseudomonas oryzihabitans*	WP_059316391	YNKLDLLDGVEPHIQRDDE	T	GRPIRVWVSAREGRGLELVGQA
	Chryseomonas asuensis	WP_188864674	---I---EN--------E-	-	-------A----NQ----IC--
	Chryseomonas duriflava	WP_145144239	---I---E-I-------E-	-	-T----------EQ--P-IR--
	Chryseomonas luteola	WP_019365459	---I---EN--------E-	-	-------A----N-----IR--
	Pseudomonas psychrotolerans	WP_058791030	-------------------	-	----------------------
	Pseudomonas rhizoryzae	WP_058760779	---I-------------E-	-	-----------NAQ--------
	Pseudomonas zeshuii	WP_010796922	---I---EN--------E-	-	-------A----N-----IR--
**Other *Pseudomonas***	*Pseudomonas aeruginosa*	MCF3990258	---V---PS---------S		-K-V---L--QT-E--D-LR--
	Pseudomonas alcaliphila	WP_074676246	------------Q----AD		---L---L----------LR--
	Pseudomonas campi	WP_173209724	---I---EH---Q----GD		-K-L---L---D-K----LR--
	Pseudomonas chengduensis	WP_017678665	------------Q----AD		-K-L---L----------LR--
	Pseudomonas composti	NYG64872	-------E----Q----GD		-K-V---L----------LR--
	Pseudomonas flexibilis	WP_039559333	---I---E----Q----G-		-K-Q---L-----K--D-LR-T
	Pseudomonas fluvialis	WP_184680004	---------I-------EQ		-K-L---L----------LK--
	Pseudomonas guguanensis	WP_090427961	-------E----Q----GD		-K-V---L----------LR--
	Pseudomonas guryensis	WP_182832143	---I---EH---Q----GD		-K-L---L---D-K----LR--
	Pseudomonas indoloxydans	WP_108234427	------------Q----AD		-K-L---L----------LR--
	Pseudomonas japonica	WP_181110843	-------EA-------GED		-K-Q-------D--------K-
	Pseudomonas jinjuensis	WP_084314002	F--I---PDF--Q---NED		---V---L----A---D-LR--
	Pseudomonas karstica	WP_154742416	-------E----Q----EN		---Q---L---D-S----LK--
	Pseudomonas khazarica	WP_134676025	-------E----Q----AD		-K-L---L----------LR--
	Pseudomonas laurentiana	WP_189395455	-------E----Q---NE-		-K-Q-------D----------
	Pseudomonas mendocina	WP_047590150	-------E----Q----AD		-K-L---L----------LR--
	Pseudomonas multiresinivorans	WP_169936104	---I---PDLQ-M----EL		-K-V---L----S-----LE--
	Pseudomonas nicosulfuronedens	WP_138524136	---I---PDLQ-M----EL		-K-V---L----S-----LE--
	Pseudomonas nitritireducens	WP_184595200	---I---PDIQ-M----EL		-K-V---L----S-----LE--
	Pseudomonas nitroreducens	WP_017520392	---I---PDIQ-M----EL		---V---L----S-----LE--
	Pseudomonas oleovorans	NYF61107	-------E----Q----AD		-K-L---L----------LR--
	Pseudomonas otitidis	WP_172434783	---I---ESM--Q----EH		-K-E---L-----K----LE--
	Pseudomonas ovata	WP_109512913	------VE--------NA-		-K-E-------D------LK--
	Pseudomonas pharmacofabricae	WP_101194040	-------E-I-------AA		-K-Q---L---D------LR--
	Pseudomonas pohangensis	WP_090196343	-------E-I-------VD		---E---L---D------LR--
	Pseudomonas putida	WP_192092225	-------E----Q----AD		-K-V-------D-------K--
	Pseudomonas resinovorans	WP_077523751	---V---AN--------ED		-K-E---L---D-S----LE--
	Pseudomonas rhizosphaerae	WP_043191142	-------E----Q----AD		-K-V-------D------IK--
	Pseudomonas rhodesiae	WP_094065106	-------E----Q----EN		-K-Q---L---D------LE--
	Pseudomonas sediminis	WP_179545906	------------Q----AD		-K-L---L----------LR--
	Pseudomonas sihuiensis	MBA2831252	------------Q----AD		-K-L---L----------LR--
	Pseudomonas syringae pv. berbe	RMM14651	-------E----Q----AD		-K-Q-------D----D-LK--
	Pseudomonas taiwanensis	WP_179062427	---V---AN--------ED		-K-E---L---D-S----LE--
	Pseudomonas thermotolerans	WP_017937111	---I--------Q----GS		---Q---L---D------LR--
	Pseudomonas toyotomiensis	WP_074913807	------------Q----AD		-K-L---L----------LR--
	Pseudomonas viridiflava	WP_122451581	-------E----Q----EA		---Q---L---D----D-LA--
	Pseudomonas viridiflava	WP_205039819	-------E----Q----AD		-K-Q-------D----D-LK--
	Pseudomonas wenzhouensis	WP_230926544	-------E----Q----GD		-K-L---L---D------LR--
**Other Bacteria**	*Azotobacter vinelandii*	WP_012699426	-------E-M--Q----VD		-K-L-------D------LR--
	Denitrificimonas caeni	WP_232365716	---I----N--------EQ		-K-V---I--AKQQ-IA-LQ--
	Endozoicomonas montiporae	WP_034879338	---I---EN---K-----Q		-K-V------MQ-E-----L--
	Halopseudomonas pelagia	WP_022961366	---I---E---------ES		-L-V------QQ-L-----Q--
	Halopseudomonas salegens	WP_092387090	---I---PAI-------E-		-V-V------QNNQ--D--N--
	Halopseudomonas xinjiangensis	WP_093391562	---V---ENF--QV---EN		-K-L------------D-LA--

Figure- S61

Partial sequence alignments of a protein GTPase HflX showing a 1aa Ins(highlighted), which is specific for all species from the Oryzihabitans clade but not shared by any other *Pseudomonas* species.

Group	Species	Accession	192		230
**Oryzihabitans Clade (Genus *Chryseomonas* emend.) (7/7)**	*Pseudomonas oryzihabitans*	WP_059313194	LRRLSQQLDAVETDQRELLDE	K	NVPSEISRLIRSLNRLL
	Chryseomonas asuensis	WP_188866320	-H---R----I-----D----	-	-----------------
	Chryseomonas duriflava	WP_145145063	-----R----I-----D----	R	-----V-----------
	Chryseomonas luteola	WP_125889607	-H---R----I-----D----	-	-----------------
	Pseudomonas psychrotolerans	WP_058789018	-----R----------D----	-	-----------------
	Pseudomonas rhizoryzae	WP_081049761	-----R----------D----	-	-----------------
	Pseudomonas zeshuii	WP_010796232	-H---R----I-----D----	-	-----------------
**Other *Pseudomonas***	*Pseudomonas anuradhapurensis*	WP_186687976	-----RE--E--AGA-DG-SG		EH-R-LL--TG------
	Pseudomonas bubulae	WP_130871932	------E--EI--GE-DS-S-		QH-R-LL--TG------
	Pseudomonas chlororaphis	WP_123328422	-----RE--QI-SGT--S-S-		QH-R-LL--TG------
	Pseudomonas entomophila	WP_011532639	-----RE--E--SGA-DG-SR		EH-R-LL--TG------
	Pseudomonas fulva	WP_196179507	-----RE--E--SG--QS-SG		DH-R-LL--TG------
	Pseudomonas japonica	WP_181112881	-----HE--EI-SG--QG-S-		RH---LL--T-------
	Pseudomonas lundensis	WP_186350154	------E--QI--GE-DS-S-		QH-R-LL--TG------
	Pseudomonas nitrititolerans	WP_214330220	-K---RE--Q--SGHLGQ-S-		HH-H-LL--T-------
	Pseudomonas oryzicola	WP_186680172	-----RE--E--AGA-DG-SR		EH-R-LL--TG------
	Pseudomonas plecoglossicida	WP_016391623	-----RE--E--SGA-DG-SA		EH-R-LL--TG------
	Pseudomonas putida	WP_012313114	-----RE--E--AGA-DG-SA		EH-R-LL--TG------
	Pseudomonas stutzeri	WP_199270140	-K---RE--Q--AGK-DR-SD		QH-Q-LL--T-------
	Pseudomonas versuta	WP_060691011	------E--QI--GE-DS-S-		QH-R-LL--TG------
	Pseudomonas weihenstephanensis	WP_048362836	------E--QI--GE-DS-S-		QH-R-LL--TG------
**Other Bacteria**	*Entomomonas moraniae*	WP_127162005	----KY---EI-AGK--Q---		------Y--T-------

Figure- S62

Partial sequence alignments of ATP-binding protein showing a 1aa Ins(highlighted), which is specific for all species from the Oryzihabitans clade but not for any other *Pseudomonas* species.

Group	Species	Accession	77		115
**Oryzihabitans Clade (Genus *Chryseomonas* emend.) (7/7)**	*Pseudomonas oryzihabitans*	WP_059313310	FGLNPLFTLHQGDAMKYDLSSI		AAGDRLRVVGNLPYNIS
	Chryseomonas asuensis	WP_188867317	-----Q--------L-FNFAEL		-N--K------------
	Chryseomonas duriflava	WP_145139630	-----N--------L-F-F-AL		-GS-K------------
	Chryseomonas luteola	WP_125888570	-----Q--------L-FNFAEL		VS-EK------------
	Pseudomonas psychrotolerans	WP_058760256	-------------------A--		-G---------------
	Pseudomonas rhizoryzae	WP_058768483	-------------------A--		-GS--------------
	Pseudomonas zeshuii	WP_010795977	-----Q--------L-FNFAEL		VS-EK------------
**Other *Pseudomonas***	*Pseudomonas aeruginosa*	WP_121500453	---ESR-S------L-F-FT-L	V	ES-EK------------
	Pseudomonas carbonaria	WP_187670362	---E-R-S------L-F-FAQL	S	SEPHS------------
	Pseudomonas citronellolis	WP_116425444	---K-N-R------L-F-FATL	P	Q--EK------------
	Pseudomonas delhiensis	KAF1056023	--IK-N-R------L-F-FT-L	P	Q--EK------------
	Pseudomonas fluvialis	WP_184684187	--I--Q--------L-F-FA--	G	SPS-K------------
	Pseudomonas guangdongensis	WP_090212958	---D-K--------L-F-FA-L	V	EG-EK------------
	Pseudomonas indica	WP_084335976	--IH-G--------L-F-FA-L	D	-SPRS------------
	Pseudomonas jinjuensis	WP_084314930	---K-N-A------L-F-FATL	P	---EK------------
	Pseudomonas knackmussii	WP_043248831	---K-N-R--------F-F-TL	P	Q--EK------------
	Pseudomonas matsuisoli	WP_188984055	---L-N-N--------F-FTTL	D	-GSHE------------
	Pseudomonas oryzae	WP_090350342	-----N----------F-F--L	V	SGD-K------------
	Pseudomonas panipatensis	WP_090264730	---K-N-R------L-F-F-TL	P	S--EK------------
	Pseudomonas schmalbachii	WP_208315845	---K-N--------L-F-F-TL	P	S--EK------------
	Pseudomonas thermotolerans	WP_027896267	---EKR--------L-F-FA-L	G	DKAHT------------
	Pseudomonas yangmingensis	WP_093472759	-----Q-A--R---L-F-FA-L	V	NSDEK--I---------
**Other Bacteria**	*Agrobacterium tumefaciens*	WP_166701634	---ESR-S------L-F-FT-L	V	ES-EK------------
	Atopomonas hussainii	WP_071870819	---K-N-A--S---L-F-FATL	G	SPEQP------------
	Atopomonas hussainii	WP_074867306	---K-N-A--S---L-F-FA-L	G	SPEQP------------
	Azotobacter beijerinckii	WP_090621677	---D-C-H--------F-FAQL	C	SKPHS--I---------
	Azotobacter beijerinckii	WP_090731329	---D-C-H--------F-FAQL	C	SKPHS--I---------
	Azotobacter salinestris	WP_152388386	---D-H-H--------F-FAQL	C	STPHS--I---------
	Azotobacter vinelandii	WP_012703151	---E-R-H------L-F-FARL	C	GTPHS--I---------
	Endozoicomonas arenosclerae	WP_062260695	-----E-RI-E---L-F-F--L	I	KE-ET--L---------
	Endozoicomonas montiporae	WP_034876300	-----N-RI-E---L-F-FARL	K	TDDKP--L---------
	Halopseudomonas bauzanensis	WP_074777536	--G--Q----K---L-F-FR-L	A	QP-EK------------
	Listeria monocytogenes	MCB2711210	---ESR-S------L-F-FA-R	V	ES-EK------------
	Thiopseudomonas alkaliphila	WP_053108754	-----Q--------LRF-FQ-L	E	PQAHS------------
	synthetic construct	AAT50842	---ESR-S------L-F-FA-L	V	ES-EK------------

Figure- S63

Partial sequence alignments of a protein 16S rRNA (adenine(1518)-N(6)/adenine(1519)-N(6))-dimethyltransferase RsmA showing a 1aa Del(highlighted), which is specific for all species from the Oryzihabitans clade but not found in any other *Pseudomonas* species.

Group	Species	Accession	36		75
Oryzihabitans Clade (Genus *Chryseomonas* emend.) (7/7)	*Pseudomonas oryzihabitans*	HJE68896	VEIHDGPEATLLTDDQVARAELVVVV		HTGAVDLSRFVGKR
	Chryseomonas asuensis	WP_188864410	----------------I---------		-------R------
	Chryseomonas duriflava	WP_145141637	----------M-S---I--S--II--		-------R------
	Chryseomonas luteola	WP_125887153	----------------I--S--I---		-------R--A---
	Pseudomonas psychrotolerans	WP_058792213	--------------------------		--------------
	Pseudomonas rhizoryzae	WP_058763569	----------------L---------		--------------
	Pseudomonas zeshuii	WP_010797217	----------------I--S--I---		-------R--A---
Other *Pseudomonas*	*Paucimonas lemoignei*	SQF95562	--VV-PRNPESKLTAEQIATADW-L-	V	N--E---T------
	Pseudomonas akappageensis	WP_166360687	-----RQHPERQLSQATID-AEW-L-	V	S---L---------
	Pseudomonas azotifigens	WP_028241300	--V--PRAIGSPLTA-EIAEAEL---	V	K--PLA-D------
	Pseudomonas balearica	WP_237146317	--V--PKAIGSPLTADQIANADL---	V	K--PLS-A------
	Pseudomonas chloritidismutans	WP_023445944	--V--PKAIGSPLTPAQIANADL---	V	K--PLS-Q------
	Pseudomonas chlororaphis	WP_210475457	--V--EAHPERQLSAATLE-AEW-L-	V	S--P--M-------
	Pseudomonas dryadis	WP_131177148	--V--PKAIGSPLS-ADIA-AEL---	V	K--EL--Q------
	Pseudomonas farsensis	WP_186536442	--VQ-SEHPERRLTSAQIAGADW-L-	V	S--P------T---
	Pseudomonas fluorescens	WP_015634132	--V--EAHPERQLSAATIE-AEW-L-	V	S--P--M-------
	Pseudomonas kunmingensis	WP_230697630	--V--PKAIGSPLTPAQIANADL---	V	K--PLS-Q------
	Pseudomonas kurunegalensis	WP_225521704	--VQEAGHPERQLSA-QIAEADW-L-	V	S------A------
	Pseudomonas kuykendallii	PZP23365	--V--PRAIGSPLS-ADIAGADL---	V	R--ELA-G------
	Pseudomonas lutea	WP_191944337	-----PRHPEKQLSAADIA-ADW-L-	V	N---L--T--S---
	Pseudomonas migulae	WP_182343501	--V---AHPERQLSAA-IDDAEW-LL	V	S-------------
	Pseudomonas monteilii	WP_200929097	--VQEAGHPERQLSA-QIAEADW-L-	V	T------A------
	Pseudomonas parafulva	WP_028631638	--VQ-TEHPERQLSAAQIAEADW-L-	V	S--S----------
	Pseudomonas piscis	WP_152899512	--V--PARPERQLSAATLA-AEW-L-	V	SS-P----------
	Pseudomonas protegens	WP_232643030	--V--DAHPERQLSATTIE-AEW-L-	V	S--P----------
	Pseudomonas putida	WP_019099748	--VQEAGHPERQLSA-QIAEADW-L-	V	S------V------
	Pseudomonas saponiphila	WP_092319546	--V--DAHPERQLSATTIE-AEW-L-	V	S--P----------
	Pseudomonas sessilinigenes	WP_124345728	--V--PARPERQLSAATLA-AEW-L-	V	SS-P----------
	Pseudomonas songnenensis	WP_126189532	--V--PKAIGSPLTAEQIAEADL---	V	K--PLS-A------
	Pseudomonas stutzeri	WP_156244318	--V--PKAIGSPLTADQIANADL---	V	N--PLS-Q------
	Pseudomonas syringae	WP_047579782	--V--PRNPEQQLSAEQIA-ADW-L-	V	N---I--N------
	Pseudomonas viridiflava	WP_122725669	--VF-PRNPEPKLTAEQIA-ADW-L-	V	N--E---T------
	Pseudomonas xanthomarina	WP_200636570	--V--PKAIGSPLTPAQIANADL---	V	K--PLS-Q------
	Pseudomonas zhaodongensis	WP_241529132	--V--PKAIGSPLTPAQIANADL---	V	K--PLS-Q------
Other Bacteria	*Azotobacter beijerinckii*	WP_090622229	--V--PKAIGSPLS-ADIA-AEL---	V	K--ELA-E------

Figure- S64

Partial sequence alignments of a protein PTS fructose transporter subunit IIBC showing a 1aa Del(highlighted), which is specific for all species from the Oryzihabitans clade.

Group	Species	Accession	164		201
Oryzihabitans Clade (Genus *Chryseomonas* emend.) (7/7)	*Pseudomonas oryzihabitans*	WP_007158679	LPINTQREFDIWKILKRQFG	N	HVSAERVVSGSGLVNLY
	Chryseomonas asuensis	WP_010798450	---G-P---E----------	-	------II--N------
	Chryseomonas duriflava	WP_145143620	---G-P---E--------Y-	-	------II--N------
	Chryseomonas luteola	WP_019365547	---G-P---E----------	-	------II--N------
	Pseudomonas psychrotolerans	WP_140216779	--------------------	-	-----------------
	Pseudomonas rhizoryzae	WP_058762541	--------------------	-	-----------------
	Pseudomonas zeshuii	WP_010798450	---G-P---E----------	-	------II--N------
Other *Pseudomonas*	*Pseudomonas alcaliphila*	WP_074681965	---GSP--A-L-QV-Y--L-		--R--D-L--N--LA--
	Pseudomonas chengduensis	WP_186351563	---GSP--AEL-Q--Y--L-		--R--D-L--N--LV--
	Pseudomonas citronellolis	KAF1065366	--VSSE-D-A--QL-REKY-		------AL-----E-I-
	Pseudomonas composti	WP_061237528	---GSP--AEL-QV-Y--L-		--R--D-L--N--LV--
	Pseudomonas denitrificans	QEY75891	--VTSE-D-AL-QL-REKY-		-------L-----E---
	Pseudomonas guguanensis	WP_090431138	---GSP--AEL-Q--Y--L-		--R--D-L--N--LV--
	Pseudomonas indoloxydans	WP_108233557	---GSP--VEL-Q--Y--L-		--R--D-L--N--LV--
	Pseudomonas khazarica	WP_134677444	---GSP--AEL-QV-HQ-L-		--R--D-L--N--LA--
	Pseudomonas khazarica	WP_210389061	---GSL--AEL-Q--Y--L-		--R--D-L--N--LA--
	Pseudomonas knackmussii	WP_156958142	--VTGE-D-A--QL-REKY-		-------L-----E---
	Pseudomonas kuykendallii	PZP21929	---GSE--A-L-SY-H--L-		-----NLL------S--
	Pseudomonas mendocina	NYK07069	---GSP--AEL-QV-Y--L-		--R--D-L--N--LA--
	Pseudomonas multiresinivorans	WP_169940650	--VTSE-D-AL-QL-REKY-		-------L-----E---
	Pseudomonas nicosulfuronedens	WP_138522459	--VTSE-D-AL-QL-REKY-		-------L-----E---
	Pseudomonas nitritireducens	WP_184589341	--VTSE-D-AL-QL-REKY-		-------L-----E---
	Pseudomonas nitroreducens	WP_017516661	--VTSE-D-AL-QL-REKY-		-------L-----E---
	Pseudomonas oleovorans	WP_241199371	---GSP--VEL-Q--Y--L-		--R--D-L--N--LV--
	Pseudomonas sediminis	WP_099523558	---GSP--AEL-Q--Y--L-		--R--D-L--N--LV--
	Pseudomonas segetis	WP_089358944	---GSA--AQL-QV-Y--L-		--R--D-L--N--LV--
	Pseudomonas toyotomiensis	WP_074918223	---GSP--AEL-Q--Y--L-		--R--D-L--N--LV--
	Pseudomonas wenzhouensis	WP_230927160	---GSP--AEL-Q--Y--L-		--R--D-L--N--LV--
	Pseudomonas yangonensis	WP_161866648	---ASP--AKL-Q--H--L-		--R--D-L--N--LA--
Other Bacteria	*Streptomyces sp. DH7*	WP_228927763	---GSP--VEL-Q--Y--L-		--R--D-L--N--LV--

Figure- S65

Partial sequence alignments of a protein Glucokinase showing a 1aa Ins(highlighted), which is specific for all species from the Oryzihabitans clade.

Group	Species	Accession	40		77
**Oryzihabitans Clade (Genus *Chryseomonas* emend.) (7/7)**	*Pseudomonas oryzihabitans*	WP_160922865	NWMGVSHGAVEQAKALA	E	ADGLAYLVVDFYGRNVRPQN
	Chryseomonas asuensis	WP_188868097	------Q--IDLT-R--	S	E---V-FI-----QS---S-
	Chryseomonas duriflava	WP_145142635	------Q--L-L--K--	K	E---I--------QG---T-
	Chryseomonas luteola	WP_019365388	------Q--IDL--R--	S	E---V-FI-----QK---A-
	Pseudomonas psychrotolerans	WP_145005081	--------------R--	-	--------------D-----
	Pseudomonas rhizoryzae	WP_058762461	------N-------Q--	-	--------------D-----
	Pseudomonas zeshuii	WP_010797813	------Q--IDL--R--	S	E---V-FI-----QK---A-
**Other *Pseudomonas***	*Pseudomonas aeruginosa*	WP_207943965	------A--L-I-RQV-		GR-RVV--A-L---D-----
	Pseudomonas aeruginosa SD9	OPF44733	------AA-LDI-RQV-		GR-HVV--A-L---D-----
	Pseudomonas alcaligenes	WP_187805923	------A--I-M-RQV-		-R-YVV-LA-V--ADL----
	Pseudomonas bijieensis	WP_176689298	-----GA--E-I--SV-		-N-YVV-IA-L--QD---S-
	Pseudomonas cavernae	WP_119893981	------A--I-M--Q--		GPDYVIFIA-V--QK-----
	Pseudomonas fluorescens	WP_108560347	------A--E-I--SV-		-K-YVV-IA-V--QA-----
	Pseudomonas frederiksbergensis	WP_205887272	------A--E-I--SV-		-K-YVV-IA-L--QT-----
	Pseudomonas glycinae	WP_236196575	------A--E-I--SV-		-K-YVV-IA-V--QA-----
	Pseudomonas koreensis	WP_198759071	------A--E-I--SV-		-K-YVV-IA-V--QA-----
	Pseudomonas mandelii	WP_221107014	------A--E-I--SV-		-K-YVV-IA-L--QS-----
	Pseudomonas putida	WP_098967877	------A--E-I--SV-		-K-YVV-IA-V--QA-----
**Other Bacteria**	*Arenimonas metalli*	WP_034212970	-----TDA---K--Q--		--DYVI-L--M--KG---A-
	Cedecea davisae	WP_016535242	------AA-I----SI-		-Q-YVV--A-L--QGK--T-
	Cedecea lapagei	WP_213713266	------A-------GI-		-Q-YVV--A-L--QGK--T-
	Cedecea neteri	SQA96979	------A-------GI-		-Q-YVV--A-L--QGK--T-
	Cedecea neteri	WP_039290886	------A-------GI-		-Q-YVV--A-L--QGK--T-
	Cedecea neteri	WP_039300995	------A-------GI-		-Q-YVV--A-L--QGKL-T-
	Cedecea neteri	WP_061275495	------A-------GI-		-Q-YVV--A-L--QGK--T-
	Enterobacter sp. Ag1	EJF29564	------T-------GI-		-Q-YVV--A-L--QGK--T-
	Enterobacter sp. Ap-1006	NIF48423	------A-------GI-		-Q-YVV-IA-L--QGK--T-
	Escherichia coli	WP_192514365	------AA-LDI-RQV-		GR-HVV--A-L---D-----
	Escherichia coli	WP_192530118	------AA-LDI-RQVD		GR-HVV--A-L---D-----
	Halopseudomonas salina	GGC90217	-----TE----K-QRVG		GSEYIVF-A-M--KDI----
	Klebsiella michiganensis	AJZ88472	------T-------GI-		-Q-YVV--A-L--QGQ--T-
	Klebsiella sp. Ap-873	NIG74752	------A--I----GI-		-Q-YVV--A-L--QGK--T-

Figure- S66

Partial sequence alignments of Dienelactone hydrolase family protein showing a 1aa Ins (highlighted), which is specific for all species from the Oryzihabitans clade.

Group	Species	Accession	415		457
Oryzihabitans Clade (Genus *Chryseomonas* emend.) (7/7)	*Pseudomonas oryzihabitans*	WP_059313726	TPERLLEQVRPDVLVKGGDY	K	SVEEVVGAQIVQGYGGEVRILG
	Chryseomonas asuensis	WP_188864540	---E--K--K----------	-	--D-----PL--A-----KV--
	Chryseomonas duriflava	WP_145141340	---E--R--H--I-------	-	-ID-----S---E-----KV--
	Chryseomonas psychrotolerans	WP_140220749	--------------------	-	----------------------
	Pseudomonas luteola	WP_125887237	---E--K--K----------	-	--D-----PL--A-----KV--
	Pseudomonas rhizoryzae	WP_058765260	--------------------	-	----------------------
	Pseudomonas zeshuii	WP_010797069	---E--K--K----------	-	--D-----PL--A-----KV--
Other *Pseudomonas*	*Pseudomonas aeruginosa*	PXC01217	--------------------		G--Q-------KA------V--
	Pseudomonas alcaliphila	WP_074676310	------A--Q----------		GI-Q----D---A------V--
	Pseudomonas bohemica	WP_110951000	---N--TH------------		--DQ----S---A------V--
	Pseudomonas chengduensis	WP_017678628	------K--Q----------		GIDQ----D---A------V--
	Pseudomonas citronellolis	WP_197887862	------A-------------		--DQ----D--A-------V--
	Pseudomonas composti	WP_061237408	------G-------------		GI-Q----D---A------V--
	Pseudomonas coronafaciens	KGS12316	---N--TH-K----------		G-DQ----D---A------V--
	Pseudomonas delhiensis	WP_089389948	------A-------------		--DQ----D--A-------V--
	Pseudomonas humi	WP_069863150	------A-------------		--DQ----D--A-------V--
	Pseudomonas khazarica	WP_134676043	------K--Q----------		G-DQ----D---A------V--
	Pseudomonas mendocina	WP_047591118	------K--Q----------		G--Q----D---A------V--
	Pseudomonas nicosulfuronedens	WP_138524056	------SE------------		G--Q----D--KA------V--
	Pseudomonas nitritireducens	WP_184594336	------SE------------		G--Q----D--KA------V--
	Pseudomonas oleovorans	WP_125874465	------K--Q----------		GIDQ----D---A------V--
	Pseudomonas panipatensis	WP_090262934	------G-------------		--DQ----D--E-------V--
	Pseudomonas punonensis	WP_073266455	------K--Q----------		GIDQ----D---A------V--
	Pseudomonas savastanoi	RMS84740	---N--SH-K----------		G-DQ----D---A---D--V--
	Pseudomonas savastanoi pv. glycinea	RMM87510	---N--SH-K----------		G-DQ----D---A------V--
	Pseudomonas sediminis	WP_099522743	------K--Q----------		D-DQ----D---A------V--
	Pseudomonas syringae	MCF5393149	---N--TH-K----------		G-DQ----D---A------V--
	Pseudomonas syringae pv. aptata	RMU75294	---N--TH-K----------		G-DQ----D---A------V--
	Pseudomonas syringae pv. avii	RMU55170	---N--TH-K----------		GIDQ----D---A------V--
	Pseudomonas wenzhouensis	WP_230926510	------K--Q----------		G-DQ----D---A------V--
Other Bacteria	*Azotobacter chroococcum*	WP_039801938	--------I-----------		G--Q----DL-RAH-----V--
	Acinetobacter baumannii	WP_242046860	PER-- --------------		G--Q-------KA------V--
	Staphylococcus argenteus	MBE2117219	--------------------		G--Q-------KA------V--

Figure- S67

Partial sequence alignments of a protein bifunctional D-glycero-beta-D-manno-heptose-7-phosphate kinase/D-glycero-beta-D-manno-heptose 1-phosphate adenylyltransferase HldE showing a 1aa Ins(highlighted), which is specific for all species from the Oryzihabitans clade.

Group	Species	Accession	209		245
**Oryzihabitans Clade (Genus *Chryseomonas* emend.) (7/7)**	*Pseudomonas oryzihabitans*	WP_197850824	PQRDIYGDLARGKPAWFVE	A	ASQGYWSELHNSLVRNLE
	Chryseomonas asuensis	WP_188868114	---E--S--L-Q----L-A	E	GDAE---------T----
	Chryseomonas duriflava	WP_145142579	------Y----Q--V---S	N	GDAE---------T----
	Chryseomonas luteola	WP_019365374	------F--S-Q----LIA	G	GEAE---------T----
	Pseudomonas psychrotolerans	WP_058774096	------A--S--------A	-	------------------
	Pseudomonas rhizoryzae	WP_058762478	------A--S--------A	-	------------------
	Pseudomonas zeshuii	WP_010797795	------F--S-Q----L-A	G	GEAE---------T----
**Other *Pseudomonas***	*Pseudomonas aeruginosa*	PBD27351	------SQ---Y-LS----		DDAD--N--N-R-T----
	Pseudomonas carnis	WP_197900415	-----FAQ-T-I-L---CD		DDAD--N------T-Y--
	Pseudomonas cavernae	WP_119893332	------AQ-S-NRLP---D		GNAD--N--N-H-T----
	Pseudomonas citronellolis	WP_074986038	------SQ-T-I-LS-TLG		DDAD--N--Y-R-T----
	Pseudomonas delhiensis	WP_089391947	------AQ-T-I-LS-TLA		DDAD--N--Y-R-T----
	Pseudomonas fluorescens	WP_137271210	-----FAQ-T-I-L---CD		DDAD--N------T-Y--
	Pseudomonas indica	WP_095648469	---E-FAQM--H-LS---D		DDTV--N------T----
	Pseudomonas khavaziana	WP_217883209	-----FAQ-T-I-L---CD		DDAD--N------T-Y--
	Pseudomonas lactis	HJH17581	-----FAQ-T-I-L---CD		DDAD--N------T-Y--
	Pseudomonas matsuisoli	WP_188985023	---E-FAQ---QRFE--ST		DDAA--N------T----
	Pseudomonas orientalis	WP_104504355	-----FAQ-T-I-L---CD		DDAD--N------T-Y--
	Pseudomonas paralactis	WP_198707035	-----FAQ-T-I-L---CD		DDAD--N------T-Y--
	Pseudomonas pohangensis	WP_090193858	-----FFEMSKE-LG---G		DQAKD-N--G---T-H--
	Pseudomonas salmasensis	WP_186606914	-----FAQ-T-I-L---CD		DDAD--N------T-Y--
	Pseudomonas synxantha	WP_124378994	-----FAQ-T-I-L---CD		DDAD--N------T-Y--
	Pseudomonas viridiflava	WP_122736939	-----FAQ-T-I-L---CD		DDAD--N------T-Y--
**Other Bacteria**	*Achromobacter insolitus*	WP_207545771	------SQ---Y-LS----		DDAD--N--N-R-T----
	Klebsiella pneumoniae	NDM57831	------SQ---Y-LS----		DDAD--N--N-R-T----
	Escherichia coli	MBE1188546	------SQ---Y-LS----		DDAD--N--N-R-T----

Figure- S68

Partial sequence alignments of a protein Zinc transporter ZntB showing a 1aa Ins(highlighted), which is specific for all species from the Oryzihabitans clade.

Group	Species	Accession	180		220
**Oryzihabitans Clade (Genus *Chryseomonas* emend.) (7/7)**	*Pseudomonas oryzihabitans*	WP_208691271	SNCPVTGQPDWGTVVIDYE	E	GLALEPASLLRYLVAFRQHAD
	Chryseomonas asuensis	WP_188868185	------------S--V---	G	TSTIDR----A-I-S--R---
	Chryseomonas luteola	WP_125888308	------------S---E-K	G	RAV-DR----A-IIS--R---
	Chryseomonas psychrotolerans	WP_140216268	-------------L-V--Q	-	------E-----V--------
	Pseudomonas rhizoryzae	WP_058773128	-------------L-V--Q	-	------E-----V--------
	Pseudomonas zeshuii	WP_010794882	------------S---E--	G	RAV-DR----A-IIS--R---
	Pseudomonas duriflava	WP_244309164	------------S------	G	AQ-DREA--A-V-S------
**Other *Pseudomonas***	*Pseudomonas aeruginosa*	KEA39874	-------------L-V--Q		-P--D-----A---S----Q-
	Pseudomonas azotifigens	WP_028238002	------------SL-V--R		-P--Q-E---A-V-S------
	Pseudomonas citronellolis	WP_074980988	-------------L-V--A		-P--D-----A-V-S----Q-
	Pseudomonas daroniae	WP_131192310	------------S--VE-R		-A--------A---S------
	Pseudomonas delhiensis	WP_089392012	-------------L-V--A		-P--D-----A-V-S----Q-
	Pseudomonas flavescens	WP_179538391	------------S--VE-R		-A--------A---S------
	Pseudomonas fulva	WP_042554128	------------S--VE-R		-A--------A---S------
	Pseudomonas humi	WP_069866264	-------------L-V--A		-P--D-----A-V-S----Q-
	Pseudomonas khazarica	WP_210389312	------------SL-VH-Q		-A--D-----A---S------
	Pseudomonas knackmussii	WP_160287581	-------------L-V--S		-P--D-----A-V-S----Q-
	Pseudomonas kunmingensis	WP_104097837	------------S--VE-R		-A--Q-E---A---S------
	Pseudomonas lopnurensis	WP_193680566	------------S--VE-R		-A--Q-E---A---S------
	Pseudomonas mendocina	WP_047591447	------------SL-VH-Q		-P--D-----A---S------
	Pseudomonas oleovorans	HIQ45960	------------SL-VH-Q		-A--D-----A---S------
	Pseudomonas stutzeri	WP_063543970	------------S--VE-R		-A--Q-E---A---S------
	Pseudomonas peli	WP_090247742	-------------L-VE-R		----DH----A---S------
	Pseudomonas seleniipraecipitan	WP_070883424	------------S--VQ-R		-A--------A---S------
**Other Bacteria**	*Enterobacter hormaechei*	MBX8630753	-------------L-V--R		-P--D-V---A---S----Q-
	Stenotrophomonas maltophilia	KAF1050782	-------------L-V--A		-P--D-----A-V-S----Q-
	Azomonas macrocytogenes	WP_183165349	------------SI-V--R		-N--D-G---A-I-S------
	Azomonas agilis	WP_144572441	------------SL----R		----------A-I-S--R---
	Azotobacter beijerinckii	WP_090936164	------------S--V--R		-P--D-----A---S------
	Azotobacter chroococcum	WP_131253561	------------S--V--R		-P--D-----A---S------
	Azotobacter vinelandii	WP_175555976	------------SL-V--H		-P--D-----A-V-S------

Figure- S69

Partial sequence alignments of a protein NADPH-dependent 7-cyano-7-deazaguanine reductase QueF showing a 1aa Ins(highlighted), which is specific for all species from the Oryzihabitans clade.

Group	Species	Accession	27		75
**Thermotolerans Clade (*Zestomonas* gen. nov.) (4/4)**	*Pseudomonas thermotolerans*	WP_017939833	LLGQVVDRRLNLRSWRDLLAHLRG	SALHEA	PVQIDERELLFRLLGRLAK
	Pseudomonas carbonaria	WP_187670356	----------R--G----------	---EAP	AAE--------I-------
	Pseudomonas cavernae	WP_119891967	----------R-D---A--S----	- -AA-	-A L--G----V-------
	Pseudomonas insulae	WP_205349415	-----L----R-HD--A--T----	-VTS -	-RL -------L-------
**Other *Pseudomonas***	*Pseudomonas aeruginosa*	MBF3224246	----LL----R-E---G---R---		RAVN--DD---Q---Y---
	Pseudomonas aestus	ERO63278	----AL---MQ-H--AH-RER-G-		RPALRND----V-------
	Pseudomonas alcaligenes	MBB4818528	----------K-D--AS-R-R--R		GMAVEQED---V-------
	Pseudomonas alkylphenolica	WP_128323443	----AM----Q-H--AH-RER-G-		RPALRDD-V--V-------
	Pseudomonas alliivorans	WP_210075432	----AL----K-Y--AH-RER-G-		RPAVHDD----V-------
	Pseudomonas avellanae	WP_024699149	----AL----K-Q--AH-RER-G-		QAA-P-D----V-------
	Pseudomonas azerbaijanoriens	WP_217836514	----AL----H-Q--GH-REK-G-		RPVLRND----V-------
	Pseudomonas borbori	WP_090498324	----------R-H--AA-RER-GA		RPYPGDA----V-------
	Pseudomonas brenneri	WP_090292205	----AL----Q----AH-RER-G-		RPALRND----V-------
	Pseudomonas chlororaphis	WP_123327459	----AL----Q-H--AHVRER-G-		RPALRND----V-------
	Pseudomonas deceptionensis	WP_048361112	----AL----Q-H--AH--ER-GA		RPALRND----V-------
	Pseudomonas floridensis	WP_083184409	----AL----K-Y--AH-RER-G-		RPA-HDD----V-------
	Pseudomonas fluorescens	VVP52293	----AL----H-Q--AH-REK-G-		RPALRND----V-------
	Pseudomonas frederiksbergensis	WP_123410532	----AL----H-Q--AH-REK-G-		RPALRND----V-------
	Pseudomonas furukawaii	WP_003450731	-----L----K-D--SA-W-R---		RSE-ASGD---V-------
	Pseudomonas fuscovaginae	WP_054064663	----AL----Q-H--AH-RER-G-		KPALRND----V-------
	Pseudomonas helleri	WP_153378008	----TL----Q-H--AH-RER-G-		RPALRND----V-------
	Pseudomonas huaxiensis	WP_110971542	----AM----Q-H--AH-REK-G-		RPVLRDD-V--V-------
	Pseudomonas lalkuanensis	WP_151131533	-----F----K-E--G--R-R---		DPA-E-QD---V-------
	Pseudomonas laurylsulfatiphila	WP_104449997	----AL----H-Q--GH-REK-G-		RPVLRND----V-------
	Pseudomonas marginalis	KTC16405	----AL----K-Y--AH-RER-G-		RPAVHDD----V-------
	Pseudomonas massiliensis	WP_040259814	----L-----D-Q--A--W-R---		KAGLA-GD---V-------
	Pseudomonas mohnii	WP_196585058	----AL----H-Q--GH-REK-G-		-PVLRND----V-------
	Pseudomonas putida	WP_064302976	----AM----Q-H--AH-REK-G-		RPVLRDD-V--V-------
	Pseudomonas reinekei	WP_075946149	----AL----H-Q--AH-REK-G-		RPALRND----V-------
	Pseudomonas resinovorans	WP_077520852	---H-L----H-D--GA-R-R---		DPA--QQD---I-------
	Pseudomonas saxonica	WP_146426196	----TL----Q-H--AH-RER-G-		RPALRND----V-------
	Pseudomonas schmalbachii	MBO3277560	-----L----Q------VVE-M-S		EPPLADE----V-------
	Pseudomonas stutzeri	WP_103456026	-----L----G-D--AS-RKR-A-		KPVLQDN----F-------
	Pseudomonas syringae	WP_058419007	----AL----K-Q--AH-RER-G-		RAA-P-D----V-------
	Pseudomonas taiwanensis	WP_179058121	---H-L-Q--H-D--GA-R-R---		DPG--QQD---I-------
	Pseudomonas vanderleydeniana	WP_186685343	----AL----Q-H--AH-RER-G-		KPALRND----V-------
	Pseudomonas viridiflava	WP_122668072	----AL----K-Y--AH-REC-G-		RPAVHDD----V-------

Figure- S70

Partial sequence alignments of TerB family tellurite resistance protein showing a 6aa Ins (highlighted), which is uniquely shared by all species from the Thermotolerans clade.

Group	Species	Accession	175		206
**Thermotolerans Clade (*Zestomonas* gen. nov.) (4/4)**	*Pseudomonas cavernae*	WP_119894903	DAAPVSFTYVNLSLRSG		LRQRLDGRLTLPDGQP
	Pseudomonas insulae	WP_205348737	N---I--S-L-------		S--------V------
	Pseudomonas thermotolerans	WP_017938668	-TE-F-L-GI----Q--		S-------FS------
	Pseudomonas carbonaria	WP_187671334	----F----A-------		T---------------
**Other *Pseudomonas***	*Pseudomonas allii*	WP_179029797	-Q---TL---G---HT-	V	T-----A---------
	Pseudomonas allokribbensis	WP_192559783	-Q--LT----G-N-KT-	L	N-----A---------
	Pseudomonas amygdali	WP_016981176	----LTL-------AT-	I	YH----A---------
	Pseudomonas avellanae	WP_024419636	----LTL-------TT-	S	YH----A---------
	Pseudomonas azotoformans	WP_071494146	-Q---TM---G---HT-	V	T-----A---------
	Pseudomonas canadensis	WP_123478171	-Q---TL---G---HT-	I	T-----A---------
	Pseudomonas cavernicola	WP_119952252	GE--LT--HA-----N-	V	SH--------------
	Pseudomonas cedrina	WP_076951167	-Q---TL---G---HT-	M	T-----A---------
	Pseudomonas corrugata	WP_175385523	-Q--L-L---G----T-	V	S-----A---------
	Pseudomonas ekonensis	WP_217893658	-H--LT----G-N-KT-	A	V-----V---------
	Pseudomonas fluorescens	KTC63140	-Q---TL---G---HT-	I	N-----A---------
	Pseudomonas furukawaii	WP_231992300	AQ--LT-A-------T-	I	T--------N------
	Pseudomonas koreensis	WP_110646348	-H--MTL---G-N--T-	A	S-----A---------
	Pseudomonas mediterranea	WP_230165137	-Q--LTL---G----T-	V	S-----A---------
	Pseudomonas reactans	WP_177002403	-Q---TL---G---HT-	I	T-----A---------
	Pseudomonas savastanoi	WP_192485492	----LTL-------AT-	I	YH----A---------
	Pseudomonas simiae	WP_021491887	-Q---TL---G---HT-	V	T-----A---------
	Pseudomonas sivasensis	WP_181642841	-Q---TL---G---HT-	I	T-----A---------
	Pseudomonas syringae	WP_080397465	----LTL-------TT-	S	YH----A---------
	Pseudomonas trivialis	WP_049709991	-H---TL---G---HT-	I	T-----A---------
	Pseudomonas veronii	WP_232591266	-Q--LT----G---QT-	A	T-----A---------
	Pseudomonas viridiflava	WP_122764738	-Q---TL---G---HT-	I	T-----A---------

Figure- S71

Partial sequence alignments of a protein TIGR02099 family protein showing a 1aa Del (highlighted), which is uniquely shared by all species from the Thermotolerans clade.

Group	Species	Accession	359		390
**Thermotolerans Clade (*Zestomonas* gen. nov.) (4/4)**	*Pseudomonas carbonaria*	WP_187671317	AIAEQIGKPFITTKG	G	KGFGLGLFLSQASVTR
	Pseudomonas cavernae	WP_119894923	---------------	-	----------------
	Pseudomonas thermotolerans	WP_017938652	-----L---------	-	----------------
	Pseudomonas insulae	WP_205348721	---------------	-	----------------
**Other *Pseudomonas***	*Pseudomonas aeruginosa*	WP_128447160	---G------F----		----------------
	Pseudomonas alcaligenes	WP_187807652	----------F----		----------------
	Pseudomonas azotifigens	WP_181068768	-------R--F----		----------------
	Pseudomonas campi	WP_173203580	----------F----		----------------
	Pseudomonas coronafaciens	KGS12209	----------F----		----------K-----
	Pseudomonas fuscovaginae	WP_017904782	----------F----		----------K-----
	Pseudomonas guryensis	WP_182834309	----------F----		----------------
	Pseudomonas mangrovi	WP_108104950	-----L----F----		----------------
	Pseudomonas nitrititolerans	HJE30261	-------R-------		----------------
	Pseudomonas nosocomialis	WP_138407392	-------R--F----		----------------
	Pseudomonas saudiphocaensis	WP_037023576	-------R-------		----------------
	Pseudomonas savastanoi pv. fra	KUG45008	----------F----		----------K-----
	Pseudomonas stutzeri	WP_143514357	-------R-------		----------------
	Pseudomonas syringae pv. macul	RML93385	----------F----		----------K-----
	Pseudomonas ullengensis	WP_183089014	----------F----		----------------
	Pseudomonas viridiflava	WP_122452382	-----L---------		----------------
	Pseudomonas zhaodongensis	WP_122168213	-------R-------		----------------
**Other Bacteria**	*Klebsiella pneumoniae*	SVJ79186	----------F----		----------------
	Stenotrophomonas maltophilia	WP_239520025	----------F----		----------------

Figure- S72

Partial sequence alignments of a protein HAMP domain-containing histidine kinase showing a 1aa Ins (highlighted), which is uniquely shared by all species from the Thermotolerans clade.

Group	Species	Accession	47		87
**Thermotolerans Clade (*Zestomonas* gen. nov.) (4/4)**	*Pseudomonas cavernae*	WP_119895222	YDLKGDQASRTDEFREGIGRLW		QAETPALAGDYLRVVRELN
	Pseudomonas insulae	WP_205348360	---L-P-------W-D--A---		-G-A----D---A-LQA--
	Pseudomonas carbonaria	WP_187670420	---L-E-------W--------		-S-A----S---KAIMA--
	Pseudomonas thermotolerans	WP_017937049	---L-E-------WQD------		AS-A--------KAILA--
**Other *Pseudomonas***	*Pseudomonas agarici*	WP_017130110	-----------G-YL-------	G	EQDL--VTA-----LH-M-
	Pseudomonas alliivorans	WP_210073405	--------T--G-WL-------	E	AEDV----A---Q-L-DM-
	Pseudomonas amygdali	WP_044320072	--------T--G-WL-------	D	APDL----S---Q-LHDM-
	Pseudomonas amygdali pv. eriob	RMM03743	--------T--G-WL-------	D	APDL----S---Q-LHDM-
	Pseudomonas amygdali pv. lachr	RMP47573	--------T--G-WL-------	D	APDL----S---Q-LHDM-
	Pseudomonas amygdali pv. mori	KPX88372	--------T--G-WL-------	D	APDL----S---Q-LHDM-
	Pseudomonas avellanae	WP_148283658	--------T--G-WL-------	N	TPDL----A--MQ-LHDM-
	Pseudomonas balearica	WP_165562620	---A-------G-WLQ--A-I-	Q	AETR---LD---G-I-Q--
	Pseudomonas cannabina	WP_057414617	--------T--G-WL--V----	N	TEDL----A---Q-LHDM-
	Pseudomonas cavernicola	WP_119953497	-----------G-WL---N-M-	E	A-DV-E--A-----L----
	Pseudomonas cichorii	RMR51806	--------T--G-WL-------	N	APDL----A---Q-LHDM-
	Pseudomonas congelans	WP_032612345	--------T--G-WL-------	N	ATDL----S---Q-LHDM-
	Pseudomonas coronafaciens	WP_147477657	-----------G-WL-------	D	TPDL---TA---Q-LHDM-
	Pseudomonas floridensis	WP_083184890	--------T--G-WL-------	D	AQDL----A---Q-LHDM-
	Pseudomonas fluorescens	WP_150582998	---Q-------G-YL-------	D	-PDL---TA--MK-LHDM-
	Pseudomonas fuscovaginae	WP_054060246	-----------G-YL---A---	G	-QDL--MTA-----LH-M-
	Pseudomonas gingeri	WP_177090616	-----------G-YL---A---	G	EQ-L--VTA--M--LH-M-
	Pseudomonas japonica	WP_181119085	---Q-------G-YL--VT---	A	VENP-E--A-----L-K--
	Pseudomonas jessenii	WP_115145969	--------N--G-YL---A---	D	-PDL---TA--MK-LH-M-
	Pseudomonas kirkiae	WP_131182822	---A-------G-WSQ------	Q	AEGM--VFA---G-I-Q--
	Pseudomonas lalkuanensis	WP_151137737	---L---------W--------	D	LPDL--PMA---K------
	Pseudomonas massiliensis	WP_040259613	---H-------G-YL--VS---	G	AQ-V-E--A----IL-K--
	Pseudomonas putida	WP_196188913	---Q----T--G-YL--VA---	N	HDGL--M------IIKR--
	Pseudomonas resinovorans	WP_077524769	---L---------W--------	D	LPDL--PMA-----IH---
	Pseudomonas savastanoi	RMV11162	--------T--G-WL-------	D	APDL----S---Q-LHDM-
	Pseudomonas stutzeri	WP_077682206	---A-------G-WLQ--A-I-	Q	AETR---LD---G-I----
	Pseudomonas syringae	WP_236450218	--------T--G-WL-------	N	ATDL----S---Q-LHDM-
	Pseudomonas taiwanensis	WP_179062486	---L---------W--------	D	LPDL--PMA-----IH---
	Pseudomonas vanderleydeniana	WP_186683264	-----------G-YL---A---	A	EQDL--VTA-----LH-M-
	Pseudomonas viridiflava	WP_122593266	--------T--G-WL-------	E	A-DV----A---Q-L-DM-

Figure- S73

Partial sequence alignments of 23S rRNA (adenine(2030)-N(6))- methyltransferase RlmJ protein showing a 1aa Del (highlighted), which is uniquely shared by all species from the Thermotolerans clade.

Group	Species	Accession	261		299
**Thermotolerans Clade (*Zestomonas* gen. nov.) (4/4)**	*Pseudomonas cavernae*	WP_119895183	CWSFAAAALSESRRY	G	PQTFGMAEGLALDAEGAWIGLDN
	Pseudomonas insulae	WP_205348399	------E--Q-A---	-	S-AY------S--EK-------S
	Pseudomonas carbonaria	WP_187669890	-----GE--Q-P---	-	M----L----VI-EK--------
	Pseudomonas thermotolerans	WP_017939434	-----EE--Q-P---	-	R-A--R----E--EQ-------T
**Other *Pseudomonas***	*Pseudomonas alkylphenolica*	WP_128321568	------D--T-----		A-P--L--A-VI--K-----V--
	Pseudomonas alloputida	WP_232862449	------D--V-----		A-P--L--A-VI--K--------
	Pseudomonas asplenii	WP_090202687	------E--T-P---		--NY-L--A-VV--D--------
	Pseudomonas brassicacearum	WP_123345125	------E--QDN---		--AY-L--A-VV-----------
	Pseudomonas chlororaphis	WP_123329522	------E--Q-Q---		--PY-L--A-VV--T--------
	Pseudomonas defluvii	WP_065758736	------E--T-P---		S-P--L--A-VI------V----
	Pseudomonas ekonensis	WP_217893914	-----ED--Q-K---		--PY-L--A-VI--Q--------
	Pseudomonas fluorescens	WP_019691487	------E--Q-H---		--PY----A-VV--D-----I--
	Pseudomonas frederiksbergensis	WP_071552149	------E--Q-H---		--PY-L--A-VV--------I--
	Pseudomonas fulva	WP_110606946	------D--T-P---		D-L--V--A-VV-----------
	Pseudomonas fuscovaginae	WP_010450436	------E--T-P---		--SY-L--A-VV--D--------
	Pseudomonas izuensis	WP_160105210	-----DE--Q-N---		--P--L--A-VV--------V--
	Pseudomonas japonica	WP_042120325	------E--VD----		G-PY-L--A-VI--D--------
	Pseudomonas laurentiana	WP_189395462	------E--T-P---		S-P--L--A-VI------V-V--
	Pseudomonas monteilii	WP_016712339	------D--V-----		A-P--L--A-VI--K--------
	Pseudomonas ogarae	WP_079301634	------E--QDN---		--PY-L--A-VV-----------
	Pseudomonas parafulva	WP_058603995	------D--T-P---		D-L--V--A-VV-----------
	Pseudomonas piscis	WP_152899101	------E--V-----		--PY-L--A-VV-----------
	Pseudomonas protegens	WP_047283673	-----GE--V-H---		--PY-L--A-VV--K--------
	Pseudomonas putida	BAW25965	------D--A-A---		--P--L--A-VI--K--------
	Pseudomonas tructae	WP_130262825	------D--A-P---		S-P--L--A-VI--K--------
	Pseudomonas vanderleydeniana	WP_186683334	------E--T-P---		--NY-L--A-VV--D-V------
	Pseudomonas viciae	WP_135843404	------E--QDN---		--AY-L--A-VV-----------

Figure- S74

Partial sequence alignments of Esterase-like activity of phytase family protein showing a 1aa Ins (highlighted), which is uniquely shared by all species from the Thermotolerans clade. One *Pseudomonas* strain UL073 also shares this CSI.

			464		500
**Flexibilis clade (Genus *Serpens* emend.) (3/3)**	*Pseudomonas flexibilis*	WP_039562945	YSLQTGEQVEIITTKQG	G	APSRDWLNPNLGYITTSRAR
	Pseudomonas tuomuerensis	WP_027590415	-----------------	-	--------------------
	Serpens gallinarum	WP_251837344	-T----------ASK--	-	-------------V----S-
**Other *Pseudomonas***	*Pseudomonas alcaligenes*	MBB4817224	-------------G---		S-------------------
	Pseudomonas atacamensis	WP_223631159	-------------S-H-		T------------V------
	Pseudomonas atagonensis	WP_166218381	-------------S-H-		T------------V------
	Pseudomonas azerbaijanoccidens	WP_217873631	-------------S-H-		T------------V------
	Pseudomonas azotifigens	WP_028241835	-------------S---		S-------------------
	Pseudomonas azotoformans	RMT65298	-------------S-H-		T-------------------
	Pseudomonas balearica	WP_061340971	-------------S---		S-----------------S-
	Pseudomonas bananamidigenes	WP_065261670	-------------S-H-		T------------V------
	Pseudomonas brassicacearum	WP_214509574	-------------S-H-		T-------------------
	Pseudomonas chlororaphis	WP_124321864	-------------S-H-		T------------V------
	Pseudomonas composti	WP_061239955	-------------S---		S------------V------
	Pseudomonas corrugata	WP_053192435	-------------S-H-		T-------------------
	Pseudomonas costantinii	WP_071482952	-------------S-H-		T-------------------
	Pseudomonas deceptionensis	WP_048358885	-------------S-H-		T-------------------
	Pseudomonas fluorescens	WP_078803584	-------------S-H-		T-------------------
	Pseudomonas fragi	WP_095037602	-------------S-H-		T------------V------
	Pseudomonas frederiksbergensis	WP_105342822	-------------S-H-		T------------V------
	Pseudomonas gozinkensis	WP_192562048	-------------S-H-		T------------V------
	Pseudomonas guguanensis	WP_090425818	-------------S---		S------------V------
	Pseudomonas indoloxydans	WP_108233465	-------------S---		T------------V------
	Pseudomonas izuensis	WP_160106599	-------------S-H-		T------------V------
	Pseudomonas khazarica	WP_210390875	-------------S---		S------------V------
	Pseudomonas khorasanensis	WP_186529704	-------------S-H-		T------------V------
	Pseudomonas kielensis	WP_185818751	-------------S-H-		T-------------------
	Pseudomonas koreensis	WP_198759537	-------------S-H-		T------------V------
	Pseudomonas kunmingensis	WP_234175041	-------------S-H-		S-----------------S-
	Pseudomonas lopnurensis	WP_193680809	-------------S-H-		S-----------------S-
	Pseudomonas mangiferae	WP_143487674	-------------G---		--------------------
	Pseudomonas mendocina	WP_003247032	-------------S---		S------------V------
	Pseudomonas moorei	WP_090324501	-------------S-H-		T------------V------
	Pseudomonas moraviensis	NYH10498	-------------S-H-		T------------V------
	Pseudomonas nitrititolerans	WP_170910706	-------------S---		S-----------------S-
	Pseudomonas nosocomialis	WP_138409635	-------------S---		S-------------------
	Pseudomonas oleovorans	WP_104729628	-------------S---		-------------V------
	Pseudomonas oryzae	WP_090349314	-------------G-H-		-------------V------
	Pseudomonas otitidis	WP_165670149	-------------G---		--------------------
	Pseudomonas protegens	WP_207166381	-------------S-H-		T------------V------
	Pseudomonas psychrophila	WP_019827736	-------------S-H-		T-------------------
	Pseudomonas putida	WP_110964449	-------------S-H-		T------------V------
	Pseudomonas resinovorans	WP_016494320	-------------G-H-		--------------------
	Pseudomonas savastanoi	WP_147463181	-------------S-H-		T-------S-----------
	Pseudomonas stutzeri	WP_148926379	-------------S---		S-----------------S-
	Pseudomonas stutzeri	WP_181093921	-------------S---		S-----------------S-
	Pseudomonas syringae	KEZ71396	-------------S-H-		T-------S-----------
	Pseudomonas tolaasii	WP_016973364	-------------S-H-		T-------------------
	Pseudomonas urumqiensis	WP_120997785	-------------S---		S-------------------
	Pseudomonas vancouverensis	WP_093215436	-------------S-H-		T------------V------
	Pseudomonas veronii	WP_046385202	-------------S-H-		T-------------------
	Pseudomonas wenzhouensis	WP_230924830	-------------S---		T------------V------
	Pseudomonas xanthomarina	WP_041013657	-------------S-H-		S-----------------S-
	Pseudomonas xanthomarina	WP_065984831	-------------S---		S-----------------S-
**Other Bacteria**	*Acinetobacter pittii*	WP_163120517	-------------G-HS		G-------S----V------
	Azomonas agilis	WP_144572099	----------V--S---		T------------V------
	Streptococcus pneumoniae	CJL07450	-------------S-H-		S-----------------S-

Figure- S75

Partial sequence alignments of a protein GTP diphosphokinase showing a 1aa Ins (highlighted), which is specific for all three species from flexibilis Clade.

Group	Organism	Accession	85		120
Flexibilis clade (Genus *Serpens* emend.) (3/3)	*Pseudomonas flexibilis*	WP_039607122	LGMLAPTMLALGLVTMSLL		EVRVDLLGYLFGDLLA
	Pseudomonas tuomuerensis	WP_027588704	-------------------		----------------
	Serpens gallinarum	WP_251835472	--I--HST-S----AI-FM		---I--MA--------
Other *Pseudomonas*	*Pseudomonas abietaniphila*	WP_062386410	--I---ST-S----VL-FM	R	---I--M---------
	Pseudomonas akappageensis	WP_166364633	--I---ST-S----VL-FM	H	D--I--M---------
	Pseudomonas alcaligenes	WP_110680530	--I----T-S----VL-FM	D	------MA--------
	Pseudomonas alliivorans	WP_210070489	--I---ST-S----VL-FM	H	---I--MA--------
	Pseudomonas amygdali	WP_044323856	--I---ST-S----VL-FM	H	---I--MA--------
	Pseudomonas baltica	WP_185794586	--I---ST-S----VL-FM	H	D--I--M---------
	Pseudomonas batumici	WP_040062884	--I---ST-S----AL-FM	H	---I--M---------
	Pseudomonas bohemica	WP_110951753	--I---ST-S----VL-FM	R	---I--M---------
	Pseudomonas bubulae	WP_235571909	--I---ST-S----VL-FM	H	---I--MA--------
	Pseudomonas campi	WP_173210644	--I----T-S----VL-FM	D	---I--MA--------
	Pseudomonas capsici	WP_206402968	--I---ST-S----VL-FM	H	---I--MA--------
	Pseudomonas caricapapayae	WP_122339308	--I---ST-S----VL-FM	H	---I--MA--------
	Pseudomonas cavernae	WP_119891563	--I----T-S----VL-FM	Q	D--I--M---------
	Pseudomonas cavernicola	WP_119952981	--I----T-S----VL-FM	R	D--I--M---------
	Pseudomonas cichorii	WP_122314881	--I---ST-S----VL-FM	H	D--I--M---------
	Pseudomonas coleopterorum	WP_192000435	--I---ST-S----VL-FM	H	D--I--M---------
	Pseudomonas congelans	WP_096103174	--I---ST-S----VL-FM	H	---I--MA--------
	Pseudomonas coronafaciens	WP_122311271	--I---ST-S----VL-FM	H	---I--MA--------
	Pseudomonas costantinii	WP_177015206	--I---ST-S----VL-FM	H	---I--MA--------
	Pseudomonas cremoricolorata	WP_028695809	--I---ST-S----VL-FM	H	D--I--M---------
	Pseudomonas entomophila	WP_181095160	--I---ST-S-----L--M	H	D--I--MA--------
	Pseudomonas extremaustralis	WP_010566710	--I---ST-S----VL-FM	H	---I--MA--------
	Pseudomonas floridensis	WP_083181114	--I---ST-S----VL-FM	H	---I--MA--------
	Pseudomonas fluorescens	WP_232545307	--I---ST-S----VL-FM	H	---I--MA--------
	Pseudomonas fragi	WP_095027678	--I---ST-S----VL-FM	H	---I--MA--------
	Pseudomonas frederiksbergensis	WP_205888934	--I---ST-S----VL-FM	H	---I--MA--------
	Pseudomonas gingeri	WP_177093780	--I---ST-S----VL-FM	H	---I--M---------
	Pseudomonas graminis	WP_065987954	--I---ST-S----VL-FM	R	D--I--M---------
	Pseudomonas guryensis	WP_182832663	--I----T-S----VL-FM	D	---I--MA--------
	Pseudomonas japonica	WP_181111241	--I---ST-S----AL-FM	R	D--I--M---------
	Pseudomonas karstica	WP_154741526	--I---ST-S----VL-FM	H	---I--MA--------
	Pseudomonas kirkiae	WP_131184587	--I--HST-S-----L-FM	H	---I--M---------
	Pseudomonas libanensis	WP_057011609	--I---ST-S----VL-FM	H	---I--MA--------
	Pseudomonas linyingensis	WP_090313354	--I--HST-S-----I---	D	------MA--------
	Pseudomonas lundensis	WP_047296324	--I---ST-S----VL-FM	H	---I--MA--------
	Pseudomonas lutea	WP_037017072	--I---ST-S----VL-FM	R	D--I--M---------
	Pseudomonas marginalis	WP_058413895	--I---ST-S----VL-FM	H	---I--MA--------
	Pseudomonas monteilii	AMA47679	--I---ST-S-----L-FM	H	D--I--MA--------
	Pseudomonas mucidolens	WP_084378441	--I---ST-S----VL-FM	H	---I--MA--------
	Pseudomonas plecoglossicida	WP_016394243	--I---ST-S----VL-FM	K	D--I--M---------
	Pseudomonas pohangensis	WP_090198127	--I----T------LL-FM	S	N--I--M---------
	Pseudomonas protegens	WP_210696018	--I---ST-S----VL-FM	H	---I--MA--------
	Pseudomonas putida	WP_192237293	--I---ST-S----VL-FM	R	D--I--M---------
	Pseudomonas rhizosphaerae	WP_043191926	--I---ST-S----VL-FM	R	D--I--M---------
	Pseudomonas sagittaria	WP_092434564	--I--HST-S-----I---	D	------MA--------
	Pseudomonas savastanoi	RMT25920	--I---ST-S----VL-FM	H	---I--MA--------
	Pseudomonas synxantha	WP_046070274	--I---ST-S----VL-FM	H	---I--MA--------
	Pseudomonas syringae	WP_236451284	--I---ST-S----VL-FM	H	---I--MA--------
	Pseudomonas taetrolens	WP_048381290	--I---ST-S----VL-FM	H	---I--MA--------
	Pseudomonas thermotolerans	WP_027896354	--I----T-S----AL-FM	S	---I--MA--------
	Pseudomonas ullengensis	WP_183087730	--I----T-S----VL-FM	D	N--I--M---------
	Pseudomonas viridiflava	WP_122449945	--I----T-S----VL-FM	H	D--I--M---------
	Pseudomonas viridiflava	WP_122850134	--I---ST-S----VL-FM	H	---I--MA--------
	Pseudomonas weihenstephanensis	WP_048363901	--I---ST-S----VL-FM	H	---I--MA--------
Other Bacteria	*Azotobacter beijerinckii*	WP_090619225	--I---ST-S----VL-FM	R	D--I--M---------
	Denitrificimonas caeni	WP_205342030	--I--HST-S-----L-FM	E	S-----M---------
	Ensete ventricosum	RWW90831	--I---ST-S----VL-FM	H	---I--MA--------
	Salmonella enterica subsp. ent	MCC1687564	--I---ST-S----VL-FM	H	---I--MA--------

Figure- S76

Partial sequence alignments of a protein zinc ABC transporter permease subunit ZnuB showing a 1aa Del (highlighted), which is specific for the species from Flexibilis clade.

Group	Species	Accession	433		469
**Flexibilis clade (Genus *Serpens* emend.) (3/3)**	*Pseudomonas flexibilis*	WP_039560866	YRLFGFFATRLRSLTP		NIGPWAQNHAAPKPAARSLH
	Pseudomonas tuomuerensis	WP_039606419	----------F-G---		--------------------
	Serpens gallinarum	WP_251834978	--V---------G---		---------S----------
**Other *Pseudomonas***	*Pseudomonas alliivorans*	WP_210042694	------------G---	G	-V---T---S----------
	Pseudomonas arsenicoxydans	WP_140663986	------------A---	S	-----T---S----------
	Pseudomonas asturiensis	WP_073172277	------------G---	G	-----T---S----------
	Pseudomonas balearica	MBD3735772	------------A---	S	-----T---S----------
	Pseudomonas chloritidismutans	WP_228208263	--V---------G---	S	-----T---S----------
	Pseudomonas chlororaphis	WP_038357471	------------A---	N	-V---T---S----------
	Pseudomonas crudilactis	WP_180698275	------------A---	S	-V---T---S----------
	Pseudomonas farris	WP_217857591	------------A---	S	-V---T---S----------
	Pseudomonas fluorescens	WP_154972589	----------------	G	-V---T---S----------
	Pseudomonas frederiksbergensis	WP_205890914	------------A---	S	-V---T---S----------
	Pseudomonas granadensis	WP_203420688	------------A---	K	-V---T---S----------
	Pseudomonas indica	MBU3058338	--T-S-------G---	S	-----T---S----------
	Pseudomonas izuensis	WP_160109203	------------A---	K	-V---T---S----------
	Pseudomonas jessenii	WP_110718993	------------A---	S	-V---T---S----------
	Pseudomonas kunmingensis	WP_104096817	--V---------G---	S	-----T---S----------
	Pseudomonas laurylsulfativoran	WP_103394080	------------A---	S	-V---T---S----------
	Pseudomonas lopnurensis	WP_193679495	------------A---	S	-V---T---S----------
	Pseudomonas mandelii	WP_019582461	------------A---	S	-V---T---S----------
	Pseudomonas marginalis	KTC12198	----------------	G	-V---T---S----------
	Pseudomonas migulae	WP_182343478	------------A---	N	-V---T---S----------
	Pseudomonas mohnii	WP_196585161	------------A---	N	-V---T---S----------
	Pseudomonas saudiphocaensis	WP_125839736	--V-S-------A---	S	-V---T---S----------
	Pseudomonas songnenensis	WP_122098108	--V---------G---	S	-----T---S----------
	Pseudomonas stutzeri	WP_003288742	------------A---	S	-----T---S----------
	Pseudomonas syringae	WP_192062701	----------------	G	-V---T---S----------
	Pseudomonas viridiflava	WP_122857617	----------------	G	-V---T---S----------
	Pseudomonas xanthomarina	WP_125875450	--V---------G---	S	-----T---S----------
**Other Bacteria**	*Streptococcus pneumoniae*	CJL34494	--V---------G---	S	-L---T---S----------

Figure-S77

Partial sequence alignments of LutB/LldF family L-lactate oxidation iron-sulfur protein showing a 1aa Del (highlighted), which is specific for the species for the species from Flexibilis clade.

Group	Species	Accession	226		267
**Fluvialis Clade (*Caenipseudomonas* gen. nov.) (2/2)**	*Pseudomonas pharmacofabricae*	WP_101192990	AFNTMLERMANRERELQEA		HAQTRQQSEAHERSNRKLALEV
	Pseudomonas fluvialis	WP_093985177	-------------------		----------------------
**Other *Pseudomonas***	*Pseudomonas aeruginosa*	WP_132503449	------T-IEA--QQ-KR-	RDDAQEA	VE-AQSXA-ETR------E---
	Pseudomonas argentinensis	WP_074881278	--------IEA--QQ-KR-	RDDSEEA	YN-A-GLA-ETRL-----E---
	Pseudomonas bohemica	WP_110948530	------S--EA--QQ-KR-	RDDSQEA	YD-AQGLA-ETRHT----E---
	Pseudomonas cichorii	WP_122317014	------S-IEA--QQ-KR-	RDDSQEA	Y--AQGMA-ETRHT----E---
	Pseudomonas citronellolis	SFC21545	------T-IEA--QQ-KR-	RDDAQDA	M--AQDLA-ETR------E---
	Pseudomonas delhiensis	WP_089390707	------T-IEA--QQ-KR-	RDDAQDA	M--AQDLA-ETR------E---
	Pseudomonas fluorescens	WP_126450720	------T-IEA--QQ-KR-	RDDAQEA	VE-AQSLA-ETR------E---
	Pseudomonas graminis	HEF25547	------S--EA--QQ-RR-	RDDSQEA	YD-AQGLA-ETRHT----E---
	Pseudomonas jinjuensis	SDO98821	------T-IEA--QQ-KR-	RDDAQEA	VE-AQSLA-ETR------E---
	Pseudomonas knackmussii	WP_160288155	------T-IEA--QQ-KR-	RDDAQEA	V--AQGLA-ETR------E---
	Pseudomonas lutea	WP_037014650	------S--QA--QQ-KR-	RDESQEA	YD-AQGLA-ETRHT----E---
	Pseudomonas nicosulfuronedens	WP_138521415	------T-IEA--QQ-KR-	RDDAQEA	VE-AQSLA-ETR------E---
	Pseudomonas nitritireducens	MBB4863590	------T-IEA--QQ-KR-	RDDAQEA	VE-AQSLA-ETR------E---
	Pseudomonas ovata	WP_109511370	------A-IEA--QQ-KR-	RDDSQDA	YD-A-GLA-ETR-T----E---
	Pseudomonas punonensis	WP_073261909	--------IEA--QQ-KR-	RDDSEEA	YN-A-GLA-ETRL-----E---
	Pseudomonas schmalbachii	WP_208316184	------T-IEA--QQ-KR-	RDDAQEA	VE-AQSLA-ETR------E---
	Pseudomonas stutzeri	WP_003288545	------S--QA--QQ-KR-	RDDAQEA	FD-A-GLA-ETRT---R-E---
	Pseudomonas viridiflava	WP_122673063	------S-IEA--QQ-KR-	RDDSQEA	YD-AQGLA-ETRHT----E---
**Other Bacteria**	*Tanacetum cinerariifolium*	GEY60321	------S--QA--QQ-KR-	RDESQEA	YD-AQGLA-ETRHT----E---

Figure- S78

Partial sequence alignments of ATP-binding protein showing a 7aa Del (highlighted), which is specific for the Fluvialis clade but not present in other *Pseudomonas* species or other bacterial species.

			146				197
**Fluvialis Clade (*Caenipseudomonas* gen. nov.) (2/2)**	*Pseudomonas pharmacofabricae*	WP_101193738	LIGESFANQLD		ALLDEVLPGVLAGAFSAASEPGTAQVES		VWRQWPCLLQQLG
	Pseudomonas fluvialis	WP_093986559	-----------		----------------------------		-------------
**Other *Pseudomonas***	*Pseudomonas aeruginosa*	HBN8232177	----P------	E	-VRHALEHMLD-DT-D-E-ARAYRENLK	PRVVA	A-Q-----SR---
	Pseudomonas alcaligenes	MBB4817959	----P------	E	-VHHA-DGMLDETV-ER--VQHYRDRLH	PKVEA	A-KR----SR---
	Pseudomonas alcaliphila	WP_074677570	----P------	E	-VHHALAGMLDDAS-DQP-VQAYRERLA	PDTAS	A--R----S----
	Pseudomonas anguilliseptica	WP_233685674	----P------	E	-VHHA-DGMLDDAS-AQD-VQAYRERLH	PQTEA	A-QR----SR---
	Pseudomonas benzenivorans	WP_090446040	----P------	E	-VHHA-DGMLDEAS-GQG-VLAYRERL-	PHTEA	A-KR----SR---
	Pseudomonas borbori	WP_090500824	----P------	E	-VHHA-DGMLDEAS-DQ--VRAYRERL-	PQTEA	A-KR----SG---
	Pseudomonas carbonaria	WP_187670054	----P------	E	-VHHA-EGMLPED---QE-VQAYR-RL-	PQIEA	A-KR----S----
	Pseudomonas cavernae	WP_119891548	----P------	E	-VHHA-DGLLDEA--AQD-VQAYR-RL-	PQTEA	A-KR----SRR--
	Pseudomonas chengduensis	WP_196447350	----P------	E	-VHHALTGMLDDAS-DQP-VQAYRERLA	PDTAS	A-QR----S----
	Pseudomonas cichorii	WP_221607825	----E------	E	-VHHA-DQLLNEES-ALE-VETYRDRLG	ADTEA	A-KR----SR---
	Pseudomonas citronellolis	KAF1070714	----P------	D	-VHRA-EGLLASDT-DTP-AQAYRARL-	PQTVT	A-------SRRF-
	Pseudomonas composti	WP_037002213	----P------	E	-VHHALDGMLDDAS-DQP-VQAYRERLA	PDTVT	A--R----S----
	Pseudomonas guguanensis	WP_090431436	----P------	E	SVHHALAGMLDDAS-DQP-VQAYRERLA	PDTAS	A-QR----S----
	Pseudomonas indica	WP_084338990	----P------	E	-VHHA-DNLLDDTT-AQ--VQAYRERLN	PQTEA	A--R----SR---
	Pseudomonas jinjuensis	WP_084311455	----P------	T	SVQQALGGMIHD-L-ASPAAQAYRASL-	PQVAN	A------MSR---
	Pseudomonas khazarica	WP_134677556	----P------	E	-VHHALAGMLDDSS-DQP-VQAYRERLA	PNTAS	A-QR----S----
	Pseudomonas lalkuanensis	WP_151131113	----P------	E	-VHHA-DGLLDDQV--QP-AQAYR-QLH	PKVEE	A-KR---MS----
	Pseudomonas mendocina	WP_143501110	----P------	E	-VHHALAGMLDDAS-DQS-VQAYRERLA	PDTAS	A--R----S----
	Pseudomonas multiresinivorans	WP_169935225	----P------	E	-VHHA-NGMLDDST---E-AVSYRASLN	PQVVM	A-Q-----SR-F-
	Pseudomonas nicosulfuronedens	WP_138519949	----P------	E	-VHHA-NGMLDDST---E-AVSYRARLN	PQVVM	A-Q-----SR-F-
	Pseudomonas nitroreducens	WP_017517730	----P------	E	-VHHA-DGMLDDST---E-AVSYRASLN	PQVVM	A-Q-----SR-F-
	Pseudomonas oleovorans	WP_119692480	----P------	E	-VHHALSGMLDDAS-DQP-VQAYRERLA	PDTAS	A--R----S----
	Pseudomonas oryzae	WP_090350782	----N------	A	-VHHASEALLDP-I-T-E-AETYRAKLA	PKLLA	A-------SRR--
	Pseudomonas otitidis	WP_236211186	----P------	E	-VHLA-DGMLDP-V-QRE-VQQYRARLH	PKVEA	A-KR----SR---
	Pseudomonas peli	WP_090254376	----A------	E	-VHHA-DGMLDEAS-AHD-VQAYRERLN	PQTDA	A-KR----SR---
	Pseudomonas resinovorans	WP_016489993	----P------	E	-VHHA-DGLLEEEV--QP-AQAYR-QLH	PKVEA	A-KR----S----
	Pseudomonas schmalbachii	WP_208313837	----P------	D	-VHQA-GVMLQDSL-AKV-AQAYRARLN	PQVAS	A------MSR---
	Pseudomonas sediminis	WP_179543381	----P------	E	-VHHALDGMLDDAN-DQP-VQAYRERLA	PDTAS	A--R----S----
	Pseudomonas sihuiensis	WP_092379137	----P------	E	-VHHALTGMLDDAS-DQP-VQAYRERLA	PDTAS	A-QR----S----
	Pseudomonas taeanensis	WP_025163654	----P------	E	-VHHA-DGMLDEAS-AQD-VVAYRKRL-	PQTEA	A--R----SG---
	Pseudomonas taiwanensis	WP_179061506	----P------	E	-VHHA-DGLLDEDV--QP-AQAYR-QLH	PKVEE	A-KR---MSE---
	Pseudomonas tohonis	WP_173174727	----P------	E	-VHHA-DGMLDEAV-ER--VQHYRDRLH	PKVEA	A-KR----SR---
	Pseudomonas toyotomiensis	WP_045733641	----P------	E	-VHHALTGMLDDAS-DQP-VQAYRERLA	PDTAS	A-QR----S----
	Pseudomonas wenzhouensis	WP_230926158	----P------	E	-VHHALAGMLDDAS-DQS-VQAYRERLA	PDTAS	A-QR----S----
	Pseudomonas xionganensis	WP_160343177	----P------	E	-VHHA-DGMLDEQS-AQ--VAAYRERL-	PQTEA	A--R----S-R--
	Pseudomonas yangonensis	WP_161864145	----P------	E	-VHHALAGMLDDAS-DQP-VQAYRERLA	PDTAS	A--R----S----
	Pseudomonas nitritireducens	WP_184586154	----P------	E	-VHHA-NGMLDDST---E-AISYRASLN	PQVVM	A-Q-----SR-F-
**Other Bacteria**	*Halopseudomonas aestusnigri*	WP_233048717	-M--G------	T	-VETA-SGLLPEDL-QSEPFRAYQASQD	AQVRR	L-T----MSR---
	Halopseudomonas pachastrellae	WP_083725629	-L--P---R--	A	-VEEA-AGLLPEDL-QS-EFQAYRAQLD	ASVQR	L-A----MS----
	Halopseudomonas salegens	WP_092389258	-L--P------	S	-VEEA-TGLLP-SL-ASEEFQQHLNQLD	SQRQQ	L-D-----SSR--

Figure- S79

Partial sequence alignments of a hypothetical protein showing a 1aa and 5aa Del (highlighted), which are specific for Fluvialis clade but not present in other *Pseudomonas* species.

Group	Species	Accession	415		452
**Fluvialis Clade (*Caenipseudomonas* gen. nov.) (2/2)**	*Pseudomonas pharmacofabricae*	WP_101193981	ALLRELASYATASCVWLLPGE		DPQRLLDWQQALQAAGL
	Pseudomonas fluvialis	WP_093984868	---------------------		-----------------
**Other *Pseudomonas***	*Pseudomonas allii*	WP_179029758	--IA---RS---TR----QAP	PGQAL	-AE--G--HA---QLQ-
	Pseudomonas alliivorans	MBP0946779	--IA---RC-G-TR-----AP	AGQTL	--E--D--HV--DQLQ-
	Pseudomonas asturiensis	WP_024684725	--IA---RC-G-TR-----AP	AGQAL	--E--D--HV--DQLQ-
	Pseudomonas baltica	MBC2679754	G-IA---RC--VTR-----AP	PGQAL	----HG---AG-D-L--
	Pseudomonas bohemica	WP_110949412	--IA---RC---TRI----AP	VGEAL	-AN--S--HS---QLE-
	Pseudomonas brenneri	WP_229752144	--IG---RC-S-TR----QAP	PGQAL	-AE--G--HT---QL--
	Pseudomonas capsici	MBN6713681	--IA---R--GKTRI----AP	AGQTL	-----N--HV---QLE-
	Pseudomonas caspiana	WP_087268436	--VA---RC-G-TR-----AP	DGQHL	N-E-VG---A---QLD-
	Pseudomonas chlororaphis	WP_198919486	--IA---RS---TR----QAP	PGQAL	-AE--G---V---QLE-
	Pseudomonas cichorii	WP_201018897	--IA---R--SQTRI----AP	AGQTL	-----N--HA---QLE-
	Pseudomonas coleopterorum	MBD8756322	G-IA-M-RC--VTR-----AP	PGQVL	-----G--RTG-D-L--
	Pseudomonas cremoricolorata	WP_038411685	---G---RN-G-TRI----AA	PGEQL	-AA--G--HE--TRL--
	Pseudomonas deceptionensis	WP_048361290	--IG---RS-SETR----QAP	PGQAL	-AD--G--HT---QL--
	Pseudomonas entomophila	MBA1194192	---A---RT-GDTRI----AA	PDLPV	--A--D--H---EQL--
	Pseudomonas extremaustralis	WP_078834389	--IA---RS---TR----QAP	PGQAL	-AE--G--HT---QLD-
	Pseudomonas fluorescens	WP_232626473	--IA---RS---TR----QAP	PGQAL	-AE--G--HA---QLE-
	Pseudomonas fluorescens	WP_150653956	--IA---RN-S-TR----QAP	PGEAL	-AE--G--HM---QLN-
	Pseudomonas fragi	WP_095036112	--IG---RS-RETRI---QAP	PGQAL	-AD--G--HT---KL--
	Pseudomonas frederiksbergensis	MCE6979288	--IA---RS---TR----QAP	PGEAL	-AE--G--HV---QLD-
	Pseudomonas helleri	MQU04672	--IG---RS-A-TRI---QAP	PGQAL	-AD--S--HH--EKL--
	Pseudomonas indica	WP_084335919	---G---RT-S-TR----QAP	PGEAL	-SE--E--H---AQL--
	Pseudomonas juntendi	WP_161893439	---AD--RN-G-TR----QAA	PGQAL	-AR--G--HE--ERL--
	Pseudomonas kunmingensis	WP_104098815	--IG--SRC-A-TR----QAP	PGEAL	-SA--N--H---D-L--
	Pseudomonas lundensis	WP_094991684	--IG---RS-A-TR----QAP	PGQAL	-AE--S--HA--EQL--
	Pseudomonas mandelii	WP_019580588	--IA---RS---TR----QAP	PGEAL	-AE--G--HV---QLD-
	Pseudomonas mangiferae	WP_143490262	---G---RC-G-TR-----AP	PGEAL	--A--D--RR--DTL--
	Pseudomonas marginalis	KTC17434	--IA---RC-G-TRI----AP	AGQTL	--E--D--HV--DQLQ-
	Pseudomonas monteilii	WP_196171323	---A---R--G-TRI---QA-	PGQAL	-A---G--HE--DRL--
	Pseudomonas oleovorans	PZP76794	---G---RC-S-TRI----PA	PGESL	-SA--A--H--IANL--
	Pseudomonas orientalis	WP_124425354	--IA---RS---TR----QAP	PGQAL	-AE--G--HG---QL--
	Pseudomonas oryzae	WP_090351339	---G---RS-S-TRI----AP	PGEAL	-S---G--HA--D-LH-
	Pseudomonas panacis	WP_154843200	--IG---RC-S-TR----QAP	PGQAL	-AE--G--HT---QL--
	Pseudomonas poae	WP_236325549	--IA---RS---TR----QAP	PGQAL	-AE--G--HS---QLE-
	Pseudomonas pohangensis	WP_090194333	--IA---QS-A-TR-----A-	PGEAL	-AD--S--H---GQLQ-
	Pseudomonas promysalinigenes	WP_186476244	---A---RH-G-TRI----AA	PGQAL	-GA--D--HD--ERL--
	Pseudomonas psychrophila	WP_233099336	--IG---RS-SETR----QAP	PGQAL	-AD--G--HT---QL--
	Pseudomonas putida	WP_133325130	---A---R--G-TRI---QA-	PGQAL	-A---G--HE--DRL--
	Pseudomonas resinovorans	WP_016490187	--IG---RS-S-TR-----AP	SGEAL	-S---E--H----RLD-
	Pseudomonas saxonica	WP_146385600	--MG---RS-SETR----QAP	PGQAL	-AD--S--HT--EQL--
	Pseudomonas stutzeri	WP_102823993	--IG--SRC-A-TH----QAP	PGEAL	-SS--D--H---E-L--
	Pseudomonas syringae	WP_052964617	--IA---RS---TR----QAP	PGEAL	-AE--G--HV---QLD-
	Pseudomonas syringae	WP_197983258	--VA---RC-G-TR-----AP	AGQTL	--E--D--HV--DQLQ-
	Pseudomonas taiwanensis	WP_186617670	---A---RN-G-TRI---QAA	PGEAL	-AE--G--HE--DRL--
	Pseudomonas toyotomiensis	WP_206418777	---G---RS-S-TRI----PA	PGECL	-SA--A--H---GTL--
	Pseudomonas triticumensis	WP_214654131	--IA---RC-G-TR-----AP	AGQAL	--E--D--HV--DQLQ-
	Pseudomonas trivialis	WP_057006489	--IA---RS---TR----QAP	PGQAL	-AE--G--HS---QLE-
	Pseudomonas umsongensis	WP_018928670	--IA---RS---TR----QAP	PGEAL	-AE--G--HV---QLD-
	Pseudomonas viridiflava	WP_122660088	--IA---RC-G-TR-----AP	AGQTL	--E--D--HV--DQLQ-

Figure- S80

Partial sequence alignments of DUF2868 domain-containing protein showing a 5aa Del (highlighted), which is specific for the Fluvialis clade but not present in other *Pseudomonas* species.

Group	Species	Accession	101		143
**Fluvialis Clade (*Caenipseudomonas* gen. nov.) (2/2)**	*Pseudomonas fluvialis*	WP_093984635	RKVVRAELQASASEEVALGVI		PRNQASAPRQALAQRLGQALPA
	Pseudomonas pharmacofabricae	WP_101193825	---------------------		----------------------
**Other *Pseudomonas***	*Pseudomonas lalucatii*	MBS7691199	----A---L-TSR-QL--HAD	GL	--GE---T-RL-IRH-A-----
	Pseudomonas sagittaria	WP_092429735	-A-IQS-VR-TSPA-LNRHAN	GL	-QMN---A-R-------K----
	Pseudomonas aeruginosa	MBN0023926	----A--VL-TRRDQLLQHSN	GL	--QT--E--R--VK--A-V---
	Pseudomonas linyingensis	WP_090308977	-A-IHS-VR-TSPT-LNRHAD	GL	-QVN---A-R-------N----
	Pseudomonas citronellolis	GBL58684	--IAA--TL-TR-DQL-QYAN	GL	--Q--TPQ--Q-----SR----
	Pseudomonas humi	WP_069863036	--IAA--TL-TR-DQL-QYAN	GL	--Q--TPQ--Q-----SR----
	Pseudomonas viridiflava	WP_122497662	--I-Q--VD-TRP--L-KHAN	GI	--I--E-T-AL-IRH-A-----
	Pseudomonas anguilliseptica	WP_233685574	--I-D---L-TR-DQL-RYAN	GL	--SE---T-RL-IRH-A-----
	Pseudomonas syringae	WP_024656018	--I-N---T-TRADQL-KHAQ	GI	-HM--D-T--L-IGH-A-----
	Pseudomonas avellanae	WP_024696536	--I-N---T-TRADQL-KHAQ	GI	-HM--D-T--L-IGH-A-----
	Pseudomonas cichorii	WP_200979461	--I-K---T-TR-DQL-KHAQ	GL	-QME-D-T--L-IGH-A-----
	Pseudomonas peli	WP_090254564	--I-D---L-TR-DQL-RYAN	GL	--SE---T-RL-IRH-A-----
	Pseudomonas leptonychotis	WP_136664005	--I-D---L-TR-DQL-RYAN	GL	--SE---T-SL-IRH-A-----
	Pseudomonas delhiensis	WP_089390848	H-IAA--TL-TR-DQL-QYAN	GL	--Q--TPQ--Q-----SR----
	Pseudomonas benzenivorans	WP_090446679	--I-A---L-THR-QL-RYAD	GL	--TE-D-T-RL-IRH-A-----
	Pseudomonas alcaligenes	WP_187808107	--I-A--TR-TRRDQL-QHAN	GL	--TD-D-T-RL-IRH-A-----
	Pseudomonas stutzeri	WP_237262784	----A--VL-ARR-QL-QH-N	GL	--MD-GSN--L-IRH-A--I--
	Pseudomonas schmalbachii	WP_208314225	---TA--TF-TR-DQL-QHAN	GL	--Q---EQ-RQ-I---S-T---
	Pseudomonas cannabina	KPW77004	--I-N---T-TRADQL-KHAQ	GI	-HME-D-T--L-IGH-A-----
	Pseudomonas thermotolerans	WP_017938111	----A--VK-TSR--LLKNAN	GL	--M--E-T-RL-IRH-A-----
	Pseudomonas amygdali	WP_044324322	--I-N---T-TRADQL-MHAQ	GL	-HIE-D-T--L-IGH-A-----
	Pseudomonas caricapapayae	WP_122342058	--I-N---T-TRADQL-KHAQ	GL	-HIE-D-T--L-IGH-A-----
	Pseudomonas coronafaciens pv.	RMM36869	--I-N---T-TRADQL-KHAQ	GL	-HI--D-T--L-IGH-A-----
	Pseudomonas foliumensis	WP_187523292	--I-N---T-TRPDQL-KNAK	GL	-QI--D-T--L-IGH-A-----
	Pseudomonas caspiana	WP_087265886	--I-N---T-TRPDQL-KNAK	GL	-QI--D-T--L-IGH-A-----
	Pseudomonas oryzae	WP_090351335	-A--AR-VR-TSPA-LQRHAD	GL	-QVN---A-R--V----K----
	Pseudomonas psychrophila	WP_019410270	--I-A---L-TRRDQL-KHAQ	GL	--TE---T--L-INH-SN----
	Pseudomonas carbonaria	WP_187669204	--I-A--VK-TSR--LLKNAN	GL	--VE-E-T-RL-IRH-A-----
	Pseudomonas nitrititolerans	WP_214330816	--I-AS-VL-GR--QL-KYEN	GL	--VE-GSN--L-IRH-A-----
	Paucimonas lemoignei	SQF94159	--I-N---T-TRPDQL-KNAQ	GL	-QI--D-T--L-IGH-A-----
**Other Bacteria**	*Azotobacter chroococcum*	WP_165892699	--I-A---A-TR-DQL-RHAN	GL	--M--D-T-RL-IRH-A--I--
	Azotobacter salinestris	WP_152388227	--I-A---A-TR-DQL-RHAN	GL	--M--D-T-RL-IRH-A--I--
	Azotobacter vinelandii	WP_012703303	--I-A---T-TR-DQL-RHAN	GL	--M--D-T-RL-IRH-A--I--
	Azotobacter beijerinckii	WP_090622881	--I-A---A-TR--QL-RNAK	GL	--M--E-T-RL-IRH-A--I--

Figure- S81

Partial sequence alignments of a hypothetical protein showing a 2aa Del (highlighted), which is specific for the Fluvialis clade.

Group	Species	Accession	72		119
**Fluvialis Clade** (*Caenipseudomonas* gen. nov.) (2/2)	*Pseudomonas fluvialis*	GGH90722	LRGAGQAWVFARSVAAHS		QGEGLDLAQLGCRSLGELLF
	Pseudomonas pharmafabricae	WP_093986556	------------------		--------------------
**Other** ***Pseudomonas***	*Pseudomonas alcaligenes*	MBB4818033	---H-E----------R-	AL	E-S--A-GE--S--------
	Pseudomonas alcaliphila	WP_074682268	---H--P---------R-	AL	E-S--N--E--S--------
	Pseudomonas anguilliseptica	WP_233683206	---H--P---------R-	AL	E-S--A-D---S--------
	Pseudomonas argentinensis	WP_070885835	---N--P---------RQ	AL	--S----Q---S--------
	Pseudomonas borbori	WP_090499825	---H--P---------R-	AL	E-S--A-E---S--------
	Pseudomonas brenneri	WP_229752155	---H------------R-	AL	--D--HMDE--S--------
	Pseudomonas carnis	WP_240357370	---H--P---------R-	AL	--D--HMDE--S--------
	Pseudomonas cavernicola	WP_119953179	---H--P---------R-	SL	E-S--A-RE--S--------
	Pseudomonas cedrina subsp. flu	MBN2994146	---H--P---------R-	AL	--D--H-DE--S--------
	Pseudomonas cerasi	WP_222944498	---N-KH-----------	AL	-DG--NMDE--T--------
	Pseudomonas chengduensis	WP_230874323	---H--P---------R-	VL	E-S--N--E--S--------
	Pseudomonas citronellolis	GBL58841	---H--P---------R-	AL	EAS-F------S--------
	Pseudomonas composti	NYG65816	---H--P---------R-	VL	E-S--N--E--S--------
	Pseudomonas composti	WP_061238296	---NE-P---------R-	VL	E-S--N--E--S--------
	Pseudomonas costantinii	WP_218169790	---H--P---------R-	AL	--D--HMDE--S--------
	Pseudomonas delhiensis	SDK78578	---H--P---------R-	AL	EAS-F------R--------
	Pseudomonas fluorescens	WP_150773026	---H------------R-	AL	--D--HMDE--T--------
	Pseudomonas frederiksbergensis	WP_163910613	----------------R-	AL	--D--HMDE--S--------
	Pseudomonas fulva	WP_013789404	---N--P---------RQ	AL	E-SN---QE--S--------
	Pseudomonas gessardii	WP_154220704	---H------------R-	AL	--D--HMDE--S--------
	Pseudomonas guguanensis	WP_090429849	---H--P---------R-	VL	E-S--N--E--S--------
	Pseudomonas haemolytica	WP_153837568	---H--P---------R-	AL	--D--HMDE--S--------
	Pseudomonas indoloxydans	WP_108234771	---H------------R-	AL	E-S--N--E--S--------
	Pseudomonas izuensis	WP_160108488	---H-----------SR-	AL	--D--HMDE--S--------
	Pseudomonas kairouanensis	WP_135289281	------P---------R-	AL	--D--HMDE--S--------
	Pseudomonas karstica	WP_154741564	---H------------R-	AL	--D--HMDE--S--------
	Pseudomonas khazarica	WP_134677621	---H--P---------R-	VL	E-S--N--E--S--------
	Pseudomonas lalkuanensis	WP_151138442	---H-EI---------R-	AL	E-S----H---S--------
	Pseudomonas lalucatii	WP_215730919	---H--P---------RC	AL	E-S--N-D---S--------
	Pseudomonas leptonychotis	WP_136663633	---H--P---------R-	AL	E-S--A-D---S--------
	Pseudomonas lurida	WP_219855148	---H--P---------R-	AL	--D--QMDE--S--------
	Pseudomonas mandelii PD30	KDD67228	---H-E----------R-	AL	--D--HMDE--S--------
	Pseudomonas mangrovi	PTU73091	----DRP---------R-	AL	E-S-V------S--------
	Pseudomonas mendocina	WP_106742880	---HD-P---------R-	AL	E-S--N--E--S--------
	Pseudomonas mendocina	WP_143507474	---H------------R-	AL	E-S--N--E--S--------
	Pseudomonas oleovorans	HIQ41174	---H--P---------R-	VL	E-S--N--E--S--------
	Pseudomonas palleroniana	WP_232965738	---H--P---------R-	AL	--D--HMDE--S--------
	Pseudomonas panacis	WP_154843320	---H------------R-	AL	--D--HMDE--S--------
	Pseudomonas paralactis	WP_189663556	---H--P---------R-	AL	--D--HMDE--S--------
	Pseudomonas peli	WP_186343838	---H--P---------R-	AL	E-S--A-D---S--------
	Pseudomonas piscicultura e	WP_228758362	---H--P---------R-	AL	--D--HMDE--S--------
	Pseudomonas poae	WP_105695933	---H--P---------R-	AL	--D--HMDE--S--------
	Pseudomonas pohangensis	SDU38321	---N-EP---------R-	AL	E-S--------S--------
	Pseudomonas proteolytica	WP_186351795	---H------------R-	AL	--D--HMDE--S--------
	Pseudomonas reactans	WP_218178763	---H--P---------R-	AL	--D--HMDE--S--------
	Pseudomonas resinovorans	WP_028626918	---H-E----------RA	AL	E-S----H---S--------
	Pseudomonas rhodesiae	WP_221433540	---H--P---------R-	AL	--D--QMDE--S--------
	Pseudomonas sediminis	WP_179543594	---H------------R-	AL	E-S--N--E--S--------
	Pseudomonas seleniipraecipitan	WP_070880381	---N-EP---------QR	VL	N-S----Q---S--------
	Pseudomonas spelaei	WP_155582534	---H------------R-	AL	--D--HMDE--S--------
	Pseudomonas straminea	WP_093500580	---N--P---------RQ	AL	E-S----Q---S--------
	Pseudomonas taeanensis	WP_025163863	---H--P---------R-	AL	E-S-MG-D---S--------
	Pseudomonas taiwanensis	NWL79455	---H-EI---------RA	AL	E-S----H---S--------
	Pseudomonas tohonis	WP_173174862	---H-E----------R-	AL	E-S--A-GE--S--------
	Pseudomonas toyotomiensis	WP_206418865	---H--P---------R-	AL	E-S--N--E--S--------
	Pseudomonas toyotomiensis	WP_230875443	---H-LP---------R-	AL	E-S--N--E--S--------
	Pseudomonas trivialis	WP_049710900	---Q--P---------R-	AL	--D--HMDE--S--------
	Pseudomonas ullengensis	WP_183087663	-----E----------R-	AL	E-S-F--GK--S--------
	Pseudomonas wenzhouensis	WP_230926023	---H--P---------R-	VL	EDS--N--E--S--------
	Pseudomonas xionganensis	WP_218572006	---H--P---------R-	AL	E-S--N--E--S--------
	Pseudomonas yamanorum	WP_179448528	---H------------R-	AL	--D--HMDE--S--------

Figure- S82

Partial sequence alignments of Putative chorismate pyruvate-lyase protein showing a 2aa Del (highlighted), which is specific for the Fluvialis clade.

Group	Species	Accession	77		117
Fluvialis Clade (*Caenipseudomonas* gen. nov.) (2/2)	*Pseudomonas fluvialis*	WP_093984289	LYLQVLAIGGSEQHPQLGVDA	GA	PVIEYALQMRQFPQSQLL
	Pseudomonas pharmacofabricae	WP_101194183	-------------Q-------	--	------------------
Other *Pseudomonas*	*Pseudomonas alcaligenes*	WP_110683875	---E--P-T---DA----G-G		AA---V-K----------
	Pseudomonas alcaliphila	WP_075749432	---D--P-----DA-S-DGST		-A-----K------E---
	Pseudomonas anguilliseptica	WP_090376075	---E--P-S--TDT----GSG		-A-----K----------
	Pseudomonas argentinensis	WP_070887301	------P----PEA--I-S--		A-F----K------E---
	Pseudomonas azadiae	MBV4452655	---D--P-T-TAEA----G-G		-------K----------
	Pseudomonas borbori	WP_090502392	---E--P-S--VEA----G-G		-A-----K------D---
	Pseudomonas brassicacearum	WP_123344900	---E--P-T--AEA----G-G		-A-----K----------
	Pseudomonas campi	WP_173203874	---E--P-S---SA-L--GSG		-A---V-K----------
	Pseudomonas canadensis	WP_123475610	---E--P-T--AEA----G-G		--V----K----------
	Pseudomonas carbonaria	WP_187670293	---E--P-T--AES----D-G		-A-----K----------
	Pseudomonas cavernae	WP_119892314	---E--P-T--AEA---AGAG		-A-----K----------
	Pseudomonas cavernicola	WP_119956155	---E--P-S--LEA----G-G		-A-----K----------
	Pseudomonas chengduensis	WP_230874166	---D--P-----DA-R-DGST		-A-----K------E---
	Pseudomonas daroniae	WP_131180316	---E--P-S--VDA-R-DGAD		-I-----K------E---
	Pseudomonas denitrificans	QEY73601	---D-I--T---TA---NGEG		-A---M--C-E---T---
	Pseudomonas dryadis	WP_131176734	------P-S--SEA-N-NG-G		--------------E---
	Pseudomonas flexibilis	WP_039606406	--R---P-C----A--F-S--		------------------
	Pseudomonas fluorescens	WP_240055022	---E--P-T--AEA----G-G		-------K----------
	Pseudomonas fulva	WP_013789966	---E--P-S--AEA--I-G-S		A-F----K------E---
	Pseudomonas guguanensis	WP_090435324	---E--P-----DA-S-DGST		-A-----K------E---
	Pseudomonas guryensis	WP_220494098	---E--P-S---NA----GEG		-A-----K----------
	Pseudomonas japonica	MBA1245321	---D--SVT----A-A--GEG		-A-----K----------
	Pseudomonas khazarica	WP_210389654	---E-V------DA-S-DGST		-A-----K------E---
	Pseudomonas knackmussii	WP_160288407	---D--P-T---SE-R--G-G		-A-----KC-E----G--
	Pseudomonas lalucatii	MBS7661958	---E--P-S--VEA-R-SG-G		-I-----K------D---
	Pseudomonas leptonychotis	WP_136665187	---E-VP----VDA-E--GNG		-A-----K----------
	Pseudomonas mangiferae	WP_143490238	---E--P-T---TE-R-AG-G		-A-----K----S-----
	Pseudomonas massiliensis	WP_040259381	---E----T----A-N--GSG		-A-D---K----------
	Pseudomonas mendocina	WP_096827264	---E--P-----TA-T-NDG-		-A-----K------EN--
	Pseudomonas mohnii	MBH8614659	---E--P-T--AEA----G-G		--V----K----------
	Pseudomonas moorei	WP_090325189	---E--P-T--AED----G-G		-------K----------
	Pseudomonas multiresinivorans	WP_169936541	---D-I--T---SA--I-G-G		-A---MI-C-E-------
	Pseudomonas nitritireducens	WP_170859962	---D-I--T---SA--I-G-G		-A---MI-C-E-------
	Pseudomonas nitroreducens	WP_017516320	---D-I--T---SA----G-G		-A---VI-C-E-------
	Pseudomonas oleovorans	PZP82454	---D--P-----DA-S-DGST		-A-----K------E---
	Pseudomonas oleovorans	WP_150606445	---E-V------DA-S-DGST		-A-----K------E---
	Pseudomonas oryzihabitans	WP_059313345	--QD-V--T-T-SA----G-G		-------R--E-------
	Pseudomonas panipatensis	WP_090263307	---E--P-T---SE-HI-G-G		-------KC-E---TG--
	Pseudomonas peli	WP_186343989	---E--P-S--VDA--F-S-G		-------K----------
	Pseudomonas poae	WP_197626751	---E--P-T--AEA----G-G		-------K----------
	Pseudomonas pohangensis	WP_090196008	------P-T--AEA---DGSG		---------A--------
	Pseudomonas psychrophila	WP_026014195	---D--P-T-TIEA----G-G		-------K----------
	Pseudomonas reactans	NWE89576	---E--P-T--AEA----G-G		--V----K----------
	Pseudomonas rhodesiae	WP_187697015	---E--P-T-TAES----G-G		-------K----------
	Pseudomonas salomonii	WP_218178158	---D--P-T--AEA----G-G		-------K----------
	Pseudomonas sediminis	WP_099526066	---D--P-----DA-S-DGGT		-A-----K------E---
	Pseudomonas simiae	WP_217908709	---E--P-T--AEA----G-G		-------K----------
	Pseudomonas stutzeri	WP_102829569	---E--P-T----A---DGAG		AP-----K--E-------
	Pseudomonas thivervalensis	WP_053125273	---E--P-T--TEA----G-G		-A-----K----------
	Pseudomonas toyotomiensis	MBG0841341	---D--PV----DA-S-DGST		-------K------E---
	Pseudomonas tructae	WP_130266329	------P-T--A-A----GEG		AP--------------M-
	Pseudomonas ullengensis	MBB2497444	---E--P-T---SA----GAG		-A---V-K----------
	Pseudomonas viridiflava	WP_122491886	---E--P-T--ADA--INGSG		---D---K----------
	Pseudomonas wenzhouensis	WP_230925350	------P-----DA-S-DGST		-A-----K------E---
	Pseudomonas yangonensis	WP_161867157	---D--P-----DA-S-DGST		-A-----K------E---
Other Bacteria	*Azotobacter beijerinckii*	SEI80079	---D--P-T---SA---AGEG		------VK----------
	Azotobacter salinestris	WP_152387219	---E--P-T---NA---AG-G		------VK----------

Figure-S83

Partial sequence alignments of Bifunctional aminoglycoside phosphotransferase/ATP-binding protein showing a 2aa Ins (highlighted), which is specific for the Fluvialis clade.

Group	Species	Accession	160		200
**Fluvialis Clade (*Caenipseudomonas* gen. nov.) (2/2)**	*Pseudomonas pharmacofabricae*	WP_101192354	GDIAAGTLVVYRETTLSS	R	PPLPEAIPQRPPLPLHLDEQRA
	Pseudomonas fluvialis	WP_229727124	----------------F-	-	----------------------
**Other *Pseudomonas***	*Pseudomonas aeruginosa*	WP_148105466	-------------PE--R		-S--DVE--L--F-MS-E----
	Pseudomonas chloritidismutans	WP_221102885	--L----------RPQAR		-T---VDAAK--FA-S------
	Pseudomonas chlororaphis	WP_123328611	--L------I---QAVQR		------QARHA-F--R-N----
	Pseudomonas fluorescens	MBM7764739	-----------S-RP-AR		-Q--D-L-RSS-V--N-G----
	Pseudomonas khorasanensis	WP_186530060	-----------G-RP--R		-S----E-R-A-I--T-S----
	Pseudomonas kunmingensis	SFI82685	--L----------RPQVR		-T---VEAAK--FA-S------
	Pseudomonas mohnii	WP_090464663	---------I---QP--R		-Q--P-E-R--AF--T-T----
	Pseudomonas moraviensis	WP_133339781	-----------S-R---R		-Q--D-E-R-C-V--T-N----
	Pseudomonas otitidis	WP_165670325	-------------EKA-R		-N----T-L-S-V--S-E----
	Pseudomonas stutzeri	VEI36460	--L----------RPQAR		-T---VEAAK--FA-S------
	Pseudomonas syringae	WP_236480436	-----------N-QP-KR		-Q--Q-E-R-S-V--T-N----
	Pseudomonas xanthomarina	WP_073301373	--L----------RNV-A		-A----EAVT-----K----H-
**Other Bacteria**	*Acinetobacter baumannii*	SST10451	-------------PE--R		-S--DVE--L--F-MS-E----

Figure- S84

Partial sequence alignments of RDD family protein showing a 1aa Ins (highlighted), which is specific for the Fluvialis clade.

Group	Species	Accession	493		539
Fluvialis Clade (*Caenipseudomonas* gen. nov.) (2/2)	*Pseudomonas fluvialis*	WP_093986880	WSLAPLQLQLGDNRIDGQASL	GS	GQRLAGELRWKLPALQQLWPELAG
	Pseudomonas pharmacofabricae	WP_101192570	---------------------	--	------------------------
Other *Pseudomonas*	*Pseudomonas agarici*	WP_218165936	-D-SA-EVR------S-NG--		R-----Q-ELNM-R-G----Q-R-
	Pseudomonas arsenicoxydans	WP_208669060	---NA--IR----S-S-KG--		Q-K---QIDI---R-A----Q-R-
	Pseudomonas brassicacearum	WP_123366267	-N-SA--IR----S-N-KG--		Q-K---QIDI---R-A----Q-R-
	Pseudomonas caspiana	WP_140894232	-N-SA--IR----S-N-KG--		Q-K---QIDI---R-A----Q-R-
	Pseudomonas chlororaphis	WP_124321382	---SA-DIR------N-SGR-		Q-----Q-DI---R-G------R-
	Pseudomonas farris	WP_225923200	-N-ST--IR----S-N--G--		Q-K---RIDI-M-R-A----Q-R-
	Pseudomonas fluorescens	MBI3908688	-N-NA--VR----S-S-KG--		Q-K---QIDL---R-A------R-
	Pseudomonas frederiksbergensis	WP_241015394	-N-SA--IR----S-N--G--		Q-K---Q-DI-M-R-A----Q-R-
	Pseudomonas gingeri	WP_218170695	-N--A-DIR-----VT-NG--		Q-----Q-DLDM-R-G----Q-R-
	Pseudomonas granadensis	MBN6776355	-N-NA--IR----S-S-KG--		Q-K---QIDI---R-A----Q-R-
	Pseudomonas gregormendelii	MBN3965377	-N-SA--IR----S-N-KG--		Q-K---QIDI-M-R-A----Q-R-
	Pseudomonas jessenii	WP_110661251	-N-NA--IR----S-S-KG--		Q-K---QIDL---R-A------R-
	Pseudomonas kribbensis	WP_134827390	-N-NA--IR----S-N-KG--		Q-K---QIDI---R-A----Q-R-
	Pseudomonas laurylsulfativoran	WP_103395457	-N-NA--VR----S-S-KG--		Q-K---QIDL---R-A------R-
	Pseudomonas lini	WP_050683223	-N-SA--IR----S-N--G--		Q-K---RIDI-M-R-A----Q-R-
	Pseudomonas mandelii	WP_140680070	-N-SA--IR----S-N-KG--		Q-K---QIDI---R-A----Q-R-
	Pseudomonas marvdashtae	MBV4551184	-N-SA-DIR------N-S-N-		Q-K-T-QIDI--SR-A----R-R-
	Pseudomonas mohnii	WP_090468392	-N-DA--IR----S-N-KG--		Q-K---QIDI---R-A----Q-R-
	Pseudomonas protegens	WP_210715285	-T-GA-DIR------N-SG--		Q-KI--Q-EI-M-R-G------R-
	Pseudomonas putida	WP_110963762	-N-NA--IR----S-N-KG--		Q-K---QIDI---R-A----Q-R-
	Pseudomonas sagittaria	WP_092430692	-Q--E-A-R---------GA-		NE--D-QF-LA-AR-D------H-
	Pseudomonas silesiensis	WP_064678773	-N-SA--IR----S-N-KG--		Q-K---QIDI---R-A----Q-R-
	Pseudomonas syringae	WP_052967724	-N-ST--IR----S-N--G--		Q-K---RIDI-M-R-A----Q-R-
	Pseudomonas umsongensis	WP_018925468	-N-SA--IR----S-N--G--		Q-K---QIDI-M-R-A----Q-R-
	Pseudomonas viridiflava	WP_198696511	-N--G-SMR------E-RG--		D---Q-Q-DLN--R-G----R-Q-
	Pseudomonas zanjanensis	MBV4494871	-N-SA-DIR------N-S---		Q-K-T-QIDI--SR-A----Q-R-

Figure- S85

Partial sequence alignments of Translocation/assembly module TamB protein showing a 2aa Ins (highlighted), which is specific for the Fluvialis clade.

Group	Species	Accession	138		176
Genus *Azotobacter* (4/4)	*Azotobacter vinelandii*	WP_012699745	YPGEHLRIGRQRIRS	LT	NEGTWWDIHMESVNWTMDTSLL
	Azotobacter salinestris	WP_152388783	-------L----V--	--	---------I--L---------
	Azotobacter chroococcum	WP_089169063	-------L----V--	--	---------I--L---------
	Azotobacter beijerinckii	WP_090619573	-------L----V--	-S	G-A-----RI--L---------
Other *Pseudomonadaceae*	*Pseudomonas abietaniphila*	WP_074753710	----I-KF----L-N		DD-Q-H-TNI-AL---F--T--
	Pseudomonas aeruginosa	WP_003110464	-------F----L-E		DS-Q-Q-TNI-AL--SFE-T--
	Pseudomonas aestus	WP_031320935	----Q-KF----L-N		DD-Q-R-TNI-AL---F--T--
	Pseudomonas agarici	WP_017130747	----I-KF----L-N		DD-Q-R-TNI-AL---F--T--
	Pseudomonas alcaligenes	WP_061904331	--Q-S--F------A		DD-L---TNI-ALR-NFE-T--
	Pseudomonas alcaliphila	WP_075751071	-------V----L--		D------TNI-A---RF--T--
	Pseudomonas alkylphenolica	WP_038607104	----Q--F----L-N		DD-M-R-TNI-AL---F--T--
	Pseudomonas allii	WP_058422921	----I-KL----L-N		DD-Q-R-TNI-AL---F--T--
	Pseudomonas amygdali	WP_044318219	----I-KV----L-N		DD-Q-H-TNI-AI--NF--T--
	Pseudomonas anguilliseptica	WP_090387612	-------L----LHN		ED-Q-R-SNI-AL---F--T--
	Pseudomonas antarctica	WP_064450795	----L-KF----LNN		DD-Q-R-TNI-AL---F--T--
	Pseudomonas arsenicoxydans	WP_090188169	----M-KL----LHN		AD-Q-R-TNI-AL---F--T--
	Pseudomonas asiatica	WP_013974153	----Q--F----L--		DD-M-R-TNI-AL---F--T--
	Pseudomonas asplenii	WP_090205266	----I-KF----L-N		DD-Q-R-TNI-AL---F--T--
	Pseudomonas asturiensis	WP_073163373	----I-KV----L-N		DD-Q-H-TNI-AI---F--T--
	Pseudomonas atacamensis	WP_016771599	----I-KF----L-N		DD-Q-R-TNI-AL---F--T--
	Pseudomonas avellanae	WP_005615404	----I-KV----L-N		DD-Q-H-TNI-AI--NF--T--
	Pseudomonas azotoformans	WP_033899569	----I-KL----L-N		DD-Q-R-TNI-AL---F--T--
	Pseudomonas baetica	WP_100846172	----M-KF----L-N		DD-Q-R-TNI-AL---F--T--
	Pseudomonas benzenivorans	WP_090442676	-------F----V--		D------SNI-ALR-SF--T--
	Pseudomonas bohemica	WP_110948615	----I-KF----L-N		DD-Q-H-TNI-AL---F--T--
	Pseudomonas borbori	WP_090502715	-------L----LHN		DD-Q-R-T-I-AL---F--T--
	Pseudomonas brassicacearum	WP_025215370	----M-KF----L-N		DD-Q-R-TNI-AL---F--T--
	Pseudomonas brassicae	WP_163947358	----Q--F----L-N		DD-M-R-TNI-AL---F--T--
	Pseudomonas brenneri	WP_090291972	----I-KL----L-N		-D-Q-R-TNI-AL---F--T--
	Pseudomonas canadensis	WP_028618926	----I-KL----L-N		DD-Q-R-TNI-AL---F--T--
	Pseudomonas cannabina	WP_054999158	----I-KV----L-N		DD-Q-H-TNI-AI--SF--T--
	Pseudomonas capeferrum	WP_033702876	----Q--F----L--		DD-M-R-TNI-AL--SF--T--
	Pseudomonas caricapapayae	WP_055007591	----I-KV----L-N		DD-Q-H-TNI-AI--SF--T--
	Pseudomonas carnis	WP_034128776	----I-KF----L-N		DD-Q-R-TNI-AL---F--T--
	Pseudomonas caspiana	WP_087268952	----I-KV----L-N		DD-Q-H-TNI-A---VF--T--
	Pseudomonas cedrina	WP_076951266	----I-KL----L-N		DD-Q-R-TNI-AL---F--T--
	Pseudomonas cerasi	WP_003433626	----I-KV----L-N		DD-Q-H-TNI-AI--SF--T--
	Pseudomonas chengduensis	WP_017678173	-------V----L--		D------TNI-A---RF--T--
	Pseudomonas chlororaphis	WP_038636446	----Q-KF----L-N		DD-Q-R-TNI-AL---F--T--
	Pseudomonas cichorii	WP_025258719	----I-KV----L-N		DD-Q-H-TNI-AI---F--T--
	Pseudomonas citronellolis	WP_074984677	-------F----L-D		DS-M-Q-TNI-AL---F--T--
	Pseudomonas composti	WP_074935908	-------V----L--		DD-A---TNI-A---RF--T--
	Pseudomonas congelans	WP_010435466	----I-KV----L-N		DD-Q-H-TNI-AI--SF--T--
	Pseudomonas coronafaciens	WP_024690122	----I-KV----L-N		DD-Q-H-TNI-AI--SF--T--
	Pseudomonas corrugata	WP_024777608	----Q-KF----L-N		DD-Q-R-TNI-AL---F--T--
	Pseudomonas costantinii	WP_071486066	----I-KL----L-N		DD-Q-R-TNI-AL---F--T--
	Pseudomonas cremoris	WP_185708436	----I-KL----L-N		DD-Q-R-TNI-AL---F--T--
	Pseudomonas cuatrocienegasensi	WP_069520055	-------L----L--		D----R-TNI-AL--SF--T--
	Pseudomonas delhiensis	WP_089389835	-------F----L-D		DS-M-Q-TNI-AL---F--T--
	Pseudomonas denitrificans	WP_003110464	-------F----L-E		DS-Q-Q-TNI-AL--SFE-T--
	Pseudomonas donghuensis	WP_010220710	----Q--F----L-N		DD-M-R-TNI-AL---F--T--
	Pseudomonas edaphica	WP_017135216	----L-KL----L-N		DD-Q-R-TNI-AL---F--T--
	Pseudomonas entomophila	WP_011535587	----Q--F----L--		DD-M-R-TNI-AL---F--T--
	Pseudomonas extremaustralis	WP_010565647	----I-KL----L-N		DD-Q-R-TNI-AL---F--T--
	Pseudomonas extremorientalis	WP_071488567	----I-KL----L-N		DD-Q-R-TNI-AL---F--T--
	Pseudomonas ficuserectae	WP_004658317	----I-KV----L-N		DD-Q-H-TNI-AI--NF--T--
	Pseudomonas fildesensis	WP_048723792	----I-KL----L-N		DD-Q-R-TNI-AL---F--T--
	Pseudomonas floridensis	WP_083185092	----I-KV----L-N		DD-Q-H-TNI-A----F--T--
	Pseudomonas fluorescens	WP_053254506	----L-KV----LNN		DD-Q-R-TNI-AL---F--T--
	Pseudomonas furukawaii	WP_003448855	-------F----V--		D------TNI-ALR-SF--T--
	Pseudomonas fuscovaginae	WP_010445125	----I-KF----L-N		DD-Q-R-TNI-AL---F--T--
	Pseudomonas gessardii	WP_076965134	----I-KL----L-N		-D-Q-R-TNI-AL---F--T--
	Pseudomonas gingeri	WP_017124117	----L-KF----L-N		DD-Q-R-TNI-AL---F--T--
	Pseudomonas graminis	WP_074884191	----I-KL----L-N		DD-Q-H-VNI-AL---F--T--
	Pseudomonas granadensis	WP_090281862	----I-KF----L-N		DD-Q-R-TNI-AL---F--T--
	Pseudomonas grimontii	WP_090409219	----I-KL----L-N		DD-Q-R-TNI-AL---F--T--
	Pseudomonas guariconensis	WP_043207420	----Q--L----L--		DD-M-R-TNI-AL---F--T--
	Pseudomonas guguanensis	WP_090427039	-------F----L--		D------TNI-A---RF--T--
	Pseudomonas haemolytica	WP_034118209	----I-KF----L-N		DD-Q-R-TNI-AL---F--T--
	Pseudomonas helmanticensis	WP_134174732	----M-KF----L-N		DD-Q-R-TNI-AL---F--T--
	Pseudomonas humi	WP_069866609	-------F----L-D		DS-M-Q-TNI-AL---F--T--
	Pseudomonas hunanensis	WP_003251756	----Q--F----L--		DD-M-R-TNI-AL---F--T--
	Pseudomonas hydrolytica	WP_041977073	-------V----L--		DD-A---TNI-A---RF--T--
	Pseudomonas indica	WP_084336279	-------F----V--		D------TNI-AL---F--T--
	Pseudomonas inefficax	WP_013974153	----Q--F----L--		DD-M-R-TNI-AL---F--T--
	Pseudomonas japonica	WP_042122921	----E--F----L-N		DD-M-R-TNI-AL---F--T--
	Pseudomonas jessenii	WP_090457585	----I-KL----L-N		DD-Q-R-TNI-AL---F--T--
	Pseudomonas jinjuensis	WP_090416078	-------F----L-E		ES-L-Q-TNI-AL--SF--T--
	Pseudomonas juntendi	WP_054905664	----Q--L----L--		DD-M-R-TNI-AL---F--T--
	Pseudomonas kairouanensis	WP_135287506	----I-KL----L-N		DD-Q-R-TNI-AL---F--T--
	Pseudomonas khazarica	WP_134678144	-------V----L--		D--S---TNI-A---RF--T--
	Pseudomonas kilonensis	WP_077505786	----Q-KF----L-N		DD-Q-R-TNI-AL---F--T--
	Pseudomonas kitaguniensis	WP_152745241	----I-KL----L-N		DD-Q-R-TNI-AL---F--T--
	Pseudomonas knackmussii	WP_084166574	-------F----L-D		DS-M-A-TNI-AL---F--T--
	Pseudomonas koreensis	WP_041479487	----M-KF----L-N		DD-Q-R-TNI-AL---F--T--
	Pseudomonas kribbensis	WP_114881482	----I-KF----L-N		DD-Q-R-TNI-AL---F--T--
	Pseudomonas lactis	WP_003188737	----I-KF----L-N		DD-Q-R-TNI-AL---F--T--
	Pseudomonas laurylsulfatiphila	WP_104449447	----I-KL----LHN		DD-Q-R-TNI-AL---F--T--
	Pseudomonas leptonychotis	WP_136662921	-------L----M--		DD-----TNI-ALR-SF--T--
	Pseudomonas libanensis	WP_057012991	----I-KF----L-N		DD-Q-R-TNI-AL---F--T--
	Pseudomonas lini	WP_048396516	----I-KL----L-N		DD-Q-R-TNI-AL---F--T--
	Pseudomonas lundensis	WP_047275508	----Q-KF----L-N		DD---R-TNI-ALA--F--T--
	Pseudomonas lurida	WP_034102285	----I-KL----L-N		DD-Q-R-TNI-AL---F--T--

	Pseudomonas lutea	WP_037014967	----I-KV----L-N	DD-Q-H-TNI-A----F--T--
	Pseudomonas mandelii	WP_010463117	----I-KL----L-N	DD-Q-R-TNI-AL---F--T--
	Pseudomonas marginalis	WP_064054807	----I-KL----L-N	DD-Q-R-TNI-AL---F--T--
	Pseudomonas massiliensis	WP_040263459	----L-KF----L-N	DD-Q-R-TNI-ALA--F--T--
	Pseudomonas mediterranea	WP_047701451	----Q-KF----L-N	DD-Q-R-TNI-AL---F--T--
	Pseudomonas meliae	WP_004658317	----I-KV----L-N	DD-Q-H-TNI-AI--NF--T--
	Pseudomonas mendocina	WP_041772813	-------V----L--	D------TNI-A-H-NF--T--
	Pseudomonas migulae	WP_084320711	----Q-KL----L-N	DD-Q-R-TNI-AL---F--T--
	Pseudomonas migulae	WP_084322869	-------L----L-E	DS-Q-M-TNI-AL---FE-T--
	Pseudomonas mohnii	WP_090464896	----I-KV----LHN	DD-Q-R-TNI-A----F--T--
	Pseudomonas monteilii	WP_003257139	----Q--F----L--	DD-M-R-TNI-AL---F--T--
	Pseudomonas moorei	WP_090324994	----I-KF----LHN	DD-Q-R-TNI-AL---F--T--
	Pseudomonas moraviensis	WP_083354260	----I-KF----L-N	DD-Q-R-TNI-AL---F--T--
	Pseudomonas mosselii	WP_084941976	----Q--F----L--	DD-M-R-TNI-AL---F--T--
	Pseudomonas mucidolens	WP_090221349	----V-KF----L-N	DD-Q-R-TNI-AL---F--T--
	Pseudomonas multiresinivorans	WP_169942573	-------F----L-E	DS-M-Q-TNI-AL---F--T--
	Pseudomonas nabeulensis	WP_135309922	----I-KL----L-N	DD-Q-R-TNI-AL---F--T--
	Pseudomonas nitritireducens	WP_065086699	-------F----L-E	DS-M-Q-TNI-AL---F--T--
	Pseudomonas nitroreducens	WP_065086699	-------F----L-E	DS-M-Q-TNI-AL---F--T--
	Pseudomonas oleovorans	WP_150610194	-------V----L--	D--S---TNI-AI--RF--T--
	Pseudomonas orientalis	WP_057724654	----I-KF----L-N	DD-Q-R-TNI-AL---F--T--
	Pseudomonas otitidis	WP_074970782	-------F----V--	A------TNI-ALR-SF--T--
	Pseudomonas ovata	WP_109512500	----I-KV----L-N	DD-Q-R-VNI-AI---F--T--
	Pseudomonas palleroniana	WP_090366647	----M-KL----L-N	DD-Q-R-TNI-AL---F--T--
	Pseudomonas panacis	WP_057004281	----L-KF----L-N	DD-Q-R-TNI-AL---F--T--
	Pseudomonas parafulva	WP_028635237	----Q--L----L--	DD-M-R-TNI-AL---F--T--
	Pseudomonas paralactis	WP_057701521	----I-KF----L-N	DD-Q-R-TNI-AL---F--T--
	Pseudomonas piscis	WP_152897304	----Q-KF----L-N	DD-Q-R-TNI-AL---F--T--
	Pseudomonas plecoglossicida	WP_013974153	----Q--F----L--	DD-M-R-TNI-AL---F--T--
	Pseudomonas poae	WP_004371916	----I-KF----L-N	DD-Q-R-TNI-AL---F--T--
	Pseudomonas prosekii	WP_092272975	----M-KL----L-N	DD-Q-R-SNI-AL---F--T--
	Pseudomonas protegens	WP_015634237	----Q-KF----L-N	DD-Q-R-TNI-AL---F--T--
	Pseudomonas proteolytica	WP_070995618	----I-KL----L-N	-D-Q-R-TNI-AL---F--T--
	Pseudomonas pudica	WP_046617052	----Q--F----L--	DD-M-R-TNI-AL---F--T--
	Pseudomonas putida	WP_016498006	----Q--L----L--	DD-M-R-TNI-AL---F--T--
	Pseudomonas qingdaonensis	WP_058540863	----Q--L----L--	DD-M-R-TNI-AL---F--T--
	Pseudomonas reactans	WP_177002465	----I-KL----L-N	DD-Q-R-TNI-AL---F--T--
	Pseudomonas reidholzensis	WP_119138487	----Y--F----L-N	DD-M-R-TNI-AL--SF--T--
**Other *Pseudomonadaceae***	*Pseudomonas reinekei*	WP_075945591	----I-KF----L-N	DD-Q-R-TNI-AL---F--T--
	Pseudomonas resinovorans	WP_016491118	-------F----V--	D------TNI-ALR-SF--T--
	Pseudomonas rhodesiae	WP_034111837	----I-KF----L-N	DD-Q-R-TNI-AL---F--T--
	Pseudomonas salomonii	WP_069786116	----L-KL----L-N	DD-Q-R-TNI-AL---F--T--
	Pseudomonas saponiphila	WP_092319790	----Q-KF----L-N	DD-Q-R-TNI-AL---F--T--
	Pseudomonas savastanoi	WP_004658317	----I-KV----L-N	DD-Q-H-TNI-AI--NF--T--
	Pseudomonas saxonica	WP_122786467	----E--L----L-N	DD-L-R-TNI-AL---F--T--
	Pseudomonas sediminis	WP_099522384	-------V----L--	D------TNI-A---RF--T--
	Pseudomonas segetis	WP_089359438	-------L----V--	D------TNI-AL--EF--T--
	Pseudomonas shirazica	WP_013974153	----Q--F----L--	DD-M-R-TNI-AL---F--T--
	Pseudomonas sichuanensis	WP_110991667	----Q--L----L--	DD-M-R-TNI-AL---F--T--
	Pseudomonas sihuiensis	WP_017678173	-------V----L--	D------TNI-A---RF--T--
	Pseudomonas silesiensis	WP_064679630	----I-KL----L-N	DD-Q-R-TNI-AL---F--T--
	Pseudomonas simiae	WP_010212974	----I-KL----L-N	DD-Q-R-TNI-AL---F--T--
	Pseudomonas sivasensis	WP_032886361	----I-KL----L-N	DD-Q-R-TNI-AL---F--T--
	Pseudomonas soli	WP_094011732	----Q--F----L--	DD-M-R-TNI-AL---F--T--
	Pseudomonas synxantha	WP_046070997	----I-KF----L-N	DD-Q-R-TNI-AL---F--T--
	Pseudomonas syringae	WP_025390557	----I-KV----L-N	DD-Q-H-TNI-AI--SF--T--
	Pseudomonas taeanensis	WP_025164987	----E--F----L-N	DDSQ-R-TNI-AL---F--T--
	Pseudomonas taiwanensis	WP_023378768	----Q--L----L--	DD-M-R-TNI-AL---F--T--
	Pseudomonas thivervalensis	WP_053125242	----Q-KF----L-N	DD-Q-R-TNI-AL---F--T--
	Pseudomonas tolaasii	WP_016974202	----I-KF----L-N	DD-Q-R-TNI-AL---F--T--
	Pseudomonas toyotomiensis	WP_074913529	-------V----L--	-------TNI-A---RF--T--
	Pseudomonas tremae	WP_024690122	----I-KV----L-N	DD-Q-H-TNI-AI--SF--T--
	Pseudomonas trivialis	WP_049709894	----I-KL----L-N	DD-Q-R-TNI-AL---F--T--
	Pseudomonas typographi	WP_190418459	----IMKF----L-Q	-D-Q-H-NYI--LT-NF--T--
	Pseudomonas umsongensis	WP_020796865	----I-KL----LNN	DD-Q-R-TNI-AL---F--T--
	Pseudomonas vancouverensis	WP_093214081	----I-KL----L-N	DD-Q-R-TNI-AL---F--T--
	Pseudomonas veronii	WP_046384627	----L-KF----L-N	DD-Q-R-TNI-AL---F--T--
	Pseudomonas versuta	WP_060694962	----Q-KL----LHN	DD-M-R-TNI-AL---F--T--
	Pseudomonas viridiflava	WP_004886241	----I-KV----L-N	DD-Q-H-TNI-AI---F--T--
	Pseudomonas vranovensis	WP_028943232	----Q--F----L-N	ED-M-R-TNI-AL---F--T--
	Pseudomonas vranovensis	WP_028946189	----S--L---K---	AD-L---SNI-ALR-NFE-T--
	Pseudomonas wadenswilerensis	WP_115085392	----Q--F----L-N	DD-M-R-TNI-AL---F--T--
	Pseudomonas weihenstephanensis	WP_048363231	----Q-KF----L-N	DD---R-TNI-AL---F--T--
	Pseudomonas yamanorum	WP_003216288	----I-KL----L-N	DD-Q-R-TNI-AL---F--T--
	Stutzerimonas stutzeri	AZO84467	----I-KF----LHN	DD-Q-R-TNI-AL---F--T--
	Acinetobacter baumannii	SST10298	-------F----L-E	DS-Q-Q-TNI-AL--SFE-T--
	Alteromonas sediminis	WP_165870423	------TL-L--L-N	GD-L---VDI--LT-QG--TQ-
	Stenotrophomonas rhizophila	AXQ49657	----Q--L----L--	DD-M-R-TNI-AL---F--T--
**Other Bacteria**	*Priestia aryabhattai*	QPN46347	----Q--L----L--	DD-M-R-TNI-AL---F--T--
	Aeromonas caviae	GJB79127	----Q--F----L--	DD-M-R-TNI-AL---F--T--
	Escherichia coli	MRF39009	----Q--F----L--	DD-M-R-TNI-AL---F--T--
	Paucimonas lemoignei	SQG00114	----K-K-----L-N	DD-Q-H-TNI-----LF--T--
	Streptococcus dysgalactiae	VTS54730	-------F----L-E	DS-Q-Q-TNI-AL--SFE-T--

Figure-S86

Partial sequence alignments of Alginate export family protein showing a 2aa ins (highlighted), which is specific for the genus *Azotobacter*.

Group	Species	Accession	88		132
Genus *Azotobacter* (4/4)	*Azotobacter vinelandii*	WP_012702399	MDAKGYRNLTELVSRGWTEGQ	H	GDGLVIIQRDWVKEAAEGVIALS
	Azotobacter beijerinckii	WP_090620060	---------------------	-	------------------L----
	Azotobacter salinestris	WP_152388976	---------------------	-	-------R----R-----L----
	Azotobacter chroococcum	WP_089168891	---R-------------AD--	-	------------------L----
Other *Pseudomonadaceae*	*Azomonas agilis*	WP_144571762	-NPQ---H----I----I---		QNER-V-E-S--------L----
	Azomonas macrocytogenes	WP_183166640	-NTA--------I-----N--		RND----E---L----G-LVV--
	Oblitimonas alkaliphila	WP_053102450	-NNQ-------I--L-Y-Q--		HL-QMLV-KE--VANS--L---C
	Thiopseudomonas denitrificans	WP_101496282	---Q--------I----G---		HH-ISVL--E-IA-----L----
	Pseudomonas aeruginosa	WP_003098578	-N----------I----S---		RN-EI--E----------L----
	Pseudomonas guangdongensis	WP_090215606	--VQ-----------------		HN----------------L----
	Pseudomonas anguilliseptica	WP_090383265	-N---------------S---		SND---------------L----
	Pseudomonas peli	WP_090248374	-N---------------S---		SND---------------L----
	Pseudomonas sagittaria	WP_092430890	--GG--------I--------		HN-----------A----L----
	Pseudomonas linyingensis	WP_090307412	--GG-----------------		HN-----------A----L----
	Pseudomonas otitidis	WP_074971937	--G---------I----SD--		SN----------------L----
	Pseudomonas indica	WP_084337001	-N----------I-----D--		R---I--E----------L----
	Pseudomonas balearica	WP_043221036	---------------------		RNE----E-----A----L----
	Pseudomonas indoloxydans	WP_108233761	-N----------I----A---		SND---------------L----
	Pseudomonas pseudoalcaligenes	WP_004424028	-N----------I----A---		SND---------------L----
	Pseudomonas kuykendallii	WP_090226668	-NP---------------D--		SNDM--------------L----
	Pseudomonas kunmingensis	WP_090518991	-NP---------I--------		RND----E-----Q---------
	Pseudomonas sediminis	WP_099523459	-N----------I----S---		SND----------D----L----
	Pseudomonas benzenivorans	WP_090443234	-N-------------------		SND----------A----L----
	Pseudomonas toyotomiensis	WP_074918156	-N----------I----S---		SND----------D----L----
	Pseudomonas straminea	WP_093503860	-NP---------I----S---		SND------------S--L----
	Pseudomonas leptonychotis	WP_136662658	-N---------------S---		SND----------A----L----
	Pseudomonas azotifigens	WP_028239533	-N-R-----------------		RNEM---E----------L----
	Pseudomonas stutzeri	WP_013982337	-NP---------I--------		RND----E-E--------L----
	Pseudomonas chengduensis	WP_055985219	-N----------I----A---		SND----------D----L----
	Pseudomonas oryzae	WP_090348865	--EQ-----------------		LN--------------A-L----
	Pseudomonas hydrolytica	WP_041981058	-N----------I----A---		SND----------D----L----
	Pseudomonas cuatrocienegasensi	WP_069516081	-NP---------------D--		SND------E--------L----
	Pseudomonas chloritidismutans	WP_003299354	-NP------------------		RND----E-----QSS--L----
	Pseudomonas songnenensis	WP_126190005	-NP---------I--------		RND----E-----R----L----
	Pseudomonas pharmafabricae	WP_101192480	---G--------I--------		HND--V-----L-Q-SA-L----
	Pseudomonas fluvialis	WP_093985685	---G--------I--------		HND--V-----L-Q-SA-L----
	Pseudomonas guineae	WP_090238843	-N---------------S---		SND----------A----L----
	Pseudomonas composti	WP_074937036	-N----------I----S---		SND----------D-T--L----
	Pseudomonas seleniipraecipitan	WP_092365843	-NP--------------S---		SND----------A-S--L----
	Pseudomonas flavescens	WP_084304203	-NP--------------S---		SND----------A-S--L----
	Pseudomonas punonensis	WP_073261653	-NP--------------S---		SND----------A-S--L----
	Pseudomonas daroniae	WP_131190293	-NP--------------S---		SND----------A-S--L----
	Pseudomonas mendocina	WP_012019180	-NP---------I----A---		SND----------D----L----
	Pseudomonas alcaligenes	WP_061903641	-N----------I----A---		DN------------S---L----
	Pseudomonas resinovorans	WP_016494380	-S---------------S---		SN-----------Q-S-DL----
	Pseudomonas borbori	WP_090500262	-NP------------------		SNDQ---------A----L----
	Pseudomonas oleovorans	WP_150605824	-N----------I----S---		SND----------A--D-L----
	Pseudomonas fulva	WP_013792503	-NP---------I----S---		SND----------A-S--L----
	Pseudomonas sihuiensis	WP_092376594	-N----------I----A---		SND----------G----L----
	Pseudomonas alcaliphila	WP_075748722	-N----------I----A---		SND----------G----L----
	Pseudomonas argentinensis	WP_074881164	-NP--------------SD--		SND----------A-S--L----
	Pseudomonas segetis	WP_089359318	-N---------------S---		SND----------A-N--L----
	Pseudomonas tuomuerensis	WP_039606229	------L--------------		RN-M-V-E-------S--L----
	Pseudomonas flexibilis	WP_039560216	------L--------------		RN-M-V-E-------S--L----
	Pseudomonas khazarica	WP_134677474	-N----------I----S---		SND----------AS-D-L----
	Pseudomonas nosocomialis	WP_138408743	--NR-----------------		SNE----------G----L-C--
	Pseudomonas furukawaii	WP_004421091	-H-D-------------S---		SN-----------A--D-L----
	Pseudomonas guguanensis	WP_090431236	-NP---------I----A---		SND------E---D----L----
	Pseudomonas hydrolytica	WP_129484124	-NP---------I----A---		SND------E---D----L----
	Pseudomonas matsuisoli	WP_188981516	-N----------I----M---		SND------E---D----L----
	Pseudomonas dryadis	WP_131177985	-NP--------------S---		RNDQ---------A-S--L----
	Pseudomonas marincola	WP_090508891	-NG--------------S---		SND----------A----L----
	Pseudomonas denitrificans	WP_003098578	-N----------I----S---		RN-EI--E----------L----
	Pseudomonas taeanensis	WP_025165217	-NTL-----------------		SND----------G----L----
	Pseudomonas hussainii	WP_074865742	-NPE-------------SD--		SND-------------N-L----
	Pseudomonas saudiphocaensis	WP_037023298	-NTQ--------I--------		RND----E-----A-S--L----
	Pseudomonas kirkiae	WP_131184431	------------------D--		SNEM----PE--------L----
	Pseudomonas nitrititolerans	WP_170911426	-NGQ-------------A---		RND----E-----Q--D-L----
	Pseudomonas nitroreducens	WP_024766072	-N----------I----Q---		RN-EI--E-Q--------L----
	Pseudomonas nitritireducens	WP_024766072	-N----------I----Q---		RN-EI--E-Q--------L----
	Pseudomonas panipatensis	WP_090262884	-N----------I----SD--		RN-EI--E-E--------L----
	Pseudomonas jilinensis	WP_119701039	----------------F----		SN--STVR-E-IA--S-------
	Pseudomonas oryzihabitans	WP_059314057	-N----------L---FQ---		Q--QIV---------C--L----
	Pseudomonas jinjuensis	WP_084310044	-N-R-------MI----SD--		RN-EI--E-E--------L----
	Pseudomonas humi	WP_043271174	-N----------I----Q---		RN-EI--E----------L----
	Pseudomonas multiresinivorans	WP_169940910	-N----------I----Q---		RN-EI--E-Q---D----L----
	Pseudomonas thermotolerans	WP_017938812	-N-T---K-------------		RN---V-E-E----S---L----
	Pseudomonas rhizoryzae	WP_058772498	-N----------L---FQ---		QE-QI----------S--L----
	Pseudomonas psychrotolerans	WP_074529533	-N----------L---FQ---		QE-QI----------S--L----
	Pseudomonas psychrotolerans	WP_074529533	-N----------L---FQ---		QE-QI----------S--L----
	Pseudomonas xanthomarina	WP_073300829	-NSH-----------------		RNDQI--E-E---G--D-L----
	Pseudomonas duriflava	WP_145140107	----------------YQ---		R--RI--E-E---A-S--L----
	Pseudomonas xinjiangensis	WP_093394429	-NK-------------Y----		RN---T-R-E-IA--S-------
	Pseudomonas sabulinigri	WP_092288052	-NQR--------I---Y----		RN---TL--E-IA--S-------
	Pseudomonas citronellolis	WP_074985534	-N----------I----Q---		RN-EI--E------G---L----
	Pseudomonas japonica	WP_042121911	---T--------I----V---		RN-------E-IA-----L----
	Pseudomonas bauzanensis	WP_036990856	--RQ--------I---Y-H--		RN---T-R-E--A--S-------
	Pseudomonas massiliensis	WP_040261122	---H----I---I---YI---		RN--I--E-E-IA--SD------
	Pseudomonas asuensis	WP_188864246	-NKQ------------YQ---		HE-QI------I-A--D-L----
	Pseudomonas alkylphenolica	WP_038613978	------------I----V---		RN---V-E---IPA-S--L----
	Pseudomonas pachastrellae	WP_083724534	--P-------------F----		RN---TVR-E--A--S-------
	Pseudomonas gallaeciensis	WP_118130848	--P-------------F----		RN---TVR-E--A--S-------
	Pseudomonas abyssi	WP_096002957	--P-------------F----		RN---TVR-E--A--S-------
	Pseudomonas donghuensis	WP_010224829	------------I----VD--		RN-----E-P-IA--S--L----
	Pseudomonas delhiensis	WP_089394586	-N----------I----Q---		RN-EI--E---L-Q----L----

Other *Pseudomonadaceae*

Pseudomonas formosensis	WP_090536298	--NV----I-------Y----	RN---TVH----A--SA------
Pseudomonas knackmussii	WP_043249929	-N----------I----QH--	RN-EI--E-E--R-----L----
Pseudomonas taiwanensis	WP_023381961	-NP---------I--------	RN----L----IAP-S-------
Pseudomonas wadenswilerensis	WP_115088375	------------I----VD--	RN-----E-Q-IV--S--L----
Pseudomonas saudimassiliensis	WP_044498520	-NRQ--------I---Y-Q--	RN---T-R-E-IR-SS-------
Pseudomonas pohangensis	WP_090195797	-NG---------I---FI---	RN-E---E-----Q----L----
Pseudomonas litoralis	WP_090273788	-NRQ--------I---Y-H--	RN---T-R-E--A--S-------
Pseudomonas caeni	WP_028244322	-TPQ----------L-F-D--	HN--A-L------Q--T-L----
Pseudomonas profundi	WP_150300699	-NRP--------I---Y----	KN---T-R-E-IA--S-------
Pseudomonas luteola	WP_074828100	-NKQ------------YQ---	HE-QI-V--E---A--D-L----
Pseudomonas zeshuii	WP_037030045	-NKQ------------YQ---	HE-QI-V--E---A--D-L----
Pseudomonas vranovensis	WP_028943000	--P---------I----VD--	RN-----E---IAP-S--L----
Pseudomonas putida	WP_016501156	------------I-----D--	RN----L--E-IAP-S--L----
Pseudomonas soli	WP_023628728	-NP---------I-----D--	RN---------IAP-S--L----
Pseudomonas mosselii	WP_096049496	-NP---------I-----D--	RN---------IAP-S--L----
Pseudomonas entomophila	WP_011535265	-NP---------I-----D--	RN---------IAP-S--L----
Pseudomonas aestusnigri	WP_088273406	-NP-------------Y----	RN-V-TVR-E-IA--S-------
Pseudomonas pertucinogena	WP_188634857	-NNQ--------I---Y----	RN--ATVE----AA-S-------
Pseudomonas oceani	WP_104736978	-NP-------------Y----	RN-V-TVR-E-IA--S-------
Pseudomonas hunanensis	WP_103518110	--P---------I-----D--	RN----L--E-IAP-S--L----
Pseudomonas reidholzensis	WP_119144977	-NP---------I----VD--	RN---------IPP-S--L----
Pseudomonas guariconensis	WP_043208844	-NP---------I--------	RN-------E-IAP-S--L----
Pseudomonas ovata	WP_056834965	-NPQ--------I---F----	RN-F-V---E--AQ-S--L----
Pseudomonas brassicae	WP_163949094	---Q--------I----MD--	RN-V-V---E-IAP-S--L----
Pseudomonas piscis	WP_152896755	-N-V--------I---F----	RN--T--E----VQ----L-M--
Pseudomonas aestus	WP_022642587	-N-V--------I---F----	RN--T--E----VQ----L-M--
Pseudomonas plecoglossicida	WP_038408817	-NP---------I--------	RN----L--E-IAP-S--L----
Pseudomonas shirazica	WP_054573192	-NP---------I--------	RN----L--E-IAP-S--L----
Pseudomonas monteilii	WP_024086397	-NP---------I--------	RN----L--E-IAP-S--L----
Pseudomonas asiatica	WP_013971262	-NP---------I--------	RN----L--E-IAP-S--L----
Pseudomonas pudica	WP_046616626	-NP---------I--------	RN----L--E-IAP-S--L----
Pseudomonas juntendi	WP_161892720	-NP---------I-----D--	RN----L--E-IAP-S--L----
Pseudomonas salegens	WP_092384035	-NDR--------I---F----	RN--ATL--E-IAD-S--L----
Pseudomonas syringae	WP_024644398	-N-Q--------I---FI---	RN-QIV---Q--AQ-S-------
Pseudomonas caricapapayae	WP_055010685	-N-Q--------I---FI---	RN-QIV---Q--AQ-S-------
Pseudomonas avellanae	WP_005615998	-N-Q--------I---FI---	RN-QIV---Q--AQ-S-------
Pseudomonas meliae	WP_044344073	-N-Q--------I---FI---	RN-QIV---Q--AQ-S-------
Pseudomonas savastanoi	WP_004666023	-N-Q--------I---FI---	RN-QIV---Q--AQ-S-------
Pseudomonas amygdali	WP_004666023	-N-Q--------I---FI---	RN-QIV---Q--AQ-S-------
Pseudomonas congelans	WP_003415548	-N-Q--------I---FI---	RN-QIV---Q--AQ-S-------
Pseudomonas cannabina	WP_055001123	-N-Q--------I---FI---	RN-QIV---Q--AQ-S-------
Pseudomonas ficuserectae	WP_054996111	-N-Q--------I---FI---	RN-QIV---Q--AQ-S-------
Pseudomonas cerasi	WP_003433366	-N-Q--------I---FI---	RN-QIV---Q--AQ-S-------
Pseudomonas tremae	WP_054997860	-N-Q--------I---FI---	RN-QIV---Q--AQ-S-------
Pseudomonas coronafaciens	WP_024671822	-N-Q--------I---FI---	RN-QIV---Q--AQ-S-------
Pseudomonas pelagia	WP_022964025	-NRN--------I---Y----	RN-V-T-R-E--A-SS-D-----
Pseudomonas inefficax	WP_133970545	-NP---------I-----D--	RN----L--E-IAP-S--L----
Pseudomonas fuscovaginae	WP_019361572	-N-E--------I---YI---	RN--I--E-E--A---P-L-M--
Pseudomonas sichuanensis	WP_110992409	-NPQ--------I-----D--	RN-------E-IAP-S--L----
Pseudomonas atacamensis	WP_136492047	-N-Q--------I---FI---	RN-M--VE-E--A--S--L-M--
Pseudomonas helmanticensis	WP_038357916	-N----------I---FI---	RN-MI-VE-E--A--N--L-M--
Pseudomonas yangmingensis	WP_093473388	--QQ--------I---F----	RN--ATL--E-LAS-SD-L---C
Pseudomonas baetica	WP_100847334	-N----------I---FI---	RN-MI--E-E--A--N--L-M--
Pseudomonas qingdaonensis	WP_058541490	-N----------I----MD--	RN-V-V-E-E-IAP-S--L----
Pseudomonas asplenii	WP_090201977	-N-E--------I---YMD--	RN--IV-E-E--A---Q-L-M--
Pseudomonas granadensis	WP_090282011	-N----------I---FI---	RN-MI-VE-Q--A--S--L-M--
Pseudomonas typographi	WP_190417990	-NRE----I---I---YL---	QG-Q-VL--A--A--SA------
Pseudomonas koreensis	WP_041478124	-N-Q--------I---FI---	RN-M--VE-E--A--N--L-M--
Pseudomonas cremoricolorata	WP_038412351	-NPE--------I----V---	RN---------IAP-SA-L-G--
Pseudomonas floridensis	WP_083183487	-N-Q--------I---FI---	RN-QIV---E--AQ-S--L----
Pseudomonas caspiana	WP_087273925	-N----------I---FID--	RN-QI--E-E--A--N--L----
Pseudomonas salina	WP_150277731	-NRN--------I---Y----	RN-V-T-R-E--A-SS-D-----
Pseudomonas parafulva	WP_028634994	-NPQ--------I-----D--	RN----L--E-IAP-S--L----
Pseudomonas cichorii	WP_025258959	-NPQ--------I---FI---	RN--I-VE-E-IA--N--L----
Pseudomonas moraviensis	WP_083352100	-N-Q--------I---FI---	RN-M--VE-E--A--N--L-M--
Pseudomonas endophytica	WP_055102569	-N-V--------I---FID--	RN-Q---E-E--A-----L-M--
Pseudomonas saxonica	WP_146385272	-NPL--------I---YI---	RN-Q---E-E--A-----L-M--
Pseudomonas capeferrum	WP_033700870	-NPT--------I----V---	RN----L--E-IAP-S--L----
Pseudomonas viridiflava	WP_004879041	-N-Q--------I---FI---	RN-QIV---E--AQ-SD-L----
Pseudomonas asturiensis	WP_073164037	-N-Q--------I---FI---	RN-QIV---E--AQ-SD-L----
Entomomonas moraniae	WP_127164536	-NE---------I-Q-YEN--	HE-M---EKE-LIQ-N--L-V--
Pseudomonas rhizosphaerae	WP_043185107	-N-V----I---I---FI---	RN-Q--VE-Q--A--S----C--
Pseudomonas lutea	WP_037014226	-NP---L-----I---FMH--	RN---V-E-E--A--S--L----
Pseudomonas graminis	WP_074891288	-NP---L-----I---FMH--	RN---V-E-E--A--S--L----
Pseudomonas bohemica	WP_110951331	-NP---L-----I---FMD--	RN-----D-E--A--S--L----
Pseudomonas migulae	WP_084322282	-N-V--------I---FID--	RN-S---E-E--A--S--L-M--
Pseudomonas arsenicoxydans	WP_090187801	-N-V--------I---FID--	RN-S---E-E--A--S--L-M--
Pseudomonas silesiensis	WP_064676114	-N-V--------I---FID--	RN-S---E-E--A--S--L-M--
Pseudomonas helleri	WP_048368084	-N-V--------I---FID--	RN-Q---E-E--A---D-L-M--
Pseudomonas abietaniphila	WP_074752722	-NP---L-----I---FMH--	RN---V-E-E--A--S--L----
Pseudomonas versuta	WP_060694726	-NGV--------I---FID--	RN-Q---E-E--A-----L-M--
Pseudomonas orientalis	WP_057723193	-NGV--------I---FI---	RN-SI--E-E--A--S----M--
Pseudomonas coleopterorum	WP_090359747	-N-V----I---I---FID--	RN-Q--VE-Q--A--S----C--
Pseudomonas taetrolens	WP_048378890	-NGA--------I---FID--	RN-Q---E-E--A-----L-M--
Pseudomonas veronii	WP_046384461	-NGV--------I---FID--	RN-A---E-E--A--S----M--
Pseudomonas panacis	WP_046384461	-NGV--------I---FID--	RN-A---E-E--A--S----M--
Pseudomonas lini	WP_038979156	-N-V--------I---FID--	RN-SI--E-E--A--S--L-M--
Pseudomonas lundensis	WP_047282991	-NGV--------I---FID--	RN-Q---E-E--A-----L-M--
Pseudomonas gingeri	WP_017127132	-NPL--------I---FID--	RN-MI--E-E--A--N--L-M--
Pseudomonas protegens	WP_015634337	-N-V--------I---FID--	RN-QI--E-E--A-----L-M--
Pseudomonas deceptionensis	WP_048359896	-NGV--------I---FID--	RN-Q---E-E--A-----L-M--
Pseudomonas saponiphila	WP_092320045	-N-V--------I---FID--	RN-QI--E-E--A-----L-M--
Pseudomonas chlororaphis	WP_009047221	-N-V--------I---FID--	RN-QI--E-E--A-----L-M--
Pseudomonas bubulae	WP_095001318	-NPL--------I---YID--	RN-Q---E-E--A---D-L-M--
Pseudomonas fragi	WP_083369605	-NPL--------I---YID--	RN-Q---E-E--A---D-L-M--
Pseudomonas fluorescens	WP_053254747	-NGV--------I---FID--	RN-SI--E-E--A--S----M--
Pseudomonas libanensis	WP_057012051	-NGV--------I---FID--	RN-SI--E-E--A--S----M--

Other *Pseudomonadacea*	*Pseudomonas antarctica*	WP_064451161	-NGV--------I---FID--	RN-SI--E-E--A--S----M--
	Pseudomonas cedrina	WP_076950032	-NGV--------I---FID--	RN-SI--E-E--A--S----M--
	Pseudomonas synxantha	WP_057021919	-NGV--------I---FID--	RN-SI--E-E--A--S----M--
	Pseudomonas costantinii	WP_071487441	-NGV--------I---FID--	RN-SI--E-E--A--S----M--
	Pseudomonas batumici	WP_040064774	-N-V--------I---FID--	RN-MI--E-E--A--N--L-M--
	Pseudomonas salomonii	WP_065928981	-NGV--------I---FID--	RN-SI--E-E--A--S----M--
	Pseudomonas edaphica	WP_017137152	-NGV--------I---FID--	RN-SI--E-E--A--S----M--
	Pseudomonas frederiksbergensis	WP_071551630	-N-V--------I---FID--	RN-QI--E-E--A--S--L-M--
	Pseudomonas mandelii	WP_010462801	-N-V--------I---FID--	RN-S---E-E--A--S--L-M--
	Pseudomonas umsongensis	WP_020799856	-N-A--------I---FI---	RN-SI--E-E--A--SD-L-M--
	Pseudomonas vancouverensis	WP_093214918	-N-Q--------I---FID--	RN-SI--E-E--A--N--L-M--
	Pseudomonas kitaguniensis	WP_058412412	-NGV--------I---FID--	RN-SI--E-E--A--S----M--
	Pseudomonas laurylsulfatiphila	WP_007978370	-N-E--------I---FI---	RN-AI--E-E--A--SA-L-M--
	Pseudomonas kribbensis	WP_114881643	-N-L--------I---FID--	RN-MI--E-E--V--N--L-M--
	Pseudomonas weihenstephanensis	WP_048363365	-NGV--------I---FID--	RN-Q---E-E--A--S--L-M--
	Pseudomonas mucidolens	WP_084377156	-SGV--------I---FI---	RN-SI--E-E--A--S--L-M--
	Pseudomonas thivervalensis	WP_053118870	-N-V--------I---FID--	RN-SI--E-E--A--S--L-M--
	Pseudomonas mediterranea	WP_047702000	-N-V--------I---FID--	RN-SI--E-E--A--S--L-M--
	Pseudomonas corrugata	WP_024779025	-N-V--------I---FID--	RN-SI--E-E--A--S--L-M--
	Pseudomonas kilonensis	WP_024619347	-N-L--------I---FID--	RN-SI--E-E--A--S--L-M--
	Pseudomonas brassicacearum	WP_025212124	-N-L--------I---FID--	RN-SI--E-E--A--S--L-M--
	Pseudomonas psychrophila	WP_048351382	-NPL--------I---YID--	RN-Q---E-E--A---A-L-M--
	Pseudomonas haemolytica	WP_034118461	-NGV--------I---FID--	RN-SI--E-E--AQ-S----M--
	Pseudomonas poae	WP_003231210	-NGV--------I---FID--	RN-SI--E-E--AQ-S----M--
	Pseudomonas canadensis	WP_028619210	-NGV--------I---FID--	RN-SI--E-E--A--S--L-M--
	Pseudomonas yamanorum	WP_063032894	-NGV--------I---FID--	RN-SI--E-E--A--S--L-M--
	Pseudomonas trivialis	WP_049709657	-NGV--------I---FID--	RN-SI--E-E--A--S--L-M--
	Pseudomonas prosekii	WP_092272422	-NGV--------I---FID--	RN-SI--E-E--A--S--L-M--
	Pseudomonas carnis	WP_003189180	-NGV--------I---FID--	RN-SI--E-E--A--S--L-M--
	Pseudomonas nabeulensis	WP_105696259	-NGV--------I---FID--	RN-SI--E-E--A--S--L-M--
	Pseudomonas marginalis	WP_046036638	-NGV--------I---FID--	RN-SI--E-E--A--S--L-M--
	Pseudomonas sivasensis	WP_032891643	-NGV--------I---FID--	RN-SI--E-E--A--S--L-M--
	Pseudomonas lactis	WP_032893179	-NGV--------I---FID--	RN-SI--E-E--A--S--L-M--
	Pseudomonas grimontii	WP_090408486	-NGV--------I---FID--	RN-SI--E-E--A--S--L-M--
	Pseudomonas proteolytica	WP_029297262	-NGV--------I---FID--	RN-SI--E-E--A--S--L-M--
	Pseudomonas lurida	WP_034109083	-NGV--------I---FID--	RN-SI--E-E--A--S--L-M--
	Pseudomonas palleroniana	WP_060754048	-NGV--------I---FID--	RN-SI--E-E--A--S--L-M--
	Pseudomonas extremorientalis	WP_071488805	-NGV--------I---FID--	RN-SI--E-E--A--S--L-M--
	Pseudomonas simiae	WP_042570667	-NGV--------I---FID--	RN-SI--E-E--A--S--L-M--
	Pseudomonas rhodesiae	WP_034096250	-NGV--------I---FID--	RN-SI--E-E--A--S--L-M--
	Pseudomonas cremoris	WP_185707380	-NGV--------I---FID--	RN-SI--E-E--A--S--L-M--
	Pseudomonas tolaasii	WP_016974414	-NGV--------I---FID--	RN-SI--E-E--A--S--L-M--
	Pseudomonas reactans	WP_177003311	-NGV--------I---FID--	RN-SI--E-E--A--S--L-M--
	Pseudomonas extremaustralis	WP_010563531	-NGV--------I---FID--	RN-SI--E-E--A--S--L-M--

Figure-S87

Partial sequence alignments of DNA polymerase III subunit alpha protein showing a 1aa ins (highlighted), which is specific for the genus *Azotobacter*.

Group	Species	Accession	238		276
**Genus *Azotobacter* (4/4)**	*Azotobacter salinestris*	WP_152387189	GSEMGHLPQSGAGGMLEVL		DGLDGRKVLIHINNTNPIL
	Azotobacter vinelandii	WP_012702669	-------------------		-------------------
	Azotobacter chroococcum	WP_089167280	-------------------		---R---------------
	Azotobacter beijerinckii	WP_090623842	---------R---------		---R---------------
**Other *Pseudomonadaceae***	*Azomonas macrocytogenes*	WP_183167386	--A------T-P-------	E	KLPRQ--------------
	Thiopseudomonas denitrificans	WP_101496339	-RQ--------E-----T-	A	QFPQR-H------------
	Pseudomonas aeruginosa	WP_003106464	-RQ----A---P-------	A	KVPAA--------------
	Pseudomonas panipatensis	WP_090259563	-------A-----------	D	GLPGP--------------
	Pseudomonas jinjuensis	WP_084315303	-----------P---I---	D	GIQGP--------------
	Pseudomonas furukawaii	WP_003455957	-----------P---I---	D	GIPGP--------------
	Pseudomonas otitidis	WP_074968559	-----------P---I---	D	GIPGP--------------
	Pseudomonas hydrolytica	WP_129482321	-----------P-------	D	GLPTA--I-----------
	Pseudomonas multiresinivorans	WP_169939064	-------A---P---I---	D	GIDGP--------------
	Pseudomonas mandelii	WP_010455121	-------------------	E	GFTRQ--------------
	Pseudomonas balearica	WP_043219975	-----------P---I---	D	GVPGK--I-----------
	Pseudomonas borbori	WP_090505610	-----------P---I---	D	GLPGKQ-I-----------
	Pseudomonas nitrititolerans	WP_170910216	-----------P---I---	D	GLPAA--I-----------
	Pseudomonas kuykendallii	WP_090231222	-----------E-----L-	D	GLAAP--------------
	Pseudomonas nitritireducens	WP_193452534	-------A---P--II---	D	GIDGP--------------
	Pseudomonas jessenii	WP_090454971	-----------P-------	E	GFNRQ--------------
	Pseudomonas hydrolytica	WP_129482231	-----------P-------	E	GLPAA--I-----------
	Pseudomonas migulae	WP_084320895	-----------P-------	E	GFSRQ--------------
	Pseudomonas nitroreducens	WP_084357815	-------A---P---I---	D	SIDGP--------------
	Pseudomonas sihuiensis	WP_092375739	-----------P-------	E	GLPAA--I-----------
	Pseudomonas resinovorans	WP_016493009	--A--------P---I---	D	GIPGP--------------
	Pseudomonas delhiensis	WP_089392308	-------A---P-------	D	GVPGP--------------
	Pseudomonas citronellolis	WP_043271909	-------A---P-------	D	GVPGP--------------
	Pseudomonas humi	WP_069864459	-------A---P-------	D	GVPGP--------------
	Pseudomonas moorei	WP_090320639	-----------P-------	E	GFPRQ--------------
	Pseudomonas oryzae	WP_090351893	-------------------	E	RLPRQ--------------
	Pseudomonas kirkiae	WP_131185536	-----------R---I---	E	GFPEQ--------------
	Pseudomonas lini	WP_048394650	-----------P----D--	E	GFNRQ--------------
	Pseudomonas benzenivorans	WP_090446942	-----------P---I-I-	D	GLPAKQ-I-----------
	Pseudomonas laurylsulfatiphila	WP_104447408	-----------P----D--	E	GFNRQ--------------
	Pseudomonas guangdongensis	WP_090214700	-----------E-------	E	RLPEP--------------
	Pseudomonas indica	WP_084334952	-----------P---IS--	D	GLPAP--I---V-------
	Pseudomonas toyotomiensis	WP_074916624	-------C---P---I---	D	GLPSA--------------
	Pseudomonas cuatrocienegasensi	WP_069521991	-------C---P---I---	D	GLPSA--------------
	Pseudomonas composti	WP_074941229	-------C---P---I---	D	GLPSA--------------
	Pseudomonas oryzihabitans	WP_059316330	-T---------P-------	A	GFERQ--------------
	Pseudomonas alcaliphila	WP_075748164	-------C---P---I---	D	GLSSA--I-----------
	Pseudomonas rhizoryzae	WP_058774338	-T---------P-------	A	GFERQ--------------
	Pseudomonas sihuiensis	WP_017676587	-------C---P---I---	D	GLPSA--I-----------
	Pseudomonas chengduensis	WP_017676587	-------C---P---I---	D	GLPSA--I-----------
	Pseudomonas psychrotolerans	WP_074528686	-T---------P-------	A	GFERQ--------------
	Pseudomonas psychrotolerans	WP_074528686	-T---------P-------	A	GFERQ--------------
	Pseudomonas sediminis	WP_099523005	-------C---P---I---	D	GLPSA--I-----------
	Pseudomonas hydrolytica	WP_041981014	-------C---P---I---	D	GLPSA--I-----------
	Pseudomonas peli	WP_090252832	-------C---P---I---	D	GLPSA--I-----------
	Pseudomonas linyingensis	WP_090310490	--A----------------	E	RLPKQ--------------
	Pseudomonas oleovorans	WP_134678045	-------C---P-------	E	GLPAA--------------
	Pseudomonas khazarica	WP_134678045	-------C---P-------	E	GLPAA--------------
	Pseudomonas umsongensis	WP_033041442	--D--------P-------	Q	GFPRQ--------------
	Pseudomonas sagittaria	WP_092432124	--A----------------	E	RLPRP--------------
	Pseudomonas pseudoalcaligenes	WP_003461200	-------C---P---I---	D	GLPSV--I-----------
	Pseudomonas reidholzensis	WP_119137686	-R-----A-N-P-------	D	GFDRQ--------------
	Pseudomonas kunmingensis	WP_090520069	-------Q---P---I---	D	GMPAV--I-----------
	Pseudomonas stutzeri	WP_014596673	-------Q---P---I---	D	GMPAV--I-----------
	Pseudomonas chloritidismutans	WP_023446621	-------Q---P---I---	D	GMPAV--I-----------
	Pseudomonas flavescens	WP_084308982	-R-----A-N---------	D	GFPRQ--------------
	Pseudomonas matsuisoli	WP_188982886	-Q-----A---P---I---	D	GLSKP--------------
	Pseudomonas soli	WP_094011523	-R-----A-N---------	D	GFPRQ--------------
	Pseudomonas japonica	WP_042128770	-R---------P-------	E	HLPAR--------------
	Pseudomonas guguanensis	WP_090430593	-------C---P---I-L-	D	GLPTA--I-----------
	Pseudomonas mendocina	WP_012018421	-------C---P---I-L-	D	GLPTA--I-----------
	Pseudomonas songnenensis	WP_126188918	-------Q---L---I---	D	GMPAV--I-----------
	Pseudomonas zhaodongensis	WP_128121420	-------H---P---I---	D	GMPAA--I-----------
	Pseudomonas litoralis	WP_090272861	-----------E---I---	N	AYPES-R------------
	Pseudomonas indoloxydans	WP_108232738	-------C---P---I---	N	GLPSA--I-----------
	Pseudomonas cannabina	WP_054999110	-R-----A-N-P-------	E	GFP-Q--------------
	Pseudomonas massiliensis	WP_040259752	-R-----A-N-P-------	D	GLNVP--------------
	Pseudomonas qingdaonensis	WP_054915970	-R-----A-N-P-------	E	GFP-Q--------------
	Pseudomonas taeanensis	WP_025167780	-------C---P---I---	D	GLPAAQ-I-----------
	Pseudomonas laurylsulfatovoran	WP_103393710	-------A---P----D--	E	GFNRQ--------------
	Pseudomonas japonica	WP_042123970	---------N-P-------	E	-FPTQ--------------
	Pseudomonas knackmussii	WP_043252516	-------A---P------F	E	RVG-S--------------
	Pseudomonas arsenicoxydans	WP_090184801	-----------P-----LF	E	QFPRQ--------------
	Pseudomonas xanthomarina	WP_073299788	-------Q---P---I---	D	SMPAA--I-----------
	Pseudomonas cichorii	WP_025262193	-R-----A-N---------	E	GFTKQ--------------
	Pseudomonas ovata	WP_109512475	-R-----A---P-------	E	GFPQQ--------------
	Pseudomonas aestus	WP_022643639	-------A---P-------	E	AFPRA--------------
	Pseudomonas guariconensis	WP_043210355	-R-----A-N-P-------	D	GFSRQ--------------
	Pseudomonas luteola	WP_074821793	-R-----A---P-------	D	QLTSP--------------
	Pseudomonas mosselii	WP_096049203	-R-----A-N-T-------	D	GFPRQ--------------
	Pseudomonas thermotolerans	WP_017938757	-----------P-----E-	A	-LPAA--------------
	Pseudomonas mohnii	WP_090468839	--D----S---P-------	E	GFPRQ--------------
	Pseudomonas tremae	WP_054997535	-R-----A---P-------	E	GFPQQ--------------
	Pseudomonas coronafaciens	WP_053931832	-R-----A---P-------	E	GFPQQ--------------
	Pseudomonas argentinensis	WP_074882248	-R-----A-N-P-------	D	GFARQ--------------
	Pseudomonas juntendi	WP_110679033	-R-----A-N-P-------	D	GFPRQ--------------
	Pseudomonas taiwanensis	WP_023382702	-R-----A-N-P-------	D	GFPRQ--------------
	Pseudomonas hunanensis	WP_003255590	-R-----A-N-P-------	D	GFPRQ--------------
	Pseudomonas capeferrum	WP_033703075	-R-----A-N-P-------	D	GFPRQ--------------
	Pseudomonas sichuanensis	WP_110994306	-R-----A-N-P-------	D	GFPRQ--------------
	Pseudomonas putida	WP_016497684	-R-----A-N-P-------	D	GFPRQ--------------

Other *Pseudomonadaceae*

Pseudomonas entomophila	WP_011531815	-R-----A-N-P-------	D	GFPRQ--------------
Pseudomonas saponiphila	WP_092316034	-------A---P-------	E	RFPRA--------------
Pseudomonas asiatica	WP_015268707	-R-----A-N-P-------	D	GFPRQ--------------
Pseudomonas protegens	WP_015635022	-------A---P-------	E	RFPRA--------------
Pseudomonas shirazica	WP_024086143	-R-----A-N-P-------	D	GFPRQ--------------
Pseudomonas plecoglossicida	WP_024086143	-R-----A-N-P-------	D	GFPRQ--------------
Pseudomonas monteilii	WP_024086143	-R-----A-N-P-------	D	GFPRQ--------------
Pseudomonas pertucinogena	WP_188636981	-----------E---I---	S	AYPEA-R--T---------
Pseudomonas inefficax	WP_133970042	-R-----A-N-P-------	D	GFPRQ--------------
Pseudomonas pudica	WP_085600082	-R-----A-N-P-------	D	GFPRQ--------------
Pseudomonas alkylphenolica	WP_038605989	-R-----A-N-P-------	E	GFPEQ--------------
Pseudomonas savastanoi	WP_004654742	-R-----A-N-P-------	E	GFPEQ--------------
Pseudomonas meliae	WP_002555560	-R-----A-N-P-------	E	GFPEQ--------------
Pseudomonas congelans	WP_032611800	-R-----A-N-P-------	E	GFPEQ--------------
Pseudomonas syringae	WP_025388761	-R-----A-N-P-------	E	GFPEQ--------------
Pseudomonas ficuserectae	WP_054996791	-R-----A-N-P-------	E	GFPEQ--------------
Pseudomonas caricapapayae	WP_054985654	-R-----A-N-P-------	E	GFPEQ--------------
Pseudomonas amygdali	WP_005735079	-R-----A-N-P-------	E	GFPEQ--------------
Pseudomonas cerasi	WP_058416069	-R-----A-N-P-------	E	GFPEQ--------------
Pseudomonas parafulva	WP_028633642	-R-----A-N-S-------	D	AFPRQ--------------
Pseudomonas mediterranea	WP_047702307	-R-----A-N-P-------	E	QLPGP--------------
Pseudomonas rhizosphaerae	WP_043192435	-R-----A-N-P-------	E	GLPRQ--------------
Pseudomonas duriflava	WP_145141123	-R-----A-N-P-------	D	QLTSP--------------
Pseudomonas vranovensis	WP_028942174	-R-----A-N-P-------	E	GFPAQ--------------
Pseudomonas laurylsulfatovoran	WP_103396639	-R-----A-N-P-------	E	QLPGQ--------------
Pseudomonas zeshuii	WP_010795929	-R-----A-N-P-------	D	QLTSP--------------
Pseudomonas corrugata	WP_024779400	-R-----A-N-P-------	E	QLPGP--------------
Pseudomonas coleopterorum	WP_056845285	-R-----A-N-P-------	E	GLPRQ--------------
Pseudomonas asuensis	WP_188866715	-R-----A-N-P-------	D	QLTSP--------------
Pseudomonas versuta	WP_060696699	-R-----A-N-P-------	E	QLPGQ--------------
Pseudomonas piscis	WP_152897210	-------A---P-------	E	AFPRA------------T-
Pseudomonas bauzanensis	WP_074781195	-----------P---I---	S	AYPQS-R--T---------
Pseudomonas formosensis	WP_090539957	-----------E---I---	S	AYPQS-R--T---------
Pseudomonas jessenii	WP_090451766	-R-----A---P-------	E	QLPNQ--------------
Pseudomonas saudimassiliensis	WP_044499594	-----------E---I---	S	AYPQS-R--T---------
Pseudomonas saudiphocaensis	WP_037023061	------M--I-P---I-L-	D	EMPAS--I-----------
Pseudomonas asturiensis	WP_073167035	-R-----A-N-P-------	E	GFPTQ--------------
Pseudomonas cremoricolorata	WP_038411148	-T-----A-H-P-------	E	GFPRQ--------------
Pseudomonas pachastrellae	WP_083724654	-------Q---P---I-L-	D	AQPAS--------------
Pseudomonas oceani	WP_104737120	-------Q---P---I-L-	D	AQPAS--------------
Pseudomonas aestusnigri	WP_088277984	-------Q---P---I-L-	D	AQPAS--------------
Pseudomonas abyssi	WP_096006505	-------Q---P---I-L-	D	AQPAS--------------
Pseudomonas sabulinigri	WP_092288260	-------Q---P---I-L-	D	AQPAS--------------
Pseudomonas gallaeciensis	WP_118130526	-------Q---P---I-L-	D	AQPAS--------------
Pseudomonas azotoformans	WP_033898097	-R-----A-N-P-------	E	KLPEQ--------------
Pseudomonas kilonensis	WP_053189961	-R-----A-N-P-------	E	QVPGP--------------
Pseudomonas thivervalensis	WP_053125962	-R-----A-N-P-------	E	QLPEQ--------------
Pseudomonas kribbensis	WP_064599867	-R-----A-N-P-------	E	QLPEQ--------------
Pseudomonas endophytica	WP_055104766	-R-----A-N-P-------	E	QLPEQ--------------
Pseudomonas agarici	WP_060781913	-Q-----A-N-P-------	A	QLPGP--------------
Pseudomonas nabeulensis	WP_135306925	-R-----A-N-P-------	E	QLPEQ--------------
Pseudomonas granadensis	WP_090286881	-R-----A-N-P-------	E	QLPEQ--------------
Pseudomonas baetica	WP_100846462	-R-----A-N-P-------	E	QLPEQ--------------
Pseudomonas koreensis	WP_041476710	-R-----A-N-P-------	E	QLPEQ--------------
Pseudomonas atacamensis	WP_016772875	-R-----A-N-P-------	E	QLPEQ--------------
Pseudomonas cremoris	WP_185709262	-R-----A-N-P-------	E	QLPEQ--------------
Pseudomonas reactans	WP_177002712	-R-----A-N-P-------	E	QLPEQ--------------
Pseudomonas moraviensis	WP_065616547	-R-----A-N-P-------	E	QLPEQ--------------
Pseudomonas graminis	WP_065991311	-T-----A-N-P-------	E	KLPQQ--------------
Pseudomonas extremaustralis	WP_010566321	-R-----A-N-P-------	E	QLPEQ--------------
Pseudomonas tolaasii	WP_016972366	-R-----A-N-P-------	E	QLPEQ--------------
Pseudomonas salomonii	WP_069787027	-R-----A-N-P-------	E	QLPEQ--------------
Pseudomonas fuscovaginae	WP_029530374	-R-----A-N-P-------	E	QLPAQ--------------
Pseudomonas extremorientalis	WP_058424746	-R-----A-N-P-------	E	QLPEQ--------------
Pseudomonas allii	WP_058424746	-R-----A-N-P-------	E	QLPEQ--------------
Pseudomonas sivasensis	WP_032883902	-R-----A-N-P-------	E	QLPEQ--------------
Pseudomonas poae	WP_060548204	-R-----A-N-P-------	E	QLPEQ--------------
Pseudomonas costantinii	WP_071484865	-R-----A-N-P-------	E	QLPEQ--------------
Pseudomonas lundensis	WP_047283190	-R-----A-N-P-------	E	QLPEQ--------------
Pseudomonas trivialis	WP_049711248	-R-----A-N-P-------	E	QLPEQ--------------
Pseudomonas taetrolens	WP_048380783	-R-----A-N-P-------	E	QLPEQ--------------
Pseudomonas fildesensis	WP_048722109	-R-----A-N-P-------	E	QLPEQ--------------
Pseudomonas deceptionensis	WP_048361138	-R-----A-N-P-------	E	QLPEQ--------------
Pseudomonas bubulae	WP_048361138	-R-----A-N-P-------	E	QLPEQ--------------
Pseudomonas asplenii	WP_090210923	-R-----A-N-P-------	E	QLPAQ--------------
Pseudomonas saxonica	WP_146385976	-R-----A-N-P-------	E	QLPEQ--------------
Pseudomonas helleri	WP_048372265	-R-----A-N-P-------	E	QLPEQ--------------
Pseudomonas brassicacearum	WP_025215817	-Q-----A-N-P-------	E	QLPNQ--------------
Pseudomonas lutea	WP_037019096	-T-----A-N-P-------	E	RLPQQ--------------
Pseudomonas gingeri	WP_017128718	-R-----A-N-P-------	E	QLPAP--------------
Pseudomonas bohemica	WP_110951568	-T-----A-N-P-------	E	KLPRQ--------------
Pseudomonas abietaniphila	WP_062385268	-T-----A-N-P-------	E	KLPRQ--------------
Pseudomonas chlororaphis	WP_038636349	-R-----A-N-P-------	E	QLPEQ--I-----------
Pseudomonas psychrophila	WP_019823040	-R-----A-N-P-------	E	QLPAQ--------------
Pseudomonas avellanae	WP_005614034	-R-----A-N-P----D--	K	GFPEQ--------------
Pseudomonas caspiana	WP_087268582	-T-----A-N-P-------	E	HLPKQ--------------
Pseudomonas lini	WP_048397792	-R-----A-N-P-------	E	QLPKQ--------------
Pseudomonas typographi	WP_190416611	-R-----A-H-P----D--	E	GLPTP--------------
Pseudomonas helmanticensis	WP_134178016	-R-----A-N-P-------	E	QLPKQ--------------
Pseudomonas umsongensis	WP_020798846	-R-----A-N-P-------	E	QLPKQ--------------
Pseudomonas weihenstephanensis	WP_048364209	-R-----A-N-P-------	E	QLPKQ--------------
Pseudomonas rhodesiae	WP_034139564	-R-----A-N-P-------	E	QLPQQ--------------
Pseudomonas arsenicoxydans CEC	WP_090179050	-R-----A-N-P-------	E	QLPKQ--------------
Pseudomonas prosekii	WP_092277222	-R-----A-N-P-------	E	QLPKQ--------------
Pseudomonas mucidolens	WP_084379576	-R-----A-N-P-------	E	QLPKQ--------------
Pseudomonas silesiensis	WP_064680122	-R-----A-N-P-------	E	QLPKP--------------
Pseudomonas kairouanensis	WP_135289032	-R-----A-N-P-------	E	QLPKQ--------------

Other *Pseudomonadaceae*

Pseudomonas fluorescens	WP_053258333	-R-----A-N-P------- E QLPKQ--------------
Pseudomonas caeni	WP_022965001	-------A---P-C----- A -YPNA-RI-----------
Pseudomonas canadensis	WP_028618179	-R-----A-N-P------- E QLPKQ--------------
Pseudomonas kitaguniensis	WP_152746268	-R-----A-N-P------- E QLPKQ--------------
Pseudomonas piscis	WP_152899547	-R-----A-N-P------- E QLPRQ--------------
Pseudomonas grimontii	WP_090401792	-R-----A-N-P------- E QLPKQ--------------
Pseudomonas carnis	WP_034128837	-R-----A-N-P------- E QLPKQ--------------
Pseudomonas antarctica	WP_064454408	-R-----A-N-P------- E QLPKQ--------------
Pseudomonas aestus	WP_022641450	-R-----A-N-P------- E QLPRQ--------------
Pseudomonas viridiflava	WP_029244088	-R-----A-N-P----D-- E GFPTQ--------------
Pseudomonas simiae	WP_010207003	-R-----A-N-P------- E QLPKQ--------------
Pseudomonas paralactis	WP_057702243	-R-----A-N-P------- E QLPKQ--------------
Pseudomonas mandelii	WP_010467139	-R-----A-N-P------- E QLPKQ--------------
Pseudomonas haemolytica	WP_010207003	-R-----A-N-P------- E QLPKQ--------------
Pseudomonas edaphica	WP_010207003	-R-----A-N-P------- E QLPKQ--------------
Pseudomonas saponiphila	WP_092309233	-Q-----A-N-P------- E QLPRQ--------------
Pseudomonas libanensis	WP_057013932	-R-----A-N-P------- E QLPKQ--------------
Pseudomonas yamanorum	WP_093204386	-R-----A-N-P------- E QLPKQ--------------
Pseudomonas synxantha	WP_057022937	-R-----A-N-P------- E QLPKQ--------------
Pseudomonas lactis	WP_057710295	-R-----A-N-P------- E QLPKQ--------------
Pseudomonas fragi	WP_016782462	-R-----A-N-P------- E QLPQQ--------------
Pseudomonas orientalis	WP_057723650	-R-----A-N-P------- E QLPKQ--------------
Pseudomonas vancouverensis	WP_093229502	-R-----A-N-P------- E QLPRQ--------------
Pseudomonas protegens	WP_011063849	-R-----A-N-P------- E QLPRQ--------------
Pseudomonas migulae	WP_084318406	-R-----A-N-P------- E KLPRQ--------------
Pseudomonas gessardii	WP_076962250	-R-----A-N-P------- E QLPRQ--------------
Pseudomonas moorei	WP_090325737	-R-----A-N-P------- E QLPTQ--------------
Pseudomonas mohnii	WP_047534191	-R-----A-N-P------- E QLPTQ--------------
Pseudomonas laurylsulfatiphila	WP_104450014	-R-----A-N-P------- E QLPTQ--------------
Pseudomonas donghuensis	WP_010221422	-R-----A-N-P------- E SFPRQ--------------
Pseudomonas lini	WP_048392900	-R-----A-N-P------- E HLPKQ--------------
Pseudomonas cedrina	WP_076953287	-R-----A-N-P------- E QLPRQ--------------
Pseudomonas veronii	WP_017847831	-R-----A-N-P------- E HLPKQ--------------
Pseudomonas panacis	WP_017847831	-R-----A-N-P------- E HLPKQ--------------
Pseudomonas wadenswilerensis	WP_115085011	-R-----A-N-P------- E SFPRQ--------------
Pseudomonas frederiksbergensis	WP_071553092	-R-----A-H-P------- E QLPKQ--------------
Pseudomonas palleroniana	WP_060757261	-R-----A-N-P------- A QLPEQ--------------
Pseudomonas marginalis	WP_064054232	-R-----A-N-P----D-- E QLPEQ--------------
Pseudomonas floridensis	WP_083180979	-R-----A-N-P------- E NFPTQ--------------
Pseudomonas reinekei	WP_075946174	-R-----A-N-P------F E QLPQP--------------
Pseudomonas proteolytica	WP_092230900	-R-----A-N-P----Q-- E QLPKQ--------------
Pseudomonas denitrificans	WP_003106464	-RQ----A---P------- A KVPAA--------------
Pseudomonas brenneri	WP_032858567	-R-----A-N-P----Q-- E QLPKQ--------------
Pseudomonas lurida	WP_034109682	-R-----A-N-P------- A QLPKQ--------------

Figure-S88

Partial sequence alignments of Pyrroloquinoline quinone biosynthesis protein showing a 1aa Del (highlighted), which is specific for the genus *Azotobacter*.

.

Group	Species	Accession	33		71
Genus *Azotobacter* (4/4)	*Azotobacter vinelandii*	WP_012699152	PKAPEIFRQDWKPSVQLDLNTRQKALD	G	GDFYEVVLSLS
	Azotobacter salinestris	WP_152387562	---------------------K----T	-	--------T--
	Azotobacter chroococcum	WP_039806380	---------------------K--P-N	-	--------T--
	Azotobacter beijerinckii	WP_090620300	---------------------K--P-N	-	--------T--
Other *Pseudomonadacea*	*Azomonas agilis*	WP_144570755	--S-----K----GIS--I----NH--		NH----I-T--
	Azomonas macrocytogenes	WP_183167191	--S--------T-GIS--I----NN-E		--------T--
	Entomomonas moraniae	WP_109704159	----A--LKE---A-N-E-----NTIE		EGV---I-TV-
	Oblitimonas alkaliphila	WP_053100624	--S-----AA-E-A-G-E---S-SNIG		D-------T-T
	Pseudomonas aeruginosa	WP_003096050	--S------E-N--IS--------Q--		--------TV-
	Pseudomonas peli	WP_090254767	--S------E-A---AM----------		---H----T--
	Pseudomonas otitidis	WP_044405414	--S------E-S---A--------N--		--------T--
	Pseudomonas guineae	WP_090244258	--S------E-T---AM----------		---H----T--
	Pseudomonas dryadis	WP_131176700	--S------E-T---AM----------		---H----T--
	Pseudomonas knackmussii	WP_043256063	---------E-Q--ISM-------Q--		--------TV-
	Pseudomonas thermotolerans	WP_017937552	--S------E-A--LS-----------		---H----T--
	Pseudomonas anguilliseptica	WP_090379533	--S------E-V---AM----------		---H----T--
	Pseudomonas marincola	WP_069902421	--S------E-T---EM-------S--		---H----T--
	Pseudomonas azotifigens	WP_028238297	--S--------N---S--------G-E		---H----T--
	Pseudomonas oleovorans	WP_037055281	--S------E-T---G----------E		---H----T--
	Pseudomonas khazarica	WP_037055281	--S------E-T---G----------E		---H----T--
	Pseudomonas hydrolytica	WP_013717617	--S------E-T---G----------E		---H----T--
	Pseudomonas sediminis	WP_099526016	--S------E-T---G----------E		---H----T--
	Pseudomonas alcaliphila	WP_075750514	--S------E-T---G----------E		---H----T--
	Pseudomonas toyotomiensis	WP_017675937	--S------E-T---G----------E		---H----T--
	Pseudomonas nitroreducens	WP_024762250	---------E-Q-AISM-------Q--		--------TV-
	Pseudomonas nitritireducens	WP_024762250	---------E-Q-AISM-------Q--		--------TV-
	Pseudomonas pseudoalcaligenes	WP_003464143	--S------E-T---G----------E		---H----T--
	Pseudomonas indoloxydans	WP_003464143	--S------E-T---G----------E		---H----T--
	Pseudomonas multiresinivorans	WP_169935822	---------E-Q-AISM-------Q--		--------TV-
	Pseudomonas straminea	WP_013792982	--S------E-T---SM---------E		---H----T--
	Pseudomonas fulva	WP_013792982	--S------E-T---SM---------E		---H----T--
	Pseudomonas argentinensis	WP_013792982	--S------E-T---SM---------E		---H----T--
	Pseudomonas resinovorans	WP_016495202	--S------E-S---A--------Q--		---H----T--
	Pseudomonas composti	WP_074939487	--S------E-T---G----------E		---H----T--
	Pseudomonas chengduensis	WP_074939487	--S------E-T---G----------E		---H----T--
	Pseudomonas panipatensis	WP_090265284	---------E-Q-AISM-------Q--		--------TV-
	Pseudomonas delhiensis	WP_089394544	---------E-Q-AISM-------Q--		--------TV-
	Pseudomonas taeanensis	WP_025164139	--S------E-T---AM-------S--		---H----T--
	Pseudomonas leptonychotis	WP_136664146	--S------E-T---AM-------S--		---H----T--
	Pseudomonas kirkiae	WP_131182793	--S------E-Q-N-A-----K--T--		--------T--
	Pseudomonas cuatrocienegasensi	WP_069516341	--S-----KE-T---AM----------		---H----T--
	Pseudomonas songnenensis	WP_095460301	--S------E-N---A-----K----E		---H----T--
	Pseudomonas kunmingensis	WP_014818643	--S------E-N---A-----K----E		---H----T--
	Pseudomonas chloritidismutans	WP_014818643	--S------E-N---A-----K----E		---H----T--
	Pseudomonas punonensis	WP_073262512	--S------E-T---AM---------E		---H----T--
	Pseudomonas humi	WP_009613819	---------E-Q-AISM-------Q--		--------TV-
	Pseudomonas citronellolis	WP_009613819	---------E-Q-AISM-------Q--		--------TV-
	Pseudomonas flavescens	WP_084308298	--S------E-T---AM---------E		---H----T--
	Pseudomonas seleniipraecipitan	WP_070880554	--S------E-T---AM---------E		---H----T--
	Pseudomonas kuykendallii	WP_090225569	--S--------T---A--------P-E		---H----T--
	Pseudomonas sihuiensis	WP_074680186	--S------E-T---GM---------E		---H----T--
	Pseudomonas furukawaii	WP_003453349	--S------Q-N---S--------Q--		---H----T--
	Pseudomonas benzenivorans	WP_090441006	--S------E-T---A--------P--		---H----T--
	Pseudomonas jinjuensis	WP_084311750	-----------Q--IAM-------Q-E		---H----TV-
	Pseudomonas nosocomialis	WP_138406703	--S------E-N---A-----K----E		---H----T--
	Pseudomonas fluvialis	WP_093984564	--S--------A----------H-E-E		AG-H-------
	Pseudomonas pharmafabricae	WP_101193575	--S--------A----------H-E-E		AG-H-------
	Pseudomonas saudiphocaensis	WP_037024217	--S------E-N---G--------S-E		---H----T--
	Pseudomonas borbori	WP_090501203	--S-----KE-T---A--------S--		---H----T--
	Pseudomonas mosselii	WP_011531802	--S-Q----Q-E---S----------E		---H----T--
	Pseudomonas entomophila	WP_011531802	--S-Q----Q-E---S----------E		---H----T--
	Pseudomonas soli	WP_023628821	--S-Q----Q-E---S----------E		---H----T--
	Pseudomonas caeni	WP_022967430	--S-----E--S-A-E-------R--E		---F----T-T
	Pseudomonas mendocina	WP_004373984	--S------E-T---G--------T-E		---H----T--
	Pseudomonas hydrolytica	WP_004373984	--S------E-T---G--------T-E		---H----T--
	Pseudomonas guguanensis	WP_090429566	--S------E-T---G--------T-E		---H----T--
	Pseudomonas denitrificans	WP_003096050	--S------E-N--IS--------Q--		--------TV-
	Pseudomonas linyingensis	WP_090306625	--S--V---E---AIS--------D-G		---H----TV-
	Pseudomonas zhaodongensis	WP_102836573	--S------E-N---A-----K--S-E		---H----T--
	Pseudomonas xanthomarina	WP_073299263	--S------E-N---A-----K--S-E		---H----T--
	Pseudomonas oryzae	WP_090349936	--S------E---GIT--------D-G		---H----TV-
	Pseudomonas canadensis	WP_016979126	--S-A----E-T---A----------E		---H----T--
	Pseudomonas fluorescens	WP_053253894	--S-A----E-T---A----------E		---H----T--
	Pseudomonas simiae	WP_010213875	--S-A----E-T---A----------E		---H----T--
	Pseudomonas reactans	WP_010213875	--S-A----E-T---A----------E		---H----T--
	Pseudomonas lurida	WP_010213875	--S-A----E-T---A----------E		---H----T--
	Pseudomonas extremorientalis	WP_010213875	--S-A----E-T---A----------E		---H----T--
	Pseudomonas azotoformans	WP_010213875	--S-A----E-T---A----------E		---H----T--
	Pseudomonas trivialis	WP_003237490	--S-A----E-T---A----------E		---H----T--
	Pseudomonas poae	WP_003237490	--S-A----E-T---A----------E		---H----T--
	Pseudomonas cedrina	WP_003237490	--S-A----E-T---A----------E		---H----T--
	Pseudomonas tuomuerensis	WP_039562544	--S------E-N----------T-T--		N--H----T--
	Pseudomonas flexibilis	WP_039562544	--S------E-N----------T-T--		N--H----T--
	Pseudomonas kitaguniensis	WP_058413146	--S-A----E-T---A----------E		---H----T--
	Pseudomonas salomonii	WP_012721755	--S-A----E-T---A----------E		---H----T--
	Pseudomonas nabeulensis	WP_012721755	--S-A----E-T---A----------E		---H----T--
	Pseudomonas marginalis	WP_012721755	--S-A----E-T---A----------E		---H----T--
	Pseudomonas edaphica	WP_012721755	--S-A----E-T---A----------E		---H----T--
	Pseudomonas antarctica	WP_064450254	--S-A----E-T---A----------E		---H----T--
	Pseudomonas fildesensis	WP_048723371	--S-A----E-T---A----------E		---H----T--
	Pseudomonas costantinii	WP_071482611	--S-A----E-T---A----------E		---H----T--
	Pseudomonas yamanorum	WP_003218009	--S-A----E-T---A----------E		---H----T--
	Pseudomonas tolaasii	WP_003171119	--S-A----E-T---A----------E		---H----T--
	Pseudomonas sivasensis	WP_014716520	--S-A----E-T---A----------E		---H----T--
	Pseudomonas rhodesiae	WP_014716520	--S-A----E-T---A----------E		---H----T--

Other
Pseudomonadacea

Pseudomonas paralactis	WP_014716520	--S-A----E-T---A----------E	---H----T--
Pseudomonas libanensis	WP_014716520	--S-A----E-T---A----------E	---H----T--
Pseudomonas cremoricolorata	WP_028695698	--S-A----Q-E---A-----K-----	---H----T--
Pseudomonas synxantha	WP_043047290	--S-A----E-T---A----------E	---H----T--
Pseudomonas grimontii	WP_090401027	--S-A----E-T---A----------E	---H----T--
Pseudomonas matsuisoli	WP_188981717	--S------E-A-N-A-----K----E	---H----T--
Pseudomonas orientalis	WP_057724496	--S-A----E-T---A----------E	---H----T--
Pseudomonas kairouanensis	WP_005783933	--S-A----E-T---A----------E	---H----T--
Pseudomonas veronii	WP_017849929	--S-A----E-T---G----------E	---H----T--
Pseudomonas panacis	WP_017849929	--S-A----E-T---G----------E	---H----T--
Pseudomonas haemolytica	WP_034117291	--S-A----E-T---A----------E	---H----T--
Pseudomonas nitrititolerans	WP_014854470	--S-----HE-N---A-----K----E	---H----T--
Pseudomonas alcaligenes	WP_061902541	--S------E-A---G-----K--Q--	---H----T--
Pseudomonas sichuanensis	WP_110995318	--S-Q----Q-E-N-S----------E	---H----T--
Pseudomonas helmanticensis	WP_007909233	--S-A----Q-D-A-A----------E	--------T--
Pseudomonas daroniae	WP_131179628	--S------E-T---AM-------P-E	---H----T--
Pseudomonas taiwanensis	WP_027907926	--S-Q----Q-E---A-----K----E	---H----T--
Pseudomonas guangdongensis	WP_090212045	--T--V---E-Q-AISV-------E-G	---H----T--
Pseudomonas umsongensis	WP_008058540	--S-A----Q-E---G----------E	---H----T--
Pseudomonas kribbensis	WP_085712776	--S-A----Q-D-T-G----------E	--------T--
Pseudomonas lini	WP_007897410	--S-A----Q-E---G----------E	---H----T--
Pseudomonas cichorii	WP_025262429	--S-A----E-T---S--------P-E	--------T--
Pseudomonas gingeri	WP_017126979	--S-A----Q-E---G----------E	---H----T--
Pseudomonas prosekii	WP_092274668	--S-A----Q-E---G----------E	---H----T--
Pseudomonas mandelii	WP_007949026	--S-A----Q-E---G----------E	---H----T--
Pseudomonas arsenicoxydans	WP_007949026	--S-A----Q-E---G----------E	---H----T--
Pseudomonas moraviensis	WP_016772114	--S-A----Q-D-A-G----------E	--------T--
Pseudomonas koreensis	WP_016772114	--S-A----Q-D-A-G----------E	--------T--
Pseudomonas atacamensis	WP_016772114	--S-A----Q-D-A-G----------E	--------T--
Pseudomonas migulae	WP_017336062	--S-A----Q-E---G----------E	---H----T--
Pseudomonas capeferrum	WP_033701969	--S-Q----Q-E---A-----K----E	---H----T--
Pseudomonas agarici	WP_017132488	--S-A----Q-E---G----------E	---H----T--
Pseudomonas juntendi	WP_009685000	--S-Q----T-E---A-----K----E	---H----T--
Pseudomonas shirazica	WP_013974581	--S-Q----T-E---A-----K----E	---H----T--
Pseudomonas putida	WP_016489596	--S-Q----T-E---A-----K----E	---H----T--
Pseudomonas pudica	WP_013974581	--S-Q----T-E---A-----K----E	---H----T--
Pseudomonas plecoglossicida	WP_013974581	--S-Q----T-E---A-----K----E	---H----T--
Pseudomonas monteilii	WP_013974581	--S-Q----T-E---A-----K----E	---H----T--
Pseudomonas massiliensis	WP_040260143	--S-A----Q-E---S----------	S--H----T--
Pseudomonas inefficax	WP_013974581	--S-Q----T-E---A-----K----E	---H----T--
Pseudomonas asiatica	WP_013974581	--S-Q----T-E---A-----K----E	---H----T--
Pseudomonas palleroniana	WP_090367361	--S-A----E-T---A--------S-E	---H----T--
Pseudomonas segetis	WP_010488600	--S-----KE-T-A-EM-------P--	---H-I--T--
Pseudomonas pohangensis	WP_090192591	--S-----EE-R---E--I----RT-E	---H----K--
Pseudomonas carnis	WP_032894017	--S-A----E-T---A--------S-E	---H----T--
Pseudomonas stutzeri	WP_011915080	--S------E-N---A-----K--Q-E	S-------T--
Pseudomonas parafulva	WP_028631981	--S-Q----T-E---G-----K----E	---H----T--
Pseudomonas lactis	WP_003187799	--S-A----E-T---A--------S-E	---H----T--
Pseudomonas coleopterorum	WP_049861717	--S-A----Q-E---S--------G-E	---H----T--
Pseudomonas syringae	WP_024646364	--S-A----E-T-T-S--------Q-E	--------T--
Pseudomonas caspiana	WP_027898930	--S-A----E-T-T-S--------P-E	--------T--
Pseudomonas floridensis	WP_024685362	--S-A----E-T-T-S--------P-E	--------T--
Pseudomonas asturiensis	WP_024685362	--S-A----E-T-T-S--------P-E	--------T--
Pseudomonas savastanoi	WP_002551468	--S-A----E-T-T-S--------Q-E	--------T--
Pseudomonas meliae	WP_002551468	--S-A----E-T-T-S--------Q-E	--------T--
Pseudomonas indica	WP_084335856	--S------E-S---S--------H-E	A--H----T--
Pseudomonas ficuserectae	WP_002551468	--S-A----E-T-T-S--------Q-E	--------T--
Pseudomonas caricapapayae	WP_002551468	--S-A----E-T-T-S--------Q-E	--------T--
Pseudomonas amygdali	WP_002551468	--S-A----E-T-T-S--------Q-E	--------T--
Pseudomonas avellanae	WP_005621582	--S-A----E-T-T-S--------P-E	--------T--
Pseudomonas tremae	WP_005896108	--S-A----E-T-T-S--------Q-E	--------T--
Pseudomonas coronafaciens	WP_005896108	--S-A----E-T-T-S--------Q-E	--------T--
Pseudomonas cannabina	WP_005896108	--S-A----E-T-T-S--------Q-E	--------T--
Pseudomonas viridiflava	WP_004883102	--S-A----E-T-T-S--------P-E	--------T--
Pseudomonas rhizosphaerae	WP_043191461	--S-A----Q-E---S--------G-E	---H----T--
Pseudomonas guariconensis	WP_090343305	--S-Q----Q-E---S----------E	S--H----T--
Pseudomonas congelans	WP_003372189	--S-A----E-T-T-S--------Q-E	--------T--
Pseudomonas cerasi	WP_003372189	--S-A----E-T-T-S--------Q-E	--------T--
Pseudomonas granadensis	WP_064117041	--S-A----Q-D-A-A----------E	---F----T--
Pseudomonas lundensis	WP_047272909	--S-A----Q-E-T-A--------P-E	--------T--
Pseudomonas sagittaria	WP_092433235	--S--V---E---AIS--------D-G	N--H----TV-
Pseudomonas extremaustralis	WP_010563140	--S-A----E-T---A--------P-E	---H----T--
Pseudomonas asuensis	WP_188867450	--S-D------Q-N-N---S------N	E-------T-T
Pseudomonas hunanensis	WP_003249206	--S-Q----T-E---A-----K--S-E	---H----T--
Pseudomonas cremoris	WP_185704729	--S-A----E-T---A--------P-E	---H----T--
Pseudomonas zeshuii	WP_010796135	--S-D------Q-N-N---S------N	E-------T-T
Pseudomonas luteola	WP_010796135	--S-D------Q-N-N---S------N	E-------T-T
Pseudomonas vancouverensis	WP_093227599	--S-A----Q-E---A----------E	A--H----T--
Pseudomonas helleri	WP_048366783	--S-A----Q-E---A--------P-E	---F----T--
Pseudomonas saponiphila	WP_092318769	--S-A----Q-E---A----------E	A--H----T--
Pseudomonas mucidolens	WP_084381845	--S-A----E-T---G--------Q-E	---H----T--
Pseudomonas brenneri	WP_065944518	--S-A----E-T---G--------Q-E	---H----T--
Pseudomonas proteolytica	WP_029295909	--S-A----E-T---G--------Q-E	---H----T--
Pseudomonas gessardii	WP_029295909	--S-A----E-T---G--------Q-E	---H----T--
Pseudomonas laurylsulfatovoran	WP_015093084	--S-A----Q-E---A----------E	S--H----T--
Pseudomonas ovata	WP_056842166	--S-A----E-T---S--------E-E	N-------T--
Pseudomonas piscis	WP_022639251	--S-A----Q-E---S----------E	T--H----T--
Pseudomonas aestus	WP_022639251	--S-A----Q-E---S----------E	T--H----T--
Pseudomonas reidholzensis	WP_119146715	--S-QV---Q-E-Q-A-----K----E	---H----T--
Pseudomonas rhizoryzae	WP_058761466	--S--------Q-N-N---A----T-G	D--H----T-T
Pseudomonas psychrotolerans	WP_058761466	--S--------Q-N-N---A----T-G	D--H----T-T
Pseudomonas psychrotolerans	WP_058761466	--S--------Q-N-N---A----T-G	D--H----T-T
Pseudomonas laurentiana	WP_163938651	--S-A----Q-E---A-----K--G-E	---H----T--
Pseudomonas moorei	WP_090328053	--S-A----Q-E---G----------E	N--H----T--
Pseudomonas brassicae	WP_163941054	--S-A----Q-E---A-----K--G-E	---H----T--
Pseudomonas silesiensis	WP_064675449	--S-A----Q-E---G----------E	N--H----T--
Pseudomonas mohnii	WP_047536344	--S-A----Q-E---G----------E	N--H----T--

Other *Pseudomonadacea*	*Pseudomonas qingdaonensis*	WP_043862197	--S-A----Q-E---A-----K--G-E	---H----T--
	Pseudomonas frederiksbergensis	WP_071552365	--S-A----Q-E---G----------E	S--H----T--
	Pseudomonas alkylphenolica	WP_038615493	--S-A----Q-E---A-----K--G-E	---H----T--
	Pseudomonas versuta	WP_060696097	--S-A----Q-E---G--------P-E	---H----T--
	Pseudomonas reinekei	WP_056852207	--S-A----Q-E---G----------E	S--H----T--
	Pseudomonas japonica	WP_042120535	--S-A----Q-E---A-----K--G-E	---H----T--
	Pseudomonas deceptionensis	WP_046810335	--S-A----Q-E---G--------P-E	---H----T--
	Pseudomonas psychrophila	WP_019824834	--S-A----Q-E---G--------P-E	---H----T--
	Pseudomonas fragi	WP_003438592	--S-A----Q-E---G--------P-E	---H----T--
	Pseudomonas bubulae	WP_003438592	--S-A----Q-E---G--------P-E	---H----T--
	Pseudomonas balearica	WP_043217925	--S------E-N---A-----K--P-E	S--F----T--
	Pseudomonas duriflava	WP_145139169	--S-D------Q-N-N---S------T	D-------T-T
	Pseudomonas batumici	WP_040063502	--S-A----Q-E---G----------E	S--H----T--
	Pseudomonas vranovensis	WP_028943971	--S-A----Q-E---A-----K--G-E	---H----T--
	Pseudomonas donghuensis	WP_010222288	--S-A----Q-E---A-----K--G-E	---H----T--
	Pseudomonas wadenswilerensis	WP_038997589	--S-A----Q-E---A-----K--G-E	---H----T--
	Pseudomonas baetica	WP_095190086	--S-A----Q-D---G--------E-E	---F----T--
	Pseudomonas chlororaphis	WP_009046533	--S-A----Q-E---G----------E	EG-H----T--
	Pseudomonas weihenstephanensis	WP_048364616	--S-A----Q-E-T-A--------P-E	---H----T--
	Pseudomonas mediterranea	WP_047699815	--S-A----Q-E---G----------E	E--H----T--
	Pseudomonas kilonensis	WP_018606305	--S-A----Q-E---G----------E	D--H----T--
	Pseudomonas brassicacearum	WP_024618249	--S-A----Q-E---G----------E	D--H----T--
	Pseudomonas corrugata	WP_024778064	--S-A----Q-E---G----------E	E--H----T--
	Pseudomonas thivervalensis	WP_053117531	--S-A----Q-E---G----------E	D--H----T--
	Pseudomonas jessenii	WP_057397192	--S-A----Q-E---A--------T-E	A--H----T--
	Pseudomonas fuscovaginae	WP_010453507	--S-A----Q-E---G----------E	D--H----T--
	Pseudomonas asplenii	WP_010453507	--S-A----Q-E---G----------E	D--H----T--
	Pseudomonas abietaniphila	WP_074758056	--S-A----E-T---S--------Q-E	N-------T--
	Pseudomonas laurylsulfatiphila	WP_104451152	--S-A----Q-E---A--------V-E	S--H----T--
	Pseudomonas oryzihabitans	WP_059313278	--S------E-Q-N-N---A----T-G	D--H----T-T
	Pseudomonas bohemica	WP_110950471	--S-A----E-T---S--------E-E	N-------T--
	Pseudomonas lutea	WP_037016263	--S-A----E-T---S--------Q-E	N-------T--
	Pseudomonas graminis	WP_074889799	--S-A----E-T---S--------Q-E	N-------T--
	Pseudomonas protegens	WP_011058749	--S-A----Q-E---G-------T--E	A--H----T--
	Pseudomonas endophytica	WP_055101216	--S-A----Q-E---A--------P-E	D--F----T--
	Pseudomonas taetrolens	WP_048383973	--S-A----Q-E---G--------P-E	D--H----T--
	Pseudomonas saxonica	WP_122785265	--S-A----A-E---A--------P-E	D--H----T--
	Pseudomonas hussainii	WP_071870972	----Q----E---Q-G-----KHTQ-E	---H----T--
	Pseudomonas typographi	WP_190425053	--S-A----A-E---S-E------P-E	N--H----T--
	Pseudomonas salegens	WP_092383410	-----V-QTQ-N-Q-N------HNK-Q	EGI--------
	Pseudomonas saudimassiliensis	WP_044498979	----AV-QSQ-N-Q-N------HNQ-Q	DGI--------
	Pseudomonas pertucinogena	WP_188636715	----AV-QAQ-N-Q-N------HNK-Q	D-I--------
	Pseudomonas bauzanensis	WP_074779444	----AV-QSQ-N-Q-N------HTE-Q	DGIH-------
	Pseudomonas litoralis	WP_090274592	----AV-QSQ-N-K-N------HTE-Q	EGI--------
Other Bacteria	*Xenorhabdus khoisanae*	WP_047964861	----Q--Q---Q-E-K---D-ASSE-A	QGV-----RVT

Figure-S89

Partial sequence alignments of the Protein-export chaperone SecB showing a 1aa Ins (highlighted), which is specific for the genus *Azotobacter*.

			25		70
Genus *Azotobacter* (4/4)	*Azotobacter vinelandii*	WP_012701585	FDIALLVAILTSLVVVMLDSL		QGFARYGDLFHALEWGLTALFASEY
	Azotobacter beijerinckii	WP_090622957	------L--FA---------V		P-YD---E-LY-----F-M---A--
	Azotobacter salinestris	WP_152386362	---------FA------F---		-SYDH-SG--Y-I--------TA--
	Azotobacter chroococcum	WP_089167728	------L--FA---------V		-SYDH--E--Y----SF-M---A--
Other *Pseudomonadaceae*	*Pseudomonas oleovorans*	WP_150605679	--NW----------------I	E	RFHNQ-AG--S----FF-----V--
	Pseudomonas marincola	WP_090515254	--NI---T--A---------I	Q	GVHSQ-KS-MSG---MF-----A--
	Pseudomonas khazarica	WP_037050516	--NW------A---------I	E	RFHNQ-AG--S----FF-----V--
	Pseudomonas stutzeri	WP_011913004	--TW---I-FA---------I	A	SFNE-H-Q-LTN----F--I--V--
	Pseudomonas toyotomiensis	WP_074916067	--NW---S--A---------I	E	HFHNQ-A-V-G----FF-----I--
	Pseudomonas sediminis	WP_099523100	--NW------A---------I	E	RFHSQ-AGI-S----FF-----I--
	Pseudomonas composti	WP_074937386	--NW------A---------I	E	RFHSQ-AGV-S----FF-----I--
	Pseudomonas endophytica	WP_055104346	--TT--LI-M------V---I	N	SIHI---N-LA-I---F-FI--V--
	Pseudomonas sihuiensis	WP_017679193	--NW---T--A---------I	E	HFHNQ-A-V-G----FF-----I--
	Pseudomonas chloritidismutans	WP_031298120	--TW---I--A------V--I	A	SFNE-H-E-LTT----F-TI--L--
	Pseudomonas guguanensis	WP_090428090	--NW---T--A---------V	E	HFHS--AEV-K----FF-V---I--
	Pseudomonas kunmingensis	WP_090519715	--TW---I--A------F--I	A	SFNE-H-E-LTT----F-TI--L--
	Pseudomonas chengduensis	WP_055986346	--NW---T--A--I------I	E	HFHNQ-A-V-G----FF-----I--
	Pseudomonas songnenensis	WP_106155120	--TW---I--A------F--I	A	AFNE-H-E-LTN---AF--I--V--
	Pseudomonas taeanensis	WP_025165633	--RY--L---A---------V	N	DVHSQ-DQW-TS----F-----A--
	Pseudomonas indica	WP_084339026	--TW--GI-FA------F--V	E	RHHRN-----M----CF-----V--
	Pseudomonas matsuisoli	WP_188981305	---S---V--A--L------I	D	TVH-A---WLAG---VF-T---I--
	Pseudomonas benzenivorans	WP_090448714	--RW--LT--A----------	S	DVHD---E-LLN----F-----I--
	Pseudomonas rhizoryzae	WP_058773617	--TL--AV-MA--T------I	Q	AIHL---SWLYG---IF-----L--
	Pseudomonas mendocina	WP_012018993	--NW---T--A---------V	E	HFHNQ-AEV-R----FF-V---I--
	Pseudomonas hydrolytica	WP_012018993	--NW---T--A---------V	E	HFHNQ-AEV-R----FF-V---I--
	Pseudomonas guangdongensis	WP_090214057	--EL--L--GA--L------I	A	AVNRL--VPLTV----F-----A--
	Pseudomonas atacamensis	WP_122611606	--GI---I--A---I-----I	D	SIHRN-A-VLAYI---F--I-LG--
	Pseudomonas kuykendallii	WP_090230376	--SW--GI--A------V--I	Q	PLHQ-F-T-LL-----F-LI--V--
	Pseudomonas borbori	WP_090499177	--RW--LT--A----------	S	DVHD---Q-LLN---AF-----I--
	Pseudomonas anguilliseptica	WP_090386589	--RY--LT-MA---------V	D	SVHSQ-AG-LASI---F-----I--
	Pseudomonas alcaliphila	WP_075748361	--NW---T--A---------I	E	HFHNQHAVV-G----FF-----I--
	Pseudomonas guineae	WP_090242081	--RY------A------V--I	D	SIHTQ--ETLTVF--AF-----M--
	Pseudomonas peli	WP_090253544	--RY--LT-MA---------I	D	SVHSQ-AP-LAGI---F-----I--
	Pseudomonas oryzihabitans	WP_059315664	--TL--AV-MA--G------I	Q	SIHL---SWLYG---IF-G---I--
	Pseudomonas parafulva	WP_028632127	--KT--LI--A---T-I---I	D	EVHRG--G-LA-----F--V-LC--
	Pseudomonas deceptionensis	WP_048360803	--T---LI-MC-----V---I	N	SIHRD-A--LAYI---F-LV--I--
	Pseudomonas amygdali	WP_044318029	--KIV-----L--L-TVI--I	E	SFHRD-AM--AWI---F-L---I--
	Pseudomonas nosocomialis	WP_138408582	--TW--IV-MA------F--V	A	VYRE-H-A-LTG----F--I--I--
	Pseudomonas cerasi	WP_003370300	--K-V-----L--L-TII--I	E	SIHRD-AM--AWI---F-LM--I--
	Pseudomonas congelans	WP_010436157	--K-V-----L--L-TII--I	E	SIHRD-AM--AWI---F-LM--I--
	Pseudomonas segetis	WP_089360629	--NI---T-VA--I------I	Q	AIHSQ-RAWMSG---MF-----V--
	Pseudomonas pharmafabricae	WP_101192468	--TW-I-L--A--L--L---I	E	VIQQ---LW-NL----F-LF--V--
	Pseudomonas fluvialis	WP_093985698	--TW-I-L--A--L--L---I	E	VIQQ---LW-NL----F-LF--V--
	Pseudomonas helleri	WP_048371321	--TT--LI-MA-----I---I	N	SIHLQ----LAYI---F-FV--I--
	Pseudomonas vancouverensis	WP_093224974	--ST--LI--A-----I---I	D	DVHRN-A--LAYI---F-VI-LG--
	Pseudomonas oryzae	WP_090347355	--KF---T--A--L------V	A	SVSQAH-R-LSL---SF-S---L--
	Pseudomonas versuta	WP_060693787	--TT--LI--C-----V---I	D	SIHRD-A--LAYI---F-FV--V--
	Pseudomonas tremae	WP_005890319	--KIV-----L--L-TVI--I	E	SIHRD-AT--AWI---F-L---I--
	Pseudomonas coronafaciens	WP_005890319	--KIV-----L--L-TVI--I	E	SIHRD-AT--AWI---F-L---I--
	Pseudomonas fragi	WP_016781516	--T---LI--C-----V---I	N	SIHRD-AK-LAYI---F-FV--V--
	Pseudomonas cuatrocienegasensi	WP_069516765	--NL--LT--A---------V	E	SV-----E-LT-V--AF-L---V--
	Pseudomonas bubulae	WP_095002068	--T---LI--C-----V---I	N	SIHRD-AK-LAYI---F-FV--V--
	Pseudomonas duriflava	WP_145145937	--E---F---A---------V	E	MFREQ-RQELSI---TF-I--GI--
	Pseudomonas zeshuii	WP_010794815	--E---FT--A---------V	A	EIH-K-DEILSLC--AF-V--TI--
	Pseudomonas luteola	WP_010794815	--E---FT--A---------V	A	EIH-K-DEILSLC--AF-V--TI--
	Pseudomonas azotifigens	WP_051231946	--TW--LI-FA---------V	A	NVNEQ--S-LTG---AF--I--V--
	Pseudomonas avellanae	WP_005619395	--KIV-----L--L-TVI--I	E	SIHRD-AT--AWI---F-L---I--
	Pseudomonas syringae	WP_024647421	--KIV-----L--L-TVI--I	E	SIHRD-AA--AWI---F-L---I--
	Pseudomonas geniculata	WP_057502928	--LI-V---VA-IL---I--V	Q	HLH-EWSTGLYI----F-II-TA--
	Pseudomonas cannabina	WP_007251176	--KIV-----L--L-TII--I	E	SIHRD-AM--AWI---F-L---I--
	Pseudomonas meliae	WP_005780523	--KIV-----L--L-TVI--I	E	SIHRD-AM--AWI---F-L---I--
	Pseudomonas savastanoi	WP_002554214	--KIV-----L--L-TVI--I	E	SIHRD-AM--AWI---F-L---I--
	Pseudomonas ficuserectae	WP_002554214	--KIV-----L--L-TVI--I	E	SIHRD-AM--AWI---F-L---I--
	Pseudomonas psychrophila	WP_019828880	--T---LI-MC-----V---I	N	SIHRD-AN-LAYI---F-FV--I--
	Pseudomonas lundensis	WP_047282776	--TT--LI--C-----I---I	D	SIHRD-AN-LAYI---F-FV--V--
	Pseudomonas caricapapayae	WP_055007996	--KIV-----L--L-TVI--I	E	SIHRD-AM--AWI---F-L---I--
	Pseudomonas zhaodongensis	WP_128121139	--TW---V-FA---------V	A	LYSD---G-LDS---LF-GV-LI--
	Pseudomonas costantinii	WP_071487693	--ST--LI--A---I-I---I	Q	EVHDN-A--LAYI---F-II--I--
	Pseudomonas asuensis	WP_188868137	--E---FT--A---------V	A	EIH-K-DEILSLC--AF-F--TI--
	Pseudomonas palleroniana	WP_090365976	--ST--LI--A---I-I---I	Q	AVHDN-A-VLAYI---F-II--I--
	Pseudomonas pohangensis	WP_090193741	--EC--IV-IA---------I	Q	EIHDD-KL--YY---LF-GI--I--
	Pseudomonas brenneri	WP_032859137	--GI--LT--A---I-----I	D	-IHQN-A-VLAYI---F-II--I--

Figure-S90

Partial sequence alignments of the Protein Ion transporter showing a 1aa Del (highlighted), which is specific for the genus *Azotobacter*.

			268		309
Genus *Azotobacter* (4/4)	*Azotobacter vinelandii*	WP_012701826	GILCGISSGAAMAAAVRLAE	H	EPALQGKTLVVILPDSGERYL
	Azotobacter salinestris	WP_152386550	-------G------------	-	---M-----------------
	Azotobacter beijerinckii	WP_090623147	--------------------	T	--S------------------
	Azotobacter chroococcum	WP_089168518	-------C------------	-	--S-----I------------
Other *Pseudomonadacea*	*Pseudomonas citronellolis*	WP_074977209	-V----------V-------		---M----I-------A----
	Pseudomonas knackmussii	WP_043253797	-------------V------		---M----I-------A----
	Thiopseudomonas denitrificans	WP_101497684	---S--------V------Q		Q--M----I--V---------
	Pseudomonas mendocina	WP_003244618	-------C----V-------		K-EM----I------------
	Pseudomonas nosocomialis	WP_138408710	-------------V------		H-EMK--NI------------
	Pseudomonas khazarica	WP_037053461	-------C----V------Q		K-DM----I------------
	Pseudomonas resinovorans	WP_016491795	-----------L-V-A--S-		R-EM----I------------
	Pseudomonas seleniipraecipitan	WP_070882900	-------C-----V------		K-EM----I------------
	Pseudomonas veronii	WP_017848760	-------C-----V------		K-EM----I------------
	Pseudomonas panacis	WP_017848760	-------C-----V------		K-EM----I------------
	Pseudomonas silesiensis	WP_064679207	-------------V------		T-EM----I------------
	Pseudomonas gessardii	WP_076962447	-------C-----V------		K-DM----I------------
	Pseudomonas taeanensis	WP_025167269	-------C-----V------		K-EM----I------------
	Pseudomonas agarici	WP_017133443	-------------V------		T-EM-------V---------
	Pseudomonas jessenii	WP_090453716	-------------V------		T-EM----I------------
	Pseudomonas furukawaii	WP_003454865	-------C----V-------		R-EM----F------------
	Pseudomonas daroniae	WP_131192059	-------C-----V------		K-EM----I------------
	Pseudomonas argentinensis	WP_074884627	-------C-----V------		Q-EM----I------------
	Pseudomonas alcaliphila	WP_021490024	-------C-----V------		K-EM----M------------
	Pseudomonas indoloxydans	WP_084340656	-------C----V-------		K-EM----M------------
	Pseudomonas chengduensis	WP_017677426	-------C-----V------		K-EM----I------------
	Pseudomonas chlororaphis	WP_009047584	-------------V------		T-EM----I--V---------
	Pseudomonas sihuiensis	WP_092374648	-------C-----V------		K-EM----M------------
	Pseudomonas sediminis	WP_099525312	-------C----V-------		K-EM----I------------
	Pseudomonas composti	WP_074941699	-------C-----V------		K-EM----I------------
	Pseudomonas toyotomiensis	WP_074914351	-------C-----V------		K-EM----I------------
	Pseudomonas hydrolytica	WP_129483051	-------C----V-------		K-EML---I------------
	Pseudomonas allii	WP_058425079	-------C-----V------		K-EM-----------------
	Pseudomonas flavescens	WP_084304146	-------C-----V------		K-EM----I------------
	Pseudomonas kilonensis	WP_018607561	-------C-----V------		K-EM----I------------
	Pseudomonas bohemica	WP_110948960	-------C----Q-------		K-GM----I------------
	Pseudomonas haemolytica	WP_034118677	-------C-----V------		K-EM----I------------
	Pseudomonas costantinii	WP_071483640	-------C-----V------		K-EM----I------------
	Pseudomonas qingdaonensis	WP_043864186	-------C-----V------		K-EM----I------------
	Pseudomonas brassicacearum	WP_025212408	-------C-----V------		K-EM----I------------
	Pseudomonas mediterranea	WP_047701731	-------C-----V------		K-EM----I------------
	Pseudomonas oleovorans	WP_150609166	-------C----V--G---Q		K-DM----I------------
	Pseudomonas proteolytica	WP_092234375	-------C-----V------		K-EM----I------------
	Pseudomonas azotoformans	WP_061435728	-------C-----V------		K-EM----I------------
	Pseudomonas thivervalensis	WP_053120124	-------C-----V------		K-EM----I------------
	Pseudomonas edaphica	WP_177032583	-------C-----V------		K-EM----I------------
	Pseudomonas cedrina	WP_076950271	-------C-----V------		K-EM----I------------
	Pseudomonas synxantha	WP_057022080	-------C-----V------		K-EM----I------------
	Pseudomonas lurida	WP_034102531	-------C-----V------		K-EM----I------------
	Pseudomonas simiae	WP_044286832	-------C-----V------		K-EM----I------------
	Pseudomonas palleroniana	WP_090366173	-------C-----V------		K-EM----I------------
	Pseudomonas canadensis	WP_010212098	-------C-----V------		K-EM----I------------
	Pseudomonas extremorientalis	WP_071488996	-------C-----V------		K-EM----I------------
	Pseudomonas grimontii	WP_027604255	-------C-----V------		K-EM----I------------
	Pseudomonas trivialis	WP_049709444	-------C-----V------		K-EM----I------------
	Pseudomonas salomonii	WP_056857297	-------C-----V------		K-EM----I------------
	Pseudomonas libanensis	WP_057013320	-------C-----V------		K-EM----I------------
	Pseudomonas cremoris	WP_185709391	-------C-----V------		K-EM----I------------
	Pseudomonas tolaasii	WP_016971401	-------C-----V------		K-EM----I------------
	Pseudomonas marginalis	WP_064052291	-------C-----V------		K-EM----I------------
	Pseudomonas kitaguniensis	WP_152748099	-------C-----V------		K-EM----I------------
	Pseudomonas segetis	WP_089358872	-------C-----V-M-I-N		--NM----I------------
	Pseudomonas koreensis	WP_041478837	-------C-----V------		K-EM----I--V---------
	Pseudomonas atacamensis	WP_016771214	-------C-----V------		K-EM----I--V---------
	Pseudomonas helmanticensis	WP_134177681	-------C-----V------		T-EM----I------------
	Pseudomonas reinekei	WP_075947559	-------C-----V------		T-EM----I------------
	Pseudomonas prosekii	WP_092271673	-------C-----V------		T-EM----I------------
	Pseudomonas baetica	WP_100847545	-------C-----V------		T-EM----I------------
	Pseudomonas reactans	WP_177003453	-------C-----V------		K-EM----I--V---------
	Pseudomonas migulae	WP_084323271	-------C-----V------		T-EM----I------------
	Pseudomonas mandelii	WP_010462101	-------C-----V------		T-EM----I------------
	Pseudomonas benzenivorans	WP_090448237	-------C-----V---I-A		--EM----V------------
	Pseudomonas vancouverensis	WP_093225481	-------C-----V------		T-EM----I------------
	Pseudomonas moorei	WP_090317558	-------C-----V------		T-EM----I------------
	Pseudomonas mohnii	WP_047526884	-------C-----V------		T-EM----I------------
	Pseudomonas umsongensis	WP_033043476	-------C-----V------		T-EM----I------------
	Pseudomonas laurylsulfatiphila	WP_104450493	-------C-----V------		T-EM----I------------
	Pseudomonas brenneri	WP_032863490	-------C-----V------		T-EM----I------------
	Pseudomonas saponiphila	WP_092320449	-------C-----V------		T-EM----I------------
	Pseudomonas piscis	WP_022642925	-------C-----V------		T-EM----I------------
	Pseudomonas aestus	WP_022642925	-------C-----V------		T-EM----I------------
	Pseudomonas protegens	WP_011059857	-------C-----V------		T-EM----I------------
	Pseudomonas moraviensis	WP_064362519	-------C-----V------		T-EM----I--V---------
	Pseudomonas paralactis	WP_057704623	-------C-----V------		K-EM----I------------
	Pseudomonas granadensis	WP_090282246	-------C-----V------		T-EM----I--V---------
	Pseudomonas kuykendallii	WP_090229032	-------C------------		H-EM----I------------
	Pseudomonas yamanorum	WP_063032516	-------C-----V------		K-EM----I--V---------
	Pseudomonas nabeulensis	WP_135306790	-------C-----V-----Q		K-EM----I------------
	Pseudomonas fuscovaginae	WP_019363745	-------C-----V------		T-EM----I--V---------
	Pseudomonas asplenii	WP_090206151	-------C-----V------		T-EM----I--V---------
	Pseudomonas kribbensis	WP_114881819	-------C-----V------		T-EM----I--V---------
	Pseudomonas kairouanensis	WP_105696738	-------C-----V-----Q		K-EM----I------------
	Pseudomonas psychrophila	WP_019828721	-------------V------		T-EM----I------------
	Pseudomonas gingeri	WP_017124539	-------C-----V------		T-EM----I--V---------
	Pseudomonas graminis	WP_074890628	-------C----H-------		K-EM----I--V---------
	Azomonas macrocytogenes	WP_183167707	-------C------------		H-DM----I------------
	Pseudomonas arsenicoxydans	WP_090181341	-------C-----V------		T-EM----I------------

Other *Pseudomonadacea*

Pseudomonas rhodesiae	WP_034096460	-------C-----V-----Q	K-EM----I--V---------
Pseudomonas denitrificans	WP_003090573	--------------------	--NM----I------------
Pseudomonas aeruginosa	WP_003090573	--------------------	--NM----I------------
Pseudomonas cichorii	WP_025261173	-------C----------S-	K-EM----I------------
Pseudomonas xanthomarina	WP_073300459	-------C----V-------	K-EM---NI------------
Pseudomonas marincola	WP_090510518	-------C----S--IK--N	--NMM---I------------
Pseudomonas sagittaria	WP_092431055	-------C---------I-N	---M----I------------
Pseudomonas fildesensis	WP_048725776	-------C-----V------	K-EM----I------------
Pseudomonas linyingensis	WP_090307597	-------C---------I-N	---M----I------------
Pseudomonas poae	WP_003234021	-------C-----V------	K-EM----I------------
Pseudomonas oryzae	WP_090348815	-------C---------I-Q	---M----I------------
Pseudomonas sivasensis	WP_122764924	-------C-----V------	K-EM----I------------
Pseudomonas extremaustralis	WP_010563733	-------C-----V------	K-EM----I------------
Pseudomonas antarctica	WP_064451371	-------C-----V------	K-EM----I------------
Pseudomonas orientalis	WP_057723000	-------C-----V------	K-EM----I------------
Pseudomonas indica	WP_084339610	-------C-----------Q	H-EM---NI------------
Pseudomonas multiresinivorans	WP_169940843	----------------K---	--TM----I--V---------
Pseudomonas fluorescens	WP_053255023	-------C-----V------	K-EM----I--V---------
Pseudomonas bubulae	WP_095002681	-------C-----V------	T-EM----I------------
Pseudomonas lutea	WP_037013912	-------C----Q-------	K-EM----I--V---------
Pseudomonas nitroreducens	WP_037009637	----------------K---	--NM----I--V---------
Pseudomonas nitritireducens	WP_037009637	----------------K---	--NM----I--V---------
Pseudomonas caeni	WP_022966296	-------C-----------Q	Q-SM----M------------
Pseudomonas lundensis	WP_047282806	-------C-----V------	K-EM----I------------
Pseudomonas abietaniphila	WP_074752619	-------C----H-------	K-EM----I--V---------
Pseudomonas tuomuerensis	WP_039561977	-V-----G------------	H-DM---NI------------
Pseudomonas flexibilis	WP_039561977	-V-----G------------	H-DM---NI------------
Pseudomonas guangdongensis	WP_090214397	-------C-----------N	--DM----I------------
Pseudomonas carnis	WP_060764442	-------C-----V------	K-EM----I------------
Pseudomonas lactis	WP_003189630	-------C-----V------	K-EM----I------------
Pseudomonas versuta	WP_060694035	-------C-----V------	T-EM----I------------
Pseudomonas lini	WP_048394089	---S---C-----V------	T-EM----I------------
Pseudomonas frederiksbergensis	WP_071551386	---S---C-----V------	T-EM----I------------
Pseudomonas zeshuii	WP_010794769	-------C-----------R	---M----I------------
Pseudomonas luteola	WP_074824086	-------C-----------R	---M----I------------
Pseudomonas asuensis	WP_188867593	-------C-----------R	---M----I------------
Pseudomonas yangmingensis	WP_093475381	-------C---------I-H	--EM-------V---------
Pseudomonas mucidolens	WP_084376856	---S---C-----V-----Q	K-EM----I--V---------
Pseudomonas batumici	WP_040065138	---S---C-----V-----Q	T-EM----I--V---------
Pseudomonas otitidis	WP_074968705	-------C------------	K-EM-----------------
Pseudomonas taiwanensis	WP_027907426	-------C------------	K--M----I------------
Pseudomonas formosensis	WP_090538561	--------------------	---M-NR-I------------
Pseudomonas punonensis	WP_073268215	-------C------------	Q-DM-----------------
Pseudomonas thermotolerans	WP_017937772	-------G------------	R-EM-----------------
Pseudomonas donghuensis	WP_010223109	--------------------	K-EM----I------------
Pseudomonas straminea	WP_093503402	-------C------------	Q-EM-----------------
Pseudomonas fulva	WP_013791013	-------C------------	Q-DM-----------------
Pseudomonas humi	WP_069861182	-------C------------	---M----I-------A----
Pseudomonas alkylphenolica	WP_038613704	--------------------	K-EM----I--V---------
Pseudomonas hydrolytica	WP_041980923	-------C------------	K-DM----I------------
Pseudomonas wadenswilerensis	WP_115088246	--------------------	K-EM----I------------
Pseudomonas jinjuensis	WP_084309593	---S---C---------I--	--NM----I------------
Pseudomonas typographi	WP_190419156	-------C------------	K-EM-----------------
Pseudomonas massiliensis	WP_040262081	-------C------------	K-EM----I------------
Pseudomonas guariconensis	WP_090345350	-------C------------	K-EM-----------------
Pseudomonas vranovensis	WP_028943143	--------------------	K-EM-----------------
Pseudomonas rhizoryzae	WP_058762836	-------C------------	A-EM----I------------
Pseudomonas psychrotolerans	WP_058762836	-------C------------	A-EM----I------------
Pseudomonas bauzanensis	WP_036989633	-----------------I--	--DM--R-I--V---------
Pseudomonas delhiensis	WP_089393630	-------C------------	--SM----I-------A----
Pseudomonas xinjiangensis	WP_093392142	-------G-----------Q	D--M-N--I--V---------
Pseudomonas japonica	WP_042126597	-------C------------	K-EM----I------------
Pseudomonas panipatensis	WP_090262658	-------C-----------Q	--TM----I-------A----
Pseudomonas alcaligenes	WP_061903848	-------C------------	K-EM----I------------
Pseudomonas cremoricolorata	WP_038412534	-------C------------	K-EM----I------------
Pseudomonas reidholzensis	WP_119141115	-------C------------	K-EM----I------------
Pseudomonas parafulva	WP_028635430	-------C------------	K-EM-----------------
Pseudomonas guguanensis	WP_090425658	-------C------------	K-EMH---I------------
Pseudomonas hussainii	WP_074869620	-------C--------KV-Q	---M----I------------
Pseudomonas capeferrum	WP_033699727	-------C------------	K-EM----I------------
Pseudomonas zhaodongensis	WP_045428091	-------C------------	K-EM---NI------------
Pseudomonas songnenensis	WP_106158409	-------C------------	R-EM---NI------------
Pseudomonas kunmingensis	WP_014820772	-------C------------	R-EM---NI------------
Pseudomonas chloritidismutans	WP_023445110	-------C------------	R-EM---NI------------
Pseudomonas stutzeri	WP_013983084	-------C------------	R-EM---NI------------
Pseudomonas entomophila	WP_011535084	-------C------------	K-EM----I------------
Pseudomonas saudimassiliensis	WP_044499839	-----------------I--	--QM-NR-I--V---------
Pseudomonas putida	WP_016498392	-------C------------	K-EM----I------------
Pseudomonas shirazica	WP_013973746	-------C------------	K-EM----I------------
Pseudomonas pudica	WP_046616455	-------C------------	K-EM----I------------
Pseudomonas plecoglossicida	WP_013973746	-------C------------	K-EM----I------------
Pseudomonas monteilii	WP_013973746	-------C------------	K-EM----I------------
Pseudomonas inefficax	WP_013973746	-------C------------	K-EM----I------------
Pseudomonas hunanensis	WP_004576043	-------C------------	K-EM----I------------
Pseudomonas asiatica	WP_013973746	-------C------------	K-EM----I------------
Pseudomonas mosselii	WP_062574841	-------C------------	K-EM----I------------
Pseudomonas balearica	WP_043219512	-------G------------	R-EM---NI------------
Pseudomonas fluvialis	WP_093985792	-------C-----------A	---M-----------------
Pseudomonas deceptionensis	WP_048360682	--------------------	T-EM----I------------
Pseudomonas pharmafabricae	WP_101192410	-------C-----------A	---M-----------------
Azomonas agilis	WP_144570437	-------G---V---LK--N	---Q----I--V---------
Pseudomonas ovata	WP_056835162	-------C-----------Q	K-EM-----------------
Pseudomonas juntendi	WP_009686784	-------C------------	K-EM----I--V---------
Pseudomonas pertucinogena	WP_188635388	---------------L-I--	--GM-NR-I------------
Pseudomonas viridiflava	WP_088235765	-------C------------	K-EM----I------------
Pseudomonas avellanae	WP_005619816	-------C------------	K-EM----I------------
Pseudomonas tremae	WP_005892144	-------C------------	K-EM----I------------

Other *Pseudomonadacea*	*Pseudomonas coronafaciens*	WP_005892144	-------C------------	K-EM----I------------
	Pseudomonas syringae	WP_025390387	-------C------------	K-EM----I------------
	Pseudomonas floridensis	WP_083184555	-------C------------	K-EM----I------------
	Pseudomonas amygdali	WP_002552621	-------C------------	K-EM----I------------
	Pseudomonas fragi	WP_016781662	--------------------	T-EM----I---F--------
	Pseudomonas cannabina	WP_055000685	-------C------------	K-EM----I------------
	Pseudomonas savastanoi	WP_011168110	-------C------------	K-EM----I------------
	Pseudomonas caricapapayae	WP_055010039	-------C------------	K-EM----I------------
	Pseudomonas matsuisoli	WP_188981284	-------C-----------G	--DM----I--V---------
	Pseudomonas meliae	WP_044344564	-------C------------	K-EM----I------------
	Pseudomonas soli	WP_094010040	-------C------------	K-EM----I------------
	Pseudomonas kirkiae	WP_131184130	---A---G-------I----	Q-E-----I------------
	Pseudomonas jilinensis	WP_080050791	---S---C------------	K-EF-----------------
	Pseudomonas cerasi	WP_054080789	-------C------------	K-EM----I--V---------
	Pseudomonas duriflava	WP_145141937	-------C---------I-R	--SMA---I------------
	Pseudomonas sichuanensis	WP_110992492	-------C------------	K-EM----I------------
	Pseudomonas congelans	WP_054994545	-------C------------	K-EM----I--V---------
	Pseudomonas asturiensis	WP_073171056	-------C------------	K-EM----I--V---------
	Pseudomonas nitrititolerans	WP_014852558	-------G------------	R-EM--RNI------------
	Pseudomonas saudiphocaensis	WP_037021903	-------G------------	R-EMN-R-I------------
	Pseudomonas pelagia	WP_022964170	-------C---------M--	---M----M------------
	Pseudomonas saxonica	WP_122784086	-------C------------	T-EM----I------------
	Pseudomonas profundi	WP_150301224	-------G-----------Q	L-EM--RMI------------
	Pseudomonas oceani	WP_104739553	---V---C-------C-V--	--GM----I------------
	Oblitimonas alkaliphila	WP_053102585	---------------LQ---	Q-EM--------I--------
	Pseudomonas weihenstephanensis	WP_048366357	-------C------------	T-EM----I------------
	Pseudomonas laurentiana	WP_163932865	-------C------------	K-EM----I------------
	Pseudomonas borbori	WP_090503158	-------C-------L-I-A	---M----I------------
	Pseudomonas azotifigens	WP_028239901	-------G---V--------	Q-DM----I-FV---------
	Pseudomonas dryadis	WP_131176355	-------C-----V---M--	K-EM----I------------
	Pseudomonas sabulinigri	WP_092283853	-------C---------M--	--EM----I------------
	Pseudomonas peli	WP_090253204	-------C-------L-I-A	--GM----I------------
	Pseudomonas cuatrocienegasensi	WP_069518328	-------C-----V---M--	R-EM----M--V---------
	Pseudomonas leptonychotis	WP_136664859	-------C-------L---A	--GM----I------------
	Pseudomonas abyssi	WP_096004750	---V---C---------V--	--SM----I------------
	Pseudomonas corrugata	WP_055135328	-------C-----V-A----	K-EM----I------------
	Pseudomonas gallaeciensis	WP_118129604	---V---C---------V--	--SM----I------------
	Pseudomonas litoralis	WP_090273043	-----------------M-Q	--EM-NQ-I------------
	Pseudomonas aestusnigri	WP_088275124	---V---C---------V--	---M----I--V---------
	Pseudomonas pachastrellae	WP_083727460	---V---C---------V--	--NM----I------------
	Pseudomonas rhizosphaerae	WP_043193136	-------C-------A----	K-EM----I------------

Figure-S91

Partial sequence alignments of the protein Cysteine synthase A showing a 1aa Ins (highlighted), which is specific for the genus *Azotobacter*.

Group	Species	Accession	156		195
Genus *Azotobacter* (4/4)	*Azotobacter vinelandii*	WP_012702209	LPMADLDEQLVATPENL		ASGTGLQGVSGRYGANAMLTVRA
	Azotobacter salinestris	WP_152389091	-----------------		-G-----E---------------
	Azotobacter beijerinckii	WP_090624907	-----------------		------A-A----------A---
	Azotobacter chroococcum	WP_089168763	-----------------		---A--E------------S---
Other *Pseudomonadaceae*	*Pseudomonas aeruginosa*	WP_003102400	--L------I-G-----	TA	-QPDA-RAA-E--A-D-L-A-D-
	Pseudomonas taeanensis	WP_025165876	--L--------------	AA	SEP---RAA-E--A-D-L-V-Q-
	Pseudomonas veronii	WP_017845319	--LG-----V---AP--	ES	-DA-P-RAA-E----D---A-H-
	Pseudomonas panacis	WP_046382481	--LG-----V---AP--	ES	-DA-P-RAA-E----D---A-H-
	Pseudomonas silesiensis	WP_064679017	--L---N--I---AP--	ES	-DPAP-R-A----N-D-L-A-H-
	Pseudomonas cedrina	WP_076952930	--LG-----V---AS--	ES	SDA-P-RAA-E----D-L-A-H-
	Pseudomonas lurida	WP_098467178	--LG-----V---AP--	ES	-DA-P-RAA-E----D-L-A-H-
	Pseudomonas reactans	WP_177004464	--LG-----V---AP--	ES	-DA-P-RAA-E----D-L-A-H-
	Pseudomonas marginalis	WP_064052704	--LG-----V---AP--	ES	-DA-P-RAA-E----D-L-A-H-
	Pseudomonas canadensis	WP_028617372	--LG-----V---AP--	ES	-DA-P-RAA-E----D-L-A-H-
	Pseudomonas allii	WP_179029597	--LG-----V---AP--	ES	-DA-P-RAA-E----D-L-A-H-
	Pseudomonas trivialis	WP_049712104	--LG-----V---AP--	ES	-DA-P-RAA-E----D-L-A-H-
	Pseudomonas kairouanensis	WP_135288646	--LG-----V---AP--	ES	-DA-P-RAA-E----D-L-A-H-
	Pseudomonas edaphica	WP_017134882	--LG-----V---AP--	ES	-DA-P-RAA-E----D-L-A-H-
	Pseudomonas borbori	WP_090497662	--I---N---L------	GA	-EPQA-RTA-E--A-D-L-A-Q-
	Pseudomonas paralactis	WP_057700955	--LG-----V---AP--	ES	-DA-P-RAA-E----D-L-A-H-
	Pseudomonas extremorientalis	WP_071491320	--LG-----V---AP--	ES	-DA-P-RAA-E----D-L-A-H-
	Pseudomonas tolaasii	WP_016972428	--LG-----V---AP--	ES	-DA-P-RAA-E----D-L-A-H-
	Pseudomonas salomonii	WP_069788061	--LG-----V---AP--	ES	-DA-P-RAA-E----D-L-A-H-
	Pseudomonas grimontii	WP_090400119	--LG-----V---AP--	ES	-DA-P-RAA-E----D-L-A-H-
	Pseudomonas sivasensis	WP_181639427	--LG-----V---AP--	ES	-DA-P-RAA-E----D-L-A-H-
	Pseudomonas fildesensis	WP_048720763	--LG-----V---AP--	ES	-DA-P-RAA-E----D-L-A-H-
	Pseudomonas synxantha	WP_057023067	--LG-----V---AP--	ES	-DA-P-RAA-E----D-L-A-H-
	Pseudomonas orientalis	WP_057721292	--LG-----V---AP--	ES	-DA-P-RAA-E----D-L-A-H-
	Pseudomonas mandelii	WP_010456808	--L---S------AP--	EG	-DA-P-RAA-D--N-D-L-A-H-
	Pseudomonas fluorescens	WP_053257359	--LG-----V---AP--	ES	-DA-P-RAA-E----D-L-A-H-
	Pseudomonas azotoformans	WP_061449205	--LG-----V---AP--	ES	-DA-P-RDA-E----D-L-A-H-
	Pseudomonas lactis	WP_003193058	--LG-----V---AP--	ES	PDA-P-RAA-E----D-L-A-H-
	Pseudomonas antarctica	WP_064453507	--LG-----V---AP--	ES	-DA-P-RAA-E----D-L-A-H-
	Pseudomonas carnis	WP_034128325	--LG-----V---AP--	ES	PDA-P-RAA-E----D-L-A-H-
	Pseudomonas reinekei	WP_075948458	--L---N--I---AP--	ES	-DPAA-R-A-E--N-D-L-A-H-
	Pseudomonas haemolytica	WP_153871098	--LG-----V---AP--	ES	-DA-P-RAA-E----D-L-A-H-
	Pseudomonas simiae	WP_045791771	--LG-----V---AP--	ES	-DA-P-RTA-E----D-L-A-H-
	Pseudomonas yamanorum	WP_063032446	--LG-----I---AP--	ES	PDA-P-RAA-E----D-L-A-H-
	Pseudomonas fuscovaginae	WP_019360683	--L---E--I---AK--	EG	SDASP--A--E----D-L-A-H-
	Pseudomonas saponiphila	WP_092311752	--LG-----I---AP--	EG	-DP-P-RQA-E----D-L-A-H-
	Pseudomonas lini	WP_038979704	--L---N--I---AP--	ES	-DPAP-R-A-D--N-D-L-A-H-
	Pseudomonas palleroniana	WP_060756093	--LG-----V---AP--	ES	-DA-P-RAA-E----D-L-A-H-
	Pseudomonas costantinii	WP_071482943	--LG-----V---AP--	ES	-DA-P-RAA-E----D-L-A-H-
	Pseudomonas agarici	WP_017130965	--L------I---AK--	EG	-DASP-H-A-E----D-L-A-H-
	Pseudomonas marincola	WP_090513230	M-L---S----------	AA	SQPDA-EPI-E----DSL-A-T-
	Pseudomonas benzenivorans	WP_090441317	--L---G----------	GA	SEPDA-RSA-E--A-D-L-A-Q-
	Pseudomonas kitaguniensis	WP_152746540	--LG-----V---AP--	ES	-DAAP-RAA-E----D-L-A-H-
	Pseudomonas cremoris	WP_185703574	--LG--E--V---AP--	ES	-DA-P-RAA-E----D-L-A-H-
	Pseudomonas asplenii	WP_090205782	--L---E--I---AK--	EG	SDASP--AA-E----D-L-A-H-
	Pseudomonas rhodesiae	WP_034138889	--LG--E--V---AP--	ES	-DA-P-RAA-E----D-L-A-H-
	Pseudomonas frederiksbergensis	WP_071553893	---G--N--I---APH-	EG	NDPAP-H-I-E----D-L-A-H-
	Pseudomonas libanensis	WP_057011828	--LG-----V-G-AP--	ES	PDA-P-RAA-E----D-L-A-H-
	Pseudomonas arsenicoxydans	WP_090181668	--L---S------AP--	EG	TDA-P-RAA-D--N-D-L-A-H-
	Pseudomonas poae	WP_060548804	--LG--N--V---AP--	ES	-DA-P-RAA-E----D-L-A-H-
	Pseudomonas vancouverensis	WP_093224682	--L---N--I---AQ--	EG	TDPAP-HS--E--N-D-L-A-H-
	Pseudomonas weihenstephanensis	WP_048362861	--L---N----G-V---	EG	TNPAP-REA-E----D-L-A-H-
	Pseudomonas nabeulensis	WP_135307931	--LG--E--V---AP--	ES	-DA-P-RAA-E----D-L-A-H-
	Pseudomonas extremaustralis	WP_010563918	--LG-----V---AP--	ES	-DA-P-RAA-E----D-L-A-H-
	Pseudomonas indica	WP_084334671	--L---S--I-G-AD--	TA	-RPDA-REA-D--A-D-L-A---
	Pseudomonas moorei	WP_090324156	--L---N--V---AP--	ES	SDPAA-RAA-E--S-D-V-A-H-
	Pseudomonas mohnii	WP_090464049	--L---N--V---AP--	ES	SDPAA-RAA-E--N-D-V-A-H-
	Pseudomonas proteolytica	WP_029292401	--LG-----I---AP--	ES	-DPAP-RSA-E----D-L-A-H-
	Pseudomonas brenneri	WP_090290652	--LG-----I---AP--	ES	-DPAP-RSA-E----D-L-A-H-
	Pseudomonas seleniipraecipitan	WP_070881715	--L---S---L--E---	AA	KQPDA-RTA-E----D-L-A---
	Pseudomonas jinjuensis	WP_084309856	--L---G--SLG-----	AA	-QPEA-RAA-E----D-I-S-V-
	Pseudomonas deceptionensis	WP_048358879	--L---S----G-AD--	EG	SNPAP-KDA-E----D-L-A-H-
	Pseudomonas kuykendallii	WP_090228122	--L---P------ADT-	KA	S-PDA-REI-T----D-L-A-D-
	Pseudomonas fragi	WP_016779860	--L---S----G-A---	EG	SNPAP-KDA-E----D-L-A-H-
	Pseudomonas gingeri	WP_017127760	--L------I---AK--	EG	SDSSP-HAA-E----D-L-A-H-
	Pseudomonas migulae	WP_084322343	--L---S------AP--	EG	TDAAP-R-A-D--N-D-L-A-H-
	Pseudomonas lundensis	WP_047276387	--L---S----G-VD-I	EG	-NPAP-REA-E----D-L-A-H-
	Pseudomonas daroniae	WP_131193218	--L---S---L--D---	SA	QDPDS-RSA-E----D-L-A---
	Pseudomonas matsuisoli	WP_188981436	--L---E------TD--	AN	PG-GA-TDA-Q--D-DGI-----
	Pseudomonas otitidis	WP_074971900	--L---S---A------	TA	-DPAA-HPA-E----D-L-A-L-
	Pseudomonas koreensis	WP_041479549	--L---S------APA-	ES	-DPAP-RSA-E--S-D-L---H-
	Pseudomonas flavescens	WP_084306044	--L---G---L--D---	SA	KDPGA-RSA-E----D-L-A---
	Pseudomonas dryadis	WP_131176595	--L-------L--AD--	GA	SDPEA-RSA-E----D-L-A-H-
	Pseudomonas piscis	WP_152898708	--LG--N--I---AP--	EG	-DPAP-REA-E----D-L-A-H-
	Pseudomonas chlororaphis	WP_038634280	--L---N--I---AP--	EG	SDSAP-RAA-E----D-L-A-H-
	Pseudomonas laurylsulfatovoran	WP_103394806	--L---N--I---AP--	EG	-DPAP-HAA-E--N-D-L-A-H-
	Pseudomonas umsongensis	WP_083348463	--L---N--I-G-GP--	ES	-DPSA-RSA-Q--N-D-L-A-H-
	Pseudomonas aestus	WP_031320312	--LG--N--I---AP--	EG	-DPAP-REA-E----D-L-A-H-
	Pseudomonas segetis	WP_089358714	--L---S------A---	SA	K-PDE-RSA-E--S-D-L-A-Q-
	Pseudomonas bubulae	WP_095003078	--L---S----G-A---	EG	SNPAP-KDA-E----D-L-A-H-
	Pseudomonas granadensis	WP_090282432	--L---S------APT-	EG	-DAAP-RSA-E--N-D-L-A-H-
	Pseudomonas taetrolens	WP_048377892	--L---S----G-A---	EG	SDPAP-KEA-E----DVL-A-H-
	Pseudomonas gessardii	WP_076963011	--LG-----I---GP--	ES	-DPAP-RSA-E----D-L-A-H-
	Pseudomonas versuta	WP_060691035	--L---S----G-A---	EG	SNPAP-KDA-E----D-L-A-H-
	Pseudomonas protegens	WP_015636645	--LG--N--I---AP--	EG	SDPAP-REA-E----D-L-A-H-
	Pseudomonas cuatrocienegasensi	WP_069518555	--L---S---L----A-	AA	TDPQA-RDA-E--AGD-L-A-L-
	Pseudomonas saxonica	WP_146383970	--L---S----G-A---	EG	SNPAP-KEA-E----D-L-A-H-
	Pseudomonas baetica	WP_100845383	--L---S------APA-	EG	SDPAP-R-A-E--N-D-L-A-H-
	Pseudomonas psychrophila	WP_048351049	--L---S----G-A---	EG	SNPAP-KEA-E----D-L-A-H-
	Pseudomonas nitroreducens	WP_065085792	--I---S--Q-G-----	NA	-QPDV-KSA-E----D-L-A-D-
	Pseudomonas nitritireducens	WP_193452123	--I---S--Q-G-----	NA	-QPDV-KSA-E----D-L-A-D-

Other *Pseudomonadaceae*	*Pseudomonas argentinensis*	WP_074883341	--L---S------K-A-	GA	KDPGE-RSA-E----D-L-A---
	Pseudomonas alcaligenes	WP_061905015	--L---Q---A----S-	GA	TQPDA-REP-E--A-D-L-A-Q-
	Pseudomonas massiliensis	WP_040262401	------P---A--AK--	EA	-DPAP-----Q----D-L-A-H-
	Pseudomonas helmanticensis	WP_134175159	--L---S------APA-	EG	TDPAP-R-A-E--N-D-L-A-H-
	Pseudomonas zhaodongensis	WP_128119398	--L---S--------TV	TA	KDPQA-RE--E--D-DGL---M-
	Pseudomonas asuensis	WP_188865612	--L---S----G-A--I	DS	GKPDA-SEA-K--NSDVL-A-H-
	Pseudomonas atacamensis	WP_136492228	--L---S------APA-	ES	-DPAP-RSA-E--S-D-L-A-H-
	Pseudomonas peli	WP_090252541	--L---A-----V----	TA	NDPAA-REA-E--ATD-L-A-M-
	Pseudomonas anguilliseptica	WP_090385167	--L---A-----V---F	TA	NDPAA-REA-E--A-D-L-A-M-
	Pseudomonas cichorii	WP_025261332	--L---S--G-G-AQ--	ES	NDA-P-KE--E----D-I-A-H-
	Pseudomonas laurylsulfatiphila	WP_104449857	--L---N--I---AP--	EG	-DSGP-LAA-E--N-D-L-A-H-
	Pseudomonas jessenii	WP_090453905	--L---N--I---AP--	EG	-DPAP-HAA-E--N-D-L-A-H-
	Pseudomonas prosekii	WP_092271293	--L---S------AP--	EG	NDAAP-RDA-E--N-D-L-A-H-
	Pseudomonas kribbensis	WP_114881950	--L---S------APT-	EG	TDPAP-HAA-E--N-D-L-A-H-
	Pseudomonas helleri	WP_048369741	--L---S----G-A---	EG	SNPAP-KEA-E----D-L-A-H-
	Pseudomonas resinovorans	WP_041770400	--L---N---L----Q-	TS	-QPGA-TQA-E--D-D-L-A-D-
	Pseudomonas endophytica	WP_055101644	--L---S----G-A---	EG	SNPAP-KEA-E----D-L-A-H-
	Pseudomonas fulva	WP_013790612	--L---S------K-A-	GA	KDPGE-RSA-E----D-L-A---
	Pseudomonas punonensis	WP_073265663	--L---S------K-A-	GA	KDPGE-RSA-E----D-L-A---
	Pseudomonas shirazica	WP_139657452	--L---Q-----NA-QI	EG	SDPAA-RDA------D-L-A-H-
	Pseudomonas plecoglossicida	WP_013971367	--L---Q-----NA-QI	EG	SDPAA-REA------D-L-A-H-
	Pseudomonas monteilii	WP_013971367	--L---Q-----NA-QI	EG	SDPAA-REA------D-L-A-H-
	Pseudomonas moraviensis	WP_083353282	--L---S------APA-	ES	-DPAP-RSA-E--N-D-L-A-H-
	Pseudomonas asiatica	WP_015269236	--L---Q-----NA-QI	EG	SDPAA-REA------D-L-A-H-
	Pseudomonas zeshuii	WP_010795203	--L---S----G-A--I	DS	GKPDA-REA-K--SSDVL-A-H-
	Pseudomonas luteola	WP_074824601	--L---S----G-A--I	DS	GKPDA-REA-K--SSDVL-A-H-
	Pseudomonas pudica	WP_108480673	--L---Q-----NA-QI	EG	SDPAA-REA------D-L-A-H-
	Pseudomonas japonica	WP_042127817	--LG--E------AK--	EG	TDPAP--AA------D-L-A-H-
	Pseudomonas multiresinivorans	WP_169937291	--I---S--Q-G-A---	NA	-QPDA-KAA-E----D-L-A-D-
	Pseudomonas duriflava	WP_145136934	--L---S----G-A-TI	DA	GKPDA-REA-E--N-D-L---H-
	Pseudomonas mucidolens	WP_084379408	--LG-----I---AP--	ES	-DPAP-RAA-E----D-L-A-H-
	Pseudomonas oryzihabitans	WP_059314148	--L---N---AG-AA-I	DA	NDP-A-RQA-E------L-A-H-
	Pseudomonas inefficax	WP_133970572	--L---Q-----NA-QI	EG	SDPGA-REA------D-L-A-H-
	Pseudomonas straminea	WP_093502689	--L---S------K-A-	GA	KDPGE-RSA-E----D-L-A--T
	Pseudomonas rhizosphaerae	WP_043187949	--L---G--G-G-AQ-I	EA	SDPAA-R-A-E----D-L-A-H-
	Pseudomonas vranovensis	WP_028943582	--L---N----G-AK--	EG	-DPAP-R-A-E----D-L-A-H-
	Pseudomonas oleovorans	WP_150605783	--L---S------G-A-	AA	NDPQA-REA-E--A-D---A-Q-
	Pseudomonas khazarica	WP_134675431	--L---S------G-A-	AA	NDPQA-REA-E--A-D---A-Q-
	Pseudomonas alkylphenolica	WP_038608120	--L---N------AK--	EG	-DPAP-REA-E----D-L-A-H-
	Pseudomonas nosocomialis	WP_138409127	--L---S---E----AV	RA	S--DA-RML-E--D-DV--A-Y-
	Pseudomonas guineae	WP_090237871	--L---S---MGV---F	TA	NDPAV-REA-E--A-D-L-A-M-
	Pseudomonas coleopterorum	WP_090357242	--L---G--G-G-AQ-I	EA	SDSAA-RSA-E----D-L-A-H-
	Pseudomonas rhizoryzae	WP_058773425	--L---N---AG-AA-I	DA	SDP-V-RQA-E------L-A-H-
	Pseudomonas kirkiae	WP_165491548	--L---Q---L----T-	TQ	-DAGT-HDA-A--D-D-L-A-L-
	Pseudomonas psychrotolerans	WP_074529582	--L---N---AG-AA-I	DA	SDP-V-RQA-E------L-A-H-
	Pseudomonas saudiphocaensis	WP_037022768	--L---S---AV-A-T-	AA	KDPQE-REL-E--D-DGL-A---
	Pseudomonas xanthomarina	WP_073300730	--L---S-------DTI	TA	RDPQA-RD--E--DTDGL-A-M-
	Pseudomonas azotifigens	WP_028238498	--L-------A---DA-	DA	GDAGA-HDL-E--D-DGLMS-H-
	Pseudomonas donghuensis	WP_010220360	--L---Q------GK--	EG	TDPAP-REA-E----D-L-A-H-
	Pseudomonas wadenswilerensis	WP_115085738	--L---Q------GK--	EG	TDPAP-REA-E----D-L-A-H-
	Pseudomonas caspiana	WP_087272383	--L---E--G-G-AK-I	EG	TDP-A-KQA-E----D-L-A-H-
	Pseudomonas humi	WP_069863613	------S--G-G-AQ-I	TA	-QPDA-RAA-E--S-DGL-A-D-
	Pseudomonas citronellolis	WP_074981453	------S--G-G-AQ-I	TA	-QPDA-RAA-E--S-DGL-A-D-
	Pseudomonas panipatensis	WP_090262825	--L---S--G-G-VQ-I	TA	-QPDA-RAA-E--S-DGL-A-A-
	Pseudomonas entomophila	WP_011532664	--L---Q-----NAKQ-	EG	NDPAP-RAA-E------L-A-H-
	Pseudomonas denitrificans	WP_023103452	--L------I-G-----	TA	-QPDA-RAA-E--A-D-L-A-D-
	Pseudomonas reidholzensis	WP_119142669	--L---Q------AKQ-	EG	KDPAA-REA-E----D-L-A-H-
	Pseudomonas kunmingensis	WP_090521380	--L---S---L---DAF	GA	N-RET-GEA-E--D-DVL-A-H-
	Pseudomonas chloritidismutans	WP_023446275	--L---S---L---DAF	GA	N-RET-GEA-E--D-DVL-A-H-
	Pseudomonas ovata	WP_109512017	--L---G--G-G-A-H-	AD	KD-VDIKQA-E---TD-I-A-H-
	Pseudomonas pohangensis	WP_090194693	--L---Q------S-S-	SA	-QPDA-LPA-Q--A-D-L-G-V-
	Pseudomonas delhiensis	WP_089394055	------S--G-G-AQ-I	TA	-QPDA-LAA-E--S-DGL-A-D-
	Pseudomonas sichuanensis	WP_110993033	--L---Q-----NAKQ-	EG	NDPAP-REA-E------L-A-H-
	Pseudomonas parafulva	WP_028635046	--L---Q-----NAKQI	DA	SKPGP-REA-E----D-L-A-H-
	Pseudomonas asturiensis	WP_073165899	--L---S--GIG-AKT-	EG	TDAAP-KEA-E----D-I-A-H-
	Pseudomonas brassicacearum	WP_025215045	--L---S--I---AP--	EG	NDPAP-REA-E--ATD-L-A-H-
	Pseudomonas taiwanensis	WP_023379163	--L---Q-----NGKQ-	EG	KDPAP-RE--E----D-L-A-H-
	Pseudomonas juntendi	WP_110680059	--L---Q-----NAKQ-	EG	SDPAP-REAAE----D---A-H-
	Pseudomonas guariconensis	WP_090343588	--L---Q-----NAKQ-	EG	SDPAP-REA-E----D-L-A-H-
	Pseudomonas hydrolytica	WP_041981042	--L---S--ALGSADS-	LA	NDPKA-REA-E----D-L-A---
	Pseudomonas furukawaii	WP_003450660	--L---E---L----Q-	TS	-QPGA-TQA-E----D-L-A-D-
	Pseudomonas stutzeri	WP_014597242	--L---S---L----AF	GA	N-QQT-GEA-E--D-DVL-A-H-
	Pseudomonas putida	WP_016501107	--L---Q-----NAKQI	EG	SDPAP-REA-E----D-L-A-H-
	Pseudomonas qingdaonensis	WP_100635022	---G--N------AK--	EG	TDPAP-REA-E--A-D-L-A-H-
	Pseudomonas mediterranea	WP_047700499	--L---S--I---AP--	EG	SDPAP-RDA-E--A-D-L-A-H-
	Pseudomonas cerasi	WP_065349359	--L---S--GIGNAKT-	EG	TDPAP-KEA-E----D-I-A-H-
	Pseudomonas congelans	WP_054993517	--L---S--GIGNAKT-	EG	TDPAP-KEA-E----D-I-A-H-
	Pseudomonas savastanoi	WP_011169243	--L---S--SIGNAKT-	EG	TDAAP-KEA-E------I-A-H-
	Pseudomonas floridensis	WP_083184661	--L---S--GIG-AKT-	EG	TDAAP-KEA-D----D-I-A-H-
	Pseudomonas syringae	WP_025389175	--L---S--SIGDAKT-	EG	TDPAP-KTA-E----D-I-A-H-
	Pseudomonas coronafaciens	WP_024669906	--L---S--GIGDAKT-	EA	TDPAP-KEA-E----D-I-A-H-
	Pseudomonas mendocina	WP_003244304	--L---S---LG-ADV-	LA	NDPQA-REA-E--A-D-L-A-Q-
	Pseudomonas hydrolytica	WP_003244304	--L---S---LG-ADV-	LA	NDPQA-REA-E--A-D-L-A-Q-
	Pseudomonas tremae	WP_024690087	--L---S--GIGDAKT-	EA	TDPAP-KEA-E----D-I-A-H-
	Pseudomonas hunanensis	WP_014861002	--L---Q-----NAKQV	EG	NDPAP-REA-E----D-L-A-H-
	Pseudomonas guguanensis	WP_090431306	--L---S--ALGSADV-	LA	NDPQA-REA-E----D-L-A---
	Pseudomonas cannabina	WP_055001424	--L---S--SIGNAKT-	EG	TDPAP-KTA-E----D-I-A-H-
	Pseudomonas kilonensis	WP_053189706	--L---S--I---AP--	EG	SDPAP-RDA-E--ATD-L-A-H-
	Pseudomonas viridiflava	WP_088234780	--L---S--GIG-AKT-	EG	TDAAP-KEA-E----D-I-A-H-
	Pseudomonas mosselii	WP_096048517	--L---Q-----NAKL-	EG	NDPAP-REA-E----D-L-A-H-
	Pseudomonas balearica	WP_043221280	--I---G---M----AF	AG	KALEAVREP-E--D-DVV-A-H-
	Pseudomonas soli	WP_094011807	--L---Q-----NAKL-	EG	NDPAP-REA-E----D-L-A-H-
	Pseudomonas corrugata	WP_053192441	--L---S--I---AP--	EG	SDPAP-HDA-E--A-D-L-A-H-
	Pseudomonas thivervalensis	WP_053124291	--L---S--I---AP--	EG	SDPAP-REA-E--ATD-L-A-H-
	Pseudomonas sediminis	WP_099523365	--L---S--ALG-ADV-	LA	NDPQA-REA-E----D-L-A-Q-
	Pseudomonas capeferrum	WP_033700810	--L---Q-----NAKQI	EA	SDPAP-REA-E--A-D-L-A-H-
	Pseudomonas flexibilis	WP_039562949	--L---T--MLI--DV-	MA	HDAQE-RKA-Q---VD-L-A-Q-

Other *Pseudomonadaceae*	*Pseudomonas brassicae*	WP_163943428	--LG--Q------AG--	EG	TDPAP-REA-E--A-D-L-A-H-
	Pseudomonas tuomuerensis	WP_039606178	--L---T--MMI--DV-	MA	HDAQE-RKA-Q---VD-L-A-Q-
	Pseudomonas alcaliphila	WP_075748674	--L---S--ALGNADT-	LA	NDPQA-REA-E----D-L-A-Q-
	Pseudomonas meliae	WP_044323584	--L---S--G-GNAKT-	EG	TDAAP-KEA-E----D-I-A-H-
	Pseudomonas amygdali	WP_002554609	--L---S--G-GNAKT-	EG	TDAAP-KEA-E----D-I-A-H-
	Pseudomonas songnenensis	WP_126189809	--L---S---L---DAF	GA	N-RQT-GDA-E--D-DVL-A-H-
	Pseudomonas typographi	WP_190419882	--L---S--PL-SAQA-	EG	SDP---RSA-E----D-L-A-H-
	Pseudomonas indoloxydans	WP_108235117	--L---S--ALGNADV-	LA	NDPQA-REA-E----D-L-A-Q-
	Pseudomonas chengduensis	WP_017678993	--L---S--ALGSADT-	LA	NDPRA-GEA-E----D-L-A-Q-
	Pseudomonas caricapapayae	WP_055009593	--L---S--GIGNAKT-	EG	TDPAP-KEA-E----D-I-A-H-
	Pseudomonas composti	WP_074937077	--L---S--A-G-TDV-	LA	NDPQA-REA-E--A-D-L-A-Q-
	Pseudomonas abietaniphila	WP_074752972	--L---G--GIGNAKT-	DG	NDV-P-KQA-E---SD-I-A-H-
	Pseudomonas toyotomiensis	WP_074918098	--L---S--ALGSADT-	LA	NDPQA-REA-E----D-L-A-Q-
	Pseudomonas bohemica	WP_110949011	--L---G--GIGNAKT-	DG	SDVAP-KQA-E---SD-I-A-H-
	Pseudomonas lutea	WP_037013201	--L---G--GIGNAKT-	DG	NNVGP-KEA-E---SD-I-A-H-
	Pseudomonas graminis	WP_074890980	--L---G--GIGNAKT-	DG	NNVGP-KEA-E---SD-I-A-H-
	Pseudomonas avellanae	WP_024420670	--L---S--G-GDAKT-	EG	TDPAP-KEA-E----D-I-A-H-
	Pseudomonas leptonychotis	WP_136665024	--L---S-----V----	TA	NDPAA-RDA-E--A-D-L-A-V-
	Pseudomonas nitrititolerans	WP_058075316	--L---G--MLGVA-AA	QG	QDAQA-REA-E--D-D-L-A-S-
	Pseudomonas pharmafabricae	WP_101192325	--L---S---LISD-L-	QG	DAEAV--EPAV--E-DMQ-L-L-
	Pseudomonas fluvialis	WP_093986754	--L---S---LISD-L-	QG	DAEAV--EPAV--E-DMQ-L-L-

Figure-S92

Partial sequence alignments of DUF2066 domain-containing protein showing a 2aa del (highlighted), which is specific for the genus *Azotobacter*.

Group	Species	Accession	389		431
Genus *Azotobacter* (4/4)	*Azotobacter vinelandii*	WP_012702302	DGRSPEKLLQKAEQTMALAK		RSHGRYQFYIASVDREMRHRREL
	Azotobacter salinestris	WP_152389061	----------------T---		---S--------------Y----
	Azotobacter chroococcum	WP_089168837	----------------T---		--YS-------------------
	Azotobacter beijerinckii	WP_090619631	----------------T---		---N--H--------K-------
Other *Pseudomonadaceae*	*Pseudomonas aeruginosa*	WP_003094007	--ETT-----------T---	T	--RN----------S---R----
	Pseudomonas floridensis	WP_083186427	--D-------------T---	A	--RN----------S---R----
	Pseudomonas viridiflava	WP_004881457	--D-------------T---	A	--RN----------C---R----
	Pseudomonas fulva	WP_013790283	--D-------------T---	S	--RN----------S---R----
	Pseudomonas straminea	WP_093506112	--D-------------T---	S	--RN----------S---R----
	Pseudomonas argentinensis	WP_074882755	--D-------------T---	S	--RN----------S---R----
	Pseudomonas punonensis	WP_073265234	--D-------------T---	S	--RN----------S---R----
	Pseudomonas flavescens	WP_084304759	--D-------------T---	S	--RN----------S---R----
	Pseudomonas daroniae	WP_131179901	--G-------------T---	S	--RN----------S---R----
	Pseudomonas seleniipraecipitan	WP_070881377	--D-------------T---	S	--RS----------S---R----
	Pseudomonas fuscovaginae	WP_019361971	--D-T-----------T---	T	--RN----------S---R----
	Pseudomonas asplenii	WP_090201964	--D-T-----------T---	T	--RN----------S---R----
	Pseudomonas mosselii	WP_062364497	--D-T-----------T---	A	--RN----------S---R----
	Pseudomonas caspiana	WP_087264814	--D-T-----------T---	S	--RN----------S---R----
	Pseudomonas japonica	WP_042123007	--D-T-----------T---	T	--RN----------S---R----
	Pseudomonas brassicae	WP_163947220	--D-T-----------T---	S	--RN----------S---R----
	Pseudomonas qingdaonensis	WP_054914822	--D-T-----------T---	S	--RN----------S---R----
	Pseudomonas lurida	WP_034109033	--D-T-----------T---	T	--RN----------S---R----
	Pseudomonas alkylphenolica	WP_038607324	--D-T-----------T---	T	--RN----------S---R----
	Pseudomonas vranovensis	WP_028943327	--D-T-----------T---	T	--RN----------S---R----
	Pseudomonas lutea	WP_037014598	--D-T-----------T---	T	--RN----------S---R----
	Pseudomonas sichuanensis	WP_110993719	--D-T-----------T---	A	--RN----------S---R----
	Pseudomonas piscis	WP_022640578	--D-T-----------T---	S	--RN----------S---R----
	Pseudomonas aestus	WP_022640578	--D-T-----------T---	S	--RN----------S---R----
	Pseudomonas soli	WP_038707431	--D-T-----------T---	A	--RN----------S---R----
	Pseudomonas cannabina	WP_007248905	--D-T-----------T---	A	--RN----------S---R----
	Pseudomonas juntendi	WP_029887117	--D-T-----------T---	A	--RN----------S---R----
	Pseudomonas donghuensis	WP_010220831	--D-T-----------T---	A	--RN----------S---R----
	Pseudomonas protegens	WP_011063081	--D-T-----------T---	T	--RN----------S---R----
	Pseudomonas entomophila	WP_011532440	--D-T-----------T---	A	--RN----------S---R----
	Pseudomonas wadenswilerensis	WP_115085465	--D-T-----------T---	A	--RN----------S---R----
	Pseudomonas tremae	WP_054996970	--D-T-----------T---	A	--RN----------S---R----
	Pseudomonas thivervalensis	WP_053119168	--D-T-----------T---	S	--RN----------S---R----
	Pseudomonas taiwanensis	WP_023378871	--D-T-----------T---	A	--RN----------S---R----
	Pseudomonas graminis	WP_074884554	--D-T-----------T---	T	--RN----------S---R----
	Pseudomonas cerasi	WP_044310414	--D-T-----------T---	A	--RN----------S---R----
	Pseudomonas taeanensis	WP_025166791	--D-T-----------T---	N	--RN----------S---R----
	Pseudomonas coronafaciens	WP_024670858	--D-T-----------T---	A	--RN----------S---R----
	Pseudomonas inefficax	WP_112250360	--D-T-----------T---	A	--RN----------S---R----
	Pseudomonas saponiphila	WP_092311067	--D-T-----------T---	S	--RN----------S---R----
	Pseudomonas kilonensis	WP_018610956	--D-T-----------T---	S	--RN----------S---R----
	Pseudomonas hunanensis	WP_003255193	--D-T-----------T---	A	--RN----------S---R----
	Pseudomonas extremorientalis	WP_071489433	--D-T-----------T---	T	--RN----------S---R----
	Pseudomonas corrugata	WP_024781209	--D-T-----------T---	T	--RN----------S---R----
	Pseudomonas batumici	WP_040070316	--D-T-----------T---	T	--RN----------S---R----
	Pseudomonas putida	WP_016498084	--D-T-----------T---	A	--RN----------S---R----
	Pseudomonas borbori	WP_090503461	--D-T-----------T---	S	--RN----------S---R----
	Pseudomonas mediterranea	WP_047701845	--D-T-----------T---	T	--RN----------S---R----
	Pseudomonas kairouanensis	WP_135289400	--D-T-----------T---	T	--RN----------S---R----
	Pseudomonas asturiensis	WP_073168212	--D-T-----------T---	A	--RN----------S---R----
	Pseudomonas abietaniphila	WP_074755472	--D-T-----------T---	T	--RN----------S---R----
	Pseudomonas pudica	WP_046616943	--D-T-----------T---	A	--RN----------S---R----
	Pseudomonas capeferrum	WP_033702797	--D-T-----------T---	A	--RN----------S---R----
	Pseudomonas brassicacearum	WP_025212279	--D-T-----------T---	A	--RN----------S---R----
	Pseudomonas shirazica	WP_015271685	--D-T-----------T---	A	--RN----------S---R----
	Pseudomonas plecoglossicida	WP_015271685	--D-T-----------T---	A	--RN----------S---R----
	Pseudomonas monteilii	WP_015271685	--D-T-----------T---	A	--RN----------S---R----
	Pseudomonas asiatica	WP_015271685	--D-T-----------T---	A	--RN----------S---R----
	Pseudomonas umsongensis	WP_020797458	--N-T-----------T---	T	--RN----------T---R----
	Pseudomonas marginalis	WP_064053712	--D-T-----------T---	T	--RN----------S---R----
	Pseudomonas chlororaphis	WP_009050526	--D-T-----------T---	S	--RN----------S---R----
	Pseudomonas avellanae	WP_005620256	--D-T-----------T---	A	--RN----------S---R----
	Pseudomonas silesiensis	WP_064679451	--N-T-----------T---	T	--RN----------T---R----
	Pseudomonas resinovorans	WP_016494543	--D-T-----------T---	S	--RN----------S---R----
	Pseudomonas guariconensis	WP_090344922	--D-T-----------T---	A	--RN----------S---R----
	Pseudomonas furukawaii	WP_003451373	--D-T-----------T---	S	--RN----------S---R----
	Pseudomonas typographi	WP_190416901	--D-T-----------T---	S	--RN----------S---R----
	Pseudomonas congelans	WP_054994037	--D-T-----------T---	A	--RN----------S---R----
	Pseudomonas caricapapayae	WP_055009586	--D-T-----------T---	A	--RN----------S---R----
	Pseudomonas parafulva	WP_028635150	--D-T-----------T---	A	--RS----------S---R----
	Pseudomonas coleopterorum	WP_090356570	--D-T-----------T---	V	--RN----------T---R----
	Pseudomonas rhizosphaerae	WP_043186909	--D-T-----------T---	V	--RN----------T---R----
	Pseudomonas cichorii	WP_025261674	--D-T-----------T---	T	--RN----------S---R----
	Pseudomonas cuatrocienegasensi	WP_069519980	--D-T-----------T---	S	--RN----------S---R----
	Pseudomonas cedrina	WP_076949704	--D-T-----------T---	T	--RN----------S---R----
	Pseudomonas proteolytica	WP_029296372	--D-T-----------T---	T	--RN----------S---R----
	Pseudomonas brenneri	WP_032862546	--D-T-----------T---	S	--RN----------S---R----
	Pseudomonas benzenivorans	WP_090447594	--D-T-----------T---	S	--RN----------S---R----
	Pseudomonas otitidis	WP_044409444	--D-T-----------T---	S	--RN----------S---R----
	Pseudomonas thermotolerans	WP_017936897	--D-T-----------T---	S	--RN----------S---R----
	Pseudomonas humi	WP_043314055	--E-T-----------T---	T	--RN----------S---R----
	Pseudomonas delhiensis	WP_089389705	--E-T-----------T---	T	--RN----------S---R----
	Pseudomonas citronellolis	WP_074982320	--E-T-----------T---	T	--RN----------S---R----
	Pseudomonas jinjuensis	WP_084314595	--ETA------G----T---	A	--RH----------S--------
	Pseudomonas leptonychotis	WP_136663011	--D-T-----------T---	S	--RN----------S---R----
	Pseudomonas veronii	WP_017847174	--D-T-----------T---	S	--RN----------S---R----
	Pseudomonas panacis	WP_017847174	--D-T-----------T---	S	--RN----------S---R----
	Pseudomonas dryadis	WP_131175994	--D-T-----------T---	S	--RN----------S---R---M
	Pseudomonas synxantha	WP_057023826	--DTT-----------T---	S	--RN----------S---R----
	Pseudomonas guineae	WP_090239740	--D-T-----------T---	S	--RN----------S---R----
	Pseudomonas lundensis	WP_047282315	--D-S-----------T---	S	--RS----------T---R----

Other *Pseudomonadaceae* (bracket spanning *Pseudomonas peli* through *Pseudomonas weihenstephanensis*)

Pseudomonas libanensis	WP_057013676	--DTT-----------T--- S --RN----------S---R----
Pseudomonas peli	WP_090249034	--D-T-----------T--- S --RN----------S---R----
Pseudomonas vancouverensis	WP_093226397	--D-T-----------T--- T --RN----------T---R----
Pseudomonas jessenii	WP_090457513	--D-T-----------T--- T --RN----------T---R----
Pseudomonas laurylsulfatovoran	WP_103396356	--D-T-----------T--- T --RN----------T---R----
Pseudomonas mohnii	WP_047532630	--D-T-----------T--- T --RN----------T---R----
Pseudomonas migulae	WP_084323900	--D-T-----------T--- T --RN----------T---R----
Pseudomonas reinekei	WP_075945504	--D-T-----------T--- T --RN----------T---R----
Pseudomonas edaphica	WP_138449653	--D-T-----------T--- S --RN----------S---R----
Pseudomonas nitroreducens	WP_024762590	--ETT-----------T--- S --RN----------S---R----
Pseudomonas nitritireducens	WP_024762590	--ETT-----------T--- S --RN----------S---R----
Pseudomonas frederiksbergensis	WP_071553604	--D-T-----------T--- T --RN----------S---R----
Pseudomonas anguilliseptica	WP_090382541	--D-T-----------T--- S --RN----------S---R----
Pseudomonas salomonii	WP_065930472	--D-T-----------T--- T --RN----------S---R----
Pseudomonas lini	WP_038981897	--D-T-----------T--- T --RN----------T---R----
Pseudomonas nabeulensis	WP_135309916	--D-T-----------T--- T --RN----------S---R----
Pseudomonas grimontii	WP_090400526	--D-T-----------T--- S --RN----------S---R----
Pseudomonas tolaasii	WP_080520192	--D-T-----------T--- S --RN----------S---R----
Pseudomonas syringae	WP_025388949	--D-T-----------T--- A --RN----------S---R----
Pseudomonas laurylsulfatiphila	WP_104449268	--D-T-----------T--- T --RN----------T---R----
Pseudomonas lactis	WP_003193777	--D-T-----------T--- S --RN----------S---R----
Pseudomonas allii	WP_179030397	--D-T-----------T--- T --RN----------S---R----
Pseudomonas cremoricolorata	WP_038410772	--D-T-----------T--- T --RN----------S---R---M
Pseudomonas kitaguniensis	WP_152746027	--D-T-----------T--- S --RN----------S---R----
Pseudomonas baetica	WP_100845224	--D-T-----------T--- T --RN----------S---R----
Pseudomonas prosekii	WP_092278685	--D-T-----------T--- T --RN----------S---R----
Pseudomonas haemolytica	WP_034115712	--D-T-----------T--- S --RN----------S---R----
Pseudomonas sivasensis	WP_099602785	--D-T-----------T--- S --RN----------S---R----
Pseudomonas antarctica	WP_064453881	--D-T-----------T--- S --RN----------S---R----
Pseudomonas palleroniana	WP_060754140	--D-T-----------T--- S --RN----------S---R----
Pseudomonas kribbensis	WP_085709556	--D-T-----------T--- T --RN----------S---R----
Pseudomonas koreensis	WP_016773430	--D-T-----------T--- T --RN----------S---R----
Pseudomonas helmanticensis	WP_038366843	--D-T-----------T--- T --RN----------S---R----
Pseudomonas granadensis	WP_090285796	--D-T-----------T--- T --RN----------S---R----
Pseudomonas atacamensis	WP_016773430	--D-T-----------T--- T --RN----------S---R----
Pseudomonas indica	WP_084335652	--D-A-----------T--- S --RN----------S---R----
Pseudomonas orientalis	WP_057723797	--D-T-----------T--- S --RN----------S---R----
Pseudomonas moorei	WP_090324858	--D-T-----------T--- T --RN----------T---R----
Pseudomonas fluorescens	WP_053257708	--D-T-----------T--- S --RN----------S---R----
Pseudomonas multiresinivorans	WP_169936646	--ETT-----------T--- S --RN----------S---R----
Pseudomonas moraviensis	WP_042607344	--D-T-----------T--- T --RN----------S---R----
Pseudomonas carnis	WP_106118368	--D-T-----------T--- S --RN----------S---R----
Pseudomonas trivialis	WP_049711789	--D-T-----------T--- T --RN----------S---R----
Pseudomonas ficuserectae	WP_054994687	--DNT-----------T--- A --RN----------S---R----
Pseudomonas oleovorans	WP_037049246	--D-T-----------T--- S --RN----------S---R----
Pseudomonas khazarica	WP_037049246	--D-T-----------T--- S --RN----------S---R----
Pseudomonas gingeri	WP_017125975	--D-T-----------T--- T --RN----------S---R----
Pseudomonas cremoris	WP_185707984	--D-T-----------T--- T --RN----------S---R----
Pseudomonas canadensis	WP_028617709	--D-T-----------T--- T --RN----------S---R----
Pseudomonas azotoformans	WP_028617709	--D-T-----------T--- T --RN----------S---R----
Pseudomonas paralactis	WP_057704253	--D-T-----------T--- S --RN----------S---R----
Pseudomonas arsenicoxydans	WP_090180839	--D-T-----------T--- T --RN----------T---R----
Pseudomonas mendocina ymp	WP_003246027	--D-T-----------T--- S --RN----------S---R----
Pseudomonas hydrolytica	WP_003246027	--D-T-----------T--- S --RN----------S---R----
Pseudomonas yamanorum	WP_063027689	--D-T-----------T--- S --RN----------S---R----
Pseudomonas simiae	WP_010208167	--D-T-----------T--- T --RN----------S---R----
Pseudomonas savastanoi	WP_011169411	--DNT-----------T--- A --RN----------S---R----
Pseudomonas rhodesiae	WP_034100084	--D-T-----------T--- S --RN----------S---R----
Pseudomonas fildesensis	WP_048726891	--D-T-----------T--- S --RN----------S---R----
Pseudomonas reactans	WP_095017410	--D-T-----------T--- T --RN----------S---R----
Pseudomonas mandelii	WP_010457618	--D-T-----------T--- T --RN----------T---R----
Pseudomonas extremaustralis	WP_010567097	--D-T-----------T--- S --RN----------S---R----
Pseudomonas meliae	WP_044345427	--DNT-----------T--- A --RN----------S---R----
Pseudomonas balearica	WP_043218966	--D-------------T--- S -ARN-----V--I-K---Q----
Pseudomonas amygdali	WP_005734694	--DNT-----------T--- A --RN----------S---R----
Pseudomonas saudiphocaensis	WP_037026149	--N-T-----------T--- S --RN--------I-S---E----
Pseudomonas sediminis	WP_099525729	--D-T-----------T--- S --RN----------S---R----
Pseudomonas hydrolytica	WP_017362763	--D-T-----------T--- S --RN----------S---R----
Pseudomonas composti	WP_037003169	--D-T-----------T--- S --RN----------S---R----
Pseudomonas toyotomiensis	WP_072425838	--D-T-----------T--- S --RN----------S---R----
Pseudomonas sihuiensis	WP_017675061	--D-T-----------T--- S --RN----------S---R----
Pseudomonas indoloxydans	WP_108233991	--D-T-----------T--- S --RN----------S---R----
Pseudomonas chengduensis	WP_017675061	--D-T-----------T--- S --RN----------S---R----
Pseudomonas kuykendallii	WP_090227539	--D-T-----------T--- S --RN----------S---R----
Pseudomonas gessardii	WP_076962966	--D-T-----------T--- S --RN----------S---R----
Pseudomonas alcaliphila	WP_075749129	--D-T-----------T--- S --RN----------S---R----
Pseudomonas bohemica	WP_110948551	--D-T-----------T--- T --RN----------S-I-R----
Pseudomonas guguanensis	WP_090434052	--D-T-----------T--- S --RN----------S---R----
Pseudomonas poae	WP_060549663	--D-T-----------T--- S --RN----------S-I-R----
Pseudomonas ovata	WP_109511355	--D-T-----------T--- T --RN----------S-I-R----
Pseudomonas panipatensis	WP_090267952	--E-T-----------T--- T --RN----------S---R----
Pseudomonas mucidolens	WP_084381082	--D-T-----------T--- S --RS----------S---R----
Pseudomonas knackmussii	WP_043256917	--E-T-----------T--- T --RN----------S---R----
Pseudomonas kirkiae	WP_131183578	--D-T-----------T--- S -A-------V--I-SA--E----
Pseudomonas alcaligenes	WP_061903556	--D-T-----------T--- S --RN----------S---R----
Pseudomonas costantinii	WP_071484403	--D-T------S----T--- S --RN----------S---R----
Pseudomonas denitrificans	WP_003094007	--ETT-----------T--- T --RN----------S---R----
Pseudomonas fluvialis	WP_093985080	--ENT-----------T--- S --RN----------S---R----
Pseudomonas pharmafabricae	WP_101192925	--ENT-----------T--- S --RN----------S---R----
Pseudomonas azotifigens	WP_028239718	--D-T-----------T--- S --RS-----V--I-S---Q----
Pseudomonas agarici	WP_060782228	--A-T-----------T--- T --RN----------S-I-R----
Pseudomonas chloritidismutans	WP_023444112	--DNT-----------T--- S --RN-----V--I-S---A----
Pseudomonas kunmingensis	WP_090522231	--DNT-----------T--- S --RN-----V--I-S---A----
Pseudomonas songnenensis	WP_122098203	--DNT-----------T--- S --RN-----V--I-S---A----
Pseudomonas nosocomialis	WP_138409541	--D-A-----------T--- N --RN-----V--I-S---Q----
Pseudomonas taetrolens	WP_048377937	--DTS-Q---R-----T--- N S-R---------------RI---
Pseudomonas weihenstephanensis	WP_048362816	--DTS-----------T--- S --RS----------S-I-R----

Group	Species	Accession	Sequence
	Pseudomonas zhaodongensis	WP_128121862	--S-T-----------T--- S --RN-----V--I-S---T----
Other *Pseudomonadaceae*	*Pseudomonas stutzeri*	WP_014597467	--DNT-----------T--- S --RN-----V--I-SQ--A----
	Pseudomonas pseudoalcaligenes	WP_003462544	--D-T-----------T--- S --RN----------S---R---Q
	Pseudomonas matsuisoli	WP_188981637	--D-A-----------T--- V --RN----------L---R--T-
	Pseudomonas fragi	WP_016779806	--DTT-Q---------T--- S Y-RS--------------RM---
	Pseudomonas deceptionensis	WP_048358927	--DTT-Q---------T--- S Y-RS--------------RM---
	Pseudomonas bubulae	WP_130871923	--DTT-Q---------T--- S Y-RS--------------RM---
	Pseudomonas xanthomarina	WP_073302572	--DNT-----------T--- N --RN-----V--I-S---T----
	Pseudomonas segetis	WP_010486244	--D-T-----------T--- S --RN----------S---RL---
	Pseudomonas saxonica	WP_146384019	-SDTA-Q---------T--- T --R------------Q--RM---
	Pseudomonas psychrophila	WP_019827797	--DTT-Q---------T--- S Y-RS--------------RM---
	Pseudomonas marincola	WP_090512987	--DNT-Q---------T--- S --RN----------S-I-R--G-
	Pseudomonas nitritireducens	WP_014853755	--D-T-----------T--- A -ARN-----V--I-S---E--K-
	Pseudomonas hussainii	WP_071871953	--D-A-----------T--- T --RN--------L-S---Q----
	Pseudomonas jilinensis	WP_119702066	--DNA-----------M--- A --RN--------L-S---I----
	Pseudomonas salegens	WP_092386258	--DNA-----------M--- G --RN--------L-S---V----
	Pseudomonas massiliensis	WP_040261867	--D-A-----------TV-- S --RN-----V----S---R--A-
	Pseudomonas salina	WP_150278492	--DDA-----------M--- A --RN--------L-S---V----
	Pseudomonas pertucinogena	WP_188635855	--MD-S----------M--- T --RN----------S---M----
	Pseudomonas pelagia	WP_022962829	--DDA-----------M--- A --RN--------L-S---I----
	Pseudomonas yangmingensis	WP_093476174	--QNA-----------M--- A --RN--------L-S---V----
	Pseudomonas xinjiangensis	WP_093397333	--DDA-----------M--- A --RN--------L-S---I----
	Pseudomonas formosensis	WP_090537670	--MD-G----------M--- A --RN-F--------T---M----
	Pseudomonas sabulinigri	WP_092284906	--DNA-----------M--- A --RN--------L-S---V----
	Pseudomonas profundi	WP_150299243	--ADA-TI--------M--- A --RS--------L-A---V----
	Pseudomonas aestusnigri	WP_088273612	--DNA-----------M--- A --RN--------L-S---V----
	Pseudomonas litoralis	WP_090272162	---DAG----------M--- V --RN----------S---V----
	Pseudomonas oceani	WP_104737888	--DNA-----------M--- A --RN--------L-S---V----
	Pseudomonas abyssi	WP_096003609	--DDA-----------M--- A --RN--------L-S---V----
	Pseudomonas gallaeciensis	WP_118129359	--DDA-----------M--- A --RN--------L-S---V----
	Pseudomonas pachastrellae	WP_083723395	--DDA-----------M--- A --RN--------L-S---V----
	Pseudomonas flexibilis	WP_039559578	--ADSGD------L--T--- R -ARN--------------R----
	Pseudomonas tuomuerensis	WP_039606004	--ADSGD------L--T--- R -ARN--------------R----
	Pseudomonas bauzanensis	WP_074778734	--LDAG----------M--- T --RN----------S---D----
	Azomonas macrocytogenes	WP_183167098	--DHA-----------T--- Q --RN-----V--L-CKI-Q----
	Pseudomonas pohangensis	WP_090196065	--DNT-Q--L------T--- T --RN----F---L-N-V-R----
	Pseudomonas saudimassiliensis	WP_044500274	--LDAG----------M--- A --RN----------S---E--Q-
	Pseudomonas zeshuii	WP_010795281	--QTADQ-M-R-------V- T QG-SG-K--V----QDI-F--G-
	Pseudomonas luteola	WP_074824746	--QTADQ-M-R-------V- T QG-SG-K--V----QDI-F--G-
	Pseudomonas guangdongensis	WP_090211230	--H-A-Y---R-----T--- Q QARSG----V----QQ--QQ---
	Pseudomonas caeni	WP_028244216	--DNT-V---Q-----Q--- K GARN----F---I-M---K--R-
	Pseudomonas endophytica	WP_055100919	--DTS-Q---------T--- N --R---------------R----
	Pseudomonas asuensis	WP_188865538	--QTADQ-M-R-------V- T QGQNG-K--V----QDI-F--G-

Figure-S93

Partial sequence alignments of GGDEF domain-containing phosphodiesterase showing a 1aa del (highlighted), which is specific for the genus *Azotobacter*.

Group	Species	Accession	131		167
**Genus *Azotobacter* (4/4)**	*Azotobacter vinelandii*	WP_012700992	GRYLFGGYQDSSPPFVKD	N	TSGAVAYQGDEGLILQQ
	Azotobacter salinestris	WP_152385867	----------N-------	S	LT---S----D-VVA--
	Azotobacter chroococcum	WP_089167998	----------K-------	A	LT---S----A-VVA--
	Pseudomonas guangdongensis	WP_090212237	--------R-N-------	P	---R-T-I--T-VAE-K
**Other *Pseudomonadaceae***	*Pseudomonas oryzae*	WP_090347759	-------H--GA----Q-		AD-R-S-I----VKA--
	Pseudomonas linyingensis	WP_090310907	-------H--GT----Q-		AD-R-G-V--D-VKA--
	Pseudomonas sagittaria	WP_092428453	--------R-GT----Q-		AD-R-S-V--T-VKA--
	Pseudomonas oceani	WP_104739766	-E-------S-E-----N		AD-TYS------QRSV-
	Pseudomonas veronii	WP_079444655	-N---S---SQ--A-AT-		AN-DLV----NNAVT--
	Pseudomonas acidophila	WP_096722202	-N-----F-NT-A--STS		AT---V-T--A-NRTM-
	Pseudomonas extremaustralis	WP_010567398	-N---S---SQ--A-ATN		AA-ELV----SNAVT-L
	Pseudomonas pelagia	WP_022963236	-E-----F-SQT---I-E		PD-TYT------QRSI-
	Pseudomonas aestusnigri	WP_088276680	-E-----F-S-EA----N		PD-TYT------QRSV-
	Pseudomonas nabeulensis	WP_135311438	-N---S---SQ--A-ATN		DA-ELV----TNAVT-L
	Pseudomonas furukawaii	WP_003456933	-E-I-S-F-GKTQ---RN		AD-SYS-E----QRKL-
	Pseudomonas songnenensis	WP_102821437	-E---S-F-GKTQ---R-		GA-SYS------QRKL-
	Pseudomonas salina	WP_150277448	-E-----F-SQT----RE		PN-TYT-E--Q-QRSI-
	Pseudomonas saudimassiliensis	WP_044500597	-E-------SD-E----N		GD-SYT-A----QRFI-
	Pseudomonas punonensis	WP_073264297	-E---S-F-GKAE---RN		AD-TYS------QRKLE
	Pseudomonas litoralis	WP_090273315	-E-------SD-Q----N		AD-SYT-A----QRSI-
	Pseudomonas dryadis	WP_131174010	-E---S-F-GKAE---RN		AD-TYS------QRKLE
	Pseudomonas bauzanensis	WP_036990519	-E-------SDTQ----N		PD-SYS-A----QRSI-
	Pseudomonas marincola	WP_090508674	-E---A-F-GK-Q---RN		PD-SYS-T----QRTL-
	Pseudomonas chloritidismutans	WP_023445467	-E---S-F-GKTQ---RA		AD-SYS------QRKL-
	Pseudomonas zhaodongensis	WP_128119199	-E---S-F-GKTQ---RA		AD-SYS------QRKL-
	Pseudomonas pharmafabricae	WP_101192434	-E---A-F-GKTQ---RQ		AD-SY-------QRKI-
	Pseudomonas fluvialis	WP_093985748	-E---A-F-GKTQ---RQ		AD-SY-------QRKI-
	Pseudomonas guineae	WP_090238611	-E---S-F-GK-Q---RE		SD-SYR------QRKL-
	Pseudomonas indica	WP_084334754	-E-----F-GKTQ--IRN		AD-GYS-V----QRKI-
	Pseudomonas fulva	WP_013792187	-E---S-F-GK-E---RN		AD-TYS------QRKLE
	Pseudomonas multiresinivorans	WP_169942746	-N---S-SKSTVQ---QN		AD-SFS----QSTLKL-
	Pseudomonas straminea	WP_093502115	-E---S-F-GKAE---RN		AD-TYS------QRKLE
	Pseudomonas salegens	WP_092386841	-E-------SDQA---RE		SD-SYS------QRSI-
	Pseudomonas argentinensis	WP_074880682	-E---S-F-GKAE---RN		AD-TYS------QRKLE
	Pseudomonas aeruginosa	WP_003086414	-K---S-S-G-VQ---RN		ED-TYS-M---SQREV-
	Pseudomonas otitidis	WP_074968163	-K-----S-ADTA---RN		AD-TYS-L----RREV-
	Pseudomonas kunmingensis	WP_090519053	-E---S-F-GKTQ---RT		GD-SYS------QRKL-
	Pseudomonas pertucinogena	WP_188635156	-E-----FRSDTQ---RQ		PD-SYS------QRSI-
	Pseudomonas stutzeri	WP_014596207	-E---S-F-GKTQ---RA		AD-SYS------QRKL-
	Pseudomonas luteola	WP_074827280	-Q-----AKS--A--TQN		PD-SYS-T--Q-TLEV-
	Pseudomonas matsuisoli	WP_188981353	-K---S-SMGDTQ--MRN		AD-TYS------QRNV-
	Pseudomonas thermotolerans	WP_017939546	-E---S-F-GKTQ--LRN		AD-TYV----D-QRKL-
	Pseudomonas nitroreducens	WP_084357599	-N---S-SKSTVQ---QN		AD-SFS----QSTLKL-
	Pseudomonas monteilii	WP_024087475	-D-M---SKS-T--Y-RN		AD-TYS----QTQLSL-
	Pseudomonas citronellolis	WP_058072089	-N---S-SKSTVQ--IQN		AD-TYS----QSSLNL-
	Pseudomonas benzenivorans	WP_090447453	-E---A-F-GKTQ---RQ		AD-SYS-E----QRKL-
	Pseudomonas gallaeciensis	WP_118130384	-E-------S-EQ----N		PD-SYS------QRSI-
	Pseudomonas pachastrellae	WP_083726605	-E-------S-EQ----N		PD-SYS------QRSI-
	Pseudomonas abyssi	WP_096004238	-E-------S-EQ----N		PD-SYS------QRSI-
	Pseudomonas duriflava	WP_145142842	-E-----F-GKTQ-Y-RN		-D-SY--V----QRSV-
	Pseudomonas resinovorans	WP_016493887	-E-I-S-F-GKTQ---RN		AD-SYS-E----QRKL-
	Pseudomonas sabulinigri	WP_092282922	-E-------SNQA----N		PD-SYS------QRSV-
	Pseudomonas kairouanensis	WP_135292368	-N---S---SQ--A-AT-		AD-NLV----SNAVT-L
	Pseudomonas denitrificans	WP_023115483	-K---S-S-GDTQ---RN		PD-TYS-N----QREV-
	Pseudomonas hunanensis	WP_004575773	-D-M---SKS-T--Y-RN		AD-TYS----QTQLSL-
	Pseudomonas formosensis	WP_090537887	-E-----FKSDAR--ERQ		AD-SYR------QRSI-
	Pseudomonas vranovensis	WP_028945505	-N-M---SKT----Y-RN		SD-TYS-H--QTQLSL-
	Pseudomonas mosselii	WP_096049610	-D-M---TKT----Y-RN		AD-TYS----QTQLSL-
	Pseudomonas jinjuensis	WP_084311359	-K---S-S-GDTQ--IRN		AD-TYS----DSQRQV-
	Pseudomonas sichuanensis	WP_110991881	-D-M---TKT----Y-RN		AD-SYS----QTQLSL-
	Pseudomonas peli	WP_090248266	-E---S-FLGKTE--LRN		PD-TYS-------RSL-
	Pseudomonas umsongensis	WP_083348428	-N-M-S-SKT-I--Y-RN		ND-TYT----DTQLSL-
	Pseudomonas balearica	WP_043219262	-E---S-F-GKTQ---RT		GD-SYR-D----QRKL-
	Pseudomonas delhiensis	WP_089391795	-N---S-SKSTVQ--IQN		AD-TYS----QSALSL-
	Pseudomonas caeni	WP_022965708	-E---A-F-GKTQ---RQ		AD-SFS-E----QRKL-
	Pseudomonas plecoglossicida	WP_041505713	-E-M---SKNAN--Y-RN		AD-TYS----QTQLSL-

Figure-S94

Partial sequence alignments of Flagellar hook-associated protein FlgL showing a 1aa Ins (highlighted), which is specific for the genus *Azotobacter*.

			87		130
**Genus *Azotobacter* (4/4)**	*Azotobacter vinelandii*	WP_012699059	PTEAARQFATLDHFSDGRAALRL		FDDGGPQSDGDFLERGDWPAR
	Azotobacter salinestris	WP_152387642	------------------V----		V---M--R-----D-S-----
	Azotobacter beijerinckii	WP_090621890	--------------C---L----		V---E--R--------E-H--
	Azotobacter chroococcum	WP_089169531	--------------C------L-		VE--V--R-------S-----
**Other *Pseudomonadaceae***	*Azomonas agilis*	WP_144571869	--V----L-------G----VNI	ITG	GN-AEQ-R-----SHDERY--
	Azomonas macrocytogenes	WP_183165189	--L---KL----NLL---L--NV	ISG	GS-AEQRK-----DHDQRY--
	Pseudomonas kirkiae	WP_131186072	--F----------L-Q----INV	ITG	G-S-DL-R-----DKDARY--
	Pseudomonas dryadis	WP_131176886	--F----------L-R---SINV	ITG	G-S-DL-R-----DKDARY--
	Pseudomonas straminea	WP_093501745	--F----------L-R----INV	ITG	G-S-DL-R---Y-DKDARY--
	Pseudomonas flavescens	WP_084307565	--F----------L-R---SINV	ITG	G-S-DL-R-----DKDARY--
	Pseudomonas daroniae	WP_131192323	--F----------L-R---SINV	ITG	G-S-DL-R-----DKDARY--
	Pseudomonas parafulva	WP_028633586	--F---------QL-R----INV	ITG	G-S-DL-R-----DKDARY--
	Pseudomonas japonica	WP_042130451	--F----------L-R---SINV	ITG	G-S-DL-R-----DKDARY--
	Pseudomonas punonensis	WP_073264654	--F----------L-R---SINV	ITG	G-S-DL-R---Y-DKDARY--
	Pseudomonas hunanensis	WP_004374416	--F---------QL-R---SINV	ITG	G-S-DL-R-----DKEARY--
	Pseudomonas putida	WP_016499880	--F---------QL-G---SINV	ITG	G-S-DL-R-----DKDARY--
	Pseudomonas inefficax	WP_133972333	--F---------QL-R---SINV	ITG	G-S-DL-R-----TKEARY--
	Pseudomonas reidholzensis	WP_119141427	--F---------QL-R---SINV	ITG	G-S-DL-R---Y-DKDARY--
	Pseudomonas cremoricolorata	WP_038413878	--F----L----QL-A----INV	ITG	G-S-DL-R-----DKDARY--
	Pseudomonas acidophila	WP_096720801	--L----L-------A----VHI	ISG	G--VEQ-R-----THDERYQ-
	Pseudomonas typographi	WP_190417298	--F---------QL-G----INV	ITG	G-S-DL-R---Y-DKDARY--
	Pseudomonas taiwanensis	WP_027907789	--F----L----QL-R---SINI	ITG	G-S-DL-R-----DKDARY--
	Pseudomonas qingdaonensis	WP_100632639	--L---KL-----LL---L--HV	ISG	GN--EQRK---Y-DHDQRY--
	Pseudomonas bohemica	WP_110950776	--V----L----Q--E--L-VHI	ISG	GT-VDQ-R------HDERY--
	Pseudomonas thermotolerans	WP_017938772	-PL----L----Q-----L--NV	ISG	GS-IDQ-R-----DHDQRY--
	Pseudomonas graminis	WP_074888185	--V---------V-TG--I-VHI	ITG	G--QELRA--S-IGKDERY--
	Pseudomonas bohemica	WP_110950831	--V---------V-NG--T-VHI	ITG	G--QELRA--S-IGKDERY--
	Pseudomonas matsuisoli	WP_188981915	--L---KL---E-LL---L-VHI	ISG	GN-AEQRK---Y-NHDERY--
	Pseudomonas floridensis	WP_083186076	--V---------V-NG--T-VHI	ITG	G--QELRA--SYIGKDERY--
	Pseudomonas dryadis	WP_131174647	--L---KL-----LL---L--HV	ISG	GS-VEQRK---Y-DHDQRY--
	Pseudomonas citronellolis	WP_074982878	--L---KL---E-LL---L-VHI	ISG	GS-ADQRK---Y-DHDQRY--
	Pseudomonas humi	WP_058071687	--L---KL---E-LL---L-VHI	ISG	GS-ADQRK---Y-DHDQRY--
	Pseudomonas congelans	WP_054993551	--V---------V-NG--T-VHI	ITG	G--KELRA--SHIGKDERY--
	Pseudomonas denitrificans	WP_003097607	--L---KL---E-LL---L-VHI	ISG	GS-ADQRK---Y-DHDQRY--
	Pseudomonas aeruginosa	WP_003097607	--L---KL---E-LL---L-VHI	ISG	GS-ADQRK---Y-DHDQRY--
	Pseudomonas cerasi	WP_003434496	--V---------V-NG--T-VHI	ITG	G--KELRA--SHIGKDERY--
	Pseudomonas nitroreducens	WP_081754021	-PL----L----Q-----L--NV	VSG	GN-EDLAR---Y--HDRRY--
	Pseudomonas nitritireducens	WP_170858940	-PL----L----Q-----L--NV	VSG	GN-EDLAR---Y--HDRRY--
	Pseudomonas kuykendallii	WP_090224811	--L---KL---E-LL---L-VNV	ISG	GS-AEQRR-----SHDERY--
	Pseudomonas multiresinivorans	WP_169936376	-PL----L----Q-----L--NV	VSG	GN-EDLAR---Y--HDRRY--
	Pseudomonas oryzihabitans	WP_059314619	--L---KL-----LL---L-VHI	ISG	GS--EQRK---W-DHDQRY--
	Pseudomonas ovata	WP_109511925	--V---------T-NG--T-VHI	ITG	GN-QELRA--SHIDKDARY--
	Pseudomonas chlororaphis	WP_038632532	--L-----T---V-NG--T-VHI	ITG	G--RELRA--SYIDKDQRY--
	Pseudomonas amygdali	WP_044318896	--V---------V-NG--T-VHI	ITG	G--KELRA--SHIGKDERY--
	Pseudomonas savastanoi	WP_011169196	--V---------V-NG--T-VHI	ING	G--KELRA--SHIGKDERY--
	Pseudomonas meliae	WP_002554516	--V---------V-NG--T-VHI	ITG	G--KELRA--SHIGKDERY--
	Pseudomonas ficuserectae	WP_002554516	--V---------V-NG--T-VHI	ITG	G--KELRA--SHIGKDERY--
	Pseudomonas caricapapayae	WP_055008380	--V---------V-NG--T-VHI	ITG	G--KELRA--SHIGKDERY--
	Pseudomonas viridiflava	WP_058430928	--V---------V-NG--T-VHI	ITG	G--KELRA--SHIGKDERY--
	Pseudomonas delhiensis	WP_089390143	--L---KL---E-LL---L-VHI	ISG	GS-ADQRK---Y-DHDQRY--
	Pseudomonas syringae	WP_025389225	--V---------V-NG--T-VHI	ITG	G--KELRA--SHIGKDERY--
	Pseudomonas cannabina	WP_055001597	--V---------V-NG--T-VHI	ITG	G--KELRA--SHIGKDERY--
	Pseudomonas asturiensis	WP_073166180	--V---------V-NG--T-VHI	ITG	G--KELRA--SHIGKDERY--
	Pseudomonas cichorii	WP_025261230	--V---------V-NG--T-VHI	ITG	G--KELRA--SHIGKDERY--
	Pseudomonas punonensis	WP_073264686	-PL----L----Q--N--L--NV	ISG	GS-ADQ-R---W-NHDQRY--
	Pseudomonas abietaniphila	WP_074757711	--V----L----V-NG--T-VHI	ITG	G--QELRA--S-IGKDERY--
	Pseudomonas daroniae	WP_131192943	-SL----L----Q--G--L--NV	VSG	GN-EDL-R---Y-NHTQRY--
	Pseudomonas straminea	WP_093502510	-PL----L----Q--N--L--NV	ISG	GS-ADQ-R---W-NHDQRY--
	Pseudomonas caspiana	WP_087265202	--V---------V-NG--T-IHI	ITG	G--KELRA--SHIGKDERY--
	Pseudomonas kuykendallii	WP_090225200	-PL----L----Q--N--V--NV	ISG	GS-ADQRR---Y-DHDARY--
	Pseudomonas humi	WP_083280338	-PL----L----Q-----L--NV	VSG	GN-EDLAR---Y-DHDRRY--
	Pseudomonas delhiensis	WP_089390141	-PL----L----Q-----L--NV	VSG	GN-EDLAR---Y-DHDRRY--
	Pseudomonas mucidolens	WP_084378774	--L-----N---V-NG--T-VHI	ITG	G--RELRA--SYIGKDERY--
	Pseudomonas citronellolis	WP_083426808	-PL----L----Q-----L--NV	VSG	GN-EDLAR---Y-DHDRRY--
	Pseudomonas acidophila	WP_096722536	--L----L----QL-G--L-VHF	ISG	GS-TEQRR-----AHDERY--
	Pseudomonas ovata	WP_109511949	--L---KL-----LL---L-VHV	ISG	GS-AEQRK---S-DHDQRY--
	Pseudomonas yamanorum	WP_063031002	--L---------V-NG--T-VHI	ITG	G--RELRA--SHIDKDERY--
	Pseudomonas synxantha	WP_057022699	--L---------V-NG--T-VHI	ITG	G--NELRA--SHIGKDERY--
	Pseudomonas libanensis	WP_057011330	--L---------V-NG--T-VHI	ITG	G--NELRA--SHIGKDERY--
	Pseudomonas kitaguniensis	WP_152746431	--L-----T---V-NG--T-VHI	ITG	G--RELRA--SYIGKDERY--
	Pseudomonas cremoricolorata	WP_038413760	-PL----L----Q--A--L--NV	VSG	GN-EDLAR---Y-DHDQRY--
	Pseudomonas fluorescens	WP_053255590	--L---------V-NG--T-VHI	ITG	G--RELRA--SHIGKDERY--
	Pseudomonas antarctica	WP_064451880	--L---------V-NG--T-VHI	ITG	G--RELRA--SHIGKDERY--
	Pseudomonas reactans	WP_177004108	--L---------V-NG--T-VHI	ITG	G--RELRA--SHIGKDERY--
	Pseudomonas canadensis	WP_028616633	--L---------V-NG--T-VHI	ITG	G--RELRA--SHIGKDERY--
	Pseudomonas sivasensis	WP_181640550	--L---------V-NG--T-VHI	ITG	G--RELRA--SHIGKDERY--
	Pseudomonas lurida	WP_098466773	--L---------V-NG--T-VHI	ITG	G--RELRA--SHIGKDERY--
	Pseudomonas grimontii	WP_090403232	--L---------V-NG--T-VHI	ITG	G--RELRA--SHIGKDERY--
	Pseudomonas azotoformans	WP_061436381	--L---------V-NG--T-VHI	ITG	G--RELRA--SHIGKDERY--
	Pseudomonas simiae	WP_021492714	--L---------V-NG--T-VHI	ITG	G--RELRA--SHIGKDERY--
	Pseudomonas edaphica	WP_017137767	--L---------V-NG--T-VHI	ITG	G--RELRA--SHIGKDERY--
	Pseudomonas salomonii	WP_069785852	--L---------V-NG--T-VHI	ITG	G--RELRA--SHIGKDERY--
	Pseudomonas lactis	WP_057710446	--L---------V-NG--T-VHI	ITG	G--RELRA--SHIGKDERY--
	Pseudomonas haemolytica	WP_153872201	--L---------V-NG--T-VHI	ITG	G--KELRG--SHIGKDERY--
	Pseudomonas palleroniana	WP_090371628	--L---------V-NG--T-VHI	ITG	G--RELRA--SHIGKDERY--
	Pseudomonas orientalis	WP_057725288	--L---------V-NG--T-VHI	ITG	G--RELRA--SHIGKDERY--
	Pseudomonas ovata	WP_109512815	-PL----L----Q--N--LS-NV	ISG	GS-TDQ-R---W-DHDQRY--
	Pseudomonas thermotolerans	WP_017938774	--L---KL---ENLL---L-VHI	ISG	GS-AEQRK---Y-DHDQRY--
	Pseudomonas marginalis	WP_012724828	--L-----T---V-NG--T-VHI	ITG	G--RELRA--SHIGKDERY--
	Pseudomonas allii	WP_179028394	--L-----T---V-NG--T-VHI	ITG	G--RELRA--SHIGKDERY--
	Pseudomonas extremorientalis	WP_071490092	--L-----T---V-NG--T-VHI	ITG	G--RELRA--SHIGKDERY--
	Pseudomonas trivialis	WP_049712860	--L-----T---V-NG--T-VHI	ITG	G--RELRA--SHIGKDERY--
	Pseudomonas tolaasii	WP_016970492	--L-----T---V-NG--T-VHI	ITG	G--RELRA--SHIGKDERY--
	Pseudomonas kairouanensis	WP_135290325	--L-----T---V-NG--T-VHI	ITG	G--RELRA--SHIGKDERY--
	Pseudomonas nabeulensis	WP_135308474	--L-----T---V-NG--T-VHI	ITG	G--RELRA--SHIGKDERY--
	Pseudomonas cremoris	WP_185707009	--L-----T---V-NG--T-VHI	ITG	G--RELRA--SHIGKDERY--

Group	Species	Accession	Sequence
**Other *Pseudomonadacea***	*Pseudomonas fildesensis*	WP_048731405	--L-----N---V-NG--T-VHI ITG G--RELRA--SHIGKDERY--
	Pseudomonas costantinii	WP_071482492	--L-----N---V-NG--T-VHI ITG G--RELRA--SHIGKDERY--
	Pseudomonas cedrina	WP_076952545	--L-----S---V-NG--T-VHI ITG G--RELRA--SHIGKDERY--
**Other Bacteria**	*Klebsiella grimontii*	WP_049112194	--L---K----E-LLG--L-VHI ISG GN-AEQRR---Y-DHDQRY--
	Raoultella terrigena	WP_041146013	--L---K----E-LLG--L-VHI ISG GN-AEQRR---Y-NHDQRY--
	Klebsiella huaxiensis	WP_112213449	--L---K----E-LLG--L-VHI ISG GN-AEQRR---Y-DHDQRY--
	Klebsiella oxytoca	WP_014229688	--L---K----E-LLG--L-VHI ISG GN-AEQRR---Y-DHDQRY--
	Klebsiella aerogenes	WP_015705145	--L---K----E-LLG--L-VHI ISG GN-AEQRR---Y-DHDQRY--
	Enterobacter aerogenes	YP_004593638	--L---K----E-LLG--L-VHI ISG GN-AEQRR---Y-DHDQRY--
	Raoultella planticola	WP_032699564	--L---K----E-LLG--L-VHI ISG GN-AEQRR---Y-NHDQRY--
	Klebsiella variicola	WP_008804493	--L---K----E-LLG--L-VHI ISG GN-AEQRR---Y-DHDQRY--
	Klebsiella quasivariicola	WP_008804493	--L---K----E-LLG--L-VHI ISG GN-AEQRR---Y-DHDQRY--
	Klebsiella quasipneumoniae	WP_044523781	--L---K----E-LLG--L-VHI ISG GN-AEQRR---Y-DHDQRY--
	Klebsiella indica	WP_138358771	--L---K----E-LLG--L-VHI ISG GN-AEQRR---Y-DHDQRY--
	Klebsiella pneumoniae	YP_005227393	--L---K----E-LLG--L-VHI ISG GN-AEQRR---Y-DHDQRY--

Figure-S95

Partial sequence alignments of LLM class flavin-dependent oxidoreductase protein showing a 3aa Del (highlighted), which is specific for the genus *Azotobacter*.

			555		595
Genus *Azomonas* (2/2)	*Azomonas agilis*	WP_144570020	WLCHTLYFPGEKRVAKREVNRSPKTI	GEDLL	EYFKPKVRTY
	Azomonas macrocytogenes	WP_183167424	----------D---T--A------I-	DG---	----------
Other *Pseudomonadaceae*	*Atopomonas hussainii*	WP_071870343	----S------------A--FA---V		PA-E------
	Azotobacter beijerinckii	WP_090623108	-----------------A--FA---V		PA-E------
	Azotobacter chroococcum	TCL34636	-----------------A--FA---V		PA-E------
	Halopseudomonas pertucinogena	WP_188635250	----SM-H-QT-QLT--G--F----V		PA-E------
	Pseudomonas aeruginosa	WP_144148884	--------------S--S--FA---V		PA-E------
	Pseudomonas agarici	WP_017132988	-----------------A--F----V		PT-E------
	Pseudomonas akappageensis	WP_166366776	-----------------A--FA---V		PA-E------
	Pseudomonas alcaligenes	WP_187805654	----S------------A--FA---V		PA-E------
	Pseudomonas alkylphenolica	WP_038613120	-----------------A--FA---V		PA-E------
	Pseudomonas anguilliseptica	WP_090386126	----S--I------S--S--F----V		PA-E------
	Pseudomonas argentinensis	WP_074888370	----S------------D--F----V		PA-E------
	Pseudomonas asplenii	WP_090210869	-----------------A--F----V		PT-E------
	Pseudomonas batumici	WP_040065289	--------------T--A--F----V		PT-E------
	Pseudomonas bohemica	WP_110947223	-----------------A--FA---V		PA-E--I---
	Pseudomonas brassicacearum	WP_123346034	------F----------A--F----V		PT-E--I---
	Pseudomonas caspiana	WP_087274175	-----------------A--FA---V		PA-E--I---
	Pseudomonas chlororaphis	WP_009042788	-----------------A--F----V		PT-E------
	Pseudomonas cichorii	WP_221619488	----------D------A--F----V		PT-E--I---
	Pseudomonas citronellolis	KAF1069943	-----------------S--FA---V		PA-E------
	Pseudomonas corrugata	WP_208554863	-----------------A--F----V		PT-E------
	Pseudomonas daroniae	WP_131182203	----S---------T--D--F----V		PA-E------
	Pseudomonas defluvii	WP_065759453	-----------------A--FA---V		PA-E------
	Pseudomonas delhiensis	WP_089390390	-----------------A--FA---V		PA-E------
	Pseudomonas dryadis	WP_131174184	-----------------D--FA---V		PA-E------
	Pseudomonas entomophila	WP_060394216	-----------------A--FA---V		PA-E------
	Pseudomonas extremaustralis	WP_010564995	--------------S--A--F----V		PM-E--I---
	Pseudomonas flavescens	WP_084303385	----S---------T--D--F----V		PA-E------
	Pseudomonas floridensis	WP_083183600	----------D------A--F----V		PT-E--I---
	Pseudomonas fluorescens	VVO04961	--------------T--A--F----V		PT-E--I---
	Pseudomonas fluvialis	WP_184682732	-----------------S--FA---V		PA-E--I---
	Pseudomonas frederiksbergensis	URM27495	----------D------A--F----V		PT-E--I---
	Pseudomonas fulva	WP_196180141	-----------------A--FA---V		PA-E------
	Pseudomonas fuscovaginae	WP_054057169	-----------------A--F----V		PA-E------
	Pseudomonas graminis	WP_133771630	-----------------A--F----V		PT-E--I---
	Pseudomonas guangdongensis	WP_090214814	-----------------G--F----V		PA-E------
	Pseudomonas indica	WP_084333830	-----------------A--FA---V		PA-E------
	Pseudomonas japonica	WP_042123365	-----------------A--FA---V		PA-E------
	Pseudomonas jessenii	WP_115148234	-----------------A--F----V		PT-E--I---
	Pseudomonas knackmussii	WP_043251335	-----------------A--FA---V		PA-E------
	Pseudomonas lalkuanensis	WP_151133054	-----------------A--FA---V		PA-E------
	Pseudomonas laurentiana	WP_163938072	-----------------A--FA---V		PA-E------
	Pseudomonas lopnurensis	WP_193682245	-----------------A--FA---V		PA-E------
	Pseudomonas lutea	WP_037009388	----------D------A--F----V		PT-E--I---
	Pseudomonas luteola	WP_019365381	---------ED---T--A--F----V		PT-E------
	Pseudomonas massiliensis	WP_040262764	-----------------A--FA---V		PA-E------
	Pseudomonas mendocina	WP_147809751	----S------------D--FA---V		PA-E------
	Pseudomonas mohnii	WP_242070806	--------------S--A--F----V		PM-E--I---
	Pseudomonas moorei	WP_090324183	-----------------A--F----V		PT-E--I---
	Pseudomonas moraviensis	WP_095667150	-----------------A--F----V		PT-E------
	Pseudomonas nicosulfuronedens	WP_138525725	-----------------A--FA---V		PA-E------
	Pseudomonas nitrititolerans	HJE30365	--------------S--A--F----V		PT-E------
	Pseudomonas nitroreducens	WP_017518062	-----------------A--FA---V		PA-E------
	Pseudomonas oleovorans	PZQ31947	----S------------D--FA---V		PA-E------
	Pseudomonas oleovorans	PZQ31947	----S------------D--FA---V		PA-E------
	Pseudomonas oryzae	WP_090348633	-----------------G--F----V		PA-E------
	Pseudomonas oryzihabitans	WP_059314193	--------------S--D--FA---V		PM-E------
	Pseudomonas panipatensis	WP_090262221	-----------------S--FA---V		PA-E------
	Pseudomonas pharyngis	WP_236189681	-----------------A--F----V		PT-E------
	Pseudomonas protegens	PZP02503	-----------------A--F----V		PT-E------
	Pseudomonas protegens	WP_210457397	-----------------A--F----V		PT-E------
	Pseudomonas psychrotolerans	WP_145005058	--------------S--D--FA---V		PM-E------
	Pseudomonas punonensis	WP_073267514	----S------------D--F----V		PA-E------
	Pseudomonas putida	WP_033045243	-----------------A--F----V		PT-E--I---
	Pseudomonas reidholzensis	WP_119142977	-----------------A--FA---V		PA-E------
	Pseudomonas reinekei	WP_075948472	----------D------A--F----V		PT-E--I---
	Pseudomonas resinovorans	WP_077525313	--------------T--A--FA---V		PA-E--I---
	Pseudomonas sagittaria	WP_092429178	----S---------T--G--F----V		PA-E------
	Pseudomonas schmalbachii	WP_208312038	-----------------A--FA---V		PA-E------
	Pseudomonas seleniipraecipitan	WP_070883125	----S---------T--D--F----V		PA-E------
	Pseudomonas thermotolerans	WP_017939312	-----------------A--FA---V		PA-E------
	Pseudomonas tructae	WP_130263894	-----------------A--FA---V		PA-E------
	Stutzerimonas stutzeri	MCQ4256486	-----------------A--F----V		PT-E------
	Stutzerimonas xanthomarina	WP_073299505	-----------------A--F----V		PT-E------
Other Bacteria	*Acinetobacter baumannii*	SST09293	--------------S--S---A---V		PA-E------
	Cellvibrio zantedeschiae	WP_189416251	----S--F-------------K-H-M		-A-Q--K---
	Enterobacter cloacae	SAJ25143	--------------S--S---A---V		PA-E------
	Escherichia coli	MBE0758292	--------------S--S---A---V		PA-E------
	Priestia aryabhattai	QPN43882	-----------------G---A---V		PA-E------
	Pseudomaricurvus alkylphenolicus	WP_166993965	----SM--------G--A---A---M		-A-E------
	Sessilibacter corallicola	WP_233088707	----S-F----------A--FA---V		-R-E------
	Streptococcus pneumoniae	CJL72534	----------D------A---A---V		PA-E------
	Teredinibacter haidensis	WP_075187927	----SM--------G--D--FA---M		-A-E------
	Teredinibacter purpureus	WP_045859240	----SM--------T--D--FT---M		-A-E------
	Teredinibacter turnerae	WP_015819918	----SM--------G--D--FA---M		-A-E------

Figure-S96

Partial sequence alignments of Succinate dehydrogenase flavoprotein subunit protein showing a 5aa ins (highlighted), which is specific for the genus *Azomonas*.

			790		819
Genus *Azomonas* (2/2)	*Azomonas macrocytogenes*	WP_183165886	YLDNFILYQAGSGA		NMTSISLKDVLSALLI
	Azomonas agilis	WP_144570721	-----T--EF-A--		TA-A---R-F-A---M
Other *Pseudomonadaceae*	*Azotobacter salinestris*	WP_152387354	----IV--EFA-E-	AGAA	SLVP---R---A----
	Azotobacter vinelandii	WP_012702992	----IV--EFTG-T	GATA	SLVP---R-L-A----
	Pseudomonas aeruginosa	WP_126644214	----VT---YTA-T	GEAA	TSVP---R-F-A--A-
	Pseudomonas atacamensis	WP_206421179	----IT--EYT--T	GANM	S-VP--IG-M-G--I-
	Pseudomonas baetica	WP_100846481	----IT--EYT--T	GANM	S-VP--IG-M-G--I-
	Pseudomonas botevensis	WP_217832012	----IT--EYT--T	GANM	S-VP--IG-M-G--I-
	Pseudomonas brassicacearum	WP_025211473	----IT--EYT--T	GANM	S-VP--IG-M-G--I-
	Pseudomonas bubulae	WP_235572250	----IT--EYT--T	GANM	S-VP--IG--IG--V-
	Pseudomonas chlororaphis	WP_009041854	----IT--EYT--T	GANM	S-VP--IG-M-G--I-
	Pseudomonas deceptionensis	WP_048360394	----IT--EYT--S	GANM	S-VP--IG--IG--V-
	Pseudomonas endophytica	WP_055104858	-----T--EYT--T	GANM	S-VP--IG--VG--V-
	Pseudomonas farris	WP_217855454	----IT--EYT--T	GANM	S-VP--IG-M-G--I-
	Pseudomonas fluorescens	CAB1400399	----IT--EYT--T	GANM	S-VP--IG-M-G--I-
	Pseudomonas fragi	WP_016783235	----IT--EYT--T	GANM	S-VP--IG--IG--V-
	Pseudomonas frederiksbergensis	MCE6978125	----IT--EYT--T	GANM	S-VP--IG-M-G--I-
	Pseudomonas granadensis	WP_090280523	----IT--EYT--T	GANM	S-VP--IG-M-G--I-
	Pseudomonas hamedanensis	WP_186549453	----IT--EYT--T	GANM	S-VP--IG-M-G--I-
	Pseudomonas helleri	WP_048366665	----IT--EYT--T	GANM	S-VP--IG---G--I-
	Pseudomonas helmanticensis	WP_134175897	----IT--EYT--T	GANM	S-VP--IG-M-G--I-
	Pseudomonas indica	MBU3059894	----IV--EFT--S	GDAV	SS-P---R-L-G----
	Pseudomonas jinjuensis	WP_084314535	----VT---FT--S	GEAA	TTSP---G-L-T--V-
	Pseudomonas kilonensis	WP_253471164	----IT--EYT--T	GANM	S-VP--IG-M-G--I-
	Pseudomonas koreensis	WP_129998234	----IT--EYT--T	GANM	S-VP--IG-M-G--I-
	Pseudomonas lini	WP_050681738	----IT--EYT--T	GANM	S-VP--IG-M-G--I-
	Pseudomonas lopnurensis	WP_193683014	----VA--EFT--S	GDAL	TTST---N-LIG--A-
	Pseudomonas lundensis	WP_047291520	----IT--EYT--T	GANV	S-VP--IG-M-G--I-
	Pseudomonas mandelii	WP_253546898	----IT--EYT--T	GANM	S-VP--IG-M-G--I-
	Pseudomonas migulae	WP_084320979	----IT--EYT--T	GANM	S-VP--IG-M-G--I-
	Pseudomonas moraviensis	WP_065615464	----IT--EYT--T	GANM	S-VP--IG-M-G--I-
	Pseudomonas nicosulfuronedens	WP_138524002	----VT---YT--T	GDAA	ST-P---S-F-M--V-
	Pseudomonas nitroreducens	WP_253446057	----VT---YT--T	GDAA	ST-P---S-F-M--V-
	Pseudomonas ogarae	WP_014336227	----IT--EYT--T	GANM	S-VP--IG-M-G--I-
	Pseudomonas oligotrophica	WP_237255739	----IT--EYT--S	GETL	TTSA---NNL-G----
	Pseudomonas prosekii	SDS72730	----IT--EYT--T	GANM	S-VP--IG-M-G--I-
	Pseudomonas psychrophila	WP_019825338	----IT--EYT--S	GANM	S-VP--IG--IG--V-
	Pseudomonas putida	WP_075802112	--N--T--EYT--T	GATA	S-VP---G-L-G--V-
	Pseudomonas rustica	WP_212549894	----IT--EYT--T	GANM	S-VP--IG-M-G--I-
	Pseudomonas saudiphocaensis	WP_037024004	-----A--EFT--S	GDAL	TVSI---N-L-G--A-
	Pseudomonas silesiensis	WP_064675544	----IT--EYT--T	GANM	S-VP--IG-M-G--I-
	Pseudomonas siliginis	WP_217851102	----IT--EYT--T	GANM	S-VP--IG-M-G--I-
	Pseudomonas syringae	WP_052965101	----IT--EYT--T	GANM	S-VP--IG-M-G--I-
	Pseudomonas taetrolens	WP_048382840	-----T--EYT--S	GATM	S-VP--MG-MIG--V-
	Pseudomonas tehranensis	WP_186655428	----IT--EYT--T	GANM	S-VP--IG-M-G--I-
	Pseudomonas thivervalensis	WP_053181370	----IT--EYT--T	GANM	S-VP--IG-M-G--I-
	Pseudomonas triticicola	WP_217864562	----IT--EYT--T	GANM	S-VP--IG-M-G--I-
	Pseudomonas versuta	WP_060695890	----IT--EYT--T	GANM	S-VP--IG--IG--I-
	Pseudomonas versuta	WP_073514944	----IT--EYT--T	GANM	S-VP--IG--IG--I-
	Pseudomonas viciae	WP_135843356	----IT--EYT--T	GANM	S-VP--IG-M-G--I-
	Pseudomonas viridiflava	WP_122508524	----IT--EYT--T	GANM	S-VP--IG-M-G--I-
	Pseudomonas weihenstephanensis	WP_048364512	----IT--EYT--T	GANM	S-VP--IG--IG--V-
	Pseudomonas xionganensis	WP_160343554	----IT--EYS--T	GETA	SLVP---S---G--I-
	Pseudomonas zarinae	WP_186651245	----IT--EYT--T	GANM	S-VP--IG-M-G--I-
	Stutzerimonas kirkiae	WP_131185758	----VT--EFT--S	GENA	VNKP---N-L-G----
	Stutzerimonas stutzeri	MBF6622533	----VA--EFT--S	GDAL	TTSA---N-L-G--A-
Other Bacteria	*Thauera aromatica*	WP_247736540	----IV---FSASD	GS	SG-P---R---G----
	Thauera chlorobenzoica	WP_075146619	----IV---FTAAD	GS	SG-P---R---G----

Figure- S97

Partial sequence alignments of Mechanosensitive channel MscK protein showing a 4aa Del (highlighted), which is specific for the genus *Azomonas*.

Group	Species	Accession	73		110
Genus *Azomonas* (2/2)	*Azomonas agilis*	WP_144571310	NKQSTQSLVINASQDT		RPLLEQIGQILNGYPCTLELIL
	Azomonas macrocytogenes	WP_183165877	---AV--I--TPDGRE		AAV-K-VALA-GCDGHAVGDVM
Other *Pseudomonadaceae*	*Pseudomonas aeruginosa*	WP_251738048	--DAVL-V--S-RTAA	DE	TS--R-VA-G-SINQAS--A--
	Pseudomonas arsenicoxydans	WP_208670434	----V--V-VS-RGAG	DS	AGV-R-VA-A--VEQAEIGV--
	Pseudomonas batumici	WP_040063674	----V--V-VS-RGAG	DA	AGV-R--A-A--VAQADIRE--
	Pseudomonas caspiana	WP_087271143	----V--V--S-RGA-	DA	AG--R-VA-A--VAQPEIQA--
	Pseudomonas chlororaphis	WP_106696326	----V--V-VS-RGAG	DA	AGV-R-VA-A--VEQAEIGT--
	Pseudomonas cremoricolorata	WP_028693354	----V--V-VS-RGAA	DA	ASV-G-VA-S--VAKAEMQA--
	Pseudomonas entomophila	WP_011531756	----V--V-VS-RGAS	DA	ASV-G-VA-S--VAQPEVQA--
	Pseudomonas fluorescens	WP_150802243	----V--V-VS-RGAG	DS	AGV-R-VA-A--VAQAEIGA--
	Pseudomonas foliumensis	WP_187521115	----V--V-VS-RGAA	DA	AG--R-VA-A--VAQPEMRA--
	Pseudomonas frederiksbergensis	WP_123402538	----V--V-VS-RGAG	DA	AGV-R--A-A--VAQAEIGA--
	Pseudomonas fuscovaginae	WP_054062079	----V--V-VS-RGAG	DA	AGV-Y--A-A--VAEADVSS--
	Pseudomonas gingeri	WP_042935609	----V--V-VS-RGAG	DA	AGV-R--A-A--VAQADIGS--
	Pseudomonas guariconensis	WP_102081436	----V--V-VS-RGAG	DA	ASV-A-VA-A--VANAEVQT--
	Pseudomonas helleri	WP_153429317	----V--V-VS-RGAG	DA	AGI-R-VA-T--VARPEVDA--
	Pseudomonas lalkuanensis	WP_151137774	---AV----VS-RNSA	EA	GG--R--A-G--IAQADVRS--
	Pseudomonas lopnurensis	WP_193679249	----V--V--TPQATI	DA	SA--V--A-A--SQDADFDAVM
	Pseudomonas mandelii	WP_094469512	----V--V-VS-RGAG	DA	AGV-R-VA-A--VAQAEVNA--
	Pseudomonas mosselii	WP_028689107	----V--V-VS-RGAS	DA	ASV-G-VA-S--VAQPEVQA--
	Pseudomonas nitrititolerans	HJE27919	---AVM-V--TPQGTA	DS	SAV-Q----A--IQHADFDGVM
	Pseudomonas oligotrophica	WP_237256241	----V--I--TPQGSL	DA	SA--Q----A-GVT-ADYDSVM
	Pseudomonas paraversuta	WP_203008946	----V--V-VS-RGAG	DA	AGI-R-VA-T--AARADVGA--
	Pseudomonas peradeniyensis	WP_186732462	----V--V-VS-RGAS	DA	ASV-G-VA-S--VAQPEVQA--
	Pseudomonas protegens	WP_110595397	----V--V-VS-RGAS	DA	AGV-H-VA-A--VAQAEIGA--
	Pseudomonas putida	WP_198744924	----V--V-VS-RGAS	DA	ASV-G-VA-S--VAQPEVQA--
	Pseudomonas resinovorans	WP_016495116	---AV-----S-RNSA	EA	GG--R-VA-G--VAQAD-RS--
	Pseudomonas saxonica	WP_122785189	----V--V-VS-RGGM	DA	AGI-R-VA-T--AASADVGA--
	Pseudomonas songnenensis	WP_126189120	----V--V--TPQGTM	DA	SV--A--A-A--SPVADFDG-M
	Pseudomonas stutzeri NF13	EMD99905	---AV--V--TPQATM	DA	SA--A--A-A--SQDADFDGVM
	Pseudomonas syringae	MCQ2994864	----V--V-VS-RGAA	DA	SG--R-VA-A--VAQPEMRA--
	Pseudomonas umsongensis	WP_248686820	---TV----VS-RGAG	DS	AG--Q-VA-A--VAQAEIGA--
	Pseudomonas urmiensis	WP_186556573	----V--V-VS-RGAS	DA	ASV-G-VA-A-GVAQAEVQA--
	Pseudomonas vancouverensis	WP_093227464	----V--V-VS-RGAG	DA	AGV-R-VA-A--VAQADIDA--
	Pseudomonas vanderleydeniana	WP_186688867	----V--V-VS-RGAG	DA	AGV-Y--A-A--VAEADVSS--
	Pseudomonas versuta	WP_060695970	----V--V-VS-RGAG	DA	AGI-R-VA-T--AARADVGA--
	Pseudomonas viridiflava	WP_122664157	----V--V--S-RGAS	DA	SG--R-VA-A--VAQPEMHA--
	Pseudomonas wayambapalatensis	QXI43629	----V--V-VS-RGAS	DA	ASV-G-VA-A--VAQPEVQA--
	Stutzerimonas balearica	MBC7198763	----V--V--TP-GSL	DA	SA--Q--A-AVSAPVADF-A-M
	Stutzerimonas chloritidismutan	WP_221104124	---AV--V--TPQATM	DA	SA--A--A-A--SQEADFDGVM
	Stutzerimonas frequens	WP_110778194	---AV--V--TPQDT-	DS	TA-IA-VA-A--SREADF-GVM
	Stutzerimonas kunmingensis	WP_234173707	---AV--V--TPQATM	DA	SA--A--A-A--SQEADFDGVM
	Stutzerimonas stutzeri	AKN25553	---AV--V-VTPQSTM	DA	SA--A--A-A--SQDADFDG-M
	Stutzerimonas stutzeri	WP_254162353	---AV--V-VTPQSTM	DA	SA--A--A-A--SQDADFDG-M
	Stutzerimonas xanthomarina	CEG53788	---AV--V--TPQATM	DA	SA--A--A-A--SQEADFDGVM
Other Bacteria	*Staphylococcus aureus*	NGV52199	--DAVL-V--S-RTAA	DE	TS--R-VA-G-SINQAS--A--
	Streptococcus pneumoniae	CJK73033	---AV--V-VTPQSTM	DA	SA--A--A-A--SQDADFDG-M
	Paucimonas lemoignei	SQF94426	----V--V-VS-RGAS	DA	AGV-R-VA-A--VAQAEMQA--
	Priestia aryabhattai	QPN45856	----V--V-VS-RGAS	DA	ASV-G-VA-A-DVA-AEVQA--

Figure- S98

Partial sequence alignments of SPOR domain-containing protein showing a 2aa Del (highlighted), which is specific for the genus *Azomonas*.

Group	Species	Accession	153		185
Genus *Azomonas* (2/2)	*Azomonas macrocytogenes*	WP_183165719	WLYPRLCTLYGTPLGQD		GQPQRMVILGMGKLGA
	Azomonas agilis	WP_144571373	---T-F-AQ--I-RNIQ		--A-HL-V--------
Other *Pseudomonadaceae*	*Pseudomonas aeruginosa*	WP_247247670	--HR-Q-EQF---I-RR	S	-E-----V--------
	Pseudomonas argentinensis	WP_070885134	-----Y-EQF---V-RR	S	-E--H-----------
	Pseudomonas composti	WP_037003476	-----H--QF---V-RR	S	----H-----------
	Pseudomonas dryadis	WP_131198771	---S-H-QQF---V--R	S	----Q-----------
	Pseudomonas entomophila	WP_181099793	-----H-QQF---I-RS		-A--H--V--------
	Pseudomonas farsensis	WP_186536869	-----H-QQF---I--R	S	----H--V--------
	Pseudomonas flavescens	WP_084308605	-----H-EQF---V-GR	S	-E--H-----------
	Pseudomonas fluorescens	WP_150776997	-----H-QQF---I-HR	S	----H--V--------
	Pseudomonas fulva	WP_042553896	-----H-EQF---V-RR	T	-E--H-----------
	Pseudomonas huaxiensis	WP_110972448	-------QQF---T-HR	S	----Q--V--------
	Pseudomonas japonica	WP_042124061	-------EQF---T-SR	S	----Q--V--------
	Pseudomonas juntendi	WP_182365235	-----H-QQF---I-NR	S	----H--V--------
	Pseudomonas lalucatii	WP_213639572	-----H-AQF---I-RR	S	----H-----------
	Pseudomonas mendocina	WP_143502429	-----H--QF---V-RR	T	----H-----------
	Pseudomonas mendocina	WP_143504464	-----H--QF---V-RR	S	----H-----------
	Pseudomonas oleovorans	WP_119692061	-------AQF---V-RR	S	----H-----------
	Pseudomonas putida	SUD73004	-----H-QQF---I-NR	S	----H--V--------
	Pseudomonas sediminis	WP_179545950	-----H--QF---V-RR	S	----H-----------
	Pseudomonas thermotolerans	WP_027896696	-----H-EQF---I-RR	S	----HL-V--------
	Pseudomonas urmiensis	WP_186552740	-----H-QQF---I-NR	S	-E--H--V--------
	Pseudomonas vranovensis	WP_123565324	-----H-QQF---I-HR	S	----H--V--------
	Pseudomonas yangonensis	WP_161864653	-----H--QF---V-RR	T	----H-----------

Figure- S99

Partial sequence alignments of a protein Bifunctional [glutamate--ammonia ligase]-adenylyl-L-tyrosine phosphorylase/[glutamate--ammonia-ligase] adenylyltransferase showing a 1aa Del (highlighted), which is specific for the genus *Azomonas*.

Group	Species	Accession	366		398
Genus *Azomonas* (2/2)	*Azomonas agilis*	WP_144571471	GVEAAIDLAGVVAHVTLLTNSV		LKADAVLQRKL
	Azomonas macrocytogenes	WP_183167175	-------------------HNT		-R---------
Other *Pseudomonadaceae*	*Pseudomonas aeruginosa*	WP_247072797	----------I-------EFDS	K	-R---------
	Pseudomonas citronellolis	WP_116425805	----------I-------EFDS	K	-R---------
	Pseudomonas coronafaciens	RMS28273	----------I-S-----EFD-	Q	-R---------
	Pseudomonas delhiensis	WP_089392528	----------I-------EFDS	K	-R---------
	Pseudomonas fluorescens	WP_126450832	----------I-------EFDS	K	-R---------
	Pseudomonas frederiksbergensis	WP_185022293	----------I-S-----EFD-	Q	-R---------
	Pseudomonas nitritireducens	WP_184586199	----------I-------EFDS	K	-R---------
	Pseudomonas panipatensis	WP_090268273	----------I-------EFDS	K	-R---------
	Pseudomonas syringae	EPM80925	----------I-S-----EFD-	Q	-R---------
	Pseudomonas viridiflava	WP_163021829	----------I-S-----EFD-	Q	-R---------
	Azotobacter beijerinckii	WP_090623986	----------I--------HGE	K	-R---------
	Azotobacter chroococcum	WP_198867940	----------I--------HGE	K	-R---------
	Azotobacter salinestris	WP_152386028	----------I--------HGE	A	-R---------
	Azotobacter vinelandii	WP_012701199	-------------------HGE	A	-R---------
	Stutzerimonas azotifigens	WP_181073273	----------I------IEFDS	Q	-R------K--
	Stutzerimonas degradans	WP_008567747	----------I-------EFGE	E	-R---------
	Stutzerimonas stutzeri	WP_037000184	----------I-------EFGE	D	-R---------
Other Bacteria	*Azospirillum soli*	WP_209877979	----------I------IEFDS	V	-R---------
	Candidatus Accumulibacter phos	MBL8424953	------------------EFDS	I	-R---I--K--
	Cellvibrio japonicus	WP_012486058	----------I-------EFDS	T	-R---------
	Escherichia coli	WP_192521800	----------I-------EFDS	K	-R---------
	Klebsiella pneumoniae	WP_180355290	----------I-------EFDS	K	-R---------
	Lacisediminimonas profundi	WP_151448822	----------I-------EFDS	Q	-R---------
	Listeria monocytogenes	WP_207144793	----------I-------EFDS	K	-R---------
	Propionivibrio sp.	MBP8279112	----------I-------EFDS	I	-R------N--
	Quisquiliibacterium transsilva	WP_183970373	------------------EFDS	Q	-R---------
	Rhodococcus gordoniae	WP_255161706	-----------------IEFDS	V	-R--E------
	Rhodococcus pyridinivorans	WP_024102019	-----------------IEFDS	V	-R--E------
	Rhodococcus rhodochrous	WP_192378580	-----------------IEFDS	V	-R--E------
	Salmonella enterica	MCC1705884	----------I-S-----EFDS	K	-R---------
	Xanthomonas citri	MBD4391472	----------I-------EFDS	K	-R---------

Figure- S100

Partial sequence alignments of a protein Alkyl hydroperoxide reductase subunit F showing a 1aa Del (highlighted), which is specific for the genus *Azomonas*.
